# Integrated analysis identified core signal pathways and hypoxic characteristics of human glioblastoma

**DOI:** 10.1111/jcmm.14507

**Published:** 2019-07-07

**Authors:** Yixing Gao, Erlong Zhang, Bao Liu, Kai Zhou, Shu He, Lan Feng, Gang Wu, Mianfu Cao, Haibo Wu, Youhong Cui, Xia Zhang, Xindong Liu, Yan Wang, Yuqi Gao, Xiuwu Bian

**Affiliations:** ^1^ Institute of Pathology and Southwest Cancer Center Southwest Hospital, Army Medical University (Third Military Medical University), and Key Laboratory of Tumor Immunopathology, Ministry of Education of China Chongqing China; ^2^ Institute of Medicine and Equipment for High Altitude Region, College of High Altitude Military Medicine Army Medical University, and Key Laboratory of Extreme Environmental Medicine, Ministry of Education of China Chongqing China; ^3^ Collaborative Innovation Center for Cancer Medicine Sun Yat‐Sen University Guangzhou China

**Keywords:** core signal pathways, glioblastoma, hypoxia, transcriptome

## Abstract

As a hallmark for glioblastoma (GBM), high heterogeneity causes a variety of phenotypes and therapeutic responses among GBM patients, and it contributes to treatment failure. Moreover, hypoxia is a predominant feature of GBM and contributes greatly to its phenotype. To analyse the landscape of gene expression and hypoxic characteristics of GBM cells and their clinical significance in GBM patients, we performed transcriptome analysis of the GBM cell line U87‐MG and the normal glial cell line HEB under normoxia and hypoxia conditions, with the results of which were analysed using established gene ontology databases as well as The Cancer Genome Atlas and the Cancer Cell Line Encyclopedia. We revealed core signal pathways, including inflammation, angiogenesis and migration, and for the first time mapped the components of the toll‐like receptor 6 pathway in GBM cells. Moreover, by investigating the signal pathways involved in homoeostasis, proliferation and adenosine triphosphate metabolism, the critical response of GBM to hypoxia was clarified. Experiments with cell lines, patient serum and tissue identified IL1B, CSF3 and TIMP1 as potential plasma markers and VIM, STC1, TGFB1 and HMOX1 as potential biopsy markers for GBM. In conclusion, our study provided a comprehensive understanding for signal pathways and hypoxic characteristics of GBM and identified new biomarkers for GBM patients.

## 1 INTRODUCTION

Glioblastoma (GBM) is the most prevalent kinds of brain malignancy, and the average survival is around 1 year with the 5‐year survival rate less than 10%. The tumour recurrence after standard therapies is almost inevitable in GBM patients, and it ultimately results in the death of patients.^1^, ^2^ The main reason is the intra‐ and inter‐tumoural heterogeneity,^3^, ^4^ the complexities of which obscure the mechanisms underlying GBM tumourigenesis and cause the difficulty in choosing therapeutic targets for GBM patients. With the progression of sequencing technology, genome‐wide profiling of cancers, such as The Cancer Genome Atlas (TCGA)^5^ and The Cancer Cell Line Encyclopedia (CCLE),^6^ provides deep insights into the molecular basis of tumour initiation and progression.^7^ Moreover, transcriptome‐based profiling has clustered GBM patients into four molecular subtypes (proneural, neural, classical and mesenchymal), with inherent differences in responses to chemo‐ and radiotherapies.^8^ Genomic analyses on GBM have also described a panel of critical signalling pathways, including cell cycle checkpoint, apoptosis, TGF‐β, EGFR, PI3K/AKT, Rb, p53, NF‐κB and Notch signalling pathways.^5^, ^9^ Therefore, genome‐wide studies, including transcriptome profiling, are powerful tools to identify core signal pathways in cancers, which could be pharmacologically targeted.^4^ Owing to the heterogeneity of GBM, there are generally multiple important signalling pathways in different individual cases; the strategies used in most of the current databases are mainly derived from analyses of cohorts with large number of patients. Integrated analysis of established databases and transcriptomic profiling from GBM cells could not only advance the understanding of pathophysiological signal pathways and identify therapeutic targets for GBM development, but also provide a new analytical approach to support targeted tumour therapy.

To this end, we utilized U87‐MG (GBM cell line) and HEB (normal brain glial cell line) as models. For preclinical studies, cancer cell lines are good compromises for deciphering tumourigenesis mechanisms and evaluating drug effects. Large‐scale cell line panels are extensively used for drug screening and omics data generation.^10^ In a screen of 479 cancer cell lines of 36 different tumour types, tumour cell type‐ or lineage‐specific molecular signatures were identified as effective predictors of responses to several clinically relevant compounds.^6^ Although the DNA profile of the U87‐MG cells was found to be different from that of the original cells, it was likely a bona fide human GBM cell line of unknown origin.^11^ Many studies have successfully illustrated the pathogenesis of GBM and the pharmacological function of new pharmaceutical products on GBM with the U87‐MG cell line in recent years.^12^, ^13^ In this study, we developed a concise and reliable analysis through combining our transcriptome analyses on U87‐MG and HEB cells with established databases and in vitro and ex vivo experiments, which successfully revealed a set of core pathways in GBM cells under normoxic and hypoxic conditions.

## 2 MATERIALS AND METHODS

### 2.1 Cell culture and hypoxic treatment

The human GBM cell lines U87‐MG and LN229 were obtained from the American Type Culture Collection. The HEB cell line was generously provided by Professor Guang‐Mei Yan (Department of Pharmacology, Sun Yat‐Sen University, Guangzhou, China).^14^ The human astrocyte cell line HA was obtained from ScienCell Research Laboratories (ScienCell, Carlsbad, CA, USA). Cells were grown in Astrocyte Medium and maintained following the instructions. The primary human glioma cells 091214 were obtained from the specimen of a glioma patient.^15^ These cells were incubated in DMEM supplemented with 10% foetal bovine serum, 2 mmol/L l‐glutamine, 100 U/mL penicillin and 100 μg/mL streptomycin for 24 hours at normoxia (21% O_2_) followed by 24 hours at normoxia or hypoxia (1% O_2_) in hypoxic chambers (Thermo Scientific), separately.

### 2.2 mRNA‐Seq library preparation and sequencing

Total RNA was extracted with Trizol reagent and quantified using a Nanodrop ND‐1000 spectrophotometer. RNA integrity was verified on an Agilent 2100 Bioanalyzer. Illumina mRNA‐seq libraries were prepared using the TruSeq RNA kit using 200 ng of total RNA. The library was sequenced on an IlluminaHiSeq^™^ 2000 sequencing machine. RNA‐seq reads were mapped against the human genome build hg19 using Bowtie2 (version 2.2.4). More details of the RNA‐seq analyses were provided in Figure Doc S1. The raw data have been deposited to Gene Expression Omnibus under accession number GSE77307.

### 2.3 Enzyme‐linked immunosorbent assay

Plasma samples were collected from 13 GBM patients and 13 healthy persons in accordance with the protocols approved by the Institutional Review Board at Army Medical University, and written informed consent was obtained from all patients at the time of enrolment in Southwest Hospital, Army Medical University. Enzyme‐linked immnuosorbent assays were performed to measure IL1B, CSF3 and TIMP‐1, with commercially available kits (RayBiotech, ELH‐IL1b, ELH‐GCSF‐1, ELH‐TIMP1‐1). The information of the patients was listed in Table S1.

### 2.4 Protein extraction and Western blot

Whole cell lysates were obtained by resuspending cell pellets in RIPA buffer (Beyotime, P0013E) with a freshly added protease inhibitor tablet (Thermo Scientific, 88265). Western blot analyses were performed with anti‐β‐actin (Kangcheng, KC‐5A08), anti‐VIM (R&D, MAB2958), anti‐STC‐1 (Santa Cruz, sc‐14346), anti‐HMOX1 (Abcam, ab13243) and anti‐TGFB1 (Abcam, ab66043) antibodies.

### 2.5 Immunohistochemistry

Glioblastoma tissues were surgically obtained from 10 patients from Southwest Hospital, Army Medical University between 2009 and 2012 in accordance with the protocols approved by the Institutional Review Board at Army Medical University, and written informed consent was obtained from all patients at the time of enrolment in Southwest Hospital, Army Medical University, according to the guidelines of the Research Ethics Committees of Southwest Hospital, Army Medical University. Table S2 showed the main clinicopathological information of the GBM patients. Classification of the GBM cases was determined according to the criteria of the World Health Organization 2007. Immunohistochemistry (IHC) staining was performed on the paraffin sections of GBM tissues. The whole process was conducted using the Dako REAL EnVision Detection System according to the manufacturer's instructions. The protein abundance of VIM, STC1, TGFB1 and HMOX1 was detected through incubation with the primary antibodies of anti‐human VIM, STC‐1, HMOX1 and TGFB1, respectively, overnight at 4°C. Then, the corresponding polyclonal antimouse or anti‐rabbit secondary antibodies were added and incubated at 37°C for 30 minutes. The tissue sections were stained with diaminobenzidine as a substrate for colour development and counterstained with haematoxylin. Positive and negative controls were included in each immunohistochemical reaction.

### 2.6 cDNA synthesis and quantitative PCR

RNA from cells was isolated using the Total RNA Kit I (Takara, R6834‐02) according to the manufacturer's protocol. cDNA was synthesized using the PrimeScript RT reagent kit (Takara, RR047A) with random primers for RT priming. quantitative PCR (qPCR) was performed using SYBR Green (Bio‐Rad, RR820A) according to the manufacturer's instructions. PCR primers were listed in Table S3.

### 2.7 TCGA analysis

Bioinformatics analysis of TCGA data from the cBioPortal for Cancer Genomics (http://www.cbioportal.org) was performed to examine gene expression in GBM. The association of gene expression with survival was analysed using GEPIA (http://gepia.cancer-pku.cn/index.html).

### 2.8 Statistical analyses

Statistical analyses were performed using SPSS 13.0. For all tests, statistical significance was defined as *P* < 0.05 using independent *t* tests.

## 3 RESULTS

### 3.1 Biological process‐based categorization of up‐regulated genes in U87‐MG cells

**Figure 1:**
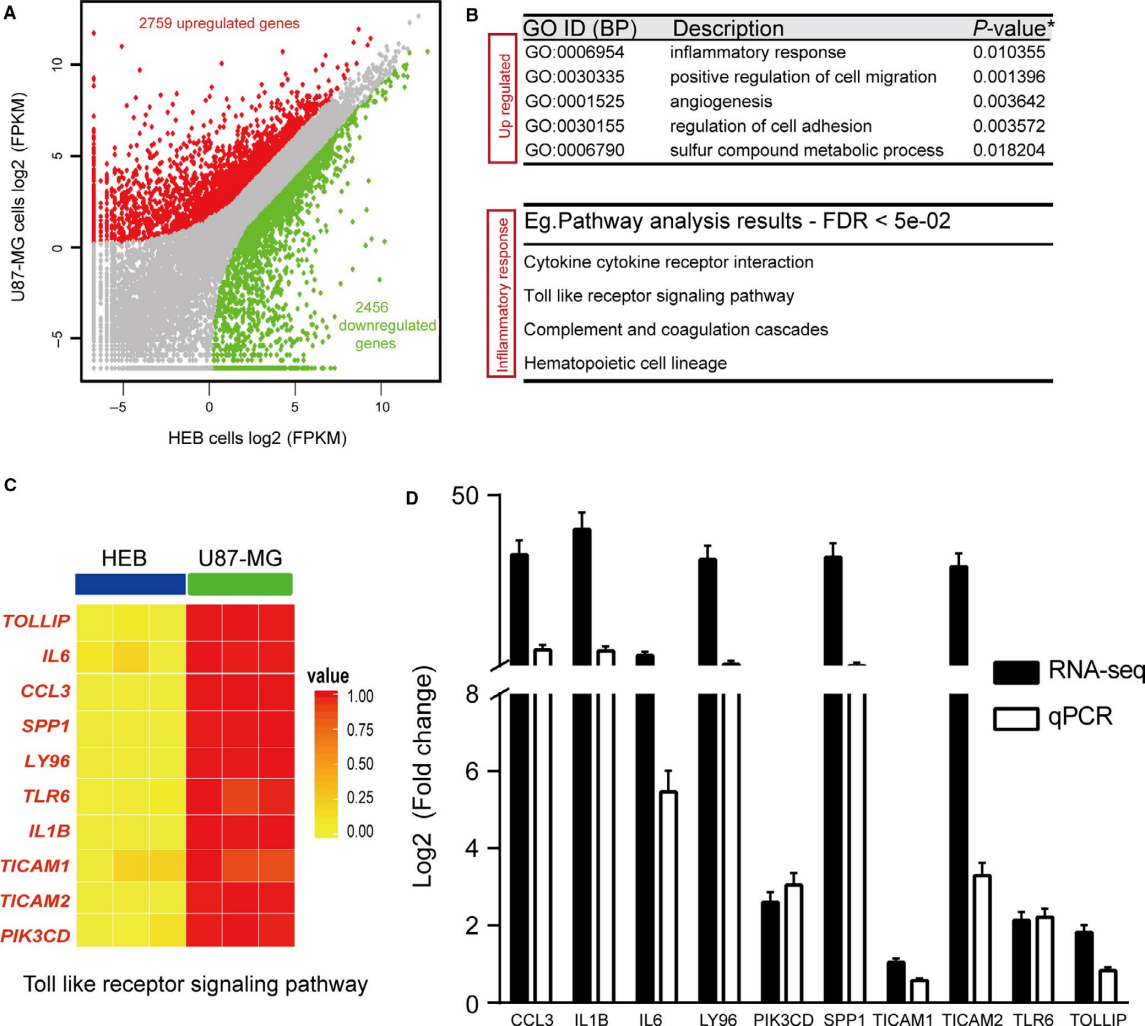
The landscape of transcriptomic alterations in U87‐MG cells vs HEB cells. A, The scatter plot description of the number of up‐regulated (red) and down‐regulated (blue) genes in U87‐MG cells compared to HEB cells. B, Gene ontology biological process subcategories (GO BP) for up‐regulated genes in U87‐MG cells vs HEB cells. The major signal pathways related with inflammation response for up‐regulated genes in U87‐MG cells vs HEB cells. The *P* values were corrected for multiple testing by the Benjamini and Hochberg procedure. C, Heat map of up‐regulated genes involved in the Toll‐like receptor (TLR) signal pathway in U87‐MG cells vs HEB cells. D, qPCR assay verified genes involved in TLR6 signal pathway from the RNA‐seq data. The ordinate represented the log_2_ ratio of gene expression in U87‐MG cells compared with that of HEB cells. Vertical error bars on data points represented the standard errors of mean obtained from replicates. qPCR, quantitative PCR

To identify core signal pathways in GBM cells, we analysed the global transcriptomes from U87‐MG and HEB cells through unsupervised hierarchal clustering analysis and principal component analysis. Under stringent criteria (probability > 0.8 and median fold change > 2),^16^ 5215 genes (27.8% of all genes analysed) were found to be differentially expressed between U87‐MG cells vs HEB cells with 2759 genes up‐regulated and 2456 genes down‐regulated (Figure 1A and Table S4). In clinical practice, the up‐regulated molecules are more suitable as diagnostic markers or therapeutic targets than the down‐regulated ones, and thus, we focused our analyses on the genes with higher expression levels in U87‐MG cells than in HEB cells. To determine the signal pathways correlated with the up‐regulated gene populations, we performed functional enrichment,^17^ and the results indicated that these genes were significantly classified into inflammatory response, cell migration, angiogenesis, cell adhesion and sulphur compound metabolism (Figure 1B and Table S5). Because of the critical functions of inflammatory response in GBM initiation and progression,^18^ we then investigated the major signal pathways related with inflammation, and we confirmed the involvement of the cytokine/cytokine receptor interaction and Toll‐like receptor 6 (TLR6) signal pathways (Figure 1C,D). Cytokines have been well documented to participate in GBM initiation and progression,^19^ but the reports on the TLR signal pathway are very limited and the functions of TLR in GBM remain unclear.^20^, ^21^ Further analysis on TLR6 signal pathway revealed a panel of up‐regulated genes, including CCL3, IL1B, IL6, LY96, PIK3CD, SPP1, TICAM1, TICAM2, TLR6 and TOLLIP, which were further verified by qPCR (Figure 1D and Figure S1). Interestingly, by examination of the CCLE database, we found that this set of involved genes was actually a representative signature for U87‐MG cells (Figure S2), which suggested that our analysis accurately reflected the genetic profile of the U87‐MG cells. Therefore, the analyses on the global transcriptomes provided several candidates for core signal pathways in GBM and had potential applications for mapping components of those signal pathways.

### 3.2 Signal pathway‐based categorization of highly up‐regulated genes in U87‐MG cells

**Figure 2:**
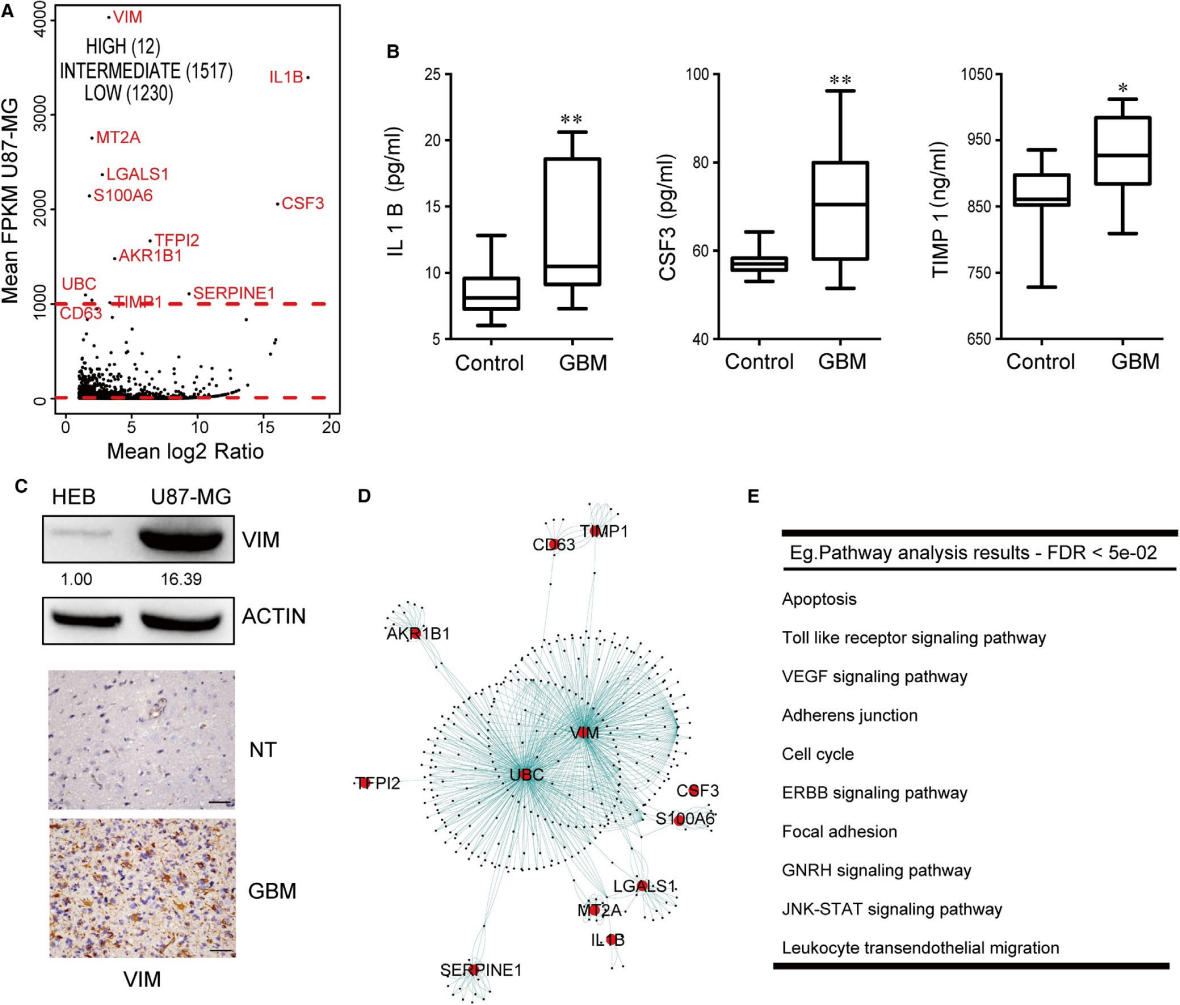
The analysis of highly up‐regulated genes in U87‐MG cells vs HEB cells. A, A scatter plot representation of RNA‐seq expression data. Every point represents a single gene plotted according to the mean log_2_ ratio between U87‐MG cells and HEB cells (x‐axis) and the mean U87‐MG expression (y‐axis). These genes were further sorted according to their expression in U87‐MG cells into high (≥1000), intermediate (<1000 and ≥10) and low (<10) expression categories. B, The levels of IL1B, CSF3 and TIMP1 in the serum of GBM patients were significantly higher than those of control patients. *P < 0.05, **P < 0.01. C, VIM protein expression level was higher in U87‐MG cells than in HEB cells and GBM tissues showed higher level of VIM than normal brain tissues. NT, normal tissue. Bars = 50 μm. D, Protein interaction of genes distributed into high category from protein interaction databases. E, Enriched signal pathway of GBM high expression genes and their interation genes

To further evaluate which signal pathways could be considered as core signal pathways in GBM cells, we classified all up‐regulated genes into three categories as previously described^22^: high (≥1000), intermediate (<1000 and ≥10) and low (<10) (Figure 2A). Twelve genes fell into the high expression category, which in this work were deemed representative genes and potential biomarkers for GBM cells. These included nine genes previously reported to be of significance in GBM, such as IL1B,^23^ SERPINE1,^24^ TFPI2,^25^ LGALS1,^26^ CD63,^27^ VIM,^28^ CSF3,^29^ TIMP1^30^ and S100A6,^31^ as well as three genes (AKR1B1, MT2A and UBC) not yet reported to have an effect in GBM cells. AKR1B1 promoted breast cancer progression by activation of EMT.^32^ MT2A played a tumour suppressor role through inhibiting NF‐κB signalling and was a prognostic biomarker for tumour patients.^33^ UBC was regarded as a promising therapeutic target for ovarian cancer patients with recurrent UBB silencing.^34^ Noticeably, both our data and the CCLE database showed the overexpression of AKR1B1, MT2A and UBC in GBM cells (Figure 2A and Figure S3), implying that these genes might play oncogenic roles in GBM cells. Moreover, CCLE data and expression analysis from TCGA data supported that all 12 genes could be a typical signature for U87‐MG cells among all glioma cells (Figures S3 and S4). However, the prognosis analysis from TCGA data indicated that only elevated expression of AKR1B1 had prognostic value for GBM patients (Figure S5). Next, we performed functional analysis of the 12 genes based on the protein interaction database, as previously described.^35^ The genes that interacted with these 12 genes were mainly enriched into several signal pathways (Figure 2D,E). Interestingly, some pathways overlapped with the biological processes identified above (Figure 1B). For example, TLR, JAK‐STAT and apoptosis were related to inflammation, VEGF related to angiogenesis and adhesion regulation related to migration. Therefore, inflammation‐, angiogenesis‐ and migration‐related signal pathways could be considered as core signal pathways in U87‐MG cells. Among the 12 genes, IL1B, CSF3 and TIMP1 were involved in inflammation response and their products were all secreted proteins. Therefore, whether these proteins could be regarded as plasma markers for GBM patients was determined. We measured their expressions with enzyme‐linked immunosorbent assay (ELISA) kits, and the results showed that their levels in GBM patients were up‐regulated (Figure 2B). Additionally, as an important marker for migration, high expression of VIM in GBM was also validated by Western blot and IHC in patient samples, and the result was consistent with our transcriptomic data (Figure 2C), suggesting that VIM could be used as a biopsy marker for GBM diagnosis. Taken together, through overlaying signal pathways from top‐ranked up‐regulated genes and globally up‐regulated genes, we confirmed three groups of core signal pathways in U87‐MG cells, as well as four candidate biomarkers for GBM.

### 3.3 Convergence of the core signal pathways on the hypoxia response in U87‐MG cells

**Figure 3:**
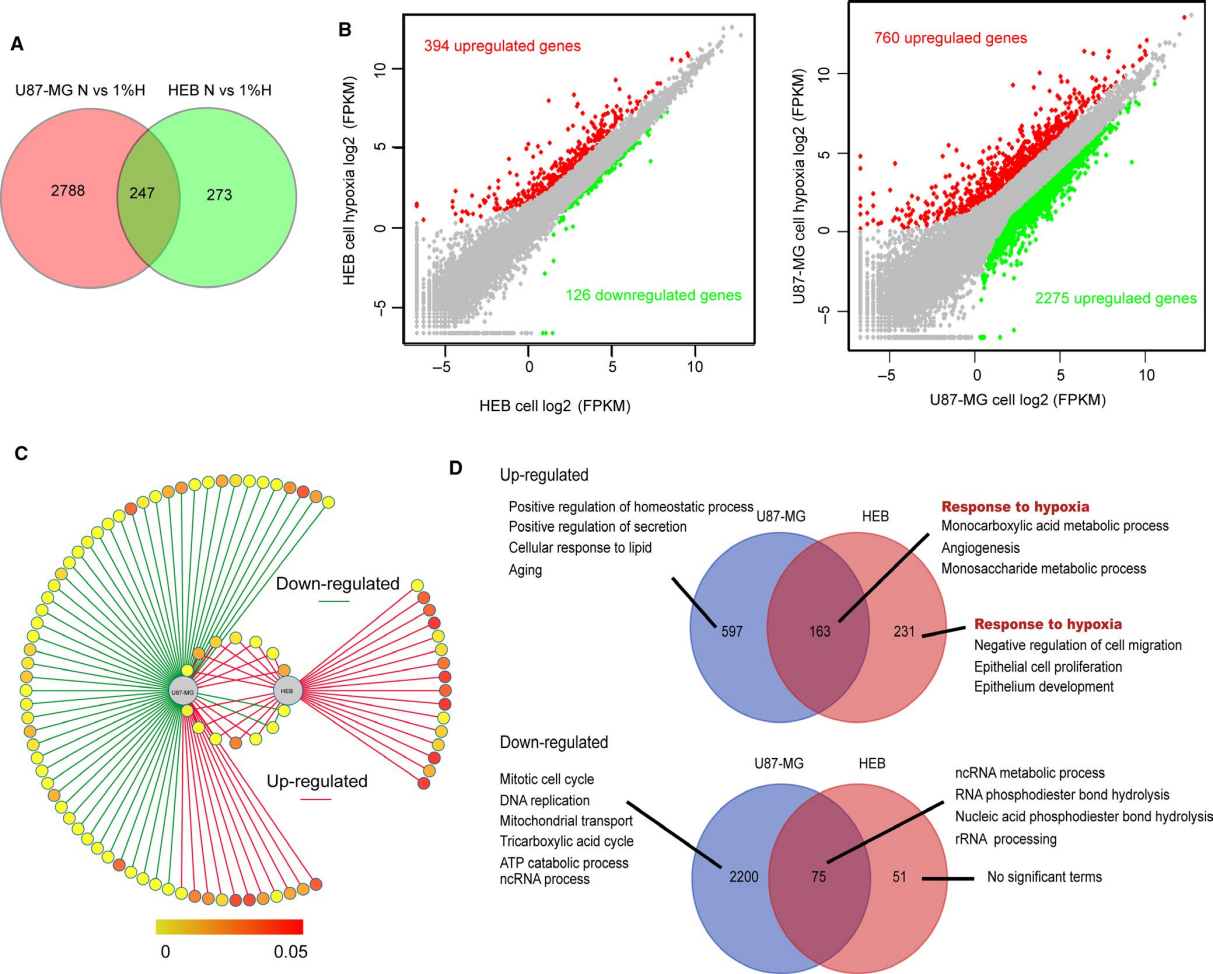
The landscape of transcriptomic alterations in U87‐MG cells vs HEB cells under hypoxic condition. A, Venn diagram of altered gene expression in response to hypoxia (H) vs normoxia (N) in U87‐MG cells and HEB cells respectively. B, Scatter plot of the comparison of log_2_ transformed gene expression levels and the differentially expressed gene distribution pattern of HEB and U87‐MG cells cultured in either normoxic or hypoxic conditions. Green and red points indicated down‐ and up‐regulation of gene expression, separately. C, Network summary of U87‐MG and HEB cells’ responses to hypoxia were organized based on up‐ and down‐regulated expression. Nodes represent biological processes and are coloured by adjusted *P* value. Red edges indicate up‐regulated. Green edges indicate down‐regulated. D, Biological process enrichment for U87‐MG and HEB cells in response to hypoxia. Venn diagrams summarized biological processes separated and overlapped for U87‐MG and HEB cells

Noticeably, the three groups of core signal pathways have been extensively reported to support the survival and invasion of cancer cells under hypoxic condition,^36^ therefore, their constitutive activation could aid GBM cells in adapting to hypoxia. To clarify the mechanism of GBM cell response to hypoxia, we investigated the landscape of hypoxia‐responded signal pathways via analysing the transcriptomes of U87‐MG and HEB cells under hypoxia compared to normoxia. As expected, transcriptome analyses showed dramatic differences in hypoxia‐induced response profiles (*R* = 0.03) (Figure 3A and Tables S6 and S7). Among the hypoxia‐response genes, 3035 genes and 520 genes were significantly changed in U87‐MG and HEB cells, respectively (Figure 3B), but only 238 genes were shared by the two groups (7.8% in U87‐MG group and 45.8% in HEB group) (Figure 3C). Interestingly, in U87‐MG cells, the majority of changed genes were down‐regulated (2275 down vs 760 up). Signal pathway enrichment revealed that the down‐regulated genes under hypoxic conditions were mainly distributed in the following categories: mitotic cell cycle, DNA replication, mitochondrial transport, tricarboxylic acid cycle (TCA) and ATP catabolism (Figure 3D and Table S8). Mitotic cell cycle and DNA replication are markers for cell proliferation.^37^ Mitochondrial transport of proteins or other biological materials is necessary to induce apoptosis.^38^ A decrease in TCA levels is a typical hallmark for the enhancement of the Warburg effects in cancer cells, and the Warburg effect has been shown to support the survival of cancer cells under hypoxic conditions by producing ATP with less oxygen consumption than TCA.^39^ In addition, ATP catabolism was down‐regulated, which potentially acted to maintain a reservoir of ATP and provided necessary energy for cancer cells under hypoxic conditions. Moreover, we also observed positive regulation of the homoeostatic process. Recent studies suggested that hypoxia‐inducible factor (HIF) proteins, which are induced by hypoxia, could promote cellular homoeostasis in glioma.^40^ All the above responses showed protective roles for survival, but these were not observed in HEB cells (Table S9). In conclusion, our data comprehensively mapped the response of GBM cells to hypoxia, which was dramatically distinct from normal cells.

### 3.4 Potential GBM molecular biomarkers identified from the hypoxia‐respondent transcriptome

**Figure 4:**
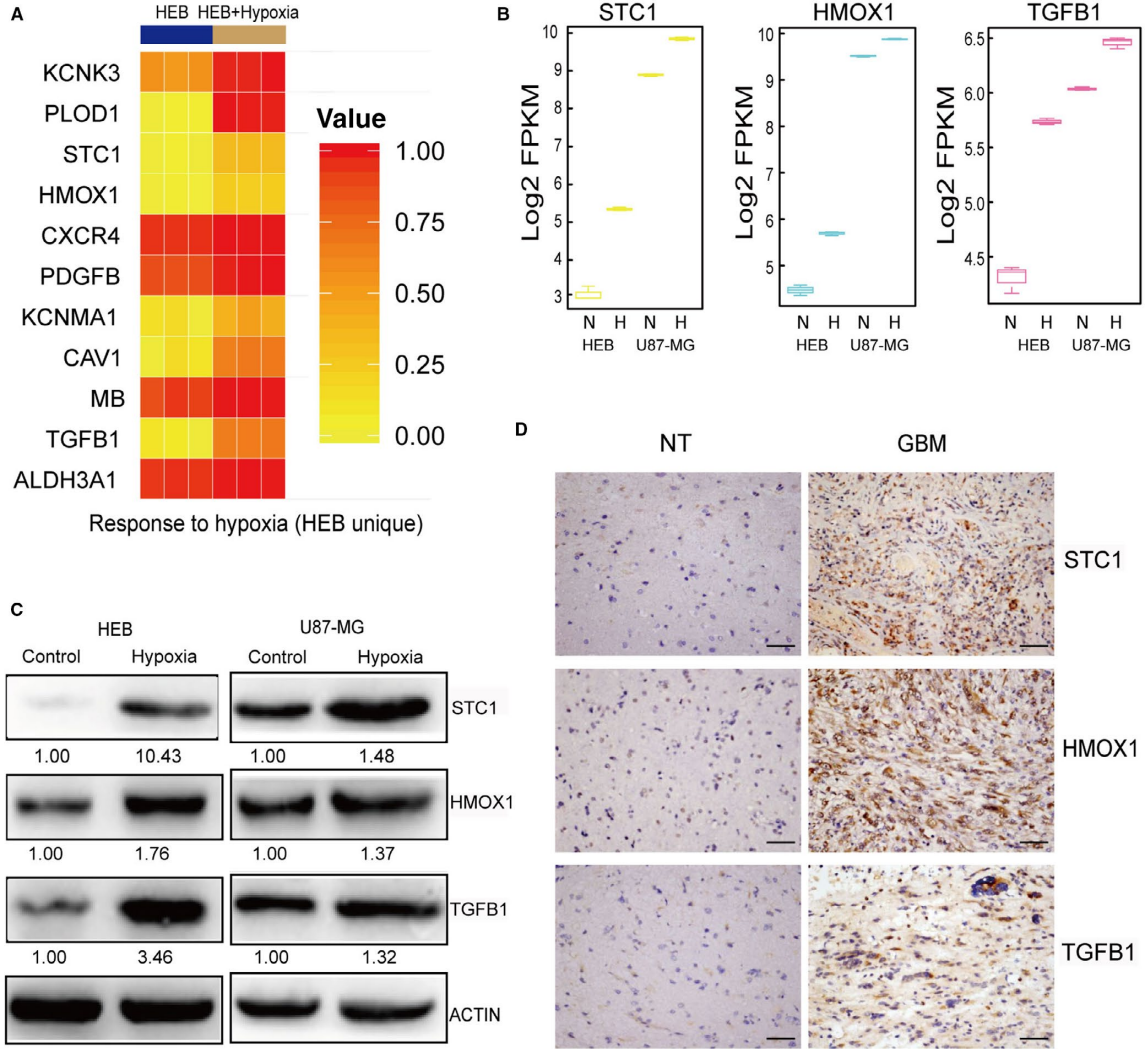
Potential diagnostic biomarkers for GBM identified from hypoxia‐related genes. A, Heat map of genes enriched in biological process of response to hypoxia in HEB cells alone. B, Box‐plots showing mRNA expressions of STC1, TGFB1 and HMOX1 in HEB and U87‐MG cells under normoxia (N) and hypoxia (H) conditions. C, Protein expression levels of STC1, TGFB1 and HMOX1 under normoxia and hypoxia conditions in HEB and U87 cells, respectively. D, GBM tissues harboured significantly higher levels of STC1, TGFB1 and HMOX1 than normal brain tissues. NT, normal tissue. Bars = 50 μm. GBM, glioblastoma

Next, we thoroughly analysed the response to hypoxia to illustrate the hypoxic characteristics of GBM. We suggested that hypoxia could lead to up‐regulation of some genes critical for cell survival and proliferation, and with these genes up‐regulated under normoxia, tumour cells could be better adapted to hypoxic conditions. To examine this hypothesis, we focused on the genes remarkably respondent to hypoxia only in HEB cells. Eleven eligible genes were identified, namely, ALDH3A1, TGFB1, MB, CAV1, KCNMA1, platelet‐derived growth factor B (PDGFB), CXCR4, HMOX1, STC1, PLOD1 and KCNK3 (Figure 4A). Most of them were involved in tumour development and progression. ALDH3A1 could mediate GBM resistance.^41^ As a downstream gene of HIF‐1, TGFB1 promoted the malignant phenotype of GBM by regulating proliferation and metastasis.^42^ Quann et al reported that CAV1 negatively regulated key cell growth and survival pathways and was an effective biomarker for predicting response to chemotherapy in GBM.^43^ KCNMA1 was involved the induction of paraptosis and was coupled with the mitochondrial respiratory chain in GBM.^44^ PDGFB was a HIF‐1α target and a potent angiogenic growth factor involved in GBM development and progression.^45^ The CXCL12/CXCR4 axis operated in GBM cells under hypoxia to promote survival and cell cycle progression.^46^ HMOX1 was correlated with stemness and promoted GBM metastasis.^47^ STC1 and PLOD1 were targets of HIF‐1 in tumour cells. A study by our group suggested that STC1 was a novel non‐canonical NOTCH ligand and acted as a crucial regulator of stemness in GBM.^48^, ^49^ KCNK3‐regulated apoptosis and proliferation in a subset of NSCLC.^50^ MB was found to be expressed in various tumours and could be associated with metastasis.^51^ Among these genes, TGFB1, PDGFB, CXCR4, HMOX1, STC1, PLOD1 and MB have been reported as downstream targets of HIF. Additionally, given that these genes were induced by hypoxia in normal cells, we suggested that some of these hypoxia‐inducible genes could be recognized as hallmarks in GBM cells. Furthermore, the TCGA and CCLE data showed that STC1, HMOX1 and TGFB1 were obviously up‐regulated in most glioma cells (Figures S4 and S6). The transcriptome data, qPCR and Western blot experiments also showed that glioma cells harboured much higher basal levels of the three genes than normal cells, and hypoxia presented less of an effect on these genes’ expression in U87‐MG cells than in HEB cells (Figure 4B,C and Figure S7). Moreover, the findings from patient samples validated that the protein levels of STC1, HMOX1 and TGFB1 were significantly higher in GBM tissues than in adjacent brain tissues (Figure 4D), implying that these genes could be potential biopsy biomarkers for GBM diagnosis. However, the prognosis analysis from TCGA data indicated that only STC1 served as an independent prognostic indicator for GBM patients. (Figure S5).

## 4 DISCUSSION

In this study, with analysis of global signal pathways and highly expressed gene‐related pathways, we identified three core signal pathways in GBM, namely, inflammation, angiogenesis and migration. Inflammation was ubiquitously observed in GBM and greatly supported GBM progression.^19^ Our data suggested that, among inflammation responses, cytokine/cytokine interaction was the core the signalling pathway in GBM cells. Furthermore, a set of genes involved in the TLR6 signal pathway were up‐regulated in GBM cells. Interestingly, CCLE data consistently indicated that the TLR6 gene signature in U87‐MG cells was actually common throughout all glioma cell lines. Importantly, based on our transcriptomic analyses, three inflammation‐related, highly expressed and secreted proteins, IL1B, CSF3 and TIMP1, were identified and could be used for GBM diagnosis in future. Moreover, robust angiogenesis was not only an important mechanism for tumour growth but also a typical response to inflammation in GBM. Increased migration ability was also a hallmark for GBM and was characterized by rapid infiltrative and diffuse growth into the surrounding brain tissue. Therefore, our transcriptomic‐based analysis was concise and reliable in term of identifying core signalling pathways in GBM cells.

With transcriptome‐based analyses, we further provided comprehensive insight into the hypoxic characteristics of GBM cells. Hypoxia was a typical microenvironment condition in GBM and many biological behaviors of GBM were induced by hypoxia.^37^ Distinct from normal cells, GBM cells were better suited for suivival in this condition, mainly through HIF‐induced signal pathways. This work, for the first time, clearly profiled the biological processes of response to hypoxia in GBM cells and revealed that GBM cells can protect themselves from hypoxia by enhancing homoeostasis, reducing proliferation and apoptosis, decreasing oxygen‐dependent ATP production and inhibiting ATP catabolism. However, all these protective pathways were not observed in normal glial cells. Surprisingly, three hypoxia‐response genes in normal cells, TGFB1, STC1 and HMOX1 showed distinctly higher expression in GBM cells than in normal cells, which suggested that they could be considered as biopsy markers for GBM diagnosis.

Integrated analysis with cellular sequencing and established gene ontology databases could provide accurate diagnosis or therapeutic information and direction for future research. To design personalized therapeutic strategies, patient‐specific targets should first be identified, which requires comparing the GBM samples and normal brain tissues from the same patient. However, the lack of tumour‐adjacent normal tissues is often a major problem. As a result of the aggressive and invasive growth of GBM cells, even the peri‐tumour areas often harbour molecular and metabolic changes that are very different from normal brain tissue and are similar to transformed cells. Alternatively, in GBM, unrelated normal brain tissues from people who have died of accidents or other non‐cancerous causes have been used as GBM tissue control. However, because of variations in gene backgrounds in normal individuals, it has been difficult to find real targets from patients using normal brain tissue from other donors as control. Therefore, the therapeutic targets and personalized treatments for GBM patients remain to be studied. In this study, we have analysed integrated transcriptome analysis of GBM and normal cells and TCGA and CCLE database to identify core signal pathways in GBM cells.

In conclusion, our analysis revealed GBM's core signal pathways, including inflammation, angiogenesis and migration and TLR6 pathway and hypoxic characteristics, involved in homoeostasis, proliferation and ATP metabolism. Moreover, three potential plasma markers, IL1B, CSF3 and TIMP1, as well as four biopsy markers, VIM, STC1, TGFB1 and HMOX1, were discovered for GBM diagnosis. These will provide new ideas for therapy and diagnosis for GBM patients.

## CONFLICT OF INTEREST

The authors confirm that there are no conflicts of interest.

## AUTHORS’ CONTRIBUTIONS

Y‐xG and EZ designed the study, performed experiments, interpreted the data and wrote the manuscript. BL and YC were involved in biological informatics analysis. KZ performed the IHC experiments. MC was involved in TCGA analysis. SH, LF and HW participated in cellular experiments. YW did the CCLE analysis. XZ and XL participated in manuscript writing. GW participated in the figure drawing. XB and Y‐qG conceived the study design, experiments plan and manuscript writing. All authors discussed the results and critically reviewed the manuscript.
