# Supplementary figures and images for: Integrated analysis identified core signal pathways and hypoxic characteristics of human glioblastoma
Source: J Cell Mol Med. 2019 Jul 7;23(9):6228–37. doi: 10.1111/jcmm.14507 (PMC6714287; doi:10.1111/jcmm.14507)

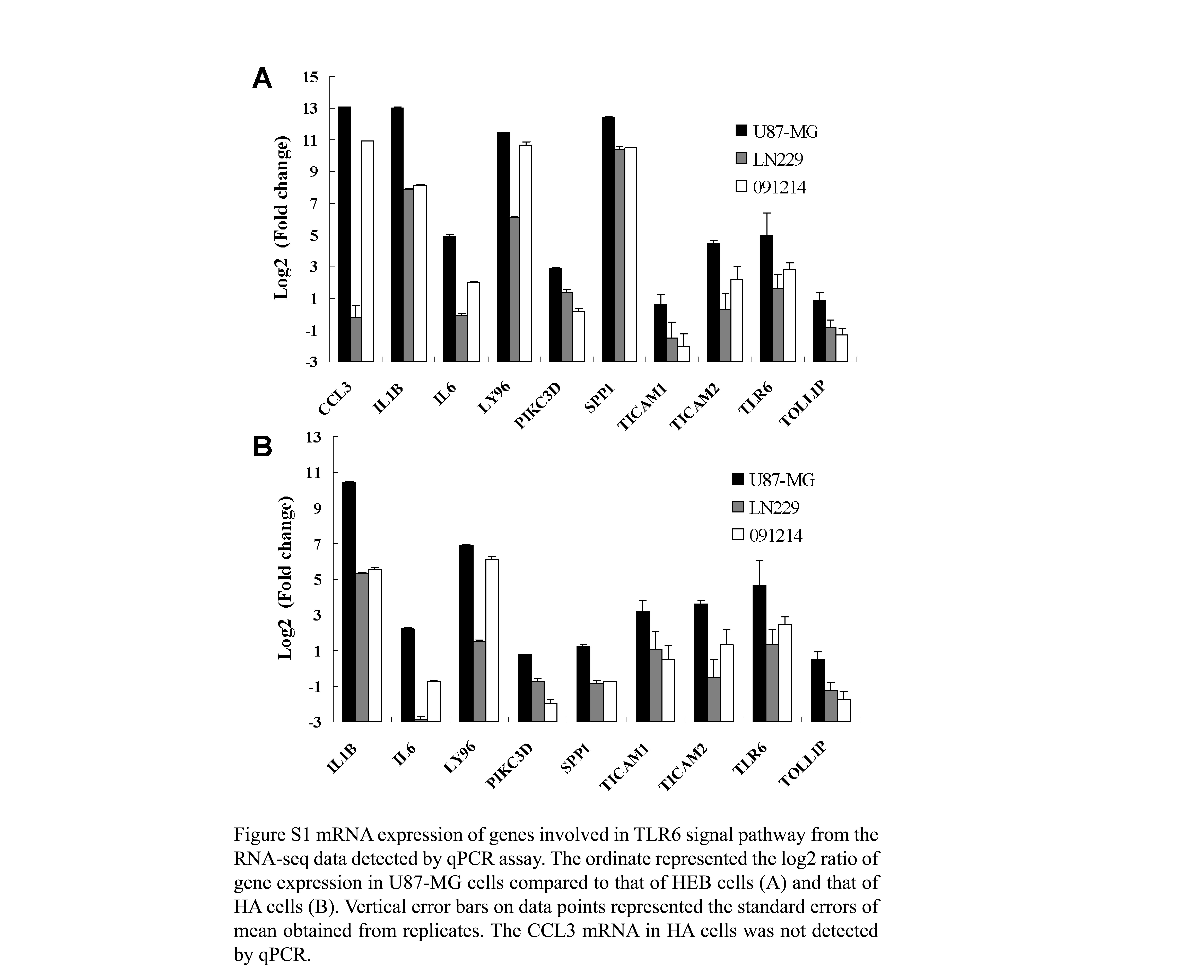

Supplement: Supplementary file 1 [file JCMM-23-6228-s001.jpg]

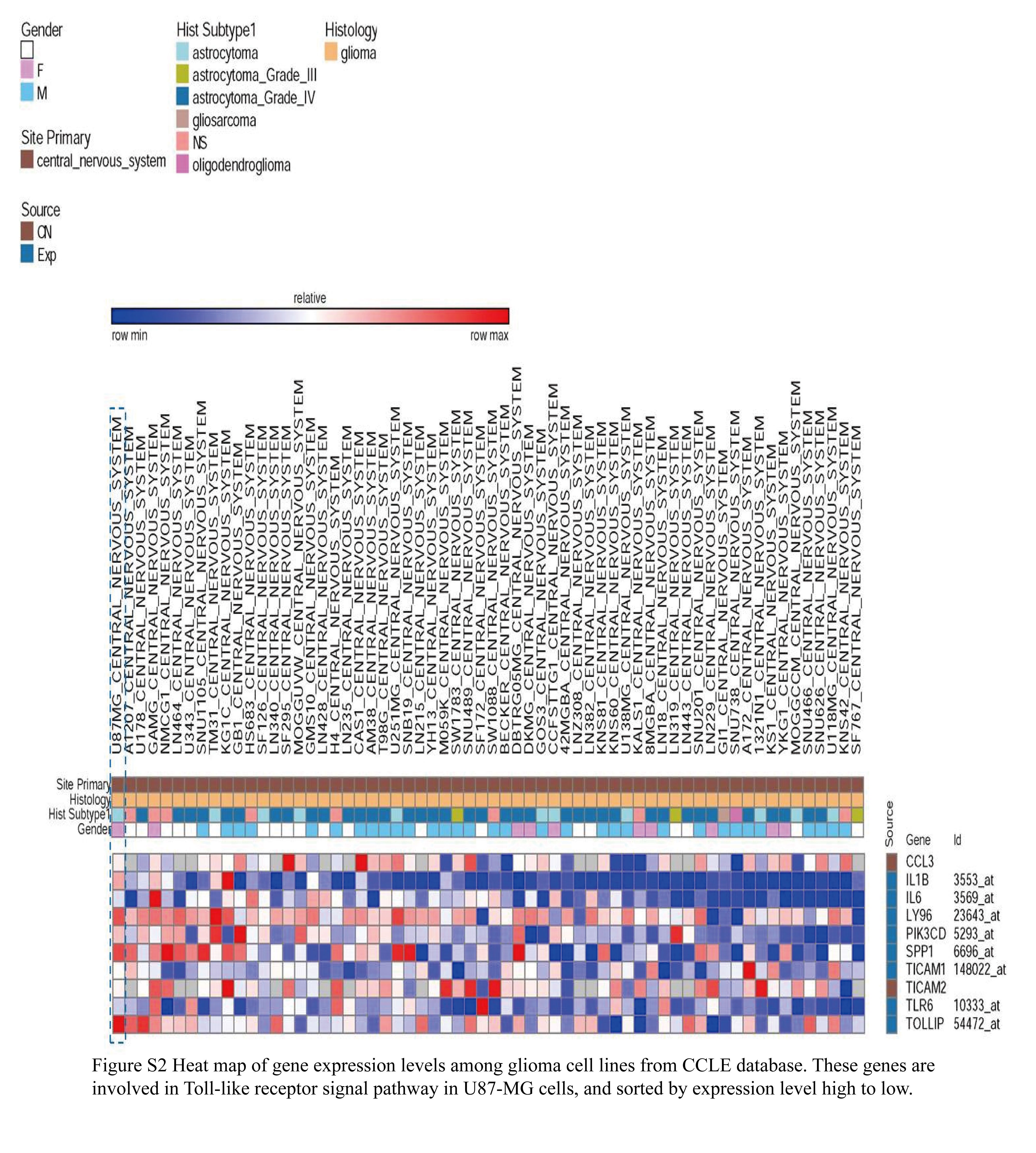

Supplement: Supplementary file 2 [file JCMM-23-6228-s007.jpg]

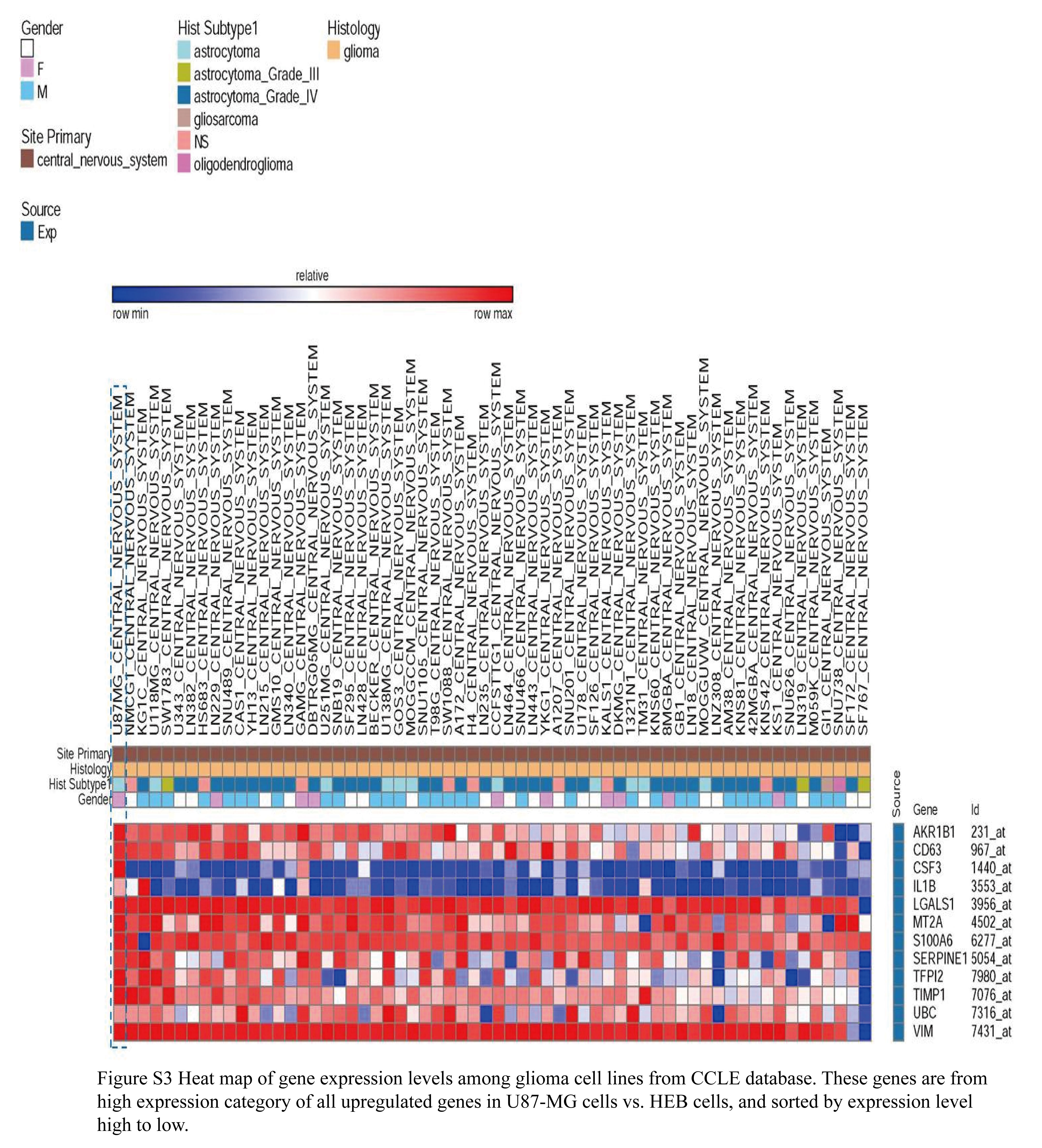

Supplement: Supplementary file 3 [file JCMM-23-6228-s002.jpg]

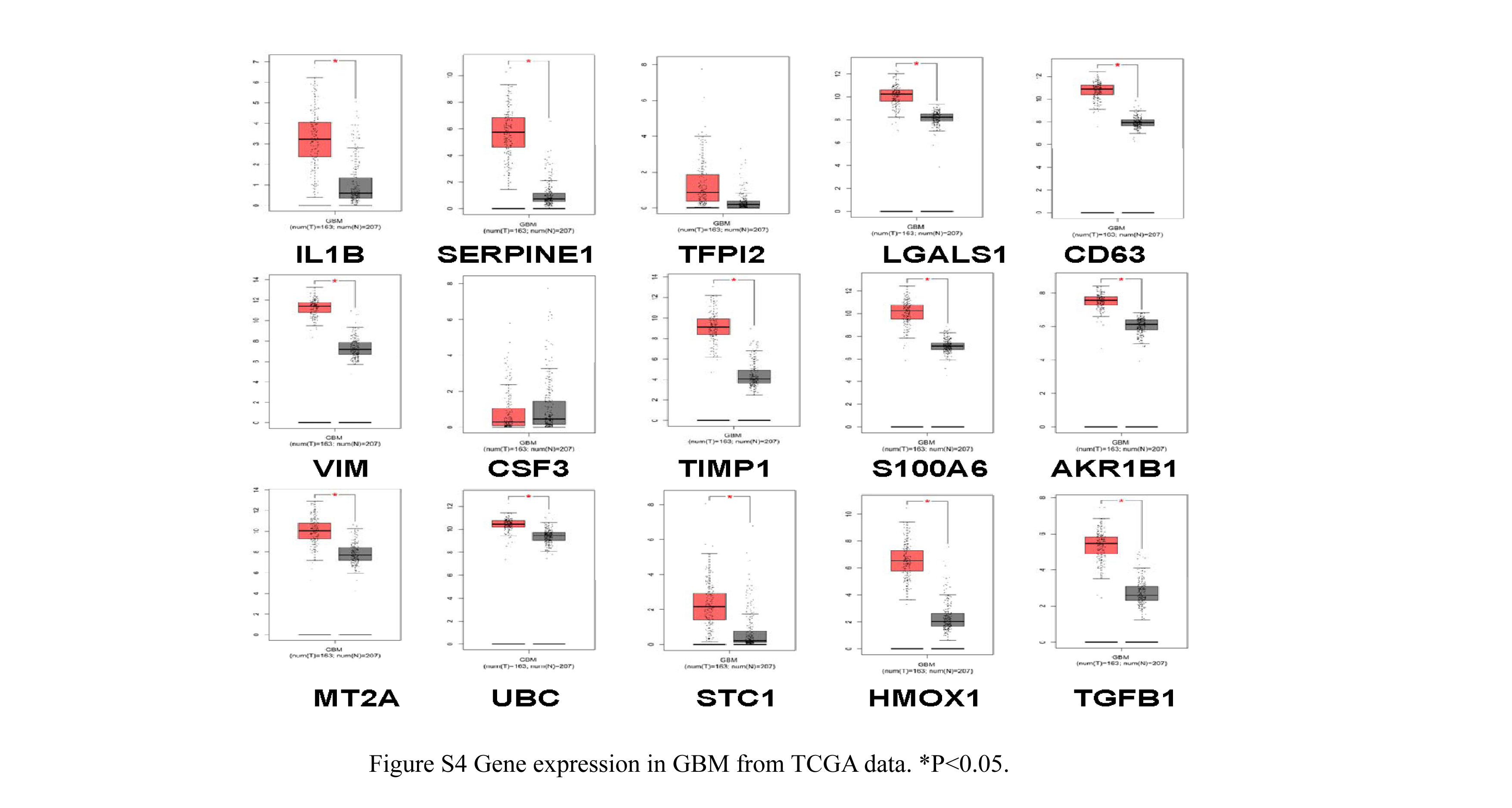

Supplement: Supplementary file 4 [file JCMM-23-6228-s003.jpg]

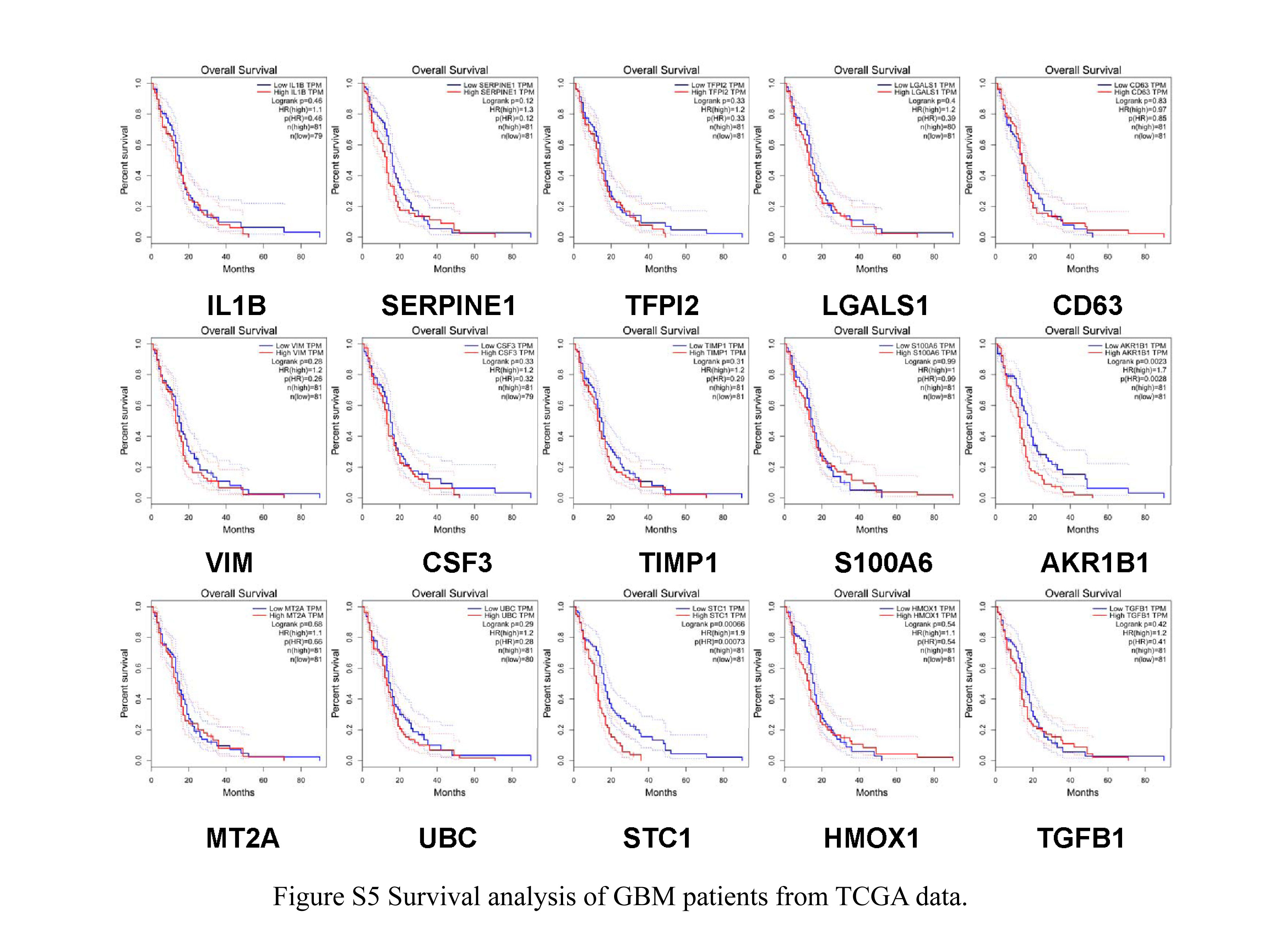

Supplement: Supplementary file 5 [file JCMM-23-6228-s004.jpg]

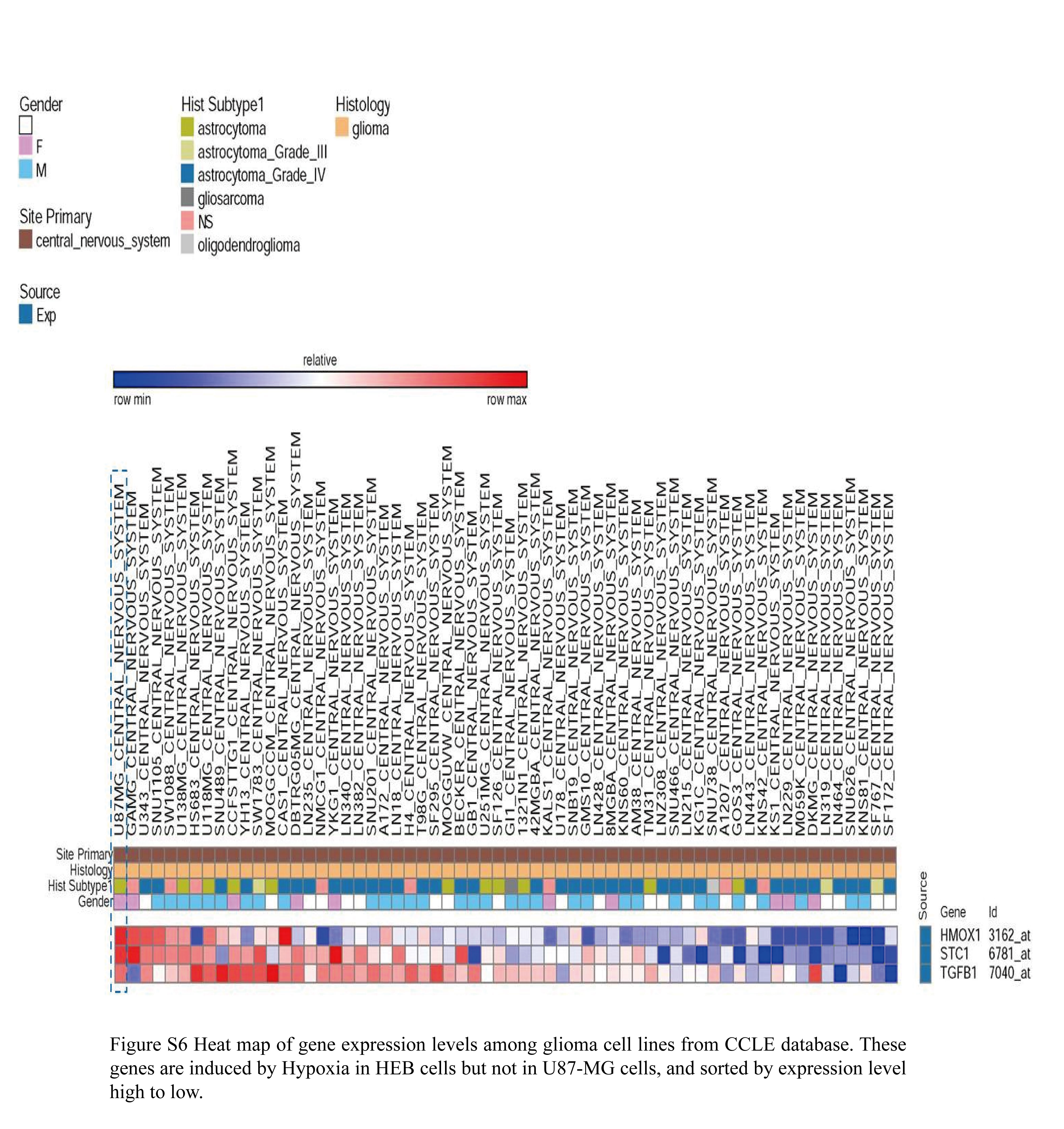

Supplement: Supplementary file 6 [file JCMM-23-6228-s005.jpg]

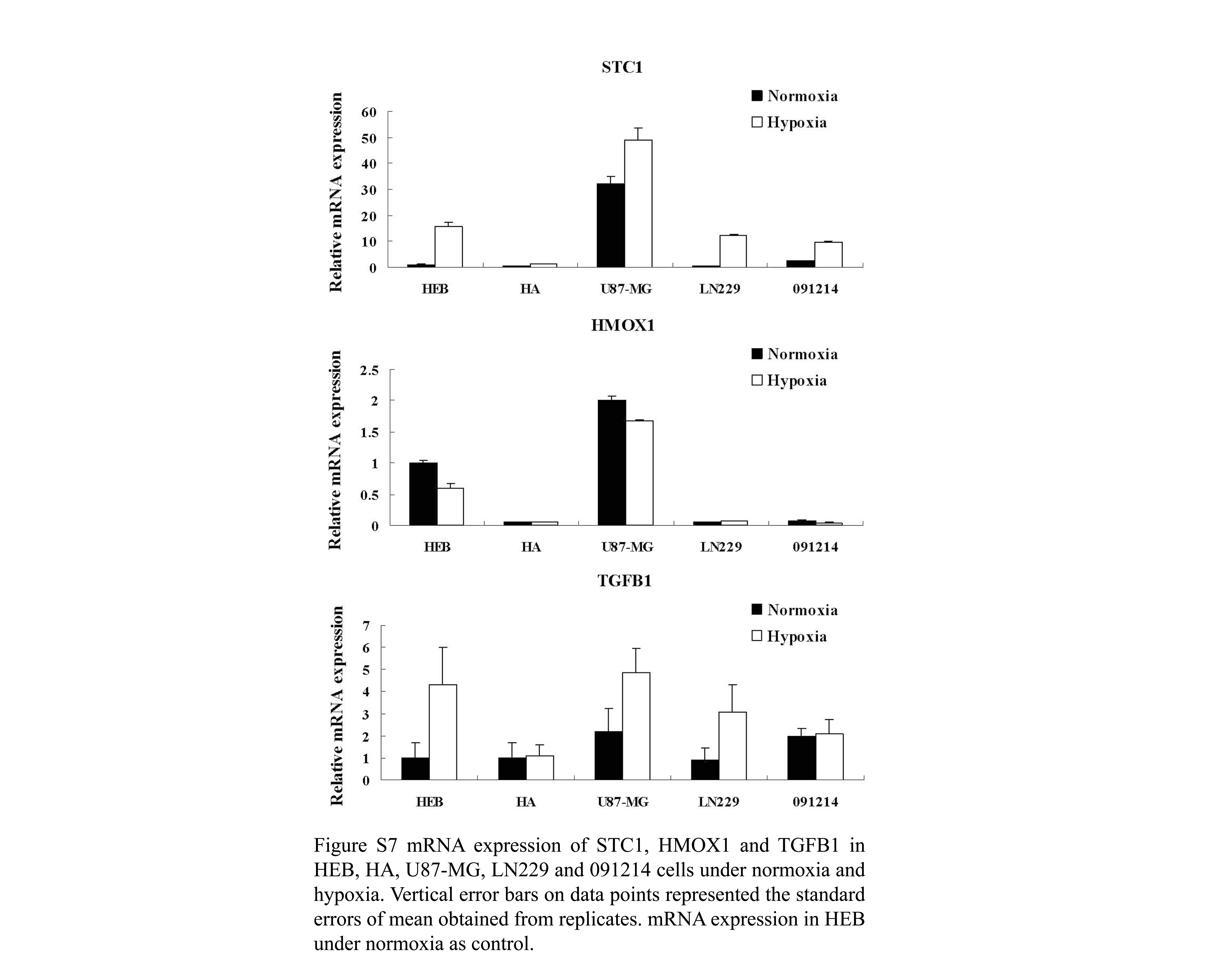

Supplement: Supplementary file 7 [file JCMM-23-6228-s006.jpg]
