# Supplementary material for: Integrated analysis identified core signal pathways and hypoxic characteristics of human glioblastoma
Source: J Cell Mol Med. 2019 Jul 7;23(9):6228–37. doi: 10.1111/jcmm.14507 (PMC6714287; doi:10.1111/jcmm.14507)
Supplement: Supplementary file 11 [file JCMM-23-6228-s012.pdf]

Table S4 Differential gene expression analysis by Noiseq for pairwise comparison of U87-MG versus HEB, based on RNA-seq data.

	Gene	Symbol	log_2Ratio	Probability	Ratio	Description
UP in HEB	**1387**	CREBBP	-2.053069	0.9322808	0.240971	CREB binding protein
UP in HEB	**51599**	LSR	-3.50041	0.9683005	0.088363	lipolysis stimulated lipoprotein receptor
UP in HEB	**7022**	TFAP2C	-2.28423	0.9170622	0.205295	transcription factor AP-2 gamma (activating enhancer binding protein 2 gamma)
UP in HEB	**1244**	ABCC2	-6.431567	0.9968354	0.011585	ATP-binding cassette, sub-family C (CFTR/MRP), member 2
UP in HEB	**1969**	EPHA2	-1.165778	0.8882518	0.445724	EPH receptor A2
UP in HEB	**79805**	VASH2	-6.577429	0.8173058	0.010471	vasohibin 2
UP in HEB	**84275**	SLC25A33	-1.84193	0.9192846	0.278948	solute carrier family 25 (pyrimidine nucleotide carrier), member 33
UP in HEB	**56666**	PANX2	-3.825604	0.9464683	0.070531	pannexin 2
UP in HEB	**5464**	PPA1	-2.2433	0.942957	0.211203	pyrophosphatase (inorganic) 1
UP in HEB	**91664**	ZNF845	-7.146357	0.8375469	0.007059	zinc finger protein 845
UP in HEB	**10919**	EHMT2	-1.96167	0.9272406	0.256731	euchromatic histone-lysine N-
UP in HEB	**646960**	PRSS56	-7.354617	0.8615037	0.00611	protease, serine, 56
UP in HEB	**6631**	SNRPC	-1.207881	0.8965278	0.432904	small nuclear ribonucleoprotein polypeptide
UP in HEB	**79924**	ADM2	-4.201634	0.8607659	0.054348	adrenomedullin 2
UP in HEB	**3419**	IDH3A	-1.088584	0.8892385	0.470223	isocitrate dehydrogenase 3 (NAD+) alpha
UP in HEB	**39**	ACAT2	-1.270628	0.8963678	0.414479	acetyl-CoA acetyltransferase 2
UP in HEB	**51025**	PAM16	-1.166086	0.8914342	0.445629	presequence translocase-associated motor 16 homolog (S. cerevisiae)
UP in HEB	**79823**	CAMKMT	-1.140586	0.8409693	0.453575	calmodulin-lysine N-methyltransferase
UP in HEB	**60436**	TGIF2	-7.500643	0.9900528	0.005522	TGFB-induced factor homeobox 2
UP in HEB	**23318**	ZCCHC11	-1.099706	0.8779935	0.466612	zinc finger, CCHC domain containing 11
UP in HEB	**64777**	RMND5B	-1.760366	0.9232137	0.295173	required for meiotic nuclear division 5 homolog B (S. cerevisiae)
UP in HEB	**23379**	KIAA0947	-1.177068	0.891052	0.442249	KIAA0947
UP in HEB	**113802**	HENMT1	-11.09188	0.9955198	0.000458	HEN1 methyltransferase homolog 1 (Arabidopsis)
UP in HEB	**57380**	MRS2	-2.028472	0.9351521	0.245114	MRS2 magnesium homeostasis factor homolog (S. cerevisiae)
UP in HEB	**8776**	MTMR1	-1.965408	0.9175689	0.256067	myotubularin related protein 1
UP in HEB	**8711**	TNK1	-4.694058	0.9618469	0.038632	tyrosine kinase, non-receptor, 1
UP in HEB	**57447**	NDRG2	-4.655103	0.9449571	0.039689	NDRG family member 2
UP in HEB	**23225**	NUP210	-13.77376	0.9998044	7.14E-05	nucleoporin 210kDa
UP in HEB	**20**	ABCA2	-1.960897	0.9252227	0.256869	ATP-binding cassette, sub-family A (ABC1), member 2
UP in HEB	**23295**	MGRN1	-1.119735	0.8823848	0.460178	mahogunin ring finger 1, E3 ubiquitin protein ligase
UP in HEB	**79085**	SLC25A23	-6.196618	0.9946486	0.013634	solute carrier family 25 (mitochondrial carrier; phosphate carrier), member 23
UP in HEB	**9401**	RECQL4	-1.075093	0.8835493	0.47464	RecQ protein-like 4
UP in HEB	**57332**	CBX8	-1.933167	0.9133287	0.261854	chromobox homolog 8
UP in HEB	**23277**	CLUH	-1.088711	0.8893363	0.470181	clustered mitochondria (cluA/CLU1)
UP in HEB	**7087**	ICAM5	-2.042483	0.9041282	0.242746	intercellular adhesion molecule 5,
UP in HEB	**492307**	C8orf22	-11.8243	0.9975999	0.000276	chromosome 8 open reading frame 22
UP in HEB	**285696**	LOC28569	-3.31797	0.8788469	0.100275	uncharacterized LOC285696
UP in HEB	**9133**	CCNB2	-1.561618	0.9091774	0.338771	cyclin B2
UP in HEB	**6513**	SLC2A1	-1.19293	0.891932	0.437414	solute carrier family 2 (facilitated glucose transporter), member 1
UP in HEB	**148534**	TMEM56	-3.214225	0.9583356	0.107751	transmembrane protein 56
UP in HEB	**55959**	SULF2	-7.659104	0.9707362	0.004947	sulfatase 2
UP in HEB	**6744**	SSFA2	-1.033896	0.8857539	0.48839	sperm specific antigen 2

UP in HEB	**6502**	SKP2	-2.38707	0.9505218	0.19117	S-phase kinase-associated protein 2, E3 ubiquitin protein ligase
UP in HEB	**222161**	DKFZP586 I1420	-1.928066	0.8437961	0.262781	uncharacterized protein DKFZp586I1420
UP in HEB	**7111**	TMOD1	-1.0872	0.858988	0.470674	tropomodulin 1
UP in HEB	**146909**	KIF18B	-1.62118	0.9185557	0.32507	kinesin family member 18B
UP in HEB	**1675**	CFD	-7.195987	0.9924618	0.00682	complement factor D (adipsin)
UP in HEB	**649**	BMP1	-1.574885	0.9038971	0.33567	bone morphogenetic protein 1
UP in HEB	**64216**	TFB2M	-1.122494	0.8871229	0.459299	transcription factor B2, mitochondrial
UP in HEB	**412**	STS	-1.67758	0.8508365	0.312606	steroid sulfatase (microsomal), isozyme S
UP in HEB	**6239**	RREB1	-1.483816	0.8676907	0.357542	ras responsive element binding protein 1
UP in HEB	**2886**	GRB7	-4.304205	0.9607979	0.050618	growth factor receptor-bound protein 7
UP in HEB	**29984**	RHOD	-9.774238	0.9856792	0.001142	ras homolog family member D
UP in HEB	**51293**	CD320	-1.681936	0.9237382	0.311664	CD320 molecule
UP in HEB	**1289**	COL5A1	-2.309865	0.9420414	0.201679	collagen, type V, alpha 1
UP in HEB	**53354**	PANK1	-1.921301	0.8441428	0.264016	pantothenate kinase 1
UP in HEB	**27115**	PDE7B	-6.189825	0.9685228	0.013699	phosphodiesterase 7B
UP in HEB	**2953**	GSTT2	-3.088845	0.9635358	0.117534	glutathione S-transferase theta 2
UP in HEB	**256691**	MAMDC2	-4.330981	0.9876527	0.049687	MAM domain containing 2
UP in HEB	**8899**	PRPF4B	-1.742292	0.9207069	0.298894	PRP4 pre-mRNA processing factor 4 homolog B (yeast)
UP in HEB	**79716**	NPEPL1	-1.355103	0.8903319	0.390907	aminopeptidase-like 1
UP in HEB	**23151**	GRAMD4	-1.333119	0.8715043	0.396909	GRAM domain containing 4
UP in HEB	**7262**	PHLDA2	-1.508304	0.9056483	0.351524	pleckstrin homology-like domain, family A, member 2
UP in HEB	**10451**	VAV3	-1.911394	0.9218536	0.265836	vav 3 guanine nucleotide exchange factor
UP in HEB	**3832**	KIF11	-1.486914	0.9049816	0.356775	kinesin family member 11
UP in HEB	**100527964**	LOC10052 7964	-2.092018	0.8354401	0.234552	uncharacterized LOC100527964
UP in HEB	**11269**	DDX19B	-2.015091	0.9311341	0.247399	DEAD (Asp-Glu-Ala-Asp) box polypeptide
UP in HEB	**55299**	BRIX1	-1.149859	0.893781	0.450669	BRX1, biogenesis of ribosomes, homolog (S. cerevisiae)
UP in HEB	**2806**	GOT2	-1.049092	0.8887141	0.483272	glutamic-oxaloacetic transaminase 2, mitochondrial (aspartate aminotransferase 2)
UP in HEB	**9241**	NOG	-6.981092	0.8165324	0.007916	noggin
UP in HEB	**23240**	KIAA0922	-2.927149	0.9511441	0.131474	KIAA0922
UP in HEB	**1058**	CENPA	-1.150625	0.8865717	0.45043	centromere protein A
UP in HEB	**84667**	HES7	-4.153703	0.9821146	0.056184	hairy and enhancer of split 7 (Drosophila)
UP in HEB	**3775**	KCNK1	-10.80655	0.9944086	0.000558	potassium channel, subfamily K, member 1
UP in HEB	**10891**	PPARGC1 A	-3.646046	0.9674116	0.079879	peroxisome proliferator-activated receptor gamma, coactivator 1 alpha
UP in HEB	**80742**	PRR3	-2.531753	0.9423436	0.172928	proline rich 3
UP in HEB	**158257**	MIRLET7 DHG	-2.0434	0.8919231	0.242591	MIRLET7D host gene (non-protein coding)
UP in HEB	**5152**	PDE9A	-7.815917	0.9055061	0.004438	phosphodiesterase 9A
UP in HEB	**63915**	BLOC1S5	-2.245052	0.9076751	0.210946	biogenesis of lysosomal organelles complex-1, subunit 5, muted
UP in HEB	**10735**	STAG2	-1.437288	0.9038971	0.369261	stromal antigen 2
UP in HEB	**1030**	CDKN2B	-7.944468	0.9158888	0.00406	cyclin-dependent kinase inhibitor 2B (p15, inhibits CDK4)
UP in HEB	**342897**	NCCRP1	-5.996923	0.9841769	0.015658	non-specific cytotoxic cell receptor protein 1 homolog (zebrafish)
UP in HEB	**28984**	RGCC	-5.681824	0.9909595	0.019481	regulator of cell cycle
UP in HEB	**8330**	HIST1H2A K	-3.126532	0.8798514	0.114504	histone cluster 1, H2ak
UP in HEB	**23220**	DTX4	-6.509115	0.9794656	0.010979	deltex homolog 4 (Drosophila)
UP in HEB	**134353**	LSM11	-1.209887	0.8257329	0.432302	LSM11, U7 small nuclear RNA associated

UP in HEB	**23604**	DAPK2	-5.938599	0.808852	0.016304	death-associated protein kinase 2
UP in HEB	**730092**	RRN3P1	-6.969626	0.8151013	0.007979	RNA polymerase I transcription factor homolog (S. cerevisiae) pseudogene 1
UP in HEB	**7049**	TGFBR3	-2.482221	0.9370366	0.178969	transforming growth factor, beta receptor III
UP in HEB	**23047**	PDS5B	-1.251169	0.860997	0.420108	PDS5, regulator of cohesion maintenance, homolog B (S. cerevisiae)
UP in HEB	**8312**	AXIN1	-1.530698	0.8995058	0.34611	axin 1
UP in HEB	**3487**	IGFBP4	-2.081999	0.9407791	0.236187	insulin-like growth factor binding protein 4
UP in HEB	**152002**	XXYLT1	-2.691833	0.9523975	0.154767	xyloside xylosyltransferase 1
UP in HEB	**85379**	KIAA1671	-1.135383	0.8544456	0.455214	KIAA1671
UP in HEB	**23363**	OBSL1	-2.237499	0.9373744	0.212054	obscurin-like 1
UP in HEB	**57167**	SALL4	-6.448171	0.9875993	0.011453	sal-like 4 (Drosophila)
UP in HEB	**3823**	KLRC3	-7.478433	0.8740822	0.005607	killer cell lectin-like receptor subfamily C, member 3
UP in HEB	**58489**	ABHD17C	-1.215759	0.8743133	0.430547	abhydrolase domain containing 17C
UP in HEB	**27346**	TMEM97	-1.467612	0.9034793	0.36158	transmembrane protein 97
UP in HEB	**6337**	SCNN1A	-12.40443	0.9987555	0.000184	sodium channel, non-voltage-gated 1 alpha subunit
UP in HEB	**10921**	RNPS1	-1.480175	0.9069639	0.358445	RNA binding protein S1, serine-rich domain
UP in HEB	**79041**	TMEM38	-1.477748	0.8592814	0.359049	transmembrane protein 38A
UP in HEB	**10636**	RGS14	-4.011713	0.8968567	0.061995	regulator of G-protein signaling 14
UP in HEB	**1734**	DIO2	-6.578069	0.9929596	0.010467	deiodinase, iodothyronine, type II
UP in HEB	**114971**	PTPMT1	-1.190959	0.8927409	0.438012	protein tyrosine phosphatase, mitochondrial 1
UP in HEB	**1802**	DPH2	-1.288819	0.8963767	0.409286	DPH2 homolog (S. cerevisiae)
UP in HEB	**2775**	GNAO1	-6.178715	0.9732163	0.013805	guanine nucleotide binding protein (G protein), alpha activating activity polypeptide O
UP in HEB	**2273**	FHL1	-2.280415	0.9423525	0.205838	four and a half LIM domains 1
UP in HEB	**64718**	UNKL	-1.784394	0.9180312	0.290298	unkempt homolog (Drosophila)-like
UP in HEB	**84919**	PPP1R15B	-1.275096	0.8950166	0.413198	protein phosphatase 1, regulatory subunit
UP in HEB	**170689**	ADAMTS 15	-6.546894	0.8130745	0.010695	ADAM metallopeptidase with thrombospondin type 1 motif, 15
UP in HEB	**9231**	DLG5	-2.367373	0.9516241	0.193798	discs, large homolog 5 (Drosophila)
UP in HEB	**2331**	FMOD	-7.494522	0.8758689	0.005545	fibromodulin
UP in HEB	**4357**	MPST	-1.470963	0.9033993	0.360741	mercaptopyruvate sulfurtransferase
UP in HEB	**26517**	TIMM13	-1.777682	0.9254005	0.291652	translocase of inner mitochondrial membrane 13 homolog (yeast)
UP in HEB	**100008588**	RNA18S5	-1.150404	0.8949633	0.450499	RNA, 18S ribosomal 5
UP in HEB	**100652772**	LOC10065 2772	-2.74967	0.9310897	0.148685	uncharacterized LOC100652772
UP in HEB	**4299**	AFF1	-1.104374	0.8723665	0.465104	AF4/FMR2 family, member 1
UP in HEB	**11001**	SLC27A2	-10.41891	0.9919373	0.00073	solute carrier family 27 (fatty acid transporter), member 2
UP in HEB	**51082**	POLR1D	-1.295294	0.8992924	0.407453	polymerase (RNA) I polypeptide D, 16kDa
UP in HEB	**4145**	MATK	-5.698705	0.9861415	0.019254	megakaryocyte-associated tyrosine kinase
UP in HEB	**79646**	PANK3	-3.001548	0.9682828	0.124866	pantothenate kinase 3
UP in HEB	**9521**	EEF1E1	-1.106094	0.8882785	0.46455	eukaryotic translation elongation factor 1 epsilon 1
UP in HEB	**27333**	GOLIM4	-1.066224	0.8869095	0.477567	golgi integral membrane protein 4
UP in HEB	**5373**	PMM2	-1.585375	0.9191512	0.333238	phosphomannomutase 2
UP in HEB	**100462977**	MTRNR2L	-2.518799	0.9532153	0.174488	MT-RNR2-like 1
UP in HEB	**54438**	GFOD1	-2.003475	0.8796736	0.249399	glucose-fructose oxidoreductase domain containing 1
UP in HEB	**10457**	GPNMB	-2.740314	0.95878	0.149652	glycoprotein (transmembrane) nmb
UP in HEB	**6189**	RPS3A	-2.647049	0.9599001	0.159646	ribosomal protein S3A
UP in HEB	**2517**	FUCA1	-1.344525	0.8882163	0.393784	fucosidase, alpha-L- 1, tissue
UP in HEB	**9882**	TBC1D4	-1.581774	0.9036838	0.334071	TBC1 domain family, member 4

UP in HEB	**317749**	DHRS4L2	-1.76127	0.9130976	0.294988	dehydrogenase/reductase (SDR family) member 4 like 2
UP in HEB	**7145**	TNS1	-1.396184	0.8732732	0.379933	tensin 1
UP in HEB	**84301**	DDI2	-1.660978	0.9115864	0.316225	DNA-damage inducible 1 homolog 2 (S. cerevisiae)
UP in HEB	**23596**	OPN3	-2.451122	0.9474639	0.182868	opsin 3
UP in HEB	**2262**	GPC5	-1.827207	0.8483208	0.28181	glypican 5
UP in HEB	**29128**	UHRF1	-1.045248	0.8848739	0.484562	ubiquitin-like with PHD and ring finger
UP in HEB	**1788**	DNMT3A	-1.429341	0.8181947	0.3713	DNA (cytosine-5-)-methyltransferase 3 alpha
UP in HEB	**64789**	EXO5	-5.828991	0.9122175	0.017591	exonuclease 5
UP in HEB	**6650**	SOLH	-1.445637	0.8986168	0.36713	small optic lobes homolog (Drosophila)
UP in HEB	**157285**	SGK223	-1.89377	0.8909986	0.269103	homolog of rat pragma of Rnd2
UP in HEB	**10573**	MRPL28	-1.232856	0.8976657	0.425474	mitochondrial ribosomal protein L28
UP in HEB	**51765**	MST4	-1.248982	0.8914787	0.420745	serine/threonine protein kinase MST4
UP in HEB	**41**	ASIC1	-1.532014	0.8573791	0.345794	acid-sensing (proton-gated) ion channel 1
UP in HEB	**23195**	MDN1	-1.676969	0.9178623	0.312739	MDN1, midasin homolog (yeast)
UP in HEB	**56652**	C10orf2	-1.875163	0.9247871	0.272596	chromosome 10 open reading frame 2
UP in HEB	**6470**	SHMT1	-1.137633	0.8883496	0.454505	serine hydroxymethyltransferase 1 (soluble)
UP in HEB	**1672**	DEFB1	-7.420662	0.8678418	0.005837	defensin, beta 1
UP in HEB	**51537**	MTFP1	-1.786946	0.9218003	0.289785	mitochondrial fission process 1
UP in HEB	**54680**	ZNHIT6	-1.620053	0.8910697	0.325323	zinc finger, HIT-type containing 6
UP in HEB	**81539**	SLC38A1	-1.637714	0.9233559	0.321365	solute carrier family 38, member 1
UP in HEB	**6659**	SOX4	-1.057203	0.8732288	0.480563	SRY (sex determining region Y)-box 4
UP in HEB	**163154**	PRR22	-4.772022	0.9654026	0.0366	proline rich 22
UP in HEB	**352954**	GATS	-3.041693	0.9569488	0.121439	GATS, stromal antigen 3 opposite strand
UP in HEB	**643988**	C1orf233	-9.770389	0.9856348	0.001145	chromosome 1 open reading frame 233
UP in HEB	**6323**	SCN1A	-10.42836	0.9945153	0.000726	sodium channel, voltage-gated, type I, alpha subunit
UP in HEB	**6080**	SNORA73	-2.121548	0.9169378	0.2298	small nucleolar RNA, H/ACA box 73A
UP in HEB	**10319**	LAMC3	-10.60363	0.9963376	0.000643	laminin, gamma 3
UP in HEB	**7699**	ZNF140	-3.9667	0.9229114	0.063959	zinc finger protein 140
UP in HEB	**9022**	CLIC3	-5.53472	0.8961367	0.021572	chloride intracellular channel 3
UP in HEB	**5141**	PDE4A	-5.239346	0.981039	0.026473	phosphodiesterase 4A, cAMP-specific
UP in HEB	**80864**	EGFL8	-2.769994	0.8413249	0.146605	EGF-like-domain, multiple 8
UP in HEB	**7701**	ZNF142	-1.022933	0.8697353	0.492115	zinc finger protein 142
UP in HEB	**7163**	TPD52	-2.940519	0.964647	0.130261	tumor protein D52
UP in HEB	**54537**	FAM35A	-1.123985	0.8878607	0.458825	family with sequence similarity 35, member
UP in HEB	**2894**	GRID1	-6.981092	0.8165324	0.007916	glutamate receptor, ionotropic, delta 1
UP in HEB	**55796**	MBNL3	-3.35828	0.938699	0.097512	muscleblind-like splicing regulator 3
UP in HEB	**8644**	AKR1C3	-1.374326	0.8916387	0.385733	aldo-keto reductase family 1, member C3
UP in HEB	**143098**	MPP7	-4.571023	0.8823493	0.042071	membrane protein, palmitoylated 7 (MAGUK p55 subfamily member 7)
UP in HEB	**147166**	TRIM16L	-1.625256	0.9210802	0.324152	tripartite motif containing 16-like
UP in HEB	**54903**	MKS1	-1.160545	0.850552	0.447343	Meckel syndrome, type 1
UP in HEB	**4869**	NPM1	-1.110975	0.8926965	0.462981	nucleophosmin (nucleolar phosphoprotein B23, numatrin)
UP in HEB	**2812**	GP1BB	-7.830779	0.907115	0.004392	glycoprotein Ib (platelet), beta polypeptide
UP in HEB	**285855**	RPL7L1	-1.062361	0.8880207	0.478848	ribosomal protein L7-like 1
UP in HEB	**3670**	ISL1	-4.344176	0.9772521	0.049235	ISL LIM homeobox 1
UP in HEB	**2969**	GTF2I	-1.384577	0.9040927	0.383002	general transcription factor IIi
UP in HEB	**78988**	MRP63	-1.330521	0.8866784	0.397625	mitochondrial ribosomal protein 63
UP in HEB	**57156**	TMEM63C	-7.632995	0.9228048	0.005038	transmembrane protein 63C
UP in HEB	**84640**	USP38	-1.10238	0.8512987	0.465748	ubiquitin specific peptidase 38
UP in HEB	**29887**	SNX10	-6.60239	0.8852917	0.010292	sorting nexin 10
UP in HEB	**84992**	PIGY	-1.107519	0.8893985	0.464092	phosphatidylinositol glycan anchor biosynthesis, class Y
UP in HEB	**84650**	EBPL	-1.694518	0.9206713	0.308958	emopamil binding protein-like

UP in HEB	**1871**	E2F3	-2.153859	0.9341654	0.224711	E2F transcription factor 3
UP in HEB	**10966**	RAB40B	-1.416424	0.8567301	0.37464	RAB40B, member RAS oncogene family
UP in HEB	**79930**	DOK3	-1.99103	0.8801269	0.251559	docking protein 3
UP in HEB	**1844**	DUSP2	-9.28193	0.9937686	0.001606	dual specificity phosphatase 2
UP in HEB	**7186**	TRAF2	-1.219894	0.8910075	0.429314	TNF receptor-associated factor 2
UP in HEB	**132228**	LSMEM2	-1.359542	0.8908653	0.389706	leucine-rich single-pass membrane protein 2
UP in HEB	**7965**	AIMP2	-1.319587	0.9018348	0.40065	aminoacyl tRNA synthetase complex-interacting multifunctional protein 2
UP in HEB	**100505385**	IQCJ-SCHIP1	-1.626997	0.9047327	0.323761	IQCJ-SCHIP1 readthrough
UP in HEB	**54913**	RPP25	-5.104461	0.9936886	0.029067	ribonuclease P/MRP 25kDa subunit
UP in HEB	**1285**	COL4A3	-7.948367	0.9673405	0.004049	collagen, type IV, alpha 3 (Goodpasture
UP in HEB	**344558**	SH3RF3	-7.112005	0.83264	0.007229	SH3 domain containing ring finger 3
UP in HEB	**11167**	FSTL1	-1.659417	0.9242537	0.316567	follistatin-like 1
UP in HEB	**6383**	SDC2	-1.975439	0.9265561	0.254293	syndecan 2
UP in HEB	**151871**	DPPA2	-7.922832	0.9147243	0.004121	developmental pluripotency associated 2
UP in HEB	**256714**	MAP7D2	-1.901517	0.845494	0.267662	MAP7 domain containing 2
UP in HEB	**56901**	NDUFA4L2	-2.657579	0.8524455	0.158485	NADH dehydrogenase (ubiquinone) 1 alpha subcomplex, 4-like 2
UP in HEB	**84033**	OBSCN	-9.672425	0.984168	0.001225	obscurin, cytoskeletal calmodulin and titin-interacting RhoGEF
UP in HEB	**5885**	RAD21	-1.158453	0.8948922	0.447993	RAD21 homolog (S. pombe)
UP in HEB	**83394**	PITPNM3	-1.753069	0.8371558	0.29667	PITPNM family member 3
UP in HEB	**6235**	RPS29	-1.136965	0.8946344	0.454715	ribosomal protein S29
UP in HEB	**147804**	TPM3P9	-2.761503	0.8840383	0.14747	tropomyosin 3 pseudogene 9
UP in HEB	**116238**	TLCD1	-1.889424	0.9204935	0.269915	TLC domain containing 1
UP in HEB	**1605**	DAG1	-2.510319	0.9527975	0.175517	dystroglycan 1 (dystrophin-associated glycoprotein 1)
UP in HEB	**1945**	EFNA4	-1.891851	0.9105375	0.269461	ephrin-A4
UP in HEB	**9477**	MED20	-1.647579	0.910973	0.319175	mediator complex subunit 20
UP in HEB	**27161**	AGO2	-1.573345	0.8998524	0.336028	argonaute RISC catalytic component 2
UP in HEB	**790952**	ESRG	-2.066051	0.9173823	0.238812	embryonic stem cell related (non-protein
UP in HEB	**55561**	CDC42BPG	-4.868602	0.8211905	0.03423	CDC42 binding protein kinase gamma (DMPK-like)
UP in HEB	**55100**	WDR70	-1.165791	0.8866873	0.44572	WD repeat domain 70
UP in HEB	**7852**	CXCR4	-12.06631	0.9981155	0.000233	chemokine (C-X-C motif) receptor 4
UP in HEB	**8817**	FGF18	-3.602937	0.9658204	0.082302	fibroblast growth factor 18
UP in HEB	**220992**	ZNF485	-7.693487	0.8958433	0.004831	zinc finger protein 485
UP in HEB	**197342**	EME2	-1.932787	0.8717976	0.261923	essential meiotic endonuclease 1 homolog 2 (S. pombe)
UP in HEB	**51373**	MRPS17	-2.62199	0.95822	0.162443	mitochondrial ribosomal protein S17
UP in HEB	**3728**	JUP	-13.35861	0.9996533	9.52E-05	junction plakoglobin
UP in HEB	**55806**	HR	-5.850934	0.9827013	0.017326	hair growth associated
UP in HEB	**3768**	KCNJ12	-7.409391	0.8669796	0.005882	potassium inwardly-rectifying channel, subfamily J, member 12
UP in HEB	**23108**	RAP1GAP	-3.212214	0.9617669	0.107901	RAP1 GTPase activating protein 2
UP in HEB	**4781**	NFIB	-2.192645	0.9276673	0.21875	nuclear factor I/B
UP in HEB	**4716**	NDUFB10	-1.520399	0.908884	0.34859	NADH dehydrogenase (ubiquinone) 1 beta subcomplex, 10, 22kDa
UP in HEB	**92154**	MTSS1L	-1.088586	0.8864117	0.470222	metastasis suppressor 1-like
UP in HEB	**729359**	PLIN4	-6.080373	0.9546376	0.014778	perilipin 4
UP in HEB	**2539**	G6PD	-1.502718	0.908484	0.352888	glucose-6-phosphate dehydrogenase
UP in HEB	**85439**	STON2	-1.534503	0.8518499	0.345198	stonin 2
UP in HEB	**10314**	LANCL1	-2.20744	0.9421391	0.216518	LanC lantibiotic synthetase component C-like 1 (bacterial)
UP in HEB	**3105**	HLA-A	-1.633888	0.9237737	0.322219	major histocompatibility complex, class I, A
UP in HEB	**84190**	METTL25	-1.867985	0.8193326	0.273956	methyltransferase like 25

UP in HEB	**80863**	PRRT1	-3.151835	0.8993546	0.112513	proline-rich transmembrane protein 1
UP in HEB	**328**	APEX1	-1.503128	0.9082351	0.352788	APEX nuclease (multifunctional DNA repair enzyme) 1
UP in HEB	**192286**	HIGD2A	-1.112411	0.8920476	0.46252	HIG1 hypoxia inducible domain family, member 2A
UP in HEB	**1188**	CLCNKB	-5.561394	0.8324	0.021176	chloride channel, voltage-sensitive Kb
UP in HEB	**54552**	GNL3L	-1.423748	0.8920387	0.372743	guanine nucleotide binding protein-like 3 (nucleolar)-like
UP in HEB	**283070**	LOC28307	-2.039949	0.907444	0.243172	uncharacterized LOC283070
UP in HEB	**7001**	PRDX2	-2.741666	0.9601312	0.149512	peroxiredoxin 2
UP in HEB	**23254**	KAZN	-2.072529	0.9040038	0.237742	kazrin, periplakin interacting protein
UP in HEB	**22824**	HSPA4L	-2.071672	0.9324053	0.237884	heat shock 70kDa protein 4-like
UP in HEB	**729092**	AGAP5	-1.627088	0.8818248	0.323741	ArfGAP with GTPase domain, ankyrin repeat and PH domain 5
UP in HEB	**27090**	ST6GALNAC4	-1.075507	0.8832916	0.474504	ST6 (alpha-N-acetyl-neuraminyl-2,3-beta-galactosyl-1,3)-N-acetylgalactosaminide alpha-2,6-sialyltransferase 4
UP in HEB	**100134868**	LOC100134868	-5.117108	0.8811848	0.028814	uncharacterized LOC100134868
UP in HEB	**7153**	TOP2A	-1.289334	0.9006347	0.40914	topoisomerase (DNA) II alpha 170kDa
UP in HEB	**55616**	ASAP3	-1.181587	0.8755578	0.440866	ArfGAP with SH3 domain, ankyrin repeat and PH domain 3
UP in HEB	**54785**	C17orf59	-1.338658	0.890892	0.395388	chromosome 17 open reading frame 59
UP in HEB	**26232**	FBXO2	-3.867031	0.974932	0.068534	F-box protein 2
UP in HEB	**1021**	CDK6	-1.35973	0.870002	0.389655	cyclin-dependent kinase 6
UP in HEB	**285313**	IGSF10	-6.252219	0.989635	0.013119	immunoglobulin superfamily, member 10
UP in HEB	**948**	CD36	-8.795066	0.9817057	0.002251	CD36 molecule (thrombospondin receptor)
UP in HEB	**4982**	TNFRSF11B	-3.405887	0.9698562	0.094346	tumor necrosis factor receptor superfamily, member 11b
UP in HEB	**3229**	HOXC13	-8.982994	0.9687183	0.001976	homeobox C13
UP in HEB	**26524**	LATS2	-1.336012	0.8868206	0.396114	LATS, large tumor suppressor, homolog 2 (Drosophila)
UP in HEB	**80303**	EFHD1	-11.07213	0.9954664	0.000464	EF-hand domain family, member D1
UP in HEB	**5166**	PDK4	-5.991547	0.9971465	0.015717	pyruvate dehydrogenase kinase, isozyme 4
UP in HEB	**9143**	SYNGR3	-8.569856	0.9534197	0.002632	synaptogyrin 3
UP in HEB	**3136**	HLA-H	-3.232339	0.9442904	0.106407	major histocompatibility complex, class I, H (pseudogene)
UP in HEB	**1628**	DBP	-3.984696	0.9680783	0.063167	D site of albumin promoter (albumin D-box) binding protein
UP in HEB	**9702**	CEP57	-1.261513	0.8910786	0.417106	centrosomal protein 57kDa
UP in HEB	**54555**	DDX49	-1.0243	0.8810425	0.491649	DEAD (Asp-Glu-Ala-Asp) box polypeptide
UP in HEB	**6117**	RPA1	-1.422628	0.904066	0.373032	replication protein A1, 70kDa
UP in HEB	**5426**	POLE	-1.564735	0.9046438	0.33804	polymerase (DNA directed), epsilon, catalytic subunit
UP in HEB	**3148**	HMGB2	-2.513557	0.9530197	0.175123	high mobility group box 2
UP in HEB	**642846**	LOC642846	-1.569274	0.8935321	0.336978	DEAD/H (Asp-Glu-Ala-Asp/His) box polypeptide 11-like
UP in HEB	**50617**	ATP6V0A4	-6.028695	0.8461696	0.015317	ATPase, H+ transporting, lysosomal V0 subunit a4
UP in HEB	**5858**	PZP	-7.559696	0.883416	0.0053	pregnancy-zone protein
UP in HEB	**54742**	LY6K	-6.283754	0.9957864	0.012835	lymphocyte antigen 6 complex, locus K
UP in HEB	**158405**	KIAA1958	-2.106607	0.8700286	0.232192	KIAA1958
UP in HEB	**100292680**	LOC100292680	-6.275246	0.9898306	0.012911	uncharacterized LOC100292680
UP in HEB	**55272**	IMP3	-1.748004	0.9259872	0.297713	IMP3, U3 small nucleolar ribonucleoprotein, homolog (yeast)
UP in HEB	**90632**	LINC0047	-10.9349	0.9992711	0.000511	long intergenic non-protein coding RNA 473

UP in HEB	**2810**	SFN	-9.790698	0.9990488	0.001129	stratifin
UP in HEB	**80759**	KHDC1	-3.387363	0.932023	0.095566	KH homology domain containing 1
UP in HEB	**6542**	SLC7A2	-11.47337	0.9966754	0.000352	solute carrier family 7 (cationic amino acid transporter, y+ system), member 2
UP in HEB	**64900**	LPIN3	-4.918162	0.962398	0.033074	lipin 3
UP in HEB	**84915**	FAM222A	-5.72792	0.8085053	0.018868	family with sequence similarity 222, member
UP in HEB	**5493**	PPL	-8.222162	0.9950664	0.003349	periplakin
UP in HEB	**284217**	LAMA1	-1.124404	0.8206482	0.458691	laminin, alpha 1
UP in HEB	**7037**	TFRC	-1.608381	0.9233292	0.327966	transferrin receptor (p90, CD71)
UP in HEB	**150381**	LOC15038	-1.110778	0.8557345	0.463044	uncharacterized LOC150381
UP in HEB	**215**	ABCD1	-2.448896	0.9462816	0.183151	ATP-binding cassette, sub-family D (ALD), member 1
UP in HEB	**2653**	GCSH	-2.088954	0.9403879	0.235051	glycine cleavage system protein H (aminomethyl carrier)
UP in HEB	**414216**	BMS1P2	-1.29535	0.8177769	0.407437	BMS1 pseudogene 2
UP in HEB	**2548**	GAA	-1.534579	0.9018792	0.34518	glucosidase, alpha; acid
UP in HEB	**100885848**	PTGES3L	-1.988126	0.8086742	0.252066	prostaglandin E synthase 3 (cytosolic)-like
UP in HEB	**55003**	PAK1IP1	-1.731467	0.9224936	0.301146	PAK1 interacting protein 1
UP in HEB	**994**	CDC25B	-1.965757	0.9277295	0.256005	cell division cycle 25B
UP in HEB	**64798**	DEPTOR	-3.306962	0.9578111	0.101043	DEP domain containing MTOR-interacting protein
UP in HEB	**5613**	PRKX	-3.315832	0.9440059	0.100423	protein kinase, X-linked
UP in HEB	**6405**	SEMA3F	-9.899055	0.9934308	0.001047	sema domain, immunoglobulin domain (Ig), short basic domain, secreted, (semaphorin) 3F
UP in HEB	**80000**	GREB1L	-1.583979	0.8658684	0.333561	growth regulation by estrogen in breast cancer-like
UP in HEB	**9620**	CELSR1	-1.051596	0.8618682	0.482434	cadherin, EGF LAG seven-pass G-type
UP in HEB	**489**	ATP2A3	-7.522441	0.9599268	0.005439	ATPase, Ca++ transporting, ubiquitous
UP in HEB	**100137049**	PLA2G4B	-2.301496	0.8102921	0.202853	phospholipase A2, group IVB (cytosolic)
UP in HEB	**622**	BDH1	-6.935376	0.9889061	0.00817	3-hydroxybutyrate dehydrogenase, type 1
UP in HEB	**83468**	GLT8D2	-4.988789	0.9840525	0.031494	glycosyltransferase 8 domain containing 2
UP in HEB	**5089**	PBX2	-1.26631	0.8936654	0.415722	pre-B-cell leukemia homeobox 2
UP in HEB	**29941**	PKN3	-5.290913	0.9919818	0.025543	protein kinase N3
UP in HEB	**26150**	RIBC2	-7.360481	0.8615126	0.006085	RIB43A domain with coiled-coils 2
UP in HEB	**5139**	PDE3A	-11.1258	0.9993066	0.000448	phosphodiesterase 3A, cGMP-inhibited
UP in HEB	**80174**	DBF4B	-1.040495	0.8718243	0.486161	DBF4 homolog B (S. cerevisiae)
UP in HEB	**83480**	PUS3	-1.741837	0.9118264	0.298989	pseudouridylate synthase 3
UP in HEB	**8228**	PNPLA4	-8.934674	0.9672783	0.002044	patatin-like phospholipase domain containing
UP in HEB	**53637**	S1PR5	-3.392317	0.8873451	0.095238	sphingosine-1-phosphate receptor 5
UP in HEB	**10473**	HMGN4	-1.688668	0.9234893	0.310213	high mobility group nucleosomal binding domain 4
UP in HEB	**57407**	NMRAL1	-2.006103	0.9365211	0.248945	NmrA-like family domain containing 1
UP in HEB	**6173**	RPL36A	-1.157091	0.8952744	0.448416	ribosomal protein L36a
UP in HEB	**84321**	THOC3	-1.911552	0.9270628	0.265807	THO complex 3
UP in HEB	**10813**	UTP14A	-1.016778	0.879718	0.494219	UTP14, U3 small nucleolar ribonucleoprotein, homolog A (yeast)
UP in HEB	**57158**	JPH2	-3.157412	0.938859	0.112079	junctophilin 2
UP in HEB	**5728**	PTEN	-1.514153	0.9059505	0.350102	phosphatase and tensin homolog
UP in HEB	**5962**	RDX	-1.489116	0.9056927	0.356231	radixin
UP in HEB	**58505**	OSTC	-1.153501	0.8930699	0.449533	oligosaccharyltransferase complex subunit (non-catalytic)
UP in HEB	**55146**	ZDHHC4	-1.090028	0.8851583	0.469752	zinc finger, DHHC-type containing 4
UP in HEB	**6157**	RPL27A	-1.093585	0.8915676	0.468595	ribosomal protein L27a
UP in HEB	**7374**	UNG	-1.75575	0.9245115	0.296119	uracil-DNA glycosylase
UP in HEB	**28232**	SLCO3A1	-2.691344	0.9572333	0.154819	solute carrier organic anion transporter family, member 3A1

UP in HEB	**441177**	LINC0060	-10.52748	0.9926218	0.000678	long intergenic non-protein coding RNA 602
UP in HEB	**57181**	SLC39A10	-1.116707	0.8839583	0.461145	solute carrier family 39 (zinc transporter), member 10
UP in HEB	**25929**	GEMIN5	-1.548504	0.9045016	0.341864	gem (nuclear organelle) associated protein 5
UP in HEB	**10723**	SLC12A7	-8.77062	0.9964709	0.00229	solute carrier family 12 (potassium/chloride transporters), member 7
UP in HEB	**23389**	MED13L	-1.336849	0.8926521	0.395884	mediator complex subunit 13-like
UP in HEB	**5900**	RALGDS	-1.554634	0.901417	0.340415	ral guanine nucleotide dissociation stimulator
UP in HEB	**28970**	C11orf54	-1.293057	0.8235106	0.408085	chromosome 11 open reading frame 54
UP in HEB	**57211**	GPR126	-10.9901	0.9951909	0.000492	G protein-coupled receptor 126
UP in HEB	**22948**	CCT5	-1.17325	0.89635	0.443421	chaperonin containing TCP1, subunit 5
UP in HEB	**3017**	HIST1H2B	-2.494628	0.9416947	0.177436	histone cluster 1, H2bd
UP in HEB	**4832**	NME3	-2.811118	0.9634825	0.142485	NME/NM23 nucleoside diphosphate kinase 3
UP in HEB	**10171**	RCL1	-1.004634	0.8708109	0.498396	RNA terminal phosphate cyclase-like 1
UP in HEB	**9894**	TELO2	-1.662324	0.9190979	0.31593	TEL2, telomere maintenance 2, homolog (S. cerevisiae)
UP in HEB	**64866**	CDCP1	-2.440632	0.9397035	0.184203	CUB domain containing protein 1
UP in HEB	**55283**	MCOLN3	-6.902878	0.807412	0.008357	mucolipin 3
UP in HEB	**196441**	ZFC3H1	-1.228928	0.8898964	0.426634	zinc finger, C3H1-type containing
UP in HEB	**81621**	KAZALD1	-1.831855	0.9053194	0.280903	Kazal-type serine peptidase inhibitor domain
UP in HEB	**8473**	OGT	-1.001176	0.8842694	0.499593	O-linked N-acetylglucosamine (GlcNAc) transferase
UP in HEB	**85028**	SNHG12	-1.090281	0.886305	0.46967	small nucleolar RNA host gene 12 (non-protein coding)
UP in HEB	**57494**	RIMKLB	-3.281536	0.9705851	0.102839	ribosomal modification protein rimK-like family member B
UP in HEB	**55353**	LAPTM4B	-2.59661	0.9595178	0.165326	lysosomal protein transmembrane 4 beta
UP in HEB	**166929**	SGMS2	-1.133366	0.8399915	0.455851	sphingomyelin synthase 2
UP in HEB	**10058**	ABCB6	-2.281328	0.9392323	0.205708	ATP-binding cassette, sub-family B (MDR/TAP), member 6
UP in HEB	**10675**	CSPG5	-6.380822	0.8621704	0.012	chondroitin sulfate proteoglycan 5 (neuroglycan C)
UP in HEB	**10553**	HTATIP2	-13.24218	0.9996	0.000103	HIV-1 Tat interactive protein 2, 30kDa
UP in HEB	**81786**	TRIM7	-6.09849	0.990924	0.014594	tripartite motif containing 7
UP in HEB	**117581**	TWIST2	-10.25896	0.9908617	0.000816	twist basic helix-loop-helix transcription
UP in HEB	**875**	CBS	-6.400427	0.9974665	0.011838	cystathionine-beta-synthase
UP in HEB	**79621**	RNASEH2	-2.968464	0.9505129	0.127762	ribonuclease H2, subunit B
UP in HEB	**55244**	SLC47A1	-3.138898	0.8859761	0.113527	solute carrier family 47, member 1
UP in HEB	**64324**	NSD1	-1.420294	0.8969456	0.373636	nuclear receptor binding SET domain protein
UP in HEB	**4059**	BCAM	-5.994056	0.9810301	0.01569	basal cell adhesion molecule (Lutheran blood group)
UP in HEB	**10626**	TRIM16	-1.369696	0.9005991	0.386973	tripartite motif containing 16
UP in HEB	**9351**	SLC9A3R2	-4.016995	0.9785055	0.061768	solute carrier family 9, subfamily A (NHE3, cation proton antiporter 3), member 3 regulator 2
UP in HEB	**8463**	TEAD2	-8.528594	0.9851548	0.002708	TEA domain family member 2
UP in HEB	**121536**	AEBP2	-1.623594	0.9165822	0.324526	AE binding protein 2
UP in HEB	**666**	BOK	-7.251088	0.9627002	0.006565	BCL2-related ovarian killer
UP in HEB	**8880**	FUBP1	-1.856403	0.9271072	0.276164	far upstream element (FUSE) binding protein
UP in HEB	**400720**	ZNF772	-2.266103	0.8155191	0.207891	zinc finger protein 772
UP in HEB	**6125**	RPL5	-1.752548	0.9266894	0.296777	ribosomal protein L5
UP in HEB	**55847**	CISD1	-1.533362	0.9058616	0.345471	CDGSH iron sulfur domain 1
UP in HEB	**9753**	ZSCAN12	-1.724251	0.8948833	0.302656	zinc finger and SCAN domain containing 12
UP in HEB	**201562**	PTPLB	-1.749771	0.9237648	0.297349	protein tyrosine phosphatase-like (proline instead of catalytic arginine), member b
UP in HEB	**152217**	LOC15221	-1.104349	0.8809359	0.465112	uncharacterized LOC152217
UP in HEB	**149076**	ZNF362	-2.089637	0.8871495	0.23494	zinc finger protein 362

UP in HEB	**10733**	PLK4	-1.474114	0.8899408	0.359954	polo-like kinase 4
UP in HEB	**9469**	CHST3	-1.175607	0.8878429	0.442697	carbohydrate (chondroitin 6) sulfotransferase
UP in HEB	**25957**	PNISR	-1.052791	0.8851317	0.482035	PNN-interacting serine/arginine-rich protein
UP in HEB	**3339**	HSPG2	-2.887607	0.9606557	0.135128	heparan sulfate proteoglycan 2
UP in HEB	**6347**	CCL2	-1.181607	0.8824826	0.44086	chemokine (C-C motif) ligand 2
UP in HEB	**5867**	RAB4A	-5.714538	0.9808434	0.019044	RAB4A, member RAS oncogene family
UP in HEB	**54888**	NSUN2	-1.269745	0.8988302	0.414733	NOP2/Sun RNA methyltransferase family, member 2
UP in HEB	**6627**	SNRPA1	-1.268095	0.8995413	0.415208	small nuclear ribonucleoprotein polypeptide
UP in HEB	**1059**	CENPB	-1.084035	0.8875051	0.471708	centromere protein B, 80kDa
UP in HEB	**85358**	SHANK3	-4.373017	0.9205202	0.04826	SH3 and multiple ankyrin repeat domains 3
UP in HEB	**154**	ADRB2	-6.509775	0.9855726	0.010974	adrenoceptor beta 2, surface
UP in HEB	**84295**	PHF6	-1.432357	0.9022259	0.370525	PHD finger protein 6
UP in HEB	**84062**	DTNBP1	-9.040877	0.9908173	0.001899	dystrobrevin binding protein 1
UP in HEB	**57470**	LRRC47	-1.014017	0.879878	0.495166	leucine rich repeat containing 47
UP in HEB	**79037**	PVRIG	-3.032269	0.8883052	0.122235	poliovirus receptor related immunoglobulin domain containing
UP in HEB	**79711**	IPO4	-1.154696	0.8890163	0.449161	importin 4
UP in HEB	**3712**	IVD	-1.085198	0.8845627	0.471328	isovaleryl-CoA dehydrogenase
UP in HEB	**11156**	PTP4A3	-4.44445	0.8419027	0.045929	protein tyrosine phosphatase type IVA, member 3
UP in HEB	**9002**	F2RL3	-7.20806	0.9457127	0.006763	coagulation factor II (thrombin) receptor-like
UP in HEB	**23336**	SYNM	-6.79786	0.9932441	0.008988	synemin, intermediate filament protein
UP in HEB	**64710**	NUCKS1	-1.125325	0.8923054	0.458399	nuclear casein kinase and cyclin-dependent kinase substrate 1
UP in HEB	**165055**	CCDC138	-2.304561	0.9301741	0.202422	coiled-coil domain containing 138
UP in HEB	**10469**	TIMM44	-1.045809	0.8830338	0.484373	translocase of inner mitochondrial membrane 44 homolog (yeast)
UP in HEB	**114904**	C1QTNF6	-4.230576	0.9842747	0.053268	C1q and tumor necrosis factor related protein
UP in HEB	**8844**	KSR1	-4.0081	0.9594201	0.06215	kinase suppressor of ras 1
UP in HEB	**79228**	THOC6	-3.099367	0.9668249	0.11668	THO complex 6 homolog (Drosophila)
UP in HEB	**83903**	GSG2	-1.569754	0.8900919	0.336866	germ cell associated 2 (haspin)
UP in HEB	**54539**	NDUFB11	-1.208606	0.8966967	0.432686	NADH dehydrogenase (ubiquinone) 1 beta subcomplex, 11, 17.3kDa
UP in HEB	**27300**	ZNF544	-5.635479	0.9733941	0.020116	zinc finger protein 544
UP in HEB	**8294**	HIST1H4I	-7.489179	0.8758689	0.005566	histone cluster 1, H4i
UP in HEB	**79960**	PHF17	-1.193938	0.8707576	0.437108	PHD finger protein 17
UP in HEB	**10608**	MXD4	-1.214141	0.8899052	0.43103	MAX dimerization protein 4
UP in HEB	**1159**	CKMT1B	-4.768968	0.9897861	0.036677	creatine kinase, mitochondrial 1B
UP in HEB	**3110**	MNX1	-7.830779	0.907115	0.004392	motor neuron and pancreas homeobox 1
UP in HEB	**8125**	ANP32A	-1.443071	0.9060128	0.367784	acidic (leucine-rich) nuclear phosphoprotein 32 family, member A
UP in HEB	**148327**	CREB3L4	-1.429318	0.8781713	0.371306	cAMP responsive element binding protein 3-like 4
UP in HEB	**7942**	TFEB	-2.998527	0.9430992	0.125128	transcription factor EB
UP in HEB	**284611**	FAM102B	-1.792229	0.9198091	0.288726	family with sequence similarity 102, member
UP in HEB	**84259**	DCUN1D5	-1.227454	0.8376713	0.42707	DCN1, defective in cullin neddylation 1, domain containing 5 (S. cerevisiae)
UP in HEB	**2161**	F12	-3.754219	0.9617669	0.074108	coagulation factor XII (Hageman factor)
UP in HEB	**26049**	FAM169A	-2.192956	0.813839	0.218703	family with sequence similarity 169, member
UP in HEB	**160418**	TMTC3	-1.256684	0.8406137	0.418505	transmembrane and tetratricopeptide repeat containing 3
UP in HEB	**4507**	MTAP	-8.67015	0.9945508	0.002455	methylthioadenosine phosphorylase
UP in HEB	**4605**	MYBL2	-2.351576	0.9506285	0.195932	v-myb myeloblastosis viral oncogene homolog (avian)-like 2
UP in HEB	**26095**	PTPN20B	-7.315904	0.8563657	0.006276	protein tyrosine phosphatase, non-receptor type 20B

UP in HEB	**6456**	SH3GL2	-9.988211	0.9882572	0.000985	SH3-domain GRB2-like 2
UP in HEB	**3708**	ITPR1	-1.437996	0.9000836	0.36908	inositol 1,4,5-trisphosphate receptor, type 1
UP in HEB	**51174**	TUBD1	-1.177573	0.8487297	0.442095	tubulin, delta 1
UP in HEB	**55339**	WDR33	-1.112125	0.8818959	0.462612	WD repeat domain 33
UP in HEB	**54953**	C1orf27	-1.053439	0.8435383	0.481818	chromosome 1 open reading frame 27
UP in HEB	**25906**	ANAPC15	-1.135813	0.8893985	0.455078	anaphase promoting complex subunit 15
UP in HEB	**112817**	HOGA1	-1.324931	0.8581347	0.399168	4-hydroxy-2-oxoglutarate aldolase 1
UP in HEB	**752014**	CEMP1	-1.364484	0.8890608	0.388373	cementum protein 1
UP in HEB	**642361**	LOC64236	-1.065945	0.83368	0.47766	uncharacterized LOC642361
UP in HEB	**10351**	ABCA8	-4.987652	0.9009369	0.031519	ATP-binding cassette, sub-family A (ABC1), member 8
UP in HEB	**3945**	LDHB	-1.026247	0.8884741	0.490986	lactate dehydrogenase B
UP in HEB	**6615**	SNAI1	-4.729139	0.9669493	0.037704	snail homolog 1 (Drosophila)
UP in HEB	**51163**	DBR1	-1.05786	0.8633972	0.480344	debranching enzyme homolog 1 (S.
UP in HEB	**57523**	NYNRIN	-7.564785	0.883416	0.005282	NYN domain and retroviral integrase
UP in HEB	**2296**	FOXC1	-2.508755	0.9376678	0.175707	forkhead box C1
UP in HEB	**79814**	AGMAT	-5.036122	0.967865	0.030477	agmatine ureohydrolase (agmatinase)
UP in HEB	**9537**	TP53I11	-8.447083	0.9472683	0.002865	tumor protein p53 inducible protein 11
UP in HEB	**286077**	FAM83H	-11.21538	0.9971821	0.000421	family with sequence similarity 83, member
UP in HEB	**100048912**	CDKN2B-AS1	-1.634716	0.8164702	0.322034	CDKN2B antisense RNA 1
UP in HEB	**54107**	POLE3	-1.299195	0.9001991	0.406353	polymerase (DNA directed), epsilon 3, accessory subunit
UP in HEB	**92106**	OXNAD1	-1.176878	0.8604814	0.442308	oxidoreductase NAD-binding domain containing 1
UP in HEB	**9189**	ZBED1	-2.787078	0.9557843	0.144879	zinc finger, BED-type containing 1
UP in HEB	**2624**	GATA2	-10.61594	0.9933152	0.000637	GATA binding protein 2
UP in HEB	**4835**	NQO2	-3.228587	0.9725319	0.106684	NAD(P)H dehydrogenase, quinone 2
UP in HEB	**79940**	LINC0047	-3.721898	0.9218803	0.075787	long intergenic non-protein coding RNA 472
UP in HEB	**60370**	AVPI1	-3.680948	0.9805679	0.077969	arginine vasopressin-induced 1
UP in HEB	**57109**	REXO4	-1.139571	0.890083	0.453895	REX4, RNA exonuclease 4 homolog (S. cerevisiae)
UP in HEB	**84268**	RPAIN	-1.029868	0.8825804	0.489755	RPA interacting protein
UP in HEB	**389641**	LOC38964	-2.560491	0.9024481	0.169518	uncharacterized LOC389641
UP in HEB	**646719**	LOC64671	-1.579432	0.8878074	0.334614	uncharacterized LOC646719
UP in HEB	**64699**	TMPRSS3	-7.546894	0.9516596	0.005348	transmembrane protease, serine 3
UP in HEB	**117166**	WFIKKN1	-6.886713	0.8038473	0.008451	WAP, follistatin/kazal, immunoglobulin, kunitz and netrin domain containing 1
UP in HEB	**6146**	RPL22	-2.071826	0.9411791	0.237858	ribosomal protein L22
UP in HEB	**55922**	NKRF	-1.608197	0.913062	0.328008	NFKB repressing factor
UP in HEB	**9721**	GPRIN2	-7.785725	0.9033993	0.004532	G protein regulated inducer of neurite outgrowth 2
UP in HEB	**89941**	RHOT2	-1.271549	0.8966078	0.414215	ras homolog family member T2
UP in HEB	**10915**	TCERG1	-1.072721	0.8868917	0.475421	transcription elongation regulator 1
UP in HEB	**8218**	CLTCL1	-1.510772	0.8768557	0.350923	clathrin, heavy chain-like 1
UP in HEB	**83452**	RAB33B	-1.292393	0.8050118	0.408273	RAB33B, member RAS oncogene family
UP in HEB	**1806**	DPYD	-1.233425	0.8463474	0.425307	dihydropyrimidine dehydrogenase
UP in HEB	**4254**	KITLG	-1.440882	0.8797891	0.368342	KIT ligand
UP in HEB	**677803**	SNORA15	-7.269906	0.8512454	0.006479	small nucleolar RNA, H/ACA box 15
UP in HEB	**254439**	C11orf86	-8.058533	0.9856081	0.003751	chromosome 11 open reading frame 86
UP in HEB	**91768**	CABLES1	-6.865954	0.9705407	0.008573	Cdk5 and Abl enzyme substrate 1
UP in HEB	**55743**	CHFR	-7.950702	0.9580155	0.004042	checkpoint with forkhead and ring finger domains, E3 ubiquitin protein ligase
UP in HEB	**1027**	CDKN1B	-1.050139	0.8845005	0.482922	cyclin-dependent kinase inhibitor 1B (p27,
UP in HEB	**54433**	GAR1	-1.29981	0.8924387	0.40618	GAR1 ribonucleoprotein homolog (yeast)
UP in HEB	**28959**	TMEM176	-6.226894	0.9147599	0.013351	transmembrane protein 176B

UP in HEB	**1468**	SLC25A10	-2.520644	0.9525308	0.174265	solute carrier family 25 (mitochondrial carrier; dicarboxylate transporter), member 10
UP in HEB	**705**	BYSL	-1.41201	0.9002347	0.375788	bystin-like
UP in HEB	**8739**	HRK	-6.063395	0.8714687	0.014953	harakiri, BCL2 interacting protein (contains only BH3 domain)
UP in HEB	**317762**	CCDC85C	-5.997147	0.9919729	0.015656	coiled-coil domain containing 85C
UP in HEB	**5909**	RAP1GAP	-8.09728	0.9774477	0.003652	RAP1 GTPase activating protein
UP in HEB	**122953**	JDP2	-1.247506	0.8788735	0.421176	Jun dimerization protein 2
UP in HEB	**4726**	NDUFS6	-1.569034	0.9100308	0.337034	NADH dehydrogenase (ubiquinone) Fe-S protein 6, 13kDa (NADH-coenzyme Q reductase)
UP in HEB	**54541**	DDIT4	-2.076596	0.9412946	0.237073	DNA-damage-inducible transcript 4
UP in HEB	**9047**	SH2D2A	-3.288644	0.8390314	0.102334	SH2 domain containing 2A
UP in HEB	**10514**	MYBBP1	-1.376712	0.902377	0.385095	MYB binding protein (P160) 1a
UP in HEB	**7036**	TFR2	-9.338365	0.9781588	0.001545	transferrin receptor 2
UP in HEB	**51522**	TMEM14C	-1.250943	0.8992302	0.420174	transmembrane protein 14C
UP in HEB	**654434**	LINC0033	-1.327197	0.8716109	0.398542	long intergenic non-protein coding RNA 338
UP in HEB	**51155**	HN1	-1.149576	0.8941277	0.450758	hematological and neurological expressed 1
UP in HEB	**26136**	TES	-7.095211	0.9983377	0.007314	testis derived transcript (3 LIM domains)
UP in HEB	**150223**	YDJC	-3.037837	0.9658648	0.121764	YdjC homolog (bacterial)
UP in HEB	**55766**	H2AFJ	-3.811275	0.9704784	0.071235	H2A histone family, member J
UP in HEB	**100507547**	LOC100507547	-1.606288	0.8800114	0.328442	uncharacterized LOC100507547
UP in HEB	**100874032**	PRRT3-	-2.830587	0.9555532	0.140575	PRRT3 antisense RNA 1
UP in HEB	**389906**	LOC38990	-8.145122	0.9881771	0.003532	zinc finger protein 839 pseudogene
UP in HEB	**115273**	RAB42	-3.916979	0.9765232	0.066202	RAB42, member RAS oncogene family
UP in HEB	**51110**	LACTB2	-1.900697	0.9154088	0.267814	lactamase, beta 2
UP in HEB	**54491**	FAM105A	-2.749208	0.9497218	0.148733	family with sequence similarity 105, member
UP in HEB	**81567**	TXNDC5	-1.072746	0.8899675	0.475413	thioredoxin domain containing 5 (endoplasmic reticulum)
UP in HEB	**26053**	AUTS2	-7.481127	0.8749622	0.005597	autism susceptibility candidate 2
UP in HEB	**55172**	DNAAF2	-1.667977	0.8952833	0.314694	dynein, axonemal, assembly factor 2
UP in HEB	**140738**	TMEM37	-7.97728	0.9184045	0.003968	transmembrane protein 37
UP in HEB	**5198**	PFAS	-1.661947	0.9216225	0.316012	phosphoribosylformylglycinamidine synthase
UP in HEB	**3315**	HSPB1	-1.014102	0.887594	0.495136	heat shock 27kDa protein 1
UP in HEB	**3207**	HOXA11	-5.605427	0.9693317	0.02054	homeobox A11
UP in HEB	**23199**	GSE1	-2.060849	0.9232315	0.239675	Gse1 coiled-coil protein
UP in HEB	**7023**	TFAP4	-2.62562	0.9525664	0.162035	transcription factor AP-4 (activating enhancer binding protein 4)
UP in HEB	**218**	ALDH3A1	-8.186527	0.9325831	0.003432	aldehyde dehydrogenase 3 family, member
UP in HEB	**29105**	C16orf80	-1.198248	0.8936743	0.435804	chromosome 16 open reading frame 80
UP in HEB	**1741**	DLG3	-1.282303	0.873371	0.411139	discs, large homolog 3 (Drosophila)
UP in HEB	**8975**	USP13	-1.36022	0.8876296	0.389523	ubiquitin specific peptidase 13 (isopeptidase
UP in HEB	**23464**	GCAT	-1.358154	0.8923676	0.390081	glycine C-acetyltransferase
UP in HEB	**89958**	SAPCD2	-2.084412	0.9375344	0.235792	suppressor APC domain containing 2
UP in HEB	**283130**	SLC25A45	-1.764474	0.8679574	0.294334	solute carrier family 25, member 45
UP in HEB	**4330**	MN1	-8.306821	0.939819	0.003158	meningioma (disrupted in balanced translocation) 1
UP in HEB	**5091**	PC	-3.32939	0.9737675	0.099484	pyruvate carboxylase
UP in HEB	**6776**	STAT5A	-4.599913	0.8086298	0.041237	signal transducer and activator of transcription 5A
UP in HEB	**10991**	SLC38A3	-7.47032	0.8732199	0.005639	solute carrier family 38, member 3
UP in HEB	**2199**	FBLN2	-3.760981	0.9729319	0.073762	fibulin 2
UP in HEB	**8358**	HIST1H3B	-7.445705	0.8704731	0.005736	histone cluster 1, H3b
UP in HEB	**4286**	MITF	-1.105182	0.8650061	0.464844	microphthalmia-associated transcription
UP in HEB	**85236**	HIST1H2B	-1.047275	0.8876385	0.483881	histone cluster 1, H2bk

UP in HEB	**57624**	NYAP2	-4.618386	0.8115188	0.040712	neuronal tyrosine-phosphorylated phosphoinositide-3-kinase adaptor 2
UP in HEB	**2192**	FBLN1	-5.719781	0.9967998	0.018975	fibulin 1
UP in HEB	**7483**	WNT9A	-5.357552	0.9399879	0.02439	wingless-type MMTV integration site family, member 9A
UP in HEB	**51562**	MBIP	-1.212365	0.8834071	0.431561	MAP3K12 binding inhibitory protein 1
UP in HEB	**9644**	SH3PXD2	-1.875793	0.9236848	0.272477	SH3 and PX domains 2A
UP in HEB	**644068**	RPS14P3	-4.131074	0.8084609	0.057072	ribosomal protein S14 pseudogene 3
UP in HEB	**7157**	TP53	-1.868963	0.9209913	0.27377	tumor protein p53
UP in HEB	**326625**	MMAB	-1.316537	0.8638861	0.401497	methylmalonic aciduria (cobalamin deficiency) cblB type
UP in HEB	**57224**	NHSL1	-3.164434	0.9083062	0.111535	NHS-like 1
UP in HEB	**9314**	KLF4	-2.139981	0.867993	0.226883	Kruppel-like factor 4 (gut)
UP in HEB	**58190**	CTDSP1	-1.442665	0.9044038	0.367887	CTD (carboxy-terminal domain, RNA polymerase II, polypeptide A) small phosphatase 1
UP in HEB	**255324**	EPGN	-2.410567	0.8854428	0.188082	epithelial mitogen
UP in HEB	**9324**	HMGN3	-10.71267	0.9996089	0.000596	high mobility group nucleosomal binding domain 3
UP in HEB	**7407**	VARS	-1.39447	0.9041816	0.380384	valyl-tRNA synthetase
UP in HEB	**5586**	PKN2	-1.70631	0.9198002	0.306443	protein kinase N2
UP in HEB	**6835**	SURF2	-1.383277	0.9013014	0.383347	surfeit 2
UP in HEB	**79366**	HMGN5	-1.461931	0.8857806	0.363007	high mobility group nucleosome binding domain 5
UP in HEB	**6046**	BRD2	-1.057264	0.8877629	0.480542	bromodomain containing 2
UP in HEB	**9857**	CEP350	-1.009964	0.8340089	0.496559	centrosomal protein 350kDa
UP in HEB	**57509**	MTUS1	-9.100864	0.9959198	0.001821	microtubule associated tumor suppressor 1
UP in HEB	**6170**	RPL39	-1.235198	0.8996124	0.424784	ribosomal protein L39
UP in HEB	**8467**	SMARCA5	-1.454348	0.9031771	0.36492	SWI/SNF related, matrix associated, actin dependent regulator of chromatin, subfamily a, member 5
UP in HEB	**115**	ADCY9	-2.458867	0.9414991	0.181889	adenylate cyclase 9
UP in HEB	**389816**	LRRC26	-8.744834	0.9603979	0.002331	leucine rich repeat containing 26
UP in HEB	**8839**	WISP2	-11.82642	0.9975999	0.000275	WNT1 inducible signaling pathway protein 2
UP in HEB	**79603**	CERS4	-8.646259	0.9567888	0.002496	ceramide synthase 4
UP in HEB	**387509**	GPR153	-2.077076	0.8976745	0.236994	G protein-coupled receptor 153
UP in HEB	**11244**	ZHX1	-1.157599	0.8841538	0.448258	zinc fingers and homeoboxes 1
UP in HEB	**221756**	MGC3937 2	-6.668176	0.9432325	0.009833	serpin peptidase inhibitor, clade B (ovalbumin), member 9 pseudogene
UP in HEB	**2535**	FZD2	-1.136699	0.8374669	0.454799	frizzled family receptor 2
UP in HEB	**399753**	LOC39975	-3.23744	0.9102708	0.106031	uncharacterized LOC399753
UP in HEB	**644656**	LOC64465	-1.65929	0.8908653	0.316595	uncharacterized LOC644656
UP in HEB	**151903**	CCDC12	-1.28296	0.8975768	0.410951	coiled-coil domain containing 12
UP in HEB	**225689**	MAPK15	-6.940838	0.9297474	0.00814	mitogen-activated protein kinase 15
UP in HEB	**3107**	HLA-C	-1.254673	0.8996391	0.419089	major histocompatibility complex, class I, C
UP in HEB	**91975**	ZNF300	-4.558354	0.9773766	0.042442	zinc finger protein 300
UP in HEB	**11163**	NUDT4	-1.426623	0.8978346	0.372001	nudix (nucleoside diphosphate linked moiety X)-type motif 4
UP in HEB	**25938**	HEATR5A	-2.481901	0.9380678	0.179008	HEAT repeat containing 5A
UP in HEB	**6566**	SLC16A1	-1.778445	0.9253649	0.291497	solute carrier family 16, member 1 (monocarboxylic acid transporter 1)
UP in HEB	**23532**	PRAME	-10.33204	0.9913862	0.000776	preferentially expressed antigen in melanoma
UP in HEB	**10076**	PTPRU	-2.111937	0.9333831	0.231336	protein tyrosine phosphatase, receptor type,
UP in HEB	**160335**	TMTC2	-1.587984	0.8238128	0.332636	transmembrane and tetratricopeptide repeat containing 2
UP in HEB	**55819**	RNF130	-1.427566	0.8980123	0.371757	ring finger protein 130
UP in HEB	**30012**	TLX3	-9.639039	0.9835813	0.001254	T-cell leukemia homeobox 3

UP in HEB	**8814**	CDKL1	-3.836602	0.975172	0.069995	cyclin-dependent kinase-like 1 (CDC2-related kinase)
UP in HEB	**283951**	C16orf91	-1.851257	0.9221381	0.277151	chromosome 16 open reading frame 91
UP in HEB	**5939**	RBMS2	-1.008076	0.8509165	0.497209	RNA binding motif, single stranded interacting protein 2
UP in HEB	**151742**	PPM1L	-2.464782	0.9253116	0.181145	protein phosphatase, Mg2+/Mn2+ dependent,
UP in HEB	**5866**	RAB3IL1	-1.308653	0.8868651	0.403698	RAB3A interacting protein (rabin3)-like 1
UP in HEB	**727936**	GXYLT2	-1.555396	0.8659751	0.340235	glucoside xylosyltransferase 2
UP in HEB	**3606**	IL18	-7.859617	0.992204	0.004305	interleukin 18 (interferon-gamma-inducing
UP in HEB	**26153**	KIF26A	-11.15292	0.9957776	0.000439	kinesin family member 26A
UP in HEB	**64129**	TINAGL1	-7.004822	0.9336854	0.007786	tubulointerstitial nephritis antigen-like 1
UP in HEB	**8924**	HERC2	-1.028914	0.8787758	0.490079	HECT and RLD domain containing E3 ubiquitin protein ligase 2
UP in HEB	**9891**	NUAK1	-1.437852	0.8274308	0.369116	NUAK family, SNF1-like kinase, 1
UP in HEB	**65992**	DDRGK1	-1.030111	0.8768379	0.489673	DDRGK domain containing 1
UP in HEB	**284695**	ZNF326	-2.12113	0.9354721	0.229867	zinc finger protein 326
UP in HEB	**348**	APOE	-2.712407	0.8932743	0.152575	apolipoprotein E
UP in HEB	**993**	CDC25A	-1.651425	0.9152577	0.318326	cell division cycle 25A
UP in HEB	**113179**	ADAT3	-1.436099	0.8601525	0.369565	adenosine deaminase, tRNA-specific 3
UP in HEB	**10848**	PPP1R13L	-2.093659	0.9253916	0.234286	protein phosphatase 1, regulatory subunit 13
UP in HEB	**8741**	TNFSF13	-2.277893	0.9028482	0.206199	tumor necrosis factor (ligand) superfamily, member 13
UP in HEB	**80161**	ASMTL-AS1	-2.330666	0.9259961	0.198792	ASMTL antisense RNA 1
UP in HEB	**64284**	RAB17	-4.247928	0.8085676	0.052632	RAB17, member RAS oncogene family
UP in HEB	**23304**	UBR2	-1.44539	0.8835316	0.367193	ubiquitin protein ligase E3 component n-recognin 2
UP in HEB	**5318**	PKP2	-7	0.8988213	0.007813	plakophilin 2
UP in HEB	**10409**	BASP1	-1.92447	0.9292584	0.263437	brain abundant, membrane attached signal protein 1
UP in HEB	**22981**	NINL	-5.182692	0.9292318	0.027533	ninein-like
UP in HEB	**100996295**	LOC100996295	-5.098422	0.9301563	0.029189	uncharacterized LOC100996295
UP in HEB	**3398**	ID2	-2.129465	0.9370811	0.228543	inhibitor of DNA binding 2, dominant negative helix-loop-helix protein
UP in HEB	**55319**	TMA16	-1.885745	0.922627	0.270604	translation machinery associated 16 homolog (S. cerevisiae)
UP in HEB	**84450**	ZNF512	-1.603355	0.9091952	0.329111	zinc finger protein 512
UP in HEB	**3399**	ID3	-3.31513	0.9758654	0.100472	inhibitor of DNA binding 3, dominant negative helix-loop-helix protein
UP in HEB	**3169**	FOXA1	-7.360481	0.8615126	0.006085	forkhead box A1
UP in HEB	**8544**	PIR	-2.14267	0.9365033	0.22646	pirin (iron-binding nuclear protein)
UP in HEB	**9898**	UBAP2L	-1.178413	0.8956478	0.441837	ubiquitin associated protein 2-like
UP in HEB	**7099**	TLR4	-3.088101	0.9136843	0.117595	toll-like receptor 4
UP in HEB	**2035**	EPB41	-1.330091	0.8918342	0.397743	erythrocyte membrane protein band 4.1 (elliptocytosis 1, RH-linked)
UP in HEB	**5187**	PER1	-3.445746	0.9751187	0.091776	period circadian clock 1
UP in HEB	**84962**	AJUBA	-1.053692	0.8670951	0.481734	ajuba LIM protein
UP in HEB	**221749**	PXDC1	-1.365615	0.8960211	0.388069	PX domain containing 1
UP in HEB	**113263**	GLCCI1	-1.256466	0.8174747	0.418568	glucocorticoid induced transcript 1
UP in HEB	**387496**	RASL11A	-2.040688	0.9043505	0.243048	RAS-like, family 11, member A
UP in HEB	**3978**	LIG1	-1.594827	0.9174445	0.331062	ligase I, DNA, ATP-dependent
UP in HEB	**1111**	CHEK1	-1.410015	0.8964478	0.376308	checkpoint kinase 1
UP in HEB	**255027**	MPV17L	-4.343216	0.9033015	0.049268	MPV17 mitochondrial membrane protein-
UP in HEB	**50487**	PLA2G3	-6.830779	0.9059239	0.008785	phospholipase A2, group III
UP in HEB	**54997**	TESC	-8.350203	0.9419969	0.003064	tescalcin

UP in HEB	**65109**	UPF3B	-1.196612	0.8894697	0.436299	UPF3 regulator of nonsense transcripts homolog B (yeast)
UP in HEB	**56940**	DUSP22	-1.082347	0.8141768	0.47226	dual specificity phosphatase 22
UP in HEB	**725**	C4BPB	-7.080373	0.9704518	0.007389	complement component 4 binding protein,
UP in HEB	**100128191**	TMPO-	-1.888579	0.9105552	0.270073	TMPO antisense RNA 1
UP in HEB	**4048**	LTA4H	-1.703564	0.9233648	0.307027	leukotriene A4 hydrolase
UP in HEB	**26147**	PHF19	-2.457242	0.951713	0.182094	PHD finger protein 19
UP in HEB	**23567**	ZNF346	-1.322661	0.8785713	0.399797	zinc finger protein 346
UP in HEB	**2637**	GBX2	-7.169925	0.8397603	0.006944	gastrulation brain homeobox 2
UP in HEB	**6509**	SLC1A4	-1.004792	0.836169	0.498342	solute carrier family 1 (glutamate/neutral amino acid transporter), member 4
UP in HEB	**10518**	CIB2	-3.980112	0.9552954	0.063368	calcium and integrin binding family member
UP in HEB	**359845**	FAM101B	-4.754287	0.9764699	0.037052	family with sequence similarity 101, member
UP in HEB	**23269**	MGA	-2.055563	0.9321919	0.240555	MGA, MAX dimerization protein
UP in HEB	**8365**	HIST1H4H	-2.072106	0.8423294	0.237812	histone cluster 1, H4h
UP in HEB	**6293**	VPS52	-1.088181	0.8820559	0.470354	vacuolar protein sorting 52 homolog (S. cerevisiae)
UP in HEB	**170384**	FUT11	-1.286528	0.8874696	0.409937	fucosyltransferase 11 (alpha (1,3) fucosyltransferase)
UP in HEB	**3225**	HOXC9	-3.982272	0.946246	0.063273	homeobox C9
UP in HEB	**9212**	AURKB	-1.318688	0.901657	0.400899	aurora kinase B
UP in HEB	**440905**	LOC44090	-7.748193	0.900448	0.004651	uncharacterized LOC440905
UP in HEB	**122622**	ADSSL1	-2.78728	0.9524241	0.144859	adenylosuccinate synthase like 1
UP in HEB	**1281**	COL3A1	-11.89448	0.9997333	0.000263	collagen, type III, alpha 1
UP in HEB	**100532726**	NDUFC2-KCTD14	-6.41391	0.9894128	0.011728	NDUFC2-KCTD14 readthrough
UP in HEB	**6307**	MSMO1	-1.002385	0.8740733	0.499174	methylsterol monooxygenase 1
UP in HEB	**5422**	POLA1	-2.154431	0.9363522	0.224622	polymerase (DNA directed), alpha 1, catalytic subunit
UP in HEB	**222658**	KCTD20	-1.379311	0.8977279	0.384402	potassium channel tetramerisation domain containing 20
UP in HEB	**1942**	EFNA1	-9.279343	0.9964087	0.001609	ephrin-A1
UP in HEB	**414149**	ACBD7	-2.218047	0.8902964	0.214932	acyl-CoA binding domain containing 7
UP in HEB	**25836**	NIPBL	-1.085058	0.8784913	0.471373	Nipped-B homolog (Drosophila)
UP in HEB	**51377**	UCHL5	-1.642984	0.9195779	0.320193	ubiquitin carboxyl-terminal hydrolase L5
UP in HEB	**5711**	PSMD5	-1.014625	0.8812381	0.494957	proteasome (prosome, macropain) 26S subunit, non-ATPase, 5
UP in HEB	**100287177**	LOC100287177	-4.410129	0.9558643	0.047035	uncharacterized LOC100287177
UP in HEB	**57153**	SLC44A2	-1.342236	0.8996302	0.394409	solute carrier family 44, member 2
UP in HEB	**10276**	NET1	-1.440443	0.9006791	0.368454	neuroepithelial cell transforming 1
UP in HEB	**221150**	SKA3	-1.19426	0.8678685	0.437011	spindle and kinetochore associated complex subunit 3
UP in HEB	**3182**	HNRNPA	-1.443636	0.9065995	0.367639	heterogeneous nuclear ribonucleoprotein A/B
UP in HEB	**124976**	SPNS2	-1.321666	0.8757	0.400073	spinster homolog 2 (Drosophila)
UP in HEB	**494470**	RNF165	-7.251088	0.8490231	0.006565	ring finger protein 165
UP in HEB	**128272**	ARHGEF1	-8.802839	0.9818301	0.002239	Rho guanine nucleotide exchange factor
UP in HEB	**9618**	TRAF4	-1.778136	0.9226625	0.29156	TNF receptor-associated factor 4
UP in HEB	**89890**	KBTBD6	-1.247568	0.8668196	0.421158	kelch repeat and BTB (POZ) domain
UP in HEB	**223**	ALDH9A1	-1.298047	0.8970167	0.406676	aldehyde dehydrogenase 9 family, member
UP in HEB	**1852**	DUSP9	-4.463797	0.8906964	0.045317	dual specificity phosphatase 9
UP in HEB	**6130**	RPL7A	-1.416911	0.906395	0.374513	ribosomal protein L7a
UP in HEB	**79915**	ATAD5	-1.571906	0.8689797	0.336364	ATPase family, AAA domain containing 5
UP in HEB	**51287**	COA4	-1.399882	0.9040215	0.37896	cytochrome c oxidase assembly factor 4 homolog (S. cerevisiae)

UP in HEB	**6877**	TAF5	-1.095742	0.8276086	0.467895	TAF5 RNA polymerase II, TATA box binding protein (TBP)-associated factor, 100kDa
UP in HEB	**79680**	C22orf29	-1.35885	0.8766156	0.389893	chromosome 22 open reading frame 29
UP in HEB	**221545**	C6orf136	-1.359458	0.8918876	0.389729	chromosome 6 open reading frame 136
UP in HEB	**4678**	NASP	-1.209444	0.8965189	0.432435	nuclear autoantigenic sperm protein (histone-binding)
UP in HEB	**3304**	HSPA1B	-1.611639	0.9232937	0.327226	heat shock 70kDa protein 1B
UP in HEB	**27309**	ZNF330	-1.733815	0.9209736	0.300656	zinc finger protein 330
UP in HEB	**84078**	KBTBD7	-1.576844	0.8131811	0.335214	kelch repeat and BTB (POZ) domain
UP in HEB	**10481**	HOXB13	-7.773469	0.9498996	0.00457	homeobox B13
UP in HEB	**10882**	C1QL1	-2.012452	0.9221381	0.247851	complement component 1, q subcomponent-
UP in HEB	**4361**	MRE11A	-1.250099	0.8771134	0.420419	MRE11 meiotic recombination 11 homolog A (S. cerevisiae)
UP in HEB	**85445**	CNTNAP4	-7.132714	0.835049	0.007126	contactin associated protein-like 4
UP in HEB	**23102**	TBC1D2B	-1.181032	0.8788913	0.441036	TBC1 domain family, member 2B
UP in HEB	**389136**	VGLL3	-1.655337	0.914031	0.317464	vestigial like 3 (Drosophila)
UP in HEB	**83743**	GRWD1	-1.44352	0.8999769	0.367669	glutamate-rich WD repeat containing 1
UP in HEB	**1810**	DR1	-1.23247	0.8943321	0.425588	down-regulator of transcription 1, TBP-binding (negative cofactor 2)
UP in HEB	**50628**	GEMIN4	-1.40992	0.9003947	0.376333	gem (nuclear organelle) associated protein 4
UP in HEB	**100499177**	THAP9-	-1.476539	0.8946877	0.35935	THAP9 antisense RNA 1
UP in HEB	**2976**	GTF3C2	-1.047609	0.884945	0.483769	general transcription factor IIIC, polypeptide 2, beta 110kDa
UP in HEB	**9440**	MED17	-1.474676	0.8934521	0.359814	mediator complex subunit 17
UP in HEB	**4258**	MGST2	-11.90426	0.9977777	0.000261	microsomal glutathione S-transferase 2
UP in HEB	**79956**	ERMP1	-1.288105	0.8832738	0.409488	endoplasmic reticulum metallopeptidase 1
UP in HEB	**23157**	6-Sep	-7.716207	0.9944619	0.004755	septin 6
UP in HEB	**387097**	C6orf147	-5.918068	0.9318097	0.016538	chromosome 6 open reading frame 147
UP in HEB	**84836**	ABHD14B	-1.552541	0.9076306	0.340909	abhydrolase domain containing 14B
UP in HEB	**10866**	HCP5	-7.718031	0.9947286	0.004749	HLA complex P5 (non-protein coding)
UP in HEB	**84920**	ALG10	-1.294634	0.8485341	0.40764	ALG10, alpha-1,2-glucosyltransferase
UP in HEB	**4552**	MTRR	-1.56422	0.9067595	0.338161	5-methyltetrahydrofolate-homocysteine methyltransferase reductase
UP in HEB	**100505696**	SH3BP5-AS1	-1.988854	0.9274361	0.251939	SH3BP5 antisense RNA 1
UP in HEB	**64432**	MRPS25	-1.850869	0.9254538	0.277225	mitochondrial ribosomal protein S25
UP in HEB	**55224**	ETNK2	-7.285402	0.9375878	0.00641	ethanolamine kinase 2
UP in HEB	**148423**	C1orf52	-1.271353	0.8544722	0.414271	chromosome 1 open reading frame 52
UP in HEB	**708**	C1QBP	-1.324559	0.9032748	0.399271	complement component 1, q subcomponent binding protein
UP in HEB	**219621**	C10orf107	-4.122096	0.9069906	0.057428	chromosome 10 open reading frame 107
UP in HEB	**55020**	TTC38	-1.989698	0.9232581	0.251792	tetratricopeptide repeat domain 38
UP in HEB	**100463482**	MTRNR2L	-2.829292	0.9652248	0.140701	MT-RNR2-like 6
UP in HEB	**124739**	USP43	-8.067882	0.9244137	0.003727	ubiquitin specific peptidase 43
UP in HEB	**6181**	RPLP2	-1.260835	0.9002525	0.417302	ribosomal protein, large, P2
UP in HEB	**91614**	DEPDC7	-2.323719	0.93733	0.199752	DEP domain containing 7
UP in HEB	**27129**	HSPB7	-5.871187	0.9825857	0.017084	heat shock 27kDa protein family, member 7 (cardiovascular)
UP in HEB	**142**	PARP1	-1.374982	0.9036304	0.385558	poly (ADP-ribose) polymerase 1
UP in HEB	**10516**	FBLN5	-2.551267	0.9406902	0.170605	fibulin 5
UP in HEB	**27124**	INPP5J	-4.265293	0.9692873	0.052002	inositol polyphosphate-5-phosphatase J
UP in HEB	**22943**	DKK1	-2.93479	0.9582556	0.13078	dickkopf 1 homolog (Xenopus laevis)
UP in HEB	**2037**	EPB41L2	-1.169486	0.894261	0.44458	erythrocyte membrane protein band 4.1-like
UP in HEB	**2295**	FOXF2	-11.44553	0.996622	0.000359	forkhead box F2
UP in HEB	**6480**	ST6GAL1	-7.73245	0.8993102	0.004702	ST6 beta-galactosamide alpha-2,6-sialyltranferase 1

UP in HEB	**5018**	OXA1L	-1.22743	0.8973723	0.427077	oxidase (cytochrome c) assembly 1-like
UP in HEB	**4609**	MYC	-3.474353	0.9784877	0.089974	v-myc myelocytomatosis viral oncogene homolog (avian)
UP in HEB	**7272**	TTK	-1.268833	0.8913898	0.414995	TTK protein kinase
UP in HEB	**9374**	PPT2	-2.950261	0.9617846	0.129385	palmitoyl-protein thioesterase 2
UP in HEB	**26973**	CHORDC1	-1.25121	0.8932388	0.420096	cysteine and histidine-rich domain (CHORD) containing 1
UP in HEB	**84522**	JAGN1	-1.514124	0.9066972	0.350109	jagunal homolog 1 (Drosophila)
UP in HEB	**11177**	BAZ1A	-1.257238	0.8854606	0.418344	bromodomain adjacent to zinc finger domain,
UP in HEB	**60674**	GAS5	-1.322181	0.9032126	0.39993	growth arrest-specific 5 (non-protein coding)
UP in HEB	**3658**	IREB2	-1.709055	0.9220403	0.30586	iron-responsive element binding protein 2
UP in HEB	**8936**	WASF1	-1.387613	0.8974968	0.382197	WAS protein family, member 1
UP in HEB	**64288**	ZSCAN31	-6.364254	0.9909062	0.012139	zinc finger and SCAN domain containing 31
UP in HEB	**83872**	HMCN1	-3.860904	0.836169	0.068826	hemicentin 1
UP in HEB	**25840**	METTL7A	-5.637611	0.9955553	0.020087	methyltransferase like 7A
UP in HEB	**3704**	ITPA	-1.48434	0.9042527	0.357412	inosine triphosphatase (nucleoside triphosphate pyrophosphatase)
UP in HEB	**83879**	CDCA7	-3.214036	0.9698651	0.107765	cell division cycle associated 7
UP in HEB	**51388**	NIP7	-1.503621	0.9060572	0.352667	NIP7, nucleolar pre-rRNA processing protein
UP in HEB	**81794**	ADAMTS 10	-7.098032	0.8774068	0.007299	ADAM metallopeptidase with thrombospondin type 1 motif, 10
UP in HEB	**60493**	FASTKD5	-1.36257	0.8846161	0.388889	FAST kinase domains 5
UP in HEB	**90333**	ZNF468	-5.779172	0.8989102	0.018209	zinc finger protein 468
UP in HEB	**2**	A2M	-12.41521	0.9987733	0.000183	alpha-2-macroglobulin
UP in HEB	**2125**	EVPL	-9.345775	0.9783366	0.001537	envoplakin
UP in HEB	**6624**	FSCN1	-2.190139	0.9423436	0.21913	fascin homolog 1, actin-bundling protein (Strongylocentrotus purpuratus)
UP in HEB	**10581**	IFITM2	-4.669048	0.992853	0.039308	interferon induced transmembrane protein 2
UP in HEB	**60312**	AFAP1	-1.941808	0.9254627	0.26029	actin filament associated protein 1
UP in HEB	**84987**	COX14	-1.100996	0.8896652	0.466195	cytochrome c oxidase assembly homolog 14 (S. cerevisiae)
UP in HEB	**84842**	HPDL	-10.44624	0.9986044	0.000717	4-hydroxyphenylpyruvate dioxygenase-like
UP in HEB	**56931**	DUS3L	-1.299093	0.8940744	0.406382	dihydrouridine synthase 3-like (S. cerevisiae)
UP in HEB	**55157**	DARS2	-1.585791	0.9164311	0.333142	aspartyl-tRNA synthetase 2, mitochondrial
UP in HEB	**4798**	NFRKB	-1.746068	0.9171422	0.298113	nuclear factor related to kappaB binding
UP in HEB	**4883**	NPR3	-8.558553	0.9974399	0.002652	natriuretic peptide receptor C/guanylate cyclase C (atrionatriuretic peptide receptor C)
UP in HEB	**130340**	AP1S3	-4.379785	0.9873682	0.048035	adaptor-related protein complex 1, sigma 3 subunit
UP in HEB	**1998**	ELF2	-2.259367	0.9334898	0.208864	E74-like factor 2 (ets domain transcription
UP in HEB	**57585**	CRAMP1L	-1.241454	0.8561168	0.422946	Crm, cramped-like (Drosophila)
UP in HEB	**23186**	RCOR1	-1.256573	0.8909364	0.418537	REST corepressor 1
UP in HEB	**4776**	NFATC4	-3.411777	0.9609935	0.093962	nuclear factor of activated T-cells, cytoplasmic, calcineurin-dependent 4
UP in HEB	**25791**	NGEF	-3.211948	0.945926	0.107921	neuronal guanine nucleotide exchange factor
UP in HEB	**51008**	ASCC1	-1.684038	0.9193379	0.31121	activating signal cointegrator 1 complex
UP in HEB	**10053**	AP1M2	-10.73386	0.9940619	0.000587	adaptor-related protein complex 1, mu 2
UP in HEB	**5818**	PVRL1	-3.334556	0.9039771	0.099129	poliovirus receptor-related 1 (herpesvirus entry mediator C)
UP in HEB	**9972**	NUP153	-1.979107	0.9271161	0.253647	nucleoporin 153kDa
UP in HEB	**31**	ACACA	-1.560482	0.9044127	0.339038	acetyl-CoA carboxylase alpha
UP in HEB	**4717**	NDUFC1	-1.046804	0.8866428	0.484039	NADH dehydrogenase (ubiquinone) 1, subcomplex unknown, 1, 6kDa
UP in HEB	**3336**	HSPE1	-1.126298	0.8935054	0.45809	heat shock 10kDa protein 1 (chaperonin 10)
UP in HEB	**8991**	SELENBP	-12.40842	0.9987644	0.000184	selenium binding protein 1
UP in HEB	**92745**	SLC38A5	-3.505528	0.8938432	0.08805	solute carrier family 38, member 5

UP in HEB	**10112**	KIF20A	-2.223506	0.9420236	0.21412	kinesin family member 20A
UP in HEB	**9807**	IP6K1	-1.235116	0.8941721	0.424808	inositol hexakisphosphate kinase 1
UP in HEB	**1460**	CSNK2B	-1.288141	0.9008214	0.409478	casein kinase 2, beta polypeptide
UP in HEB	**6996**	TDG	-1.877148	0.9213291	0.272221	thymine-DNA glycosylase
UP in HEB	**143684**	FAM76B	-1.547559	0.8562501	0.342088	family with sequence similarity 76, member
UP in HEB	**254225**	RNF169	-1.304101	0.8462674	0.404973	ring finger protein 169
UP in HEB	**58191**	CXCL16	-4.847997	0.8755134	0.034722	chemokine (C-X-C motif) ligand 16
UP in HEB	**10899**	JTB	-1.198813	0.8962078	0.435633	jumping translocation breakpoint
UP in HEB	**5140**	PDE3B	-5.93507	0.9928885	0.016344	phosphodiesterase 3B, cGMP-inhibited
UP in HEB	**23310**	NCAPD3	-1.991598	0.9276317	0.25146	non-SMC condensin II complex, subunit D3
UP in HEB	**114879**	OSBPL5	-1.954606	0.9234804	0.257991	oxysterol binding protein-like 5
UP in HEB	**3909**	LAMA3	-3.185405	0.9018881	0.109925	laminin, alpha 3
UP in HEB	**7112**	TMPO	-2.322681	0.9513752	0.199896	thymopoietin
UP in HEB	**79191**	IRX3	-2.520336	0.9507529	0.174302	iroquois homeobox 3
UP in HEB	**55038**	CDCA4	-1.290007	0.8961545	0.408949	cell division cycle associated 4
UP in HEB	**4133**	MAP2	-1.634464	0.9035415	0.32209	microtubule-associated protein 2
UP in HEB	**1154**	CISH	-2.429508	0.831031	0.185629	cytokine inducible SH2-containing protein
UP in HEB	**100329109**	GCSHP3	-1.357801	0.855779	0.390177	glycine cleavage system protein H (aminomethyl carrier) pseudogene 3
UP in HEB	**116832**	RPL39L	-2.30608	0.9413213	0.202209	ribosomal protein L39-like
UP in HEB	**1122**	CHML	-2.034036	0.9366455	0.244171	choroideremia-like (Rab escort protein 2)
UP in HEB	**151313**	FAHD2B	-3.679099	0.9730208	0.078069	fumarylacetoacetate hydrolase domain containing 2B
UP in HEB	**80169**	CTC1	-2.006406	0.9175689	0.248892	CTS telomere maintenance complex
UP in HEB	**221656**	KDM1B	-1.095029	0.8559834	0.468127	lysine (K)-specific demethylase 1B
UP in HEB	**51069**	MRPL2	-1.14707	0.8897986	0.451541	mitochondrial ribosomal protein L2
UP in HEB	**6210**	RPS15A	-1.328949	0.903506	0.398058	ribosomal protein S15a
UP in HEB	**64225**	ATL2	-1.455187	0.9039149	0.364708	atlastin GTPase 2
UP in HEB	**4199**	ME1	-9.198854	0.9942486	0.001702	malic enzyme 1, NADP(+)-dependent,
UP in HEB	**2288**	FKBP4	-1.041244	0.8875496	0.485908	FK506 binding protein 4, 59kDa
UP in HEB	**23067**	SETD1B	-1.414512	0.8882252	0.375137	SET domain containing 1B
UP in HEB	**160428**	ALDH1L2	-1.42131	0.8794958	0.373373	aldehyde dehydrogenase 1 family, member
UP in HEB	**842**	CASP9	-1.489633	0.8912209	0.356103	caspase 9, apoptosis-related cysteine
UP in HEB	**1737**	DLAT	-1.817039	0.9226003	0.283803	dihydrolipoamide S-acetyltransferase
UP in HEB	**11243**	PMF1	-1.334819	0.8986079	0.396442	polyamine-modulated factor 1
UP in HEB	**280**	AMY2B	-2.830328	0.9156577	0.1406	amylase, alpha 2B (pancreatic)
UP in HEB	**100462981**	MTRNR2L	-2.009676	0.9406013	0.248329	MT-RNR2-like 2
UP in HEB	**221527**	ZBTB12	-1.612265	0.8871762	0.327084	zinc finger and BTB domain containing 12
UP in HEB	**3488**	IGFBP5	-6.866249	0.8024695	0.008571	insulin-like growth factor binding protein 5
UP in HEB	**6949**	TCOF1	-1.377578	0.9035237	0.384864	Treacher Collins-Franceschetti syndrome 1
UP in HEB	**65983**	GRAMD3	-4.125531	0.9023859	0.057292	GRAM domain containing 3
UP in HEB	**83742**	MARVEL	-1.711637	0.9219158	0.305313	MARVEL domain containing 1
UP in HEB	**23015**	GOLGA8A	-3.739295	0.9801234	0.074879	golgin A8 family, member A
UP in HEB	**3014**	H2AFX	-1.695386	0.923427	0.308772	H2A histone family, member X
UP in HEB	**160518**	DENND5B	-2.153164	0.9362099	0.224819	DENN/MADD domain containing 5B
UP in HEB	**5118**	PCOLCE	-2.605388	0.9597045	0.164324	procollagen C-endopeptidase enhancer
UP in HEB	**641**	BLM	-1.991813	0.9175689	0.251423	Bloom syndrome, RecQ helicase-like
UP in HEB	**100532746**	PPT2-	-2.579045	0.9255071	0.167352	PPT2-EGFL8 readthrough
UP in HEB	**6817**	SULT1A1	-5.344336	0.9926129	0.024615	sulfotransferase family, cytosolic, 1A, phenol-preferring, member 1
UP in HEB	**56953**	NT5M	-1.432696	0.8160435	0.370438	5',3'-nucleotidase, mitochondrial
UP in HEB	**6129**	RPL7	-1.084809	0.8913453	0.471455	ribosomal protein L7
UP in HEB	**128061**	C1orf131	-1.248901	0.8737977	0.420769	chromosome 1 open reading frame 131
UP in HEB	**57465**	TBC1D24	-1.224373	0.8413871	0.427984	TBC1 domain family, member 24
UP in HEB	**64328**	XPO4	-1.077137	0.840756	0.473969	exportin 4
UP in HEB	**7919**	DDX39B	-2.301741	0.9432503	0.202818	DEAD (Asp-Glu-Ala-Asp) box polypeptide
UP in HEB	**388969**	C2orf68	-1.403526	0.8747044	0.378004	chromosome 2 open reading frame 68

UP in HEB	**79047**	KCTD15	-9.098909	0.9720163	0.001824	potassium channel tetramerisation domain containing 15
UP in HEB	**2805**	GOT1	-1.311319	0.9002969	0.402952	glutamic-oxaloacetic transaminase 1, soluble (aspartate aminotransferase 1)
UP in HEB	**3776**	KCNK2	-5.760221	0.871682	0.01845	potassium channel, subfamily K, member 2
UP in HEB	**26097**	CHTOP	-1.123605	0.8898341	0.458946	chromatin target of PRMT1
UP in HEB	**90102**	PHLDB2	-2.482393	0.9438014	0.178947	pleckstrin homology-like domain, family B, member 2
UP in HEB	**1583**	CYP11A1	-5.211888	0.8823937	0.026981	cytochrome P450, family 11, subfamily A, polypeptide 1
UP in HEB	**54937**	SOHLH2	-9.83289	0.9965598	0.001096	spermatogenesis and oogenesis specific basic helix-loop-helix 2
UP in HEB	**100124700**	HOTAIR	-5.350939	0.89555	0.024502	HOX transcript antisense RNA
UP in HEB	**22858**	ICK	-1.322613	0.8030562	0.39981	intestinal cell (MAK-like) kinase
UP in HEB	**1917**	EEF1A2	-9.822504	0.99928	0.001104	eukaryotic translation elongation factor 1
UP in HEB	**80155**	NAA15	-1.13839	0.8870962	0.454266	N(alpha)-acetyltransferase 15, NatA auxiliary subunit
UP in HEB	**51710**	ZNF44	-3.670936	0.8287375	0.078512	zinc finger protein 44
UP in HEB	**3892**	KRT86	-5.938599	0.9334542	0.016304	keratin 86
UP in HEB	**578**	BAK1	-1.258656	0.8886607	0.417933	BCL2-antagonist/killer 1
UP in HEB	**55644**	OSGEP	-1.471219	0.8916742	0.360677	O-sialoglycoprotein endopeptidase
UP in HEB	**9144**	SYNGR2	-1.774573	0.9245471	0.292281	synaptogyrin 2
UP in HEB	**10146**	G3BP1	-1.079872	0.8905275	0.473071	GTPase activating protein (SH3 domain) binding protein 1
UP in HEB	**222234**	FAM185A	-1.424393	0.8427827	0.372576	family with sequence similarity 185, member
UP in HEB	**80031**	SEMA6D	-1.098217	0.8727221	0.467093	sema domain, transmembrane domain (TM), and cytoplasmic domain, (semaphorin) 6D
UP in HEB	**140700**	SAMD10	-1.541963	0.8350845	0.343418	sterile alpha motif domain containing 10
UP in HEB	**9585**	KIF20B	-1.066068	0.8802958	0.477619	kinesin family member 20B
UP in HEB	**8669**	EIF3J	-1.413532	0.9037015	0.375391	eukaryotic translation initiation factor 3,
UP in HEB	**57010**	CABP4	-3.648738	0.8885807	0.07973	calcium binding protein 4
UP in HEB	**124930**	ANKRD13	-2.929217	0.9599712	0.131286	ankyrin repeat domain 13B
UP in HEB	**339123**	JMJD8	-1.323509	0.8997369	0.399562	jumonji domain containing 8
UP in HEB	**9394**	HS6ST1	-1.374428	0.8841716	0.385706	heparan sulfate 6-O-sulfotransferase 1
UP in HEB	**54790**	TET2	-1.600529	0.8322755	0.329756	tet methylcytosine dioxygenase 2
UP in HEB	**642475**	MROH6	-1.984504	0.8400448	0.2527	maestro heat-like repeat family member 6
UP in HEB	**54946**	SLC41A3	-1.60321	0.9204135	0.329144	solute carrier family 41, member 3
UP in HEB	**7625**	ZNF74	-2.294183	0.8604281	0.203883	zinc finger protein 74
UP in HEB	**10198**	MPHOSP	-1.326023	0.8894786	0.398866	M-phase phosphoprotein 9
UP in HEB	**60598**	KCNK15	-8.874469	0.9651004	0.002131	potassium channel, subfamily K, member 15
UP in HEB	**1288**	COL4A6	-3.140986	0.9596867	0.113362	collagen, type IV, alpha 6
UP in HEB	**5538**	PPT1	-1.165748	0.8946344	0.445733	palmitoyl-protein thioesterase 1
UP in HEB	**131118**	DNAJC19	-1.072635	0.8835493	0.47545	DnaJ (Hsp40) homolog, subfamily C,
UP in HEB	**283377**	SPRYD4	-1.449404	0.8942699	0.366173	SPRY domain containing 4
UP in HEB	**55857**	PLK1S1	-1.115317	0.8192526	0.46159	polo-like kinase 1 substrate 1
UP in HEB	**404550**	C16orf74	-4.142958	0.9061728	0.056604	chromosome 16 open reading frame 74
UP in HEB	**2644**	GCHFR	-4.861757	0.9924885	0.034393	GTP cyclohydrolase I feedback regulator
UP in HEB	**3280**	HES1	-4.056969	0.8984835	0.06008	hairy and enhancer of split 1, (Drosophila)
UP in HEB	**100129794**	LOC100129794	-6.853829	0.8007805	0.008646	uncharacterized LOC100129794
UP in HEB	**8673**	VAMP8	-5.668393	0.9951731	0.019663	vesicle-associated membrane protein 8
UP in HEB	**1836**	SLC26A2	-2.365854	0.9497395	0.194002	solute carrier family 26 (sulfate transporter), member 2
UP in HEB	**10988**	METAP2	-1.716514	0.9231692	0.304283	methionyl aminopeptidase 2
UP in HEB	**5447**	POR	-1.0055	0.8845716	0.498098	P450 (cytochrome) oxidoreductase
UP in HEB	**84250**	ANKRD32	-2.499002	0.9485484	0.176899	ankyrin repeat domain 32
UP in HEB	**56172**	ANKH	-2.264475	0.93789	0.208125	ankylosis, progressive homolog (mouse)

UP in HEB	**100132341**	CLUHP3	-8.449836	0.9473928	0.00286	clustered mitochondria (cluA/CLU1) homolog pseudogene 3
UP in HEB	**249**	ALPL	-10.14296	0.9982844	0.000884	alkaline phosphatase, liver/bone/kidney
UP in HEB	**200172**	SLFNL1	-3.393664	0.8373424	0.095149	schlafen-like 1
UP in HEB	**858**	CAV2	-1.613355	0.9170978	0.326837	caveolin 2
UP in HEB	**84168**	ANTXR1	-1.498751	0.9052127	0.35386	anthrax toxin receptor 1
UP in HEB	**6818**	SULT1A3	-3.408392	0.9586822	0.094183	sulfotransferase family, cytosolic, 1A, phenol-preferring, member 3
UP in HEB	**9534**	ZNF254	-3.70044	0.8565435	0.076923	zinc finger protein 254
UP in HEB	**440926**	H3F3AP4	-1.602907	0.9231426	0.329213	H3 histone, family 3A, pseudogene 4
UP in HEB	**146434**	ZNF597	-5.822002	0.821626	0.017677	zinc finger protein 597
UP in HEB	**54509**	RHOF	-2.460652	0.9499351	0.181664	ras homolog family member F (in filopodia)
UP in HEB	**1756**	DMD	-3.911386	0.9749231	0.066459	dystrophin
UP in HEB	**441518**	FAM127C	-1.195684	0.8899941	0.436579	family with sequence similarity 127, member
UP in HEB	**2564**	GABRE	-10.76708	0.994213	0.000574	gamma-aminobutyric acid (GABA) A receptor, epsilon
UP in HEB	**166614**	DCLK2	-1.468879	0.8558768	0.361263	doublecortin-like kinase 2
UP in HEB	**7078**	TIMP3	-9.49865	0.9905862	0.001382	TIMP metallopeptidase inhibitor 3
UP in HEB	**57017**	COQ9	-1.450935	0.9048571	0.365784	coenzyme Q9 homolog (S. cerevisiae)
UP in HEB	**64073**	C19orf33	-12.44915	0.9988444	0.000179	chromosome 19 open reading frame 33
UP in HEB	**84327**	ZBED3	-2.432529	0.9341476	0.18524	zinc finger, BED-type containing 3
UP in HEB	**2878**	GPX3	-2.50587	0.935321	0.176059	glutathione peroxidase 3 (plasma)
UP in HEB	**51122**	COMMD2	-1.067563	0.8737088	0.477124	COMM domain containing 2
UP in HEB	**10522**	DEAF1	-2.456813	0.9503262	0.182148	DEAF1 transcription factor
UP in HEB	**57212**	TP73-AS1	-2.689987	0.9330631	0.154965	TP73 antisense RNA 1
UP in HEB	**5757**	PTMA	-2.211551	0.9426014	0.215902	prothymosin, alpha
UP in HEB	**51496**	CTDSPL2	-1.413682	0.8940032	0.375352	CTD (carboxy-terminal domain, RNA polymerase II, polypeptide A) small phosphatase like 2
UP in HEB	**26272**	FBXO4	-1.306726	0.8885096	0.404237	F-box protein 4
UP in HEB	**6432**	SRSF7	-1.279575	0.8995146	0.411917	serine/arginine-rich splicing factor 7
UP in HEB	**54874**	FNBP1L	-2.045446	0.9301029	0.242248	formin binding protein 1-like
UP in HEB	**5087**	PBX1	-5.66028	0.9925507	0.019774	pre-B-cell leukemia homeobox 1
UP in HEB	**116039**	OSR2	-2.204058	0.8455918	0.217026	odd-skipped related 2 (Drosophila)
UP in HEB	**65055**	REEP1	-4.459432	0.9158711	0.045455	receptor accessory protein 1
UP in HEB	**8503**	PIK3R3	-2.357042	0.9346187	0.195191	phosphoinositide-3-kinase, regulatory subunit 3 (gamma)
UP in HEB	**7915**	ALDH5A1	-5.261751	0.9648603	0.026065	aldehyde dehydrogenase 5 family, member
UP in HEB	**152687**	ZNF595	-4.412249	0.9665582	0.046966	zinc finger protein 595
UP in HEB	**23555**	TSPAN15	-5.397216	0.9281651	0.023729	tetraspanin 15
UP in HEB	**152503**	SH3D19	-1.860867	0.9228403	0.275311	SH3 domain containing 19
UP in HEB	**257218**	SHPRH	-1.025336	0.8122655	0.491296	SNF2 histone linker PHD RING helicase, E3 ubiquitin protein ligase
UP in HEB	**90550**	MCU	-1.375947	0.9002347	0.3853	mitochondrial calcium uniporter
UP in HEB	**3993**	LLGL2	-7.730511	0.9922218	0.004709	lethal giant larvae homolog 2 (Drosophila)
UP in HEB	**38**	ACAT1	-1.77748	0.9251249	0.291692	acetyl-CoA acetyltransferase 1
UP in HEB	**9682**	KDM4A	-1.100508	0.8818604	0.466352	lysine (K)-specific demethylase 4A
UP in HEB	**219736**	STOX1	-7.72792	0.8987502	0.004717	storkhead box 1
UP in HEB	**4129**	MAOB	-8.967707	0.9682561	0.001997	monoamine oxidase B
UP in HEB	**8514**	KCNAB2	-1.035908	0.859228	0.487709	potassium voltage-gated channel, shaker-related subfamily, beta member 2
UP in HEB	**23302**	WSCD1	-7.459432	0.8723576	0.005682	WSC domain containing 1
UP in HEB	**1870**	E2F2	-3.505015	0.9694828	0.088082	E2F transcription factor 2
UP in HEB	**10541**	ANP32B	-1.03413	0.8880918	0.48831	acidic (leucine-rich) nuclear phosphoprotein 32 family, member B
UP in HEB	**4860**	PNP	-1.013243	0.8820026	0.495431	purine nucleoside phosphorylase

UP in HEB	**23**	ABCF1	-2.016255	0.9395434	0.247199	ATP-binding cassette, sub-family F (GCN20), member 1
UP in HEB	**1678**	TIMM8A	-1.02535	0.8798514	0.491291	translocase of inner mitochondrial membrane 8 homolog A (yeast)
UP in HEB	**112869**	CCDC101	-1.1862	0.8828738	0.439459	coiled-coil domain containing 101
UP in HEB	**57484**	RNF150	-7.913887	0.9661404	0.004147	ring finger protein 150
UP in HEB	**23136**	EPB41L3	-8.271463	0.9763721	0.003236	erythrocyte membrane protein band 4.1-like
UP in HEB	**7051**	TGM1	-2.956411	0.8502409	0.128834	transglutaminase 1 (K polypeptide epidermal type I, protein-glutamine -gamma-glutamyltransferase)
UP in HEB	**3671**	ISLR	-4.025535	0.9050794	0.061404	immunoglobulin superfamily containing leucine-rich repeat
UP in HEB	**80274**	SCUBE1	-8.403438	0.9728519	0.002953	signal peptide, CUB domain, EGF-like 1
UP in HEB	**85459**	KIAA1731	-1.143893	0.8006383	0.452537	KIAA1731
UP in HEB	**64118**	DUS1L	-1.116861	0.8908831	0.461096	dihydrouridine synthase 1-like (S. cerevisiae)
UP in HEB	**10128**	LRPPRC	-1.777293	0.926405	0.29173	leucine-rich pentatricopeptide repeat
UP in HEB	**91683**	SYT12	-11.90401	0.9977777	0.000261	synaptotagmin XII
UP in HEB	**2687**	GGT5	-6.012824	0.9726741	0.015487	gamma-glutamyltransferase 5
UP in HEB	**6745**	SSR1	-1.27212	0.8938254	0.414051	signal sequence receptor, alpha
UP in HEB	**5111**	PCNA	-1.248046	0.8993546	0.421018	proliferating cell nuclear antigen
UP in HEB	**25853**	DCAF12	-1.763441	0.9230003	0.294545	DDB1 and CUL4 associated factor 12
UP in HEB	**29094**	LGALSL	-5.295233	0.881887	0.025467	lectin, galactoside-binding-like
UP in HEB	**983**	CDK1	-1.509998	0.9086351	0.351112	cyclin-dependent kinase 1
UP in HEB	**79903**	NAA60	-1.267407	0.8883318	0.415406	N(alpha)-acetyltransferase 60, NatF catalytic subunit
UP in HEB	**4004**	LMO1	-5.142157	0.985377	0.028318	LIM domain only 1 (rhombotin 1)
UP in HEB	**92312**	MEX3A	-2.187739	0.8946077	0.219495	mex-3 homolog A (C. elegans)
UP in HEB	**10228**	STX6	-1.016234	0.8620015	0.494405	syntaxin 6
UP in HEB	**24142**	NAT6	-1.066538	0.8741177	0.477464	N-acetyltransferase 6 (GCN5-related)
UP in HEB	**5832**	ALDH18A	-1.228615	0.8966167	0.426727	aldehyde dehydrogenase 18 family, member
UP in HEB	**2305**	FOXM1	-1.727042	0.9252138	0.302071	forkhead box M1
UP in HEB	**7576**	ZNF28	-4.316146	0.8473696	0.050201	zinc finger protein 28
UP in HEB	**401089**	C3orf72	-6.609179	0.9679538	0.010243	chromosome 3 open reading frame 72
UP in HEB	**513**	ATP5D	-1.047289	0.8890874	0.483877	ATP synthase, H+ transporting, mitochondrial F1 complex, delta subunit
UP in HEB	**5903**	RANBP2	-1.186765	0.892012	0.439287	RAN binding protein 2
UP in HEB	**5106**	PCK2	-1.043927	0.8716465	0.485006	phosphoenolpyruvate carboxykinase 2 (mitochondrial)
UP in HEB	**118980**	SFXN2	-1.849116	0.8898519	0.277562	sideroflexin 2
UP in HEB	**8341**	HIST1H2B	-3.079227	0.8792113	0.118321	histone cluster 1, H2bn
UP in HEB	**100532732**	MSH5-SAPCD1	-1.458883	0.8368535	0.363775	MSH5-SAPCD1 readthrough (non-protein coding)
UP in HEB	**79927**	FAM110D	-7.622052	0.8890696	0.005076	family with sequence similarity 110, member
UP in HEB	**145282**	MIPOL1	-6.934674	0.8105499	0.008174	mirror-image polydactyly 1
UP in HEB	**4194**	MDM4	-1.488267	0.8700197	0.356441	Mdm4 p53 binding protein homolog (mouse)
UP in HEB	**3833**	KIFC1	-1.641567	0.9203335	0.320508	kinesin family member C1
UP in HEB	**79017**	GGCT	-2.529026	0.952673	0.173256	gamma-glutamylcyclotransferase
UP in HEB	**8970**	HIST1H2B	-2.581215	0.944966	0.1671	histone cluster 1, H2bj
UP in HEB	**64745**	METTL17	-1.132663	0.8863317	0.456073	methyltransferase like 17
UP in HEB	**51564**	HDAC7	-1.370375	0.9001991	0.386791	histone deacetylase 7
UP in HEB	**151009**	LOC15100	-6.470714	0.9918129	0.011275	uncharacterized LOC151009
UP in HEB	**84518**	CNFN	-4.775294	0.9400501	0.036517	cornifelin
UP in HEB	**84273**	NOA1	-1.1392	0.8786691	0.454011	nitric oxide associated 1
UP in HEB	**11095**	ADAMTS 8	-8.154818	0.930423	0.003509	ADAM metallopeptidase with thrombospondin type 1 motif, 8
UP in HEB	**11185**	INMT	-2.279376	0.9136398	0.205987	indolethylamine N-methyltransferase
UP in HEB	**4929**	NR4A2	-3.728798	0.9600779	0.075426	nuclear receptor subfamily 4, group A,

UP in HEB	**79671**	NLRX1	-8.47032	0.9483617	0.00282	NLR family member X1
UP in HEB	**871**	SERPINH1	-2.009965	0.9400235	0.248279	serpin peptidase inhibitor, clade H (heat shock protein 47), member 1, (collagen binding protein 1)
UP in HEB	**56981**	PRDM11	-3.102098	0.8550412	0.11646	PR domain containing 11
UP in HEB	**121274**	ZNF641	-1.395595	0.8586414	0.380088	zinc finger protein 641
UP in HEB	**688**	KLF5	-3.050483	0.9657315	0.120702	Kruppel-like factor 5 (intestinal)
UP in HEB	**9221**	NOLC1	-1.538737	0.9092841	0.344187	nucleolar and coiled-body phosphoprotein 1
UP in HEB	**6738**	TROVE2	-1.755206	0.9104308	0.296231	TROVE domain family, member 2
UP in HEB	**29068**	ZBTB44	-2.664711	0.9451971	0.157704	zinc finger and BTB domain containing 44
UP in HEB	**78999**	LRFN4	-2.366472	0.9493751	0.193919	leucine rich repeat and fibronectin type III domain containing 4
UP in HEB	**6422**	SFRP1	-8.787903	0.9957864	0.002262	secreted frizzled-related protein 1
UP in HEB	**140609**	NEK7	-1.367348	0.8959589	0.387603	NIMA-related kinase 7
UP in HEB	**79886**	CAAP1	-2.305124	0.9327786	0.202343	caspase activity and apoptosis inhibitor 1
UP in HEB	**124401**	ANKS3	-1.864407	0.9139954	0.274636	ankyrin repeat and sterile alpha motif domain containing 3
UP in HEB	**3033**	HADH	-1.115859	0.8852028	0.461416	hydroxyacyl-CoA dehydrogenase
UP in HEB	**5268**	SERPINB5	-6.658211	0.9340231	0.009901	serpin peptidase inhibitor, clade B (ovalbumin), member 5
UP in HEB	**3242**	HPD	-8.919405	0.9986221	0.002065	4-hydroxyphenylpyruvate dioxygenase
UP in HEB	**2030**	SLC29A1	-1.669934	0.9226003	0.314268	solute carrier family 29 (nucleoside transporters), member 1
UP in HEB	**84196**	USP48	-1.162955	0.8906519	0.446597	ubiquitin specific peptidase 48
UP in HEB	**127343**	DMBX1	-3.848538	0.9276495	0.069418	diencephalon/mesencephalon homeobox 1
UP in HEB	**9319**	TRIP13	-1.04745	0.887274	0.483823	thyroid hormone receptor interactor 13
UP in HEB	**6559**	SLC12A3	-8.546894	0.9767454	0.002674	solute carrier family 12 (sodium/chloride transporters), member 3
UP in HEB	**135112**	NCOA7	-2.476691	0.945686	0.179656	nuclear receptor coactivator 7
UP in HEB	**26227**	PHGDH	-1.596833	0.9229114	0.330602	phosphoglycerate dehydrogenase
UP in HEB	**83641**	FAM107B	-2.29978	0.9389656	0.203094	family with sequence similarity 107, member
UP in HEB	**259173**	ALS2CL	-5.182203	0.8473163	0.027542	ALS2 C-terminal like
UP in HEB	**51162**	EGFL7	-9.837102	0.9865415	0.001093	EGF-like-domain, multiple 7
UP in HEB	**58155**	PTBP2	-1.947686	0.9087951	0.259232	polypyrimidine tract binding protein 2
UP in HEB	**81932**	HDHD3	-1.683526	0.8944477	0.311321	haloacid dehalogenase-like hydrolase domain containing 3
UP in HEB	**10045**	SH2D3A	-3.892121	0.9278539	0.067353	SH2 domain containing 3A
UP in HEB	**57695**	USP37	-1.358067	0.8648639	0.390105	ubiquitin specific peptidase 37
UP in HEB	**132014**	IL17RE	-4.856517	0.9343876	0.034518	interleukin 17 receptor E
UP in HEB	**466**	ATF1	-2.240136	0.9408413	0.211666	activating transcription factor 1
UP in HEB	**7296**	TXNRD1	-1.010348	0.8875762	0.496427	thioredoxin reductase 1
UP in HEB	**23598**	PATZ1	-1.711532	0.9120753	0.305336	POZ (BTB) and AT hook containing zinc
UP in HEB	**259266**	ASPM	-1.179305	0.8823848	0.441564	asp (abnormal spindle) homolog, microcephaly associated (Drosophila)
UP in HEB	**5122**	PCSK1	-6.145434	0.9959909	0.014127	proprotein convertase subtilisin/kexin type 1
UP in HEB	**404217**	CTXN1	-1.092726	0.8727488	0.468875	cortexin 1
UP in HEB	**29904**	EEF2K	-1.060779	0.872571	0.479373	eukaryotic elongation factor-2 kinase
UP in HEB	**722**	C4BPA	-6.857981	0.8008249	0.008621	complement component 4 binding protein,
UP in HEB	**30834**	ZNRD1	-2.202058	0.9398546	0.217327	zinc ribbon domain containing 1
UP in HEB	**7704**	ZBTB16	-8.407976	0.9453126	0.002944	zinc finger and BTB domain containing 16
UP in HEB	**26512**	INTS6	-1.290265	0.8835493	0.408876	integrator complex subunit 6
UP in HEB	**79871**	RPAP2	-1.101086	0.8414493	0.466165	RNA polymerase II associated protein 2
UP in HEB	**9377**	COX5A	-1.311653	0.9015681	0.402859	cytochrome c oxidase subunit Va
UP in HEB	**51622**	CCZ1	-1.189176	0.8911231	0.438553	CCZ1 vacuolar protein trafficking and biogenesis associated homolog (S. cerevisiae)
UP in HEB	**3655**	ITGA6	-10.41222	0.997991	0.000734	integrin, alpha 6

UP in HEB	**54930**	HAUS4	-1.096808	0.8831582	0.46755	HAUS augmin-like complex, subunit 4
UP in HEB	**64087**	MCCC2	-1.181402	0.8908031	0.440923	methylcrotonoyl-CoA carboxylase 2 (beta)
UP in HEB	**57698**	KIAA1598	-1.649597	0.9177112	0.318729	KIAA1598
UP in HEB	**1877**	E4F1	-1.8867	0.9216669	0.270425	E4F transcription factor 1
UP in HEB	**55615**	PRR5	-6.190615	0.973483	0.013691	proline rich 5 (renal)
UP in HEB	**441478**	NRARP	-9.030667	0.9884794	0.001912	NOTCH-regulated ankyrin repeat protein
UP in HEB	**197407**	ZNF48	-1.322593	0.8648283	0.399816	zinc finger protein 48
UP in HEB	**64793**	CEP85	-1.315305	0.8920031	0.40184	centrosomal protein 85kDa
UP in HEB	**51363**	CHST15	-9.176589	0.9742208	0.001728	carbohydrate (N-acetylgalactosamine 4-sulfate 6-O) sulfotransferase 15
UP in HEB	**283120**	H19	-9.858758	0.9945775	0.001077	H19, imprinted maternally expressed transcript (non-protein coding)
UP in HEB	**51066**	SSUH2	-7.222392	0.8457696	0.006696	ssu-2 homolog (C. elegans)
UP in HEB	**10211**	FLOT1	-1.480775	0.9071684	0.358296	flotillin 1
UP in HEB	**1522**	CTSZ	-11.86873	0.9996	0.000267	cathepsin Z
UP in HEB	**220594**	USP32P2	-2.013297	0.804034	0.247706	ubiquitin specific peptidase 32 pseudogene 2
UP in HEB	**3485**	IGFBP2	-12.34106	0.9986577	0.000193	insulin-like growth factor binding protein 2, 36kDa
UP in HEB	**253039**	LOC25303	-2.195501	0.859068	0.218317	uncharacterized LOC253039
UP in HEB	**6573**	SLC19A1	-1.560592	0.8985101	0.339012	solute carrier family 19 (folate transporter), member 1
UP in HEB	**199857**	ALG14	-1.101628	0.8680018	0.46599	ALG14, UDP-N-acetylglucosaminyl-transferase subunit
UP in HEB	**116138**	KLHDC3	-2.344622	0.9503618	0.196879	kelch domain containing 3
UP in HEB	**5136**	PDE1A	-7.346361	0.9984177	0.006145	phosphodiesterase 1A, calmodulin-dependent
UP in HEB	**51517**	NCKIPSD	-1.695546	0.9218803	0.308738	NCK interacting protein with SH3 domain
UP in HEB	**4113**	MAGEB2	-11.59168	0.9970754	0.000324	melanoma antigen family B, 2
UP in HEB	**2108**	ETFA	-1.209572	0.8971767	0.432397	electron-transfer-flavoprotein, alpha
UP in HEB	**84832**	ANKRD36BP1	-1.298362	0.8526677	0.406587	ankyrin repeat domain 36B pseudogene 1
UP in HEB	**285367**	RPUSD3	-1.888416	0.9278806	0.270104	RNA pseudouridylate synthase domain containing 3
UP in HEB	**94005**	PIGS	-1.072688	0.8850783	0.475432	phosphatidylinositol glycan anchor biosynthesis, class S
UP in HEB	**134145**	FAM173B	-1.190103	0.8750333	0.438272	family with sequence similarity 173, member
UP in HEB	**6419**	SETMAR	-2.161854	0.9316052	0.223469	SET domain and mariner transposase fusion
UP in HEB	**56935**	SMCO4	-1.324491	0.8666596	0.39929	single-pass membrane protein with coiled-coil domains 4
UP in HEB	**4067**	LYN	-2.200792	0.9138621	0.217518	v-yes-1 Yamaguchi sarcoma viral related oncogene homolog
UP in HEB	**84787**	SUV420H2	-1.067712	0.8265863	0.477075	suppressor of variegation 4-20 homolog 2 (Drosophila)
UP in HEB	**10579**	TACC2	-1.398081	0.8911053	0.379433	transforming, acidic coiled-coil containing protein 2
UP in HEB	**2820**	GPD2	-1.198638	0.8915409	0.435686	glycerol-3-phosphate dehydrogenase 2 (mitochondrial)
UP in HEB	**117143**	TADA1	-1.604093	0.8986079	0.328942	transcriptional adaptor 1
UP in HEB	**55084**	SOBP	-7.184875	0.931783	0.006873	sine oculis binding protein homolog
UP in HEB	**360**	AQP3	-10.21257	0.9990933	0.000843	aquaporin 3 (Gill blood group)
UP in HEB	**501**	ALDH7A1	-3.991927	0.977661	0.062851	aldehyde dehydrogenase 7 family, member
UP in HEB	**9188**	DDX21	-2.061974	0.9408057	0.239488	DEAD (Asp-Glu-Ala-Asp) box helicase 21
UP in HEB	**7329**	UBE2I	-1.286653	0.898679	0.409901	ubiquitin-conjugating enzyme E2I
UP in HEB	**9244**	CRLF1	-5.033423	0.8622682	0.030534	cytokine receptor-like factor 1
UP in HEB	**9203**	ZMYM3	-1.076498	0.8819937	0.474178	zinc finger, MYM-type 3
UP in HEB	**3178**	HNRNPA1	-1.515972	0.9091952	0.349661	heterogeneous nuclear ribonucleoprotein A1
UP in HEB	**84908**	FAM136A	-1.202913	0.8955678	0.434397	family with sequence similarity 136, member

UP in HEB	**84262**	PSMG3	-1.361202	0.9020659	0.389258	proteasome (prosome, macropain) assembly chaperone 3
UP in HEB	**119504**	ANAPC16	-1.34781	0.8999147	0.392888	anaphase promoting complex subunit 16
UP in HEB	**4801**	NFYB	-1.87783	0.920458	0.272093	nuclear transcription factor Y, beta
UP in HEB	**84464**	SLX4	-1.297112	0.8206304	0.40694	SLX4 structure-specific endonuclease subunit homolog (S. cerevisiae)
UP in HEB	**5144**	PDE4D	-3.492993	0.974692	0.088819	phosphodiesterase 4D, cAMP-specific
UP in HEB	**481**	ATP1B1	-1.935134	0.9267872	0.261497	ATPase, Na+/K+ transporting, beta 1 polypeptide
UP in HEB	**79754**	ASB13	-2.058851	0.9334276	0.240007	ankyrin repeat and SOCS box containing 13
UP in HEB	**10529**	NEBL	-3.087463	0.8068164	0.117647	nebulette
UP in HEB	**9121**	SLC16A5	-8.136758	0.9880971	0.003553	solute carrier family 16, member 5 (monocarboxylic acid transporter 6)
UP in HEB	**2648**	KAT2A	-1.665627	0.9219069	0.315207	K(lysine) acetyltransferase 2A
UP in HEB	**89932**	PAPLN	-1.641231	0.8422494	0.320583	papilin, proteoglycan-like sulfated
UP in HEB	**2263**	FGFR2	-5.836839	0.9915284	0.017496	fibroblast growth factor receptor 2
UP in HEB	**4222**	MEOX1	-9.768184	0.9855992	0.001147	mesenchyme homeobox 1
UP in HEB	**7748**	ZNF195	-1.26553	0.8686508	0.415947	zinc finger protein 195
UP in HEB	**219743**	TYSND1	-1.224115	0.8881274	0.42806	trypsin domain containing 1
UP in HEB	**135293**	PM20D2	-1.401017	0.8963411	0.378662	peptidase M20 domain containing 2
UP in HEB	**9538**	EI24	-2.380508	0.9510641	0.192042	etoposide induced 2.4
UP in HEB	**51085**	MLXIPL	-3.641308	0.9510285	0.080141	MLX interacting protein-like
UP in HEB	**8572**	PDLIM4	-7.230243	0.9909862	0.00666	PDZ and LIM domain 4
UP in HEB	**1104**	RCC1	-1.378846	0.9039949	0.384526	regulator of chromosome condensation 1
UP in HEB	**84318**	CCDC77	-2.058789	0.9378278	0.240017	coiled-coil domain containing 77
UP in HEB	**100131205**	RPL21P28	-1.239086	0.8055719	0.423641	ribosomal protein L21 pseudogene 28
UP in HEB	**83746**	L3MBTL2	-1.262917	0.8927409	0.416701	l(3)mbt-like 2 (Drosophila)
UP in HEB	**23268**	DNMBP	-1.110747	0.8792202	0.463054	dynamin binding protein
UP in HEB	**493812**	HCG11	-2.796069	0.9322897	0.143979	HLA complex group 11 (non-protein coding)
UP in HEB	**23246**	BOP1	-1.128698	0.8911053	0.457328	block of proliferation 1
UP in HEB	**56256**	SERTAD4	-5.212994	0.8225594	0.026961	SERTA domain containing 4
UP in HEB	**65987**	KCTD14	-7.241586	0.8478763	0.006608	potassium channel tetramerisation domain containing 14
UP in HEB	**100303755**	PET117	-1.105493	0.8692552	0.464744	PET117 homolog (S. cerevisiae)
UP in HEB	**4651**	MYO10	-1.325	0.8994435	0.399149	myosin X
UP in HEB	**54887**	UHRF1BP	-3.608586	0.9714563	0.08198	UHRF1 binding protein 1
UP in HEB	**7582**	ZNF33B	-2.119589	0.9208047	0.230113	zinc finger protein 33B
UP in HEB	**54820**	NDE1	-1.169778	0.8828826	0.44449	nudE nuclear distribution E homolog 1 (A. nidulans)
UP in HEB	**57001**	ACN9	-2.05545	0.9281739	0.240574	ACN9 homolog (S. cerevisiae)
UP in HEB	**55958**	KLHL9	-9.865733	0.986826	0.001072	kelch-like family member 9
UP in HEB	**3855**	KRT7	-9.007321	0.9999111	0.001943	keratin 7
UP in HEB	**11277**	TREX1	-1.30586	0.8927054	0.40448	three prime repair exonuclease 1
UP in HEB	**4172**	MCM3	-1.413879	0.904866	0.375301	minichromosome maintenance complex component 3
UP in HEB	**64395**	GMCL1	-1.023723	0.8715932	0.491845	germ cell-less, spermatogenesis associated 1
UP in HEB	**25861**	DFNB31	-2.462958	0.9360944	0.181374	deafness, autosomal recessive 31
UP in HEB	**346171**	ZFP57	-9.100662	0.9720696	0.001821	ZFP57 zinc finger protein
UP in HEB	**133522**	PPARGC1 B	-1.821445	0.8720198	0.282937	peroxisome proliferator-activated receptor gamma, coactivator 1 beta
UP in HEB	**55799**	CACNA2D 3	-3.160371	0.894421	0.111849	calcium channel, voltage-dependent, alpha 2/delta subunit 3
UP in HEB	**414777**	HCG18	-1.16073	0.843965	0.447286	HLA complex group 18 (non-protein coding)
UP in HEB	**340784**	HMX3	-4.081388	0.8343645	0.059072	H6 family homeobox 3
UP in HEB	**55252**	ASXL2	-1.134518	0.8844383	0.455487	additional sex combs like 2 (Drosophila)
UP in HEB	**51053**	GMNN	-3.158842	0.9692072	0.111968	geminin, DNA replication inhibitor
UP in HEB	**139886**	SPIN4	-1.043237	0.8140968	0.485237	spindlin family, member 4

UP in HEB	**79697**	C14orf169	-3.728241	0.9627802	0.075455	chromosome 14 open reading frame 169
UP in HEB	**79627**	OGFRL1	-1.018656	0.880527	0.493576	opioid growth factor receptor-like 1
UP in HEB	**10736**	SIX2	-11.10918	0.9955998	0.000453	SIX homeobox 2
UP in HEB	**1284**	COL4A2	-3.988881	0.9818479	0.062984	collagen, type IV, alpha 2
UP in HEB	**23478**	SEC11A	-1.028377	0.887914	0.490261	SEC11 homolog A (S. cerevisiae)
UP in HEB	**6655**	SOS2	-1.21918	0.8511921	0.429527	son of sevenless homolog 2 (Drosophila)
UP in HEB	**55885**	LMO3	-10.24832	0.9976177	0.000822	LIM domain only 3 (rhombotin-like 2)
UP in HEB	**51466**	EVL	-1.066351	0.8719398	0.477525	Enah/Vasp-like
UP in HEB	**6275**	S100A4	-9.097088	0.9999378	0.001826	S100 calcium binding protein A4
UP in HEB	**23401**	FRAT2	-2.490949	0.945366	0.177889	frequently rearranged in advanced T-cell lymphomas 2
UP in HEB	**221079**	ARL5B	-1.314023	0.8736288	0.402198	ADP-ribosylation factor-like 5B
UP in HEB	**146760**	RTN4RL1	-7.481127	0.8749622	0.005597	reticulon 4 receptor-like 1
UP in HEB	**6903**	TBCC	-1.067526	0.868713	0.477137	tubulin folding cofactor C
UP in HEB	**127002**	ATXN7L2	-1.403268	0.809172	0.378072	ataxin 7-like 2
UP in HEB	**1299**	COL9A3	-7.995767	0.9914484	0.003918	collagen, type IX, alpha 3
UP in HEB	**100507178**	SLFNL1-AS1	-2.003772	0.827733	0.249347	SLFNL1 antisense RNA 1
UP in HEB	**56897**	WRNIP1	-2.150849	0.9397657	0.22518	Werner helicase interacting protein 1
UP in HEB	**8263**	F8A1	-1.640363	0.9185557	0.320776	coagulation factor VIII-associated 1
UP in HEB	**2908**	NR3C1	-1.042807	0.8834871	0.485382	nuclear receptor subfamily 3, group C, member 1 (glucocorticoid receptor)
UP in HEB	**51728**	POLR3K	-2.798501	0.9598556	0.143737	polymerase (RNA) III (DNA directed) polypeptide K, 12.3 kDa
UP in HEB	**79065**	ATG9A	-1.007282	0.8836916	0.497482	autophagy related 9A
UP in HEB	**80119**	PIF1	-1.445271	0.8795402	0.367223	PIF1 5'-to-3' DNA helicase homolog (S. cerevisiae)
UP in HEB	**899**	CCNF	-1.807116	0.9204313	0.285762	cyclin F
UP in HEB	**1979**	EIF4EBP2	-1.536288	0.9043505	0.344771	eukaryotic translation initiation factor 4E binding protein 2
UP in HEB	**84896**	ATAD1	-1.563572	0.9066617	0.338312	ATPase family, AAA domain containing 1
UP in HEB	**64771**	C6orf106	-1.847294	0.9261738	0.277913	chromosome 6 open reading frame 106
UP in HEB	**57462**	KIAA1161	-3.158812	0.9568066	0.11197	KIAA1161
UP in HEB	**100287569**	LINC0017	-5.368339	0.8688463	0.024209	long intergenic non-protein coding RNA 173
UP in HEB	**1503**	CTPS1	-1.459703	0.9066528	0.363568	CTP synthase 1
UP in HEB	**339366**	ADAMTSL5	-7.533979	0.8802958	0.005396	ADAMTS-like 5
UP in HEB	**6195**	RPS6KA1	-1.022191	0.8818959	0.492368	ribosomal protein S6 kinase, 90kDa,
UP in HEB	**10607**	TBL3	-1.224686	0.8929543	0.427891	transducin (beta)-like 3
UP in HEB	**64220**	STRA6	-4.21893	0.9869326	0.0537	stimulated by retinoic acid 6
UP in HEB	**90784**	LOC90784	-1.523625	0.8864473	0.347811	uncharacterized LOC90784
UP in HEB	**55609**	ZNF280C	-1.32474	0.8001671	0.399221	zinc finger protein 280C
UP in HEB	**283643**	C14orf80	-1.048975	0.8799936	0.483311	chromosome 14 open reading frame 80
UP in HEB	**9055**	PRC1	-1.302748	0.9008125	0.405353	protein regulator of cytokinesis 1
UP in HEB	**85007**	AGXT2L2	-2.272864	0.9021014	0.206919	alanine-glyoxylate aminotransferase 2-like 2
UP in HEB	**8531**	YBX3	-1.034887	0.8877273	0.488054	Y box binding protein 3
UP in HEB	**5763**	PTMS	-1.396992	0.9053372	0.37972	parathymosin
UP in HEB	**23475**	QPRT	-2.453377	0.9502196	0.182583	quinolinate phosphoribosyltransferase
UP in HEB	**55703**	POLR3B	-2.326651	0.9336854	0.199346	polymerase (RNA) III (DNA directed) polypeptide B
UP in HEB	**162073**	ITPRIPL2	-1.769071	0.9210536	0.293398	inositol 1,4,5-trisphosphate receptor interacting protein-like 2
UP in HEB	**55787**	TXLNG	-1.425636	0.8972301	0.372255	taxilin gamma
UP in HEB	**388630**	TRABD2B	-3.67985	0.8298309	0.078029	TraB domain containing 2B
UP in HEB	**153684**	LOC15368	-2.646074	0.8390848	0.159754	uncharacterized LOC153684
UP in HEB	**29965**	CDIP1	-6.415037	0.992204	0.011719	cell death-inducing p53 target 1
UP in HEB	**268**	AMH	-1.304153	0.8808648	0.404959	anti-Mullerian hormone

UP in HEB	**10024**	TROAP	-2.022692	0.939739	0.246098	trophinin associated protein
UP in HEB	**23221**	RHOBTB2	-1.471652	0.9019503	0.360569	Rho-related BTB domain containing 2
UP in HEB	**23588**	KLHDC2	-1.016484	0.879478	0.49432	kelch domain containing 2
UP in HEB	**146540**	ZNF785	-1.477493	0.8485697	0.359112	zinc finger protein 785
UP in HEB	**7803**	PTP4A1	-2.448704	0.9522997	0.183175	protein tyrosine phosphatase type IVA, member 1
UP in HEB	**7249**	TSC2	-1.241849	0.8893008	0.42283	tuberous sclerosis 2
UP in HEB	**441058**	MGC3958	-2.957102	0.8075542	0.128773	uncharacterized LOC441058
UP in HEB	**1364**	CLDN4	-2.595105	0.9373211	0.165499	claudin 4
UP in HEB	**23524**	SRRM2	-2.154107	0.9417302	0.224672	serine/arginine repetitive matrix 2
UP in HEB	**155400**	NSUN5P1	-1.447511	0.8956211	0.366653	NOP2/Sun domain family, member 5 pseudogene 1
UP in HEB	**7866**	IFRD2	-1.332505	0.9024748	0.397078	interferon-related developmental regulator 2
UP in HEB	**56948**	SDR39U1	-1.278448	0.8954433	0.412239	short chain dehydrogenase/reductase family 39U, member 1
UP in HEB	**22836**	RHOBTB3	-3.377047	0.9755276	0.096252	Rho-related BTB domain containing 3
UP in HEB	**151230**	KLHL23	-4.65685	0.9724519	0.039641	kelch-like family member 23
UP in HEB	**65260**	SELRC1	-2.567562	0.9494106	0.168689	Sel1 repeat containing 1
UP in HEB	**80142**	PTGES2	-1.006726	0.8846427	0.497674	prostaglandin E synthase 2
UP in HEB	**684**	BST2	-11.16332	0.99984	0.000436	bone marrow stromal cell antigen 2
UP in HEB	**83732**	RIOK1	-3.098311	0.9658737	0.116766	RIO kinase 1
UP in HEB	**400322**	HERC2P2	-1.469156	0.8991235	0.361194	hect domain and RLD 2 pseudogene 2
UP in HEB	**94234**	FOXQ1	-10.21917	0.9905239	0.000839	forkhead box Q1
UP in HEB	**286262**	TPRN	-1.254127	0.8777357	0.419247	taperin
UP in HEB	**10560**	SLC19A2	-1.3931	0.8715843	0.380746	solute carrier family 19 (thiamine transporter), member 2
UP in HEB	**9112**	MTA1	-1.115081	0.8895852	0.461665	metastasis associated 1
UP in HEB	**89839**	ARHGAP1 1B	-1.2155	0.8710065	0.430624	Rho GTPase activating protein 11B
UP in HEB	**1832**	DSP	-11.77633	0.9984088	0.000285	desmoplakin
UP in HEB	**27314**	RAB30	-2.515152	0.9281828	0.17493	RAB30, member RAS oncogene family
UP in HEB	**92092**	ZC3HAV1	-1.609945	0.8890074	0.327611	zinc finger CCCH-type, antiviral 1-like
UP in HEB	**26873**	OPLAH	-10.98276	0.9951464	0.000494	5-oxoprolinase (ATP-hydrolysing)
UP in HEB	**57460**	PPM1H	-2.771504	0.9224848	0.146452	protein phosphatase, Mg2+/Mn2+ dependent, 1H
UP in HEB	**441394**	SUGT1P1	-1.426174	0.8051541	0.372116	suppressor of G2 allele of SKP1 (S. cerevisiae) pseudogene 1
UP in HEB	**54475**	NLE1	-1.191763	0.8876829	0.437768	notchless homolog 1 (Drosophila)
UP in HEB	**30818**	KCNIP3	-7.426265	0.8687397	0.005814	Kv channel interacting protein 3, calsenilin
UP in HEB	**10635**	RAD51AP	-1.282487	0.8954255	0.411086	RAD51 associated protein 1
UP in HEB	**65108**	MARCKS	-4.456467	0.9869771	0.045548	MARCKS-like 1
UP in HEB	**84951**	TNS4	-11.45296	0.9984088	0.000357	tensin 4
UP in HEB	**123036**	TC2N	-8.207828	0.9338543	0.003382	tandem C2 domains, nuclear
UP in HEB	**11339**	OIP5	-2.865392	0.9620069	0.137224	Opa interacting protein 5
UP in HEB	**90864**	SPSB3	-1.36002	0.8955055	0.389577	splA/ryanodine receptor domain and SOCS box containing 3
UP in HEB	**57711**	ZNF529	-3.129283	0.8519832	0.114286	zinc finger protein 529
UP in HEB	**4023**	LPL	-11.86857	0.9976977	0.000267	lipoprotein lipase
UP in HEB	**3020**	H3F3A	-1.134446	0.8930076	0.45551	H3 histone, family 3A
UP in HEB	**6446**	SGK1	-3.070362	0.9689939	0.11905	serum/glucocorticoid regulated kinase 1
UP in HEB	**343099**	CCDC18	-1.391094	0.8421249	0.381275	coiled-coil domain containing 18
UP in HEB	**10979**	FERMT2	-1.58688	0.9196935	0.332891	fermitin family member 2
UP in HEB	**1831**	TSC22D3	-1.952322	0.9270183	0.2584	TSC22 domain family, member 3
UP in HEB	**113000**	RPUSD1	-1.45547	0.9032926	0.364636	RNA pseudouridylate synthase domain containing 1
UP in HEB	**5557**	PRIM1	-1.092463	0.8853183	0.46896	primase, DNA, polypeptide 1 (49kDa)

UP in HEB	**9806**	SPOCK2	-4.21453	0.820106	0.053864	sparc/osteonectin, cwcv and kazal-like domains proteoglycan (testican) 2
UP in HEB	**9147**	NEMF	-1.211403	0.8829449	0.431849	nuclear export mediator factor
UP in HEB	**255403**	ZNF718	-7.377934	0.8633705	0.006012	zinc finger protein 718
UP in HEB	**2619**	GAS1	-11.30797	0.9996889	0.000394	growth arrest-specific 1
UP in HEB	**6720**	SREBF1	-2.422127	0.9504685	0.186581	sterol regulatory element binding transcription factor 1
UP in HEB	**10018**	BCL2L11	-2.935708	0.9537398	0.130696	BCL2-like 11 (apoptosis facilitator)
UP in HEB	**3151**	HMGN2	-1.494403	0.908244	0.354928	high mobility group nucleosomal binding domain 2
UP in HEB	**27290**	SPINK4	-4.005999	0.8365957	0.062241	serine peptidase inhibitor, Kazal type 4
UP in HEB	**7067**	THRA	-1.428922	0.9019148	0.371408	thyroid hormone receptor, alpha
UP in HEB	**6599**	SMARCC1	-2.142163	0.9406013	0.22654	SWI/SNF related, matrix associated, actin dependent regulator of chromatin, subfamily c, member 1
UP in HEB	**1967**	EIF2B1	-1.197623	0.8950344	0.435993	eukaryotic translation initiation factor 2B, subunit 1 alpha, 26kDa
UP in HEB	**339456**	TMEM52	-7.279224	0.8522588	0.006438	transmembrane protein 52
UP in HEB	**284098**	PIGW	-1.494417	0.9004302	0.354924	phosphatidylinositol glycan anchor biosynthesis, class W
UP in HEB	**10825**	NEU3	-1.748885	0.8748555	0.297532	sialidase 3 (membrane sialidase)
UP in HEB	**100506233**	LOC100506233	-1.18071	0.8669707	0.441134	uncharacterized LOC100506233
UP in HEB	**8019**	BRD3	-1.111636	0.8694864	0.462769	bromodomain containing 3
UP in HEB	**26032**	SUSD5	-5.905554	0.977821	0.016682	sushi domain containing 5
UP in HEB	**2159**	F10	-2.924131	0.8907231	0.131749	coagulation factor X
UP in HEB	**6161**	RPL32	-1.278543	0.9008036	0.412212	ribosomal protein L32
UP in HEB	**7629**	ZNF76	-1.165439	0.8811314	0.445829	zinc finger protein 76
UP in HEB	**1632**	ECI1	-2.528419	0.952673	0.173328	enoyl-CoA delta isomerase 1
UP in HEB	**6876**	TAGLN	-1.873215	0.8936388	0.272965	transgelin
UP in HEB	**23020**	SNRNP20	-1.034193	0.8872029	0.488289	small nuclear ribonucleoprotein 200kDa (U5)
UP in HEB	**728411**	GUSBP1	-1.514682	0.9000036	0.349974	glucuronidase, beta pseudogene 1
UP in HEB	**8436**	SDPR	-7.721099	0.8982524	0.004739	serum deprivation response
UP in HEB	**516**	ATP5G1	-1.104754	0.8922254	0.464982	ATP synthase, H+ transporting, mitochondrial Fo complex, subunit C1 (subunit 9)
UP in HEB	**80311**	KLHL15	-1.070627	0.8315732	0.476112	kelch-like family member 15
UP in HEB	**57415**	C3orf14	-3.01186	0.8879051	0.123977	chromosome 3 open reading frame 14
UP in HEB	**92703**	TMEM183	-1.462984	0.9051327	0.362742	transmembrane protein 183A
UP in HEB	**55183**	RIF1	-1.213181	0.8856828	0.431317	RAP1 interacting factor homolog (yeast)
UP in HEB	**9099**	USP2	-4.260886	0.889203	0.052161	ubiquitin specific peptidase 2
UP in HEB	**133686**	NADKD1	-1.516389	0.8940832	0.34956	NAD kinase domain containing 1
UP in HEB	**219**	ALDH1B1	-1.358323	0.8963322	0.390035	aldehyde dehydrogenase 1 family, member
UP in HEB	**9533**	POLR1C	-1.444345	0.9018437	0.367459	polymerase (RNA) I polypeptide C, 30kDa
UP in HEB	**11076**	TPPP	-3.357552	0.8083276	0.097561	tubulin polymerization promoting protein
UP in HEB	**50937**	CDON	-1.793657	0.8265507	0.28844	cell adhesion associated, oncogene regulated
UP in HEB	**5253**	PHF2	-1.046421	0.8547834	0.484168	PHD finger protein 2
UP in HEB	**3146**	HMGB1	-1.14685	0.8945099	0.45161	high mobility group box 1
UP in HEB	**728643**	HNRNPA1P33	-10.78109	0.994293	0.000568	heterogeneous nuclear ribonucleoprotein A1 pseudogene 33
UP in HEB	**4100**	MAGEA1	-2.368583	0.947455	0.193636	melanoma antigen family A, 1 (directs expression of antigen MZ2-E)
UP in HEB	**5987**	TRIM27	-1.541002	0.9069195	0.343647	tripartite motif containing 27
UP in HEB	**80162**	ATHL1	-4.646565	0.9834925	0.039925	ATH1, acid trehalase-like 1 (yeast)
UP in HEB	**125111**	GJD3	-4.84747	0.8676907	0.034735	gap junction protein, delta 3, 31.9kDa
UP in HEB	**8243**	SMC1A	-1.734688	0.9229292	0.300474	structural maintenance of chromosomes 1A
UP in HEB	**220965**	FAM13C	-2.601706	0.9210802	0.164744	family with sequence similarity 13, member

UP in HEB	**1075**	CTSC	-1.940165	0.9289562	0.260587	cathepsin C
UP in HEB	**57761**	TRIB3	-2.212619	0.9393034	0.215742	tribbles homolog 3 (Drosophila)
UP in HEB	**59307**	SIGIRR	-4.355133	0.9752342	0.048862	single immunoglobulin and toll-interleukin 1 receptor (TIR) domain
UP in HEB	**9315**	NREP	-1.88755	0.9213469	0.270266	neuronal regeneration related protein
UP in HEB	**80020**	FOXRED2	-2.651513	0.9530019	0.159153	FAD-dependent oxidoreductase domain containing 2
UP in HEB	**10057**	ABCC5	-1.855017	0.9237826	0.276429	ATP-binding cassette, sub-family C (CFTR/MRP), member 5
UP in HEB	**1604**	CD55	-1.978873	0.9283251	0.253688	CD55 molecule, decay accelerating factor for complement (Cromer blood group)
UP in HEB	**100885779**	LINC-ROR	-7.318919	0.9395968	0.006263	long intergenic non-protein coding RNA, regulator of reprogramming
UP in HEB	**401944**	LDLRAD2	-2.831847	0.9372411	0.140452	low density lipoprotein receptor class A domain containing 2
UP in HEB	**5820**	PVT1	-2.008487	0.9333831	0.248534	Pvt1 oncogene (non-protein coding)
UP in HEB	**1295**	COL8A1	-1.027014	0.8719309	0.490725	collagen, type VIII, alpha 1
UP in HEB	**387254**	SLC7A5P2	-1.134338	0.8412538	0.455544	solute carrier family 7 (amino acid transporter light chain, L system), member 5 pseudogene 2
UP in HEB	**10964**	IFI44L	-2.197658	0.9082796	0.217991	interferon-induced protein 44-like
UP in HEB	**4055**	LTBR	-7.966713	0.9989244	0.003997	lymphotoxin beta receptor (TNFR superfamily, member 3)
UP in HEB	**79899**	PRR5L	-2.967587	0.9557754	0.12784	proline rich 5 like
UP in HEB	**251**	ALPPL2	-7.193115	0.8422938	0.006834	alkaline phosphatase, placental-like 2
UP in HEB	**54947**	LPCAT2	-2.043548	0.9078351	0.242567	lysophosphatidylcholine acyltransferase 2
UP in HEB	**55769**	ZNF83	-7.483816	0.8749622	0.005587	zinc finger protein 83
UP in HEB	**55268**	ECHDC2	-7.196397	0.8434761	0.006818	enoyl CoA hydratase domain containing 2
UP in HEB	**80324**	PUS1	-1.36089	0.9004391	0.389342	pseudouridylate synthase 1
UP in HEB	**81620**	CDT1	-1.058617	0.8830071	0.480092	chromatin licensing and DNA replication
UP in HEB	**1382**	CRABP2	-1.688785	0.9241737	0.310188	cellular retinoic acid binding protein 2
UP in HEB	**729096**	BMS1P4	-1.586993	0.8891585	0.332865	BMS1 pseudogene 4
UP in HEB	**3691**	ITGB4	-5.19884	0.9758032	0.027227	integrin, beta 4
UP in HEB	**5881**	RAC3	-3.539092	0.9759632	0.086025	ras-related C3 botulinum toxin substrate 3 (rho family, small GTP binding protein Rac3)
UP in HEB	**292**	SLC25A5	-1.097122	0.8923587	0.467448	solute carrier family 25 (mitochondrial carrier; adenine nucleotide translocator), member 5
UP in HEB	**84457**	PHYHIPL	-3.034573	0.8298487	0.12204	phytanoyl-CoA 2-hydroxylase interacting protein-like
UP in HEB	**25805**	BAMBI	-4.345775	0.8214127	0.04918	BMP and activin membrane-bound inhibitor homolog (Xenopus laevis)
UP in HEB	**23212**	RRS1	-1.084633	0.8877273	0.471512	RRS1 ribosome biogenesis regulator homolog (S. cerevisiae)
UP in HEB	**79602**	ADIPOR2	-1.03477	0.8846161	0.488094	adiponectin receptor 2
UP in HEB	**728613**	LOC72861	-3.655501	0.9561132	0.079357	programmed cell death 6 pseudogene
UP in HEB	**3321**	IGSF3	-2.381181	0.9201735	0.191952	immunoglobulin superfamily, member 3
UP in HEB	**9415**	FADS2	-1.584963	0.9020126	0.333333	fatty acid desaturase 2
UP in HEB	**81688**	C6orf62	-1.300276	0.9000124	0.406048	chromosome 6 open reading frame 62
UP in HEB	**54675**	CRLS1	-1.078749	0.8549167	0.473439	cardiolipin synthase 1
UP in HEB	**4123**	MAN2C1	-1.474029	0.9030971	0.359976	mannosidase, alpha, class 2C, member 1
UP in HEB	**6929**	TCF3	-1.265886	0.8976123	0.415844	transcription factor 3
UP in HEB	**84245**	MRI1	-1.81644	0.9219247	0.283921	methylthioribose-1-phosphate isomerase homolog (S. cerevisiae)
UP in HEB	**388524**	RPSAP58	-1.807693	0.9275517	0.285647	ribosomal protein SA pseudogene 58
UP in HEB	**8969**	HIST1H2A	-3.811351	0.9467972	0.071231	histone cluster 1, H2ag

UP in HEB	**3959**	LGALS3BP	-1.444582	0.9066617	0.367399	lectin, galactoside-binding, soluble, 3 binding protein
UP in HEB	**220042**	C11orf82	-1.188854	0.8728554	0.438651	chromosome 11 open reading frame 82
UP in HEB	**148206**	ZNF714	-2.72995	0.8795314	0.150731	zinc finger protein 714
UP in HEB	**138716**	RPP25L	-1.178893	0.8910075	0.44169	ribonuclease P/MRP 25kDa subunit-like
UP in HEB	**10446**	LRRN2	-6.378512	0.8315999	0.012019	leucine rich repeat neuronal 2
UP in HEB	**158234**	TRMT10B	-2.251251	0.8830515	0.210042	tRNA methyltransferase 10 homolog B (S. cerevisiae)
UP in HEB	**115004**	MB21D1	-9.762105	0.9855192	0.001152	Mab-21 domain containing 1
UP in HEB	**64083**	GOLPH3	-1.508391	0.9081907	0.351503	golgi phosphoprotein 3 (coat-protein)
UP in HEB	**100132356**	LOC100132356	-2.606658	0.8658328	0.164179	uncharacterized LOC100132356
UP in HEB	**79609**	METTL21	-1.317666	0.8487386	0.401183	methyltransferase like 21D
UP in HEB	**344787**	ZNF860	-4.732953	0.9024659	0.037604	zinc finger protein 860
UP in HEB	**7003**	TEAD1	-1.156404	0.8927943	0.448629	TEA domain family member 1 (SV40 transcriptional enhancer factor)
UP in HEB	**9265**	CYTH3	-1.127936	0.8760467	0.45757	cytohesin 3
UP in HEB	**64757**	1-Mar	-10.81129	0.9987466	0.000557	mitochondrial amidoxime reducing
UP in HEB	**57819**	LSM2	-2.004611	0.9394101	0.249202	LSM2 homolog, U6 small nuclear RNA associated (S. cerevisiae)
UP in HEB	**55605**	KIF21A	-1.279016	0.8800558	0.412077	kinesin family member 21A
UP in HEB	**6712**	SPTBN2	-7.849543	0.9910662	0.004336	spectrin, beta, non-erythrocytic 2
UP in HEB	**4863**	NPAT	-1.487886	0.882776	0.356535	nuclear protein, ataxia-telangiectasia locus
UP in HEB	**4628**	MYH10	-6.67544	0.9946842	0.009783	myosin, heavy chain 10, non-muscle
UP in HEB	**283755**	HERC2P3	-2.477015	0.9315786	0.179616	hect domain and RLD 2 pseudogene 3
UP in HEB	**5810**	RAD1	-1.099799	0.8799403	0.466581	RAD1 homolog (S. pombe)
UP in HEB	**728**	C5AR1	-2.025773	0.919249	0.245574	complement component 5a receptor 1
UP in HEB	**5831**	PYCR1	-1.83631	0.9258894	0.280037	pyrroline-5-carboxylate reductase 1
UP in HEB	**55846**	ITFG2	-1.031353	0.8563034	0.489251	integrin alpha FG-GAP repeat containing 2
UP in HEB	**84626**	KRBA1	-1.177353	0.8438406	0.442162	KRAB-A domain containing 1
UP in HEB	**7398**	USP1	-1.240792	0.8969812	0.42314	ubiquitin specific peptidase 1
UP in HEB	**6894**	TARBP1	-1.028635	0.8517254	0.490174	TAR (HIV-1) RNA binding protein 1
UP in HEB	**4902**	NRTN	-7.149747	0.8375469	0.007042	neurturin
UP in HEB	**55695**	NSUN5	-1.355498	0.8968389	0.3908	NOP2/Sun domain family, member 5
UP in HEB	**55055**	ZWILCH	-1.081159	0.8873184	0.472649	zwilch kinetochore protein
UP in HEB	**339324**	ZNF260	-2.558183	0.8659573	0.169789	zinc finger protein 260
UP in HEB	**1975**	EIF4B	-1.671499	0.9245915	0.313927	eukaryotic translation initiation factor 4B
UP in HEB	**203197**	C9orf91	-1.287934	0.8312266	0.409537	chromosome 9 open reading frame 91
UP in HEB	**146664**	MGAT5B	-4.310396	0.9770566	0.050401	mannosyl (alpha-1,6-)-glycoprotein beta-1,6-N-acetyl-glucosaminyltransferase, isozyme B
UP in HEB	**54504**	CPVL	-5.917007	0.9832524	0.01655	carboxypeptidase, vitellogenic-like
UP in HEB	**79605**	PGBD5	-7.008028	0.9339343	0.007769	piggyBac transposable element derived 5
UP in HEB	**4582**	MUC1	-4.560996	0.9889505	0.042365	mucin 1, cell surface associated
UP in HEB	**8519**	IFITM1	-10.26927	0.9991288	0.00081	interferon induced transmembrane protein 1
UP in HEB	**23149**	FCHO1	-3.742964	0.830391	0.074689	FCH domain only 1
UP in HEB	**388561**	ZNF761	-3.714843	0.8649972	0.076159	zinc finger protein 761
UP in HEB	**91461**	PKDCC	-6.989211	0.9974132	0.007871	protein kinase domain containing,
UP in HEB	**26**	ABP1	-12.11146	0.9982132	0.000226	amiloride binding protein 1 (amine oxidase (copper-containing))
UP in HEB	**51780**	KDM3B	-1.054523	0.8779402	0.481457	lysine (K)-specific demethylase 3B
UP in HEB	**677777**	SCARNA1	-2.23848	0.9300407	0.211909	small Cajal body-specific RNA 12
UP in HEB	**3641**	INSL4	-9.134426	0.9728874	0.001779	insulin-like 4 (placenta)
UP in HEB	**4128**	MAOA	-2.095786	0.9332142	0.233941	monoamine oxidase A
UP in HEB	**64388**	GREM2	-6.33985	0.9850125	0.012346	gremlin 2, DAN family BMP antagonist
UP in HEB	**6048**	RNF5	-2.197295	0.941668	0.218046	ring finger protein 5, E3 ubiquitin protein
UP in HEB	**23022**	PALLD	-2.283515	0.9423347	0.205397	palladin, cytoskeletal associated protein
UP in HEB	**55247**	NEIL3	-1.625835	0.9020392	0.324022	nei endonuclease VIII-like 3 (E. coli)

UP in HEB	**27101**	CACYBP	-1.039403	0.8848472	0.486529	calcyclin binding protein
UP in HEB	**64110**	MAGEF1	-1.324607	0.8994169	0.399258	melanoma antigen family F, 1
UP in HEB	**84886**	C1orf198	-1.123317	0.8846694	0.459037	chromosome 1 open reading frame 198
UP in HEB	**57118**	CAMK1D	-1.289218	0.8631127	0.409173	calcium/calmodulin-dependent protein kinase
UP in HEB	**1890**	TYMP	-1.601472	0.8941899	0.329541	thymidine phosphorylase
UP in HEB	**392255**	GDF6	-7.546894	0.8817626	0.005348	growth differentiation factor 6
UP in HEB	**155060**	LOC15506	-1.840893	0.858508	0.279149	AI894139 pseudogene
UP in HEB	**91624**	NEXN	-1.217754	0.8715665	0.429952	nexilin (F actin binding protein)
UP in HEB	**2150**	F2RL1	-2.869896	0.9093196	0.136797	coagulation factor II (thrombin) receptor-like
UP in HEB	**642**	BLMH	-1.706604	0.9215514	0.30638	bleomycin hydrolase
UP in HEB	**23650**	TRIM29	-6.105035	0.9239693	0.014528	tripartite motif containing 29
UP in HEB	**113026**	PLCD3	-2.02158	0.93829	0.246288	phospholipase C, delta 3
UP in HEB	**55144**	LRRC8D	-1.863861	0.9195957	0.27474	leucine rich repeat containing 8 family,
UP in HEB	**100170841**	C17orf96	-2.234345	0.9296318	0.212518	chromosome 17 open reading frame 96
UP in HEB	**9688**	NUP93	-1.013459	0.8854161	0.495357	nucleoporin 93kDa
UP in HEB	**5129**	CDK18	-5.752659	0.9568688	0.018547	cyclin-dependent kinase 18
UP in HEB	**4681**	NBL1	-1.284208	0.8862962	0.410596	neuroblastoma 1, DAN family BMP
UP in HEB	**8076**	MFAP5	-5.489055	0.9950931	0.022265	microfibrillar associated protein 5
UP in HEB	**7869**	SEMA3B	-3.086295	0.9669671	0.117742	sema domain, immunoglobulin domain (Ig), short basic domain, secreted, (semaphorin) 3B
UP in HEB	**3628**	INPP1	-1.029771	0.8488897	0.489788	inositol polyphosphate-1-phosphatase
UP in HEB	**4176**	MCM7	-1.258612	0.8998613	0.417946	minichromosome maintenance complex component 7
UP in HEB	**126326**	GIPC3	-5.41018	0.9864526	0.023517	GIPC PDZ domain containing family,
UP in HEB	**23235**	SIK2	-1.463473	0.8937099	0.362619	salt-inducible kinase 2
UP in HEB	**440519**	ZNF724P	-2.764446	0.810381	0.14717	zinc finger protein 724, pseudogene
UP in HEB	**4815**	NINJ2	-6.501942	0.9717496	0.011034	ninjurin 2
UP in HEB	**9790**	BMS1	-1.240051	0.8938077	0.423358	BMS1 homolog, ribosome assembly protein (yeast)
UP in HEB	**4928**	NUP98	-1.049117	0.8862962	0.483264	nucleoporin 98kDa
UP in HEB	**84146**	ZNF644	-1.186631	0.866624	0.439327	zinc finger protein 644
UP in HEB	**84517**	ACTRT3	-6.457088	0.8925365	0.011382	actin-related protein T3
UP in HEB	**10101**	NUBP2	-1.776511	0.9248138	0.291889	nucleotide binding protein 2
UP in HEB	**57128**	LYRM4	-1.39998	0.9010792	0.378935	LYR motif containing 4
UP in HEB	**84191**	FAM96A	-1.12677	0.8924209	0.45794	family with sequence similarity 96, member
UP in HEB	**22998**	LIMCH1	-10.55703	0.9987999	0.000664	LIM and calponin homology domains 1
UP in HEB	**10942**	PRSS21	-12.62426	0.9990933	0.000158	protease, serine, 21 (testisin)
UP in HEB	**158511**	CSAG1	-5.083479	0.991004	0.029493	chondrosarcoma associated gene 1
UP in HEB	**493861**	EID3	-3.555789	0.956691	0.085036	EP300 interacting inhibitor of differentiation
UP in HEB	**55534**	MAML3	-4.564457	0.9573399	0.042263	mastermind-like 3 (Drosophila)
UP in HEB	**79731**	NARS2	-1.618119	0.9064039	0.32576	asparaginyl-tRNA synthetase 2, mitochondrial (putative)
UP in HEB	**282969**	FUOM	-3.013327	0.9626113	0.123851	fucose mutarotase
UP in HEB	**6398**	SECTM1	-5.73954	0.9644514	0.018717	secreted and transmembrane 1
UP in HEB	**115939**	TSR3	-1.134859	0.890972	0.455379	TSR3, 20S rRNA accumulation, homolog (S. cerevisiae)
UP in HEB	**2289**	FKBP5	-5.671802	0.9963643	0.019616	FK506 binding protein 5
UP in HEB	**2021**	ENDOG	-1.376568	0.9011592	0.385134	endonuclease G
UP in HEB	**400916**	CHCHD10	-1.890257	0.9278095	0.269759	coiled-coil-helix-coiled-coil-helix domain containing 10
UP in HEB	**6667**	SP1	-1.008257	0.8791402	0.497147	Sp1 transcription factor
UP in HEB	**408**	ARRB1	-3.487017	0.9644514	0.089187	arrestin, beta 1
UP in HEB	**3601**	IL15RA	-2.262339	0.8468452	0.208434	interleukin 15 receptor, alpha
UP in HEB	**9091**	PIGQ	-1.015083	0.8708287	0.4948	phosphatidylinositol glycan anchor biosynthesis, class Q
UP in HEB	**642826**	BMS1P6	-1.29535	0.8177769	0.407437	BMS1 pseudogene 6

UP in HEB	**28973**	MRPS18B	-1.648048	0.9232937	0.319072	mitochondrial ribosomal protein S18B
UP in HEB	**135932**	TMEM139	-4.62829	0.9708873	0.040434	transmembrane protein 139
UP in HEB	**7134**	TNNC1	-7.40031	0.9933152	0.005919	troponin C type 1 (slow)
UP in HEB	**3156**	HMGCR	-1.012502	0.8775668	0.495686	3-hydroxy-3-methylglutaryl-CoA reductase
UP in HEB	**3303**	HSPA1A	-9.678001	0.9998844	0.001221	heat shock 70kDa protein 1A
UP in HEB	**23683**	PRKD3	-1.503932	0.9062172	0.352591	protein kinase D3
UP in HEB	**1854**	DUT	-1.031111	0.886954	0.489333	deoxyuridine triphosphatase
UP in HEB	**28960**	DCPS	-2.198704	0.9370455	0.217833	decapping enzyme, scavenger
UP in HEB	**285958**	SNHG15	-1.404867	0.9013814	0.377653	small nucleolar RNA host gene 15 (non-protein coding)
UP in HEB	**23642**	SNHG1	-1.012537	0.8864651	0.495674	small nucleolar RNA host gene 1 (non-protein coding)
UP in HEB	**284004**	HEXDC	-2.115957	0.9114531	0.230693	hexosaminidase (glycosyl hydrolase family 20, catalytic domain) containing
UP in HEB	**6540**	SLC6A13	-3.784635	0.8149857	0.072562	solute carrier family 6 (neurotransmitter transporter, GABA), member 13
UP in HEB	**129293**	TRABD2A	-2.399324	0.9493129	0.189553	TraB domain containing 2A
UP in HEB	**197335**	WDR90	-2.217614	0.9333742	0.214997	WD repeat domain 90
UP in HEB	**5530**	PPP3CA	-2.83289	0.9438281	0.140351	protein phosphatase 3, catalytic subunit, alpha isozyme
UP in HEB	**80728**	ARHGAP3	-1.413635	0.8547923	0.375365	Rho GTPase activating protein 39
UP in HEB	**100289341**	LOC100289341	-2.077041	0.8614593	0.237	uncharacterized LOC100289341
UP in HEB	**10392**	NOD1	-1.84285	0.873211	0.278771	nucleotide-binding oligomerization domain containing 1
UP in HEB	**60386**	SLC25A19	-2.053325	0.9361744	0.240928	solute carrier family 25 (mitochondrial thiamine pyrophosphate carrier), member 19
UP in HEB	**57116**	ZNF695	-5.362756	0.9458549	0.024302	zinc finger protein 695
UP in HEB	**28982**	FLVCR1	-1.372595	0.8596725	0.386196	feline leukemia virus subgroup C cellular receptor 1
UP in HEB	**5801**	PTPRR	-2.352516	0.9285473	0.195804	protein tyrosine phosphatase, receptor type,
UP in HEB	**5287**	PIK3C2B	-1.959256	0.8852028	0.257161	phosphatidylinositol-4-phosphate 3-kinase, catalytic subunit type 2 beta
UP in HEB	**160760**	PPTC7	-1.305372	0.8936566	0.404617	PTC7 protein phosphatase homolog (S. cerevisiae)
UP in HEB	**9847**	C2CD5	-1.383762	0.8889719	0.383218	C2 calcium-dependent domain containing 5
UP in HEB	**1763**	DNA2	-1.645536	0.9101552	0.319628	DNA replication helicase 2 homolog (yeast)
UP in HEB	**224**	ALDH3A2	-1.363726	0.8992213	0.388577	aldehyde dehydrogenase 3 family, member
UP in HEB	**54811**	ZNF562	-1.969975	0.8763667	0.255257	zinc finger protein 562
UP in HEB	**2064**	ERBB2	-3.482626	0.9724341	0.089459	v-erb-b2 erythroblastic leukemia viral oncogene homolog 2, neuro/glioblastoma derived oncogene homolog (avian)
UP in HEB	**5424**	POLD1	-1.24241	0.8949811	0.422666	polymerase (DNA directed), delta 1, catalytic subunit
UP in HEB	**643650**	LINC0084	-7.741467	0.8998524	0.004673	long intergenic non-protein coding RNA 842
UP in HEB	**6923**	TCEB2	-1.197378	0.8970523	0.436067	transcription elongation factor B (SIII), polypeptide 2 (18kDa, elongin B)
UP in HEB	**5919**	RARRES2	-9.278449	0.9768077	0.00161	retinoic acid receptor responder (tazarotene induced) 2
UP in HEB	**1663**	DDX11	-1.507609	0.9053994	0.351693	DEAD/H (Asp-Glu-Ala-Asp/His) box
UP in HEB	**134637**	ADAT2	-1.17147	0.8192526	0.443969	adenosine deaminase, tRNA-specific 2
UP in HEB	**79133**	NDUFAF5	-1.129506	0.8276619	0.457072	NADH dehydrogenase (ubiquinone) complex I, assembly factor 5
UP in HEB	**25942**	SIN3A	-1.190182	0.8908831	0.438247	SIN3 transcription regulator homolog A
UP in HEB	**10614**	HEXIM1	-1.318244	0.89611	0.401023	hexamethylene bis-acetamide inducible 1
UP in HEB	**171546**	SPTSSA	-1.88562	0.9236404	0.270627	serine palmitoyltransferase, small subunit A
UP in HEB	**23526**	HMHA1	-1.426265	0.8547656	0.372093	histocompatibility (minor) HA-1

UP in HEB	**51809**	GALNT7	-1.059955	0.8790158	0.479647	UDP-N-acetyl-alpha-D-galactosamine:polypeptide N-acetylgalactosaminyltransferase 7 (GalNAc-T7)
UP in HEB	**57291**	DANCR	-1.288805	0.8998347	0.40929	differentiation antagonizing non-protein coding RNA
UP in HEB	**10642**	IGF2BP1	-9.053021	0.9887016	0.001883	insulin-like growth factor 2 mRNA binding protein 1
UP in HEB	**7089**	TLE2	-10.167	0.9900706	0.00087	transducin-like enhancer of split 2 (E(sp1) homolog, Drosophila)
UP in HEB	**7991**	TUSC3	-3.911383	0.981519	0.066459	tumor suppressor candidate 3
UP in HEB	**404203**	SPINK6	-7.979087	0.9930841	0.003963	serine peptidase inhibitor, Kazal type 6
UP in HEB	**3875**	KRT18	-11.75725	1	0.000289	keratin 18
UP in HEB	**26030**	PLEKHG3	-9.280771	0.9844881	0.001608	pleckstrin homology domain containing, family G (with RhoGef domain) member 3
UP in HEB	**56134**	PCDHAC2	-3.265995	0.8681263	0.103953	protocadherin alpha subfamily C, 2
UP in HEB	**23215**	PRRC2C	-1.514199	0.9056483	0.350091	proline-rich coiled-coil 2C
UP in HEB	**5984**	RFC4	-1.281682	0.8988746	0.411316	replication factor C (activator 1) 4, 37kDa
UP in HEB	**94239**	H2AFV	-1.474392	0.9073462	0.359885	H2A histone family, member V
UP in HEB	**8187**	ZNF239	-7.419057	0.9557754	0.005843	zinc finger protein 239
UP in HEB	**5019**	OXCT1	-2.015347	0.939819	0.247355	3-oxoacid CoA transferase 1
UP in HEB	**6493**	SIM2	-3.873606	0.8551478	0.068223	single-minded homolog 2 (Drosophila)
UP in HEB	**114884**	OSBPL10	-2.08293	0.9287162	0.236035	oxysterol binding protein-like 10
UP in HEB	**84461**	NEURL4	-1.078115	0.8738244	0.473647	neuralized homolog 4 (Drosophila)
UP in HEB	**114984**	FLYWCH	-2.727873	0.9595178	0.150948	FLYWCH family member 2
UP in HEB	**3373**	HYAL1	-3.417258	0.9400501	0.093606	hyaluronoglucosaminidase 1
UP in HEB	**23286**	WWC1	-3.90014	0.9777855	0.066979	WW and C2 domain containing 1
UP in HEB	**27141**	CIDEB	-1.076266	0.8137234	0.474255	cell death-inducing DFFA-like effector b
UP in HEB	**9656**	MDC1	-2.833059	0.9626736	0.140334	mediator of DNA-damage checkpoint 1
UP in HEB	**2138**	EYA1	-2.222392	0.9227514	0.214286	eyes absent homolog 1 (Drosophila)
UP in HEB	**64949**	MRPS26	-1.688379	0.9225114	0.310275	mitochondrial ribosomal protein S26
UP in HEB	**112495**	GTF3C6	-1.574984	0.9078262	0.335647	general transcription factor IIIC, polypeptide 6, alpha 35kDa
UP in HEB	**248**	ALPI	-8.573647	0.9536242	0.002625	alkaline phosphatase, intestinal
UP in HEB	**5608**	MAP2K6	-2.608439	0.9423525	0.163977	mitogen-activated protein kinase kinase 6
UP in HEB	**440498**	HSBP1L1	-5.869838	0.9927996	0.0171	heat shock factor binding protein 1-like 1
UP in HEB	**54922**	RASIP1	-7.494078	0.9885149	0.005547	Ras interacting protein 1
UP in HEB	**8743**	TNFSF10	-7.60733	0.9922129	0.005128	tumor necrosis factor (ligand) superfamily, member 10
UP in HEB	**27250**	PDCD4	-1.588844	0.9179334	0.332438	programmed cell death 4 (neoplastic transformation inhibitor)
UP in HEB	**64784**	CRTC3	-1.724798	0.9178356	0.302541	CREB regulated transcription coactivator 3
UP in HEB	**9131**	AIFM1	-1.119166	0.8903942	0.46036	apoptosis-inducing factor, mitochondrion-associated, 1
UP in HEB	**1123**	CHN1	-1.703043	0.9189823	0.307138	chimerin 1
UP in HEB	**150094**	SIK1	-2.212511	0.9417391	0.215758	salt-inducible kinase 1
UP in HEB	**594**	BCKDHB	-1.569398	0.8973812	0.336949	branched chain keto acid dehydrogenase E1, beta polypeptide
UP in HEB	**221002**	RASGEF1	-7.859676	0.9847103	0.004305	RasGEF domain family, member 1A
UP in HEB	**55023**	PHIP	-1.053485	0.8662684	0.481803	pleckstrin homology domain interacting
UP in HEB	**10782**	ZNF274	-1.487987	0.8727221	0.35651	zinc finger protein 274
UP in HEB	**126353**	C19orf21	-8.320048	0.9910573	0.003129	chromosome 19 open reading frame 21
UP in HEB	**4830**	NME1	-1.238098	0.8994969	0.423931	NME/NM23 nucleoside diphosphate kinase 1
UP in HEB	**22**	ABCB7	-1.445423	0.8961011	0.367184	ATP-binding cassette, sub-family B (MDR/TAP), member 7
UP in HEB	**8013**	NR4A3	-1.863138	0.8629794	0.274878	nuclear receptor subfamily 4, group A,

UP in HEB	**2528**	FUT6	-5.690182	0.9748253	0.019368	fucosyltransferase 6 (alpha (1,3) fucosyltransferase)
UP in HEB	**1677**	DFFB	-1.417225	0.8577346	0.374432	DNA fragmentation factor, 40kDa, beta polypeptide (caspase-activated DNase)
UP in HEB	**84662**	GLIS2	-2.986456	0.9592778	0.126179	GLIS family zinc finger 2
UP in HEB	**3757**	KCNH2	-9.793874	0.985937	0.001127	potassium voltage-gated channel, subfamily H (eag-related), member 2
UP in HEB	**26005**	C2CD3	-1.132755	0.8286131	0.456044	C2 calcium-dependent domain containing 3
UP in HEB	**79148**	MMP28	-7.745954	0.900448	0.004658	matrix metallopeptidase 28
UP in HEB	**284612**	SYPL2	-6.436295	0.8387736	0.011547	synaptophysin-like 2
UP in HEB	**2013**	EMP2	-2.967241	0.9648603	0.127871	epithelial membrane protein 2
UP in HEB	**7675**	ZNF121	-1.246674	0.8591036	0.421419	zinc finger protein 121
UP in HEB	**5612**	PRKRIR	-1.072566	0.8763579	0.475472	protein-kinase, interferon-inducible double stranded RNA dependent inhibitor, repressor of (P58 repressor)
UP in HEB	**552889**	ATXN7L3	-1.574811	0.9038171	0.335687	ataxin 7-like 3B
UP in HEB	**8532**	CPZ	-5.444635	0.9762387	0.022962	carboxypeptidase Z
UP in HEB	**3930**	LBR	-2.31085	0.9414191	0.201542	lamin B receptor
UP in HEB	**7020**	TFAP2A	-2.775025	0.9517574	0.146095	transcription factor AP-2 alpha (activating enhancer binding protein 2 alpha)
UP in HEB	**23576**	DDAH1	-1.619367	0.9025637	0.325478	dimethylarginine dimethylaminohydrolase 1
UP in HEB	**5157**	PDGFRL	-9.205386	0.9748609	0.001694	platelet-derived growth factor receptor-like
UP in HEB	**57476**	GRAMD1	-3.373458	0.9669138	0.096491	GRAM domain containing 1B
UP in HEB	**10772**	SRSF10	-1.241393	0.8982346	0.422964	serine/arginine-rich splicing factor 10
UP in HEB	**6165**	RPL35A	-1.237801	0.8996124	0.424019	ribosomal protein L35a
UP in HEB	**388272**	C16orf87	-1.121047	0.8511476	0.45976	chromosome 16 open reading frame 87
UP in HEB	**55504**	TNFRSF19	-2.045394	0.9239426	0.242256	tumor necrosis factor receptor superfamily, member 19
UP in HEB	**114990**	VASN	-1.028591	0.8744555	0.490189	vasorin
UP in HEB	**10085**	EDIL3	-1.605769	0.9161111	0.328561	EGF-like repeats and discoidin I-like
UP in HEB	**163227**	ZNF100	-4.530923	0.8096254	0.043257	zinc finger protein 100
UP in HEB	**2050**	EPHB4	-3.001963	0.9653226	0.12483	EPH receptor B4
UP in HEB	**57673**	BEND3	-2.510015	0.8978079	0.175554	BEN domain containing 3
UP in HEB	**23242**	COBL	-11.43137	0.9976265	0.000362	cordon-bleu WH2 repeat protein
UP in HEB	**150368**	FAM109B	-6.117108	0.9527353	0.014407	family with sequence similarity 109, member
UP in HEB	**51439**	FAM8A1	-1.387583	0.8791669	0.382205	family with sequence similarity 8, member
UP in HEB	**1415**	CRYBB2	-3.723697	0.9568866	0.075693	crystallin, beta B2
UP in HEB	**29968**	PSAT1	-1.158587	0.893941	0.447951	phosphoserine aminotransferase 1
UP in HEB	**83543**	AIF1L	-3.915956	0.97919	0.066249	allograft inflammatory factor 1-like
UP in HEB	**100463486**	MTRNR2L	-2.213434	0.9426014	0.21562	MT-RNR2-like 8
UP in HEB	**163081**	ZNF567	-7.156504	0.8386492	0.007009	zinc finger protein 567
UP in HEB	**4659**	PPP1R12A	-2.226795	0.9403435	0.213633	protein phosphatase 1, regulatory subunit
UP in HEB	**6940**	ZNF354A	-8.78899	0.9619535	0.002261	zinc finger protein 354A
UP in HEB	**114822**	RHPN1	-5.846455	0.9647003	0.01738	rhophilin, Rho GTPase binding protein 1
UP in HEB	**10591**	DNPH1	-5.620085	0.9958842	0.020332	2'-deoxynucleoside 5'-phosphate N-hydrolase
UP in HEB	**100049076**	GUSBP9	-2.48226	0.8250484	0.178964	glucuronidase, beta pseudogene 9
UP in HEB	**124402**	UBALD1	-2.08169	0.9351165	0.236237	UBA-like domain containing 1
UP in HEB	**64146**	PDF	-2.624583	0.9574288	0.162152	peptide deformylase (mitochondrial)
UP in HEB	**1287**	COL4A5	-2.425681	0.9469483	0.186122	collagen, type IV, alpha 5
UP in HEB	**7480**	WNT10B	-4.860052	0.9733941	0.034433	wingless-type MMTV integration site family, member 10B
UP in HEB	**55269**	PSPC1	-1.35398	0.8995235	0.391211	paraspeckle component 1
UP in HEB	**1674**	DES	-6.184167	0.9664604	0.013752	desmin
UP in HEB	**100499405**	LOC100499405	-2.290145	0.9431259	0.204455	uncharacterized LOC100499405
UP in HEB	**51170**	HSD17B11	-2.150552	0.9368677	0.225226	hydroxysteroid (17-beta) dehydrogenase 11
UP in HEB	**9768**	KIAA0101	-2.240869	0.9426547	0.211559	KIAA0101

UP in HEB	**1999**	ELF3	-5.005143	0.9449571	0.031139	E74-like factor 3 (ets domain transcription factor, epithelial-specific)
UP in HEB	**341032**	C11orf53	-6.914883	0.8091454	0.008287	chromosome 11 open reading frame 53
UP in HEB	**54494**	C11orf71	-1.482016	0.8104166	0.357988	chromosome 11 open reading frame 71
UP in HEB	**1992**	SERPINB1	-1.358731	0.9002525	0.389925	serpin peptidase inhibitor, clade B (ovalbumin), member 1
UP in HEB	**1466**	CSRP2	-1.320753	0.8887674	0.400326	cysteine and glycine-rich protein 2
UP in HEB	**8906**	AP1G2	-4.081209	0.9678561	0.059079	adaptor-related protein complex 1, gamma 2 subunit
UP in HEB	**27092**	CACNG4	-7.541742	0.8811225	0.005367	calcium channel, voltage-dependent, gamma subunit 4
UP in HEB	**51203**	NUSAP1	-1.341303	0.9028393	0.394664	nucleolar and spindle associated protein 1
UP in HEB	**54974**	THG1L	-1.100118	0.8726421	0.466478	tRNA-histidine guanylyltransferase 1-like (S. cerevisiae)
UP in HEB	**1407**	CRY1	-1.343044	0.8902164	0.394188	cryptochrome 1 (photolyase-like)
UP in HEB	**605**	BCL7A	-3.367371	0.9309652	0.096899	B-cell CLL/lymphoma 7A
UP in HEB	**729830**	FAM160A	-3.130474	0.9323342	0.114191	family with sequence similarity 160, member
UP in HEB	**84448**	ABLIM2	-6.429616	0.8376536	0.011601	actin binding LIM protein family, member 2
UP in HEB	**146857**	SLFN13	-5.140233	0.8645172	0.028355	schlafen family member 13
UP in HEB	**55749**	CCAR1	-1.08208	0.8878518	0.472347	cell division cycle and apoptosis regulator 1
UP in HEB	**7306**	TYRP1	-8.663558	0.9953509	0.002466	tyrosinase-related protein 1
UP in HEB	**7430**	EZR	-3.603721	0.9807279	0.082257	ezrin
UP in HEB	**4041**	LRP5	-10.53722	0.9949953	0.000673	low density lipoprotein receptor-related
UP in HEB	**4300**	MLLT3	-9.58809	0.9828258	0.001299	myeloid/lymphoid or mixed-lineage leukemia (trithorax homolog, Drosophila); translocated to, 3
UP in HEB	**92421**	CHMP4C	-8.030455	0.989475	0.003825	charged multivesicular body protein 4C
UP in HEB	**2275**	FHL3	-2.70388	0.9519974	0.15348	four and a half LIM domains 3
UP in HEB	**10869**	USP19	-1.721856	0.923747	0.303158	ubiquitin specific peptidase 19
UP in HEB	**3480**	IGF1R	-3.183122	0.9685761	0.110099	insulin-like growth factor 1 receptor
UP in HEB	**8914**	TIMELES	-1.376784	0.8995235	0.385076	timeless circadian clock
UP in HEB	**2264**	FGFR4	-8.090995	0.9839014	0.003667	fibroblast growth factor receptor 4
UP in HEB	**2875**	GPT	-3.236748	0.8724199	0.106082	glutamic-pyruvate transaminase (alanine aminotransferase)
UP in HEB	**58487**	CREBZF	-1.49685	0.8988568	0.354326	CREB/ATF bZIP transcription factor
UP in HEB	**22859**	LPHN1	-2.086961	0.9137376	0.235376	latrophilin 1
UP in HEB	**677805**	SNORA18	-6.878562	0.8038473	0.008499	small nucleolar RNA, H/ACA box 18
UP in HEB	**254427**	PROSER2	-2.994248	0.9512863	0.125499	proline and serine-rich protein 2
UP in HEB	**100188893**	TOMM6	-1.752182	0.9263161	0.296852	translocase of outer mitochondrial membrane 6 homolog (yeast)
UP in HEB	**115572**	FAM46B	-4.14582	0.965287	0.056492	family with sequence similarity 46, member
UP in HEB	**150726**	FBXO41	-2.07131	0.9190357	0.237943	F-box protein 41
UP in HEB	**6418**	SET	-1.013622	0.8874073	0.495301	SET nuclear oncogene
UP in HEB	**728461**	CSAG2	-7.238405	0.8478763	0.006623	CSAG family, member 2
UP in HEB	**60485**	SAV1	-1.07223	0.8689086	0.475583	salvador homolog 1 (Drosophila)
UP in HEB	**9026**	HIP1R	-2.977308	0.9632603	0.126982	huntingtin interacting protein 1 related
UP in HEB	**6653**	SORL1	-8.188177	0.9328231	0.003429	sortilin-related receptor, L(DLR class) A repeats containing
UP in HEB	**10901**	DHRS4	-1.437279	0.8963145	0.369263	dehydrogenase/reductase (SDR family)
UP in HEB	**9727**	RAB11FIP	-1.310812	0.8814337	0.403094	RAB11 family interacting protein 3 (class II)
UP in HEB	**100133091**	LOC100133091	-1.209305	0.8717532	0.432477	uncharacterized LOC100133091
UP in HEB	**5411**	PNN	-1.268739	0.8984479	0.415022	pinin, desmosome associated protein
UP in HEB	**27430**	MAT2B	-1.092772	0.8897097	0.46886	methionine adenosyltransferase II, beta
UP in HEB	**7103**	TSPAN8	-7.235536	0.9675627	0.006636	tetraspanin 8
UP in HEB	**9020**	MAP3K14	-1.071473	0.8431116	0.475833	mitogen-activated protein kinase kinase

UP in HEB	**57622**	LRFN1	-7.204571	0.9547354	0.00678	leucine rich repeat and fibronectin type III domain containing 1
UP in HEB	**51076**	CUTC	-2.073274	0.9382723	0.23762	cutC copper transporter homolog (E. coli)
UP in HEB	**3910**	LAMA4	-1.283338	0.8804114	0.410844	laminin, alpha 4
UP in HEB	**163786**	SASS6	-1.482269	0.8825982	0.357925	spindle assembly 6 homolog (C. elegans)
UP in HEB	**27229**	TUBGCP4	-1.269964	0.8910164	0.41467	tubulin, gamma complex associated protein 4
UP in HEB	**4884**	NPTX1	-10.04348	0.9887105	0.000948	neuronal pentraxin I
UP in HEB	**8412**	BCAR3	-1.080861	0.8793358	0.472747	breast cancer anti-estrogen resistance 3
UP in HEB	**63943**	FKBPL	-1.318946	0.8807047	0.400828	FK506 binding protein like
UP in HEB	**121512**	FGD4	-3.469485	0.8047007	0.090278	FYVE, RhoGEF and PH domain containing
UP in HEB	**161424**	NOP9	-1.168691	0.8820382	0.444825	NOP9 nucleolar protein
UP in HEB	**79088**	ZNF426	-2.426815	0.9016303	0.185976	zinc finger protein 426
UP in HEB	**389903**	CSAG3	-4.192356	0.8953633	0.054698	CSAG family, member 3
UP in HEB	**7690**	ZNF131	-1.705307	0.9207602	0.306656	zinc finger protein 131
UP in HEB	**2968**	GTF2H4	-1.542574	0.9040127	0.343272	general transcription factor IIH, polypeptide 4, 52kDa
UP in HEB	**2975**	GTF3C1	-1.126012	0.8863851	0.45818	general transcription factor IIIC, polypeptide 1, alpha 220kDa
UP in HEB	**163590**	TOR1AIP2	-1.571713	0.8970434	0.336409	torsin A interacting protein 2
UP in HEB	**4691**	NCL	-1.377016	0.9049905	0.385014	nucleolin
UP in HEB	**6623**	SNCG	-5.0187	0.9901239	0.030848	synuclein, gamma (breast cancer-specific protein 1)
UP in HEB	**57799**	RAB40C	-1.847489	0.9155955	0.277876	RAB40C, member RAS oncogene family
UP in HEB	**29901**	SAC3D1	-1.276294	0.8954522	0.412855	SAC3 domain containing 1
UP in HEB	**79957**	PAQR6	-3.740174	0.9583356	0.074833	progestin and adipoQ receptor family
UP in HEB	**8140**	SLC7A5	-4.591692	0.992444	0.041473	solute carrier family 7 (amino acid transporter light chain, L system), member 5
UP in HEB	**375035**	SFT2D2	-1.151353	0.8531922	0.450203	SFT2 domain containing 2
UP in HEB	**517**	ATP5G2	-1.013961	0.8875407	0.495185	ATP synthase, H+ transporting, mitochondrial Fo complex, subunit C2 (subunit 9)
UP in HEB	**4171**	MCM2	-1.162797	0.8939855	0.446646	minichromosome maintenance complex component 2
UP in HEB	**4086**	SMAD1	-1.765098	0.9038527	0.294207	SMAD family member 1
UP in HEB	**2194**	FASN	-2.134101	0.9410191	0.227809	fatty acid synthase
UP in HEB	**1396**	CRIP1	-9.634995	0.9999378	0.001258	cysteine-rich protein 1 (intestinal)
UP in HEB	**11336**	EXOC3	-1.278375	0.8973634	0.41226	exocyst complex component 3
UP in HEB	**27246**	RNF115	-1.278239	0.8911409	0.412298	ring finger protein 115
UP in HEB	**5596**	MAPK4	-10.97752	0.995102	0.000496	mitogen-activated protein kinase 4
UP in HEB	**8581**	LY6D	-5.399659	0.8828826	0.023689	lymphocyte antigen 6 complex, locus D
UP in HEB	**4102**	MAGEA3	-1.954074	0.9266272	0.258086	melanoma antigen family A, 3
UP in HEB	**400506**	KNOP1	-1.50908	0.9016303	0.351335	lysine-rich nucleolar protein 1
UP in HEB	**100507217**	LOC100507217	-1.393329	0.9000658	0.380685	uncharacterized LOC100507217
UP in HEB	**100505738**	LOC100505738	-2.860837	0.9372855	0.137658	uncharacterized LOC100505738
UP in HEB	**114991**	ZNF618	-1.732747	0.8665529	0.300878	zinc finger protein 618
UP in HEB	**60529**	ALX4	-9.798742	0.986017	0.001123	ALX homeobox 4
UP in HEB	**4137**	MAPT	-5.621238	0.9822657	0.020316	microtubule-associated protein tau
UP in HEB	**389840**	MAP3K15	-1.620152	0.807083	0.325301	mitogen-activated protein kinase kinase
UP in HEB	**2146**	EZH2	-1.201595	0.8928921	0.434794	enhancer of zeste homolog 2 (Drosophila)
UP in HEB	**51491**	NOP16	-1.641884	0.9228314	0.320438	NOP16 nucleolar protein
UP in HEB	**54934**	KANSL2	-1.187274	0.887754	0.439132	KAT8 regulatory NSL complex subunit 2
UP in HEB	**5742**	PTGS1	-7.037653	0.972363	0.007611	prostaglandin-endoperoxide synthase 1 (prostaglandin G/H synthase and cyclooxygenase)
UP in HEB	**57801**	HES4	-9.336135	0.9781233	0.001547	hairy and enhancer of split 4 (Drosophila)

UP in HEB	**23250**	ATP11A	-9.448461	0.9802478	0.001431	ATPase, class VI, type 11A
UP in HEB	**81853**	TMEM14B	-1.105468	0.8911053	0.464752	transmembrane protein 14B
UP in HEB	**391356**	PTRHD1	-1.820052	0.9254094	0.283211	peptidyl-tRNA hydrolase domain containing
UP in HEB	**6832**	SUPV3L1	-1.125161	0.8889807	0.458451	suppressor of var1, 3-like 1 (S. cerevisiae)
UP in HEB	**8347**	HIST1H2B	-1.357442	0.8487475	0.390274	histone cluster 1, H2bc
UP in HEB	**64759**	TNS3	-1.626957	0.9188312	0.32377	tensin 3
UP in HEB	**5292**	PIM1	-2.073361	0.9254805	0.237605	pim-1 oncogene
UP in HEB	**171391**	NS3BP	-2.601241	0.9162178	0.164797	NS3BP
UP in HEB	**57646**	USP28	-3.987228	0.9756876	0.063056	ubiquitin specific peptidase 28
UP in HEB	**6934**	TCF7L2	-2.255627	0.9278717	0.209406	transcription factor 7-like 2 (T-cell specific, HMG-box)
UP in HEB	**85415**	RHPN2	-2.400148	0.9091329	0.189445	rhophilin, Rho GTPase binding protein 2
UP in HEB	**89944**	GLB1L2	-8.254241	0.9362988	0.003275	galactosidase, beta 1-like 2
UP in HEB	**3416**	IDE	-1.056034	0.878918	0.480952	insulin-degrading enzyme
UP in HEB	**23462**	HEY1	-3.103885	0.9648426	0.116315	hairy/enhancer-of-split related with YRPW motif 1
UP in HEB	**6536**	SLC6A9	-3.485358	0.9699451	0.08929	solute carrier family 6 (neurotransmitter transporter, glycine), member 9
UP in HEB	**29103**	DNAJC15	-3.410901	0.9668871	0.094019	DnaJ (Hsp40) homolog, subfamily C,
UP in HEB	**10815**	CPLX1	-7.083922	0.924636	0.007371	complexin 1
UP in HEB	**80023**	NRSN2	-2.530108	0.9469927	0.173126	neurensin 2
UP in HEB	**175**	AGA	-1.299002	0.8627038	0.406407	aspartylglucosaminidase
UP in HEB	**4973**	OLR1	-8.964822	0.9682027	0.002001	oxidized low density lipoprotein (lectin-like) receptor 1
UP in HEB	**2683**	B4GALT1	-2.244171	0.9428681	0.211075	UDP-Gal:betaGlcNAc beta 1,4-galactosyltransferase, polypeptide 1
UP in HEB	**7057**	THBS1	-5.879684	0.9947019	0.016984	thrombospondin 1
UP in HEB	**25893**	TRIM58	-4.694525	0.9483261	0.03862	tripartite motif containing 58
UP in HEB	**56997**	ADCK3	-2.329565	0.9431703	0.198944	aarF domain containing kinase 3
UP in HEB	**5836**	PYGL	-3.770612	0.981199	0.073271	phosphorylase, glycogen, liver
UP in HEB	**6229**	RPS24	-1.924678	0.929294	0.263399	ribosomal protein S24
UP in HEB	**11165**	NUDT3	-2.036119	0.9353832	0.243819	nudix (nucleoside diphosphate linked moiety X)-type motif 3
UP in HEB	**1029**	CDKN2A	-14.00825	0.9998489	6.07E-05	cyclin-dependent kinase inhibitor 2A
UP in HEB	**286336**	FAM78A	-3.554102	0.8858339	0.085135	family with sequence similarity 78, member
UP in HEB	**55177**	RMDN3	-1.330342	0.8996124	0.397674	regulator of microtubule dynamics 3
UP in HEB	**79929**	MAP6D1	-2.714464	0.871522	0.152358	MAP6 domain containing 1
UP in HEB	**79918**	SETD6	-1.602104	0.9162266	0.329396	SET domain containing 6
UP in HEB	**25**	ABL1	-1.259348	0.8979946	0.417733	c-abl oncogene 1, non-receptor tyrosine
UP in HEB	**644538**	SMIM10	-2.188297	0.9329831	0.21941	small integral membrane protein 10
UP in HEB	**6654**	SOS1	-1.120282	0.8799403	0.460004	son of sevenless homolog 1 (Drosophila)
UP in HEB	**6839**	SUV39H1	-1.34552	0.8926521	0.393512	suppressor of variegation 3-9 homolog 1 (Drosophila)
UP in HEB	**537**	ATP6AP1	-1.070389	0.8885985	0.47619	ATPase, H+ transporting, lysosomal accessory protein 1
UP in HEB	**6636**	SNRPF	-1.89618	0.9284762	0.268654	small nuclear ribonucleoprotein polypeptide
UP in HEB	**51645**	PPIL1	-1.678103	0.9204402	0.312493	peptidylprolyl isomerase (cyclophilin)-like 1
UP in HEB	**51259**	TMEM216	-6.982613	0.9762743	0.007907	transmembrane protein 216
UP in HEB	**23530**	NNT	-3.607273	0.97983	0.082055	nicotinamide nucleotide transhydrogenase
UP in HEB	**4739**	NEDD9	-9.215128	0.9943197	0.001683	neural precursor cell expressed, developmentally down-regulated 9
UP in HEB	**147**	ADRA1B	-3.456923	0.9488062	0.091067	adrenoceptor alpha 1B
UP in HEB	**1052**	CEBPD	-2.557003	0.9491528	0.169928	CCAAT/enhancer binding protein (C/EBP),
UP in HEB	**25992**	SNED1	-4.997686	0.9519174	0.0313	sushi, nidogen and EGF-like domains 1
UP in HEB	**10906**	TRAFD1	-1.189687	0.8881274	0.438398	TRAF-type zinc finger domain containing 1
UP in HEB	**80150**	ASRGL1	-7.612254	0.888394	0.005111	asparaginase like 1
UP in HEB	**374383**	NCR3LG1	-3.093109	0.9593401	0.117188	natural killer cell cytotoxicity receptor 3

UP in HEB	**11247**	NXPH4	-3.517848	0.9698473	0.087302	neurexophilin 4
UP in HEB	**115650**	TNFRSF13 C	-4.00273	0.8507031	0.062382	tumor necrosis factor receptor superfamily, member 13C
UP in HEB	**2178**	FANCE	-2.163715	0.9220403	0.223181	Fanconi anemia, complementation group E
UP in HEB	**290**	ANPEP	-5.888743	0.9521574	0.016878	alanyl (membrane) aminopeptidase
UP in HEB	**348093**	RBPMS2	-7.151198	0.9423703	0.007035	RNA binding protein with multiple splicing 2
UP in HEB	**129804**	FBLN7	-6.695145	0.97951	0.009651	fibulin 7
UP in HEB	**8131**	NPRL3	-4.310983	0.9866926	0.050381	nitrogen permease regulator-like 3 (S.
UP in HEB	**57217**	TTC7A	-1.206968	0.8865362	0.433178	tetratricopeptide repeat domain 7A
UP in HEB	**400236**	FOXN3-	-2.378512	0.8510409	0.192308	FOXN3 antisense RNA 1
UP in HEB	**10040**	TOM1L1	-1.367601	0.8966345	0.387535	target of myb1 (chicken)-like 1
UP in HEB	**6721**	SREBF2	-1.847672	0.9261738	0.27784	sterol regulatory element binding transcription factor 2
UP in HEB	**7080**	NKX2-1	-9.735274	0.9851637	0.001173	NK2 homeobox 1
UP in HEB	**1303**	COL12A1	-1.617566	0.9193557	0.325885	collagen, type XII, alpha 1
UP in HEB	**91695**	RRP7B	-1.480954	0.8939677	0.358252	ribosomal RNA processing 7 homolog B (S. cerevisiae)
UP in HEB	**7916**	PRRC2A	-1.255775	0.8990524	0.418769	proline-rich coiled-coil 2A
UP in HEB	**30833**	NT5C	-1.279166	0.8979057	0.412034	5', 3'-nucleotidase, cytosolic
UP in HEB	**341**	APOC1	-3.404231	0.9540242	0.094455	apolipoprotein C-I
UP in HEB	**121227**	LRIG3	-2.068349	0.9063417	0.238432	leucine-rich repeats and immunoglobulin-like domains 3
UP in HEB	**55320**	MIS18BP1	-1.476739	0.8847672	0.3593	MIS18 binding protein 1
UP in HEB	**283659**	PRTG	-8.044394	0.9614024	0.003788	protogenin
UP in HEB	**4747**	NEFL	-7.950313	0.9163955	0.004043	neurofilament, light polypeptide
UP in HEB	**83481**	EPPK1	-7.030565	0.9894039	0.007649	epiplakin 1
UP in HEB	**65078**	RTN4R	-6.70044	0.9519263	0.009615	reticulon 4 receptor
UP in HEB	**11124**	FAF1	-11.70347	0.9973154	0.0003	Fas (TNFRSF6) associated factor 1
UP in HEB	**92345**	NAF1	-1.323903	0.8850428	0.399453	nuclear assembly factor 1 ribonucleoprotein
UP in HEB	**93663**	ARHGAP1	-2.599213	0.9552421	0.165028	Rho GTPase activating protein 18
UP in HEB	**7516**	XRCC2	-1.344526	0.8945099	0.393783	X-ray repair complementing defective repair in Chinese hamster cells 2
UP in HEB	**2651**	GCNT2	-1.719601	0.8714331	0.303633	glucosaminyl (N-acetyl) transferase 2, I-branching enzyme (I blood group)
UP in HEB	**163732**	CITED4	-12.17638	0.9983732	0.000216	Cbp/p300-interacting transactivator, with Glu/Asp-rich carboxy-terminal domain, 4
UP in HEB	**27254**	CSDC2	-4.992187	0.9790389	0.03142	cold shock domain containing C2, RNA
UP in HEB	**58516**	FAM60A	-2.214421	0.9395612	0.215473	family with sequence similarity 60, member
UP in HEB	**10846**	PDE10A	-4.111136	0.9578822	0.057866	phosphodiesterase 10A
UP in HEB	**65268**	WNK2	-7.149747	0.9110175	0.007042	WNK lysine deficient protein kinase 2
UP in HEB	**254528**	MEIOB	-8.281614	0.9896972	0.003214	meiosis specific with OB domains
UP in HEB	**649946**	RPL23AP6	-1.718964	0.8123367	0.303767	ribosomal protein L23a pseudogene 64
UP in HEB	**7464**	CORO2A	-3.251251	0.9539531	0.105021	coronin, actin binding protein, 2A
UP in HEB	**8632**	DNAH17	-3.576349	0.8331733	0.083832	dynein, axonemal, heavy chain 17
UP in HEB	**7351**	UCP2	-2.893849	0.9637758	0.134544	uncoupling protein 2 (mitochondrial, proton carrier)
UP in HEB	**26156**	RSL1D1	-1.182642	0.8947944	0.440544	ribosomal L1 domain containing 1
UP in HEB	**80745**	THUMPD	-1.547835	0.8948033	0.342023	THUMP domain containing 2
UP in HEB	**64981**	MRPL34	-1.180744	0.8952477	0.441124	mitochondrial ribosomal protein L34
UP in HEB	**112399**	EGLN3	-8.624247	0.9785144	0.002534	egl nine homolog 3 (C. elegans)
UP in HEB	**6159**	RPL29	-1.473678	0.9076751	0.360063	ribosomal protein L29
UP in HEB	**51385**	ZNF589	-1.698998	0.9031682	0.308	zinc finger protein 589
UP in HEB	**83959**	SLC4A11	-7.841302	0.9077106	0.00436	solute carrier family 4, sodium borate transporter, member 11
UP in HEB	**84950**	PRPF38A	-1.312147	0.8962967	0.402721	PRP38 pre-mRNA processing factor 38 (yeast) domain containing A
UP in HEB	**28969**	BZW2	-1.577087	0.9216847	0.335158	basic leucine zipper and W2 domains 2

UP in HEB	**344595**	LOC34459	-1.048371	0.8688197	0.483514	uncharacterized LOC344595
UP in HEB	**9588**	PRDX6	-1.62986	0.9236137	0.32312	peroxiredoxin 6
UP in HEB	**8898**	MTMR2	-1.10355	0.8822515	0.46537	myotubularin related protein 2
UP in HEB	**25864**	ABHD14A	-1.650302	0.9115331	0.318573	abhydrolase domain containing 14A
UP in HEB	**100507424**	LOC10050 7424	-1.805788	0.9235426	0.286025	uncharacterized LOC100507424
UP in HEB	**400954**	EML6	-2.12266	0.8756023	0.229623	echinoderm microtubule associated protein
UP in HEB	**51005**	AMDHD2	-1.510673	0.8995858	0.350948	amidohydrolase domain containing 2
UP in HEB	**5437**	POLR2H	-1.296615	0.900448	0.40708	polymerase (RNA) II (DNA directed) polypeptide H
UP in HEB	**64901**	RANBP17	-5.531381	0.8745978	0.021622	RAN binding protein 17
UP in HEB	**255231**	MCOLN2	-1.253673	0.8209682	0.419379	mucolipin 2
UP in HEB	**284996**	RNF149	-2.928398	0.9551354	0.13136	ring finger protein 149
UP in HEB	**10216**	PRG4	-4.139551	0.8164969	0.056738	proteoglycan 4
UP in HEB	**6248**	RSC1A1	-1.247164	0.8814337	0.421275	regulatory solute carrier protein, family 1, member 1
UP in HEB	**2058**	EPRS	-1.16323	0.8940921	0.446512	glutamyl-prolyl-tRNA synthetase
UP in HEB	**5859**	QARS	-1.306437	0.9013014	0.404318	glutaminyl-tRNA synthetase
UP in HEB	**28992**	MACROD	-2.738384	0.9567266	0.149853	MACRO domain containing 1
UP in HEB	**84627**	ZNF469	-1.315057	0.8129056	0.40191	zinc finger protein 469
UP in HEB	**9093**	DNAJA3	-1.407541	0.9028304	0.376954	DnaJ (Hsp40) homolog, subfamily A,
UP in HEB	**28999**	KLF15	-6.653442	0.8899852	0.009934	Kruppel-like factor 15
UP in HEB	**3184**	HNRNPD	-1.075328	0.8905808	0.474563	heterogeneous nuclear ribonucleo- protein D (AU-rich element RNA binding protein 1, 37kDa)
UP in HEB	**10695**	CNPY3	-1.099484	0.8848472	0.466683	canopy 3 homolog (zebrafish)
UP in HEB	**5052**	PRDX1	-1.126093	0.8936299	0.458155	peroxiredoxin 1
UP in HEB	**5430**	POLR2A	-1.469253	0.9067773	0.361169	polymerase (RNA) II (DNA directed) polypeptide A, 220kDa
UP in HEB	**3189**	HNRNPH3	-1.003675	0.8858961	0.498728	heterogeneous nuclear ribonucleoprotein H3 (2H9)
UP in HEB	**93622**	LOC93622	-2.007569	0.9270806	0.248692	Morf4 family associated protein 1-like 1 pseudogene
UP in HEB	**388591**	RNF207	-2.974733	0.9474994	0.127208	ring finger protein 207
UP in HEB	**60496**	AASDHPP T	-1.494487	0.9031593	0.354907	aminoadipate-semialdehyde dehydrogenase-phosphopantetheinyl transferase
UP in HEB	**7343**	UBTF	-1.179978	0.8925098	0.441358	upstream binding transcription factor, RNA polymerase I
UP in HEB	**26063**	DECR2	-1.565309	0.9011325	0.337905	2,4-dienoyl CoA reductase 2, peroxisomal
UP in HEB	**1482**	NKX2-5	-9.139232	0.9927107	0.001773	NK2 homeobox 5
UP in HEB	**1066**	CES1	-2.932625	0.9556954	0.130976	carboxylesterase 1
UP in HEB	**378825**	LINC0016	-7.982994	0.9188579	0.003953	long intergenic non-protein coding RNA 162
UP in HEB	**6751**	SSTR1	-7.309855	0.8563657	0.006303	somatostatin receptor 1
UP in HEB	**25974**	MMACHC	-1.82723	0.9075595	0.281805	methylmalonic aciduria (cobalamin deficiency) cblC type, with homocystinuria
UP in HEB	**54517**	PUS7	-1.77404	0.9190535	0.292389	pseudouridylate synthase 7 homolog (S. cerevisiae)
UP in HEB	**9421**	HAND1	-8.271463	0.9374011	0.003236	heart and neural crest derivatives expressed 1
UP in HEB	**440590**	ZYG11A	-4.377305	0.9725852	0.048117	zyg-11 family member A, cell cycle regulator
UP in HEB	**28977**	MRPL42	-1.344089	0.8953544	0.393903	mitochondrial ribosomal protein L42
UP in HEB	**3856**	KRT8	-9.937605	1	0.00102	keratin 8
UP in HEB	**729234**	FAHD2CP	-2.070389	0.8417516	0.238095	fumarylacetoacetate hydrolase domain containing 2C, pseudogene
UP in HEB	**8317**	CDC7	-1.364657	0.8876385	0.388327	cell division cycle 7
UP in HEB	**55753**	OGDHL	-9.615936	0.9832347	0.001274	oxoglutarate dehydrogenase-like
UP in HEB	**3897**	L1CAM	-11.46352	0.9991288	0.000354	L1 cell adhesion molecule
UP in HEB	**2040**	STOM	-1.705936	0.9248138	0.306522	stomatin

UP in HEB	**1031**	CDKN2C	-12.29462	0.9985866	0.000199	cyclin-dependent kinase inhibitor 2C (p18, inhibits CDK4)
UP in HEB	**5455**	POU3F3	-9.935165	0.9875104	0.001021	POU class 3 homeobox 3
UP in HEB	**7263**	TST	-2.175995	0.9375967	0.221289	thiosulfate sulfurtransferase (rhodanese)
UP in HEB	**6188**	RPS3	-1.768248	0.926885	0.293565	ribosomal protein S3
UP in HEB	**55011**	PIH1D1	-1.370442	0.9006703	0.386773	PIH1 domain containing 1
UP in HEB	**440193**	CCDC88C	-7.948367	0.9425836	0.004049	coiled-coil domain containing 88C
UP in HEB	**8487**	GEMIN2	-1.66991	0.894421	0.314273	gem (nuclear organelle) associated protein 2
UP in HEB	**7920**	ABHD16A	-1.106341	0.8796469	0.46447	abhydrolase domain containing 16A
UP in HEB	**6286**	S100P	-12.37965	0.9987199	0.000188	S100 calcium binding protein P
UP in HEB	**57670**	KIAA1549	-1.768857	0.9094352	0.293441	KIAA1549
UP in HEB	**6136**	RPL12	-1.758454	0.926725	0.295565	ribosomal protein L12
UP in HEB	**83690**	CRISPLD1	-3.541515	0.9645937	0.085881	cysteine-rich secretory protein LCCL domain containing 1
UP in HEB	**144501**	KRT80	-1.545351	0.8965634	0.342612	keratin 80
UP in HEB	**7068**	THRB	-1.350639	0.8007183	0.392118	thyroid hormone receptor, beta
UP in HEB	**55692**	LUC7L	-2.190616	0.9392945	0.219058	LUC7-like (S. cerevisiae)
UP in HEB	**10555**	AGPAT2	-10.23512	0.9972354	0.00083	1-acylglycerol-3-phosphate O-acyltransferase
UP in HEB	**10124**	ARL4A	-1.853412	0.9189646	0.276737	ADP-ribosylation factor-like 4A
UP in HEB	**11331**	PHB2	-1.134868	0.8935232	0.455377	prohibitin 2
UP in HEB	**162967**	ZNF320	-6.937815	0.893301	0.008157	zinc finger protein 320
UP in HEB	**29072**	SETD2	-1.10343	0.882696	0.465409	SET domain containing 2
UP in HEB	**100128782**	LINC0047	-1.853499	0.8716643	0.27672	long intergenic non-protein coding RNA 476
UP in HEB	**400550**	FENDRR	-4.016913	0.9579533	0.061772	FOXF1 adjacent non-coding developmental regulatory RNA
UP in HEB	**182**	JAG1	-1.108276	0.8264174	0.463848	jagged 1
UP in HEB	**9631**	NUP155	-1.028857	0.8839227	0.490098	nucleoporin 155kDa
UP in HEB	**9118**	INA	-7.292782	0.9216314	0.006378	internexin neuronal intermediate filament protein, alpha
UP in HEB	**79714**	CCDC51	-1.285703	0.8973279	0.410171	coiled-coil domain containing 51
UP in HEB	**11329**	STK38	-1.929299	0.9234004	0.262557	serine/threonine kinase 38
UP in HEB	**10965**	ACOT2	-1.03688	0.8782957	0.48738	acyl-CoA thioesterase 2
UP in HEB	**250**	ALPP	-11.04894	0.9953775	0.000472	alkaline phosphatase, placental
UP in HEB	**1373**	CPS1	-7.203695	0.9992533	0.006784	carbamoyl-phosphate synthase 1,
UP in HEB	**50861**	STMN3	-2.333367	0.8783402	0.198421	stathmin-like 3
UP in HEB	**54345**	SOX18	-5.971986	0.9225025	0.015931	SRY (sex determining region Y)-box 18
UP in HEB	**100506866**	TTN-AS1	-6.256662	0.9407257	0.013078	TTN antisense RNA 1
UP in HEB	**494115**	RBMXL1	-2.157622	0.9228048	0.224125	RNA binding motif protein, X-linked-like 1
UP in HEB	**9425**	CDYL	-1.744793	0.9202269	0.298377	chromodomain protein, Y-like
UP in HEB	**51028**	VPS36	-1.486205	0.8941099	0.35695	vacuolar protein sorting 36 homolog (S. cerevisiae)
UP in HEB	**5603**	MAPK13	-2.904422	0.8331733	0.133562	mitogen-activated protein kinase 13
UP in HEB	**23657**	SLC7A11	-1.345395	0.8781891	0.393546	solute carrier family 7 (anionic amino acid transporter light chain, xc- system), member 11
UP in HEB	**9767**	PHF16	-2.686501	0.9513663	0.15534	PHD finger protein 16
UP in HEB	**51133**	KCTD3	-1.028852	0.8828915	0.4901	potassium channel tetramerisation domain containing 3
UP in HEB	**79987**	SVEP1	-2.602756	0.9541931	0.164624	sushi, von Willebrand factor type A, EGF and pentraxin domain containing 1
UP in HEB	**113655**	MFSD3	-1.195516	0.8857895	0.43663	major facilitator superfamily domain
UP in HEB	**51760**	SYT17	-8.661778	0.9573488	0.002469	synaptotagmin XVII
UP in HEB	**84940**	CORO6	-2.760911	0.9246093	0.147531	coronin 6
UP in HEB	**780**	DDR1	-2.35287	0.9491173	0.195756	discoidin domain receptor tyrosine kinase 1
UP in HEB	**64787**	EPS8L2	-1.427227	0.8975856	0.371845	EPS8-like 2
UP in HEB	**27436**	EML4	-1.996734	0.938699	0.250567	echinoderm microtubule associated protein
UP in HEB	**79659**	DYNC2H1	-1.322397	0.841316	0.39987	dynein, cytoplasmic 2, heavy chain 1

UP in HEB	**4150**	MAZ	-1.927144	0.9290362	0.262949	MYC-associated zinc finger protein (purine-binding transcription factor)
UP in HEB	**84898**	PLXDC2	-3.64689	0.9143688	0.079832	plexin domain containing 2
UP in HEB	**91156**	IGFN1	-7.043665	0.9777677	0.00758	immunoglobulin-like and fibronectin type III domain containing 1
UP in HEB	**55604**	LRRC16A	-2.27254	0.9350276	0.206965	leucine rich repeat containing 16A
UP in HEB	**55679**	LIMS2	-6.524987	0.9425836	0.010859	LIM and senescent cell antigen-like domains
UP in HEB	**49856**	WRAP73	-1.19341	0.8797714	0.437268	WD repeat containing, antisense to TP73
UP in HEB	**140707**	BRI3BP	-1.514436	0.89659	0.350033	BRI3 binding protein
UP in HEB	**51514**	DTL	-1.263225	0.8929543	0.416612	denticleless E3 ubiquitin protein ligase homolog (Drosophila)
UP in HEB	**440081**	DDX12P	-1.554656	0.8956744	0.34041	DEAD/H (Asp-Glu-Ala-Asp/His) box polypeptide 12, pseudogene
UP in HEB	**118881**	COMTD1	-3.641782	0.9797589	0.080115	catechol-O-methyltransferase domain containing 1
UP in HEB	**55971**	BAIAP2L1	-3.383279	0.9660337	0.095837	BAI1-associated protein 2-like 1
UP in HEB	**1736**	DKC1	-1.167114	0.89507	0.445311	dyskeratosis congenita 1, dyskerin
UP in HEB	**79072**	FASTKD3	-1.765733	0.9210447	0.294077	FAST kinase domains 3
UP in HEB	**3329**	HSPD1	-1.251838	0.8997902	0.419913	heat shock 60kDa protein 1 (chaperonin)
UP in HEB	**55422**	ZNF331	-6.561246	0.9792878	0.010589	zinc finger protein 331
UP in HEB	**643911**	CRNDE	-2.111762	0.9360588	0.231364	colorectal neoplasia differentially expressed (non-protein coding)
UP in HEB	**358**	AQP1	-7.710806	0.9721763	0.004773	aquaporin 1 (Colton blood group)
UP in HEB	**2070**	EYA4	-1.194142	0.888963	0.437046	eyes absent homolog 4 (Drosophila)
UP in HEB	**84182**	FAM188B	-2.126532	0.8994524	0.229008	family with sequence similarity 188, member
UP in HEB	**5947**	RBP1	-2.969194	0.8269508	0.127698	retinol binding protein 1, cellular
UP in HEB	**7275**	TUB	-2.740821	0.9395968	0.1496	tubby homolog (mouse)
UP in HEB	**29964**	PRICKLE4	-1.59728	0.822355	0.330499	prickle homolog 4 (Drosophila)
UP in HEB	**94009**	SERHL	-1.064403	0.8093321	0.47817	serine hydrolase-like
UP in HEB	**51172**	NAGPA	-1.780758	0.9090529	0.291031	N-acetylglucosamine-1-phosphodiester alpha-N-acetylglucosaminidase
UP in HEB	**6204**	RPS10	-1.833017	0.9280851	0.280677	ribosomal protein S10
UP in HEB	**5792**	PTPRF	-9.254185	0.9966309	0.001638	protein tyrosine phosphatase, receptor type, F
UP in HEB	**100129845**	PCOLCE-AS1	-2.219852	0.9249027	0.214663	PCOLCE antisense RNA 1
UP in HEB	**752**	FMNL1	-4.38577	0.9858481	0.047836	formin-like 1
UP in HEB	**10360**	NPM3	-2.047209	0.9402457	0.241952	nucleophosmin/nucleoplasmin 3
UP in HEB	**346689**	KLRG2	-7.186527	0.8422938	0.006865	killer cell lectin-like receptor subfamily G, member 2
UP in HEB	**1894**	ECT2	-1.334654	0.9016303	0.396487	epithelial cell transforming sequence 2
UP in HEB	**57731**	SPTBN4	-2.917251	0.8268708	0.132379	spectrin, beta, non-erythrocytic 4
UP in HEB	**119016**	AGAP4	-1.742791	0.8530322	0.298791	ArfGAP with GTPase domain, ankyrin repeat and PH domain 4
UP in HEB	**4837**	NNMT	-3.915837	0.9841858	0.066255	nicotinamide N-methyltransferase
UP in HEB	**6187**	RPS2	-1.541809	0.9096574	0.343455	ribosomal protein S2
UP in HEB	**10873**	ME3	-6.023333	0.9651804	0.015374	malic enzyme 3, NADP(+)-dependent, mitochondrial
UP in HEB	**55920**	RCC2	-3.209505	0.9727541	0.108104	regulator of chromosome condensation 2
UP in HEB	**3613**	IMPA2	-3.190235	0.9727541	0.109558	inositol(myo)-1(or 4)-monophosphatase 2
UP in HEB	**445**	ASS1	-2.146873	0.9418547	0.225802	argininosuccinate synthase 1
UP in HEB	**6520**	SLC3A2	-1.015673	0.8878074	0.494597	solute carrier family 3 (activators of dibasic and neutral amino acid transport), member 2
UP in HEB	**7678**	ZNF124	-1.391398	0.8359468	0.381195	zinc finger protein 124
UP in HEB	**222484**	LNX2	-1.432959	0.8499387	0.37037	ligand of numb-protein X 2
UP in HEB	**653857**	ACTR3C	-5.255501	0.9132043	0.026178	ARP3 actin-related protein 3 homolog C
UP in HEB	**60678**	EEFSEC	-1.253412	0.8729088	0.419455	eukaryotic elongation factor, selenocysteine-tRNA-specific

UP in HEB	**692086**	SNORD17	-2.238212	0.8249062	0.211949	small nucleolar RNA, C/D box 17
UP in HEB	**7090**	TLE3	-1.639001	0.9163778	0.321079	transducin-like enhancer of split 3 (E(sp1) homolog, Drosophila)
UP in HEB	**4061**	LY6E	-3.126908	0.9694028	0.114474	lymphocyte antigen 6 complex, locus E
UP in HEB	**7727**	ZNF174	-1.274362	0.8602237	0.413408	zinc finger protein 174
UP in HEB	**10196**	PRMT3	-1.642352	0.9183068	0.320334	protein arginine methyltransferase 3
UP in HEB	**5754**	PTK7	-2.574988	0.9383789	0.167823	protein tyrosine kinase 7
UP in HEB	**201294**	UNC13D	-5.455939	0.9878482	0.022782	unc-13 homolog D (C. elegans)
UP in HEB	**57409**	MIF4GD	-1.64448	0.912742	0.319862	MIF4G domain containing
UP in HEB	**163782**	KANK4	-10.45635	0.995902	0.000712	KN motif and ankyrin repeat domains 4
UP in HEB	**3714**	JAG2	-6.920935	0.985697	0.008253	jagged 2
UP in HEB	**56654**	NPDC1	-2.255885	0.9407524	0.209368	neural proliferation, differentiation and
UP in HEB	**9308**	CD83	-2.124796	0.9004214	0.229283	CD83 molecule
UP in HEB	**221914**	GPC2	-3.446886	0.8120966	0.091703	glypican 2
UP in HEB	**65988**	ZNF747	-2.969078	0.934841	0.127708	zinc finger protein 747
UP in HEB	**1410**	CRYAB	-2.048153	0.8810781	0.241793	crystallin, alpha B
UP in HEB	**1045**	CDX2	-7.807355	0.9050616	0.004464	caudal type homeobox 2
UP in HEB	**55839**	CENPN	-1.076711	0.8869006	0.474108	centromere protein N
UP in HEB	**548596**	CKMT1A	-10.3015	0.9911551	0.000792	creatine kinase, mitochondrial 1A
UP in HEB	**2049**	EPHB3	-5.124121	0.9212047	0.028674	EPH receptor B3
UP in HEB	**4241**	MFI2	-1.556967	0.8526944	0.339865	antigen p97 (melanoma associated) identified by monoclonal antibodies 133.2 and 96.5
UP in HEB	**4881**	NPR1	-6.697663	0.8678685	0.009634	natriuretic peptide receptor A/guanylate cyclase A (atrionatriuretic peptide receptor A)
UP in HEB	**1017**	CDK2	-1.01033	0.8854783	0.496433	cyclin-dependent kinase 2
UP in HEB	**27344**	PCSK1N	-9.143808	0.973163	0.001768	proprotein convertase subtilisin/kexin type 1 inhibitor
UP in HEB	**9816**	URB2	-1.755728	0.9139687	0.296124	URB2 ribosome biogenesis 2 homolog (S. cerevisiae)
UP in HEB	**53833**	IL20RB	-4.811228	0.9877771	0.035619	interleukin 20 receptor beta
UP in HEB	**119559**	SFXN4	-1.758959	0.9226892	0.295461	sideroflexin 4
UP in HEB	**150197**	LOC15019	-4.643856	0.8250929	0.04	uncharacterized LOC150197
UP in HEB	**668**	FOXL2	-9.801978	0.9860615	0.00112	forkhead box L2
UP in HEB	**55861**	DBNDD2	-2.622321	0.9558199	0.162406	dysbindin (dystrobrevin binding protein 1) domain containing 2
UP in HEB	**80178**	C16orf59	-1.766114	0.9172667	0.294	chromosome 16 open reading frame 59
UP in HEB	**339983**	NAT8L	-8.720244	0.9728074	0.002371	N-acetyltransferase 8-like (GCN5-related, putative)
UP in HEB	**51303**	FKBP11	-2.189221	0.9382278	0.21927	FK506 binding protein 11, 19 kDa
UP in HEB	**9775**	EIF4A3	-1.103225	0.891292	0.465475	eukaryotic translation initiation factor 4A3
UP in HEB	**79689**	STEAP4	-10.09704	0.9967998	0.000913	STEAP family member 4
UP in HEB	**3177**	SLC29A2	-8.449524	0.9961242	0.00286	solute carrier family 29 (nucleoside transporters), member 2
UP in HEB	**7043**	TGFB3	-2.992329	0.9198446	0.125666	transforming growth factor, beta 3
UP in HEB	**9735**	KNTC1	-1.159699	0.8845538	0.447606	kinetochore associated 1
UP in HEB	**445329**	SULT1A4	-2.480712	0.9490728	0.179156	sulfotransferase family, cytosolic, 1A, phenol-preferring, member 4
UP in HEB	**2766**	GMPR	-2.7913	0.9452593	0.144456	guanosine monophosphate reductase
UP in HEB	**79412**	KREMEN	-3.202215	0.8198837	0.108652	kringle containing transmembrane protein 2
UP in HEB	**123**	PLIN2	-7.674139	0.9952086	0.004896	perilipin 2
UP in HEB	**3164**	NR4A1	-1.307474	0.8791669	0.404028	nuclear receptor subfamily 4, group A,
UP in HEB	**5575**	PRKAR1B	-1.642401	0.9123153	0.320323	protein kinase, cAMP-dependent, regulatory, type I, beta
UP in HEB	**1801**	DPH1	-1.518713	0.9038527	0.348997	DPH1 homolog (S. cerevisiae)
UP in HEB	**3163**	HMOX2	-1.529143	0.9037638	0.346483	heme oxygenase (decycling) 2
UP in HEB	**92196**	DAPL1	-7.549464	0.8817626	0.005338	death associated protein-like 1

UP in HEB	**57019**	CIAPIN1	-1.502896	0.907604	0.352844	cytokine induced apoptosis inhibitor 1
UP in HEB	**7004**	TEAD4	-1.955463	0.9262627	0.257838	TEA domain family member 4
UP in HEB	**5521**	PPP2R2B	-2.076929	0.8779846	0.237018	protein phosphatase 2, regulatory subunit B,
UP in HEB	**7862**	BRPF1	-1.059048	0.8809359	0.479949	bromodomain and PHD finger containing, 1
UP in HEB	**3185**	HNRNPF	-1.838585	0.9281473	0.279596	heterogeneous nuclear ribonucleoprotein F
UP in HEB	**100008587**	RNA5-8S5	-3.303159	0.9729763	0.101309	RNA, 5.8S ribosomal 5
UP in HEB	**5174**	PDZK1	-2.136225	0.8141145	0.227474	PDZ domain containing 1
UP in HEB	**400794**	LOC40079	-1.673063	0.9183068	0.313587	uncharacterized LOC400794
UP in HEB	**3149**	HMGB3	-1.399922	0.9001369	0.37895	high mobility group box 3
UP in HEB	**100302739**	PCNA-	-1.283719	0.9000569	0.410735	PCNA antisense RNA 1
UP in HEB	**55209**	SETD5	-1.194946	0.8933632	0.436803	SET domain containing 5
UP in HEB	**57338**	JPH3	-5.910322	0.8318666	0.016627	junctophilin 3
UP in HEB	**4144**	MAT2A	-1.090017	0.8914342	0.469756	methionine adenosyltransferase II, alpha
UP in HEB	**573**	BAG1	-3.664007	0.9805501	0.07889	BCL2-associated athanogene
UP in HEB	**51222**	ZNF219	-1.722831	0.9102441	0.302954	zinc finger protein 219
UP in HEB	**257000**	TINCR	-4.142472	0.9264494	0.056623	tissue differentiation-inducing non-protein coding RNA
UP in HEB	**3157**	HMGCS1	-2.13814	0.9322542	0.227172	3-hydroxy-3-methylglutaryl-CoA synthase 1 (soluble)
UP in HEB	**6097**	RORC	-3.710847	0.9291073	0.07637	RAR-related orphan receptor C
UP in HEB	**728378**	POTEF	-4.681824	0.8640728	0.038961	POTE ankyrin domain family, member F
UP in HEB	**9467**	SH3BP5	-2.075351	0.9408146	0.237278	SH3-domain binding protein 5 (BTK-
UP in HEB	**7745**	ZKSCAN8	-1.651797	0.9092396	0.318243	zinc finger with KRAB and SCAN domains 8
UP in HEB	**8462**	KLF11	-1.073657	0.8172969	0.475113	Kruppel-like factor 11
UP in HEB	**57524**	CASKIN1	-1.446571	0.8684997	0.366893	CASK interacting protein 1
UP in HEB	**51642**	MRPL48	-1.623695	0.9163155	0.324503	mitochondrial ribosomal protein L48
UP in HEB	**1944**	EFNA3	-1.956057	0.8442761	0.257732	ephrin-A3
UP in HEB	**84166**	NLRC5	-2.574196	0.9478639	0.167915	NLR family, CARD domain containing 5
UP in HEB	**100188947**	LOC10018 8947	-2.192645	0.9109375	0.21875	uncharacterized LOC100188947
UP in HEB	**3217**	HOXB7	-6.927037	0.9771543	0.008218	homeobox B7
UP in HEB	**55746**	NUP133	-1.044876	0.8805714	0.484687	nucleoporin 133kDa
UP in HEB	**84057**	MND1	-1.413173	0.892092	0.375485	meiotic nuclear divisions 1 homolog (S. cerevisiae)
UP in HEB	**5288**	PIK3C2G	-6.741467	0.8369513	0.009346	phosphatidylinositol-4-phosphate 3-kinase, catalytic subunit type 2 gamma
UP in HEB	**55388**	MCM10	-1.08598	0.8689352	0.471072	minichromosome maintenance complex component 10
UP in HEB	**2774**	GNAL	-1.429642	0.8399026	0.371223	guanine nucleotide binding protein (G protein), alpha activating activity polypeptide, olfactory type
UP in HEB	**84172**	POLR1B	-1.645598	0.9160933	0.319614	polymerase (RNA) I polypeptide B, 128kDa
UP in HEB	**10016**	PDCD6	-1.417145	0.9061017	0.374453	programmed cell death 6
UP in HEB	**220108**	FAM124A	-3.755211	0.9625224	0.074057	family with sequence similarity 124A
UP in HEB	**1718**	DHCR24	-2.824892	0.964807	0.141131	24-dehydrocholesterol reductase
UP in HEB	**11187**	PKP3	-5.429069	0.9936797	0.023211	plakophilin 3
UP in HEB	**55266**	TMEM19	-1.714876	0.9173111	0.304629	transmembrane protein 19
UP in HEB	**284751**	LOC28475	-1.79272	0.8633883	0.288627	uncharacterized LOC284751
UP in HEB	**724102**	SNHG4	-2.307735	0.9402546	0.201977	small nucleolar RNA host gene 4 (non-protein coding)
UP in HEB	**51700**	CYB5R2	-5.774567	0.9924707	0.018268	cytochrome b5 reductase 2
UP in HEB	**9088**	PKMYT1	-1.406482	0.9002169	0.37723	protein kinase, membrane associated tyrosine/threonine 1
UP in HEB	**7334**	UBE2N	-1.250834	0.8958078	0.420205	ubiquitin-conjugating enzyme E2N
UP in HEB	**7164**	TPD52L1	-9.791163	0.9964354	0.001129	tumor protein D52-like 1
UP in HEB	**10363**	HMG20A	-1.716464	0.9203513	0.304294	high mobility group 20A

UP in HEB	**100505483**	PRKAG2-AS1	-8.675957	0.9578911	0.002445	PRKAG2 antisense RNA 1
UP in HEB	**284836**	LINC0031	-2.951456	0.8178036	0.129278	long intergenic non-protein coding RNA 319
UP in HEB	**217**	ALDH2	-1.370202	0.8992568	0.386837	aldehyde dehydrogenase 2 family
UP in HEB	**55690**	PACS1	-1.011311	0.882047	0.496095	phosphofurin acidic cluster sorting protein 1
UP in HEB	**472**	ATM	-1.490011	0.8889363	0.35601	ataxia telangiectasia mutated
UP in HEB	**1368**	CPM	-2.584963	0.932752	0.166667	carboxypeptidase M
UP in HEB	**619568**	SNORA4	-8.193115	0.9331075	0.003417	small nucleolar RNA, H/ACA box 4
UP in HEB	**26505**	CNNM3	-2.341824	0.9442726	0.197261	cyclin M3
UP in HEB	**22903**	BTBD3	-4.105978	0.9870838	0.058073	BTB (POZ) domain containing 3
UP in HEB	**1838**	DTNB	-1.082526	0.8567746	0.472201	dystrobrevin, beta
UP in HEB	**8225**	GTPBP6	-1.103343	0.8857984	0.465437	GTP binding protein 6 (putative)
UP in HEB	**6319**	SCD	-3.190447	0.9729497	0.109542	stearoyl-CoA desaturase (delta-9-desaturase)
UP in HEB	**54507**	ADAMTSL4	-5.577757	0.977581	0.020938	ADAMTS-like 4
UP in HEB	**124093**	CCDC78	-1.852925	0.8427649	0.27683	coiled-coil domain containing 78
UP in HEB	**80781**	COL18A1	-5.987189	0.9908351	0.015764	collagen, type XVIII, alpha 1
UP in HEB	**4998**	ORC1	-1.827205	0.9227159	0.28181	origin recognition complex, subunit 1
UP in HEB	**9928**	KIF14	-1.138605	0.868553	0.454198	kinesin family member 14
UP in HEB	**6066**	RNU2-1	-1.78316	0.85417	0.290546	RNA, U2 small nuclear 1
UP in HEB	**202243**	CCDC125	-1.283832	0.8411826	0.410703	coiled-coil domain containing 125
UP in HEB	**63895**	PIEZO2	-10.87549	0.9946308	0.000532	piezo-type mechanosensitive ion channel component 2
UP in HEB	**143458**	LDLRAD3	-1.248047	0.8745889	0.421018	low density lipoprotein receptor class A domain containing 3
UP in HEB	**144571**	A2M-AS1	-5.958069	0.8105143	0.016086	A2M antisense RNA 1
UP in HEB	**7169**	TPM2	-3.658733	0.9809145	0.079179	tropomyosin 2 (beta)
UP in HEB	**4439**	MSH5	-2.062674	0.9200046	0.239372	mutS homolog 5 (E. coli)
UP in HEB	**10301**	DLEU1	-1.630821	0.9138976	0.322904	deleted in lymphocytic leukemia 1 (non-protein coding)
UP in HEB	**30817**	EMR2	-3.847531	0.8709798	0.069467	egf-like module containing, mucin-like, hormone receptor-like 2
UP in HEB	**7019**	TFAM	-2.113052	0.9386101	0.231157	transcription factor A, mitochondrial
UP in HEB	**55967**	NDUFA12	-1.313453	0.900128	0.402357	NADH dehydrogenase (ubiquinone) 1 alpha subcomplex, 12
UP in HEB	**387718**	TEX36	-7.312883	0.8563657	0.006289	testis expressed 36
UP in HEB	**21**	ABCA3	-3.504023	0.9737408	0.088142	ATP-binding cassette, sub-family A (ABC1), member 3
UP in HEB	**414189**	AGAP6	-1.229672	0.8873807	0.426414	ArfGAP with GTPase domain, ankyrin repeat and PH domain 6
UP in HEB	**151507**	MSL3P1	-1.587834	0.902537	0.332671	male-specific lethal 3 homolog (Drosophila) pseudogene 1
UP in HEB	**8564**	KMO	-2.474112	0.9200935	0.179978	kynurenine 3-monooxygenase (kynurenine 3-hydroxylase)
UP in HEB	**2926**	GRSF1	-1.387871	0.8982257	0.382128	G-rich RNA sequence binding factor 1
UP in HEB	**219790**	RTKN2	-2.872724	0.9217025	0.136529	rhotekin 2
UP in HEB	**57662**	CAMSAP3	-8.514714	0.9669405	0.002734	calmodulin regulated spectrin-associated protein family, member 3
UP in HEB	**133584**	EGFLAM	-7.708394	0.9916884	0.004781	EGF-like, fibronectin type III and laminin G domains
UP in HEB	**23170**	TTLL12	-1.813546	0.9250093	0.284491	tubulin tyrosine ligase-like family, member
UP in HEB	**144100**	PLEKHA7	-6.725975	0.9148132	0.009447	pleckstrin homology domain containing, family A member 7
UP in HEB	**168544**	ZNF467	-6.979459	0.9847814	0.007925	zinc finger protein 467
UP in HEB	**7784**	ZP3	-3.679207	0.9692161	0.078064	zona pellucida glycoprotein 3 (sperm
UP in HEB	**23371**	TENC1	-1.809471	0.9226181	0.285296	tensin like C1 domain containing phosphatase (tensin 2)

UP in HEB	**55322**	C5orf22	-1.371914	0.89587	0.386378	chromosome 5 open reading frame 22
UP in HEB	**2110**	ETFDH	-1.556393	0.8966078	0.34	electron-transferring-flavoprotein
UP in HEB	**654364**	NME1-NME2	-1.335972	0.9003325	0.396125	NME1-NME2 readthrough
UP in HEB	**25960**	GPR124	-4.977087	0.9850925	0.03175	G protein-coupled receptor 124
UP in HEB	**729438**	GATSL2	-1.693939	0.8894341	0.309082	GATS protein-like 2
UP in HEB	**201725**	C4orf46	-1.895722	0.9138532	0.268739	chromosome 4 open reading frame 46
UP in HEB	**84665**	MYPN	-3.898749	0.9794033	0.067044	myopalladin
UP in HEB	**54894**	RNF43	-3.337303	0.9217469	0.09894	ring finger protein 43
UP in HEB	**56919**	DHX33	-1.311651	0.8955322	0.40286	DEAH (Asp-Glu-Ala-His) box polypeptide
UP in HEB	**1181**	CLCN2	-1.141027	0.8381069	0.453437	chloride channel, voltage-sensitive 2
UP in HEB	**93100**	NAPRT1	-2.403878	0.943357	0.188956	nicotinate phosphoribosyltransferase domain containing 1
UP in HEB	**59345**	GNB4	-1.433481	0.9010436	0.370237	guanine nucleotide binding protein (G protein), beta polypeptide 4
UP in HEB	**9905**	SGSM2	-1.613399	0.9179423	0.326827	small G protein signaling modulator 2
UP in HEB	**1388**	ATF6B	-1.680516	0.9223603	0.311971	activating transcription factor 6 beta
UP in HEB	**2202**	EFEMP1	-4.920226	0.9935908	0.033027	EGF containing fibulin-like extracellular matrix protein 1
UP in HEB	**23658**	LSM5	-1.284704	0.8954611	0.410455	LSM5 homolog, U6 small nuclear RNA associated (S. cerevisiae)
UP in HEB	**2762**	GMDS	-1.373172	0.8948477	0.386042	GDP-mannose 4,6-dehydratase
UP in HEB	**90871**	C9orf123	-1.747729	0.9175423	0.29777	chromosome 9 open reading frame 123
UP in HEB	**219738**	C10orf35	-6.929492	0.9818035	0.008204	chromosome 10 open reading frame 35
UP in HEB	**7139**	TNNT2	-6.142339	0.9181734	0.014157	troponin T type 2 (cardiac)
UP in HEB	**790**	CAD	-1.417054	0.9038438	0.374476	carbamoyl-phosphate synthetase 2, aspartate transcarbamylase, and dihydroorotase
UP in HEB	**23138**	N4BP3	-9.663558	0.9840436	0.001233	NEDD4 binding protein 3
UP in HEB	**7832**	BTG2	-2.08092	0.9142265	0.236364	BTG family, member 2
UP in HEB	**203413**	CXorf61	-6.877486	0.9807545	0.008505	chromosome X open reading frame 61
UP in HEB	**293**	SLC25A6	-3.48653	0.9785855	0.089217	solute carrier family 25 (mitochondrial carrier; adenine nucleotide translocator), member 6
UP in HEB	**282997**	PDCD4-	-2.397915	0.8814159	0.189739	PDCD4 antisense RNA 1
UP in HEB	**10232**	MSLN	-10.70594	0.9993511	0.000599	mesothelin
UP in HEB	**494143**	CHAC2	-2.362051	0.9465216	0.194514	ChaC, cation transport regulator homolog 2 (E. coli)
UP in HEB	**10788**	IQGAP2	-1.885667	0.9022615	0.270619	IQ motif containing GTPase activating
UP in HEB	**51022**	GLRX2	-1.269602	0.8926432	0.414774	glutaredoxin 2
UP in HEB	**4303**	FOXO4	-1.466284	0.8884029	0.361913	forkhead box O4
UP in HEB	**283078**	MKX	-4.773942	0.9400501	0.036551	mohawk homeobox
UP in HEB	**2947**	GSTM3	-2.531311	0.9393212	0.172981	glutathione S-transferase mu 3 (brain)
UP in HEB	**4841**	NONO	-1.106336	0.8925187	0.464472	non-POU domain containing, octamer-
UP in HEB	**55572**	FOXRED1	-1.275617	0.8947855	0.413049	FAD-dependent oxidoreductase domain containing 1
UP in HEB	**9317**	PTER	-5.037951	0.9448682	0.030439	phosphotriesterase related
UP in HEB	**63922**	CHTF18	-1.922554	0.9206091	0.263787	CTF18, chromosome transmission fidelity factor 18 homolog (S. cerevisiae)
UP in HEB	**9532**	BAG2	-1.976876	0.9274539	0.254039	BCL2-associated athanogene 2
UP in HEB	**8777**	MPDZ	-1.232206	0.871522	0.425666	multiple PDZ domain protein
UP in HEB	**79888**	LPCAT1	-1.326675	0.9017548	0.398686	lysophosphatidylcholine acyltransferase 1
UP in HEB	**25764**	HYPK	-1.051631	0.8871584	0.482422	huntingtin interacting protein K
UP in HEB	**345643**	MCIDAS	-8.037089	0.9227514	0.003807	multiciliate differentiation and DNA synthesis associated cell cycle protein
UP in HEB	**55636**	CHD7	-1.615041	0.8913809	0.326456	chromodomain helicase DNA binding protein
UP in HEB	**55603**	FAM46A	-2.60552	0.9556954	0.164309	family with sequence similarity 46, member
UP in HEB	**55715**	DOK4	-2.410713	0.9453304	0.188063	docking protein 4

UP in HEB	**134429**	STARD4	-1.180102	0.8732377	0.44132	StAR-related lipid transfer (START) domain containing 4
UP in HEB	**7181**	NR2C1	-1.138102	0.8652461	0.454357	nuclear receptor subfamily 2, group C,
UP in HEB	**5579**	PRKCB	-10.62388	0.9964087	0.000634	protein kinase C, beta
UP in HEB	**80233**	C17orf70	-1.481141	0.8939055	0.358205	chromosome 17 open reading frame 70
UP in HEB	**728609**	SDHAP3	-2.559872	0.9269472	0.169591	succinate dehydrogenase complex, subunit A, flavoprotein pseudogene 3
UP in HEB	**770**	CA11	-2.076037	0.9142265	0.237165	carbonic anhydrase XI
UP in HEB	**79844**	ZDHHC11	-3.518467	0.9335253	0.087264	zinc finger, DHHC-type containing 11
UP in HEB	**58496**	LY6G5B	-1.52434	0.8956478	0.347638	lymphocyte antigen 6 complex, locus G5B
UP in HEB	**55170**	PRMT6	-3.236574	0.967296	0.106095	protein arginine methyltransferase 6
UP in HEB	**10180**	RBM6	-1.560683	0.9062706	0.338991	RNA binding motif protein 6
UP in HEB	**80741**	LY6G5C	-8.046215	0.9229826	0.003783	lymphocyte antigen 6 complex, locus G5C
UP in HEB	**163071**	ZNF114	-1.586523	0.8198837	0.332973	zinc finger protein 114
UP in HEB	**100507118**	LOC100507118	-1.269348	0.8844027	0.414847	uncharacterized LOC100507118
UP in HEB	**10084**	PQBP1	-1.000345	0.885025	0.49988	polyglutamine binding protein 1
UP in HEB	**7433**	VIPR1	-6.882643	0.8038473	0.008475	vasoactive intestinal peptide receptor 1
UP in HEB	**5190**	PEX6	-1.158888	0.8039273	0.447858	peroxisomal biogenesis factor 6
UP in HEB	**84312**	BRMS1L	-1.579068	0.8844383	0.334698	breast cancer metastasis-suppressor 1-like
UP in HEB	**64425**	POLR1E	-1.276025	0.8963056	0.412932	polymerase (RNA) I polypeptide E, 53kDa
UP in HEB	**127602**	DNAH14	-8.062496	0.9240493	0.003741	dynein, axonemal, heavy chain 14
UP in HEB	**140838**	NANP	-1.896164	0.8518854	0.268657	N-acetylneuraminic acid phosphatase
UP in HEB	**23761**	PISD	-1.190797	0.891532	0.438061	phosphatidylserine decarboxylase
UP in HEB	**26054**	SENP6	-1.405284	0.8910875	0.377544	SUMO1/sentrin specific peptidase 6
UP in HEB	**1620**	DBC1	-5.708739	0.8662773	0.01912	deleted in bladder cancer 1
UP in HEB	**145853**	C15orf61	-1.092526	0.845734	0.46894	chromosome 15 open reading frame 61
UP in HEB	**6272**	SORT1	-3.67985	0.976621	0.078029	sortilin 1
UP in HEB	**6164**	RPL34	-1.277238	0.9007058	0.412585	ribosomal protein L34
UP in HEB	**100287042**	LOC100287042	-2.07287	0.9038349	0.237686	uncharacterized LOC100287042
UP in HEB	**26256**	CABYR	-1.058716	0.8024072	0.480059	calcium binding tyrosine-(Y)-phosphorylation regulated
UP in HEB	**100289019**	LOC100289019	-1.793247	0.8633883	0.288522	uncharacterized LOC100289019
UP in HEB	**65250**	C5orf42	-1.590703	0.8962167	0.33201	chromosome 5 open reading frame 42
UP in HEB	**6428**	SRSF3	-1.091806	0.8909453	0.469174	serine/arginine-rich splicing factor 3
UP in HEB	**57007**	CXCR7	-7.047605	0.9811457	0.007559	chemokine (C-X-C motif) receptor 7
UP in HEB	**29116**	MYLIP	-2.74135	0.9218358	0.149545	myosin regulatory light chain interacting
UP in HEB	**64434**	NOM1	-1.290077	0.8920565	0.408929	nucleolar protein with MIF4G domain 1
UP in HEB	**7923**	HSD17B8	-1.307455	0.8264085	0.404033	hydroxysteroid (17-beta) dehydrogenase 8
UP in HEB	**1438**	CSF2RA	-4.319707	0.9485217	0.050077	colony stimulating factor 2 receptor, alpha, low-affinity (granulocyte-macrophage)
UP in HEB	**84159**	ARID5B	-2.167236	0.9383167	0.222637	AT rich interactive domain 5B (MRF1-like)
UP in HEB	**51083**	GAL	-12.84451	0.9992889	0.000136	galanin/GMAP prepropeptide
UP in HEB	**728927**	ZNF736	-4.220729	0.8678774	0.053633	zinc finger protein 736
UP in HEB	**57591**	MKL1	-1.125003	0.8814426	0.458501	megakaryoblastic leukemia (translocation) 1
UP in HEB	**3696**	ITGB8	-3.783155	0.959509	0.072637	integrin, beta 8
UP in HEB	**574407**	C1orf145	-6.954196	0.8137678	0.008065	chromosome 1 open reading frame 145
UP in HEB	**1349**	COX7B	-1.307999	0.9016214	0.403881	cytochrome c oxidase subunit VIIb
UP in HEB	**4795**	NFKBIL1	-1.153064	0.8864206	0.449669	nuclear factor of kappa light polypeptide gene enhancer in B-cells inhibitor-like 1
UP in HEB	**4493**	MT1E	-2.767176	0.9603268	0.146892	metallothionein 1E
UP in HEB	**55215**	FANCI	-1.015128	0.8840383	0.494784	Fanconi anemia, complementation group I
UP in HEB	**56241**	SUSD2	-8.565468	0.9935374	0.00264	sushi domain containing 2
UP in HEB	**633**	BGN	-7.657562	0.998151	0.004953	biglycan
UP in HEB	**134492**	NUDCD2	-1.79484	0.9259694	0.288204	NudC domain containing 2

UP in HEB	**64795**	RMND5A	-2.14112	0.9334276	0.226704	required for meiotic nuclear division 5 homolog A (S. cerevisiae)
UP in HEB	**23204**	ARL6IP1	-1.328898	0.9032926	0.398072	ADP-ribosylation factor-like 6 interacting protein 1
UP in HEB	**79134**	TMEM185	-1.194494	0.8369069	0.43694	transmembrane protein 185B
UP in HEB	**727851**	RGPD8	-4.096276	0.9092929	0.058465	RANBP2-like and GRIP domain containing 8
UP in HEB	**84231**	TRAF7	-1.363289	0.9019148	0.388695	TNF receptor-associated factor 7, E3 ubiquitin protein ligase
UP in HEB	**10217**	CTDSPL	-2.294143	0.9297385	0.203889	CTD (carboxy-terminal domain, RNA polymerase II, polypeptide A) small phosphatase-like
UP in HEB	**9615**	GDA	-9.795499	0.9859637	0.001125	guanine deaminase
UP in HEB	**728404**	AGAP8	-1.055166	0.847503	0.481242	ArfGAP with GTPase domain, ankyrin repeat and PH domain 8
UP in HEB	**55114**	ARHGAP1	-1.076947	0.8867317	0.474031	Rho GTPase activating protein 17
UP in HEB	**5802**	PTPRS	-4.267409	0.9831991	0.051926	protein tyrosine phosphatase, receptor type, S
UP in HEB	**9368**	SLC9A3R1	-2.170157	0.9404502	0.222187	solute carrier family 9, subfamily A (NHE3, cation proton antiporter 3), member 3 regulator 1
UP in HEB	**51450**	PRRX2	-4.992466	0.9461927	0.031414	paired related homeobox 2
UP in HEB	**27293**	SMPDL3B	-5.229655	0.9247604	0.026651	sphingomyelin phosphodiesterase, acid-like
UP in HEB	**389432**	SAMD5	-2.899527	0.9405835	0.134016	sterile alpha motif domain containing 5
UP in HEB	**6860**	SYT4	-7.552029	0.8824382	0.005329	synaptotagmin IV
UP in HEB	**6194**	RPS6	-1.600209	0.923267	0.329829	ribosomal protein S6
UP in HEB	**5160**	PDHA1	-1.373508	0.9025459	0.385952	pyruvate dehydrogenase (lipoamide) alpha 1
UP in HEB	**3087**	HHEX	-8.617336	0.9863815	0.002546	hematopoietically expressed homeobox
UP in HEB	**6208**	RPS14	-1.296351	0.9014614	0.407155	ribosomal protein S14
UP in HEB	**1612**	DAPK1	-5.41555	0.9330453	0.023429	death-associated protein kinase 1
UP in HEB	**25821**	MTO1	-1.728535	0.9145999	0.301758	mitochondrial translation optimization 1 homolog (S. cerevisiae)
UP in HEB	**10528**	NOP56	-1.589594	0.9225736	0.332265	NOP56 ribonucleoprotein
UP in HEB	**4778**	NFE2	-4.366021	0.8755134	0.048495	nuclear factor (erythroid-derived 2), 45kDa
UP in HEB	**10902**	BRD8	-1.463407	0.9046349	0.362636	bromodomain containing 8
UP in HEB	**9340**	GLP2R	-7.108524	0.83264	0.007246	glucagon-like peptide 2 receptor
UP in HEB	**22796**	COG2	-1.160315	0.8823315	0.447415	component of oligomeric golgi complex 2
UP in HEB	**10299**	6-Mar	-1.76452	0.9242271	0.294325	membrane-associated ring finger (C3HC4) 6, E3 ubiquitin protein ligase
UP in HEB	**57713**	SFMBT2	-1.148533	0.8343556	0.451084	Scm-like with four mbt domains 2
UP in HEB	**6622**	SNCA	-3.432698	0.9640159	0.092609	synuclein, alpha (non A4 component of amyloid precursor)
UP in HEB	**8427**	ZNF282	-1.188787	0.8884829	0.438672	zinc finger protein 282
UP in HEB	**84264**	HAGHL	-1.563222	0.9044038	0.338395	hydroxyacylglutathione hydrolase-like
UP in HEB	**84331**	FAM195A	-2.75952	0.9571533	0.147673	family with sequence similarity 195, member
UP in HEB	**7086**	TKT	-1.564353	0.909933	0.338129	transketolase
UP in HEB	**4091**	SMAD6	-2.903469	0.9595001	0.13365	SMAD family member 6
UP in HEB	**54914**	FOCAD	-8.389862	0.9828969	0.002981	focadhesin
UP in HEB	**79774**	GRTP1	-3.797455	0.940139	0.07192	growth hormone regulated TBC protein 1
UP in HEB	**10715**	CERS1	-5.848474	0.9533753	0.017355	ceramide synthase 1
UP in HEB	**10572**	SIVA1	-1.038894	0.8880829	0.4867	SIVA1, apoptosis-inducing factor
UP in HEB	**4690**	NCK1	-1.049299	0.8822959	0.483203	NCK adaptor protein 1
UP in HEB	**7428**	VHL	-1.363157	0.9008036	0.388731	von Hippel-Lindau tumor suppressor, E3 ubiquitin protein ligase
UP in HEB	**100288181**	LOC100288181	-4.279047	0.9503085	0.051508	uncharacterized LOC100288181
UP in HEB	**9886**	RHOBTB1	-2.829233	0.9604246	0.140707	Rho-related BTB domain containing 1
UP in HEB	**1797**	DOM3Z	-2.02369	0.9338543	0.245928	dom-3 homolog Z (C. elegans)
UP in HEB	**4037**	LRP3	-7.47032	0.8732199	0.005639	low density lipoprotein receptor-related

UP in HEB	**50512**	PODXL2	-4.011394	0.9846125	0.062008	podocalyxin-like 2
UP in HEB	**54556**	ING3	-1.300504	0.8760912	0.405984	inhibitor of growth family, member 3
UP in HEB	**9672**	SDC3	-5.274883	0.9779455	0.025829	syndecan 3
UP in HEB	**54536**	EXOC6	-3.566563	0.9619535	0.084403	exocyst complex component 6
UP in HEB	**84733**	CBX2	-5.222269	0.9916884	0.026788	chromobox homolog 2
UP in HEB	**56475**	RPRM	-9.685917	0.9843903	0.001214	reprimo, TP53 dependent G2 arrest mediator candidate
UP in HEB	**23218**	NBEAL2	-2.708921	0.9553665	0.152944	neurobeachin-like 2
UP in HEB	**9718**	ECE2	-2.268604	0.9378456	0.207531	endothelin converting enzyme 2
UP in HEB	**2542**	SLC37A4	-2.386887	0.945366	0.191195	solute carrier family 37 (glucose-6-phosphate transporter), member 4
UP in HEB	**6001**	RGS10	-1.233017	0.8846694	0.425427	regulator of G-protein signaling 10
UP in HEB	**25845**	PP7080	-1.560849	0.9024392	0.338952	uncharacterized LOC25845
UP in HEB	**10439**	OLFM1	-8.511753	0.975892	0.00274	olfactomedin 1
UP in HEB	**22934**	RPIA	-1.107626	0.8751756	0.464057	ribose 5-phosphate isomerase A
UP in HEB	**2261**	FGFR3	-7.72792	0.9292229	0.004717	fibroblast growth factor receptor 3
UP in HEB	**11033**	ADAP1	-5.44922	0.9546731	0.022889	ArfGAP with dual PH domains 1
UP in HEB	**6324**	SCN1B	-4.763015	0.9630469	0.036829	sodium channel, voltage-gated, type I, beta subunit
UP in HEB	**728053**	BMS1P1	-1.846921	0.9066084	0.277985	BMS1 pseudogene 1
UP in HEB	**11322**	TMC6	-8.696968	0.9585845	0.00241	transmembrane channel-like 6
UP in HEB	**55790**	CSGALNACT1	-1.977318	0.9206091	0.253962	chondroitin sulfate N-acetylgalactosaminyltransferase 1
UP in HEB	**3816**	KLK1	-7.146357	0.8375469	0.007059	kallikrein 1
UP in HEB	**2395**	FXN	-2.095401	0.9260405	0.234003	frataxin
UP in HEB	**10347**	ABCA7	-1.691723	0.8931054	0.309557	ATP-binding cassette, sub-family A (ABC1), member 7
UP in HEB	**783**	CACNB2	-3.694023	0.8778779	0.077266	calcium channel, voltage-dependent, beta 2 subunit
UP in HEB	**1152**	CKB	-6.948024	0.9990133	0.008099	creatine kinase, brain
UP in HEB	**26521**	TIMM8B	-2.320317	0.9486728	0.200224	translocase of inner mitochondrial membrane 8 homolog B (yeast)
UP in HEB	**3615**	IMPDH2	-1.394155	0.9051861	0.380468	IMP (inosine 5'-monophosphate) dehydrogenase 2
UP in HEB	**101060200**	ZNF891	-1.947259	0.8123633	0.259309	zinc finger protein 891
UP in HEB	**23224**	SYNE2	-1.175487	0.8553434	0.442734	spectrin repeat containing, nuclear envelope
UP in HEB	**55726**	ASUN	-1.000988	0.8816559	0.499658	asunder, spermatogenesis regulator
UP in HEB	**10272**	FSTL3	-3.386175	0.9700251	0.095644	follistatin-like 3 (secreted glycoprotein)
UP in HEB	**3911**	LAMA5	-1.903158	0.9273739	0.267357	laminin, alpha 5
UP in HEB	**2950**	GSTP1	-1.270817	0.9002702	0.414425	glutathione S-transferase pi 1
UP in HEB	**8751**	ADAM15	-1.914722	0.9284317	0.265223	ADAM metallopeptidase domain 15
UP in HEB	**64983**	MRPL32	-1.293603	0.8997547	0.407931	mitochondrial ribosomal protein L32
UP in HEB	**90861**	HN1L	-1.912849	0.927285	0.265568	hematological and neurological expressed 1-
UP in HEB	**8612**	PPAP2C	-1.082853	0.8867673	0.472094	phosphatidic acid phosphatase type 2C
UP in HEB	**54460**	MRPS21	-2.061247	0.9406635	0.239609	mitochondrial ribosomal protein S21
UP in HEB	**64097**	EPB41L4A	-3.102021	0.9443615	0.116466	erythrocyte membrane protein band 4.1 like
UP in HEB	**144983**	HNRNPA1L2	-1.122974	0.8279908	0.459146	heterogeneous nuclear ribonucleoprotein A1-like 2
UP in HEB	**4363**	ABCC1	-1.642274	0.9210625	0.320351	ATP-binding cassette, sub-family C (CFTR/MRP), member 1
UP in HEB	**55349**	CHDH	-6.97728	0.8165324	0.007937	choline dehydrogenase
UP in HEB	**134147**	CMBL	-3.295272	0.9703895	0.101865	carboxymethylenebutenolidase homolog (Pseudomonas)
UP in HEB	**100462983**	MTRNR2L	-1.809122	0.9267872	0.285365	MT-RNR2-like 3
UP in HEB	**4155**	MBP	-5.573991	0.9659893	0.020992	myelin basic protein
UP in HEB	**114908**	TMEM123	-1.550213	0.908004	0.34146	transmembrane protein 123
UP in HEB	**10609**	LEPREL4	-1.029906	0.8794691	0.489742	leprecan-like 4

UP in HEB	**9789**	SPCS2	-1.542708	0.9057994	0.343241	signal peptidase complex subunit 2 homolog (S. cerevisiae)
UP in HEB	**646962**	HRCT1	-2.75536	0.9387434	0.1481	histidine rich carboxyl terminus 1
UP in HEB	**100190986**	LOC100190986	-1.003444	0.8705264	0.498808	uncharacterized LOC100190986
UP in HEB	**84838**	ZNF496	-1.243139	0.872411	0.422453	zinc finger protein 496
UP in HEB	**29990**	PILRB	-1.782803	0.9238804	0.290618	paired immunoglobin-like type 2 receptor
UP in HEB	**283298**	OLFML1	-5.857981	0.9336942	0.017241	olfactomedin-like 1
UP in HEB	**100128881**	LOC100128881	-4.291296	0.9820435	0.051073	uncharacterized LOC100128881
UP in HEB	**9132**	KCNQ4	-7.431846	0.8696375	0.005792	potassium voltage-gated channel, KQT-like subfamily, member 4
UP in HEB	**90850**	ZNF598	-1.699944	0.9221114	0.307798	zinc finger protein 598
UP in HEB	**55584**	CHRNA9	-1.804194	0.9207958	0.286341	cholinergic receptor, nicotinic, alpha 9
UP in HEB	**9569**	GTF2IRD1	-1.517518	0.9035504	0.349286	GTF2I repeat domain containing 1
UP in HEB	**55591**	VEZT	-1.013305	0.8587125	0.49541	vezatin, adherens junctions transmembrane protein
UP in HEB	**101243544**	CSB-	-1.243733	0.8536544	0.422278	Cockayne syndrome B-piggyBac fusion
UP in HEB	**6389**	SDHA	-1.619618	0.923507	0.325422	succinate dehydrogenase complex, subunit A, flavoprotein (Fp)
UP in HEB	**79817**	MOB3B	-9.250298	0.9881949	0.001642	MOB kinase activator 3B
UP in HEB	**91433**	RCCD1	-1.957694	0.9234181	0.25744	RCC1 domain containing 1
UP in HEB	**1915**	EEF1A1	-1.035681	0.8889007	0.487786	eukaryotic translation elongation factor 1
UP in HEB	**2906**	GRIN2D	-7.085694	0.9250004	0.007362	glutamate receptor, ionotropic, N-methyl D-aspartate 2D
UP in HEB	**388564**	TMEM238	-4.704261	0.9084573	0.03836	transmembrane protein 238
UP in HEB	**441376**	AARD	-1.836864	0.886554	0.27993	alanine and arginine rich domain containing protein
UP in HEB	**169611**	OLFML2A	-3.147226	0.9630025	0.112873	olfactomedin-like 2A
UP in HEB	**439990**	LINC0085	-6.84549	0.9451437	0.008696	long intergenic non-protein coding RNA 857
UP in HEB	**147015**	DHRS13	-2.393602	0.939899	0.190307	dehydrogenase/reductase (SDR family) member 13
UP in HEB	**80115**	BAIAP2L2	-8.031586	0.9223336	0.003822	BAI1-associated protein 2-like 2
UP in HEB	**25819**	CCRN4L	-2.433197	0.9314897	0.185155	CCR4 carbon catabolite repression 4-like (S. cerevisiae)
UP in HEB	**90381**	TICRR	-2.273953	0.9352677	0.206763	TOPBP1-interacting checkpoint and replication regulator
UP in HEB	**27034**	ACAD8	-1.032697	0.8701264	0.488796	acyl-CoA dehydrogenase family, member 8
UP in HEB	**92181**	UBTD2	-1.152372	0.8788202	0.449885	ubiquitin domain containing 2
UP in HEB	**284114**	TMEM102	-1.357236	0.8373424	0.390329	transmembrane protein 102
UP in HEB	**23129**	PLXND1	-3.418349	0.9724074	0.093535	plexin D1
UP in HEB	**306**	ANXA3	-7.562242	0.883416	0.005291	annexin A3
UP in HEB	**126133**	ALDH16A	-1.884954	0.9177378	0.270752	aldehyde dehydrogenase 16 family, member
UP in HEB	**7175**	TPR	-1.998056	0.9369389	0.250337	translocated promoter region, nuclear basket protein
UP in HEB	**51316**	PLAC8	-5.139391	0.9933063	0.028372	placenta-specific 8
UP in HEB	**51095**	TRNT1	-1.120516	0.8842605	0.459929	tRNA nucleotidyl transferase, CCA-adding, 1
UP in HEB	**100288911**	LOC100288911	-1.338457	0.8216616	0.395443	uncharacterized LOC100288911
UP in HEB	**100289137**	FAM95C	-5.602036	0.9015236	0.020588	family with sequence similarity 95, member
UP in HEB	**57210**	SLC45A4	-5.549734	0.9709851	0.021348	solute carrier family 45, member 4
UP in HEB	**10131**	TRAP1	-2.627085	0.9596423	0.161871	TNF receptor-associated protein 1
UP in HEB	**340061**	TMEM173	-5.962152	0.9783188	0.01604	transmembrane protein 173
UP in HEB	**55132**	LARP1B	-1.240274	0.8463651	0.423292	La ribonucleoprotein domain family, member
UP in HEB	**4001**	LMNB1	-2.96504	0.9648603	0.128066	lamin B1
UP in HEB	**7917**	BAG6	-1.132398	0.8924565	0.456157	BCL2-associated athanogene 6
UP in HEB	**91807**	MYLK3	-6.093757	0.9915818	0.014642	myosin light chain kinase 3

UP in HEB	**57619**	SHROOM	-5.037209	0.9731452	0.030454	shroom family member 3
UP in HEB	**3707**	ITPKB	-3.579609	0.9241293	0.083643	inositol-trisphosphate 3-kinase B
UP in HEB	**57180**	ACTR3B	-1.463749	0.8913542	0.36255	ARP3 actin-related protein 3 homolog B
UP in HEB	**64979**	MRPL36	-1.270099	0.8997191	0.414631	mitochondrial ribosomal protein L36
UP in HEB	**158376**	LOC15837	-6.203872	0.9434548	0.013566	uncharacterized LOC158376
UP in HEB	**284459**	HKR1	-2.69488	0.8045851	0.15444	HKR1, GLI-Kruppel zinc finger family
UP in HEB	**54149**	C21orf91	-2.249907	0.9043682	0.210238	chromosome 21 open reading frame 91
UP in HEB	**6628**	SNRPB	-1.064755	0.8897363	0.478054	small nuclear ribonucleoprotein polypeptides B and B1
UP in HEB	**3251**	HPRT1	-1.557901	0.9094352	0.339645	hypoxanthine phosphoribosyltransferase 1
UP in HEB	**80746**	TSEN2	-1.979249	0.9218003	0.253622	tRNA splicing endonuclease 2 homolog (S. cerevisiae)
UP in HEB	**1063**	CENPF	-1.281951	0.8980123	0.411239	centromere protein F, 350/400kDa
UP in HEB	**8659**	ALDH4A1	-1.094424	0.8232884	0.468323	aldehyde dehydrogenase 4 family, member
UP in HEB	**29914**	UBIAD1	-1.07001	0.8616904	0.476316	UbiA prenyltransferase domain containing 1
UP in HEB	**22834**	ZNF652	-1.17166	0.8065764	0.44391	zinc finger protein 652
UP in HEB	**1662**	DDX10	-1.482565	0.8965545	0.357852	DEAD (Asp-Glu-Ala-Asp) box polypeptide
UP in HEB	**196528**	ARID2	-1.035979	0.859148	0.487685	AT rich interactive domain 2 (ARID, RFX-
UP in HEB	**91860**	CALML4	-1.632337	0.8898697	0.322565	calmodulin-like 4
UP in HEB	**54996**	2-Mar	-6.67948	0.9746742	0.009756	mitochondrial amidoxime reducing
UP in HEB	**6813**	STXBP2	-5.227125	0.9883905	0.026698	syntaxin binding protein 2
UP in HEB	**55607**	PPP1R9A	-4.493246	0.8555301	0.044402	protein phosphatase 1, regulatory subunit 9A
UP in HEB	**8543**	LMO4	-1.072596	0.8661173	0.475463	LIM domain only 4
UP in HEB	**9156**	EXO1	-1.387097	0.8985279	0.382333	exonuclease 1
UP in HEB	**7475**	WNT6	-9.99341	0.9882572	0.000981	wingless-type MMTV integration site family, member 6
UP in HEB	**8449**	DHX16	-1.108323	0.8870784	0.463833	DEAH (Asp-Glu-Ala-His) box polypeptide
UP in HEB	**8971**	H1FX	-1.378337	0.9035237	0.384662	H1 histone family, member X
UP in HEB	**10933**	MORF4L1	-1.116781	0.8931765	0.461122	mortality factor 4 like 1
UP in HEB	**51603**	METTL13	-1.052248	0.8798869	0.482216	methyltransferase like 13
UP in HEB	**159371**	SLC35G1	-1.112031	0.8431205	0.462642	solute carrier family 35, member G1
UP in HEB	**7726**	TRIM26	-1.630941	0.918049	0.322878	tripartite motif containing 26
UP in HEB	**124935**	SLC43A2	-3.406575	0.9665493	0.094302	solute carrier family 43, member 2
UP in HEB	**100463488**	MTRNR2L 10	-2.435034	0.9509485	0.184919	MT-RNR2-like 10
UP in HEB	**23511**	NUP188	-1.268149	0.8980923	0.415192	nucleoporin 188kDa
UP in HEB	**100505761**	LOC10050 5761	-2.213049	0.9192846	0.215678	uncharacterized LOC100505761
UP in HEB	**27143**	PALD1	-10.18653	0.990195	0.000858	phosphatase domain containing, paladin 1
UP in HEB	**347735**	SERINC2	-1.738259	0.9121553	0.299731	serine incorporator 2
UP in HEB	**84985**	FAM83A	-7.05745	0.9029637	0.007508	family with sequence similarity 83, member
UP in HEB	**255738**	PCSK9	-9.530731	0.9817857	0.001352	proprotein convertase subtilisin/kexin type 9
UP in HEB	**100861532**	RNA45S5	-1.479158	0.8943766	0.358698	RNA, 45S pre-ribosomal 5
UP in HEB	**9052**	GPRC5A	-1.763755	0.9232226	0.294481	G protein-coupled receptor, family C, group 5, member A
UP in HEB	**55742**	PARVA	-2.430395	0.9415791	0.185515	parvin, alpha
UP in HEB	**1207**	CLNS1A	-2.138216	0.9412769	0.227161	chloride channel, nucleotide-sensitive, 1A
UP in HEB	**153218**	SPINK13	-4.351532	0.9140399	0.048984	serine peptidase inhibitor, Kazal type 13 (putative)
UP in HEB	**1028**	CDKN1C	-11.82972	0.9976088	0.000275	cyclin-dependent kinase inhibitor 1C (p57,
UP in HEB	**3983**	ABLIM1	-2.09545	0.9373922	0.233995	actin binding LIM protein 1
UP in HEB	**27076**	LYPD3	-4.093622	0.9218803	0.058573	LY6/PLAUR domain containing 3
UP in HEB	**4436**	MSH2	-1.281553	0.8972923	0.411352	mutS homolog 2, colon cancer, nonpolyposis type 1 (E. coli)
UP in HEB	**4324**	MMP15	-4.198992	0.9830924	0.054447	matrix metallopeptidase 15 (membrane-
UP in HEB	**83595**	SOX7	-4.306751	0.9149466	0.050529	SRY (sex determining region Y)-box 7
UP in HEB	**27134**	TJP3	-6.228819	0.8127011	0.013333	tight junction protein 3

UP in HEB	**9329**	GTF3C4	-1.237451	0.8903142	0.424121	general transcription factor IIIC, polypeptide 4, 90kDa
UP in HEB	**79000**	AUNIP	-1.930845	0.9061017	0.262275	aurora kinase A and ninein interacting
UP in HEB	**890**	CCNA2	-1.634583	0.9209202	0.322063	cyclin A2
UP in HEB	**6891**	TAP2	-1.092762	0.8832382	0.468863	transporter 2, ATP-binding cassette, sub-family B (MDR/TAP)
UP in HEB	**1282**	COL4A1	-8.937815	0.9841236	0.002039	collagen, type IV, alpha 1
UP in HEB	**9828**	ARHGEF1	-1.691724	0.9043416	0.309557	Rho guanine nucleotide exchange factor
UP in HEB	**3183**	HNRNPC	-1.099202	0.8921187	0.466775	heterogeneous nuclear ribonucleoprotein C (C1/C2)
UP in HEB	**148789**	B3GALNT	-1.197921	0.8825715	0.435903	beta-1,3-N-acetylgalactosaminyltransferase 2
UP in HEB	**51734**	MSRB1	-1.410969	0.8953366	0.376059	methionine sulfoxide reductase B1
UP in HEB	**260294**	NSUN5P2	-1.59316	0.8955411	0.331445	NOP2/Sun domain family, member 5 pseudogene 2
UP in HEB	**9096**	TBX18	-1.283728	0.8550412	0.410733	T-box 18
UP in HEB	**55218**	EXD2	-8.174926	0.954122	0.00346	exonuclease 3'-5' domain containing 2
UP in HEB	**51660**	MPC1	-1.39028	0.9003236	0.381491	mitochondrial pyruvate carrier 1
UP in HEB	**6167**	RPL37	-1.501876	0.9086707	0.353094	ribosomal protein L37
UP in HEB	**283310**	OTOGL	-5	0.9236493	0.03125	otogelin-like
UP in HEB	**5997**	RGS2	-2.31349	0.9264494	0.201173	regulator of G-protein signaling 2, 24kDa
UP in HEB	**3227**	HOXC11	-7.171594	0.9306452	0.006936	homeobox C11
UP in HEB	**7913**	DEK	-2.034486	0.9403702	0.244095	DEK oncogene
UP in HEB	**65979**	PHACTR4	-1.017748	0.8757267	0.493887	phosphatase and actin regulator 4
UP in HEB	**5064**	PALM	-7.247928	0.9626202	0.006579	paralemmin
UP in HEB	**5092**	PCBD1	-1.314787	0.8986613	0.401985	pterin-4 alpha-carbinolamine dehydratase/dimerization cofactor of hepatocyte nuclear factor 1 alpha
UP in HEB	**10140**	TOB1	-1.645752	0.9197024	0.31958	transducer of ERBB2, 1
UP in HEB	**9466**	IL27RA	-3.290901	0.9008569	0.102174	interleukin 27 receptor, alpha
UP in HEB	**400569**	MED11	-1.139144	0.8835049	0.454029	mediator complex subunit 11
UP in HEB	**1431**	CS	-1.053802	0.8890074	0.481697	citrate synthase
UP in HEB	**3226**	HOXC10	-7.633951	0.9905684	0.005034	homeobox C10
UP in HEB	**9145**	SYNGR1	-8.654636	0.9571266	0.002481	synaptogyrin 1
UP in HEB	**81611**	ANP32E	-1.021432	0.8847939	0.492627	acidic (leucine-rich) nuclear phosphoprotein 32 family, member E
UP in HEB	**5074**	PAWR	-3.317479	0.9715985	0.100309	PRKC, apoptosis, WT1, regulator
UP in HEB	**9213**	XPR1	-1.070389	0.8584636	0.47619	xenotropic and polytropic retrovirus receptor
UP in HEB	**246777**	SPESP1	-8.398031	0.9445748	0.002964	sperm equatorial segment protein 1
UP in HEB	**25830**	SULT4A1	-8.579944	0.9538909	0.002613	sulfotransferase family 4A, member 1
UP in HEB	**4495**	MT1G	-11.35938	0.9963998	0.000381	metallothionein 1G
UP in HEB	**84929**	FIBCD1	-4.823535	0.9823457	0.035316	fibrinogen C domain containing 1
UP in HEB	**7092**	TLL1	-8.336878	0.9413035	0.003093	tolloid-like 1
UP in HEB	**23279**	NUP160	-1.856417	0.925756	0.276161	nucleoporin 160kDa
UP in HEB	**113763**	C7orf29	-8.613237	0.9782566	0.002554	chromosome 7 open reading frame 29
UP in HEB	**84866**	TMEM25	-7.273018	0.9366811	0.006466	transmembrane protein 25
UP in HEB	**65999**	LRRC61	-9.675201	0.9946664	0.001223	leucine rich repeat containing 61
UP in HEB	**8797**	TNFRSF10 A	-4.003726	0.9680694	0.062339	tumor necrosis factor receptor superfamily, member 10a
UP in HEB	**100303728**	SLC25A5-AS1	-1.038374	0.8745533	0.486876	SLC25A5 antisense RNA 1
UP in HEB	**8908**	GYG2	-9.73358	0.9851192	0.001175	glycogenin 2
UP in HEB	**84881**	RPUSD4	-1.553846	0.901737	0.340601	RNA pseudouridylate synthase domain containing 4
UP in HEB	**29766**	TMOD3	-1.222751	0.8955322	0.428465	tropomodulin 3 (ubiquitous)
UP in HEB	**55076**	TMEM45	-1.043692	0.8719398	0.485085	transmembrane protein 45A
UP in HEB	**26135**	SERBP1	-1.337743	0.9030615	0.395639	SERPINE1 mRNA binding protein 1
UP in HEB	**653**	BMP5	-10.61164	0.9932974	0.000639	bone morphogenetic protein 5

UP in HEB	**23457**	ABCB9	-1.651849	0.8828826	0.318232	ATP-binding cassette, sub-family B (MDR/TAP), member 9
UP in HEB	**84126**	ATRIP	-1.21861	0.8869895	0.429697	ATR interacting protein
UP in HEB	**6584**	SLC22A5	-2.329591	0.9383345	0.198941	solute carrier family 22 (organic cation/carnitine transporter), member 5
UP in HEB	**10799**	RPP40	-1.887973	0.9216669	0.270186	ribonuclease P/MRP 40kDa subunit
UP in HEB	**8622**	PDE8B	-4.18194	0.977661	0.055095	phosphodiesterase 8B
UP in HEB	**5590**	PRKCZ	-2.121664	0.9174445	0.229782	protein kinase C, zeta
UP in HEB	**154141**	MBOAT1	-2.020628	0.9029993	0.246451	membrane bound O-acyltransferase domain containing 1
UP in HEB	**100131193**	KIAA1984 -AS1	-1.693487	0.8117766	0.309179	KIAA1984 antisense RNA 1
UP in HEB	**8645**	KCNK5	-8.390885	0.944317	0.002979	potassium channel, subfamily K, member 5
UP in HEB	**126321**	MFSD12	-1.048651	0.8882429	0.48342	major facilitator superfamily domain
UP in HEB	**4920**	ROR2	-10.42136	0.991964	0.000729	receptor tyrosine kinase-like orphan receptor
UP in HEB	**7837**	PXDN	-8.497747	0.9990311	0.002766	peroxidasin homolog (Drosophila)
UP in HEB	**54830**	NUP62CL	-7.761551	0.9014436	0.004608	nucleoporin 62kDa C-terminal like
UP in HEB	**342667**	STAC2	-4.399827	0.9782122	0.047372	SH3 and cysteine rich domain 2
UP in HEB	**27109**	ATP5S	-1.877004	0.8933099	0.272249	ATP synthase, H+ transporting, mitochondrial Fo complex, subunit s (factor B)
UP in HEB	**11270**	NRM	-1.757627	0.9227337	0.295734	nurim (nuclear envelope membrane protein)
UP in HEB	**80223**	RAB11FIP	-6.129948	0.9902128	0.014279	RAB11 family interacting protein 1 (class I)
UP in HEB	**283248**	RCOR2	-7.922832	0.9664249	0.004121	REST corepressor 2
UP in HEB	**9794**	MAML1	-1.254541	0.8908208	0.419127	mastermind-like 1 (Drosophila)
UP in HEB	**9267**	CYTH1	-1.347337	0.8948566	0.393017	cytohesin 1
UP in HEB	**79075**	DSCC1	-1.340758	0.8922965	0.394813	defective in sister chromatid cohesion 1 homolog (S. cerevisiae)
UP in HEB	**54985**	HCFC1R1	-1.819833	0.9256049	0.283254	host cell factor C1 regulator 1 (XPO1
UP in HEB	**196996**	GRAMD2	-7.031219	0.9009903	0.007645	GRAM domain containing 2
UP in HEB	**51337**	THEM6	-2.366987	0.9468149	0.19385	thioesterase superfamily member 6
UP in HEB	**11214**	AKAP13	-1.045118	0.8686774	0.484605	A kinase (PRKA) anchor protein 13
UP in HEB	**283989**	TSEN54	-1.042493	0.8761445	0.485488	tRNA splicing endonuclease 54 homolog (S. cerevisiae)
UP in HEB	**10410**	IFITM3	-5.673969	0.9968443	0.019587	interferon induced transmembrane protein 3
UP in HEB	**9516**	LITAF	-2.272869	0.9429036	0.206918	lipopolysaccharide-induced TNF factor
UP in HEB	**7070**	THY1	-10.71825	0.993973	0.000594	Thy-1 cell surface antigen
UP in HEB	**56521**	DNAJC12	-1.608809	0.8983857	0.327869	DnaJ (Hsp40) homolog, subfamily C,
UP in HEB	**11080**	DNAJB4	-1.619091	0.91596	0.32554	DnaJ (Hsp40) homolog, subfamily B,
UP in HEB	**6581**	SLC22A3	-10.66267	0.9936174	0.000617	solute carrier family 22 (extraneuronal monoamine transporter), member 3
UP in HEB	**143686**	SESN3	-2.249476	0.9109286	0.2103	sestrin 3
UP in HEB	**23109**	DDN	-4.540659	0.9563621	0.042966	dendrin
UP in HEB	**54502**	RBM47	-1.532645	0.879958	0.345643	RNA binding motif protein 47
UP in HEB	**220729**	LOC22072 9	-1.101112	0.8767312	0.466157	succinate dehydrogenase complex, subunit A, flavoprotein (Fp) pseudogene
UP in HEB	**29102**	DROSHA	-1.669454	0.9212047	0.314372	drosha, ribonuclease type III
UP in HEB	**23521**	RPL13A	-1.230664	0.8993635	0.426121	ribosomal protein L13a
UP in HEB	**55274**	PHF10	-1.409206	0.8988746	0.376519	PHD finger protein 10
UP in HEB	**1286**	COL4A4	-6.798742	0.9424858	0.008982	collagen, type IV, alpha 4
UP in HEB	**139285**	AMER1	-2.630551	0.9196668	0.161482	APC membrane recruitment protein 1
UP in HEB	**4744**	NEFH	-9.73957	0.9957864	0.00117	neurofilament, heavy polypeptide
UP in HEB	**283871**	PGP	-2.200014	0.9390456	0.217635	phosphoglycolate phosphatase
UP in HEB	**55559**	HAUS7	-1.196217	0.8873273	0.436418	HAUS augmin-like complex, subunit 7
UP in HEB	**84656**	GLYR1	-1.969588	0.927125	0.255326	glyoxylate reductase 1 homolog
UP in HEB	**153090**	DAB2IP	-1.419669	0.876669	0.373798	DAB2 interacting protein
UP in HEB	**440894**	LOC44089	-4.554589	0.9873504	0.042553	uncharacterized LOC440894

UP in HEB	**56905**	C15orf39	-2.392192	0.9454726	0.190493	chromosome 15 open reading frame 39
UP in HEB	**84451**	KIAA1804	-1.070302	0.8238306	0.476219	mixed lineage kinase 4
UP in HEB	**57645**	POGK	-1.41409	0.8975145	0.375246	pogo transposable element with KRAB
UP in HEB	**81544**	GDPD5	-2.06301	0.8900297	0.239316	glycerophosphodiester phosphodiesterase domain containing 5
UP in HEB	**55374**	TMCO6	-1.805809	0.9005725	0.286021	transmembrane and coiled-coil domains 6
UP in HEB	**94031**	HTRA3	-10.23162	0.9906039	0.000832	HtrA serine peptidase 3
UP in HEB	**8334**	HIST1H2A	-1.880731	0.9228314	0.271546	histone cluster 1, H2ac
UP in HEB	**29855**	UBN1	-1.004432	0.87514	0.498466	ubinuclein 1
UP in HEB	**2177**	FANCD2	-1.679732	0.9203069	0.312141	Fanconi anemia, complementation group D2
UP in HEB	**587**	BCAT2	-1.457506	0.899728	0.364122	branched chain amino-acid transaminase 2, mitochondrial
UP in HEB	**6222**	RPS18	-1.672002	0.9246538	0.313817	ribosomal protein S18
UP in HEB	**753**	LDLRAD4	-7.407693	0.9290184	0.005889	low density lipoprotein receptor class A domain containing 4
UP in HEB	**8293**	SERF1A	-7.526173	0.8794158	0.005425	small EDRK-rich factor 1A (telomeric)
UP in HEB	**2171**	FABP5	-2.709404	0.9594912	0.152893	fatty acid binding protein 5 (psoriasis-
UP in HEB	**22823**	MTF2	-1.727047	0.8964745	0.30207	metal response element binding transcription factor 2
UP in HEB	**56946**	C11orf30	-1.383714	0.8703931	0.383231	chromosome 11 open reading frame 30
UP in HEB	**2170**	FABP3	-2.246201	0.8472363	0.210778	fatty acid binding protein 3, muscle and heart (mammary-derived growth inhibitor)
UP in HEB	**9182**	RASSF9	-6.800312	0.9563888	0.008972	Ras association (RalGDS/AF-6) domain family (N-terminal) member 9
UP in HEB	**25956**	SEC31B	-3.325016	0.935641	0.099786	SEC31 homolog B (S. cerevisiae)
UP in HEB	**2631**	GBAS	-1.602834	0.921178	0.32923	glioblastoma amplified sequence
UP in HEB	**27237**	ARHGEF1	-9.455327	0.9803545	0.001425	Rho guanine nucleotide exchange factor
UP in HEB	**1290**	COL5A2	-3.004687	0.9666649	0.124595	collagen, type V, alpha 2
UP in HEB	**26048**	ZNF500	-1.652209	0.8730777	0.318153	zinc finger protein 500
UP in HEB	**57486**	NLN	-1.171878	0.8818337	0.443843	neurolysin (metallopeptidase M3 family)
UP in HEB	**51027**	BOLA1	-2.242916	0.9281917	0.211259	bolA homolog 1 (E. coli)
UP in HEB	**100874323**	HOXA-	-7.019035	0.9598734	0.00771	HOXA cluster antisense RNA 4
UP in HEB	**949**	SCARB1	-3.423958	0.9755809	0.093172	scavenger receptor class B, member 1
UP in HEB	**5212**	VIT	-7.994353	0.9940086	0.003922	vitrin
UP in HEB	**729082**	OIP5-AS1	-2.14249	0.939659	0.226489	OIP5 antisense RNA 1
UP in HEB	**84527**	ZNF559	-4.461952	0.8705442	0.045375	zinc finger protein 559
UP in HEB	**978**	CDA	-3.009228	0.9649848	0.124203	cytidine deaminase
UP in HEB	**4774**	NFIA	-3.114143	0.9404413	0.115491	nuclear factor I/A
UP in HEB	**80305**	TRABD	-1.481923	0.9049105	0.358011	TraB domain containing
UP in HEB	**9355**	LHX2	-5.41879	0.8134212	0.023377	LIM homeobox 2
UP in HEB	**9774**	BCLAF1	-1.382755	0.9013814	0.383486	BCL2-associated transcription factor 1
UP in HEB	**2870**	GRK6	-1.092527	0.8846783	0.468939	G protein-coupled receptor kinase 6
UP in HEB	**23089**	PEG10	-3.540692	0.9785677	0.08593	paternally expressed 10
UP in HEB	**4211**	MEIS1	-2.421587	0.9319697	0.186651	Meis homeobox 1
UP in HEB	**55270**	NUDT15	-1.114761	0.8811492	0.461768	nudix (nucleoside diphosphate linked moiety X)-type motif 15
UP in HEB	**6392**	SDHD	-1.269949	0.8990613	0.414674	succinate dehydrogenase complex, subunit D, integral membrane protein
UP in HEB	**11091**	WDR5	-1.062382	0.8850694	0.478841	WD repeat domain 5
UP in HEB	**7543**	ZFX	-2.660101	0.9545487	0.158208	zinc finger protein, X-linked
UP in HEB	**389362**	PSMG4	-1.422984	0.9007236	0.37294	proteasome (prosome, macropain) assembly chaperone 4
UP in HEB	**100506834**	LOC10050 6834	-4.02319	0.8202837	0.061503	uncharacterized LOC100506834
UP in HEB	**64359**	NXN	-2.583321	0.9565666	0.166856	nucleoredoxin
UP in HEB	**79840**	NHEJ1	-1.43898	0.8918253	0.368828	nonhomologous end-joining factor 1
UP in HEB	**200424**	TET3	-1.596778	0.8816737	0.330614	tet methylcytosine dioxygenase 3

UP in HEB	**23082**	PPRC1	-1.158836	0.890732	0.447874	peroxisome proliferator-activated receptor gamma, coactivator-related 1
UP in HEB	**1952**	CELSR2	-6.942515	0.9697939	0.00813	cadherin, EGF LAG seven-pass G-type
UP in HEB	**1525**	CXADR	-3.972511	0.9731986	0.063702	coxsackie virus and adenovirus receptor
UP in HEB	**125488**	TTC39C	-1.967084	0.9245026	0.255769	tetratricopeptide repeat domain 39C
UP in HEB	**84300**	MNF1	-1.571684	0.9025993	0.336415	mitochondrial nucleoid factor 1
UP in HEB	**85409**	NKD2	-6.934674	0.8105499	0.008174	naked cuticle homolog 2 (Drosophila)
UP in HEB	**11183**	MAP4K5	-1.512809	0.9035415	0.350428	mitogen-activated protein kinase kinase kinase kinase 5
UP in HEB	**4040**	LRP6	-1.139705	0.8582858	0.453852	low density lipoprotein receptor-related
UP in HEB	**677798**	SNORA9	-6.853829	0.8007805	0.008646	small nucleolar RNA, H/ACA box 9
UP in HEB	**2181**	ACSL3	-1.797042	0.9251427	0.287764	acyl-CoA synthetase long-chain family
UP in HEB	**55544**	RBM38	-4.484749	0.9832702	0.044664	RNA binding motif protein 38
UP in HEB	**286257**	C9orf142	-3.36236	0.9743986	0.097236	chromosome 9 open reading frame 142
UP in HEB	**9167**	COX7A2L	-1.184122	0.8964123	0.440092	cytochrome c oxidase subunit VIIa polypeptide 2 like
UP in HEB	**338707**	B4GALNT	-9.807355	0.9861059	0.001116	beta-1,4-N-acetyl-galactosaminyl transferase
UP in HEB	**79752**	ZFAND1	-1.025281	0.8836116	0.491315	zinc finger, AN1-type domain 1
UP in HEB	**22898**	DENND3	-1.040124	0.8248618	0.486286	DENN/MADD domain containing 3
UP in HEB	**5364**	PLXNB1	-4.284977	0.9784611	0.051297	plexin B1
UP in HEB	**51090**	PLLP	-6.981567	0.9433392	0.007913	plasmolipin
UP in HEB	**91608**	RASL10B	-4.708345	0.9270006	0.038251	RAS-like, family 10, member B
UP in HEB	**4255**	MGMT	-10.62388	0.9933508	0.000634	O-6-methylguanine-DNA methyltransferase
UP in HEB	**50649**	ARHGEF4	-8.938599	0.9674916	0.002038	Rho guanine nucleotide exchange factor
UP in HEB	**3949**	LDLR	-1.163243	0.885105	0.446508	low density lipoprotein receptor
UP in HEB	**6590**	SLPI	-9.067882	0.9994577	0.001863	secretory leukocyte peptidase inhibitor
UP in HEB	**388722**	C1orf53	-2.183122	0.908084	0.220199	chromosome 1 open reading frame 53
UP in HEB	**10501**	SEMA6B	-8.172428	0.953962	0.003466	sema domain, transmembrane domain (TM), and cytoplasmic domain, (semaphorin) 6B
UP in HEB	**5827**	PXMP2	-1.261736	0.8905364	0.417042	peroxisomal membrane protein 2, 22kDa
UP in HEB	**10092**	ARPC5	-1.168011	0.8952922	0.445034	actin related protein 2/3 complex, subunit 5, 16kDa
UP in HEB	**132430**	PABPC4L	-8.840253	0.9638025	0.002182	poly(A) binding protein, cytoplasmic 4-like
UP in HEB	**10149**	GPR64	-7.096056	0.9805145	0.007309	G protein-coupled receptor 64
UP in HEB	**8165**	AKAP1	-1.954002	0.9256583	0.258099	A kinase (PRKA) anchor protein 1
UP in HEB	**171177**	RHOV	-10.27096	0.9967465	0.000809	ras homolog family member V
UP in HEB	**1717**	DHCR7	-1.253111	0.894661	0.419543	7-dehydrocholesterol reductase
UP in HEB	**140730**	RIMS4	-10.28116	0.9909862	0.000804	regulating synaptic membrane exocytosis 4
UP in HEB	**117608**	ZNF354B	-8.7131	0.9593312	0.002383	zinc finger protein 354B
UP in HEB	**80854**	SETD7	-1.107274	0.8869006	0.46417	SET domain containing (lysine methyltransferase) 7
UP in HEB	**3070**	HELLS	-1.614473	0.9160577	0.326584	helicase, lymphoid-specific
UP in HEB	**4488**	MSX2	-3.664081	0.9049549	0.078886	msh homeobox 2
UP in HEB	**657**	BMPR1A	-1.090313	0.878758	0.46966	bone morphogenetic protein receptor, type IA
UP in HEB	**55592**	GOLGA2P	-3.143311	0.9453749	0.11318	golgin A2 pseudogene 5
UP in HEB	**2353**	FOS	-3.161997	0.9628158	0.111723	FBJ murine osteosarcoma viral oncogene homolog
UP in HEB	**8031**	NCOA4	-1.163219	0.8945099	0.446515	nuclear receptor coactivator 4
UP in HEB	**79897**	RPP21	-1.539894	0.9062261	0.343911	ribonuclease P/MRP 21kDa subunit
UP in HEB	**54436**	SH3TC1	-8.667703	0.9576066	0.002459	SH3 domain and tetratricopeptide repeats 1
UP in HEB	**9836**	LCMT2	-1.342766	0.8817626	0.394264	leucine carboxyl methyltransferase 2
UP in HEB	**145978**	LINC0005	-7.707359	0.8970612	0.004785	long intergenic non-protein coding RNA 52
UP in HEB	**8869**	ST3GAL5	-4.054711	0.9429481	0.060174	ST3 beta-galactoside alpha-2,3-
UP in HEB	**51157**	ZNF580	-1.016154	0.8755756	0.494433	zinc finger protein 580
UP in HEB	**100133941**	CD24	-3.585199	0.980799	0.08332	CD24 molecule
UP in HEB	**140462**	ASB9	-7.399566	0.9807812	0.005923	ankyrin repeat and SOCS box containing 9
UP in HEB	**145773**	FAM81A	-3.138472	0.946246	0.11356	family with sequence similarity 81, member

UP in HEB	**57510**	XPO5	-1.858003	0.9236315	0.275858	exportin 5
UP in HEB	**9750**	FAM65B	-8.736966	0.9601934	0.002344	family with sequence similarity 65, member
UP in HEB	**7371**	UCK2	-2.006624	0.9368677	0.248855	uridine-cytidine kinase 2
UP in HEB	**80775**	TMEM177	-1.431352	0.8882963	0.370783	transmembrane protein 177
UP in HEB	**7414**	VCL	-1.107362	0.8921809	0.464142	vinculin
UP in HEB	**201161**	CENPV	-7.91594	0.9974665	0.004141	centromere protein V
UP in HEB	**5445**	PON2	-1.403788	0.9050438	0.377935	paraoxonase 2
UP in HEB	**4111**	MAGEA12	-6.616758	0.9947108	0.01019	melanoma antigen family A, 12
UP in HEB	**65993**	MRPS34	-1.545155	0.9092929	0.342659	mitochondrial ribosomal protein S34
UP in HEB	**53947**	A4GALT	-1.092385	0.8289775	0.468986	alpha 1,4-galactosyltransferase
UP in HEB	**51326**	ARL17A	-1.692062	0.9172756	0.309484	ADP-ribosylation factor-like 17A
UP in HEB	**10588**	MTHFS	-1.739668	0.908804	0.299439	5,10-methenyltetrahydrofolate synthetase (5-formyltetrahydrofolate cyclo-ligase)
UP in HEB	**221710**	SMIM13	-1.863955	0.912982	0.274722	small integral membrane protein 13
UP in HEB	**25837**	RAB26	-7.491185	0.9918573	0.005558	RAB26, member RAS oncogene family
UP in HEB	**6882**	TAF11	-2.690368	0.9554732	0.154924	TAF11 RNA polymerase II, TATA box binding protein (TBP)-associated factor, 28kDa
UP in HEB	**64223**	MLST8	-2.644838	0.95862	0.159891	MTOR associated protein, LST8 homolog (S. cerevisiae)
UP in HEB	**3795**	KHK	-1.766948	0.8413071	0.29383	ketohexokinase (fructokinase)
UP in HEB	**4913**	NTHL1	-2.199477	0.9382812	0.217716	nth endonuclease III-like 1 (E. coli)
UP in HEB	**57473**	ZNF512B	-1.822185	0.9203513	0.282792	zinc finger protein 512B
UP in HEB	**5357**	PLS1	-1.619839	0.8920831	0.325372	plastin 1
UP in HEB	**53353**	LRP1B	-2.986061	0.8144701	0.126214	low density lipoprotein receptor-related protein 1B
UP in HEB	**154043**	CNKSR3	-4.181014	0.947215	0.05513	CNKSR family member 3
UP in HEB	**64864**	RFX7	-1.18095	0.8794958	0.441061	regulatory factor X, 7
UP in HEB	**3872**	KRT17	-5.991671	0.9538642	0.015715	keratin 17
UP in HEB	**3206**	HOXA10	-6.851269	0.9669582	0.008661	homeobox A10
UP in HEB	**84342**	COG8	-1.189253	0.8908742	0.43853	component of oligomeric golgi complex 8
UP in HEB	**54492**	NEURL1B	-2.442116	0.9424058	0.184014	neuralized homolog 1B (Drosophila)
UP in HEB	**3093**	UBE2K	-1.016316	0.8781535	0.494377	ubiquitin-conjugating enzyme E2K
UP in HEB	**2187**	FANCB	-1.094756	0.8250573	0.468215	Fanconi anemia, complementation group B
UP in HEB	**5307**	PITX1	-3.948857	0.9828969	0.064755	paired-like homeodomain 1
UP in HEB	**85462**	FHDC1	-3.489966	0.8506587	0.089005	FH2 domain containing 1
UP in HEB	**286826**	LIN9	-1.187407	0.8636105	0.439091	lin-9 homolog (C. elegans)
UP in HEB	**4736**	RPL10A	-1.38616	0.9053016	0.382582	ribosomal protein L10a
UP in HEB	**25987**	TSKU	-1.427421	0.8967856	0.371795	tsukushi, small leucine rich proteoglycan
UP in HEB	**4175**	MCM6	-1.187803	0.8936477	0.438971	minichromosome maintenance complex component 6
UP in HEB	**79622**	SNRNP25	-2.576865	0.9590378	0.167605	small nuclear ribonucleoprotein 25kDa
UP in HEB	**65985**	AACS	-1.176051	0.8861273	0.442561	acetoacetyl-CoA synthetase
UP in HEB	**6692**	SPINT1	-8.888743	0.9656071	0.00211	serine peptidase inhibitor, Kunitz type 1
UP in HEB	**125050**	RN7SK	-2.827587	0.9364677	0.140868	RNA, 7SK small nuclear
UP in HEB	**5026**	P2RX5	-7.512938	0.9901595	0.005475	purinergic receptor P2X, ligand-gated ion channel, 5
UP in HEB	**494514**	C18orf56	-1.437037	0.8534233	0.369325	chromosome 18 open reading frame 56
UP in HEB	**8527**	DGKD	-3.019958	0.9666382	0.123283	diacylglycerol kinase, delta 130kDa
UP in HEB	**8864**	PER2	-1.373587	0.8641616	0.385931	period circadian clock 2
UP in HEB	**81037**	CLPTM1L	-1.435832	0.9062083	0.369634	CLPTM1-like
UP in HEB	**8357**	HIST1H3H	-4.960002	0.9090174	0.032129	histone cluster 1, H3h
UP in HEB	**332**	BIRC5	-1.036809	0.8871229	0.487404	baculoviral IAP repeat containing 5
UP in HEB	**9540**	TP53I3	-1.618135	0.91596	0.325756	tumor protein p53 inducible protein 3
UP in HEB	**291**	SLC25A4	-4.491344	0.9546109	0.04446	solute carrier family 25 (mitochondrial carrier; adenine nucleotide translocator), member 4

UP in HEB	**55722**	CEP72	-1.712063	0.9092396	0.305223	centrosomal protein 72kDa
UP in HEB	**154007**	SNRNP48	-2.708972	0.952993	0.152939	small nuclear ribonucleoprotein 48kDa
UP in HEB	**100270746**	LOC100270746	-7.183222	0.8409426	0.006881	uncharacterized LOC100270746
UP in HEB	**100287015**	LOC100287015	-1.489543	0.8157591	0.356125	uncharacterized LOC100287015
UP in HEB	**8348**	HIST1H2B	-8.069674	0.9248049	0.003722	histone cluster 1, H2bo
UP in HEB	**104**	ADARB1	-2.248753	0.9075417	0.210406	adenosine deaminase, RNA-specific, B1
UP in HEB	**84614**	ZBTB37	-2.051977	0.8287198	0.241153	zinc finger and BTB domain containing 37
UP in HEB	**79616**	CCNJL	-8.873444	0.9651004	0.002132	cyclin J-like
UP in HEB	**23522**	KAT6B	-1.267821	0.8769712	0.415287	K(lysine) acetyltransferase 6B
UP in HEB	**192111**	PGAM5	-1.520233	0.9073106	0.34863	phosphoglycerate mutase family member 5
UP in HEB	**221883**	HOXA11-AS	-6.105304	0.9316408	0.014525	HOXA11 antisense RNA
UP in HEB	**22838**	RNF44	-1.492355	0.8965011	0.355432	ring finger protein 44
UP in HEB	**23012**	STK38L	-1.481952	0.8856917	0.358004	serine/threonine kinase 38 like
UP in HEB	**2027**	ENO3	-5.676758	0.9965065	0.019549	enolase 3 (beta, muscle)
UP in HEB	**1366**	CLDN7	-1.69168	0.8801536	0.309566	claudin 7
UP in HEB	**10669**	CGREF1	-5.223036	0.9624958	0.026774	cell growth regulator with EF-hand domain 1
UP in HEB	**27173**	SLC39A1	-1.039589	0.8880918	0.486466	solute carrier family 39 (zinc transporter), member 1
UP in HEB	**9053**	MAP7	-4.169327	0.9713318	0.055579	microtubule-associated protein 7
UP in HEB	**147111**	NOTUM	-8.363405	0.9427169	0.003036	notum pectinacetylesterase homolog (Drosophila)
UP in U87	**79572**	ATP13A3	1.5588462	0.9090974	2.946181	ATPase type 13A3
UP in U87	**2634**	GBP2	2.9543894	0.9372767	7.751037	guanylate binding protein 2, interferon-
UP in U87	**9363**	RAB33A	7.33985	0.8594681	162	RAB33A, member RAS oncogene family
UP in U87	**339263**	C17orf51	1.2553982	0.8697086	2.38733	chromosome 17 open reading frame 51
UP in U87	**10233**	LRRC23	2.3728895	0.9290007	5.179775	leucine rich repeat containing 23
UP in U87	**26002**	MOXD1	9.892391	0.9933774	950.4	monooxygenase, DBH-like 1
UP in U87	**9961**	MVP	3.4201516	0.9758476	10.70455	major vault protein
UP in U87	**8193**	DPF1	2.8664497	0.9262894	7.292683	D4, zinc and double PHD fingers family 1
UP in U87	**65059**	RAPH1	2.3876138	0.9418991	5.232911	Ras association (RalGDS/AF-6) and pleckstrin homology domains 1
UP in U87	**1203**	CLN5	1.6058337	0.9057016	3.043716	ceroid-lipofuscinosis, neuronal 5
UP in U87	**4261**	CIITA	7.7264074	0.9769943	211.7778	class II, major histocompatibility complex, transactivator
UP in U87	**100289092**	LOC100289092	1.2038029	0.8652195	2.303461	uncharacterized LOC100289092
UP in U87	**84649**	DGAT2	3.2985727	0.9570999	9.839416	diacylglycerol O-acyltransferase 2
UP in U87	**4094**	MAF	9.1480528	0.9732252	567.3333	v-maf musculoaponeurotic fibrosarcoma oncogene homolog (avian)
UP in U87	**221491**	C6orf1	1.6670596	0.9143421	3.175667	chromosome 6 open reading frame 1
UP in U87	**8091**	HMGA2	10.466276	0.9996622	1414.696	high mobility group AT-hook 2
UP in U87	**1**	A1BG	6.495855	0.9114619	90.25	alpha-1-B glycoprotein
UP in U87	**100534611**	TM4SF19-TCTEX1D2	8.8276073	0.9632158	454.3333	TM4SF19-TCTEX1D2 readthrough
UP in U87	**8100**	IFT88	2.2891502	0.8929187	4.887681	intraflagellar transport 88 homolog (Chlamydomonas)
UP in U87	**8941**	CDK5R2	5.5545889	0.8485341	47	cyclin-dependent kinase 5, regulatory subunit 2 (p39)
UP in U87	**255758**	TCTEX1D	1.5924696	0.913871	3.015651	Tctex1 domain containing 2
UP in U87	**79611**	ACSS3	8.4164457	0.9458104	341.6667	acyl-CoA synthetase short-chain family member 3
UP in U87	**23659**	PLA2G15	1.8854817	0.9183956	3.694763	phospholipase A2, group XV
UP in U87	**9842**	PLEKHM1	2.0012784	0.9346276	4.003546	pleckstrin homology domain containing, family M (with RUN domain) member 1

UP in U87	**90313**	TP53I13	1.5180336	0.9052572	2.864004	tumor protein p53 inducible protein 13
UP in U87	**8754**	ADAM9	1.1053011	0.8911586	2.151438	ADAM metallopeptidase domain 9
UP in U87	**84240**	ZCCHC9	1.0144262	0.879718	2.020099	zinc finger, CCHC domain containing 9
UP in U87	**5922**	RASA2	1.2492748	0.8384358	2.377219	RAS p21 protein activator 2
UP in U87	**92017**	SNX29	1.1515502	0.8586769	2.221525	sorting nexin 29
UP in U87	**26289**	AK5	6.2341521	0.9606379	75.27778	adenylate kinase 5
UP in U87	**24139**	EML2	1.7328514	0.9160933	3.323841	echinoderm microtubule associated protein
UP in U87	**439931**	THAP7-	1.0998525	0.8025672	2.143328	THAP7 antisense RNA 1
UP in U87	**254887**	ZDHHC23	1.3660675	0.8072075	2.57767	zinc finger, DHHC-type containing 23
UP in U87	**80820**	EEPD1	7.0306671	0.8702242	130.75	endonuclease/exonuclease/phosphatase family domain containing 1
UP in U87	**55973**	BCAP29	2.1014545	0.9375967	4.291418	B-cell receptor-associated protein 29
UP in U87	**80199**	FUZ	3.711422	0.9588511	13.09934	fuzzy planar cell polarity protein
UP in U87	**48**	ACO1	3.3513465	0.974852	10.20601	aconitase 1, soluble
UP in U87	**9955**	HS3ST3A1	2.6138928	0.9561132	6.121532	heparan sulfate (glucosamine) 3-O-sulfotransferase 3A1
UP in U87	**1155**	TBCB	1.471583	0.9061817	2.77326	tubulin folding cofactor B
UP in U87	**8634**	RTCA	1.8449889	0.9244315	3.592502	RNA 3'-terminal phosphate cyclase
UP in U87	**1843**	DUSP1	2.2511692	0.9430814	4.760685	dual specificity phosphatase 1
UP in U87	**10544**	PROCR	2.0304653	0.9399257	4.085366	protein C receptor, endothelial
UP in U87	**781**	CACNA2D 1	6.9028775	0.9478906	119.6667	calcium channel, voltage-dependent, alpha 2/delta subunit 1
UP in U87	**5327**	PLAT	1.5486488	0.8548189	2.92543	plasminogen activator, tissue
UP in U87	**112770**	C1orf85	1.1489186	0.8897452	2.217476	chromosome 1 open reading frame 85
UP in U87	**203286**	ANKS6	1.3559683	0.8799936	2.559689	ankyrin repeat and sterile alpha motif domain containing 6
UP in U87	**644128**	RPL23AP5	1.5328282	0.8713976	2.893525	ribosomal protein L23a pseudogene 53
UP in U87	**100506990**	LOC10050 6990	2.017414	0.9279873	4.048574	uncharacterized LOC100506990
UP in U87	**1318**	SLC31A2	1.7821061	0.9123509	3.439279	solute carrier family 31 (copper transporters), member 2
UP in U87	**64393**	ZMAT3	1.8626114	0.9180223	3.636653	zinc finger, matrin-type 3
UP in U87	**83882**	TSPAN10	1.8495892	0.9018703	3.603976	tetraspanin 10
UP in U87	**402778**	IFITM10	4.8094144	0.9835725	28.04	interferon induced transmembrane protein 10
UP in U87	**10597**	TRAPPC2 P1	1.1280484	0.864455	2.185629	trafficking protein particle complex 2 pseudogene 1
UP in U87	**66008**	TRAK2	1.2629911	0.8917542	2.399928	trafficking protein, kinesin binding 2
UP in U87	**2673**	GFPT1	1.4315194	0.9005636	2.697306	glutamine--fructose-6-phosphate
UP in U87	**130271**	PLEKHH2	5.1514394	0.9230803	35.54167	pleckstrin homology domain containing, family H (with MyTH4 domain) member 2
UP in U87	**51626**	DYNC2LI	1.3797137	0.8850872	2.602167	dynein, cytoplasmic 2, light intermediate
UP in U87	**5224**	PGAM2	2.0845902	0.847423	4.241546	phosphoglycerate mutase 2 (muscle)
UP in U87	**83941**	TM2D1	1.1773703	0.8880118	2.261642	TM2 domain containing 1
UP in U87	**100506939**	PRKG1-	4.6755651	0.9270894	25.55556	PRKG1 antisense RNA 1
UP in U87	**29114**	TAGLN3	10.948124	0.9949864	1975.667	transgelin 3
UP in U87	**9110**	MTMR4	1.1013016	0.8868917	2.145482	myotubularin related protein 4
UP in U87	**2121**	EVC	4.7041483	0.9914929	26.06692	Ellis van Creveld syndrome
UP in U87	**286144**	TRIQK	1.8287286	0.9133732	3.552239	triple QxxK/R motif containing
UP in U87	**375690**	WASH5P	1.2576892	0.8912564	2.391124	WAS protein family homolog 5 pseudogene
UP in U87	**79625**	NDNF	7.6048621	0.8878251	194.6667	neuron-derived neurotrophic factor
UP in U87	**9497**	SLC4A7	1.3008863	0.8901364	2.463802	solute carrier family 4, sodium bicarbonate cotransporter, member 7
UP in U87	**8745**	ADAM23	7.2075775	0.9905239	147.8077	ADAM metallopeptidase domain 23
UP in U87	**79048**	SECISBP2	1.016389	0.8708287	2.022849	SECIS binding protein 2
UP in U87	**80131**	LRRC8E	2.9498208	0.9550198	7.726531	leucine rich repeat containing 8 family,
UP in U87	**100129792**	CCDC152	3.5348537	0.9123331	11.59036	coiled-coil domain containing 152

UP in U87	**9563**	H6PD	3.1719149	0.9685761	9.012422	hexose-6-phosphate dehydrogenase (glucose 1-dehydrogenase)
UP in U87	**5077**	PAX3	8.0945176	0.926245	273.3333	paired box 3
UP in U87	**260436**	FDCSP	6.946419	0.8121678	123.3333	follicular dendritic cell secreted protein
UP in U87	**78991**	PCYOX1L	1.3765511	0.8649706	2.596469	prenylcysteine oxidase 1 like
UP in U87	**59284**	CACNG7	8.5130696	0.9504151	365.3333	calcium channel, voltage-dependent, gamma subunit 7
UP in U87	**7439**	BEST1	2.9267388	0.9110797	7.603896	bestrophin 1
UP in U87	**4294**	MAP3K10	1.9969253	0.9249027	3.991484	mitogen-activated protein kinase kinase
UP in U87	**1545**	CYP1B1	1.3596524	0.9018437	2.566233	cytochrome P450, family 1, subfamily B, polypeptide 1
UP in U87	**1535**	CYBA	1.5173098	0.9088485	2.862568	cytochrome b-245, alpha polypeptide
UP in U87	**10135**	NAMPT	4.6994632	0.9933241	25.98241	nicotinamide phosphoribosyltransferase
UP in U87	**2961**	GTF2E2	1.1103393	0.887594	2.158964	general transcription factor IIE, polypeptide 2, beta 34kDa
UP in U87	**8824**	CES2	1.4970088	0.9026615	2.822569	carboxylesterase 2
UP in U87	**11215**	AKAP11	1.2364647	0.8864917	2.356204	A kinase (PRKA) anchor protein 11
UP in U87	**153222**	CREBRF	1.6562762	0.8609526	3.152019	CREB3 regulatory factor
UP in U87	**8654**	PDE5A	2.830075	0.8109499	7.111111	phosphodiesterase 5A, cGMP-specific
UP in U87	**168455**	CCDC71L	3.865248	0.9794389	14.57322	coiled-coil domain containing 71-like
UP in U87	**116987**	AGAP1	1.0875288	0.8588814	2.125097	ArfGAP with GTPase domain, ankyrin repeat and PH domain 1
UP in U87	**7324**	UBE2E1	1.4670281	0.9059417	2.764518	ubiquitin-conjugating enzyme E2E 1
UP in U87	**100534595**	HNRNPU L2-BSCL2	1.1654302	0.8922343	2.243001	HNRNPUL2-BSCL2 readthrough
UP in U87	**26020**	LRP10	1.1322416	0.889683	2.191991	low density lipoprotein receptor-related protein 10
UP in U87	**5476**	CTSA	1.4902299	0.9081729	2.809337	cathepsin A
UP in U87	**7732**	RNF112	4.8464547	0.9830124	28.76923	ring finger protein 112
UP in U87	**23312**	DMXL2	1.1939855	0.8649795	2.287839	Dmx-like 2
UP in U87	**3687**	ITGAX	5.0761868	0.9468772	33.73529	integrin, alpha X (complement component 3 receptor 4 subunit)
UP in U87	**4597**	MVD	1.0906167	0.8799669	2.129651	mevalonate (diphospho) decarboxylase
UP in U87	**85027**	SMIM3	7.2187444	0.9984266	148.9562	small integral membrane protein 3
UP in U87	**5191**	PEX7	1.4560912	0.8423916	2.74364	peroxisomal biogenesis factor 7
UP in U87	**9352**	TXNL1	1.1283045	0.8908208	2.186017	thioredoxin-like 1
UP in U87	**51652**	CHMP3	1.2952318	0.8976034	2.454164	charged multivesicular body protein 3
UP in U87	**51200**	CPA4	3.0417727	0.9580867	8.235023	carboxypeptidase A4
UP in U87	**374928**	ZNF773	5.770829	0.9221114	54.6	zinc finger protein 773
UP in U87	**654463**	FER1L6	6.2779847	0.8198837	77.6	fer-1-like 6 (C. elegans)
UP in U87	**6609**	SMPD1	2.513395	0.9507974	5.709621	sphingomyelin phosphodiesterase 1, acid lysosomal
UP in U87	**80185**	TTI2	1.2985487	0.8717532	2.459813	TELO2 interacting protein 2
UP in U87	**10867**	TSPAN9	1.3031915	0.8381247	2.467742	tetraspanin 9
UP in U87	**1429**	CRYZ	1.1706651	0.8924298	2.251155	crystallin, zeta (quinone reductase)
UP in U87	**3611**	ILK	1.0111538	0.8865095	2.015522	integrin-linked kinase
UP in U87	**51527**	GSKIP	1.2871185	0.8913275	2.440402	GSK3B interacting protein
UP in U87	**203062**	TSNARE1	3.0552824	0.8247373	8.3125	t-SNARE domain containing 1
UP in U87	**3162**	HMOX1	5.0282782	0.9946397	32.63342	heme oxygenase (decycling) 1
UP in U87	**351**	APP	1.2247899	0.8989902	2.337214	amyloid beta (A4) precursor protein
UP in U87	**9127**	P2RX6	4.1852258	0.9621847	18.19192	purinergic receptor P2X, ligand-gated ion channel, 6
UP in U87	**57514**	ARHGAP3	5.053732	0.9772077	33.21429	Rho GTPase activating protein 31
UP in U87	**55851**	PSENEN	2.0902115	0.9408768	4.258105	presenilin enhancer 2 homolog (C. elegans)
UP in U87	**3108**	HLA-DMA	3.381681	0.9663893	10.42287	major histocompatibility complex, class II, DM alpha

UP in U87	**154761**	LOC154761	3.7985954	0.9026704	13.91525	family with sequence similarity 115, member C pseudogene
UP in U87	**56104**	PCDHGB1	3.0570309	0.8194926	8.322581	protocadherin gamma subfamily B, 1
UP in U87	**644**	BLVRA	1.1245661	0.8900652	2.18036	biliverdin reductase A
UP in U87	**83982**	IFI27L2	2.1924674	0.9420502	4.570866	interferon, alpha-inducible protein 27-like 2
UP in U87	**57616**	TSHZ3	9.6251	0.9833502	789.6667	teashirt zinc finger homeobox 3
UP in U87	**54972**	TMEM132	2.0901573	0.9411435	4.257945	transmembrane protein 132A
UP in U87	**4857**	NOVA1	2.0539259	0.8665973	4.152344	neuro-oncological ventral antigen 1
UP in U87	**4828**	NMB	1.5994212	0.9043149	3.030217	neuromedin B
UP in U87	**427**	ASAH1	1.3304732	0.8996124	2.514851	N-acylsphingosine amidohydrolase (acid ceramidase) 1
UP in U87	**51341**	ZBTB7A	1.1605713	0.8837982	2.235459	zinc finger and BTB domain containing 7A
UP in U87	**4499**	MT1M	8.937619	0.9673671	490.3333	metallothionein 1M
UP in U87	**84173**	ELMOD3	1.6040713	0.904866	3.04	ELMO/CED-12 domain containing 3
UP in U87	**11092**	C9orf9	2.2680511	0.8997458	4.81672	chromosome 9 open reading frame 9
UP in U87	**84899**	TMTC4	1.1760244	0.8431561	2.259533	transmembrane and tetratricopeptide repeat containing 4
UP in U87	**1435**	CSF1	1.5500863	0.8941188	2.928346	colony stimulating factor 1 (macrophage)
UP in U87	**9519**	TBPL1	1.054431	0.8795936	2.076899	TBP-like 1
UP in U87	**27338**	UBE2S	1.025701	0.8881807	2.035948	ubiquitin-conjugating enzyme E2S
UP in U87	**163049**	ZNF791	1.0123059	0.8409782	2.017133	zinc finger protein 791
UP in U87	**404636**	FAM45A	1.2088645	0.8901453	2.311556	family with sequence similarity 45, member
UP in U87	**1540**	CYLD	2.3743588	0.945446	5.185053	cylindromatosis (turban tumor syndrome)
UP in U87	**3075**	CFH	6.7504279	0.8989635	107.6667	complement factor H
UP in U87	**148932**	MOB3C	1.8958916	0.902857	3.721519	MOB kinase activator 3C
UP in U87	**5218**	CDK14	2.0676617	0.910573	4.192067	cyclin-dependent kinase 14
UP in U87	**285195**	SLC9A9	5.8172297	0.9112042	56.38462	solute carrier family 9, subfamily A (NHE9, cation proton antiporter 9), member 9
UP in U87	**114897**	C1QTNF1	2.9879357	0.9628869	7.93338	C1q and tumor necrosis factor related protein
UP in U87	**9162**	DGKI	8.4716752	0.9483617	355	diacylglycerol kinase, iota
UP in U87	**81493**	SYNC	1.136502	0.8906253	2.198473	syncoilin, intermediate filament protein
UP in U87	**90649**	ZNF486	6.9495349	0.8939143	123.6	zinc finger protein 486
UP in U87	**9819**	TSC22D2	1.1530421	0.8554412	2.223823	TSC22 domain family, member 2
UP in U87	**10381**	TUBB3	3.7116911	0.9822657	13.10178	tubulin, beta 3 class III
UP in U87	**8038**	ADAM12	3.5484366	0.9717052	11.7	ADAM metallopeptidase domain 12
UP in U87	**90**	ACVR1	1.5812606	0.9139243	2.992312	activin A receptor, type I
UP in U87	**79819**	WDR78	5.1979394	0.8890785	36.70588	WD repeat domain 78
UP in U87	**170622**	COMMD6	1.1924432	0.8902697	2.285395	COMM domain containing 6
UP in U87	**25797**	QPCT	7.377261	0.995342	166.2558	glutaminyl-peptide cyclotransferase
UP in U87	**26084**	ARHGEF2	4.3972162	0.8736999	21.07143	Rho guanine nucleotide exchange factor
UP in U87	**7764**	ZNF217	1.6238951	0.91716	3.08206	zinc finger protein 217
UP in U87	**9779**	TBC1D5	1.7985633	0.919009	3.478736	TBC1 domain family, member 5
UP in U87	**273**	AMPH	10.28193	0.9909951	1245	amphiphysin
UP in U87	**84542**	KIAA1841	1.3987409	0.8657795	2.636714	KIAA1841
UP in U87	**51422**	PRKAG2	3.3314091	0.9732519	10.06593	protein kinase, AMP-activated, gamma 2 non-catalytic subunit
UP in U87	**155382**	VPS37D	4.3260837	0.9335698	20.05769	vacuolar protein sorting 37 homolog D (S. cerevisiae)
UP in U87	**23096**	IQSEC2	1.5276664	0.8953011	2.883191	IQ motif and Sec7 domain 2
UP in U87	**3659**	IRF1	1.4653209	0.8856206	2.761249	interferon regulatory factor 1
UP in U87	**80019**	UBTD1	1.2704386	0.8898075	2.412349	ubiquitin domain containing 1
UP in U87	**55954**	ZMAT5	1.8252281	0.9226003	3.54363	zinc finger, matrin-type 5
UP in U87	**25790**	CCDC19	3.1874656	0.9072662	9.110092	coiled-coil domain containing 19
UP in U87	**3084**	NRG1	5.5681268	0.9951997	47.44311	neuregulin 1
UP in U87	**2887**	GRB10	1.9636354	0.9263872	3.900436	growth factor receptor-bound protein 10
UP in U87	**83992**	CTTNBP2	2.4874181	0.8818159	5.607735	cortactin binding protein 2
UP in U87	**2664**	GDI1	1.6116529	0.9228048	3.056018	GDP dissociation inhibitor 1

UP in U87	**54873**	PALMD	3.5357013	0.9755809	11.59717	palmdelphin
UP in U87	**9341**	VAMP3	1.5642769	0.9093818	2.957292	vesicle-associated membrane protein 3
UP in U87	**439**	ASNA1	1.0007132	0.8861361	2.000989	arsA arsenite transporter, ATP-binding, homolog 1 (bacterial)
UP in U87	**54981**	NMRK1	3.9903897	0.9773143	15.89377	nicotinamide riboside kinase 1
UP in U87	**374378**	GALNT18	4.0666453	0.9790211	16.75646	UDP-N-acetyl-alpha-D-galactosamine:polypeptide N-acetylgalactosaminyltransferase 18
UP in U87	**726**	CAPN5	7.1954601	0.9875371	146.5714	calpain 5
UP in U87	**157**	ADRBK2	2.4672276	0.9167244	5.529801	adrenergic, beta, receptor kinase 2
UP in U87	**2009**	EML1	4.0750172	0.9853414	16.85398	echinoderm microtubule associated protein
UP in U87	**22905**	EPN2	1.0951093	0.8832204	2.136293	epsin 2
UP in U87	**2627**	GATA6	2.1926451	0.845014	4.571429	GATA binding protein 6
UP in U87	**9209**	LRRFIP2	1.0603097	0.8817981	2.085379	leucine rich repeat (in FLII) interacting
UP in U87	**114926**	SMIM19	1.9298739	0.9225914	3.810219	small integral membrane protein 19
UP in U87	**79873**	NUDT18	1.8196459	0.8929721	3.529946	nudix (nucleoside diphosphate linked moiety X)-type motif 18
UP in U87	**9636**	ISG15	1.8493321	0.9216758	3.603333	ISG15 ubiquitin-like modifier
UP in U87	**26064**	RAI14	1.9536895	0.9290718	3.873639	retinoic acid induced 14
UP in U87	**84446**	BRSK1	5.4342041	0.980639	43.23729	BR serine/threonine kinase 1
UP in U87	**66000**	TMEM108	8.0714624	0.9248049	269	transmembrane protein 108
UP in U87	**55240**	STEAP3	1.3276794	0.9008303	2.509986	STEAP family member 3, metalloreductase
UP in U87	**4016**	LOXL1	2.3199903	0.9488417	4.993289	lysyl oxidase-like 1
UP in U87	**10006**	ABI1	1.6417002	0.9189557	3.120333	abl-interactor 1
UP in U87	**25903**	OLFML2B	6.0168083	0.9728608	64.75	olfactomedin-like 2B
UP in U87	**339122**	RAB43	3.9759708	0.9653759	15.73571	RAB43, member RAS oncogene family
UP in U87	**10098**	TSPAN5	9.7862696	0.9858215	883	tetraspanin 5
UP in U87	**141**	ADPRH	7.6088092	0.9414369	195.2	ADP-ribosylarginine hydrolase
UP in U87	**100131755**	ARMCX4	2.259903	0.9161022	4.789593	armadillo repeat containing, X-linked 4
UP in U87	**10758**	TRAF3IP2	1.2511247	0.8694153	2.380269	TRAF3 interacting protein 2
UP in U87	**972**	CD74	9.8898719	0.9996	948.7419	CD74 molecule, major histocompatibility complex, class II invariant chain
UP in U87	**53831**	GPR84	8.6911619	0.9583444	413.3333	G protein-coupled receptor 84
UP in U87	**150684**	COMMD1	1.1792442	0.8903586	2.264581	copper metabolism (Murr1) domain
UP in U87	**81576**	CCDC130	2.3146662	0.9381923	4.974895	coiled-coil domain containing 130
UP in U87	**10003**	NAALAD2	4.0071955	0.8057941	16.08	N-acetylated alpha-linked acidic dipeptidase
UP in U87	**100505679**	LOC100505679	4.3351169	0.9293829	20.18367	uncharacterized LOC100505679
UP in U87	**29800**	ZDHHC1	5.9929106	0.9863548	63.68627	zinc finger, DHHC-type containing 1
UP in U87	**51124**	IER3IP1	1.163577	0.8927854	2.240122	immediate early response 3 interacting
UP in U87	**3554**	IL1R1	5.4228718	0.9879994	42.89899	interleukin 1 receptor, type I
UP in U87	**140876**	FAM65C	8.8995248	0.9923107	477.5556	family with sequence similarity 65, member
UP in U87	**79783**	C7orf10	4.3006595	0.9101819	19.70732	chromosome 7 open reading frame 10
UP in U87	**64132**	XYLT2	1.6980111	0.9209024	3.244534	xylosyltransferase II
UP in U87	**65996**	MGC2752	1.0976849	0.8799047	2.14011	CENPB DNA-binding domains containing 1 pseudogene
UP in U87	**54899**	PXK	1.9994563	0.9322008	3.998493	PX domain containing serine/threonine
UP in U87	**83931**	STK40	2.1483351	0.9390901	4.433159	serine/threonine kinase 40
UP in U87	**8904**	CPNE1	1.249094	0.8990968	2.376921	copine I
UP in U87	**10908**	PNPLA6	1.8211691	0.9248849	3.533674	patatin-like phospholipase domain containing
UP in U87	**9747**	FAM115A	2.7762407	0.9581844	6.850649	family with sequence similarity 115, member
UP in U87	**116966**	WDR17	5.882643	0.8282842	59	WD repeat domain 17
UP in U87	**10346**	TRIM22	7.9637053	0.9949331	249.64	tripartite motif containing 22
UP in U87	**252969**	NEIL2	1.8495298	0.9192846	3.603827	nei endonuclease VIII-like 2 (E. coli)
UP in U87	**79183**	TTPAL	1.035001	0.8724376	2.049115	tocopherol (alpha) transfer protein-like
UP in U87	**23307**	FKBP15	1.0470186	0.881167	2.066255	FK506 binding protein 15, 133kDa
UP in U87	**55370**	PPP4R1L	2.5820092	0.9314719	5.98773	protein phosphatase 4, regulatory subunit 1-

UP in U87	**1390**	CREM	2.2555571	0.9350543	4.775187	cAMP responsive element modulator
UP in U87	**55902**	ACSS2	1.9271291	0.9210358	3.802977	acyl-CoA synthetase short-chain family member 2
UP in U87	**9788**	MTSS1	5.7235744	0.9874393	52.84058	metastasis suppressor 1
UP in U87	**1959**	EGR2	4.9412107	0.8697442	30.72222	early growth response 2
UP in U87	**89797**	NAV2	1.2817621	0.891932	2.431358	neuron navigator 2
UP in U87	**113146**	AHNAK2	1.5937647	0.9199068	3.01836	AHNAK nucleoprotein 2
UP in U87	**5119**	CHMP1A	1.6190755	0.9223781	3.071781	charged multivesicular body protein 1A
UP in U87	**9246**	UBE2L6	8.7297017	0.9988355	424.5238	ubiquitin-conjugating enzyme E2L 6
UP in U87	**80045**	GPR157	1.3571681	0.8359024	2.561818	G protein-coupled receptor 157
UP in U87	**29919**	C18orf8	2.1832668	0.9367344	4.541808	chromosome 18 open reading frame 8
UP in U87	**55357**	TBC1D2	3.655814	0.9771454	12.60404	TBC1 domain family, member 2
UP in U87	**25800**	SLC39A6	1.2707347	0.8969545	2.412844	solute carrier family 39 (zinc transporter), member 6
UP in U87	**54843**	SYTL2	6.8399911	0.974852	114.5625	synaptotagmin-like 2
UP in U87	**4207**	MEF2BNB-MEF2B	1.8299711	0.8800292	3.5553	MEF2BNB-MEF2B readthrough
UP in U87	**1501**	CTNND2	7.3007339	0.8552723	157.6667	catenin (cadherin-associated protein), delta 2
UP in U87	**130557**	ZNF513	1.4381771	0.8771757	2.709783	zinc finger protein 513
UP in U87	**25895**	METTL21	1.6621655	0.8831493	3.164912	methyltransferase like 21B
UP in U87	**22917**	ZP1	7.9657843	0.9175512	250	zona pellucida glycoprotein 1 (sperm
UP in U87	**3678**	ITGA5	3.9277952	0.985377	15.21893	integrin, alpha 5 (fibronectin receptor, alpha polypeptide)
UP in U87	**441282**	AKR1B15	5.2214012	0.851441	37.30769	aldo-keto reductase family 1, member B15
UP in U87	**10947**	AP3M2	1.2613611	0.8761712	2.397218	adaptor-related protein complex 3, mu 2
UP in U87	**2992**	GYG1	1.6992697	0.9219603	3.247365	glycogenin 1
UP in U87	**29109**	FHOD1	1.3699335	0.8985457	2.584586	formin homology 2 domain containing 1
UP in U87	**23743**	BHMT2	3.9380696	0.9777944	15.3277	betaine--homocysteine S-methyltransferase 2
UP in U87	**51257**	2-Mar	2.6593503	0.950184	6.317485	membrane-associated ring finger (C3HC4) 2, E3 ubiquitin protein ligase
UP in U87	**60592**	SCOC	8.5875097	0.9973954	384.6786	short coiled-coil protein
UP in U87	**5663**	PSEN1	1.0695809	0.8677441	2.098824	presenilin 1
UP in U87	**51507**	RTFDC1	1.1356682	0.8936477	2.197203	replication termination factor 2 domain containing 1
UP in U87	**131601**	TPRA1	1.4631644	0.9048127	2.757124	transmembrane protein, adipocyte
UP in U87	**3797**	KIF3C	2.6486529	0.9577311	6.270815	kinesin family member 3C
UP in U87	**10397**	NDRG1	1.2033352	0.8960656	2.302714	N-myc downstream regulated 1
UP in U87	**26167**	PCDHB5	7.5824556	0.8856739	191.6667	protocadherin beta 5
UP in U87	**5899**	RALB	1.423871	0.9027593	2.683045	v-ral simian leukemia viral oncogene homolog B (ras related; GTP binding protein)
UP in U87	**29942**	PURG	7.7725895	0.9023859	218.6667	purine-rich element binding protein G
UP in U87	**2530**	FUT8	2.7661881	0.9546554	6.80308	fucosyltransferase 8 (alpha (1,6) fucosyltransferase)
UP in U87	**2946**	GSTM2	4.7918453	0.986257	27.7006	glutathione S-transferase mu 2 (muscle)
UP in U87	**10678**	B3GNT2	1.2359165	0.8827049	2.355309	UDP-GlcNAc:betaGal beta-1,3-N-acetylglucosaminyltransferase 2
UP in U87	**53349**	ZFYVE1	2.3933805	0.9388501	5.25387	zinc finger, FYVE domain containing 1
UP in U87	**7711**	ZNF155	1.6643653	0.8146034	3.169742	zinc finger protein 155
UP in U87	**9605**	VPS9D1	2.8677757	0.9537931	7.299389	VPS9 domain containing 1
UP in U87	**1687**	DFNA5	1.9028377	0.9258449	3.73948	deafness, autosomal dominant 5
UP in U87	**149111**	CNIH3	9.716207	0.9949064	841.1429	cornichon homolog 3 (Drosophila)
UP in U87	**11274**	USP18	1.5415183	0.8365068	2.911007	ubiquitin specific peptidase 18
UP in U87	**83604**	TMEM47	7.9195255	0.9808701	242.1111	transmembrane protein 47
UP in U87	**23406**	COTL1	1.0567838	0.8887141	2.080289	coactosin-like 1 (Dictyostelium)
UP in U87	**771**	CA12	2.759926	0.9593134	6.773615	carbonic anhydrase XII

UP in U87	**8706**	B3GALNT 1	1.5989791	0.8652017	3.029289	beta-1,3-N-acetylgalactosaminyltransferase 1 (globoside blood group)
UP in U87	**2555**	GABRA2	7.8662486	0.909773	233.3333	gamma-aminobutyric acid (GABA) A receptor, alpha 2
UP in U87	**56605**	ERO1LB	4.3241123	0.8882874	20.0303	ERO1-like beta (S. cerevisiae)
UP in U87	**5627**	PROS1	1.2001967	0.8866251	2.29771	protein S (alpha)
UP in U87	**113451**	ADC	4.5631181	0.9563621	23.63934	arginine decarboxylase
UP in U87	**2697**	GJA1	8.5606243	0.9991466	377.5763	gap junction protein, alpha 1, 43kDa
UP in U87	**23237**	ARC	4.8147724	0.9795811	28.14433	activity-regulated cytoskeleton-associated protein
UP in U87	**284385**	LOC28438	4.8524428	0.9383967	28.88889	uncharacterized LOC284385
UP in U87	**4650**	MYO9B	1.6536858	0.9201913	3.146364	myosin IXB
UP in U87	**23231**	SEL1L3	2.3074467	0.9406813	4.950062	sel-1 suppressor of lin-12-like 3 (C. elegans)
UP in U87	**83719**	YPEL3	1.4564324	0.8910253	2.744289	yippee-like 3 (Drosophila)
UP in U87	**26056**	RAB11FIP	3.6442621	0.9787189	12.50352	RAB11 family interacting protein 5 (class I)
UP in U87	**115123**	3-Mar	5.3365479	0.9445393	40.40741	membrane-associated ring finger (C3HC4) 3, E3 ubiquitin protein ligase
UP in U87	**7040**	TGFB1	1.7146287	0.9241204	3.282122	transforming growth factor, beta 1
UP in U87	**6002**	RGS12	1.9310526	0.8777357	3.813333	regulator of G-protein signaling 12
UP in U87	**57600**	FNIP2	1.0351116	0.8282397	2.049272	folliculin interacting protein 2
UP in U87	**84221**	SPATC1L	2.2105373	0.9318719	4.628476	spermatogenesis and centriole associated 1-
UP in U87	**64167**	ERAP2	6.0792796	0.9901773	67.61538	endoplasmic reticulum aminopeptidase 2
UP in U87	**23671**	TMEFF2	8.4607972	0.9478106	352.3333	transmembrane protein with EGF-like and two follistatin-like domains 2
UP in U87	**56128**	PCDHB8	6	0.9372144	64	protocadherin beta 8
UP in U87	**152007**	GLIPR2	1.2422271	0.8635749	2.365634	GLI pathogenesis-related 2
UP in U87	**6505**	SLC1A1	5.2123036	0.9880883	37.07317	solute carrier family 1 (neuronal/epithelial high affinity glutamate transporter, system Xag), member 1
UP in U87	**8682**	PEA15	3.6341729	0.9808523	12.41638	phosphoprotein enriched in astrocytes 15
UP in U87	**138050**	HGSNAT	2.1315532	0.9272139	4.38189	heparan-alpha-glucosaminide N-
UP in U87	**55002**	TMCO3	2.1977326	0.9403968	4.587578	transmembrane and coiled-coil domains 3
UP in U87	**90874**	ZNF697	2.7853496	0.9463794	6.89404	zinc finger protein 697
UP in U87	**9586**	CREB5	3.0632778	0.8766512	8.358696	cAMP responsive element binding protein 5
UP in U87	**441212**	RP9P	1.5813807	0.9003325	2.992561	retinitis pigmentosa 9 pseudogene
UP in U87	**3212**	HOXB2	8.2469102	0.9920796	303.7857	homeobox B2
UP in U87	**115290**	FBXO17	1.5521173	0.9030348	2.932472	F-box protein 17
UP in U87	**342184**	FMN1	3.5292531	0.9472861	11.54545	formin 1
UP in U87	**64753**	CCDC136	4.9010034	0.989475	29.87783	coiled-coil domain containing 136
UP in U87	**79654**	HECTD3	1.3367982	0.8991324	2.525901	HECT domain containing E3 ubiquitin protein ligase 3
UP in U87	**55757**	UGGT2	1.2210062	0.8699308	2.331092	UDP-glucose glycoprotein
UP in U87	**4327**	MMP19	3.1362657	0.9590556	8.792453	matrix metallopeptidase 19
UP in U87	**3257**	HPS1	1.0130882	0.8821448	2.018227	Hermansky-Pudlak syndrome 1
UP in U87	**81562**	LMAN2L	1.3759785	0.8941277	2.595439	lectin, mannose-binding 2-like
UP in U87	**56113**	PCDHGA2	5.3339007	0.8024606	40.33333	protocadherin gamma subfamily A, 2
UP in U87	**84056**	KATNAL1	1.0267632	0.8068786	2.037448	katanin p60 subunit A-like 1
UP in U87	**89894**	TMEM116	2.3540573	0.9213647	5.112601	transmembrane protein 116
UP in U87	**123355**	LRRC28	1.2072271	0.8555212	2.308934	leucine rich repeat containing 28
UP in U87	**9249**	DHRS3	2.9528618	0.9622202	7.742834	dehydrogenase/reductase (SDR family)
UP in U87	**285237**	C3orf38	1.2445775	0.8712554	2.369492	chromosome 3 open reading frame 38
UP in U87	**81790**	RNF170	1.3195639	0.8638861	2.495906	ring finger protein 170
UP in U87	**29906**	ST8SIA5	6.4321048	0.9890839	86.34884	ST8 alpha-N-acetyl-neuraminide alpha-2,8-sialyltransferase 5
UP in U87	**5819**	PVRL2	1.4637476	0.9038971	2.758239	poliovirus receptor-related 2 (herpesvirus entry mediator B)
UP in U87	**94134**	ARHGAP1	1.277995	0.8808114	2.425017	Rho GTPase activating protein 12

UP in U87	**1200**	TPP1	1.3680356	0.9031859	2.581189	tripeptidyl peptidase I
UP in U87	**8406**	SRPX	6.7144106	0.9982221	105.012	sushi-repeat containing protein, X-linked
UP in U87	**84141**	EVA1A	6.8136285	0.9959287	112.4881	eva-1 homolog A (C. elegans)
UP in U87	**1371**	CPOX	1.3208089	0.8957811	2.498061	coproporphyrinogen oxidase
UP in U87	**55219**	TMEM57	1.1090082	0.8695042	2.156973	transmembrane protein 57
UP in U87	**121551**	BTBD11	8.8610869	0.9646026	465	BTB (POZ) domain containing 11
UP in U87	**80212**	CCDC92	4.4539707	0.987306	21.91688	coiled-coil domain containing 92
UP in U87	**118433**	RPL23AP7	1.7069495	0.9222892	3.264698	ribosomal protein L23a pseudogene 7
UP in U87	**4130**	MAP1A	5.9509399	0.9969776	61.86022	microtubule-associated protein 1A
UP in U87	**84912**	SLC35B4	1.1592419	0.8837538	2.2334	solute carrier family 35, member B4
UP in U87	**5872**	RAB13	1.8570019	0.9282806	3.622541	RAB13, member RAS oncogene family
UP in U87	**826**	CAPNS1	1.1700395	0.8963056	2.250179	calpain, small subunit 1
UP in U87	**140686**	WFDC3	5.0342699	0.8293153	32.76923	WAP four-disulfide core domain 3
UP in U87	**57097**	PARP11	4.3174126	0.8838783	19.9375	poly (ADP-ribose) polymerase family,
UP in U87	**23612**	PHLDA3	2.8733185	0.9649048	7.327487	pleckstrin homology-like domain, family A, member 3
UP in U87	**8972**	MGAM	7.7021727	0.9274895	208.25	maltase-glucoamylase (alpha-glucosidase)
UP in U87	**284454**	LOC28445	1.1441192	0.8856472	2.210112	uncharacterized LOC284454
UP in U87	**258010**	SVIP	1.4528341	0.9019237	2.737453	small VCP/p97-interacting protein
UP in U87	**51226**	COPZ2	5.5198123	0.9949775	45.8806	coatomer protein complex, subunit zeta 2
UP in U87	**9120**	SLC16A6	3.2942665	0.9712251	9.810091	solute carrier family 16, member 6 (monocarboxylic acid transporter 7)
UP in U87	**51330**	TNFRSF12 A	1.2036271	0.8966167	2.30318	tumor necrosis factor receptor superfamily, member 12A
UP in U87	**5768**	QSOX1	1.2111209	0.8945455	2.315175	quiescin Q6 sulfhydryl oxidase 1
UP in U87	**2057**	EPOR	1.8883546	0.9157822	3.702128	erythropoietin receptor
UP in U87	**10567**	RABAC1	1.7428222	0.9260761	3.346892	Rab acceptor 1 (prenylated)
UP in U87	**10640**	EXOC5	1.1190966	0.8816915	2.172109	exocyst complex component 5
UP in U87	**115207**	KCTD12	3.6751286	0.9672338	12.77391	potassium channel tetramerisation domain containing 12
UP in U87	**7799**	PRDM2	1.2572395	0.8688819	2.390379	PR domain containing 2, with ZNF domain
UP in U87	**23428**	SLC7A8	2.1216637	0.8988657	4.351955	solute carrier family 7 (amino acid transporter light chain, L system), member 8
UP in U87	**642819**	ZNF487P	2.0835647	0.8532988	4.238532	zinc finger protein 487, pseudogene
UP in U87	**63901**	FAM111A	1.5914149	0.9126887	3.013447	family with sequence similarity 111, member
UP in U87	**26575**	RGS17	7.7909458	0.9890839	221.4667	regulator of G-protein signaling 17
UP in U87	**1837**	DTNA	1.2548504	0.890732	2.386424	dystrobrevin, alpha
UP in U87	**478**	ATP1A3	2.5266495	0.9255071	5.762319	ATPase, Na+/K+ transporting, alpha 3 polypeptide
UP in U87	**92737**	DNER	3.9726408	0.9853326	15.69944	delta/notch-like EGF repeat containing
UP in U87	**54776**	PPP1R12C	1.8998012	0.9266716	3.731618	protein phosphatase 1, regulatory subunit
UP in U87	**9262**	STK17B	1.115126	0.8611393	2.166139	serine/threonine kinase 17b
UP in U87	**23024**	PDZRN3	7.587965	0.9929685	192.4	PDZ domain containing ring finger 3
UP in U87	**100127983**	LOC10012 7983	3.2192815	0.9654915	9.31323	uncharacterized LOC100127983
UP in U87	**91409**	CCDC74B	5.4614377	0.9766032	44.06122	coiled-coil domain containing 74B
UP in U87	**84803**	AGPAT9	3.0919683	0.9621224	8.526587	1-acylglycerol-3-phosphate O-acyltransferase
UP in U87	**54734**	RAB39A	7.7142455	0.8977012	210	RAB39A, member RAS oncogene family
UP in U87	**55093**	WDYHV1	1.2001719	0.8660729	2.29767	WDYHV motif containing 1
UP in U87	**6618**	SNAPC2	2.6573921	0.9581044	6.308916	small nuclear RNA activating complex, polypeptide 2, 45kDa
UP in U87	**11047**	ADRM1	1.30008	0.901257	2.462425	adhesion regulating molecule 1
UP in U87	**283050**	ZMIZ1-	7.1437311	0.9978399	141.4091	ZMIZ1 antisense RNA 1
UP in U87	**23017**	FAIM2	6.2524402	0.9685405	76.2381	Fas apoptotic inhibitory molecule 2
UP in U87	**6819**	SULT1C2	6.2384047	0.9155955	75.5	sulfotransferase family, cytosolic, 1C,
UP in U87	**5552**	SRGN	1.9527088	0.9232315	3.871007	serglycin
UP in U87	**30845**	EHD3	7.1623913	0.986337	143.25	EH-domain containing 3

UP in U87	**55841**	WWC3	2.1113706	0.9080573	4.321016	WWC family member 3
UP in U87	**57406**	ABHD6	1.784221	0.9094618	3.444324	abhydrolase domain containing 6
UP in U87	**339665**	SLC35E4	2.051723	0.928814	4.146008	solute carrier family 35, member E4
UP in U87	**339768**	ESPNL	2.7690934	0.8804381	6.816794	espin-like
UP in U87	**54852**	PAQR5	3.1689854	0.9650115	8.994141	progestin and adipoQ receptor family
UP in U87	**29767**	TMOD2	2.8479969	0.9289562	7.2	tropomodulin 2 (neuronal)
UP in U87	**55957**	LIN37	1.4697574	0.8949455	2.769753	lin-37 homolog (C. elegans)
UP in U87	**79734**	KCTD17	1.6520347	0.9140665	3.142766	potassium channel tetramerisation domain containing 17
UP in U87	**388662**	SLC6A17	8.1050355	0.9269383	275.3333	solute carrier family 6, member 17
UP in U87	**27241**	BBS9	1.6005678	0.8822959	3.032626	Bardet-Biedl syndrome 9
UP in U87	**5880**	RAC2	12.459912	0.9999556	5633.875	ras-related C3 botulinum toxin substrate 2 (rho family, small GTP binding protein Rac2)
UP in U87	**79729**	SH3D21	1.9896581	0.8693264	3.971429	SH3 domain containing 21
UP in U87	**55325**	UFSP2	1.4166285	0.8686508	2.669609	UFM1-specific peptidase 2
UP in U87	**25809**	TTLL1	2.9761345	0.9165911	7.86875	tubulin tyrosine ligase-like family, member 1
UP in U87	**388115**	C15orf52	1.6323098	0.9171422	3.100089	chromosome 15 open reading frame 52
UP in U87	**80856**	KIAA1715	1.286639	0.8817804	2.43959	KIAA1715
UP in U87	**64430**	PCNXL4	1.3178173	0.8926254	2.492887	pecanex-like 4 (Drosophila)
UP in U87	**27031**	NPHP3	2.3420858	0.9235781	5.070352	nephronophthisis 3 (adolescent)
UP in U87	**22902**	RUFY3	2.3458255	0.9296496	5.083512	RUN and FYVE domain containing 3
UP in U87	**541468**	LURAP1	6.7999754	0.9203424	111.4286	leucine rich adaptor protein 1
UP in U87	**123879**	DCUN1D3	2.048363	0.9248404	4.136364	DCN1, defective in cullin neddylation 1, domain containing 3 (S. cerevisiae)
UP in U87	**4097**	MAFG	1.0605004	0.8822959	2.085655	v-maf musculoaponeurotic fibrosarcoma oncogene homolog G (avian)
UP in U87	**285971**	ZNF775	2.0975165	0.9069461	4.27972	zinc finger protein 775
UP in U87	**4323**	MMP14	11.109528	0.9999022	2209.536	matrix metallopeptidase 14 (membrane-
UP in U87	**11343**	MGLL	4.2346104	0.9880616	18.82542	monoglyceride lipase
UP in U87	**84947**	SERAC1	1.3275472	0.8002916	2.509756	serine active site containing 1
UP in U87	**133022**	TRAM1L1	7.0149503	0.8208882	129.3333	translocation associated membrane protein 1-like 1
UP in U87	**64863**	METTL4	1.9912048	0.9066439	3.975689	methyltransferase like 4
UP in U87	**219285**	SAMD9L	7.9565214	0.9581222	248.4	sterile alpha motif domain containing 9-like
UP in U87	**6385**	SDC4	1.7800571	0.9258983	3.434398	syndecan 4
UP in U87	**79858**	NEK11	2.4989893	0.8372802	5.652893	NIMA-related kinase 11
UP in U87	**93611**	FBXO44	1.4222698	0.8851228	2.680068	F-box protein 44
UP in U87	**1033**	CDKN3	1.0727928	0.887114	2.103501	cyclin-dependent kinase inhibitor 3
UP in U87	**57608**	KIAA1462	6.8047764	0.8804647	111.8	KIAA1462
UP in U87	**116496**	FAM129A	2.1643141	0.9309208	4.482533	family with sequence similarity 129, member
UP in U87	**56623**	INPP5E	1.0169907	0.8558768	2.023693	inositol polyphosphate-5-phosphatase, 72
UP in U87	**7052**	TGM2	8.5700629	0.9998311	380.0546	transglutaminase 2 (C polypeptide, protein-glutamine-gamma-glutamyltransferase)
UP in U87	**51296**	SLC15A3	7.101538	0.8313154	137.3333	solute carrier family 15, member 3
UP in U87	**4779**	NFE2L1	1.0398758	0.887834	2.056051	nuclear factor (erythroid-derived 2)-like 1
UP in U87	**729178**	STXBP5-AS1	2.6981731	0.8367024	6.489796	STXBP5 antisense RNA 1
UP in U87	**8650**	NUMB	2.9169643	0.9646292	7.552553	numb homolog (Drosophila)
UP in U87	**3884**	KRT33B	7.0516621	0.8262396	132.6667	keratin 33B
UP in U87	**80206**	FHOD3	8.4444973	0.9943819	348.375	formin homology 2 domain containing 3
UP in U87	**22885**	ABLIM3	6.8568419	0.9977332	115.9085	actin binding LIM protein family, member 3
UP in U87	**60681**	FKBP10	1.4063917	0.9046349	2.650734	FK506 binding protein 10, 65 kDa
UP in U87	**353376**	TICAM2	9.1857014	0.974372	582.3333	toll-like receptor adaptor molecule 2
UP in U87	**6909**	TBX2	6.1739894	0.9908528	72.20313	T-box 2
UP in U87	**51657**	STYXL1	2.9253792	0.9597756	7.596734	serine/threonine/tyrosine interacting-like 1
UP in U87	**2686**	GGT7	3.3832576	0.9713585	10.43427	gamma-glutamyltransferase 7
UP in U87	**3234**	HOXD8	3.0907741	0.9475705	8.519531	homeobox D8

UP in U87	**8870**	IER3	2.0276135	0.9405213	4.077298	immediate early response 3
UP in U87	**6591**	SNAI2	3.7055879	0.9818746	13.04647	snail homolog 2 (Drosophila)
UP in U87	**29958**	DMGDH	7.3634047	0.8624993	164.6667	dimethylglycine dehydrogenase
UP in U87	**196410**	METTL7B	3.8868937	0.9738653	14.79352	methyltransferase like 7B
UP in U87	**9456**	HOMER1	1.7913426	0.8794069	3.461369	homer homolog 1 (Drosophila)
UP in U87	**79939**	SLC35E1	1.1808122	0.889283	2.267044	solute carrier family 35, member E1
UP in U87	**25966**	C2CD2	3.0168133	0.9658115	8.093778	C2 calcium-dependent domain containing 2
UP in U87	**54828**	BCAS3	1.2612413	0.8472363	2.397019	breast carcinoma amplified sequence 3
UP in U87	**22990**	PCNX	1.8862752	0.9183068	3.696795	pecanex homolog (Drosophila)
UP in U87	**5569**	PKIA	9.7284874	0.9850392	848.3333	protein kinase (cAMP-dependent, catalytic) inhibitor alpha
UP in U87	**255043**	TMEM86B	2.5571388	0.9348499	5.885393	transmembrane protein 86B
UP in U87	**85441**	HELZ2	3.7097284	0.9532242	13.08397	helicase with zinc finger 2, transcriptional coactivator
UP in U87	**64089**	SNX16	1.5911411	0.8626327	3.012876	sorting nexin 16
UP in U87	**9980**	DOPEY2	2.5870162	0.9152844	6.008547	dopey family member 2
UP in U87	**221061**	FAM171A	6.0347748	0.9884527	65.5614	family with sequence similarity 171, member
UP in U87	**307**	ANXA4	1.1736392	0.891852	2.2558	annexin A4
UP in U87	**57577**	KIAA1407	4.2439256	0.8963678	18.94737	KIAA1407
UP in U87	**8863**	PER3	5.7799017	0.9389923	54.94444	period circadian clock 3
UP in U87	**57659**	ZBTB4	1.0100149	0.8792736	2.013932	zinc finger and BTB domain containing 4
UP in U87	**79827**	CLMP	2.198048	0.9402368	4.588581	CXADR-like membrane protein
UP in U87	**3455**	IFNAR2	1.0899814	0.8745533	2.128713	interferon (alpha, beta and omega) receptor 2
UP in U87	**55273**	TMEM100	2.0136849	0.8875673	4.038123	transmembrane protein 100
UP in U87	**122961**	ISCA2	1.2532123	0.8887941	2.383716	iron-sulfur cluster assembly 2 homolog (S. cerevisiae)
UP in U87	**10589**	DRAP1	2.7773665	0.9602734	6.855997	DR1-associated protein 1 (negative cofactor 2 alpha)
UP in U87	**64208**	POPDC3	5.4460774	0.9936708	43.59459	popeye domain containing 3
UP in U87	**91050**	CCDC149	9.4079758	0.9794567	679.3333	coiled-coil domain containing 149
UP in U87	**253782**	CERS6	2.1505084	0.9322453	4.439842	ceramide synthase 6
UP in U87	**79676**	OGFOD2	1.0125453	0.8596636	2.017467	2-oxoglutarate and iron-dependent oxygenase domain containing 2
UP in U87	**10668**	CGRRF1	1.5126667	0.8886252	2.85337	cell growth regulator with ring finger domain
UP in U87	**51191**	HERC5	4.0136564	0.8968567	16.15217	HECT and RLD domain containing E3 ubiquitin protein ligase 5
UP in U87	**11037**	STON1	3.6322682	0.8322133	12.4	stonin 1
UP in U87	**147906**	DACT3	6.4644672	0.9517041	88.30769	dapper, antagonist of beta-catenin, homolog 3 (Xenopus laevis)
UP in U87	**11228**	RASSF8	1.5865398	0.9156222	3.003282	Ras association (RalGDS/AF-6) domain family (N-terminal) member 8
UP in U87	**79589**	RNF128	9.6354158	0.9835102	795.3333	ring finger protein 128, E3 ubiquitin protein
UP in U87	**1730**	DIAPH2	7.5077946	0.9752253	182	diaphanous homolog 2 (Drosophila)
UP in U87	**283554**	GPR137C	3.0628008	0.81279	8.355932	G protein-coupled receptor 137C
UP in U87	**1316**	KLF6	1.2869386	0.8962434	2.440097	Kruppel-like factor 6
UP in U87	**4163**	MCC	4.9258425	0.9846925	30.39669	mutated in colorectal cancers
UP in U87	**79705**	LRRK1	5.7142455	0.8932032	52.5	leucine-rich repeat kinase 1
UP in U87	**83943**	IMMP2L	1.293574	0.8129056	2.451346	IMP2 inner mitochondrial membrane peptidase-like (S. cerevisiae)
UP in U87	**51135**	IRAK4	1.1209321	0.8489431	2.174874	interleukin-1 receptor-associated kinase 4
UP in U87	**51065**	RPS27L	2.1801027	0.9422191	4.531858	ribosomal protein S27-like
UP in U87	**84283**	TMEM79	1.7024988	0.8990613	3.254642	transmembrane protein 79
UP in U87	**5959**	RDH5	3.3950628	0.9485573	10.52	retinol dehydrogenase 5 (11-cis/9-cis)
UP in U87	**2580**	GAK	1.6134784	0.9205202	3.059887	cyclin G associated kinase
UP in U87	**55384**	MEG3	11.270101	0.996142	2469.667	maternally expressed 3 (non-protein coding)
UP in U87	**10791**	VAMP5	4.9158862	0.9923018	30.18764	vesicle-associated membrane protein 5
UP in U87	**1291**	COL6A1	6.3311393	0.9979554	80.51241	collagen, type VI, alpha 1

UP in U87	**27330**	RPS6KA6	4.5684744	0.8579035	23.72727	ribosomal protein S6 kinase, 90kDa,
UP in U87	**2629**	GBA	1.1384765	0.8931054	2.201484	glucosidase, beta, acid
UP in U87	**26872**	STEAP1	8.8217257	0.9984355	452.4848	six transmembrane epithelial antigen of the prostate 1
UP in U87	**5799**	PTPRN2	9.8630247	0.9909773	931.25	protein tyrosine phosphatase, receptor type, N polypeptide 2
UP in U87	**401548**	SNX30	1.5799444	0.8773979	2.989583	sorting nexin family member 30
UP in U87	**83658**	DYNLRB1	2.0738175	0.9411346	4.209992	dynein, light chain, roadblock-type 1
UP in U87	**2207**	FCER1G	3.3561438	0.8856828	10.24	Fc fragment of IgE, high affinity I, receptor for; gamma polypeptide
UP in U87	**5283**	PIGH	1.4760696	0.8948833	2.781898	phosphatidylinositol glycan anchor biosynthesis, class H
UP in U87	**91445**	RNF185	1.1601252	0.8863939	2.234768	ring finger protein 185
UP in U87	**10126**	DNAL4	2.6473072	0.951953	6.264968	dynein, axonemal, light chain 4
UP in U87	**79870**	BAALC	11.792519	0.9975376	3547.333	brain and acute leukemia, cytoplasmic
UP in U87	**5333**	PLCD1	3.6812886	0.9609313	12.82857	phospholipase C, delta 1
UP in U87	**283788**	LOC28378	2.2665481	0.9117464	4.811705	FSHD region gene 1 pseudogene
UP in U87	**5764**	PTN	3.7137678	0.9775988	13.12065	pleiotrophin
UP in U87	**2920**	CXCL2	6.1523836	0.9971465	71.12987	chemokine (C-X-C motif) ligand 2
UP in U87	**81553**	FAM49A	6.6351739	0.977101	99.4	family with sequence similarity 49, member
UP in U87	**8614**	STC2	1.1615775	0.8918609	2.237019	stanniocalcin 2
UP in U87	**9900**	SV2A	2.9400615	0.9615268	7.67444	synaptic vesicle glycoprotein 2A
UP in U87	**84645**	C22orf23	3.5473482	0.9600779	11.69118	chromosome 22 open reading frame 23
UP in U87	**10333**	TLR6	2.107261	0.8049496	4.308725	toll-like receptor 6
UP in U87	**83478**	ARHGAP2	4.0144705	0.8428183	16.16129	Rho GTPase activating protein 24
UP in U87	**597**	BCL2A1	10.793332	0.9943464	1774.667	BCL2-related protein A1
UP in U87	**3484**	IGFBP1	7.961932	0.9172489	249.3333	insulin-like growth factor binding protein 1
UP in U87	**26994**	RNF11	1.0581725	0.8856739	2.082292	ring finger protein 11
UP in U87	**135**	ADORA2	4.7146071	0.9846481	26.25658	adenosine A2a receptor
UP in U87	**203228**	C9orf72	1.0431247	0.8464451	2.060686	chromosome 9 open reading frame 72
UP in U87	**283229**	EFCAB4A	2.0691711	0.9224581	4.196455	EF-hand calcium binding domain 4A
UP in U87	**6919**	TCEA2	2.157276	0.9373122	4.460718	transcription elongation factor A (SII), 2
UP in U87	**8601**	RGS20	4.5268513	0.9889239	23.0525	regulator of G-protein signaling 20
UP in U87	**3597**	IL13RA1	1.7698486	0.9244849	3.410182	interleukin 13 receptor, alpha 1
UP in U87	**100506783**	HOXD-	9.4580648	0.9803634	703.3333	HOXD cluster antisense RNA 2
UP in U87	**4824**	NKX3-1	2.3141249	0.9213025	4.973029	NK3 homeobox 1
UP in U87	**60401**	EDA2R	3.6027463	0.9646381	12.14884	ectodysplasin A2 receptor
UP in U87	**51643**	TMBIM4	1.6244048	0.9183868	3.083149	transmembrane BAX inhibitor motif
UP in U87	**55701**	ARHGEF4	1.5286389	0.8962878	2.885135	Rho guanine nucleotide exchange factor
UP in U87	**7485**	WRB	1.2836882	0.8962789	2.434606	tryptophan rich basic protein
UP in U87	**9404**	LPXN	6.5931204	0.9971198	96.54438	leupaxin
UP in U87	**9975**	NR1D2	2.4560912	0.9459082	5.48728	nuclear receptor subfamily 1, group D,
UP in U87	**10000**	AKT3	2.5866899	0.955082	6.007188	v-akt murine thymoma viral oncogene homolog 3 (protein kinase B, gamma)
UP in U87	**2668**	GDNF	10.277287	0.9909773	1241	glial cell derived neurotrophic factor
UP in U87	**100534599**	ISY1-	2.3343819	0.9382367	5.043348	ISY1-RAB43 readthrough
UP in U87	**6474**	SHOX2	2.499881	0.9337387	5.656388	short stature homeobox 2
UP in U87	**84304**	NUDT22	1.3351291	0.900528	2.522981	nudix (nucleoside diphosphate linked moiety X)-type motif 22
UP in U87	**79877**	DCAKD	1.395544	0.8980835	2.630877	dephospho-CoA kinase domain containing
UP in U87	**10752**	CHL1	6.8392038	0.9063683	114.5	cell adhesion molecule with homology to L1CAM (close homolog of L1)
UP in U87	**91300**	R3HDM4	1.2097228	0.8894074	2.312932	R3H domain containing 4
UP in U87	**10087**	COL4A3BP	1.136109	0.8781446	2.197874	collagen, type IV, alpha 3 (Goodpasture antigen) binding protein
UP in U87	**54661**	PCDHB17	6.882643	0.8038473	118	protocadherin beta 17 pseudogene

UP in U87	**79414**	LRFN3	1.6705382	0.8724021	3.183333	leucine rich repeat and fibronectin type III domain containing 3
UP in U87	**23764**	MAFF	4.6348654	0.9893328	24.84469	v-maf musculoaponeurotic fibrosarcoma oncogene homolog F (avian)
UP in U87	**64420**	SUSD1	1.5026388	0.861726	2.833605	sushi domain containing 1
UP in U87	**128869**	PIGU	1.1765788	0.8891585	2.260401	phosphatidylinositol glycan anchor biosynthesis, class U
UP in U87	**3656**	IRAK2	3.7110713	0.9806745	13.09615	interleukin-1 receptor-associated kinase 2
UP in U87	**134728**	IRAK1BP1	3.7091745	0.8348267	13.07895	interleukin-1 receptor-associated kinase 1 binding protein 1
UP in U87	**154881**	KCTD7	1.0834331	0.8401959	2.119073	potassium channel tetramerisation domain containing 7
UP in U87	**7357**	UGCG	1.6343983	0.9180312	3.10458	UDP-glucose ceramide glucosyltransferase
UP in U87	**49854**	ZBTB21	2.3799754	0.9393745	5.205279	zinc finger and BTB domain containing 21
UP in U87	**63905**	MANBAL	1.0059913	0.8849805	2.008323	mannosidase, beta A, lysosomal-like
UP in U87	**8507**	ENC1	6.0074124	0.9922218	64.32967	ectodermal-neural cortex 1 (with BTB
UP in U87	**2246**	FGF1	7.033423	0.8236884	131	fibroblast growth factor 1 (acidic)
UP in U87	**643837**	LOC64383	1.1929439	0.8523921	2.286188	uncharacterized LOC643837
UP in U87	**2157**	F8	3.7575716	0.9647448	13.52514	coagulation factor VIII, procoagulant
UP in U87	**9570**	GOSR2	1.1319915	0.886794	2.191611	golgi SNAP receptor complex member 2
UP in U87	**84270**	C9orf89	2.1953242	0.9403968	4.579926	chromosome 9 open reading frame 89
UP in U87	**26240**	FAM50B	2.3805653	0.8369069	5.207407	family with sequence similarity 50, member
UP in U87	**57477**	SHROOM	4.5377479	0.8543122	23.22727	shroom family member 4
UP in U87	**6574**	SLC20A1	2.0552773	0.9405124	4.156235	solute carrier family 20 (phosphate transporter), member 1
UP in U87	**8853**	ASAP2	1.5694009	0.8958878	2.967814	ArfGAP with SH3 domain, ankyrin repeat and PH domain 2
UP in U87	**4013**	VWA5A	4.6762559	0.9802923	25.5678	von Willebrand factor A domain containing
UP in U87	**84926**	SPRYD3	1.4357881	0.9006703	2.705299	SPRY domain containing 3
UP in U87	**10365**	KLF2	2.4012816	0.9365566	5.282723	Kruppel-like factor 2 (lung)
UP in U87	**349196**	LOC34919	2.5223119	0.9097819	5.74502	uncharacterized LOC349196
UP in U87	**1796**	DOK1	3.206467	0.9683361	9.230872	docking protein 1, 62kDa (downstream of tyrosine kinase 1)
UP in U87	**5921**	RASA1	1.9304733	0.9237559	3.811802	RAS p21 protein activator (GTPase activating protein) 1
UP in U87	**54676**	GTPBP2	1.6854245	0.9233381	3.21635	GTP binding protein 2
UP in U87	**6400**	SEL1L	1.3142672	0.8955944	2.48676	sel-1 suppressor of lin-12-like (C. elegans)
UP in U87	**348262**	FAM195B	2.2280385	0.9379167	4.684966	family with sequence similarity 195, member
UP in U87	**84984**	CEP19	2.1323771	0.897319	4.384393	centrosomal protein 19kDa
UP in U87	**387890**	TMEM233	10.847318	0.9945419	1842.333	transmembrane protein 233
UP in U87	**10536**	LEPREL2	3.3441522	0.9731719	10.15524	leprecan-like 2
UP in U87	**9245**	GCNT3	5.0797272	0.9448682	33.81818	glucosaminyl (N-acetyl) transferase 3, mucin
UP in U87	**8678**	BECN1	1.0487365	0.8860473	2.068717	beclin 1, autophagy related
UP in U87	**92565**	FANK1	3.5538016	0.8159191	11.74359	fibronectin type III and ankyrin repeat
UP in U87	**25769**	SLC24A2	7.5647846	0.883416	189.3333	solute carrier family 24 (sodium/potassium/ calcium exchanger), member 2
UP in U87	**22921**	MSRB2	1.3424326	0.873371	2.535785	methionine sulfoxide reductase B2
UP in U87	**2921**	CXCL3	7.116839	0.9969954	138.7976	chemokine (C-X-C motif) ligand 3
UP in U87	**59283**	CACNG8	4.8485384	0.9403257	28.81081	calcium channel, voltage-dependent, gamma subunit 8
UP in U87	**85013**	TMEM128	1.1448247	0.870802	2.211193	transmembrane protein 128
UP in U87	**4792**	NFKBIA	1.0005385	0.8846605	2.000747	nuclear factor of kappa light polypeptide gene enhancer in B-cells inhibitor, alpha
UP in U87	**3099**	HK2	1.5763993	0.920538	2.982246	hexokinase 2
UP in U87	**146198**	ZFP90	4.9248125	0.949864	30.375	ZFP90 zinc finger protein
UP in U87	**9945**	GFPT2	4.7247111	0.9927374	26.44112	glutamine-fructose-6-phosphate transaminase
UP in U87	**201134**	CEP112	2.7572781	0.8815581	6.761194	centrosomal protein 112kDa

UP in U87	**11022**	TDRKH	3.5339786	0.9612957	11.58333	tudor and KH domain containing
UP in U87	**51308**	REEP2	6.1085245	0.9944264	69	receptor accessory protein 2
UP in U87	**11201**	POLI	1.8669437	0.8626505	3.64759	polymerase (DNA directed) iota
UP in U87	**113277**	TMEM106	3.3146965	0.8868917	9.95	transmembrane protein 106A
UP in U87	**93185**	IGSF8	2.4621681	0.9490106	5.510442	immunoglobulin superfamily, member 8
UP in U87	**27342**	RABGEF1	1.3429494	0.89579	2.536694	RAB guanine nucleotide exchange factor
UP in U87	**1047**	CLGN	4.0124448	0.974932	16.13861	calmegin
UP in U87	**59344**	ALOXE3	3.6798504	0.8298309	12.81579	arachidonate lipoxygenase 3
UP in U87	**9641**	IKBKE	2.0153534	0.9214447	4.042796	inhibitor of kappa light polypeptide gene enhancer in B-cells, kinase epsilon
UP in U87	**8453**	CUL2	1.3445504	0.8963234	2.53951	cullin 2
UP in U87	**2678**	GGT1	2.0604081	0.9333209	4.171043	gamma-glutamyltransferase 1
UP in U87	**4054**	LTBP3	1.3957154	0.9031059	2.63119	latent transforming growth factor beta binding protein 3
UP in U87	**6700**	SPRR2A	8.8276073	0.9632158	454.3333	small proline-rich protein 2A
UP in U87	**5577**	PRKAR2B	2.0692853	0.8653528	4.196787	protein kinase, cAMP-dependent, regulatory, type II, beta
UP in U87	**211**	ALAS1	1.0831388	0.8888919	2.11864	aminolevulinate, delta-, synthase 1
UP in U87	**222389**	BEND7	6.961932	0.8151013	124.6667	BEN domain containing 7
UP in U87	**56005**	C19orf10	1.1826866	0.8964567	2.269991	chromosome 19 open reading frame 10
UP in U87	**4321**	MMP12	5.3972162	0.9281651	42.14286	matrix metallopeptidase 12 (macrophage
UP in U87	**84343**	HPS3	2.0029628	0.9361744	4.008223	Hermansky-Pudlak syndrome 3
UP in U87	**9100**	USP10	1.1058779	0.8850961	2.152298	ubiquitin specific peptidase 10
UP in U87	**160**	AP2A1	1.2676596	0.8986168	2.407707	adaptor-related protein complex 2, alpha 1 subunit
UP in U87	**1278**	COL1A2	10.841452	0.9981155	1834.857	collagen, type I, alpha 2
UP in U87	**3732**	CD82	3.7302067	0.9824791	13.27101	CD82 molecule
UP in U87	**23770**	FKBP8	1.4308317	0.9062617	2.696021	FK506 binding protein 8, 38kDa
UP in U87	**157769**	FAM91A1	1.3480168	0.8990435	2.545619	family with sequence similarity 91, member
UP in U87	**645367**	GGT8P	2.3648714	0.9370278	5.151067	gamma-glutamyltransferase 8 pseudogene
UP in U87	**64859**	NABP1	2.0308923	0.9353654	4.086575	nucleic acid binding protein 1
UP in U87	**7424**	VEGFC	7.3131422	0.9938486	159.0286	vascular endothelial growth factor C
UP in U87	**773**	CACNA1A	11.563355	0.9969776	3026.333	calcium channel, voltage-dependent, P/Q type, alpha 1A subunit
UP in U87	**7133**	TNFRSF1 B	5.4115774	0.9938397	42.56446	tumor necrosis factor receptor superfamily, member 1B
UP in U87	**55245**	UQCC	1.2739893	0.8959856	2.418293	ubiquinol-cytochrome c reductase complex chaperone
UP in U87	**7162**	TPBG	1.6971824	0.9210269	3.24267	trophoblast glycoprotein
UP in U87	**56985**	ADPRM	1.2299099	0.848543	2.345523	ADP-ribose/CDP-alcohol diphosphatase, manganese-dependent
UP in U87	**5271**	SERPINB8	4.0535644	0.9832702	16.60521	serpin peptidase inhibitor, clade B (ovalbumin), member 8
UP in U87	**51166**	AADAT	1.3069678	0.8360624	2.47421	aminoadipate aminotransferase
UP in U87	**3690**	ITGB3	10.886014	0.9998222	1892.417	integrin, beta 3 (platelet glycoprotein IIIa, antigen CD61)
UP in U87	**744**	MPPED2	4.0015275	0.9208047	16.01695	metallophosphoesterase domain containing 2
UP in U87	**91010**	FMNL3	3.7042746	0.972763	13.0346	formin-like 3
UP in U87	**287**	ANK2	5.5143079	0.9158444	45.70588	ankyrin 2, neuronal
UP in U87	**55840**	EAF2	3.1199452	0.827973	8.693548	ELL associated factor 2
UP in U87	**80325**	ABTB1	2.409858	0.9433659	5.31422	ankyrin repeat and BTB (POZ) domain containing 1
UP in U87	**56913**	C1GALT1	1.0473057	0.8181947	2.066667	core 1 synthase, glycoprotein-N-acetylgalactosamine 3-beta-galactosyltransferase, 1
UP in U87	**333**	APLP1	1.6230019	0.9130265	3.080153	amyloid beta (A4) precursor-like protein 1
UP in U87	**6525**	SMTN	4.8939185	0.9936708	29.73146	smoothelin

UP in U87	**254896**	LOC25489	6.9346738	0.8105499	122.3333	uncharacterized LOC254896
UP in U87	**51702**	PADI3	2.7284541	0.8465074	6.627451	peptidyl arginine deiminase, type III
UP in U87	**26050**	SLITRK5	7.0266771	0.9821679	130.3889	SLIT and NTRK-like family, member 5
UP in U87	**401884**	MGC5734	1.6536225	0.888074	3.146226	uncharacterized LOC401884
UP in U87	**5027**	P2RX7	3.3728895	0.9047327	10.35955	purinergic receptor P2X, ligand-gated ion channel, 7
UP in U87	**10493**	VAT1	2.1720224	0.9423703	4.506547	vesicle amine transport protein 1 homolog (T. californica)
UP in U87	**10043**	TOM1	2.8910203	0.9634914	7.417949	target of myb1 (chicken)
UP in U87	**26090**	ABHD12	1.2040916	0.8927409	2.303922	abhydrolase domain containing 12
UP in U87	**10463**	SLC30A9	1.5850874	0.9195602	3.00026	solute carrier family 30 (zinc transporter), member 9
UP in U87	**4135**	MAP6	8.0714624	0.9248049	269	microtubule-associated protein 6
UP in U87	**130132**	RFTN2	5.3037807	0.8911853	39.5	raftlin family member 2
UP in U87	**6006**	RHCE	5.0874628	0.9263961	34	Rh blood group, CcEe antigens
UP in U87	**79165**	LENG1	1.162297	0.8635483	2.238135	leukocyte receptor cluster (LRC) member 1
UP in U87	**26249**	KLHL3	5.5830828	0.9150177	47.9375	kelch-like family member 3
UP in U87	**144406**	WDR66	3.4429435	0.8333244	10.875	WD repeat domain 66
UP in U87	**9473**	THEMIS2	5.5570622	0.991244	47.08065	thymocyte selection associated family
UP in U87	**51334**	PRR16	4.3696349	0.9439348	20.67241	proline rich 16
UP in U87	**284615**	ANKRD34	2.6420756	0.9163511	6.242291	ankyrin repeat domain 34A
UP in U87	**23640**	HSPBP1	1.0553111	0.8856828	2.078166	HSPA (heat shock 70kDa) binding protein, cytoplasmic cochaperone 1
UP in U87	**340075**	ARSI	5.6532775	0.9839191	50.32759	arylsulfatase family, member I
UP in U87	**255104**	TMCO4	1.0885214	0.8694419	2.12656	transmembrane and coiled-coil domains 4
UP in U87	**10226**	PLIN3	3.0937598	0.969225	8.537181	perilipin 3
UP in U87	**51148**	CERCAM	1.0589806	0.8856739	2.083459	cerebral endothelial cell adhesion molecule
UP in U87	**91252**	SLC39A13	1.4281123	0.901897	2.690944	solute carrier family 39 (zinc transporter), member 13
UP in U87	**11030**	RBPMS	2.4059788	0.9490195	5.29995	RNA binding protein with multiple splicing
UP in U87	**57580**	PREX1	3.0281791	0.9684961	8.157794	phosphatidylinositol-3,4,5-trisphosphate-dependent Rac exchange factor 1
UP in U87	**9870**	KIAA0317	1.5604259	0.9020481	2.949409	KIAA0317
UP in U87	**4642**	MYO1D	3.9704835	0.9711718	15.67598	myosin ID
UP in U87	**4126**	MANBA	1.4414938	0.8724021	2.716019	mannosidase, beta A, lysosomal
UP in U87	**3800**	KIF5C	9.7370783	0.992364	853.4	kinesin family member 5C
UP in U87	**5090**	PBX3	1.1446974	0.8350312	2.210997	pre-B-cell leukemia homeobox 3
UP in U87	**54103**	GSAP	10.085251	0.9891905	1086.333	gamma-secretase activating protein
UP in U87	**29062**	WDR91	1.244654	0.8539211	2.369617	WD repeat domain 91
UP in U87	**100130238**	LOC100130238	8.6815898	0.9795367	410.6	uncharacterized LOC100130238
UP in U87	**85480**	TSLP	5.2506368	0.975652	38.07143	thymic stromal lymphopoietin
UP in U87	**25890**	ABI3BP	5.0271848	0.9097641	32.6087	ABI family, member 3 (NESH) binding
UP in U87	**8605**	PLA2G4C	5.4002226	0.8723843	42.23077	phospholipase A2, group IVC (cytosolic, calcium-independent)
UP in U87	**6794**	STK11	4.7807634	0.9894661	27.48864	serine/threonine kinase 11
UP in U87	**8816**	DCAF5	1.0045679	0.8538322	2.006342	DDB1 and CUL4 associated factor 5
UP in U87	**56975**	FAM20C	2.8286885	0.9635625	7.104281	family with sequence similarity 20, member
UP in U87	**23433**	RHOQ	1.1317878	0.8893985	2.191301	ras homolog family member Q
UP in U87	**54664**	TMEM106	1.6717238	0.9105463	3.18595	transmembrane protein 106B
UP in U87	**201799**	TMEM154	7.1497471	0.9110175	142	transmembrane protein 154
UP in U87	**80256**	FAM214B	3.8524428	0.9809768	14.44444	family with sequence similarity 214, member
UP in U87	**153241**	CEP120	1.1062376	0.8581258	2.152835	centrosomal protein 120kDa
UP in U87	**10311**	DSCR3	1.1356686	0.8866962	2.197204	Down syndrome critical region gene 3
UP in U87	**83442**	SH3BGRL3	4.5907512	0.992524	24.09649	SH3 domain binding glutamic acid-rich protein like 3
UP in U87	**79155**	TNIP2	1.2932288	0.89659	2.450759	TNFAIP3 interacting protein 2

UP in U87	**2026**	ENO2	2.3797714	0.9518552	5.204543	enolase 2 (gamma, neuronal)
UP in U87	**284266**	SIGLEC15	12.922275	0.9993511	7762.333	sialic acid binding Ig-like lectin 15
UP in U87	**54503**	ZDHHC13	1.7081259	0.9113108	3.267361	zinc finger, DHHC-type containing 13
UP in U87	**6103**	RPGR	1.9728882	0.902297	3.925532	retinitis pigmentosa GTPase regulator
UP in U87	**79802**	HHIPL2	1.8488009	0.8505254	3.602007	HHIP-like 2
UP in U87	**2300**	FOXL1	9.7279205	0.9973954	848	forkhead box L1
UP in U87	**26011**	TENM4	7.5156998	0.8784824	183	teneurin transmembrane protein 4
UP in U87	**95681**	CEP41	2.2700892	0.9206713	4.823529	centrosomal protein 41kDa
UP in U87	**3791**	KDR	7.7165337	0.8977012	210.3333	kinase insert domain receptor (a type III receptor tyrosine kinase)
UP in U87	**90865**	IL33	9.5533093	0.9821857	751.3333	interleukin 33
UP in U87	**9910**	RABGAP1	1.1701668	0.8247995	2.250377	RAB GTPase activating protein 1-like
UP in U87	**1312**	COMT	1.9844846	0.9281562	3.957213	catechol-O-methyltransferase
UP in U87	**5293**	PIK3CD	2.5818944	0.952193	5.987254	phosphatidylinositol-4,5-bisphosphate 3-kinase, catalytic subunit delta
UP in U87	**7462**	LAT2	2.0955393	0.8650595	4.273859	linker for activation of T cells family,
UP in U87	**3984**	LIMK1	2.7080198	0.959749	6.534242	LIM domain kinase 1
UP in U87	**148979**	GLIS1	4.665528	0.9288406	25.37838	GLIS family zinc finger 1
UP in U87	**7353**	UFD1L	1.4434831	0.9045638	2.719767	ubiquitin fusion degradation 1 like (yeast)
UP in U87	**374897**	SBSN	8.8417458	0.9977954	458.8077	suprabasin
UP in U87	**59271**	EVA1C	1.055403	0.8722421	2.078299	eva-1 homolog C (C. elegans)
UP in U87	**9448**	MAP4K4	1.1718031	0.8904297	2.252931	mitogen-activated protein kinase kinase kinase kinase 4
UP in U87	**23558**	WBP2	1.6198243	0.9232137	3.073376	WW domain binding protein 2
UP in U87	**25945**	PVRL3	1.4144942	0.8922431	2.665663	poliovirus receptor-related 3
UP in U87	**57717**	PCDHB16	6.2985536	0.9484417	78.71429	protocadherin beta 16
UP in U87	**3575**	IL7R	8.3837043	0.9622647	334	interleukin 7 receptor
UP in U87	**10561**	IFI44	3.8833038	0.9769321	14.75676	interferon-induced protein 44
UP in U87	**55777**	MBD5	1.4696008	0.8036073	2.769452	methyl-CpG binding domain protein 5
UP in U87	**80013**	FAM188A	1.201869	0.8842605	2.300375	family with sequence similarity 188, member
UP in U87	**3866**	KRT15	2.9803528	0.9422103	7.891791	keratin 15
UP in U87	**64080**	RBKS	2.2760993	0.9094529	4.843666	ribokinase
UP in U87	**55062**	WIPI1	4.4720663	0.9907195	22.19352	WD repeat domain, phosphoinositide interacting 1
UP in U87	**80128**	TRIM46	2.2886896	0.8941544	4.886121	tripartite motif containing 46
UP in U87	**58986**	TMEM8A	1.1239704	0.8827226	2.179459	transmembrane protein 8A
UP in U87	**6932**	TCF7	4.0086937	0.9827546	16.09671	transcription factor 7 (T-cell specific, HMG-
UP in U87	**6447**	SCG5	3.994007	0.973003	15.93367	secretogranin V (7B2 protein)
UP in U87	**84879**	MFSD2A	3.2931291	0.9610201	9.80236	major facilitator superfamily domain containing 2A
UP in U87	**81628**	TSC22D4	1.4408317	0.902697	2.714773	TSC22 domain family, member 4
UP in U87	**26470**	SEZ6L2	2.3634211	0.9482284	5.145892	seizure related 6 homolog (mouse)-like 2
UP in U87	**3371**	TNC	5.2982381	0.995502	39.34854	tenascin C
UP in U87	**729156**	GTF2IRD1 P1	1.3260812	0.8764023	2.507207	GTF2I repeat domain containing 1 pseusogene 1
UP in U87	**8495**	PPFIBP2	1.6287442	0.8419916	3.092437	PTPRF interacting protein, binding protein 2 (liprin beta 2)
UP in U87	**387921**	NHLRC3	1.0635357	0.8545434	2.090047	NHL repeat containing 3
UP in U87	**9435**	CHST2	6.7593334	0.9860348	108.3333	carbohydrate (N-acetylglucosamine-6-O) sulfotransferase 2
UP in U87	**1069**	CETN2	1.8672715	0.9276939	3.648419	centrin, EF-hand protein, 2
UP in U87	**611**	OPN1SW	2.8943898	0.8445784	7.435294	opsin 1 (cone pigments), short-wave-sensitive
UP in U87	**9706**	ULK2	3.3692338	0.8774335	10.33333	unc-51-like kinase 2 (C. elegans)
UP in U87	**57089**	ENTPD7	1.1556976	0.8565079	2.22792	ectonucleoside triphosphate diphosphohydrolase 7

UP in U87	**5054**	SERPINE1	9.3491967	0.9999556	652.2118	serpin peptidase inhibitor, clade E (nexin, plasminogen activator inhibitor type 1), member 1
UP in U87	**283927**	NUDT7	2.7575465	0.928814	6.762452	nudix (nucleoside diphosphate linked moiety X)-type motif 7
UP in U87	**92949**	ADAMTS L1	8.3101583	0.9703006	317.4	ADAMTS-like 1
UP in U87	**3214**	HOXB4	3.2902797	0.9114975	9.783019	homeobox B4
UP in U87	**85463**	ZC3H12C	3.1504371	0.9498551	8.879245	zinc finger CCCH-type containing 12C
UP in U87	**100861402**	CERS6-	2.2810944	0.9329742	4.860465	CERS6 antisense RNA 1
UP in U87	**222223**	KIAA1324	1.8739271	0.830471	3.665289	KIAA1324-like
UP in U87	**55909**	BIN3	2.029291	0.9304674	4.082042	bridging integrator 3
UP in U87	**6095**	RORA	6.8858997	0.9737142	118.2667	RAR-related orphan receptor A
UP in U87	**25927**	CNRIP1	3.5977411	0.9762565	12.10676	cannabinoid receptor interacting protein 1
UP in U87	**4289**	MKLN1	1.0994082	0.8779668	2.142668	muskelin 1, intracellular mediator containing kelch motifs
UP in U87	**902**	CCNH	1.2963408	0.8968834	2.456052	cyclin H
UP in U87	**2983**	GUCY1B3	8.4824719	0.9489217	357.6667	guanylate cyclase 1, soluble, beta 3
UP in U87	**9953**	HS3ST3B1	3.2357596	0.943757	9.420213	heparan sulfate (glucosamine) 3-O-sulfotransferase 3B1
UP in U87	**57511**	COG6	1.7519106	0.9125198	3.368043	component of oligomeric golgi complex 6
UP in U87	**257019**	FRMD3	2.5210903	0.929054	5.740157	FERM domain containing 3
UP in U87	**26231**	LRRC29	4.4103513	0.9402368	21.26415	leucine rich repeat containing 29
UP in U87	**822**	CAPG	1.4465209	0.9063861	2.7255	capping protein (actin filament), gelsolin-like
UP in U87	**84970**	C1orf94	11.460797	0.9966576	2818.667	chromosome 1 open reading frame 94
UP in U87	**164781**	DAW1	9.1724275	0.9740697	577	dynein assembly factor with WDR repeat domains 1
UP in U87	**9194**	SLC16A7	1.5653651	0.837618	2.959524	solute carrier family 16, member 7 (monocarboxylic acid transporter 2)
UP in U87	**131034**	CPNE4	6.3487282	0.8583036	81.5	copine IV
UP in U87	**2252**	FGF7	5.4020984	0.8835405	42.28571	fibroblast growth factor 7
UP in U87	**8720**	MBTPS1	1.0117944	0.8779046	2.016417	membrane-bound transcription factor peptidase, site 1
UP in U87	**3122**	HLA-DRA	11.774924	0.997511	3504.333	major histocompatibility complex, class II, DR alpha
UP in U87	**114805**	GALNT13	3.7715838	0.8293776	13.65714	UDP-N-acetyl-alpha-D-galactosamine:polypeptide N-acetylgalactosaminyltransferase 13 (GalNAc-T13)
UP in U87	**63925**	ZNF335	1.0405581	0.8783491	2.057023	zinc finger protein 335
UP in U87	**118812**	MORN4	2.8890008	0.9526553	7.407572	MORN repeat containing 4
UP in U87	**55577**	NAGK	2.3837334	0.9484061	5.218855	N-acetylglucosamine kinase
UP in U87	**246721**	POLR2J2	1.8756293	0.9233115	3.669617	polymerase (RNA) II (DNA directed) polypeptide J2
UP in U87	**5526**	PPP2R5B	3.5924962	0.9763098	12.06283	protein phosphatase 2, regulatory subunit B', beta
UP in U87	**81619**	TSPAN14	1.1114266	0.8818337	2.160592	tetraspanin 14
UP in U87	**3306**	HSPA2	2.1785639	0.9039504	4.527027	heat shock 70kDa protein 2
UP in U87	**28962**	OSTM1	2.7939304	0.9581222	6.935166	osteopetrosis associated transmembrane protein 1
UP in U87	**4312**	MMP1	11.371958	0.9964176	2650.333	matrix metallopeptidase 1 (interstitial
UP in U87	**81846**	SBF2	1.1481456	0.8712731	2.216288	SET binding factor 2
UP in U87	**11345**	GABARA PL2	1.1029059	0.8907497	2.147869	GABA(A) receptor-associated protein-like 2
UP in U87	**3936**	LCP1	8.7522134	0.9808256	431.2	lymphocyte cytosolic protein 1 (L-plastin)
UP in U87	**219749**	ZNF25	4.2886435	0.9699717	19.54386	zinc finger protein 25
UP in U87	**58488**	PCTP	1.580042	0.9016214	2.989785	phosphatidylcholine transfer protein

UP in U87	**5873**	RAB27A	1.4267111	0.8960922	2.688332	RAB27A, member RAS oncogene family
UP in U87	**9617**	MTRF1	1.3625701	0.8623215	2.571429	mitochondrial translational release factor 1
UP in U87	**730101**	LOC73010	1.8599271	0.8455118	3.629893	uncharacterized LOC730101
UP in U87	**3239**	HOXD13	7.1272206	0.9638114	139.8	homeobox D13
UP in U87	**26234**	FBXL5	1.5298611	0.8998258	2.88758	F-box and leucine-rich repeat protein 5
UP in U87	**2710**	GK	1.6931113	0.8777179	3.233533	glycerol kinase
UP in U87	**23362**	PSD3	1.7912189	0.9112042	3.461072	pleckstrin and Sec7 domain containing 3
UP in U87	**84707**	BEX2	10.422766	0.9919729	1372.667	brain expressed X-linked 2
UP in U87	**2937**	GSS	1.374922	0.9024037	2.593539	glutathione synthetase
UP in U87	**400581**	GRAPL	6.9924663	0.8180259	127.3333	GRB2-related adaptor protein-like
UP in U87	**79132**	DHX58	3.2147129	0.8680819	9.283784	DEXH (Asp-Glu-X-His) box polypeptide 58
UP in U87	**57060**	PCBP4	1.4357261	0.9040215	2.705183	poly(rC) binding protein 4
UP in U87	**200916**	RPL22L1	2.46117	0.9517574	5.506631	ribosomal protein L22-like 1
UP in U87	**55906**	ZC4H2	4.2746224	0.9629491	19.35484	zinc finger, C4H2 domain containing
UP in U87	**56967**	C14orf132	3.5952819	0.9686117	12.08614	chromosome 14 open reading frame 132
UP in U87	**26525**	IL36RN	10.768184	0.994213	1744	interleukin 36 receptor antagonist
UP in U87	**390928**	PAPL	11.616396	0.997111	3139.667	iron/zinc purple acid phosphatase-like protein
UP in U87	**6274**	S100A3	1.3359453	0.8836382	2.524408	S100 calcium binding protein A3
UP in U87	**606**	NBEAP1	7.0826329	0.986826	135.5455	neurobeachin pseudogene 1
UP in U87	**389813**	C9orf172	1.6602179	0.8014916	3.160643	chromosome 9 open reading frame 172
UP in U87	**221692**	PHACTR1	6.1361367	0.8336534	70.33333	phosphatase and actin regulator 1
UP in U87	**25766**	PRPF40B	2.105082	0.9095685	4.302222	PRP40 pre-mRNA processing factor 40 homolog B (S. cerevisiae)
UP in U87	**100532731**	COMMD3-BMI1	2.9012633	0.9558643	7.470803	COMMD3-BMI1 readthrough
UP in U87	**196500**	PIANP	5.6244909	0.9420591	49.33333	PILR alpha associated neural protein
UP in U87	**56925**	LXN	7.2706847	0.9758387	154.4167	latexin
UP in U87	**729440**	CCDC61	2.1754043	0.8888296	4.517123	coiled-coil domain containing 61
UP in U87	**729013**	LOC72901	2.952203	0.9397746	7.7393	uncharacterized LOC729013
UP in U87	**7041**	TGFB1I1	2.1070126	0.93749	4.307983	transforming growth factor beta 1 induced transcript 1
UP in U87	**57190**	SEPN1	3.5889952	0.9796434	12.03359	selenoprotein N, 1
UP in U87	**659**	BMPR2	1.0206482	0.8473874	2.02883	bone morphogenetic protein receptor, type II (serine/threonine kinase)
UP in U87	**54495**	TMX3	1.1396051	0.8877007	2.203207	thioredoxin-related transmembrane protein 3
UP in U87	**284293**	HMSD	9.20049	0.9747364	588.3333	histocompatibility (minor) serpin domain containing
UP in U87	**3428**	IFI16	2.1591363	0.9381034	4.466474	interferon, gamma-inducible protein 16
UP in U87	**11046**	SLC35D2	2.2828418	0.9372944	4.866356	solute carrier family 35, member D2
UP in U87	**23002**	DAAM1	1.6092611	0.825164	3.050955	dishevelled associated activator of morphogenesis 1
UP in U87	**9016**	SLC25A14	1.1650592	0.8665707	2.242424	solute carrier family 25 (mitochondrial carrier, brain), member 14
UP in U87	**23080**	AVL9	1.0387866	0.8799669	2.054499	AVL9 homolog (S. cerevisiase)
UP in U87	**79143**	MBOAT7	1.6269897	0.9228492	3.088678	membrane bound O-acyltransferase domain containing 7
UP in U87	**283481**	FGF14-	5.4084974	0.9192579	42.47368	FGF14 antisense RNA 2
UP in U87	**81848**	SPRY4	6.6071303	0.9889239	97.48649	sprouty homolog 4 (Drosophila)
UP in U87	**6038**	RNASE4	2.1919255	0.8486319	4.569149	ribonuclease, RNase A family, 4
UP in U87	**23216**	TBC1D1	2.9946862	0.9589489	7.970588	TBC1 (tre-2/USP6, BUB2, cdc16) domain family, member 1
UP in U87	**4291**	MLF1	1.0771436	0.8555834	2.109855	myeloid leukemia factor 1
UP in U87	**8082**	SSPN	7.0552824	0.8729799	133	sarcospan
UP in U87	**90956**	ADCK2	1.1598008	0.8824115	2.234266	aarF domain containing kinase 2
UP in U87	**23194**	FBXL7	8.9715436	0.9846392	502	F-box and leucine-rich repeat protein 7
UP in U87	**26578**	OSTF1	1.9533433	0.9266272	3.872709	osteoclast stimulating factor 1
UP in U87	**94120**	SYTL3	5.7714221	0.9912795	54.62245	synaptotagmin-like 3

UP in U87	**100129534**	LOC100129534	1.4640262	0.8325955	2.758772	small nuclear ribonucleoprotein polypeptide N pseudogene
UP in U87	**22936**	ELL2	2.5514052	0.9502729	5.86205	elongation factor, RNA polymerase II, 2
UP in U87	**8476**	CDC42BP	1.6288659	0.9173023	3.092698	CDC42 binding protein kinase alpha
UP in U87	**50484**	RRM2B	2.2474209	0.9385034	4.748332	ribonucleotide reductase M2 B (TP53
UP in U87	**25798**	BRI3	1.7434194	0.926405	3.348278	brain protein I3
UP in U87	**4668**	NAGA	1.46501	0.8958433	2.760654	N-acetylgalactosaminidase, alpha-
UP in U87	**467**	ATF3	3.9974597	0.9840169	15.97185	activating transcription factor 3
UP in U87	**26108**	PYGO1	1.6322682	0.8509521	3.1	pygopus homolog 1 (Drosophila)
UP in U87	**55871**	CBWD1	1.2646051	0.8947322	2.402614	COBW domain containing 1
UP in U87	**23492**	CBX7	2.1389494	0.9186446	4.404412	chromobox homolog 7
UP in U87	**10215**	OLIG2	6.8621207	0.8008249	116.3333	oligodendrocyte lineage transcription factor 2
UP in U87	**10981**	RAB32	1.5807526	0.9217114	2.991258	RAB32, member RAS oncogene family
UP in U87	**100132891**	LOC100132891	2.8807925	0.9342365	7.365546	uncharacterized LOC100132891
UP in U87	**145483**	FAM161B	2.4773895	0.899488	5.568889	family with sequence similarity 161, member
UP in U87	**6374**	CXCL5	4.8399596	0.9029726	28.64	chemokine (C-X-C motif) ligand 5
UP in U87	**5270**	SERPINE2	1.1224639	0.8895141	2.177185	serpin peptidase inhibitor, clade E (nexin, plasminogen activator inhibitor type 1), member 2
UP in U87	**22808**	MRAS	3.6656743	0.9710029	12.69048	muscle RAS oncogene homolog
UP in U87	**4157**	MC1R	1.8598931	0.8793536	3.629808	melanocortin 1 receptor (alpha melanocyte stimulating hormone receptor)
UP in U87	**4791**	NFKB2	2.0480291	0.9362544	4.135406	nuclear factor of kappa light polypeptide gene enhancer in B-cells 2 (p49/p100)
UP in U87	**55741**	EDEM2	2.1743551	0.9406191	4.513839	ER degradation enhancer, mannosidase alpha-like 2
UP in U87	**9021**	SOCS3	1.7659563	0.9243249	3.400994	suppressor of cytokine signaling 3
UP in U87	**55080**	TAPBPL	3.5990377	0.920698	12.11765	TAP binding protein-like
UP in U87	**7088**	TLE1	2.9933289	0.9571622	7.963093	transducin-like enhancer of split 1 (E(sp1) homolog, Drosophila)
UP in U87	**79008**	SLX1B	1.2973221	0.896999	2.457723	SLX1 structure-specific endonuclease subunit homolog B (S. cerevisiae)
UP in U87	**123803**	NTAN1	1.1177078	0.8894874	2.170019	N-terminal asparagine amidase
UP in U87	**83706**	FERMT3	4.2819729	0.9684961	19.4537	fermitin family member 3
UP in U87	**56938**	ARNTL2	1.6405189	0.9110797	3.117779	aryl hydrocarbon receptor nuclear translocator-like 2
UP in U87	**54762**	GRAMD1	3.3378696	0.8392181	10.11111	GRAM domain containing 1C
UP in U87	**503538**	A1BG-AS1	5.681824	0.9160666	51.33333	A1BG antisense RNA 1
UP in U87	**56911**	MAP3K7C	7.3951771	0.8651217	168.3333	MAP3K7 C-terminal like
UP in U87	**9542**	NRG2	7.3923174	0.9280406	168	neuregulin 2
UP in U87	**79094**	CHAC1	1.9964032	0.9089818	3.99004	ChaC, cation transport regulator homolog 1 (E. coli)
UP in U87	**6236**	RRAD	11.09148	0.9999556	2182.068	Ras-related associated with diabetes
UP in U87	**7791**	ZYX	1.03627	0.8880918	2.050918	zyxin
UP in U87	**10955**	SERINC3	1.296472	0.9001191	2.456275	serine incorporator 3
UP in U87	**92799**	SHKBP1	2.1006158	0.940379	4.288924	SH3KBP1 binding protein 1
UP in U87	**27175**	TUBG2	1.1972871	0.8850605	2.293081	tubulin, gamma 2
UP in U87	**716**	C1S	4.8156695	0.9915462	28.16184	complement component 1, s subcomponent
UP in U87	**10308**	ZNF267	1.6718981	0.9052305	3.186335	zinc finger protein 267
UP in U87	**595**	CCND1	2.5179735	0.9530019	5.72777	cyclin D1
UP in U87	**79571**	GCC1	1.8825907	0.9199602	3.687366	GRIP and coiled-coil domain containing 1
UP in U87	**4015**	LOX	3.7732688	0.9796345	13.6731	lysyl oxidase
UP in U87	**8881**	CDC16	1.2555251	0.8957989	2.38754	cell division cycle 16
UP in U87	**10221**	TRIB1	2.4114262	0.9445126	5.32	tribbles homolog 1 (Drosophila)
UP in U87	**51347**	TAOK3	1.7951605	0.9154622	3.470541	TAO kinase 3
UP in U87	**64762**	GAREM	1.9844031	0.8104166	3.956989	GRB2 associated, regulator of MAPK1

UP in U87	**6641**	SNTB1	3.0236819	0.9490906	8.132404	syntrophin, beta 1 (dystrophin-associated protein A1, 59kDa, basic component 1)
UP in U87	**83546**	RTBDN	4.2479275	0.9007236	19	retbindin
UP in U87	**79269**	DCAF10	1.2010475	0.866864	2.299065	DDB1 and CUL4 associated factor 10
UP in U87	**57678**	GPAM	1.8873012	0.9222536	3.699425	glycerol-3-phosphate acyltransferase, mitochondrial
UP in U87	**333926**	PPM1J	4.975422	0.9474194	31.45946	protein phosphatase, Mg2+/Mn2+ dependent,
UP in U87	**329**	BIRC2	1.028729	0.8816115	2.040226	baculoviral IAP repeat containing 2
UP in U87	**23452**	ANGPTL2	5.4551057	0.9921418	43.86826	angiopoietin-like 2
UP in U87	**2115**	ETV1	10.884679	0.9946753	1890.667	ets variant 1
UP in U87	**55860**	ACTR10	1.2827122	0.8953011	2.432959	actin-related protein 10 homolog (S.
UP in U87	**146691**	TOM1L2	1.165153	0.883656	2.24257	target of myb1-like 2 (chicken)
UP in U87	**5587**	PRKD1	2.9497992	0.8788291	7.726415	protein kinase D1
UP in U87	**91442**	C19orf40	1.3392061	0.8760289	2.53012	chromosome 19 open reading frame 40
UP in U87	**55783**	FTSJD1	1.1277356	0.8607837	2.185155	FtsJ methyltransferase domain containing 1
UP in U87	**131408**	FAM131A	1.3211974	0.8938877	2.498734	family with sequence similarity 131, member
UP in U87	**29969**	MDFIC	1.0211216	0.8730243	2.029496	MyoD family inhibitor domain containing
UP in U87	**3423**	IDS	3.028211	0.9685228	8.157974	iduronate 2-sulfatase
UP in U87	**554236**	DPY19L2P	6.662965	0.8913809	101.3333	dpy-19-like 2 pseudogene 1 (C. elegans)
UP in U87	**374654**	KIF7	1.6734322	0.9171422	3.189725	kinesin family member 7
UP in U87	**400410**	ST20	1.2123036	0.8836471	2.317073	suppressor of tumorigenicity 20
UP in U87	**3553**	IL1B	18.372849	1	339452.3	interleukin 1, beta
UP in U87	**83937**	RASSF4	2.5493237	0.9319164	5.853598	Ras association (RalGDS/AF-6) domain family member 4
UP in U87	**55619**	DOCK10	2.4694526	0.9396501	5.538336	dedicator of cytokinesis 10
UP in U87	**2888**	GRB14	4.5642889	0.9688783	23.65854	growth factor receptor-bound protein 14
UP in U87	**166336**	PRICKLE2	8.4350914	0.988266	346.1111	prickle homolog 2 (Drosophila)
UP in U87	**9564**	BCAR1	1.1095949	0.8885807	2.15785	breast cancer anti-estrogen resistance 1
UP in U87	**11259**	FILIP1L	8.6522475	0.9569933	402.3333	filamin A interacting protein 1-like
UP in U87	**9**	NAT1	1.7738922	0.849832	3.419753	N-acetyltransferase 1 (arylamine N-acetyltransferase)
UP in U87	**81558**	FAM117A	3.1465487	0.9286895	8.855346	family with sequence similarity 117, member
UP in U87	**51019**	CCDC53	1.7601552	0.9230981	3.387346	coiled-coil domain containing 53
UP in U87	**113452**	TMEM54	3.5346072	0.9767365	11.58838	transmembrane protein 54
UP in U87	**96610**	LOC96610	1.0038455	0.8410226	2.005338	BMS1 homolog, ribosome assembly protein (yeast) pseudogene
UP in U87	**4665**	NAB2	1.3018745	0.8925276	2.46549	NGFI-A binding protein 2 (EGR1 binding protein 2)
UP in U87	**6282**	S100A11	1.6280328	0.9237737	3.090912	S100 calcium binding protein A11
UP in U87	**11153**	FICD	1.840898	0.8891141	3.582329	FIC domain containing
UP in U87	**3716**	JAK1	1.3010293	0.8991235	2.464046	Janus kinase 1
UP in U87	**2962**	GTF2F1	1.2364808	0.8970434	2.356231	general transcription factor IIF, polypeptide 1, 74kDa
UP in U87	**6640**	SNTA1	1.1755716	0.8864651	2.258824	syntrophin, alpha 1
UP in U87	**348751**	FONG	4.296617	0.8301332	19.65217	uncharacterized LOC348751
UP in U87	**56912**	IFT46	2.9672501	0.9499884	7.820442	intraflagellar transport 46 homolog (Chlamydomonas)
UP in U87	**9069**	CLDN12	1.3706971	0.8984479	2.585955	claudin 12
UP in U87	**200035**	NUDT17	2.6803821	0.8593614	6.410256	nudix (nucleoside diphosphate linked moiety X)-type motif 17
UP in U87	**79953**	SYNDIG1	9.6244909	0.9833236	789.3333	synapse differentiation inducing 1
UP in U87	**7844**	RNF103	1.2712487	0.882047	2.413704	ring finger protein 103
UP in U87	**1393**	CRHBP	7.3339007	0.8585347	161.3333	corticotropin releasing hormone binding
UP in U87	**9825**	SPATA2	1.4442353	0.8771223	2.721186	spermatogenesis associated 2
UP in U87	**220213**	OTUD1	1.5545889	0.8316621	2.9375	OTU domain containing 1
UP in U87	**57687**	VAT1L	11.342445	0.9963554	2596.667	vesicle amine transport protein 1 homolog (T. californica)-like

UP in U87	**7223**	TRPC4	3.1520031	0.8092876	8.888889	transient receptor potential cation channel, subfamily C, member 4
UP in U87	**55364**	IMPACT	1.0784781	0.87434	2.111807	impact RWD domain protein
UP in U87	**2149**	F2R	1.1414556	0.879958	2.206035	coagulation factor II (thrombin) receptor
UP in U87	**25999**	CLIP3	6.6274123	0.9945242	98.86667	CAP-GLY domain containing linker protein
UP in U87	**51528**	JKAMP	1.8789126	0.9253294	3.677977	JNK1/MAPK8-associated membrane protein
UP in U87	**29015**	SLC43A3	13.121534	0.9995111	8912	solute carrier family 43, member 3
UP in U87	**27180**	SIGLEC9	6.9503129	0.8137678	123.6667	sialic acid binding Ig-like lectin 9
UP in U87	**83940**	TATDN1	1.6755825	0.9215158	3.194483	TatD DNase domain containing 1
UP in U87	**55122**	AKIRIN2	1.030954	0.8821448	2.043375	akirin 2
UP in U87	**57724**	EPG5	1.9592013	0.9125198	3.888466	ectopic P-granules autophagy protein 5 homolog (C. elegans)
UP in U87	**11193**	WBP4	1.7305424	0.9101108	3.318526	WW domain binding protein 4
UP in U87	**25976**	TIPARP	2.9046744	0.9628869	7.488488	TCDD-inducible poly(ADP-ribose)
UP in U87	**5372**	PMM1	1.2902203	0.897879	2.445654	phosphomannomutase 1
UP in U87	**861**	RUNX1	1.6099117	0.9150977	3.052332	runt-related transcription factor 1
UP in U87	**571**	BACH1	1.0937658	0.8571835	2.134304	BTB and CNC homology 1, basic leucine zipper transcription factor 1
UP in U87	**80328**	ULBP2	2.4913187	0.9349032	5.622917	UL16 binding protein 2
UP in U87	**1608**	DGKG	5.8984784	0.982079	59.65116	diacylglycerol kinase, gamma 90kDa
UP in U87	**30001**	ERO1L	1.0759582	0.8840294	2.108122	ERO1-like (S. cerevisiae)
UP in U87	**7045**	TGFBI	1.3363706	0.9036393	2.525153	transforming growth factor, beta-induced,
UP in U87	**27347**	STK39	1.5974244	0.9150532	3.026026	serine threonine kinase 39
UP in U87	**113675**	SDSL	4.0203338	0.9787633	16.22711	serine dehydratase-like
UP in U87	**9522**	SCAMP1	1.2270689	0.8839316	2.340909	secretory carrier membrane protein 1
UP in U87	**8200**	GDF5	8.2807708	0.9380678	311	growth differentiation factor 5
UP in U87	**10513**	APPBP2	1.5889744	0.9069995	3.008354	amyloid beta precursor protein (cytoplasmic tail) binding protein 2
UP in U87	**767558**	LUZP6	1.6833334	0.9247693	3.211692	leucine zipper protein 6
UP in U87	**64168**	NECAB1	3.0822148	0.8633083	8.469136	N-terminal EF-hand calcium binding protein
UP in U87	**6303**	SAT1	1.904248	0.928414	3.743137	spermidine/spermine N1-acetyltransferase 1
UP in U87	**80003**	PCNXL2	2.2072125	0.9188846	4.617822	pecanex-like 2 (Drosophila)
UP in U87	**162681**	C18orf54	1.2185836	0.823155	2.327181	chromosome 18 open reading frame 54
UP in U87	**9609**	RAB36	1.1578986	0.8270574	2.231322	RAB36, member RAS oncogene family
UP in U87	**11020**	IFT27	2.7411025	0.9499529	6.685811	intraflagellar transport 27 homolog (Chlamydomonas)
UP in U87	**23034**	SAMD4A	3.157101	0.9557043	8.920354	sterile alpha motif domain containing 4A
UP in U87	**201895**	SMIM14	2.2122385	0.9338987	4.633937	small integral membrane protein 14
UP in U87	**220064**	ORAOV1	1.468498	0.8942077	2.767336	oral cancer overexpressed 1
UP in U87	**54518**	APBB1IP	7.9176704	0.9834569	241.8	amyloid beta (A4) precursor protein-binding, family B, member 1 interacting protein
UP in U87	**7316**	UBC	1.4934405	0.9083329	2.815596	ubiquitin C
UP in U87	**3213**	HOXB3	3.3340347	0.9474461	10.08427	homeobox B3
UP in U87	**1436**	CSF1R	4.9690781	0.9247871	31.32143	colony stimulating factor 1 receptor
UP in U87	**641649**	TMEM91	2.4010149	0.9021459	5.281746	transmembrane protein 91
UP in U87	**5468**	PPARG	1.458779	0.8191637	2.748756	peroxisome proliferator-activated receptor
UP in U87	**114987**	WDR31	3.1142961	0.8837982	8.659574	WD repeat domain 31
UP in U87	**1803**	DPP4	7.2429472	0.9879727	151.4762	dipeptidyl-peptidase 4
UP in U87	**3300**	DNAJB2	1.7874894	0.9240937	3.452136	DnaJ (Hsp40) homolog, subfamily B,
UP in U87	**5069**	PAPPA	1.3934042	0.8978168	2.626978	pregnancy-associated plasma protein A, pappalysin 1
UP in U87	**23552**	CDK20	1.3152797	0.8256707	2.488506	cyclin-dependent kinase 20
UP in U87	**387104**	SOGA3	8.6806525	0.9841592	410.3333	SOGA family member 3
UP in U87	**6548**	SLC9A1	1.601308	0.9126442	3.034183	solute carrier family 9, subfamily A (NHE1, cation proton antiporter 1), member 1
UP in U87	**132299**	OCIAD2	3.3298718	0.9756343	10.05521	OCIA domain containing 2
UP in U87	**10970**	CKAP4	1.3942046	0.9032837	2.628436	cytoskeleton-associated protein 4

UP in U87	**9397**	NMT2	1.7287352	0.8954255	3.314371	N-myristoyltransferase 2
UP in U87	**28966**	SNX24	1.4374223	0.8887763	2.708365	sorting nexin 24
UP in U87	**57683**	ZDBF2	1.1351596	0.8278308	2.196429	zinc finger, DBF-type containing 2
UP in U87	**378805**	FLJ43663	2.0567071	0.9068039	4.160356	uncharacterized LOC378805
UP in U87	**286527**	TMSB15B	8.8641861	0.9647715	466	thymosin beta 15B
UP in U87	**3998**	LMAN1	1.27442	0.8989546	2.419015	lectin, mannose-binding, 1
UP in U87	**57110**	HRASLS	6.2801079	0.9693761	77.71429	HRAS-like suppressor
UP in U87	**90987**	ZNF251	1.1104538	0.8487919	2.159136	zinc finger protein 251
UP in U87	**51665**	ASB1	2.0392341	0.93701	4.110273	ankyrin repeat and SOCS box containing 1
UP in U87	**80315**	CPEB4	2.4956199	0.9443526	5.639706	cytoplasmic polyadenylation element binding protein 4
UP in U87	**9518**	GDF15	4.8514395	0.9934397	28.8688	growth differentiation factor 15
UP in U87	**100533955**	SENP3-EIF4A1	2.457045	0.8170925	5.490909	SENP3-EIF4A1 readthrough
UP in U87	**3155**	HMGCL	1.6216568	0.9173823	3.077282	3-hydroxymethyl-3-methylglutaryl-CoA lyase
UP in U87	**27132**	CPNE7	2.4426233	0.9497129	5.436293	copine VII
UP in U87	**64779**	MTHFSD	1.9207474	0.8885807	3.786192	methenyltetrahydrofolate synthetase domain containing
UP in U87	**50804**	MYEF2	2.4731965	0.9121375	5.552727	myelin expression factor 2
UP in U87	**9034**	CCRL2	7.9188632	0.9142176	242	chemokine (C-C motif) receptor-like 2
UP in U87	**54470**	ARMCX6	7.0074945	0.9929685	128.6667	armadillo repeat containing, X-linked 6
UP in U87	**10472**	ZBTB18	1.1697208	0.839467	2.249682	zinc finger and BTB domain containing 18
UP in U87	**3976**	LIF	8.2219792	0.9996711	298.5811	leukemia inhibitory factor
UP in U87	**114294**	LACTB	1.2266336	0.8917276	2.340203	lactamase, beta
UP in U87	**2738**	GLI4	1.0202111	0.8469696	2.028216	GLI family zinc finger 4
UP in U87	**93129**	ORAI3	2.1734336	0.9307696	4.510957	ORAI calcium release-activated calcium modulator 3
UP in U87	**55290**	BRF2	1.3894521	0.8867851	2.619792	BRF2, subunit of RNA polymerase III transcription initiation factor, BRF1-like
UP in U87	**54839**	LRRC49	1.9992405	0.9066084	3.997895	leucine rich repeat containing 49
UP in U87	**55008**	HERC6	6.4405726	0.8905453	86.85714	HECT and RLD domain containing E3 ubiquitin protein ligase family member 6
UP in U87	**431707**	LHX8	7.9696264	0.9180312	250.6667	LIM homeobox 8
UP in U87	**58506**	SCAF1	1.1480986	0.890163	2.216216	SR-related CTD-associated factor 1
UP in U87	**8460**	TPST1	1.1235127	0.8767401	2.178768	tyrosylprotein sulfotransferase 1
UP in U87	**51351**	ZNF117	4.3063872	0.9374544	19.78571	zinc finger protein 117
UP in U87	**55748**	CNDP2	1.0579503	0.8824915	2.081971	CNDP dipeptidase 2 (metallopeptidase M20 family)
UP in U87	**10290**	SPEG	1.8517674	0.9227514	3.609421	SPEG complex locus
UP in U87	**220323**	OAF	1.5266145	0.9000302	2.88109	OAF homolog (Drosophila)
UP in U87	**57205**	ATP10D	4.8479969	0.9530286	28.8	ATPase, class V, type 10D
UP in U87	**112398**	EGLN2	1.4457252	0.9041282	2.723997	egl nine homolog 2 (C. elegans)
UP in U87	**9215**	LARGE	8.4664407	0.9842303	353.7143	like-glycosyltransferase
UP in U87	**10763**	NES	1.1312445	0.8800025	2.190476	nestin
UP in U87	**623**	BDKRB1	8.9666327	0.9965687	500.2941	bradykinin receptor B1
UP in U87	**3885**	KRT34	8.2013071	0.933392	294.3333	keratin 34
UP in U87	**997**	CDC34	1.311651	0.8989813	2.482254	cell division cycle 34
UP in U87	**6840**	SVIL	3.1372787	0.9686205	8.798629	supervillin
UP in U87	**1649**	DDIT3	2.4245815	0.9508507	5.368732	DNA-damage-inducible transcript 3
UP in U87	**81631**	MAP1LC3B	2.6955488	0.9592512	6.478002	microtubule-associated protein 1 light chain 3 beta
UP in U87	**6935**	ZEB1	1.6650986	0.9091507	3.171353	zinc finger E-box binding homeobox 1
UP in U87	**51176**	LEF1	8.4594316	0.9478106	352	lymphoid enhancer-binding factor 1
UP in U87	**64840**	PORCN	3.0841793	0.9665404	8.480676	porcupine homolog (Drosophila)
UP in U87	**29091**	STXBP6	2.9920219	0.8229239	7.955882	syntaxin binding protein 6 (amisyn)
UP in U87	**339745**	SPOPL	1.0461746	0.8530322	2.065047	speckle-type POZ protein-like
UP in U87	**25941**	TPGS2	1.0298799	0.8852294	2.041854	tubulin polyglutamylase complex subunit 2

UP in U87	**3981**	LIG4	1.3308438	0.8804025	2.515498	ligase IV, DNA, ATP-dependent
UP in U87	**23780**	APOL2	1.6479379	0.9156933	3.133854	apolipoprotein L, 2
UP in U87	**58504**	ARHGAP2	7.0641499	0.9973776	133.82	Rho GTPase activating protein 22
UP in U87	**7148**	TNXB	3.6254903	0.9432325	12.34188	tenascin XB
UP in U87	**64838**	FNDC4	4.0746419	0.9780966	16.84959	fibronectin type III domain containing 4
UP in U87	**54462**	CCSER2	1.6195875	0.9109464	3.072872	coiled-coil serine-rich protein 2
UP in U87	**79187**	FSD1	1.5190856	0.8975145	2.866093	fibronectin type III and SPRY domain containing 1
UP in U87	**10186**	LHFP	5.7702727	0.9888083	54.57895	lipoma HMGIC fusion partner
UP in U87	**23768**	FLRT2	3.3970908	0.9615357	10.5348	fibronectin leucine rich transmembrane
UP in U87	**196**	AHR	2.3219281	0.9430192	5	aryl hydrocarbon receptor
UP in U87	**5669**	PSG1	5.1756387	0.8584991	36.14286	pregnancy specific beta-1-glycoprotein 1
UP in U87	**8492**	PRSS12	6.9471986	0.8939143	123.4	protease, serine, 12 (neurotrypsin, motopsin)
UP in U87	**54749**	EPDR1	3.9785626	0.9845325	15.76401	ependymin related protein 1 (zebrafish)
UP in U87	**54896**	PQLC2	2.2680504	0.9372678	4.816718	PQ loop repeat containing 2
UP in U87	**9478**	CABP1	7.7210992	0.8982524	211	calcium binding protein 1
UP in U87	**4035**	LRP1	2.8431611	0.9650737	7.175907	low density lipoprotein receptor-related
UP in U87	**768211**	RELL1	2.5006027	0.9258094	5.659218	RELT-like 1
UP in U87	**79750**	ZNF385D	9.4525845	0.9803101	700.6667	zinc finger protein 385D
UP in U87	**10019**	SH2B3	6.5391128	0.9982577	92.99704	SH2B adaptor protein 3
UP in U87	**4684**	NCAM1	7.9351651	0.9418458	244.75	neural cell adhesion molecule 1
UP in U87	**678655**	CD27-AS1	2.3193572	0.9148399	4.991098	CD27 antisense RNA 1
UP in U87	**374875**	HSD11B1	2.4733943	0.8954789	5.553488	hydroxysteroid (11-beta) dehydrogenase 1-
UP in U87	**11226**	GALNT6	3.4598042	0.9689495	11.00284	UDP-N-acetyl-alpha-D-galactosamine:polypeptide N-acetylgalactosaminyltransferase 6 (GalNAc-T6)
UP in U87	**4627**	MYH9	1.4606223	0.9074884	2.752271	myosin, heavy chain 9, non-muscle
UP in U87	**84617**	TUBB6	2.4558884	0.9528064	5.486509	tubulin, beta 6 class V
UP in U87	**152742**	LOC15274	5.1515932	0.8156702	35.54545	uncharacterized LOC152742
UP in U87	**6672**	SP100	2.0382805	0.9365211	4.107557	SP100 nuclear antigen
UP in U87	**6348**	CCL3	12.122936	0.9982399	4460.333	chemokine (C-C motif) ligand 3
UP in U87	**3956**	LGALS1	2.7661276	0.9603534	6.802795	lectin, galactoside-binding, soluble, 1
UP in U87	**54331**	GNG2	8.7459544	0.9603979	429.3333	guanine nucleotide binding protein (G protein), gamma 2
UP in U87	**123016**	TTC8	1.2812751	0.8899497	2.430537	tetratricopeptide repeat domain 8
UP in U87	**8553**	BHLHE40	1.139041	0.8930432	2.202346	basic helix-loop-helix family, member e40
UP in U87	**2232**	FDXR	1.1701095	0.8778868	2.250288	ferredoxin reductase
UP in U87	**5600**	MAPK11	1.4056941	0.8860473	2.649452	mitogen-activated protein kinase 11
UP in U87	**643641**	ZNF862	4.3137076	0.9177467	19.88636	zinc finger protein 862
UP in U87	**24144**	TFIP11	1.1999721	0.888803	2.297352	tuftelin interacting protein 11
UP in U87	**2012**	EMP1	3.3553393	0.9758565	10.23429	epithelial membrane protein 1
UP in U87	**55969**	C20orf24	1.2137774	0.8974345	2.319441	chromosome 20 open reading frame 24
UP in U87	**23259**	DDHD2	1.2406873	0.8852028	2.363111	DDHD domain containing 2
UP in U87	**55176**	SEC61A2	1.9802796	0.9121464	3.945695	Sec61 alpha 2 subunit (S. cerevisiae)
UP in U87	**4482**	MSRA	1.3148963	0.8781802	2.487844	methionine sulfoxide reductase A
UP in U87	**11082**	ESM1	9.2344183	0.9755898	602.3333	endothelial cell-specific molecule 1
UP in U87	**677**	ZFP36L1	1.4866908	0.9062883	2.802454	ZFP36 ring finger protein-like 1
UP in U87	**338382**	RAB7B	5.7934872	0.9661137	55.46429	RAB7B, member RAS oncogene family
UP in U87	**55697**	VAC14	1.9064158	0.927125	3.748766	Vac14 homolog (S. cerevisiae)
UP in U87	**3679**	ITGA7	4.7724542	0.9835458	27.33077	integrin, alpha 7
UP in U87	**83862**	TMEM120	1.3176655	0.8959589	2.492624	transmembrane protein 120A
UP in U87	**64131**	XYLT1	4.0186866	0.9711007	16.20859	xylosyltransferase I
UP in U87	**26468**	LHX6	3.9771152	0.9651537	15.7482	LIM homeobox 6
UP in U87	**10627**	MYL12A	1.9165503	0.9292051	3.775193	myosin, light chain 12A, regulatory, non-sarcomeric
UP in U87	**2633**	GBP1	6.681824	0.9351965	102.6667	guanylate binding protein 1, interferon-

UP in U87	**5570**	PKIB	3.3781441	0.9417036	10.39735	protein kinase (cAMP-dependent, catalytic) inhibitor beta
UP in U87	**390940**	PINLYP	7.0061591	0.993493	128.5476	phospholipase A2 inhibitor and LY6/PLAUR domain containing
UP in U87	**645**	BLVRB	1.7082795	0.9252405	3.267709	biliverdin reductase B (flavin reductase (NADPH))
UP in U87	**29940**	DSE	2.3659937	0.946326	5.155076	dermatan sulfate epimerase
UP in U87	**9647**	PPM1F	1.9433114	0.9228759	3.845874	protein phosphatase, Mg2+/Mn2+ dependent,
UP in U87	**79096**	C11orf49	1.3986214	0.8983324	2.636495	chromosome 11 open reading frame 49
UP in U87	**91133**	L3MBTL4	7.5443205	0.8817626	186.6667	l(3)mbt-like 4 (Drosophila)
UP in U87	**84765**	ZNF577	7.2854022	0.8532366	156	zinc finger protein 577
UP in U87	**56906**	THAP10	1.2370392	0.8014827	2.357143	THAP domain containing 10
UP in U87	**134285**	TMEM171	6.2548139	0.9825591	76.36364	transmembrane protein 171
UP in U87	**5002**	SLC22A18	1.2315555	0.889523	2.3482	solute carrier family 22, member 18
UP in U87	**2556**	GABRA3	2.2668203	0.9239604	4.812613	gamma-aminobutyric acid (GABA) A receptor, alpha 3
UP in U87	**56917**	MEIS3	2.7751759	0.9558199	6.845595	Meis homeobox 3
UP in U87	**8110**	DPF3	4	0.9125287	16	D4, zinc and double PHD fingers, family 3
UP in U87	**3688**	ITGB1	2.3655637	0.9520686	5.15354	integrin, beta 1 (fibronectin receptor, beta polypeptide, antigen CD29 includes MDF2, MSK12)
UP in U87	**84719**	LINC0026	3.0700712	0.8939321	8.398148	long intergenic non-protein coding RNA 260
UP in U87	**7137**	TNNI3	4.6322682	0.8010116	24.8	troponin I type 3 (cardiac)
UP in U87	**1958**	EGR1	1.1960057	0.8075631	2.291045	early growth response 1
UP in U87	**8837**	CFLAR	1.1494732	0.8879585	2.218329	CASP8 and FADD-like apoptosis regulator
UP in U87	**83707**	TRPT1	1.3325103	0.8980301	2.518405	tRNA phosphotransferase 1
UP in U87	**29780**	PARVB	1.0713181	0.8871229	2.101352	parvin, beta
UP in U87	**9792**	SERTAD2	1.9440356	0.9169911	3.847805	SERTA domain containing 2
UP in U87	**51566**	ARMCX3	10.468217	0.9959198	1416.6	armadillo repeat containing, X-linked 3
UP in U87	**1117**	CHI3L2	8.9638594	0.9681227	499.3333	chitinase 3-like 2
UP in U87	**414236**	C10orf55	3.605382	0.9542376	12.17105	chromosome 10 open reading frame 55
UP in U87	**8800**	PEX11A	1.8131899	0.8937721	3.514184	peroxisomal biogenesis factor 11 alpha
UP in U87	**112616**	CMTM7	3.6490406	0.963847	12.545	CKLF-like MARVEL transmembrane domain containing 7
UP in U87	**56894**	AGPAT3	1.2329399	0.8843761	2.350455	1-acylglycerol-3-phosphate O-acyltransferase
UP in U87	**148741**	ANKRD35	9.2167459	0.9751987	595	ankyrin repeat domain 35
UP in U87	**23253**	ANKRD12	1.3159833	0.8281242	2.48972	ankyrin repeat domain 12
UP in U87	**3310**	HSPA6	5.1241213	0.8731932	34.875	heat shock 70kDa protein 6 (HSP70B')
UP in U87	**115948**	CCDC151	2.6064422	0.8284175	6.09	coiled-coil domain containing 151
UP in U87	**100233156**	LOC100233156	1.1843022	0.8540278	2.272534	tektin 4 pseudogene
UP in U87	**285148**	IAH1	7.7528923	0.9980888	215.7015	isoamyl acetate-hydrolyzing esterase 1 homolog (S. cerevisiae)
UP in U87	**286128**	ZFP41	1.17799	0.839387	2.262613	ZFP41 zinc finger protein
UP in U87	**9668**	ZNF432	3.195016	0.9465572	9.157895	zinc finger protein 432
UP in U87	**80380**	PDCD1LG	9.1489002	0.973323	567.6667	programmed cell death 1 ligand 2
UP in U87	**96459**	FNIP1	1.1186585	0.8643661	2.17145	folliculin interacting protein 1
UP in U87	**2124**	EVI2B	7.0645503	0.9374633	133.8571	ecotropic viral integration site 2B
UP in U87	**85019**	TMEM241	1.3013546	0.8453962	2.464602	transmembrane protein 241
UP in U87	**8799**	PEX11B	1.2937747	0.8938966	2.451687	peroxisomal biogenesis factor 11 beta
UP in U87	**8609**	KLF7	1.1022363	0.8524277	2.146872	Kruppel-like factor 7 (ubiquitous)
UP in U87	**5452**	POU2F2	8.3811103	0.9936619	333.4	POU class 2 homeobox 2
UP in U87	**3899**	AFF3	2.2521275	0.9041638	4.763848	AF4/FMR2 family, member 3
UP in U87	**9907**	AP5Z1	1.0346762	0.8705175	2.048654	adaptor-related protein complex 5, zeta 1
UP in U87	**6782**	HSPA13	2.0477844	0.9373922	4.134705	heat shock protein 70kDa family, member 13
UP in U87	**5595**	MAPK3	1.5950048	0.9208758	3.020955	mitogen-activated protein kinase 3

UP in U87	**55738**	ARFGAP1	1.1081204	0.8890785	2.155646	ADP-ribosylation factor GTPase activating protein 1
UP in U87	**53916**	RAB4B	4.0883321	0.9841147	17.01025	RAB4B, member RAS oncogene family
UP in U87	**59353**	TMEM35	5.2605276	0.8431116	38.33333	transmembrane protein 35
UP in U87	**196513**	DCP1B	1.7748695	0.9076929	3.422071	DCP1 decapping enzyme homolog B (S. cerevisiae)
UP in U87	**54531**	MIER2	1.7791319	0.9138176	3.432196	mesoderm induction early response 1, family member 2
UP in U87	**51309**	ARMCX1	10.794416	0.9943464	1776	armadillo repeat containing, X-linked 1
UP in U87	**101**	ADAM8	4.4647499	0.9811812	22.08125	ADAM metallopeptidase domain 8
UP in U87	**26088**	GGA1	1.1381268	0.8876207	2.200951	golgi-associated, gamma adaptin ear containing, ARF binding protein 1
UP in U87	**4012**	LNPEP	1.3280073	0.8739933	2.510557	leucyl/cystinyl aminopeptidase
UP in U87	**51715**	RAB23	1.4860435	0.9006258	2.801197	RAB23, member RAS oncogene family
UP in U87	**58494**	JAM2	5.4615455	0.9587089	44.06452	junctional adhesion molecule 2
UP in U87	**7345**	UCHL1	15.923606	1	62156	ubiquitin carboxyl-terminal esterase L1 (ubiquitin thiolesterase)
UP in U87	**54558**	SPATA6	6.2761244	0.9351788	77.5	spermatogenesis associated 6
UP in U87	**10570**	DPYSL4	2.8637541	0.8427294	7.27907	dihydropyrimidinase-like 4
UP in U87	**55812**	SPATA7	1.7192067	0.8577791	3.292553	spermatogenesis associated 7
UP in U87	**10916**	MAGED2	1.8078779	0.927045	3.501269	melanoma antigen family D, 2
UP in U87	**245972**	ATP6V0D 2	8.4152136	0.9862037	341.375	ATPase, H+ transporting, lysosomal 38kDa, V0 subunit d2
UP in U87	**127700**	OSCP1	2.2562801	0.9240048	4.77758	organic solute carrier partner 1
UP in U87	**143187**	VTI1A	1.113045	0.8705798	2.163017	vesicle transport through interaction with t-SNAREs 1A
UP in U87	**8895**	CPNE3	1.7011649	0.9218803	3.251634	copine III
UP in U87	**7769**	ZNF226	1.1382395	0.8553967	2.201123	zinc finger protein 226
UP in U87	**444**	ASPH	1.1744737	0.8962967	2.257105	aspartate beta-hydroxylase
UP in U87	**10531**	PITRM1	2.0039646	0.9386545	4.011007	pitrilysin metallopeptidase 1
UP in U87	**136**	ADORA2B	1.5327954	0.9025192	2.893459	adenosine A2b receptor
UP in U87	**308**	ANXA5	1.0081748	0.8873718	2.011365	annexin A5
UP in U87	**8506**	CNTNAP1	1.4099994	0.8902075	2.657371	contactin associated protein 1
UP in U87	**127829**	ARL8A	1.2016339	0.8871495	2.3	ADP-ribosylation factor-like 8A
UP in U87	**170692**	ADAMTS 18	9.3339007	0.9780699	645.3333	ADAM metallopeptidase with thrombospondin type 1 motif, 18
UP in U87	**79632**	FAM184A	6.8629472	0.8858872	116.4	family with sequence similarity 184, member
UP in U87	**29926**	GMPPA	1.026246	0.8844472	2.036718	GDP-mannose pyrophosphorylase A
UP in U87	**148523**	C1orf51	5.7782086	0.9599534	54.88	chromosome 1 open reading frame 51
UP in U87	**860**	RUNX2	2.6262695	0.9189468	6.174274	runt-related transcription factor 2
UP in U87	**7084**	TK2	2.1563928	0.9294718	4.457988	thymidine kinase 2, mitochondrial
UP in U87	**253461**	ZBTB38	1.6964772	0.917889	3.241086	zinc finger and BTB domain containing 38
UP in U87	**7076**	TIMP1	3.3414011	0.9759365	10.13589	TIMP metallopeptidase inhibitor 1
UP in U87	**58515**	SELK	1.0154836	0.8836382	2.02158	selenoprotein K
UP in U87	**26751**	SH3YL1	4.7004397	0.9752609	26	SH3 domain containing, Ysc84-like 1 (S. cerevisiae)
UP in U87	**84302**	TMEM246	3.1495135	0.9630736	8.873563	transmembrane protein 246
UP in U87	**55824**	PAG1	1.2526553	0.8011716	2.382796	phosphoprotein associated with glycosphingolipid microdomains 1
UP in U87	**604**	BCL6	2.0782923	0.9325653	4.22307	B-cell CLL/lymphoma 6
UP in U87	**5798**	PTPRN	4.1654885	0.9861059	17.94473	protein tyrosine phosphatase, receptor type,
UP in U87	**3082**	HGF	9.3677787	0.97887	660.6667	hepatocyte growth factor (hepapoietin A; scatter factor)
UP in U87	**4189**	DNAJB9	1.6448474	0.9008925	3.127148	DnaJ (Hsp40) homolog, subfamily B,
UP in U87	**57787**	MARK4	2.5303562	0.93813	5.777143	MAP/microtubule affinity-regulating kinase 4
UP in U87	**374659**	HDDC3	1.1161907	0.8748378	2.167738	HD domain containing 3
UP in U87	**91039**	DPP9	1.3275535	0.9010169	2.509767	dipeptidyl-peptidase 9

UP in U87	**85440**	DOCK7	1.8557517	0.9172223	3.619403	dedicator of cytokinesis 7
UP in U87	**3233**	HOXD4	7.0516621	0.8262396	132.6667	homeobox D4
UP in U87	**254263**	CNIH2	3.6692788	0.8802425	12.72222	cornichon homolog 2 (Drosophila)
UP in U87	**3790**	KCNS3	3.8943249	0.9738742	14.86992	potassium voltage-gated channel, delayed-rectifier, subfamily S, member 3
UP in U87	**27040**	LAT	4.6766901	0.9906484	25.57549	linker for activation of T cells
UP in U87	**2827**	GPR3	1.9594885	0.866624	3.889241	G protein-coupled receptor 3
UP in U87	**1476**	CSTB	3.5266165	0.97863	11.52437	cystatin B (stefin B)
UP in U87	**222235**	FBXL13	4.7032115	0.9617846	26.05	F-box and leucine-rich repeat protein 13
UP in U87	**2245**	FGD1	2.5604956	0.9349832	5.899103	FYVE, RhoGEF and PH domain containing
UP in U87	**4313**	MMP2	3.3130665	0.9731186	9.938764	matrix metallopeptidase 2 (gelatinase A, 72kDa gelatinase, 72kDa type IV collagenase)
UP in U87	**157753**	TMEM74	2.708739	0.8069319	6.5375	transmembrane protein 74
UP in U87	**51586**	MED15	1.4081697	0.9020303	2.654002	mediator complex subunit 15
UP in U87	**115265**	DDIT4L	8.1782499	0.9322719	289.6667	DNA-damage-inducible transcript 4-like
UP in U87	**55026**	TMEM255	7.6888331	0.8950433	206.3333	transmembrane protein 255A
UP in U87	**54868**	TMEM104	1.3455221	0.8833716	2.541221	transmembrane protein 104
UP in U87	**58485**	TRAPPC1	1.4393732	0.9064395	2.71203	trafficking protein particle complex 1
UP in U87	**7728**	ZNF175	3.4532113	0.9695806	10.95267	zinc finger protein 175
UP in U87	**2059**	EPS8	2.0785887	0.9139954	4.223938	epidermal growth factor receptor pathway substrate 8
UP in U87	**100506311**	HOTAIRM 1	4.8374183	0.9872793	28.5896	HOXA transcript antisense RNA, myeloid-specific 1
UP in U87	**84910**	TMEM87B	2.7477661	0.9519174	6.716763	transmembrane protein 87B
UP in U87	**201163**	FLCN	1.0408531	0.8775135	2.057444	folliculin
UP in U87	**5829**	PXN	1.2895177	0.9006969	2.444463	paxillin
UP in U87	**57148**	RALGAPB	1.3976495	0.8999236	2.63472	Ral GTPase activating protein, beta subunit (non-catalytic)
UP in U87	**7220**	TRPC1	1.7013772	0.8966878	3.252113	transient receptor potential cation channel, subfamily C, member 1
UP in U87	**51523**	CXXC5	2.2993191	0.941108	4.922254	CXXC finger protein 5
UP in U87	**51548**	SIRT6	1.0901535	0.8815937	2.128967	sirtuin 6
UP in U87	**171490**	SPANXF1	4.3949573	0.8626593	21.03846	SPANX family, member F1
UP in U87	**3127**	HLA-DRB5	12.592924	0.9990488	6178	major histocompatibility complex, class II, DR beta 5
UP in U87	**7094**	TLN1	1.6575346	0.9243693	3.15477	talin 1
UP in U87	**1847**	DUSP5	4.2432094	0.9848792	18.93797	dual specificity phosphatase 5
UP in U87	**26585**	GREM1	8.3224352	0.9996266	320.1125	gremlin 1, DAN family BMP antagonist
UP in U87	**11031**	RAB31	1.8733978	0.9281828	3.663945	RAB31, member RAS oncogene family
UP in U87	**8651**	SOCS1	2.9084328	0.9495529	7.508021	suppressor of cytokine signaling 1
UP in U87	**22854**	NTNG1	9.447772	0.9802212	698.3333	netrin G1
UP in U87	**93349**	SP140L	5.7208459	0.9837147	52.74074	SP140 nuclear body protein-like
UP in U87	**1727**	CYB5R3	1.1012325	0.8915676	2.145379	cytochrome b5 reductase 3
UP in U87	**85012**	TCEAL3	8.1353718	0.9972799	281.1842	transcription elongation factor A (SII)-like 3
UP in U87	**100529261**	CHURC1-FNTB	1.2965725	0.8308265	2.456446	CHURC1-FNTB readthrough
UP in U87	**55450**	CAMK2N 1	2.1996665	0.9377833	4.593732	calcium/calmodulin-dependent protein kinase II inhibitor 1
UP in U87	**53840**	TRIM34	4.1578899	0.9633936	17.85047	tripartite motif containing 34
UP in U87	**5305**	PIP4K2A	2.2285894	0.9375344	4.686755	phosphatidylinositol-5-phosphate 4-kinase, type II, alpha
UP in U87	**285598**	ARL10	5.7813597	0.8582947	55	ADP-ribosylation factor-like 10
UP in U87	**23052**	ENDOD1	1.8011364	0.9100663	3.484946	endonuclease domain containing 1
UP in U87	**154860**	FEZF1-	7.7791722	0.902937	219.6667	FEZF1 antisense RNA 1
UP in U87	**4693**	NDP	9.8846793	0.9870393	945.3333	Norrie disease (pseudoglioma)

UP in U87	**4793**	NFKBIB	1.0817712	0.8828649	2.116633	nuclear factor of kappa light polypeptide gene enhancer in B-cells inhibitor, beta
UP in U87	**1795**	DOCK3	2.5268595	0.9035326	5.763158	dedicator of cytokinesis 3
UP in U87	**22979**	EFR3B	4.5757636	0.9554465	23.84746	EFR3 homolog B (S. cerevisiae)
UP in U87	**9950**	GOLGA5	1.219387	0.8924298	2.328478	golgin A5
UP in U87	**79713**	IGFLR1	1.609	0.8691752	3.050403	IGF-like family receptor 1
UP in U87	**2825**	GPR1	2.1471537	0.8107366	4.42953	G protein-coupled receptor 1
UP in U87	**147645**	VSIG10L	1.4106028	0.8234395	2.658482	V-set and immunoglobulin domain containing 10 like
UP in U87	**8717**	TRADD	2.5740757	0.9483261	5.954894	TNFRSF1A-associated via death domain
UP in U87	**8878**	SQSTM1	1.5640848	0.9099952	2.956899	sequestosome 1
UP in U87	**284131**	ENDOV	1.3700271	0.8751756	2.584754	endonuclease V
UP in U87	**1821**	DRP2	3.1576987	0.8678418	8.924051	dystrophin related protein 2
UP in U87	**1012**	CDH13	4.0646105	0.9852081	16.73284	cadherin 13, H-cadherin (heart)
UP in U87	**284021**	MILR1	5.5499481	0.9806212	46.84906	mast cell immunoglobulin-like receptor 1
UP in U87	**5028**	P2RY1	6.8538294	0.8007805	115.6667	purinergic receptor P2Y, G-protein coupled,
UP in U87	**56097**	PCDHGC5	8.0552824	0.9235781	266	protocadherin gamma subfamily C, 5
UP in U87	**100288695**	LIMS3L	3.6449985	0.9639625	12.5099	LIM and senescent cell antigen-like domains 3-like
UP in U87	**254102**	EHBP1L1	1.8966941	0.9270539	3.72359	EH domain binding protein 1-like 1
UP in U87	**50808**	AK3	1.1147092	0.8853628	2.165514	adenylate kinase 3
UP in U87	**8862**	APLN	3.5214201	0.9702651	11.48294	apelin
UP in U87	**64748**	LPPR2	2.5571585	0.9512685	5.885474	lipid phosphate phosphatase-related protein type 2
UP in U87	**81029**	WNT5B	3.281503	0.8776557	9.723684	wingless-type MMTV integration site family, member 5B
UP in U87	**84251**	SGIP1	7.1665816	0.8397603	143.6667	SH3-domain GRB2-like (endophilin) interacting protein 1
UP in U87	**80830**	APOL6	2.7833094	0.8732199	6.884298	apolipoprotein L, 6
UP in U87	**3098**	HK1	1.73585	0.9263605	3.330757	hexokinase 1
UP in U87	**100861555**	LINC0056	4.7246503	0.8930876	26.44	long intergenic non-protein coding RNA 565
UP in U87	**1261**	CNGA3	8.3074285	0.970194	316.8	cyclic nucleotide gated channel alpha 3
UP in U87	**53358**	SHC3	7.6969675	0.9840258	207.5	SHC (Src homology 2 domain containing) transforming protein 3
UP in U87	**2318**	FLNC	12.504661	0.9999111	5811.364	filamin C, gamma
UP in U87	**9140**	ATG12	1.6031649	0.9156133	3.038091	autophagy related 12
UP in U87	**55924**	FAM212B	3.8083649	0.9698473	14.0098	family with sequence similarity 212, member
UP in U87	**6884**	TAF13	2.0888069	0.9400946	4.253961	TAF13 RNA polymerase II, TATA box binding protein (TBP)-associated factor, 18kDa
UP in U87	**25832**	NBPF14	1.0434269	0.8816915	2.061118	neuroblastoma breakpoint family, member 14
UP in U87	**7130**	TNFAIP6	8.5673223	0.9532508	379.3333	tumor necrosis factor, alpha-induced protein
UP in U87	**26580**	BSCL2	4.1243096	0.9855637	17.43978	Berardinelli-Seip congenital lipodystrophy 2 (seipin)
UP in U87	**7016**	TESK1	1.9563593	0.9237293	3.880814	testis-specific kinase 1
UP in U87	**3772**	KCNJ15	4.9098931	0.8481786	30.0625	potassium inwardly-rectifying channel, subfamily J, member 15
UP in U87	**10247**	HRSP12	1.3080524	0.8960122	2.476071	heat-responsive protein 12
UP in U87	**4494**	MT1F	2.3460636	0.948175	5.084351	metallothionein 1F
UP in U87	**400043**	LOC40004	6.1935587	0.9839369	73.18919	uncharacterized LOC400043
UP in U87	**130888**	FBXO36	3.7757278	0.9484861	13.69643	F-box protein 36
UP in U87	**3755**	KCNG1	3.3932847	0.955411	10.50704	potassium voltage-gated channel, subfamily G, member 1
UP in U87	**55848**	PLGRKT	3.073833	0.9659004	8.420074	plasminogen receptor, C-terminal lysine transmembrane protein
UP in U87	**24138**	IFIT5	2.0227389	0.9170445	4.063545	interferon-induced protein with tetratricopeptide repeats 5

UP in U87	**133396**	IL31RA	10.088347	0.9892528	1088.667	interleukin 31 receptor A
UP in U87	**261729**	STEAP2	3.2998053	0.9573044	9.847826	STEAP family member 2, metalloreductase
UP in U87	**9119**	KRT75	7.8466657	0.9904706	230.1875	keratin 75
UP in U87	**23315**	SLC9A8	2.7172217	0.950264	6.576052	solute carrier family 9, subfamily A (NHE8, cation proton antiporter 8), member 8
UP in U87	**51106**	TFB1M	1.1125538	0.8636994	2.162281	transcription factor B1, mitochondrial
UP in U87	**794**	CALB2	8.4635244	0.9479972	353	calbindin 2
UP in U87	**1609**	DGKQ	1.5791685	0.8670329	2.987976	diacylglycerol kinase, theta 110kDa
UP in U87	**1780**	DYNC1I1	4.7925579	0.9115064	27.71429	dynein, cytoplasmic 1, intermediate chain 1
UP in U87	**112574**	SNX18	2.1881601	0.9230537	4.557239	sorting nexin 18
UP in U87	**84282**	RNF135	1.36649	0.8881807	2.578425	ring finger protein 135
UP in U87	**80271**	ITPKC	2.1046974	0.9109553	4.301075	inositol-trisphosphate 3-kinase C
UP in U87	**23531**	MMD	1.690996	0.9137732	3.228795	monocyte to macrophage differentiation-associated
UP in U87	**51438**	MAGEC2	9.4861167	0.9938397	717.1429	melanoma antigen family C, 2
UP in U87	**155054**	ZNF425	3.5420907	0.8047363	11.64865	zinc finger protein 425
UP in U87	**80195**	TMEM254	1.0824273	0.8521077	2.117596	transmembrane protein 254
UP in U87	**84695**	LOXL3	2.9143226	0.9615891	7.538736	lysyl oxidase-like 3
UP in U87	**2355**	FOSL2	1.3541814	0.9009103	2.55652	FOS-like antigen 2
UP in U87	**5328**	PLAU	4.5895125	0.992524	24.07581	plasminogen activator, urokinase
UP in U87	**83638**	C11orf68	2.367089	0.9507085	5.158991	chromosome 11 open reading frame 68
UP in U87	**593**	BCKDHA	1.0576525	0.8847761	2.081542	branched chain keto acid dehydrogenase E1, alpha polypeptide
UP in U87	**10123**	ARL4C	5.8649599	0.9941597	58.28125	ADP-ribosylation factor-like 4C
UP in U87	**640**	BLK	10.891277	0.9946842	1899.333	B lymphoid tyrosine kinase
UP in U87	**147650**	LINC0008	1.7016004	0.894261	3.252616	long intergenic non-protein coding RNA 85
UP in U87	**1464**	CSPG4	1.3830858	0.8995058	2.608257	chondroitin sulfate proteoglycan 4
UP in U87	**7058**	THBS2	8.4991815	0.9810123	361.8333	thrombospondin 2
UP in U87	**4354**	MPP1	3.0688787	0.965127	8.391209	membrane protein, palmitoylated 1, 55kDa
UP in U87	**56929**	FEM1C	1.3870707	0.8719932	2.615471	fem-1 homolog c (C. elegans)
UP in U87	**3053**	SERPIND1	6.1241213	0.9488951	69.75	serpin peptidase inhibitor, clade D (heparin cofactor), member 1
UP in U87	**23382**	AHCYL2	1.6582673	0.9113108	3.156372	adenosylhomocysteinase-like 2
UP in U87	**23639**	LRRC6	3.2952677	0.8714065	9.816901	leucine rich repeat containing 6
UP in U87	**199692**	ZNF627	1.6492357	0.8637972	3.136674	zinc finger protein 627
UP in U87	**80263**	TRIM45	1.3437275	0.8382669	2.538062	tripartite motif containing 45
UP in U87	**2335**	FN1	5.3643885	0.995822	41.19475	fibronectin 1
UP in U87	**57085**	AGTRAP	1.6126928	0.9223959	3.058221	angiotensin II receptor-associated protein
UP in U87	**6304**	SATB1	2.70789	0.9145643	6.533654	SATB homeobox 1
UP in U87	**387647**	PTCHD3P	1.2085866	0.8687486	2.311111	patched domain containing 3 pseudogene 1
UP in U87	**6237**	RRAS	3.7833871	0.9821857	13.76934	related RAS viral (r-ras) oncogene homolog
UP in U87	**7477**	WNT7B	4.0163499	0.9856081	16.18236	wingless-type MMTV integration site family, member 7B
UP in U87	**55304**	SPTLC3	2.2920016	0.8992035	4.897351	serine palmitoyltransferase, long chain base subunit 3
UP in U87	**137392**	FAM92A1	1.4427219	0.8958344	2.718332	family with sequence similarity 92, member
UP in U87	**114548**	NLRP3	9.0507556	0.9706562	530.3333	NLR family, pyrin domain containing 3
UP in U87	**51131**	PHF11	1.8148355	0.902217	3.518195	PHD finger protein 11
UP in U87	**3557**	IL1RN	8.8381534	0.9638025	457.6667	interleukin 1 receptor antagonist
UP in U87	**89782**	LMLN	3.2967398	0.9102974	9.826923	leishmanolysin-like (metallopeptidase M8
UP in U87	**5583**	PRKCH	5.5484366	0.8485341	46.8	protein kinase C, eta
UP in U87	**2588**	GALNS	1.9200589	0.9251605	3.784385	galactosamine (N-acetyl)-6-sulfate sulfatase
UP in U87	**53918**	PELO	1.5572119	0.9036749	2.942846	pelota homolog (Drosophila)
UP in U87	**203259**	FAM219A	2.7193399	0.9546642	6.585714	family with sequence similarity 219, member
UP in U87	**122830**	NAA30	1.4385324	0.8901364	2.71045	N(alpha)-acetyltransferase 30, NatC catalytic subunit

UP in U87	**528**	ATP6V1C1	1.0678106	0.8846961	2.09625	ATPase, H+ transporting, lysosomal 42kDa, V1 subunit C1
UP in U87	**8728**	ADAM19	3.0314782	0.9005458	8.176471	ADAM metallopeptidase domain 19
UP in U87	**7378**	UPP1	1.6081531	0.9195246	3.048613	uridine phosphorylase 1
UP in U87	**23559**	WBP1	1.0721569	0.8852028	2.102574	WW domain binding protein 1
UP in U87	**378**	ARF4	1.413435	0.9051949	2.663706	ADP-ribosylation factor 4
UP in U87	**56776**	FMN2	7.1980357	0.9326097	146.8333	formin 2
UP in U87	**154743**	C7orf60	1.3945006	0.8698331	2.628975	chromosome 7 open reading frame 60
UP in U87	**92689**	FAM114A	3.6440375	0.9785766	12.50157	family with sequence similarity 114, member
UP in U87	**5510**	PPP1R7	1.1531075	0.8928387	2.223924	protein phosphatase 1, regulatory subunit 7
UP in U87	**9783**	RIMS3	5.5354424	0.992524	46.38037	regulating synaptic membrane exocytosis 3
UP in U87	**57522**	SRGAP1	1.2378426	0.8675574	2.358456	SLIT-ROBO Rho GTPase activating protein
UP in U87	**6769**	STAC	3.5463654	0.9714829	11.68322	SH3 and cysteine rich domain
UP in U87	**140606**	SELM	1.2654428	0.8977723	2.40401	selenoprotein M
UP in U87	**23616**	SH3BP1	5.2323475	0.9709407	37.59184	SH3-domain binding protein 1
UP in U87	**79686**	LINC0034	3.0083249	0.8894697	8.046296	long intergenic non-protein coding RNA 341
UP in U87	**375133**	PI4KAP2	1.7075593	0.901257	3.266078	phosphatidylinositol 4-kinase, catalytic, alpha pseudogene 2
UP in U87	**3176**	HNMT	6.6537408	0.9693673	100.6875	histamine N-methyltransferase
UP in U87	**734**	OSGIN2	1.9810437	0.9229203	3.947786	oxidative stress induced growth inhibitor family member 2
UP in U87	**2621**	GAS6	1.531447	0.8939321	2.890756	growth arrest-specific 6
UP in U87	**9890**	LPPR4	8.659104	0.9712251	404.25	lipid phosphate phosphatase-related protein type 4
UP in U87	**56998**	CTNNBIP	1.1471397	0.815119	2.214744	catenin, beta interacting protein 1
UP in U87	**5806**	PTX3	7.2775933	0.9941775	155.1579	pentraxin 3, long
UP in U87	**91283**	MSANTD3	1.3383692	0.8999058	2.528653	Myb/SANT-like DNA-binding domain containing 3
UP in U87	**170371**	C10orf128	10.834734	0.9944708	1826.333	chromosome 10 open reading frame 128
UP in U87	**83666**	PARP9	1.4596795	0.8457784	2.750473	poly (ADP-ribose) polymerase family,
UP in U87	**23189**	KANK1	2.555631	0.9145021	5.879245	KN motif and ankyrin repeat domains 1
UP in U87	**9659**	PDE4DIP	1.7072397	0.9241382	3.265355	phosphodiesterase 4D interacting protein
UP in U87	**5359**	PLSCR1	1.0109484	0.8553434	2.015235	phospholipid scramblase 1
UP in U87	**26119**	LDLRAP1	1.5523411	0.9000658	2.932927	low density lipoprotein receptor adaptor
UP in U87	**79575**	ABHD8	2.172693	0.9348765	4.508642	abhydrolase domain containing 8
UP in U87	**1893**	ECM1	6.3541036	0.9967554	81.80423	extracellular matrix protein 1
UP in U87	**3572**	IL6ST	1.7528549	0.9231603	3.370248	interleukin 6 signal transducer (gp130, oncostatin M receptor)
UP in U87	**475**	ATOX1	1.7355527	0.9263694	3.33007	ATX1 antioxidant protein 1 homolog (yeast)
UP in U87	**4047**	LSS	1.406195	0.9021014	2.650372	lanosterol synthase (2,3-oxidosqualene-lanosterol cyclase)
UP in U87	**23266**	LPHN2	5.777212	0.9430636	54.84211	latrophilin 2
UP in U87	**1195**	CLK1	1.2557549	0.893461	2.387921	CDC-like kinase 1
UP in U87	**5646**	PRSS3	11.158399	0.9958042	2285.667	protease, serine, 3
UP in U87	**79815**	NIPAL2	6.5545889	0.9006525	94	NIPA-like domain containing 2
UP in U87	**55277**	FGGY	2.6825733	0.9317741	6.42	FGGY carbohydrate kinase domain
UP in U87	**81928**	CABLES2	2.5239123	0.9267161	5.751397	Cdk5 and Abl enzyme substrate 2
UP in U87	**7431**	VIM	3.2660973	0.9731452	9.620403	vimentin
UP in U87	**84791**	LINC0046	3.0187794	0.9541931	8.104816	long intergenic non-protein coding RNA 467
UP in U87	**79651**	RHBDF2	2.2866156	0.925676	4.879102	rhomboid 5 homolog 2 (Drosophila)
UP in U87	**2963**	GTF2F2	1.3635807	0.9012214	2.573231	general transcription factor IIF, polypeptide 2, 30kDa
UP in U87	**115294**	PCMTD1	1.1883087	0.8662329	2.278854	protein-L-isoaspartate (D-aspartate) O-methyltransferase domain containing 1
UP in U87	**51286**	CEND1	3.2388596	0.9678116	9.440476	cell cycle exit and neuronal differentiation 1
UP in U87	**11107**	PRDM5	6.9068906	0.8074386	120	PR domain containing 5
UP in U87	**1116**	CHI3L1	7.5381889	0.9672427	185.875	chitinase 3-like 1 (cartilage glycoprotein-39)

UP in U87	**58538**	MPP4	6.3253055	0.9579089	80.1875	membrane protein, palmitoylated 4 (MAGUK p55 subfamily member 4)
UP in U87	**9946**	CRYZL1	1.0181134	0.865824	2.025269	crystallin, zeta (quinone reductase)-like 1
UP in U87	**5915**	RARB	7.0326886	0.9775277	130.9333	retinoic acid receptor, beta
UP in U87	**646329**	LOC64632	4.5849625	0.9455615	24	uncharacterized LOC646329
UP in U87	**10404**	CPQ	3.7950933	0.9689317	13.88152	carboxypeptidase Q
UP in U87	**645323**	LINC0046	8.2223924	0.9345832	298.6667	long intergenic non-protein coding RNA 461
UP in U87	**57728**	WDR19	1.1650928	0.8633616	2.242476	WD repeat domain 19
UP in U87	**126129**	CPT1C	5.1517122	0.9875282	35.54839	carnitine palmitoyltransferase 1C
UP in U87	**9582**	APOBEC3 B	2.0394294	0.9369922	4.110829	apolipoprotein B mRNA editing enzyme, catalytic polypeptide-like 3B
UP in U87	**1666**	DECR1	1.142346	0.889683	2.207397	2,4-dienoyl CoA reductase 1, mitochondrial
UP in U87	**80318**	GKAP1	1.4570936	0.8165858	2.745547	G kinase anchoring protein 1
UP in U87	**5783**	PTPN13	8.0570892	0.9235781	266.3333	protein tyrosine phosphatase, non-receptor type 13 (APO-1/CD95 (Fas)-associated phosphatase)
UP in U87	**64342**	HS1BP3	1.6682832	0.9192668	3.178361	HCLS1 binding protein 3
UP in U87	**3198**	HOXA1	7.7813597	0.902937	220	homeobox A1
UP in U87	**25865**	PRKD2	1.389808	0.8926787	2.620438	protein kinase D2
UP in U87	**79991**	OBFC1	1.3629469	0.8487475	2.5721	oligonucleotide/oligosaccharide-binding fold containing 1
UP in U87	**667**	DST	1.1982866	0.8889007	2.29467	dystonin
UP in U87	**83475**	DOHH	1.7655946	0.9168133	3.400141	deoxyhypusine hydroxylase/monooxygenase
UP in U87	**7980**	TFPI2	6.3963373	0.9981777	84.23438	tissue factor pathway inhibitor 2
UP in U87	**7035**	TFPI	1.4855197	0.9057016	2.80018	tissue factor pathway inhibitor (lipoprotein-associated coagulation inhibitor)
UP in U87	**55529**	TMEM55	2.4997538	0.9449037	5.655889	transmembrane protein 55A
UP in U87	**375287**	RBM43	2.375923	0.8956922	5.190678	RNA binding motif protein 43
UP in U87	**56994**	CHPT1	1.2466486	0.8964834	2.372895	choline phosphotransferase 1
UP in U87	**8178**	ELL	1.7581821	0.9097374	3.382716	elongation factor RNA polymerase II
UP in U87	**284371**	ZNF841	2.2016339	0.9078884	4.6	zinc finger protein 841
UP in U87	**285381**	DPH3	1.1117991	0.8788291	2.16115	DPH3, KTI11 homolog (S. cerevisiae)
UP in U87	**100526694**	MSANTD 3-TMEFF1	2.1033057	0.8832027	4.296928	MSANTD3-TMEFF1 readthrough
UP in U87	**23645**	PPP1R15A	3.0741842	0.9685139	8.422125	protein phosphatase 1, regulatory subunit
UP in U87	**100506211**	MIR210H	1.4872658	0.8341423	2.803571	MIR210 host gene (non-protein coding)
UP in U87	**169792**	GLIS3	6.9937247	0.9890305	127.4444	GLIS family zinc finger 3
UP in U87	**644168**	DRGX	7.8030548	0.9044838	223.3333	dorsal root ganglia homeobox
UP in U87	**9734**	HDAC9	5.3445768	0.9843636	40.63291	histone deacetylase 9
UP in U87	**9094**	UNC119	1.8405639	0.922467	3.5815	unc-119 homolog (C. elegans)
UP in U87	**219541**	MED19	1.2095904	0.8748022	2.31272	mediator complex subunit 19
UP in U87	**283417**	DPY19L2	6.0752881	0.9360144	67.42857	dpy-19-like 2 (C. elegans)
UP in U87	**57515**	SERINC1	1.0715316	0.8892385	2.101663	serine incorporator 1
UP in U87	**390**	RND3	4.0279706	0.9865682	16.31323	Rho family GTPase 3
UP in U87	**945**	CD33	5.9209175	0.9713229	60.58621	CD33 molecule
UP in U87	**11142**	PKIG	2.0745933	0.9377033	4.212256	protein kinase (cAMP-dependent, catalytic) inhibitor gamma
UP in U87	**4338**	MOCS2	1.524134	0.9063328	2.87614	molybdenum cofactor synthesis 2
UP in U87	**3037**	HAS2	4.8551623	0.9619269	28.9434	hyaluronan synthase 2
UP in U87	**22809**	ATF5	1.9439467	0.9259961	3.847568	activating transcription factor 5
UP in U87	**5366**	PMAIP1	2.2566327	0.9375256	4.778748	phorbol-12-myristate-13-acetate-induced protein 1
UP in U87	**220**	ALDH1A3	11.42217	0.9998044	2744.2	aldehyde dehydrogenase 1 family, member
UP in U87	**439921**	MXRA7	1.7740031	0.9267428	3.420016	matrix-remodelling associated 7
UP in U87	**119548**	PNLIPRP3	8.7199592	0.9595267	421.6667	pancreatic lipase-related protein 3
UP in U87	**94115**	CGB8	6.2657453	0.9845858	76.94444	chorionic gonadotropin, beta polypeptide 8

UP in U87	**1573**	CYP2J2	7.5520286	0.8824382	187.6667	cytochrome P450, family 2, subfamily J, polypeptide 2
UP in U87	**3725**	JUN	5.9898634	0.9971554	63.5519	jun proto-oncogene
UP in U87	**2217**	FCGRT	2.7389139	0.9376145	6.675676	Fc fragment of IgG, receptor, transporter,
UP in U87	**10630**	PDPN	10.616855	0.9933241	1570.333	podoplanin
UP in U87	**54505**	DHX29	1.8061309	0.9211336	3.497032	DEAH (Asp-Glu-Ala-His) box polypeptide
UP in U87	**58513**	EPS15L1	1.0249701	0.8754156	2.034917	epidermal growth factor receptor pathway substrate 15-like 1
UP in U87	**65977**	PLEKHA3	1.1644176	0.8859584	2.241427	pleckstrin homology domain containing, family A (phosphoinositide binding specific) member 3
UP in U87	**90529**	STPG1	1.3663045	0.8267641	2.578093	sperm-tail PG-rich repeat containing 1
UP in U87	**56271**	BEX4	9.7954986	0.9859637	888.6667	brain expressed, X-linked 4
UP in U87	**9180**	OSMR	1.4565055	0.9009103	2.744428	oncostatin M receptor
UP in U87	**150967**	PKI55	1.0113953	0.8456807	2.01586	DKFZp434H1419
UP in U87	**1645**	AKR1C1	3.9436997	0.9843458	15.38764	aldo-keto reductase family 1, member C1
UP in U87	**51339**	DACT1	8.2223924	0.9345832	298.6667	dapper, antagonist of beta-catenin, homolog 1 (Xenopus laevis)
UP in U87	**79016**	DDA1	1.3005598	0.8920298	2.463244	DET1 and DDB1 associated 1
UP in U87	**2941**	GSTA4	2.7187364	0.9191157	6.58296	glutathione S-transferase alpha 4
UP in U87	**29104**	N6AMT1	1.6770807	0.9043238	3.197802	N-6 adenine-specific DNA methyltransferase 1 (putative)
UP in U87	**3749**	KCNC4	1.1972323	0.8634861	2.292994	potassium voltage-gated channel, Shaw-related subfamily, member 4
UP in U87	**3115**	HLA-DPB1	8.9068906	0.9835636	480	major histocompatibility complex, class II, DP beta 1
UP in U87	**11059**	WWP1	1.6031198	0.9142888	3.037996	WW domain containing E3 ubiquitin protein ligase 1
UP in U87	**128439**	SNHG11	1.2563212	0.8852028	2.388858	small nucleolar RNA host gene 11 (non-protein coding)
UP in U87	**79864**	C11orf63	4.3102463	0.878358	19.83871	chromosome 11 open reading frame 63
UP in U87	**22848**	AAK1	1.9461371	0.8957189	3.853414	AP2 associated kinase 1
UP in U87	**4240**	MFGE8	2.4271905	0.9492417	5.37845	milk fat globule-EGF factor 8 protein
UP in U87	**2131**	EXT1	2.629451	0.9570822	6.187905	exostosin glycosyltransferase 1
UP in U87	**119710**	C11orf74	3.2845676	0.9663627	9.744361	chromosome 11 open reading frame 74
UP in U87	**4636**	MYL5	1.9567905	0.8928298	3.881974	myosin, light chain 5, regulatory
UP in U87	**728431**	LOC72843	1.966307	0.9026704	3.907666	uncharacterized LOC728431
UP in U87	**10133**	OPTN	2.9898098	0.9636958	7.943692	optineurin
UP in U87	**112464**	PRKCDBP	1.6708742	0.9233737	3.184075	protein kinase C, delta binding protein
UP in U87	**3281**	HSBP1	1.3704008	0.9021103	2.585424	heat shock factor binding protein 1
UP in U87	**168667**	BMPER	4.7782086	0.897399	27.44	BMP binding endothelial regulator
UP in U87	**56603**	CYP26B1	2.4034221	0.9059328	5.290566	cytochrome P450, family 26, subfamily B, polypeptide 1
UP in U87	**266812**	NAP1L5	9.4015906	0.9793678	676.3333	nucleosome assembly protein 1-like 5
UP in U87	**388552**	BLOC1S3	1.4297749	0.8888563	2.694047	biogenesis of lysosomal organelles complex-1, subunit 3
UP in U87	**79744**	ZNF419	1.5221025	0.8509432	2.872093	zinc finger protein 419
UP in U87	**89846**	FGD3	4.7954612	0.9867726	27.77011	FYVE, RhoGEF and PH domain containing
UP in U87	**2760**	GM2A	2.0244392	0.9363611	4.068337	GM2 ganglioside activator
UP in U87	**843**	CASP10	3.3115862	0.8390403	9.928571	caspase 10, apoptosis-related cysteine
UP in U87	**80219**	COQ10B	1.9833261	0.9265205	3.954036	coenzyme Q10 homolog B (S. cerevisiae)
UP in U87	**7336**	UBE2V2	1.3846528	0.9020037	2.611091	ubiquitin-conjugating enzyme E2 variant 2
UP in U87	**440078**	FAM66C	5.5298209	0.8461518	46.2	family with sequence similarity 66, member
UP in U87	**101059948**	LOC101059948	2.3967588	0.8432628	5.266187	uncharacterized LOC101059948
UP in U87	**6524**	SLC5A2	1.6799756	0.8843672	3.204225	solute carrier family 5 (sodium/glucose cotransporter), member 2

UP in U87	**79791**	FBXO31	1.0481478	0.8552812	2.067873	F-box protein 31
UP in U87	**9645**	MICAL2	3.2807958	0.9708696	9.718919	microtubule associated monooxygenase, calponin and LIM domain containing 2
UP in U87	**8795**	TNFRSF10 B	1.0313913	0.8847405	2.043994	tumor necrosis factor receptor superfamily, member 10b
UP in U87	**90507**	SCRN2	1.5284576	0.905595	2.884773	secernin 2
UP in U87	**6567**	SLC16A2	8.6770149	0.991724	409.3	solute carrier family 16, member 2 (thyroid hormone transporter)
UP in U87	**51149**	C5orf45	3.018502	0.9691095	8.103258	chromosome 5 open reading frame 45
UP in U87	**6318**	SERPINB4	7.0874628	0.8302665	136	serpin peptidase inhibitor, clade B (ovalbumin), member 4
UP in U87	**10082**	GPC6	8.566054	0.9532508	379	glypican 6
UP in U87	**147372**	CCBE1	3.4826639	0.8640994	11.17857	collagen and calcium binding EGF domains 1
UP in U87	**5714**	PSMD8	2.0010888	0.9404057	4.00302	proteasome (prosome, macropain) 26S subunit, non-ATPase, 8
UP in U87	**4125**	MAN2B1	1.3799255	0.8971412	2.602549	mannosidase, alpha, class 2B, member 1
UP in U87	**493869**	GPX8	1.4268147	0.8992568	2.688525	glutathione peroxidase 8 (putative)
UP in U87	**3675**	ITGA3	3.6877772	0.9809145	12.8864	integrin, alpha 3 (antigen CD49C, alpha 3 subunit of VLA-3 receptor)
UP in U87	**64114**	TMBIM1	2.1645772	0.9412946	4.48335	transmembrane BAX inhibitor motif
UP in U87	**1742**	DLG4	1.7118067	0.9139599	3.275708	discs, large homolog 4 (Drosophila)
UP in U87	**10025**	MED16	2.2158991	0.9366633	4.64571	mediator complex subunit 16
UP in U87	**4864**	NPC1	2.6305263	0.9590823	6.192519	Niemann-Pick disease, type C1
UP in U87	**97**	ACYP1	1.0682329	0.8742777	2.096863	acylphosphatase 1, erythrocyte (common)
UP in U87	**5098**	PCDHGC3	3.857981	0.9785766	14.5	protocadherin gamma subfamily C, 3
UP in U87	**619208**	FAM229B	2.6976121	0.9287873	6.487273	family with sequence similarity 229, member
UP in U87	**126014**	OSCAR	4.1941145	0.8175547	18.30435	osteoclast associated, immunoglobulin-like receptor
UP in U87	**6714**	SRC	2.0623117	0.9343609	4.17655	v-src sarcoma (Schmidt-Ruppin A-2) viral oncogene homolog (avian)
UP in U87	**23396**	PIP5K1C	1.4080422	0.8973012	2.653768	phosphatidylinositol-4-phosphate 5-kinase, type I, gamma
UP in U87	**84897**	TBRG1	2.0944564	0.9295518	4.270652	transforming growth factor beta regulator 1
UP in U87	**5210**	PFKFB4	2.3662838	0.9477305	5.156113	6-phosphofructo-2-kinase/fructose-2,6-biphosphatase 4
UP in U87	**755**	C21orf2	1.4385003	0.8687308	2.71039	chromosome 21 open reading frame 2
UP in U87	**8740**	TNFSF14	3.4131259	0.9298185	10.65254	tumor necrosis factor (ligand) superfamily, member 14
UP in U87	**411**	ARSB	1.0487562	0.8582858	2.068746	arylsulfatase B
UP in U87	**220002**	CYB561A	1.349455	0.9009014	2.548158	cytochrome b561 family, member A3
UP in U87	**231**	AKR1B1	3.7066797	0.9825946	13.05635	aldo-keto reductase family 1, member B1 (aldose reductase)
UP in U87	**51062**	ATL1	3.1532173	0.9386723	8.896373	atlastin GTPase 1
UP in U87	**8813**	DPM1	1.1276942	0.892172	2.185092	dolichyl-phosphate mannosyltransferase polypeptide 1, catalytic subunit
UP in U87	**100128076**	LOC10012 8076	4.2941831	0.8141768	19.61905	protein tyrosine phosphatase pseudogene
UP in U87	**54809**	SAMD9	4.6317472	0.9634025	24.79104	sterile alpha motif domain containing 9
UP in U87	**79036**	KXD1	1.5766019	0.9219069	2.982665	KxDL motif containing 1
UP in U87	**642938**	FAM196A	7.5533093	0.9521041	187.8333	family with sequence similarity 196, member
UP in U87	**11160**	ERLIN2	1.3730093	0.8944833	2.590103	ER lipid raft associated 2
UP in U87	**130535**	KCTD18	2.7570007	0.941828	6.759894	potassium channel tetramerisation domain containing 18
UP in U87	**639**	PRDM1	1.8488145	0.8742866	3.602041	PR domain containing 1, with ZNF domain
UP in U87	**7766**	ZNF223	6.112005	0.8298665	69.16667	zinc finger protein 223
UP in U87	**144811**	LACC1	2.8830336	0.9445126	7.376997	laccase (multicopper oxidoreductase) domain containing 1

UP in U87	**55041**	PLEKHB2	1.7495558	0.9258538	3.36255	pleckstrin homology domain containing, family B (evectins) member 2
UP in U87	**654342**	LOC65434	1.1472835	0.8748378	2.214964	lymphocyte-specific protein 1 pseudogene
UP in U87	**1183**	CLCN4	3.9116916	0.9383345	15.05	chloride channel, voltage-sensitive 4
UP in U87	**91869**	RFT1	1.1134249	0.8662418	2.163587	RFT1 homolog (S. cerevisiae)
UP in U87	**25891**	PAMR1	11.837234	0.9976177	3659	peptidase domain containing associated with muscle regeneration 1
UP in U87	**54**	ACP5	7.0717177	0.9957953	134.5238	acid phosphatase 5, tartrate resistant
UP in U87	**403**	ARL3	1.4432517	0.8935321	2.719331	ADP-ribosylation factor-like 3
UP in U87	**4825**	NKX6-1	8.0297473	0.9220225	261.3333	NK6 homeobox 1
UP in U87	**100130776**	LOC100130776	1.2245266	0.8889096	2.336788	uncharacterized LOC100130776
UP in U87	**22871**	NLGN1	6.9068906	0.8074386	120	neuroligin 1
UP in U87	**91663**	MYADM	1.738368	0.9242182	3.336575	myeloid-associated differentiation marker
UP in U87	**9764**	KIAA0513	2.4494991	0.8100876	5.462264	KIAA0513
UP in U87	**2669**	GEM	6.3293001	0.9972532	80.40984	GTP binding protein overexpressed in skeletal muscle
UP in U87	**9638**	FEZ1	6.6030081	0.9812523	97.20833	fasciculation and elongation protein zeta 1 (zygin I)
UP in U87	**29**	ABR	2.0886219	0.9404324	4.253416	active BCR-related
UP in U87	**399687**	MYO18A	1.8217592	0.9225203	3.53512	myosin XVIIIA
UP in U87	**148252**	DIRAS1	6.0276547	0.992204	65.23864	DIRAS family, GTP-binding RAS-like 1
UP in U87	**90627**	STARD13	2.2734666	0.9037904	4.834835	StAR-related lipid transfer (START) domain containing 13
UP in U87	**100505806**	LOC100505806	9.6975468	0.9845414	830.3333	uncharacterized LOC100505806
UP in U87	**9600**	PITPNM1	1.1717696	0.8911764	2.252879	phosphatidylinositol transfer protein, membrane-associated 1
UP in U87	**2086**	ERV3-1	4.5918012	0.9782655	24.11404	endogenous retrovirus group 3, member 1
UP in U87	**155066**	ATP6V0E	1.0149503	0.877789	2.020833	ATPase, H+ transporting V0 subunit e2
UP in U87	**4784**	NFIX	3.7581674	0.9647803	13.53073	nuclear factor I/X (CCAAT-binding transcription factor)
UP in U87	**100129583**	FAM47E	5.5477514	0.8299998	46.77778	family with sequence similarity 47, member
UP in U87	**642517**	AGAP9	1.0495253	0.8269063	2.069849	ArfGAP with GTPase domain, ankyrin repeat and PH domain 9
UP in U87	**558**	AXL	1.769407	0.9253294	3.409138	AXL receptor tyrosine kinase
UP in U87	**2745**	GLRX	3.0455947	0.9687094	8.256868	glutaredoxin (thioltransferase)
UP in U87	**11332**	ACOT7	1.0342829	0.8858517	2.048095	acyl-CoA thioesterase 7
UP in U87	**2034**	EPAS1	2.0371387	0.940219	4.104307	endothelial PAS domain protein 1
UP in U87	**3552**	IL1A	6.7865964	0.8778424	110.4	interleukin 1, alpha
UP in U87	**64130**	LIN7B	2.7986611	0.9205824	6.957944	lin-7 homolog B (C. elegans)
UP in U87	**775**	CACNA1C	7.6110248	0.9546731	195.5	calcium channel, voltage-dependent, L type, alpha 1C subunit
UP in U87	**144110**	TMEM86	5.6808869	0.9849503	51.3	transmembrane protein 86A
UP in U87	**2589**	GALNT1	1.1397156	0.8821626	2.203376	UDP-N-acetyl-alpha-D-galactosamine:polypeptide N-acetylgalactosaminyltransferase 1 (GalNAc-T1)
UP in U87	**126070**	ZNF440	1.578519	0.8410937	2.986631	zinc finger protein 440
UP in U87	**55437**	STRADB	2.4804903	0.9478906	5.580871	STE20-related kinase adaptor beta
UP in U87	**84280**	BTBD10	1.480081	0.8991946	2.789644	BTB (POZ) domain containing 10
UP in U87	**100652759**	PRICKLE2-AS1	8.9725012	0.9683539	502.3333	PRICKLE2 antisense RNA 1
UP in U87	**526**	ATP6V1B2	1.0338655	0.8840294	2.047503	ATPase, H+ transporting, lysosomal 56/58kDa, V1 subunit B2
UP in U87	**157574**	FBXO16	3.2228739	0.907764	9.336449	F-box protein 16

UP in U87	**56288**	PARD3	1.1343449	0.8752911	2.195189	par-3 partitioning defective 3 homolog (C. elegans)
UP in U87	**54550**	NECAB2	5.5774288	0.8140968	47.75	N-terminal EF-hand calcium binding protein
UP in U87	**81502**	HM13	1.249047	0.8995058	2.376844	histocompatibility (minor) 13
UP in U87	**55784**	MCTP2	2.1874955	0.9200402	4.55514	multiple C2 domains, transmembrane 2
UP in U87	**340481**	ZDHHC21	1.803461	0.801305	3.490566	zinc finger, DHHC-type containing 21
UP in U87	**51382**	ATP6V1D	2.0784001	0.9399168	4.223386	ATPase, H+ transporting, lysosomal 34kDa, V1 subunit D
UP in U87	**5578**	PRKCA	1.4567685	0.9030793	2.744928	protein kinase C, alpha
UP in U87	**6281**	S100A10	1.2312794	0.8992924	2.347751	S100 calcium binding protein A10
UP in U87	**132671**	SPATA18	8.9934102	0.9690383	509.6667	spermatogenesis associated 18
UP in U87	**158158**	RASEF	7.4429435	0.8704731	174	RAS and EF-hand domain containing
UP in U87	**1454**	CSNK1E	1.2764186	0.8985813	2.422369	casein kinase 1, epsilon
UP in U87	**23207**	PLEKHM2	1.2452482	0.8963322	2.370593	pleckstrin homology domain containing, family M (with RUN domain) member 2
UP in U87	**8528**	DDO	3.7951802	0.8271019	13.88235	D-aspartate oxidase
UP in U87	**114327**	EFHC1	1.0315425	0.8447651	2.044209	EF-hand domain (C-terminal) containing 1
UP in U87	**64332**	NFKBIZ	4.2981269	0.9876527	19.67275	nuclear factor of kappa light polypeptide gene enhancer in B-cells inhibitor, zeta
UP in U87	**8793**	TNFRSF10 D	2.517829	0.947935	5.727196	tumor necrosis factor receptor superfamily, member 10d, decoy with truncated death domain
UP in U87	**5592**	PRKG1	6.1483918	0.9782033	70.93333	protein kinase, cGMP-dependent, type I
UP in U87	**541565**	C8orf58	1.0810547	0.8528455	2.115582	chromosome 8 open reading frame 58
UP in U87	**728215**	FAM155A	9.0892298	0.9717674	544.6667	family with sequence similarity 155, member
UP in U87	**51129**	ANGPTL4	3.5369254	0.9668338	11.60702	angiopoietin-like 4
UP in U87	**6660**	SOX5	7.5313815	0.8802958	185	SRY (sex determining region Y)-box 5
UP in U87	**2791**	GNG11	2.1450791	0.9420147	4.423165	guanine nucleotide binding protein (G protein), gamma 11
UP in U87	**8111**	GPR68	10.659104	0.9935997	1617	G protein-coupled receptor 68
UP in U87	**3489**	IGFBP6	7.1008645	0.9991644	137.2692	insulin-like growth factor binding protein 6
UP in U87	**79846**	C7orf63	3.0962153	0.8140612	8.551724	chromosome 7 open reading frame 63
UP in U87	**7274**	TTPA	5.1974057	0.8493075	36.69231	tocopherol (alpha) transfer protein
UP in U87	**353322**	ANKRD37	1.9612829	0.8683663	3.894081	ankyrin repeat domain 37
UP in U87	**54674**	LRRN3	7.5089266	0.9593223	182.1429	leucine rich repeat neuronal 3
UP in U87	**64744**	SMAP2	1.3599123	0.8987146	2.566696	small ArfGAP2
UP in U87	**81569**	ACTL8	5.1829303	0.9917773	36.32599	actin-like 8
UP in U87	**3141**	HLCS	1.6132065	0.8917898	3.05931	holocarboxylase synthetase (biotin-(proprionyl-CoA-carboxylase (ATP-hydrolysing)) ligase)
UP in U87	**80320**	SP6	2.5682838	0.9075151	5.931034	Sp6 transcription factor
UP in U87	**84978**	FRMD5	3.3468028	0.8752289	10.17391	FERM domain containing 5
UP in U87	**11149**	BVES	4.8617449	0.9707896	29.07576	blood vessel epicardial substance
UP in U87	**401647**	GOLGA7B	6.0074945	0.9598201	64.33333	golgin A7 family, member B
UP in U87	**50862**	RNF141	1.205174	0.866944	2.305651	ring finger protein 141
UP in U87	**23625**	FAM89B	1.0413063	0.8823937	2.05809	family with sequence similarity 89, member
UP in U87	**2571**	GAD1	1.4105425	0.8217238	2.658371	glutamate decarboxylase 1 (brain, 67kDa)
UP in U87	**22822**	PHLDA1	6.9827599	0.9987022	126.4795	pleckstrin homology-like domain, family A, member 1
UP in U87	**100287314**	LOC10028 7314	3.5969351	0.9669138	12.1	uncharacterized LOC100287314
UP in U87	**2015**	EMR1	6.9228321	0.809172	121.3333	egf-like module containing, mucin-like, hormone receptor-like 1
UP in U87	**9459**	ARHGEF6	9.5557395	0.9910306	752.6	Rac/Cdc42 guanine nucleotide exchange factor (GEF) 6
UP in U87	**143903**	LAYN	8.4463941	0.9800612	348.8333	layilin

UP in U87	**286333**	FAM225A	3.2993098	0.897159	9.844444	family with sequence similarity 225, member A (non-protein coding)
UP in U87	**54622**	ARL15	1.0920606	0.8077942	2.131783	ADP-ribosylation factor-like 15
UP in U87	**146227**	BEAN1	4.6413984	0.9458016	24.95745	brain expressed, associated with NEDD4, 1
UP in U87	**3123**	HLA-DRB1	12.927531	0.9993511	7790.667	major histocompatibility complex, class II, DR beta 1
UP in U87	**196394**	AMN1	1.4863023	0.8084431	2.8017	antagonist of mitotic exit network 1 homolog (S. cerevisiae)
UP in U87	**387856**	C12orf68	8.2573878	0.9365033	306	chromosome 12 open reading frame 68
UP in U87	**100526831**	SLX1B-SULT1A4	6.9541963	0.8137678	124	SLX1B-SULT1A4 readthrough
UP in U87	**4500**	MT1L	4.7861331	0.9934219	27.59114	metallothionein 1L (gene/pseudogene)
UP in U87	**55640**	FLVCR2	1.2739227	0.8121589	2.418182	feline leukemia virus subgroup C cellular receptor family, member 2
UP in U87	**50485**	SMARCAL1	1.6191951	0.9157911	3.072036	SWI/SNF related, matrix associated, actin dependent regulator of chromatin, subfamily a-like 1
UP in U87	**254359**	ZDHHC24	1.2471964	0.8851583	2.373797	zinc finger, DHHC-type containing 24
UP in U87	**7039**	TGFA	7.4157887	0.9956798	170.7556	transforming growth factor, alpha
UP in U87	**94032**	CAMK2N2	3.3077142	0.9638559	9.901961	calcium/calmodulin-dependent protein kinase II inhibitor 2
UP in U87	**22881**	ANKRD6	3.3539569	0.8259641	10.22449	ankyrin repeat domain 6
UP in U87	**92659**	MAFG-	2.4098758	0.8716554	5.314286	MAFG antisense RNA 1 (head to head)
UP in U87	**56006**	SMG9	1.2141465	0.8906519	2.320035	smg-9 homolog, nonsense mediated mRNA decay factor (C. elegans)
UP in U87	**27128**	CYTH4	7.8454901	0.9082884	230	cytohesin 4
UP in U87	**220388**	CCDC89	5.5109619	0.8437517	45.6	coiled-coil domain containing 89
UP in U87	**57804**	POLD4	2.5437863	0.9515619	5.831174	polymerase (DNA-directed), delta 4, accessory subunit
UP in U87	**79587**	CARS2	2.6019877	0.9587267	6.071225	cysteinyl-tRNA synthetase 2, mitochondrial (putative)
UP in U87	**6098**	ROS1	7.550397	0.9792256	187.4545	c-ros oncogene 1 , receptor tyrosine kinase
UP in U87	**2259**	FGF14	5.5681673	0.8324	47.44444	fibroblast growth factor 14
UP in U87	**284352**	PPP1R37	1.6983898	0.9176934	3.245385	protein phosphatase 1, regulatory subunit 37
UP in U87	**114803**	MYSM1	1.4755847	0.8777801	2.780963	Myb-like, SWIRM and MPN domains 1
UP in U87	**22879**	MON1B	1.3703784	0.8970256	2.585384	MON1 homolog B (yeast)
UP in U87	**5874**	RAB27B	5.3740427	0.9939463	41.47134	RAB27B, member RAS oncogene family
UP in U87	**441024**	MTHFD2L	2.2171592	0.8666684	4.64977	methylenetetrahydrofolate dehydrogenase (NADP+ dependent) 2-like
UP in U87	**2191**	FAP	4.2883755	0.984897	19.54023	fibroblast activation protein, alpha
UP in U87	**56126**	PCDHB10	7.8392038	0.9077106	229	protocadherin beta 10
UP in U87	**28996**	HIPK2	2.2909926	0.9407168	4.893927	homeodomain interacting protein kinase 2
UP in U87	**5066**	PAM	2.8543715	0.9638647	7.231884	peptidylglycine alpha-amidating
UP in U87	**7042**	TGFB2	2.9253612	0.9357744	7.596639	transforming growth factor, beta 2
UP in U87	**81618**	ITM2C	2.1185327	0.9406902	4.34252	integral membrane protein 2C
UP in U87	**813**	CALU	2.5183805	0.9532242	5.729386	calumenin
UP in U87	**6810**	STX4	1.5659033	0.9070084	2.960628	syntaxin 4
UP in U87	**1820**	ARID3A	3.5240265	0.9443881	11.5037	AT rich interactive domain 3A (BRIGHT-
UP in U87	**80210**	ARMC9	2.6361917	0.9561577	6.216884	armadillo repeat containing 9
UP in U87	**257236**	CCDC96	3.5781412	0.8686419	11.9434	coiled-coil domain containing 96
UP in U87	**8120**	AP3B2	6.1404812	0.9670649	70.54545	adaptor-related protein complex 3, beta 2
UP in U87	**8764**	TNFRSF14	2.653367	0.8659573	6.291339	tumor necrosis factor receptor superfamily, member 14
UP in U87	**285966**	FAM115C	3.9016108	0.9295162	14.94521	family with sequence similarity 115, member
UP in U87	**1809**	DPYSL3	3.7019133	0.97991	13.01329	dihydropyrimidinase-like 3
UP in U87	**9848**	MFAP3L	4.7158934	0.8925276	26.28	microfibrillar-associated protein 3-like
UP in U87	**1400**	CRMP1	2.4035997	0.9390367	5.291217	collapsin response mediator protein 1

UP in U87	**375057**	C1orf95	9.027906	0.9700428	522	chromosome 1 open reading frame 95
UP in U87	**150771**	ITPRIPL1	8.1172102	0.928005	277.6667	inositol 1,4,5-trisphosphate receptor interacting protein-like 1
UP in U87	**2055**	CLN8	1.1943919	0.8506587	2.288483	ceroid-lipofuscinosis, neuronal 8 (epilepsy, progressive with mental retardation)
UP in U87	**100505695**	LOC100505695	7.7879026	0.9033993	221	uncharacterized LOC100505695
UP in U87	**375298**	CERKL	3.4757334	0.8099988	11.125	ceramide kinase-like
UP in U87	**10971**	YWHAQ	1.3927522	0.9052305	2.625791	tyrosine 3-monooxygenase/tryptophan 5-monooxygenase activation protein, theta polypeptide
UP in U87	**26191**	PTPN22	7.6424128	0.9431703	199.8	protein tyrosine phosphatase, non-receptor type 22 (lymphoid)
UP in U87	**140885**	SIRPA	3.6369932	0.960389	12.44068	signal-regulatory protein alpha
UP in U87	**341880**	SLC35F4	7.7725895	0.9023859	218.6667	solute carrier family 35, member F4
UP in U87	**440456**	PLEKHM1P	1.6966601	0.9045105	3.241497	pleckstrin homology domain containing, family M (with RUN domain) member 1 pseudogene
UP in U87	**1437**	CSF2	10.504819	0.9925418	1453	colony stimulating factor 2 (granulocyte-macrophage)
UP in U87	**57718**	PPP4R4	7.7600162	0.9864704	216.7692	protein phosphatase 4, regulatory subunit 4
UP in U87	**116412**	ZNF837	5.9441891	0.8356535	61.57143	zinc finger protein 837
UP in U87	**11190**	CEP250	1.468405	0.8999413	2.767158	centrosomal protein 250kDa
UP in U87	**100506696**	KDM5B-AS1	6.8744691	0.8024695	117.3333	KDM5B antisense RNA 1 (head to head)
UP in U87	**23327**	NEDD4L	3.0470436	0.9616335	8.265165	neural precursor cell expressed, developmentally down-regulated 4-like, E3 ubiquitin protein ligase
UP in U87	**6016**	RIT1	1.0544104	0.8591303	2.076869	Ras-like without CAAX 1
UP in U87	**26999**	CYFIP2	2.3089987	0.9242182	4.95539	cytoplasmic FMR1 interacting protein 2
UP in U87	**9755**	TBKBP1	4.7769462	0.9830213	27.416	TBK1 binding protein 1
UP in U87	**9781**	RNF144A	1.0871107	0.8421338	2.124481	ring finger protein 144A
UP in U87	**145482**	PTGR2	1.3018509	0.8545256	2.46545	prostaglandin reductase 2
UP in U87	**51315**	KRCC1	7.4613976	0.9919818	176.24	lysine-rich coiled-coil 1
UP in U87	**100134259**	LOC100134259	3.5049092	0.915231	11.35227	uncharacterized LOC100134259
UP in U87	**9896**	FIG4	2.5773933	0.9484861	5.968603	FIG4 homolog, SAC1 lipid phosphatase domain containing (S. cerevisiae)
UP in U87	**29948**	OSGIN1	4.1131537	0.97919	17.30544	oxidative stress induced growth inhibitor 1
UP in U87	**55825**	PECR	1.3165947	0.861077	2.490775	peroxisomal trans-2-enoyl-CoA reductase
UP in U87	**9015**	TAF1A	1.0160981	0.8421694	2.022442	TATA box binding protein (TBP)-associated factor, RNA polymerase I, A, 48kDa
UP in U87	**9592**	IER2	1.0551257	0.8847227	2.077899	immediate early response 2
UP in U87	**10974**	ADIRF	2.0811629	0.8889896	4.231481	adipogenesis regulatory factor
UP in U87	**65975**	STK33	1.2964135	0.8177769	2.456175	serine/threonine kinase 33
UP in U87	**30011**	SH3KBP1	2.5602446	0.9498018	5.898077	SH3-domain kinase binding protein 1
UP in U87	**387119**	CEP85L	3.6109577	0.8773446	12.21818	centrosomal protein 85kDa-like
UP in U87	**497661**	C18orf32	1.3573322	0.8970523	2.56211	chromosome 18 open reading frame 32
UP in U87	**3576**	IL8	13.686097	1	13180.32	interleukin 8
UP in U87	**8061**	FOSL1	6.1204621	0.9976977	69.57331	FOS-like antigen 1
UP in U87	**624**	BDKRB2	6.030273	0.9838125	65.35714	bradykinin receptor B2
UP in U87	**50515**	CHST11	2.3640006	0.9362188	5.147959	carbohydrate (chondroitin 4) sulfotransferase
UP in U87	**123920**	CMTM3	1.486765	0.9022703	2.802598	CKLF-like MARVEL transmembrane domain containing 3
UP in U87	**51228**	GLTP	1.4252981	0.9022526	2.6857	glycolipid transfer protein
UP in U87	**4331**	MNAT1	1.2853775	0.8936654	2.437458	menage a trois homolog 1, cyclin H assembly factor (Xenopus laevis)

UP in U87	**25822**	DNAJB5	2.7971082	0.9558554	6.950459	DnaJ (Hsp40) homolog, subfamily B,
UP in U87	**5817**	PVR	1.0301374	0.8817181	2.042219	poliovirus receptor
UP in U87	**23125**	CAMTA2	1.9354629	0.9262183	3.825008	calmodulin binding transcription activator 2
UP in U87	**285527**	FRYL	1.1119111	0.8569346	2.161318	FRY-like
UP in U87	**51692**	CPSF3	1.3396702	0.8996213	2.530935	cleavage and polyadenylation specific factor 3, 73kDa
UP in U87	**80149**	ZC3H12A	3.5160947	0.9763365	11.44063	zinc finger CCCH-type containing 12A
UP in U87	**23118**	TAB2	1.2412494	0.8957544	2.364032	TGF-beta activated kinase 1/MAP3K7 binding protein 2
UP in U87	**124583**	CANT1	1.476193	0.9040304	2.782136	calcium activated nucleotidase 1
UP in U87	**122786**	FRMD6	1.9895583	0.9114086	3.971154	FERM domain containing 6
UP in U87	**7376**	NR1H2	1.4635146	0.9054261	2.757794	nuclear receptor subfamily 1, group H,
UP in U87	**23161**	SNX13	1.3265995	0.882047	2.508108	sorting nexin 13
UP in U87	**79083**	MLPH	9.7479955	0.9985333	859.8823	melanophilin
UP in U87	**1230**	CCR1	7.6138918	0.9741853	195.8889	chemokine (C-C motif) receptor 1
UP in U87	**5865**	RAB3B	6.346487	0.9937863	81.37349	RAB3B, member RAS oncogene family
UP in U87	**84265**	POLR3GL	1.7229896	0.9174889	3.301198	polymerase (RNA) III (DNA directed) polypeptide G (32kD)-like
UP in U87	**94027**	CGB7	7.7369656	0.8993102	213.3333	chorionic gonadotropin, beta polypeptide 7
UP in U87	**7802**	DNALI1	7.1788032	0.9603001	144.8889	dynein, axonemal, light intermediate chain 1
UP in U87	**3269**	HRH1	2.200601	0.9171867	4.596708	histamine receptor H1
UP in U87	**2632**	GBE1	1.2930545	0.8973012	2.450463	glucan (1,4-alpha-), branching enzyme 1
UP in U87	**22924**	MAPRE3	1.8541053	0.919249	3.615275	microtubule-associated protein, RP/EB family, member 3
UP in U87	**26022**	TMEM98	9.4239038	0.994453	686.875	transmembrane protein 98
UP in U87	**2073**	ERCC5	1.5004063	0.9003947	2.829224	excision repair cross-complementing rodent repair deficiency, complementation group 5
UP in U87	**2290**	FOXG1	9.4262648	0.9798034	688	forkhead box G1
UP in U87	**93664**	CADPS2	7.1111357	0.879158	138.25	Ca++-dependent secretion activator 2
UP in U87	**57466**	SCAF4	1.1482526	0.8753711	2.216453	SR-related CTD-associated factor 4
UP in U87	**2118**	ETV4	2.1644737	0.9280673	4.483029	ets variant 4
UP in U87	**23630**	KCNE1L	3.9805476	0.8203904	15.78571	KCNE1-like
UP in U87	**415**	ARSE	2.6335142	0.8483208	6.205357	arylsulfatase E (chondrodysplasia punctata 1)
UP in U87	**1292**	COL6A2	10.25726	0.9999556	1223.892	collagen, type VI, alpha 2
UP in U87	**3987**	LIMS1	1.5866924	0.920378	3.003599	LIM and senescent cell antigen-like domains
UP in U87	**1356**	CP	1.9355364	0.8399559	3.825203	ceruloplasmin (ferroxidase)
UP in U87	**5209**	PFKFB3	1.483396	0.8997458	2.796061	6-phosphofructo-2-kinase/fructose-2,6-biphosphatase 3
UP in U87	**221264**	AKD1	2.5263348	0.8299465	5.761062	adenylate kinase domain containing 1
UP in U87	**60685**	ZFAND3	1.6886343	0.9152755	3.223514	zinc finger, AN1-type domain 3
UP in U87	**5708**	PSMD2	1.2529003	0.8997547	2.3832	proteasome (prosome, macropain) 26S subunit, non-ATPase, 2
UP in U87	**79644**	SRD5A3	1.367286	0.8331378	2.579848	steroid 5 alpha-reductase 3
UP in U87	**874**	CBR3	1.262522	0.8791313	2.399148	carbonyl reductase 3
UP in U87	**162989**	DEDD2	1.4871683	0.9028393	2.803382	death effector domain containing 2
UP in U87	**23505**	TMEM131	1.0005931	0.8716909	2.000822	transmembrane protein 131
UP in U87	**550112**	LOC55011	1.3115643	0.8138834	2.482105	uncharacterized LOC550112
UP in U87	**1026**	CDKN1A	4.1982642	0.9885061	18.35707	cyclin-dependent kinase inhibitor 1A (p21,
UP in U87	**4674**	NAP1L2	5.650254	0.8432716	50.22222	nucleosome assembly protein 1-like 2
UP in U87	**10957**	PNRC1	1.0177943	0.8846072	2.024821	proline-rich nuclear receptor coactivator 1
UP in U87	**64780**	MICAL1	2.2008742	0.9366011	4.597578	microtubule associated monooxygenase, calponin and LIM domain containing 1
UP in U87	**4092**	SMAD7	1.652764	0.9103508	3.144355	SMAD family member 7
UP in U87	**3337**	DNAJB1	1.148479	0.8940832	2.216801	DnaJ (Hsp40) homolog, subfamily B,
UP in U87	**56907**	SPIRE1	1.7065313	0.9191868	3.263752	spire homolog 1 (Drosophila)
UP in U87	**9253**	NUMBL	1.3103544	0.885585	2.480025	numb homolog (Drosophila)-like
UP in U87	**11057**	ABHD2	2.3677867	0.950264	5.161487	abhydrolase domain containing 2

UP in U87	**112752**	IFT43	2.1648194	0.939099	4.484103	intraflagellar transport 43 homolog (Chlamydomonas)
UP in U87	**10282**	BET1	1.1191581	0.8805714	2.172202	Bet1 golgi vesicular membrane trafficking
UP in U87	**23786**	BCL2L13	1.3790462	0.8958789	2.600964	BCL2-like 13 (apoptosis facilitator)
UP in U87	**84557**	MAP1LC3 A	4.132736	0.9840614	17.54194	microtubule-associated protein 1 light chain 3 alpha
UP in U87	**100874392**	ANKRD20 A12P	2.5392937	0.9351965	5.813043	ankyrin repeat domain 20 family, member A12, pseudogene
UP in U87	**5654**	HTRA1	2.8598154	0.9652159	7.259224	HtrA serine peptidase 1
UP in U87	**6990**	DYNLT3	1.5548452	0.9066972	2.938022	dynein, light chain, Tctex-type 3
UP in U87	**64121**	RRAGC	1.1716558	0.8899319	2.252701	Ras-related GTP binding C
UP in U87	**6299**	SALL1	11.29959	0.996222	2520.667	sal-like 1 (Drosophila)
UP in U87	**54329**	GPR85	7.4676056	0.9111864	177	G protein-coupled receptor 85
UP in U87	**55907**	CMAS	2.0088702	0.939499	4.024669	cytidine monophosphate N-acetylneuraminic acid synthetase
UP in U87	**56978**	PRDM8	9.1463565	0.9732252	566.6667	PR domain containing 8
UP in U87	**8828**	NRP2	4.1595788	0.9870749	17.87138	neuropilin 2
UP in U87	**5025**	P2RX4	2.8736611	0.9600512	7.329227	purinergic receptor P2X, ligand-gated ion channel, 4
UP in U87	**83861**	RSPH3	2.030102	0.9012481	4.084337	radial spoke 3 homolog (Chlamydomonas)
UP in U87	**50640**	PNPLA8	1.4239341	0.892172	2.683162	patatin-like phospholipase domain containing
UP in U87	**29844**	TFPT	1.4299785	0.8929276	2.694427	TCF3 (E2A) fusion partner (in childhood Leukemia)
UP in U87	**196463**	PLBD2	2.1754633	0.9410102	4.517308	phospholipase B domain containing 2
UP in U87	**4942**	OAT	1.1382493	0.8917898	2.201138	ornithine aminotransferase
UP in U87	**114784**	CSMD2	10.57963	0.9930041	1530.333	CUB and Sushi multiple domains 2
UP in U87	**642946**	FLVCR1-AS1	1.0016903	0.8189415	2.002345	FLVCR1 antisense RNA 1 (head to head)
UP in U87	**57631**	LRCH2	7.1272206	0.9088485	139.8	leucine-rich repeats and calponin homology (CH) domain containing 2
UP in U87	**255783**	PRR24	1.5797127	0.9066706	2.989103	proline rich 24
UP in U87	**283316**	CD163L1	9.0052737	0.9918485	513.875	CD163 molecule-like 1
UP in U87	**6273**	S100A2	1.3949278	0.8725888	2.629754	S100 calcium binding protein A2
UP in U87	**57654**	UVSSA	2.0967248	0.9171334	4.277372	UV-stimulated scaffold protein A
UP in U87	**51604**	PIGT	1.0038263	0.8863406	2.005311	phosphatidylinositol glycan anchor biosynthesis, class T
UP in U87	**285533**	RNF175	8.3851434	0.9438903	334.3333	ring finger protein 175
UP in U87	**3091**	HIF1A	2.3578836	0.9518908	5.126178	hypoxia inducible factor 1, alpha subunit (basic helix-loop-helix transcription factor)
UP in U87	**161742**	SPRED1	1.4284241	0.8913009	2.691525	sprouty-related, EVH1 domain containing 1
UP in U87	**79786**	KLHL36	2.2213667	0.9246715	4.66335	kelch-like family member 36
UP in U87	**6251**	RSU1	1.4339175	0.9024126	2.701794	Ras suppressor protein 1
UP in U87	**6999**	TDO2	6.7681843	0.9309385	109	tryptophan 2,3-dioxygenase
UP in U87	**116151**	FAM210B	1.7213802	0.9201824	3.297517	family with sequence similarity 210, member
UP in U87	**768**	CA9	2.2044585	0.9387523	4.609015	carbonic anhydrase IX
UP in U87	**4803**	NGF	7.073249	0.8291198	134.6667	nerve growth factor (beta polypeptide)
UP in U87	**11096**	ADAMTS 5	3.0151069	0.9487439	8.084211	ADAM metallopeptidase with thrombospondin type 1 motif, 5
UP in U87	**151887**	CCDC80	2.544551	0.9505929	5.834265	coiled-coil domain containing 80
UP in U87	**7368**	UGT8	1.2744935	0.8684463	2.419139	UDP glycosyltransferase 8
UP in U87	**376267**	RAB15	2.6927277	0.8410315	6.465347	RAB15, member RAS oncogene family
UP in U87	**7138**	TNNT1	3.4570633	0.9779188	10.98196	troponin T type 1 (skeletal, slow)
UP in U87	**23031**	MAST3	1.0728161	0.8240706	2.103535	microtubule associated serine/threonine
UP in U87	**401022**	HOXD-	7.6235157	0.9422103	197.2	HOXD cluster antisense RNA 1
UP in U87	**6696**	SPP1	11.482808	0.9966932	2862	secreted phosphoprotein 1
UP in U87	**84223**	IQCG	3.4643675	0.9725941	11.0377	IQ motif containing G
UP in U87	**132884**	EVC2	7.8621207	0.909773	232.6667	Ellis van Creveld syndrome 2

UP in U87	**22904**	SBNO2	1.008758	0.8825537	2.012178	strawberry notch homolog 2 (Drosophila)
UP in U87	**338785**	KRT79	7.9265078	0.9728341	243.2857	keratin 79
UP in U87	**23474**	ETHE1	2.0300612	0.9392234	4.084222	ethylmalonic encephalopathy 1
UP in U87	**55509**	BATF3	2.3015363	0.9308763	4.929825	basic leucine zipper transcription factor, ATF-like 3
UP in U87	**51291**	GMIP	1.7332211	0.8992835	3.324693	GEM interacting protein
UP in U87	**2123**	EVI2A	6.0945176	0.8914431	68.33333	ecotropic viral integration site 2A
UP in U87	**79018**	GID4	1.4382562	0.8812114	2.709931	GID complex subunit 4, VID24 homolog (S. cerevisiae)
UP in U87	**6756**	SSX1	7.785289	0.9811457	220.6	synovial sarcoma, X breakpoint 1
UP in U87	**10673**	TNFSF13B	5.0552824	0.8651484	33.25	tumor necrosis factor (ligand) superfamily, member 13b
UP in U87	**10644**	IGF2BP2	2.1744446	0.9347965	4.51412	insulin-like growth factor 2 mRNA binding protein 2
UP in U87	**149775**	GNAS-	4.2051144	0.8449695	18.44444	GNAS antisense RNA 1
UP in U87	**84830**	ADTRP	4.9068906	0.9555887	30	androgen-dependent TFPI-regulating protein
UP in U87	**51409**	HEMK1	1.1547485	0.8500009	2.226455	HemK methyltransferase family member 1
UP in U87	**115557**	ARHGEF2	3.2343546	0.93709	9.411043	Rho guanine nucleotide exchange factor
UP in U87	**8309**	ACOX2	7.5974319	0.8870784	193.6667	acyl-CoA oxidase 2, branched chain
UP in U87	**6781**	STC1	5.7903529	0.9970576	55.34392	stanniocalcin 1
UP in U87	**6451**	SH3BGRL	1.1168672	0.8880029	2.168755	SH3 domain binding glutamic acid-rich protein like
UP in U87	**51084**	CRYL1	2.4804764	0.9383078	5.580817	crystallin, lambda 1
UP in U87	**4794**	NFKBIE	2.1572444	0.9089285	4.460621	nuclear factor of kappa light polypeptide gene enhancer in B-cells inhibitor, epsilon
UP in U87	**728392**	LOC72839	5.0957099	0.9864882	34.19492	uncharacterized LOC728392
UP in U87	**64506**	CPEB1	6.2124343	0.9939552	74.15306	cytoplasmic polyadenylation element binding protein 1
UP in U87	**283487**	LINC0034	2.0470413	0.869033	4.132576	long intergenic non-protein coding RNA 346
UP in U87	**950**	SCARB2	1.2548163	0.8963056	2.386368	scavenger receptor class B, member 2
UP in U87	**120227**	CYP2R1	1.6271198	0.8873362	3.088957	cytochrome P450, family 2, subfamily R, polypeptide 1
UP in U87	**80254**	CEP63	1.1284321	0.8787313	2.18621	centrosomal protein 63kDa
UP in U87	**256987**	SERINC5	2.6061247	0.9442015	6.08866	serine incorporator 5
UP in U87	**4756**	NEO1	2.3226283	0.9242182	5.002427	neogenin 1
UP in U87	**4855**	NOTCH4	5.8008999	0.930423	55.75	notch 4
UP in U87	**55879**	GABRQ	2.5226141	0.9236582	5.746224	gamma-aminobutyric acid (GABA) A receptor, theta
UP in U87	**51255**	RNF181	1.166626	0.8955322	2.244861	ring finger protein 181
UP in U87	**3201**	HOXA4	3.6888331	0.8664284	12.89583	homeobox A4
UP in U87	**3621**	ING1	1.0758775	0.8458584	2.108004	inhibitor of growth family, member 1
UP in U87	**4257**	MGST1	1.4906215	0.9082529	2.8101	microsomal glutathione S-transferase 1
UP in U87	**55190**	NUDT11	3.1733316	0.8919765	9.021277	nudix (nucleoside diphosphate linked moiety X)-type motif 11
UP in U87	**25907**	TMEM158	5.240495	0.9954131	37.80473	transmembrane protein 158
UP in U87	**83894**	TTC29	7.1665816	0.8397603	143.6667	tetratricopeptide repeat domain 29
UP in U87	**730094**	C16orf52	1.1890047	0.8492186	2.279954	chromosome 16 open reading frame 52
UP in U87	**55680**	RUFY2	1.2232929	0.8684641	2.33479	RUN and FYVE domain containing 2
UP in U87	**10505**	SEMA4F	3.0049508	0.9562999	8.0275	sema domain, immunoglobulin domain (Ig), transmembrane domain (TM) and short cytoplasmic domain, (semaphorin) 4F
UP in U87	**100506343**	LOC10050 6343	5.1626202	0.8169502	35.81818	uncharacterized LOC100506343
UP in U87	**10379**	IRF9	1.8853468	0.9155777	3.694417	interferon regulatory factor 9
UP in U87	**664**	BNIP3	1.4620337	0.9069373	2.754964	BCL2/adenovirus E1B 19kDa interacting
UP in U87	**3437**	IFIT3	2.1395227	0.8996391	4.406162	interferon-induced protein with tetratricopeptide repeats 3

UP in U87	**353355**	ZNF233	5.2439256	0.9067239	37.89474	zinc finger protein 233
UP in U87	**7539**	ZFP37	1.81483	0.8099365	3.518182	ZFP37 zinc finger protein
UP in U87	**2357**	FPR1	10.310613	0.991244	1270	formyl peptide receptor 1
UP in U87	**1519**	CTSO	2.7776076	0.8638327	6.857143	cathepsin O
UP in U87	**84892**	GTDC2	1.0374	0.859228	2.052525	glycosyltransferase-like domain containing 2
UP in U87	**10865**	ARID5A	2.6236698	0.9025726	6.163158	AT rich interactive domain 5A (MRF1-like)
UP in U87	**54101**	RIPK4	4.5400366	0.9479528	23.26415	receptor-interacting serine-threonine kinase 4
UP in U87	**55201**	MAP1S	1.7357087	0.9200846	3.330431	microtubule-associated protein 1S
UP in U87	**80017**	C14orf159	4.0798808	0.9756343	16.91089	chromosome 14 open reading frame 159
UP in U87	**9610**	RIN1	1.5495417	0.9044838	2.927241	Ras and Rab interactor 1
UP in U87	**5824**	PEX19	1.7288272	0.9220403	3.314583	peroxisomal biogenesis factor 19
UP in U87	**55279**	ZNF654	1.8908478	0.8830249	3.708531	zinc finger protein 654
UP in U87	**112483**	SAT2	1.765256	0.9243337	3.399343	spermidine/spermine N1-acetyltransferase family member 2
UP in U87	**16**	AARS	1.0827102	0.888963	2.118011	alanyl-tRNA synthetase
UP in U87	**440400**	RNASEK	1.0587508	0.8897808	2.083127	ribonuclease, RNase K
UP in U87	**5360**	PLTP	5.6268818	0.9965331	49.41516	phospholipid transfer protein
UP in U87	**145389**	SLC38A6	2.6431272	0.9528775	6.246843	solute carrier family 38, member 6
UP in U87	**1306**	COL15A1	4.2605276	0.922547	19.16667	collagen, type XV, alpha 1
UP in U87	**55081**	IFT57	3.0304554	0.9647537	8.170676	intraflagellar transport 57 homolog (Chlamydomonas)
UP in U87	**94241**	TP53INP1	3.5630011	0.9553043	11.81871	tumor protein p53 inducible nuclear protein 1
UP in U87	**58160**	NFE4	6.9924663	0.8180259	127.3333	transcription factor NF-E4
UP in U87	**4724**	NDUFS4	1.0007097	0.884056	2.000984	NADH dehydrogenase (ubiquinone) Fe-S protein 4, 18kDa (NADH-coenzyme Q reductase)
UP in U87	**92104**	TTC30A	2.8782384	0.8993369	7.352518	tetratricopeptide repeat domain 30A
UP in U87	**29126**	CD274	6.5106743	0.9419791	91.18182	CD274 molecule
UP in U87	**26960**	NBEA	2.5864552	0.8853717	6.006211	neurobeachin
UP in U87	**3363**	HTR7	9.5018372	0.9812523	725	5-hydroxytryptamine (serotonin) receptor 7, adenylate cyclase-coupled
UP in U87	**50944**	SHANK1	8.6122542	0.9551887	391.3333	SH3 and multiple ankyrin repeat domains 1
UP in U87	**5168**	ENPP2	6.3577164	0.9949242	82.00935	ectonucleotide pyrophosphatase/phosphodiesterase 2
UP in U87	**8638**	OASL	3.7217766	0.9688695	13.19369	2'-5'-oligoadenylate synthetase-like
UP in U87	**25829**	TMEM184	1.5666465	0.9075062	2.962154	transmembrane protein 184B
UP in U87	**112476**	PRRT2	1.2397465	0.8039184	2.36157	proline-rich transmembrane protein 2
UP in U87	**51343**	FZR1	1.7805646	0.9198179	3.435606	fizzy/cell division cycle 20 related 1
UP in U87	**9915**	ARNT2	9.5857137	0.9912973	768.4	aryl-hydrocarbon receptor nuclear
UP in U87	**196403**	DTX3	3.1391081	0.9616602	8.809793	deltex homolog 3 (Drosophila)
UP in U87	**5738**	PTGFRN	1.4742336	0.9024748	2.77836	prostaglandin F2 receptor inhibitor
UP in U87	**84307**	ZNF397	1.1589895	0.8411649	2.23301	zinc finger protein 397
UP in U87	**127281**	FAM213B	1.6566512	0.9147599	3.152838	family with sequence similarity 213, member
UP in U87	**9777**	TM9SF4	1.1300625	0.889923	2.188682	transmembrane 9 superfamily protein
UP in U87	**7079**	TIMP4	3.2848581	0.9665582	9.746324	TIMP metallopeptidase inhibitor 4
UP in U87	**64839**	FBXL17	2.0043841	0.9148043	4.012174	F-box and leucine-rich repeat protein 17
UP in U87	**53827**	FXYD5	3.3131821	0.9731186	9.939561	FXYD domain containing ion transport regulator 5
UP in U87	**57552**	NCEH1	1.1206796	0.8880296	2.174494	neutral cholesterol ester hydrolase 1
UP in U87	**84107**	ZIC4	6.8907709	0.8056252	118.6667	Zic family member 4
UP in U87	**64802**	NMNAT1	1.0530807	0.8474763	2.074956	nicotinamide nucleotide adenylyltransferase
UP in U87	**153129**	SLC38A9	1.0617076	0.8313066	2.087401	solute carrier family 38, member 9
UP in U87	**5732**	PTGER2	6.5628295	0.9559265	94.53846	prostaglandin E receptor 2 (subtype EP2),
UP in U87	**11344**	TWF2	1.5666014	0.9086707	2.962061	twinfilin, actin-binding protein, homolog 2 (Drosophila)
UP in U87	**100499489**	LOC100499489	5.3434078	0.8229061	40.6	uncharacterized LOC100499489

UP in U87	**90993**	CREB3L1	6.0347136	0.9946397	65.55862	cAMP responsive element binding protein 3-like 1
UP in U87	**1846**	DUSP4	1.8569623	0.9186179	3.622441	dual specificity phosphatase 4
UP in U87	**9064**	MAP3K6	1.1410026	0.8759845	2.205342	mitogen-activated protein kinase kinase
UP in U87	**133**	ADM	5.8663034	0.9960265	58.33555	adrenomedullin
UP in U87	**9371**	KIF3B	1.2188054	0.8936121	2.327539	kinesin family member 3B
UP in U87	**2151**	F2RL2	6.4789718	0.8436361	89.2	coagulation factor II (thrombin) receptor-like
UP in U87	**618**	BCYRN1	2.4857012	0.9528775	5.601065	brain cytoplasmic RNA 1
UP in U87	**56650**	CLDND1	2.5282686	0.9522552	5.768789	claudin domain containing 1
UP in U87	**83636**	C19orf12	1.439965	0.8752289	2.713143	chromosome 19 open reading frame 12
UP in U87	**8674**	VAMP4	1.9841231	0.890812	3.956221	vesicle-associated membrane protein 4
UP in U87	**51776**	ZAK	2.0486997	0.9391523	4.137329	sterile alpha motif and leucine zipper containing kinase AZK
UP in U87	**90557**	CCDC74A	3.8511366	0.9784255	14.43137	coiled-coil domain containing 74A
UP in U87	**2067**	ERCC1	1.9502939	0.9278273	3.864533	excision repair cross-complementing rodent repair deficiency, complementation group 1 (includes overlapping antisense sequence)
UP in U87	**23233**	EXOC6B	1.8641794	0.9125998	3.640608	exocyst complex component 6B
UP in U87	**79884**	MAP9	8.6712462	0.9577577	407.6667	microtubule-associated protein 9
UP in U87	**56849**	TCEAL7	12.109613	0.9982132	4419.333	transcription elongation factor A (SII)-like 7
UP in U87	**83538**	TTC25	3.9475326	0.9218803	15.42857	tetratricopeptide repeat domain 25
UP in U87	**22870**	PPP6R1	1.3167137	0.9011858	2.49098	protein phosphatase 6, regulatory subunit 1
UP in U87	**100131213**	ZNF503-AS2	1.2384729	0.8405159	2.359486	ZNF503 antisense RNA 2
UP in U87	**25771**	TBC1D22	1.1185145	0.8706775	2.171233	TBC1 domain family, member 22A
UP in U87	**23169**	SLC35D1	1.1462737	0.8706153	2.213415	solute carrier family 35 (UDP-glucuronic acid/UDP-N-acetylgalactosamine dual transporter), member D1
UP in U87	**7278**	TUBA3C	7.1223966	0.8338667	139.3333	tubulin, alpha 3c
UP in U87	**3036**	HAS1	13.800428	0.9998133	14267.33	hyaluronan synthase 1
UP in U87	**8325**	FZD8	5.2003732	0.9869682	36.76786	frizzled family receptor 8
UP in U87	**22927**	HABP4	3.5624911	0.9711096	11.81454	hyaluronan binding protein 4
UP in U87	**100505622**	LOC100505622	7.523562	0.8794158	184	uncharacterized LOC100505622
UP in U87	**23072**	HECW1	2.3179336	0.8842694	4.986175	HECT, C2 and WW domain containing E3 ubiquitin protein ligase 1
UP in U87	**3676**	ITGA4	6.212699	0.8421783	74.16667	integrin, alpha 4 (antigen CD49D, alpha 4 subunit of VLA-4 receptor)
UP in U87	**7533**	YWHAH	1.1363178	0.8933632	2.198193	tyrosine 3-monooxygenase/tryptophan 5-monooxygenase activation protein, eta polypeptide
UP in U87	**366**	AQP9	9.83289	0.9864971	912	aquaporin 9
UP in U87	**5621**	PRNP	1.2105484	0.8946077	2.314256	prion protein
UP in U87	**10950**	BTG3	1.5344721	0.9072839	2.896824	BTG family, member 3
UP in U87	**6196**	RPS6KA2	4.1976762	0.969545	18.34959	ribosomal protein S6 kinase, 90kDa,
UP in U87	**221322**	C6orf170	2.1747744	0.8302754	4.515152	chromosome 6 open reading frame 170
UP in U87	**57228**	SMAGP	2.7177072	0.9547976	6.578265	small cell adhesion glycoprotein
UP in U87	**645369**	TMEM200	5.3731068	0.9464771	41.44444	transmembrane protein 200C
UP in U87	**7422**	VEGFA	3.2150381	0.9729674	9.285877	vascular endothelial growth factor A
UP in U87	**131544**	CRYBG3	1.3251909	0.8604992	2.50566	beta-gamma crystallin domain containing 3
UP in U87	**114827**	FHAD1	4.8965487	0.9653759	29.78571	forkhead-associated (FHA) phosphopeptide binding domain 1
UP in U87	**1601**	DAB2	1.7770918	0.9199157	3.427346	Dab, mitogen-responsive phosphoprotein, homolog 2 (Drosophila)
UP in U87	**204010**	RPSAP52	8.9935817	0.9945153	509.7273	ribosomal protein SA pseudogene 52
UP in U87	**23303**	KIF13B	3.1891292	0.9621935	9.120603	kinesin family member 13B
UP in U87	**204801**	NLRP11	5.6510517	0.8225772	50.25	NLR family, pyrin domain containing 11

UP in U87	**961**	CD47	2.2071475	0.9380234	4.617614	CD47 molecule
UP in U87	**5365**	PLXNB3	2.8304494	0.9418102	7.112957	plexin B3
UP in U87	**477**	ATP1A2	6.2854022	0.9281117	78	ATPase, Na+/K+ transporting, alpha 2 polypeptide
UP in U87	**728855**	LINC0062	2.3056322	0.936041	4.943841	long intergenic non-protein coding RNA 623
UP in U87	**3726**	JUNB	1.5116722	0.9051238	2.851404	jun B proto-oncogene
UP in U87	**27075**	TSPAN13	2.7544072	0.9575622	6.747753	tetraspanin 13
UP in U87	**2944**	GSTM1	8.1950646	0.9929685	293.0625	glutathione S-transferase mu 1
UP in U87	**8836**	GGH	1.6045892	0.9223692	3.041091	gamma-glutamyl hydrolase (conjugase, folylpolygammaglutamyl hydrolase)
UP in U87	**58492**	ZNF77	1.1251198	0.8032873	2.181197	zinc finger protein 77
UP in U87	**8310**	ACOX3	2.6931796	0.948495	6.467372	acyl-CoA oxidase 3, pristanoyl
UP in U87	**84679**	SLC9A7	1.2531747	0.866544	2.383654	solute carrier family 9, subfamily A (NHE7, cation proton antiporter 7), member 7
UP in U87	**5494**	PPM1A	1.0273804	0.8541167	2.03832	protein phosphatase, Mg2+/Mn2+ dependent, 1A
UP in U87	**339855**	KY	7.8008999	0.9044838	223	kyphoscoliosis peptidase
UP in U87	**23762**	OSBP2	1.6707424	0.8822871	3.183784	oxysterol binding protein 2
UP in U87	**440465**	BAIAP2-AS1	1.9462821	0.8721532	3.853801	BAIAP2 antisense RNA 1 (head to head)
UP in U87	**23328**	SASH1	3.0577099	0.9626291	8.326498	SAM and SH3 domain containing 1
UP in U87	**26018**	LRIG1	4.2491872	0.981919	19.0166	leucine-rich repeats and immunoglobulin-like domains 1
UP in U87	**6617**	SNAPC1	2.9146526	0.9623002	7.54046	small nuclear RNA activating complex, polypeptide 1, 43kDa
UP in U87	**202**	AIM1	3.9858132	0.9730475	15.84343	absent in melanoma 1
UP in U87	**692099**	FAM86DP	1.8470607	0.9126353	3.597665	family with sequence similarity 86, member D, pseudogene
UP in U87	**55198**	APPL2	1.8486916	0.922467	3.601734	adaptor protein, phosphotyrosine interaction, PH domain and leucine zipper containing 2
UP in U87	**80270**	HSD3B7	2.9443559	0.955731	7.697318	hydroxy-delta-5-steroid dehydrogenase, 3 beta- and steroid delta-isomerase 7
UP in U87	**817**	CAMK2D	4.9756033	0.9790478	31.46341	calcium/calmodulin-dependent protein kinase II delta
UP in U87	**84856**	LINC0083	3.9561227	0.9697406	15.52071	long intergenic non-protein coding RNA 839
UP in U87	**22911**	WDR47	1.5849625	0.9076306	3	WD repeat domain 47
UP in U87	**89795**	NAV3	1.0402245	0.8021761	2.056548	neuron navigator 3
UP in U87	**2630**	GBAP1	1.4251927	0.8156168	2.685504	glucosidase, beta, acid pseudogene 1
UP in U87	**375616**	KCP	5.9201874	0.8736377	60.55556	kielin/chordin-like protein
UP in U87	**11099**	PTPN21	1.1902224	0.8380803	2.281879	protein tyrosine phosphatase, non-receptor type 21
UP in U87	**84230**	LRRC8C	1.5501037	0.8252529	2.928382	leucine rich repeat containing 8 family,
UP in U87	**55128**	TRIM68	1.2937711	0.865744	2.451681	tripartite motif containing 68
UP in U87	**3119**	HLA-DQB1	10.529105	0.9926396	1477.667	major histocompatibility complex, class II, DQ beta 1
UP in U87	**529**	ATP6V1E1	1.5251894	0.9089818	2.878245	ATPase, H+ transporting, lysosomal 31kDa, V1 subunit E1
UP in U87	**4314**	MMP3	15.843561	1	58801.33	matrix metallopeptidase 3 (stromelysin 1, progelatinase)
UP in U87	**55049**	C19orf60	1.4964491	0.9047594	2.821474	chromosome 19 open reading frame 60
UP in U87	**4900**	NRGN	1.5692404	0.9002436	2.967484	neurogranin (protein kinase C substrate,
UP in U87	**2119**	ETV5	3.0045756	0.9639447	8.025413	ets variant 5
UP in U87	**202018**	TAPT1	1.4677136	0.8647839	2.765832	transmembrane anterior posterior transformation 1
UP in U87	**53340**	SPA17	3.8782699	0.9722741	14.70536	sperm autoantigenic protein 17
UP in U87	**57336**	ZNF287	8.2223924	0.9345832	298.6667	zinc finger protein 287
UP in U87	**84080**	ENKD1	1.4110517	0.8930521	2.65931	enkurin domain containing 1

UP in U87	**118429**	ANTXR2	8.1259452	0.9930841	279.3529	anthrax toxin receptor 2
UP in U87	**140576**	S100A16	1.4322036	0.9062794	2.698586	S100 calcium binding protein A16
UP in U87	**84964**	ALKBH6	3.1433916	0.9662204	8.835989	alkB, alkylation repair homolog 6 (E. coli)
UP in U87	**10312**	TCIRG1	1.5715797	0.9083951	2.9723	T-cell, immune regulator 1, ATPase, H+ transporting, lysosomal V0 subunit A3
UP in U87	**6339**	SCNN1D	3.6395575	0.9454904	12.46281	sodium channel, non-voltage-gated 1, delta subunit
UP in U87	**9861**	PSMD6	1.2090306	0.8963767	2.311822	proteasome (prosome, macropain) 26S subunit, non-ATPase, 6
UP in U87	**22983**	MAST1	2.6121248	0.8482586	6.114035	microtubule associated serine/threonine
UP in U87	**4644**	MYO5A	2.5631031	0.9484061	5.909774	myosin VA (heavy chain 12, myoxin)
UP in U87	**56130**	PCDHB6	8.4470832	0.9472683	349	protocadherin beta 6
UP in U87	**3953**	LEPR	1.2865578	0.8183992	2.439453	leptin receptor
UP in U87	**1514**	CTSL1	2.5453111	0.9533664	5.83734	cathepsin L1
UP in U87	**1647**	GADD45A	2.583994	0.9586556	5.995974	growth arrest and DNA-damage-inducible,
UP in U87	**4867**	NPHP1	3.8505526	0.9436414	14.42553	nephronophthisis 1 (juvenile)
UP in U87	**5252**	PHF1	1.2347706	0.8907675	2.353439	PHD finger protein 1
UP in U87	**728192**	LINC0046	8.6390392	0.9564777	398.6667	long intergenic non-protein coding RNA 460
UP in U87	**1759**	DNM1	1.3547537	0.8996213	2.557535	dynamin 1
UP in U87	**1528**	CYB5A	2.3877452	0.9496951	5.233388	cytochrome b5 type A (microsomal)
UP in U87	**151963**	MB21D2	1.599659	0.8145146	3.030717	Mab-21 domain containing 2
UP in U87	**3589**	IL11	8.3522642	0.9991111	326.8	interleukin 11
UP in U87	**153396**	TMEM161	1.771731	0.9141554	3.414634	transmembrane protein 161B
UP in U87	**22875**	ENPP4	5.6846724	0.9517041	51.43478	ectonucleotide pyrophosphatase/phosphodiesterase 4 (putative)
UP in U87	**200312**	RNF215	2.1295801	0.931223	4.375901	ring finger protein 215
UP in U87	**56834**	GPR137	1.3424106	0.8994435	2.535747	G protein-coupled receptor 137
UP in U87	**55521**	TRIM36	3.2587343	0.8873807	9.571429	tripartite motif containing 36
UP in U87	**824**	CAPN2	1.8439415	0.9281739	3.589895	calpain 2, (m/II) large subunit
UP in U87	**2863**	GPR39	4.427789	0.9267428	21.52273	G protein-coupled receptor 39
UP in U87	**79772**	MCTP1	3.0894466	0.954762	8.511696	multiple C2 domains, transmembrane 1
UP in U87	**10184**	LHFPL2	2.9637798	0.9625402	7.801653	lipoma HMGIC fusion partner-like 2
UP in U87	**84959**	UBASH3B	4.9729798	0.9727541	31.40625	ubiquitin associated and SH3 domain containing B
UP in U87	**2069**	EREG	10.207657	0.9993066	1182.526	epiregulin
UP in U87	**51510**	CHMP5	1.0997891	0.890083	2.143234	charged multivesicular body protein 5
UP in U87	**90226**	UCN2	2.5712355	0.9436681	5.943182	urocortin 2
UP in U87	**55296**	TBC1D19	3.5342808	0.9679272	11.58576	TBC1 domain family, member 19
UP in U87	**5886**	RAD23A	1.2556358	0.8994346	2.387724	RAD23 homolog A (S. cerevisiae)
UP in U87	**7542**	ZFPL1	1.1892943	0.8891852	2.280412	zinc finger protein-like 1
UP in U87	**9446**	GSTO1	2.390017	0.9521752	5.241635	glutathione S-transferase omega 1
UP in U87	**23114**	NFASC	10.875493	0.9946308	1878.667	neurofascin
UP in U87	**5523**	PPP2R3A	2.416565	0.9129642	5.338983	protein phosphatase 2, regulatory subunit B'', alpha
UP in U87	**1777**	DNASE2	1.3126758	0.8974968	2.484018	deoxyribonuclease II, lysosomal
UP in U87	**246330**	PELI3	1.4265433	0.8725621	2.688019	pellino E3 ubiquitin protein ligase family member 3
UP in U87	**10622**	POLR3G	2.3505437	0.945926	5.100164	polymerase (RNA) III (DNA directed) polypeptide G (32kD)
UP in U87	**54557**	SGTB	1.0315327	0.8535744	2.044195	small glutamine-rich tetratricopeptide repeat (TPR)-containing, beta
UP in U87	**116068**	LYSMD3	1.3067544	0.8800914	2.473844	LysM, putative peptidoglycan-binding, domain containing 3
UP in U87	**7165**	TPD52L2	1.2253169	0.8989013	2.338068	tumor protein D52-like 2
UP in U87	**23355**	VPS8	1.1589656	0.8736733	2.232973	vacuolar protein sorting 8 homolog (S.
UP in U87	**100287171**	WASH1	1.4183614	0.8897986	2.672818	WAS protein family homolog 1

UP in U87	**316**	AOX1	4.9095598	0.9744786	30.05556	aldehyde oxidase 1
UP in U87	**55113**	XKR8	4.3359926	0.986337	20.19593	XK, Kell blood group complex subunit-related family, member 8
UP in U87	**55022**	PID1	1.3198367	0.8918876	2.496379	phosphotyrosine interaction domain
UP in U87	**3385**	ICAM3	1.3905105	0.8711842	2.621714	intercellular adhesion molecule 3
UP in U87	**80007**	C10orf88	1.0968085	0.8499564	2.13881	chromosome 10 open reading frame 88
UP in U87	**11009**	IL24	5.319151	0.8634238	39.92308	interleukin 24
UP in U87	**79682**	MLF1IP	1.142958	0.85505	2.208333	MLF1 interacting protein
UP in U87	**84100**	ARL6	1.8082134	0.8850072	3.502083	ADP-ribosylation factor-like 6
UP in U87	**9839**	ZEB2	1.6217814	0.910653	3.077548	zinc finger E-box binding homeobox 2
UP in U87	**23646**	PLD3	1.5509296	0.9083684	2.930059	phospholipase D family, member 3
UP in U87	**3569**	IL6	9.9281165	0.9999111	974.2286	interleukin 6 (interferon, beta 2)
UP in U87	**161176**	SYNE3	1.8817878	0.8833538	3.685315	spectrin repeat containing, nuclear envelope family member 3
UP in U87	**23673**	STX12	1.3090226	0.892172	2.477736	syntaxin 12
UP in U87	**285282**	RABL3	1.5338552	0.8915053	2.895586	RAB, member of RAS oncogene family-like
UP in U87	**55293**	UEVLD	1.6092629	0.9135065	3.050959	UEV and lactate/malate dehyrogenase
UP in U87	**9358**	ITGBL1	2.7861161	0.9473483	6.897704	integrin, beta-like 1 (with EGF-like repeat domains)
UP in U87	**6093**	ROCK1	1.0650268	0.8790424	2.092209	Rho-associated, coiled-coil containing protein kinase 1
UP in U87	**83930**	STARD3N	1.0423187	0.8846516	2.059535	STARD3 N-terminal like
UP in U87	**285440**	CYP4V2	1.0903415	0.8388803	2.129244	cytochrome P450, family 4, subfamily V, polypeptide 2
UP in U87	**158471**	PRUNE2	3.2510446	0.8700553	9.520548	prune homolog 2 (Drosophila)
UP in U87	**221393**	GPR115	8.0775787	0.9769499	270.1429	G protein-coupled receptor 115
UP in U87	**51393**	TRPV2	3.4825615	0.9426814	11.17778	transient receptor potential cation channel, subfamily V, member 2
UP in U87	**127018**	LYPLAL1	1.675415	0.9087062	3.194112	lysophospholipase-like 1
UP in U87	**83989**	FAM172A	1.3335889	0.8766245	2.520289	family with sequence similarity 172, member
UP in U87	**23043**	TNIK	5.1679199	0.9060572	35.95	TRAF2 and NCK interacting kinase
UP in U87	**28986**	MAGEH1	3.2842084	0.9448682	9.741935	melanoma antigen family H, 1
UP in U87	**56123**	PCDHB13	4.4028481	0.9138087	21.15385	protocadherin beta 13
UP in U87	**55095**	SAMD4B	1.3258308	0.8991946	2.506772	sterile alpha motif domain containing 4B
UP in U87	**7328**	UBE2H	1.0416947	0.885425	2.058644	ubiquitin-conjugating enzyme E2H
UP in U87	**6674**	SPAG1	1.3881762	0.8343112	2.617476	sperm associated antigen 1
UP in U87	**9751**	SNPH	2.8849379	0.9199868	7.38674	syntaphilin
UP in U87	**23293**	SMG6	1.3093425	0.8871762	2.478286	smg-6 homolog, nonsense mediated mRNA decay factor (C. elegans)
UP in U87	**266727**	MDGA1	5.7251958	0.868073	52.9	MAM domain containing glycosylphosphatidylinositol anchor 1
UP in U87	**120892**	LRRK2	7.4234661	0.8687308	171.6667	leucine-rich repeat kinase 2
UP in U87	**127544**	RNF19B	1.4089459	0.8873362	2.655431	ring finger protein 19B
UP in U87	**59269**	HIVEP3	2.987176	0.9357655	7.929204	human immunodeficiency virus type I enhancer binding protein 3
UP in U87	**389**	RHOC	1.9417607	0.9294629	3.841742	ras homolog family member C
UP in U87	**25758**	KIAA1549	8.2495085	0.9361121	304.3333	KIAA1549-like
UP in U87	**6649**	SOD3	6.2418748	0.970114	75.68182	superoxide dismutase 3, extracellular
UP in U87	**57606**	SLAIN2	1.5348024	0.9005903	2.897487	SLAIN motif family, member 2
UP in U87	**10254**	STAM2	2.3652617	0.942228	5.152461	signal transducing adaptor molecule (SH3 domain and ITAM motif) 2
UP in U87	**57679**	ALS2	2.0900102	0.9240671	4.257511	amyotrophic lateral sclerosis 2 (juvenile)
UP in U87	**83692**	CD99L2	1.1175654	0.8835671	2.169805	CD99 molecule-like 2
UP in U87	**1130**	LYST	1.3173225	0.8221594	2.492032	lysosomal trafficking regulator
UP in U87	**399474**	TMEM200	1.5368518	0.8499298	2.901606	transmembrane protein 200B
UP in U87	**87**	ACTN1	1.4431014	0.9062528	2.719048	actinin, alpha 1
UP in U87	**5912**	RAP2B	1.3219281	0.8656462	2.5	RAP2B, member of RAS oncogene family

UP in U87	**1440**	CSF3	16.065893	1	68598.67	colony stimulating factor 3 (granulocyte)
UP in U87	**348013**	TMEM255	9.4220648	0.97975	686	transmembrane protein 255B
UP in U87	**51762**	RAB8B	1.7063237	0.9170089	3.263282	RAB8B, member RAS oncogene family
UP in U87	**56133**	PCDHB2	5.8191146	0.9595623	56.45833	protocadherin beta 2
UP in U87	**169834**	ZNF883	2.0274807	0.8412004	4.076923	zinc finger protein 883
UP in U87	**90853**	SPOCD1	8.3281941	0.9966843	321.3929	SPOC domain containing 1
UP in U87	**3908**	LAMA2	6.3531468	0.8995324	81.75	laminin, alpha 2
UP in U87	**81557**	MAGED4	8.4864999	0.9491795	358.6667	melanoma antigen family D, 4B
UP in U87	**58528**	RRAGD	9.7039036	0.9846481	834	Ras-related GTP binding D
UP in U87	**55323**	LARP6	2.9067148	0.9649048	7.499086	La ribonucleoprotein domain family, member
UP in U87	**80339**	PNPLA3	1.6776914	0.9107152	3.199156	patatin-like phospholipase domain containing
UP in U87	**10650**	SLMO1	1.2795579	0.8051185	2.427646	slowmo homolog 1 (Drosophila)
UP in U87	**285704**	RGMB	1.8978455	0.9131598	3.726562	RGM domain family, member B
UP in U87	**100132288**	TEKT4P2	1.9736515	0.9044216	3.927609	tektin 4 pseudogene 2
UP in U87	**5743**	PTGS2	5.870002	0.9958042	58.48529	prostaglandin-endoperoxide synthase 2 (prostaglandin G/H synthase and cyclooxygenase)
UP in U87	**27350**	APOBEC3C	5.5507183	0.9945864	46.87407	apolipoprotein B mRNA editing enzyme, catalytic polypeptide-like 3C
UP in U87	**1174**	AP1S1	1.8934818	0.9285206	3.715308	adaptor-related protein complex 1, sigma 1 subunit
UP in U87	**6482**	ST3GAL1	2.385431	0.950744	5.225	ST3 beta-galactoside alpha-2,3-
UP in U87	**1508**	CTSB	2.1385453	0.941828	4.403178	cathepsin B
UP in U87	**8718**	TNFRSF25	2.4511508	0.9409391	5.468521	tumor necrosis factor receptor superfamily, member 25
UP in U87	**5329**	PLAUR	2.8341077	0.9650915	7.131016	plasminogen activator, urokinase receptor
UP in U87	**83699**	SH3BGRL2	7.2055489	0.9151333	147.6	SH3 domain binding glutamic acid-rich protein like 2
UP in U87	**23229**	ARHGEF9	1.9034627	0.8592103	3.7411	Cdc42 guanine nucleotide exchange factor (GEF) 9
UP in U87	**165215**	FAM171B	4.0478719	0.9613402	16.53982	family with sequence similarity 171, member
UP in U87	**113419**	TEX261	1.0628785	0.887434	2.089096	testis expressed 261
UP in U87	**57732**	ZFYVE28	1.7731778	0.8937188	3.41806	zinc finger, FYVE domain containing 28
UP in U87	**23275**	POFUT2	2.3151798	0.9470105	4.976667	protein O-fucosyltransferase 2
UP in U87	**8618**	CADPS	9.8073549	0.9861059	896	Ca++-dependent secretion activator
UP in U87	**8829**	NRP1	3.7906419	0.9817412	13.83875	neuropilin 1
UP in U87	**10435**	CDC42EP	1.1395899	0.8868384	2.203184	CDC42 effector protein (Rho GTPase
UP in U87	**23708**	GSPT2	9.0856937	0.971634	543.3333	G1 to S phase transition 2
UP in U87	**10488**	CREB3	1.1151376	0.8896563	2.166157	cAMP responsive element binding protein 3
UP in U87	**3425**	IDUA	2.0575025	0.9113997	4.162651	iduronidase, alpha-L-
UP in U87	**4355**	MPP2	1.5299811	0.8844294	2.887821	membrane protein, palmitoylated 2 (MAGUK p55 subfamily member 2)
UP in U87	**598**	BCL2L1	1.6955254	0.924556	3.238948	BCL2-like 1
UP in U87	**124989**	EFCAB13	2.8207754	0.8672818	7.065421	EF-hand calcium binding domain 13
UP in U87	**6035**	RNASE1	4.7548875	0.829422	27	ribonuclease, RNase A family, 1 (pancreatic)
UP in U87	**11217**	AKAP2	1.2975567	0.8982701	2.458122	A kinase (PRKA) anchor protein 2
UP in U87	**23314**	SATB2	3.5409206	0.9696873	11.6392	SATB homeobox 2
UP in U87	**386618**	KCTD4	9.5943246	0.9829058	773	potassium channel tetramerisation domain containing 4
UP in U87	**100131691**	LOC100131691	2.0266448	0.8521699	4.074561	uncharacterized LOC100131691
UP in U87	**79962**	DNAJC22	1.9285495	0.8351379	3.806723	DnaJ (Hsp40) homolog, subfamily C,
UP in U87	**8988**	HSPB3	3.2568331	0.8615837	9.558824	heat shock 27kDa protein 3
UP in U87	**9331**	B4GALT6	2.357858	0.8922076	5.126087	UDP-Gal:betaGlcNAc beta 1,4-galactosyltransferase, polypeptide 6
UP in U87	**7050**	TGIF1	1.1576352	0.8903053	2.230914	TGFB-induced factor homeobox 1
UP in U87	**116372**	LYPD1	3.6527071	0.8740199	12.57692	LY6/PLAUR domain containing 1

UP in U87	**54834**	GDAP2	1.2107202	0.8619304	2.314532	ganglioside induced differentiation associated protein 2
UP in U87	**29956**	CERS2	1.129558	0.8924654	2.187917	ceramide synthase 2
UP in U87	**388849**	LOC38884	1.9424662	0.8388981	3.843621	uncharacterized LOC388849
UP in U87	**8559**	PRPF18	1.024539	0.8706064	2.034309	PRP18 pre-mRNA processing factor 18 homolog (S. cerevisiae)
UP in U87	**84246**	MED10	1.012933	0.8839494	2.018009	mediator complex subunit 10
UP in U87	**64221**	ROBO3	5.0326886	0.8527921	32.73333	roundabout, axon guidance receptor, homolog 3 (Drosophila)
UP in U87	**11127**	KIF3A	1.0071776	0.8139634	2.009975	kinesin family member 3A
UP in U87	**114880**	OSBPL6	3.4229717	0.9549398	10.72549	oxysterol binding protein-like 6
UP in U87	**6648**	SOD2	3.4313639	0.9759809	10.78806	superoxide dismutase 2, mitochondrial
UP in U87	**2132**	EXT2	1.0995542	0.8874962	2.142885	exostosin glycosyltransferase 2
UP in U87	**79669**	C3orf52	1.5524285	0.8630149	2.933105	chromosome 3 open reading frame 52
UP in U87	**80201**	HKDC1	7.9715436	0.9818835	251	hexokinase domain containing 1
UP in U87	**7280**	TUBB2A	2.5081873	0.9492595	5.689048	tubulin, beta 2A class IIa
UP in U87	**5516**	PPP2CB	1.2575809	0.8980212	2.390945	protein phosphatase 2, catalytic subunit, beta isozyme
UP in U87	**1263**	PLK3	1.9167406	0.9141465	3.775691	polo-like kinase 3
UP in U87	**7107**	GPR137B	2.5639898	0.9282451	5.913408	G protein-coupled receptor 137B
UP in U87	**572**	BAD	1.1511214	0.8921809	2.220865	BCL2-associated agonist of cell death
UP in U87	**23417**	MLYCD	1.4143477	0.8385781	2.665392	malonyl-CoA decarboxylase
UP in U87	**10005**	ACOT8	1.3262404	0.8979234	2.507484	acyl-CoA thioesterase 8
UP in U87	**201266**	SLC39A11	1.4903256	0.8708109	2.809524	solute carrier family 39 (metal ion transporter), member 11
UP in U87	**2622**	GAS8	1.3692338	0.8879051	2.583333	growth arrest-specific 8
UP in U87	**8092**	ALX1	5.7842713	0.9392857	55.11111	ALX homeobox 1
UP in U87	**627**	BDNF	1.6593447	0.8828026	3.15873	brain-derived neurotrophic factor
UP in U87	**284297**	SSC5D	6.4024307	0.9746564	84.59091	scavenger receptor cysteine rich domain containing (5 domains)
UP in U87	**11067**	C10orf10	6.5949466	0.9937063	96.66667	chromosome 10 open reading frame 10
UP in U87	**23038**	WDTC1	1.5879541	0.9146354	3.006227	WD and tetratricopeptide repeats 1
UP in U87	**379**	ARL4D	2.1176458	0.9371967	4.339852	ADP-ribosylation factor-like 4D
UP in U87	**9572**	NR1D1	2.1751314	0.9380145	4.516269	nuclear receptor subfamily 1, group D,
UP in U87	**2260**	FGFR1	3.9260439	0.9834124	15.20047	fibroblast growth factor receptor 1
UP in U87	**83444**	INO80B	1.2475018	0.8887496	2.374299	INO80 complex subunit B
UP in U87	**1002**	CDH4	4.0669067	0.9455704	16.75949	cadherin 4, type 1, R-cadherin (retinal)
UP in U87	**54849**	DEF8	1.7655604	0.9233648	3.40006	differentially expressed in FDCP 8 homolog (mouse)
UP in U87	**4925**	NUCB2	1.5169641	0.9045016	2.861882	nucleobindin 2
UP in U87	**23467**	NPTXR	3.35813	0.972603	10.25411	neuronal pentraxin receptor
UP in U87	**7128**	TNFAIP3	3.1927027	0.961918	9.143223	tumor necrosis factor, alpha-induced protein
UP in U87	**100526783**	C15orf38-AP3S2	1.3020173	0.8492809	2.465734	C15orf38-AP3S2 readthrough
UP in U87	**6804**	STX1A	3.8843799	0.9827546	14.76777	syntaxin 1A (brain)
UP in U87	**285761**	DCBLD1	1.1400332	0.8648817	2.203861	discoidin, CUB and LCCL domain
UP in U87	**26093**	CCDC9	1.5293399	0.8984479	2.886537	coiled-coil domain containing 9
UP in U87	**718**	C3	4.1796399	0.9887194	18.12162	complement component 3
UP in U87	**441094**	FLJ42709	2.3915239	0.9287695	5.247113	uncharacterized LOC441094
UP in U87	**114928**	GPRASP2	4.9394246	0.9472239	30.68421	G protein-coupled receptor associated sorting protein 2
UP in U87	**55614**	KIF16B	1.1776745	0.8086031	2.262118	kinesin family member 16B
UP in U87	**51161**	C3orf18	1.5123894	0.8979234	2.852821	chromosome 3 open reading frame 18
UP in U87	**167359**	NIM1	3.6785138	0.8740199	12.80392	serine/threonine-protein kinase NIM1
UP in U87	**57169**	ZNFX1	1.4518761	0.9015592	2.735636	zinc finger, NFX1-type containing 1
UP in U87	**7515**	XRCC1	1.1145169	0.8872029	2.165225	X-ray repair complementing defective repair in Chinese hamster cells 1

UP in U87	**5704**	PSMC4	1.2032223	0.8966167	2.302534	proteasome (prosome, macropain) 26S subunit, ATPase, 4
UP in U87	**9823**	ARMCX2	9.1160551	0.9938308	554.8889	armadillo repeat containing, X-linked 2
UP in U87	**81847**	RNF146	2.116514	0.9308496	4.336449	ring finger protein 146
UP in U87	**54951**	COMMD8	1.5439529	0.9025192	2.915924	COMM domain containing 8
UP in U87	**2781**	GNAZ	1.5506288	0.808932	2.929448	guanine nucleotide binding protein (G protein), alpha z polypeptide
UP in U87	**91147**	TMEM67	1.1796533	0.8361513	2.265223	transmembrane protein 67
UP in U87	**79875**	THSD4	1.1824475	0.8317777	2.269615	thrombospondin, type I, domain containing 4
UP in U87	**55337**	C19orf66	3.3820111	0.9667893	10.42526	chromosome 19 open reading frame 66
UP in U87	**54532**	USP53	1.3910206	0.8663218	2.622642	ubiquitin specific peptidase 53
UP in U87	**79792**	GSDMD	1.3174905	0.892412	2.492322	gasdermin D
UP in U87	**373156**	GSTK1	1.0741352	0.8898075	2.10546	glutathione S-transferase kappa 1
UP in U87	**51098**	IFT52	1.5174518	0.9038615	2.86285	intraflagellar transport 52 homolog (Chlamydomonas)
UP in U87	**23087**	TRIM35	1.5937804	0.9109375	3.018392	tripartite motif containing 35
UP in U87	**5498**	PPOX	1.0388496	0.8516099	2.054589	protoporphyrinogen oxidase
UP in U87	**91782**	CHMP7	1.1850514	0.8907408	2.273715	charged multivesicular body protein 7
UP in U87	**84466**	MEGF10	7.2159374	0.8457696	148.6667	multiple EGF-like-domains 10
UP in U87	**5998**	RGS3	4.4612506	0.9909595	22.02776	regulator of G-protein signaling 3
UP in U87	**23139**	MAST2	1.3770783	0.8994257	2.597418	microtubule associated serine/threonine
UP in U87	**51231**	VRK3	1.3178727	0.8886341	2.492982	vaccinia related kinase 3
UP in U87	**23051**	ZHX3	1.366729	0.8902075	2.578852	zinc fingers and homeoboxes 3
UP in U87	**51320**	MEX3C	1.566097	0.9014436	2.961026	mex-3 homolog C (C. elegans)
UP in U87	**5794**	PTPRH	5.8645989	0.9285384	58.26667	protein tyrosine phosphatase, receptor type,
UP in U87	**6785**	ELOVL4	4.547953	0.9461038	23.39216	ELOVL fatty acid elongase 4
UP in U87	**9500**	MAGED1	2.1769592	0.9421302	4.521994	melanoma antigen family D, 1
UP in U87	**9805**	SCRN1	3.3343972	0.9749231	10.0868	secernin 1
UP in U87	**57149**	LYRM1	2.2167553	0.9384501	4.648468	LYR motif containing 1
UP in U87	**2892**	GRIA3	7.0370893	0.8236884	131.3333	glutamate receptor, ionotropic, AMPA 3
UP in U87	**9947**	MAGEC1	10.958553	0.9950397	1990	melanoma antigen family C, 1
UP in U87	**57142**	RTN4	1.0484284	0.8893008	2.068276	reticulon 4
UP in U87	**80853**	JHDM1D	1.6578131	0.8023006	3.155378	jumonji C domain containing histone demethylase 1 homolog D (S. cerevisiae)
UP in U87	**83938**	C10orf11	5.4034151	0.9651893	42.32432	chromosome 10 open reading frame 11
UP in U87	**10253**	SPRY2	5.8314004	0.9964709	56.94118	sprouty homolog 2 (Drosophila)
UP in U87	**84861**	KLHL22	1.9168936	0.8967767	3.776091	kelch-like family member 22
UP in U87	**3934**	LCN2	7.5104345	0.8776557	182.3333	lipocalin 2
UP in U87	**91752**	ZNF804A	9.7039036	0.9846481	834	zinc finger protein 804A
UP in U87	**1808**	DPYSL2	1.1882809	0.8930432	2.27881	dihydropyrimidinase-like 2
UP in U87	**340252**	ZNF680	1.1290301	0.8156613	2.187117	zinc finger protein 680
UP in U87	**23541**	SEC14L2	6.0197019	0.9952353	64.88	SEC14-like 2 (S. cerevisiae)
UP in U87	**6461**	SHB	1.9304244	0.9200935	3.811673	Src homology 2 domain containing adaptor protein B
UP in U87	**79993**	ELOVL7	5.4830829	0.9858126	44.72727	ELOVL fatty acid elongase 7
UP in U87	**23094**	SIPA1L3	2.1341834	0.9220403	4.389886	signal-induced proliferation-associated 1 like
UP in U87	**10152**	ABI2	1.6589421	0.9184134	3.157849	abl-interactor 2
UP in U87	**730091**	LOC73009	5.3827441	0.9117731	41.72222	uncharacterized LOC730091
UP in U87	**60412**	EXOC4	1.3440292	0.8997724	2.538593	exocyst complex component 4
UP in U87	**3661**	IRF3	1.5752852	0.9199068	2.979944	interferon regulatory factor 3
UP in U87	**1794**	DOCK2	3.1638632	0.8081942	8.962264	dedicator of cytokinesis 2
UP in U87	**112849**	L3HYPDH	1.5290931	0.9020837	2.886044	L-3-hydroxyproline dehydratase (trans-)
UP in U87	**27010**	TPK1	2.9335726	0.830471	7.64	thiamin pyrophosphokinase 1
UP in U87	**2294**	FOXF1	4.4616159	0.9868171	22.03333	forkhead box F1
UP in U87	**79004**	CUEDC2	1.3805531	0.903346	2.603682	CUE domain containing 2
UP in U87	**55624**	POMGNT 1	1.586495	0.9211514	3.003188	protein O-linked mannose beta1,2-N-acetylglucosaminyltransferase

UP in U87	**100506123**	LOC100506123	1.5979016	0.8937099	3.027027	uncharacterized LOC100506123
UP in U87	**3237**	HOXD11	4.7318039	0.9282006	26.57143	homeobox D11
UP in U87	**79674**	VEPH1	2.2199694	0.9334809	4.658836	ventricular zone expressed PH domain-containing 1
UP in U87	**57574**	4-Mar	9.5176694	0.9815012	733	membrane-associated ring finger (C3HC4) 4, E3 ubiquitin protein ligase
UP in U87	**3074**	HEXB	1.771229	0.9263161	3.413446	hexosaminidase B (beta polypeptide)
UP in U87	**352909**	DNAAF3	1.9625253	0.8141323	3.897436	dynein, axonemal, assembly factor 3
UP in U87	**3109**	HLA-DMB	6.5688428	0.9637758	94.93333	major histocompatibility complex, class II, DM beta
UP in U87	**11015**	KDELR3	3.1495153	0.9661938	8.873574	KDEL (Lys-Asp-Glu-Leu) endoplasmic reticulum protein retention receptor 3
UP in U87	**728912**	NBPF24	1.2574167	0.8764023	2.390673	neuroblastoma breakpoint family, member 24
UP in U87	**8850**	KAT2B	1.2018321	0.8560634	2.300316	K(lysine) acetyltransferase 2B
UP in U87	**3431**	SP110	2.1895414	0.9013459	4.561605	SP110 nuclear body protein
UP in U87	**83464**	APH1B	2.9130167	0.9563888	7.531915	anterior pharynx defective 1 homolog B (C. elegans)
UP in U87	**9399**	STOML1	1.0529444	0.8598236	2.07476	stomatin (EPB72)-like 1
UP in U87	**55859**	BEX1	5.7255197	0.9952531	52.91188	brain expressed, X-linked 1
UP in U87	**152098**	ZCWPW2	5.7004397	0.829022	52	zinc finger, CW type with PWWP domain 2
UP in U87	**85315**	PAQR8	2.8535662	0.828622	7.227848	progestin and adipoQ receptor family member VIII
UP in U87	**89927**	C16orf45	2.831423	0.9634292	7.117759	chromosome 16 open reading frame 45
UP in U87	**55520**	ELAC1	2.7796099	0.8722776	6.866667	elaC homolog 1 (E. coli)
UP in U87	**90637**	ZFAND2A	1.507737	0.903666	2.843636	zinc finger, AN1-type domain 2A
UP in U87	**8321**	FZD1	1.3468193	0.8585258	2.543507	frizzled family receptor 1
UP in U87	**286097**	MICU3	1.8873422	0.8161413	3.699531	mitochondrial calcium uptake family,
UP in U87	**23603**	CORO1C	1.2142771	0.8966523	2.320245	coronin, actin binding protein, 1C
UP in U87	**7466**	WFS1	1.5993766	0.9152933	3.030123	Wolfram syndrome 1 (wolframin)
UP in U87	**731220**	RFX8	9.9063896	0.987226	959.6667	RFX family member 8, lacking RFX DNA binding domain
UP in U87	**80313**	LRRC27	1.5740367	0.8513432	2.977366	leucine rich repeat containing 27
UP in U87	**83895**	KRTAP1-5	7.8483384	0.9845147	230.4545	keratin associated protein 1-5
UP in U87	**2690**	GHR	3.7780771	0.8149857	13.71875	growth hormone receptor
UP in U87	**89910**	UBE3B	1.2227399	0.8879762	2.333895	ubiquitin protein ligase E3B
UP in U87	**6676**	SPAG4	2.6644205	0.9384323	6.339726	sperm associated antigen 4
UP in U87	**54935**	DUSP23	1.4503488	0.8992835	2.732741	dual specificity phosphatase 23
UP in U87	**272**	AMPD3	8.328793	0.9949686	321.5263	adenosine monophosphate deaminase 3
UP in U87	**9583**	ENTPD4	1.6439199	0.9188401	3.125138	ectonucleoside triphosphate diphosphohydrolase 4
UP in U87	**9149**	DYRK1B	1.2226444	0.8500098	2.333741	dual-specificity tyrosine-(Y)-phosphorylation regulated kinase 1B
UP in U87	**10656**	KHDRBS3	2.0213449	0.8937899	4.059621	KH domain containing, RNA binding, signal transduction associated 3
UP in U87	**2081**	ERN1	1.8836759	0.8696019	3.690141	endoplasmic reticulum to nucleus signaling 1
UP in U87	**91694**	LONRF1	1.3782619	0.8710242	2.59955	LON peptidase N-terminal domain and ring finger 1
UP in U87	**440224**	CXADRP3	4.716207	0.8000071	26.28571	coxsackie virus and adenovirus receptor pseudogene 3
UP in U87	**9217**	VAPB	1.2571522	0.8909809	2.390235	VAMP (vesicle-associated membrane protein)-associated protein B and C
UP in U87	**9694**	EMC2	1.1766343	0.891852	2.260488	ER membrane protein complex subunit 2
UP in U87	**58472**	SQRDL	5.7154803	0.9964798	52.54495	sulfide quinone reductase-like (yeast)
UP in U87	**147965**	FAM98C	1.2651583	0.8768201	2.403536	family with sequence similarity 98, member
UP in U87	**387923**	SERP2	8.5999128	0.954682	388	stress-associated endoplasmic reticulum protein family member 2

UP in U87	**25978**	CHMP2B	1.7296022	0.9205202	3.316364	charged multivesicular body protein 2B
UP in U87	**57221**	KIAA1244	4.8107858	0.8249684	28.06667	KIAA1244
UP in U87	**22931**	RAB18	1.6326231	0.9180579	3.100763	RAB18, member RAS oncogene family
UP in U87	**2281**	FKBP1B	3.3713968	0.9332587	10.34884	FK506 binding protein 1B, 12.6 kDa
UP in U87	**55**	ACPP	4.2055489	0.9364144	18.45	acid phosphatase, prostate
UP in U87	**84874**	ZNF514	2.2352512	0.906555	4.708447	zinc finger protein 514
UP in U87	**55248**	TMEM206	1.2903925	0.8821182	2.445946	transmembrane protein 206
UP in U87	**151827**	LRRC34	5.6180708	0.9696606	49.11429	leucine rich repeat containing 34
UP in U87	**54872**	PIGG	1.185355	0.8863673	2.274194	phosphatidylinositol glycan anchor biosynthesis, class G
UP in U87	**375346**	TMEM110	1.3431574	0.8636283	2.53706	transmembrane protein 110
UP in U87	**10938**	EHD1	2.157363	0.9415169	4.460987	EH-domain containing 1
UP in U87	**100302736**	TMED7-TICAM2	1.6522444	0.8536277	3.143223	TMED7-TICAM2 readthrough
UP in U87	**11078**	TRIOBP	2.1981495	0.9414102	4.588903	TRIO and F-actin binding protein
UP in U87	**57531**	HACE1	1.1913141	0.8202837	2.283607	HECT domain and ankyrin repeat containing E3 ubiquitin protein ligase 1
UP in U87	**100126784**	LOC100126784	4.5505021	0.9835013	23.43353	uncharacterized LOC100126784
UP in U87	**51227**	PIGP	1.072253	0.8787402	2.102715	phosphatidylinositol glycan anchor biosynthesis, class P
UP in U87	**64283**	ARHGEF2	2.1633884	0.9137821	4.479657	Rho guanine nucleotide exchange factor
UP in U87	**100131089**	LOC100131089	2.3361153	0.9258983	5.049412	uncharacterized LOC100131089
UP in U87	**36**	ACADSB	1.0271671	0.8244351	2.038018	acyl-CoA dehydrogenase, short/branched
UP in U87	**100631383**	FAM47E-STBD1	7.6911619	0.8958433	206.6667	FAM47E-STBD1 readthrough
UP in U87	**26151**	NAT9	1.0090601	0.8760378	2.012599	N-acetyltransferase 9 (GCN5-related,
UP in U87	**2180**	ACSL1	1.9111907	0.9207069	3.761194	acyl-CoA synthetase long-chain family
UP in U87	**728743**	LOC72874	2.4127815	0.823555	5.325	zinc finger protein pseudogene
UP in U87	**1428**	CRYM	9.6023896	0.9830035	777.3333	crystallin, mu
UP in U87	**55297**	CCDC91	1.2316221	0.8845983	2.348309	coiled-coil domain containing 91
UP in U87	**146712**	B3GNTL1	1.9166716	0.9011947	3.77551	UDP-GlcNAc:betaGal beta-1,3-N-acetylglucosaminyltransferase-like 1
UP in U87	**6450**	SH3BGR	5.180178	0.9809234	36.25676	SH3 domain binding glutamic acid-rich
UP in U87	**64411**	ARAP3	2.2358703	0.9156399	4.710468	ArfGAP with RhoGAP domain, ankyrin repeat and PH domain 3
UP in U87	**55893**	ZNF395	2.0203742	0.9246182	4.05689	zinc finger protein 395
UP in U87	**284942**	RPL23AP8	1.8907709	0.9232492	3.708333	ribosomal protein L23a pseudogene 82
UP in U87	**9657**	IQCB1	1.0822361	0.8704198	2.117315	IQ motif containing B1
UP in U87	**5080**	PAX6	4.2764453	0.8649972	19.37931	paired box 6
UP in U87	**112939**	NACC1	1.1362016	0.8923765	2.198016	nucleus accumbens associated 1, BEN and BTB (POZ) domain containing
UP in U87	**10487**	CAP1	1.3002434	0.9012925	2.462704	CAP, adenylate cyclase-associated protein 1 (yeast)
UP in U87	**8555**	CDC14B	2.1714509	0.9101819	4.504762	cell division cycle 14B
UP in U87	**349667**	RTN4RL2	4.1882552	0.970114	18.23016	reticulon 4 receptor-like 2
UP in U87	**54472**	TOLLIP	1.8187145	0.9239248	3.527667	toll interacting protein
UP in U87	**92691**	TMEM169	7.1154772	0.8338667	138.6667	transmembrane protein 169
UP in U87	**19**	ABCA1	8.4871701	0.9952886	358.8333	ATP-binding cassette, sub-family A (ABC1), member 1
UP in U87	**145407**	C14orf37	7.8508566	0.9708251	230.8571	chromosome 14 open reading frame 37
UP in U87	**9181**	ARHGEF2	2.0993021	0.9405035	4.285021	Rho/Rac guanine nucleotide exchange factor (GEF) 2
UP in U87	**387882**	C12orf75	3.1076386	0.9690028	8.619706	chromosome 12 open reading frame 75
UP in U87	**10765**	KDM5B	6.4097681	0.9893683	85.02222	lysine (K)-specific demethylase 5B
UP in U87	**1070**	CETN3	1.3689905	0.8969012	2.582898	centrin, EF-hand protein, 3

UP in U87	**3117**	HLA-DQA1	7.1258439	0.835049	139.6667	major histocompatibility complex, class II, DQ alpha 1
UP in U87	**388121**	TNFAIP8L3	5.0835557	0.9820435	33.90805	tumor necrosis factor, alpha-induced protein 8-like 3
UP in U87	**23287**	AGTPBP1	1.1693334	0.8597525	2.249077	ATP/GTP binding protein 1
UP in U87	**57178**	ZMIZ1	1.4839213	0.9044305	2.79708	zinc finger, MIZ-type containing 1
UP in U87	**7010**	TEK	6.033423	0.8678063	65.5	TEK tyrosine kinase, endothelial
UP in U87	**79925**	SPEF2	4.3667823	0.806363	20.63158	sperm flagellar 2
UP in U87	**10318**	TNIP1	2.3477345	0.9517219	5.090243	TNFAIP3 interacting protein 1
UP in U87	**55531**	ELMOD1	2.4340067	0.9053461	5.403922	ELMO/CED-12 domain containing 1
UP in U87	**29063**	ZCCHC4	1.6447453	0.9073017	3.126927	zinc finger, CCHC domain containing 4
UP in U87	**968**	CD68	5.2106742	0.995342	37.03132	CD68 molecule
UP in U87	**149345**	SHISA4	2.8154089	0.95742	7.039187	shisa homolog 4 (Xenopus laevis)
UP in U87	**1293**	COL6A3	9.7468559	0.9998489	859.2034	collagen, type VI, alpha 3
UP in U87	**10771**	ZMYND11	1.2661474	0.892492	2.405184	zinc finger, MYND-type containing 11
UP in U87	**2303**	FOXC2	2.9890289	0.9366366	7.939394	forkhead box C2 (MFH-1, mesenchyme forkhead 1)
UP in U87	**9263**	STK17A	1.0347437	0.8806514	2.04875	serine/threonine kinase 17a
UP in U87	**84455**	EFCAB7	1.158337	0.8333067	2.232	EF-hand calcium binding domain 7
UP in U87	**200734**	SPRED2	1.8241705	0.9201557	3.541033	sprouty-related, EVH1 domain containing 2
UP in U87	**4862**	NPAS2	2.4627212	0.9481128	5.512555	neuronal PAS domain protein 2
UP in U87	**93973**	ACTR8	1.1799444	0.877789	2.26568	ARP8 actin-related protein 8 homolog (yeast)
UP in U87	**124152**	IQCK	1.1395514	0.8162035	2.203125	IQ motif containing K
UP in U87	**8440**	NCK2	1.8387191	0.9163867	3.576923	NCK adaptor protein 2
UP in U87	**254228**	FAM26E	8.7335803	0.9600512	425.6667	family with sequence similarity 26, member
UP in U87	**148022**	TICAM1	1.0277485	0.8678418	2.03884	toll-like receptor adaptor molecule 1
UP in U87	**27286**	SRPX2	4.4009933	0.9788078	21.12667	sushi-repeat containing protein, X-linked 2
UP in U87	**89848**	FCHSD1	2.5263132	0.9408324	5.760976	FCH and double SH3 domains 1
UP in U87	**10391**	CORO2B	11.902626	0.9977777	3828.667	coronin, actin binding protein, 2B
UP in U87	**100287616**	LOXL1-	1.71489	0.9023415	3.282716	LOXL1 antisense RNA 1
UP in U87	**55653**	BCAS4	2.0242249	0.929934	4.067733	breast carcinoma amplified sequence 4
UP in U87	**222950**	NYAP1	3.1604647	0.8009849	8.941176	neuronal tyrosine-phosphorylated phosphoinositide-3-kinase adaptor 1
UP in U87	**7318**	UBA7	3.935679	0.943757	15.30233	ubiquitin-like modifier activating enzyme 7
UP in U87	**64598**	MOSPD3	1.0234709	0.8675218	2.032804	motile sperm domain containing 3
UP in U87	**4311**	MME	3.5371421	0.9745586	11.60876	membrane metallo-endopeptidase
UP in U87	**93109**	TMEM44	1.517286	0.8992035	2.86252	transmembrane protein 44
UP in U87	**414**	ARSD	1.7966285	0.8897986	3.474074	arylsulfatase D
UP in U87	**3673**	ITGA2	6.0551102	0.9964443	66.49206	integrin, alpha 2 (CD49B, alpha 2 subunit of VLA-2 receptor)
UP in U87	**23324**	MAN2B2	2.496134	0.9477305	5.641716	mannosidase, alpha, class 2B, member 2
UP in U87	**83877**	TM2D2	1.197634	0.8790869	2.293632	TM2 domain containing 2
UP in U87	**25851**	TECPR1	1.3636252	0.8797714	2.57331	tectonin beta-propeller repeat containing 1
UP in U87	**267**	AMFR	1.3582979	0.9003591	2.563825	autocrine motility factor receptor, E3 ubiquitin protein ligase
UP in U87	**57045**	TWSG1	2.4895269	0.9493129	5.615938	twisted gastrulation homolog 1 (Drosophila)
UP in U87	**9296**	ATP6V1F	1.9495996	0.929694	3.862673	ATPase, H+ transporting, lysosomal 14kDa, V1 subunit F
UP in U87	**9424**	KCNK6	7.6390392	0.890323	199.3333	potassium channel, subfamily K, member 6
UP in U87	**64837**	KLC2	1.1962945	0.8921542	2.291504	kinesin light chain 2
UP in U87	**5891**	MOK	3.4831825	0.9721052	11.18259	MOK protein kinase
UP in U87	**133383**	SETD9	2.2505435	0.876429	4.758621	SET domain containing 9
UP in U87	**100506178**	LOC100506178	7.3706874	0.9425125	165.5	uncharacterized LOC100506178
UP in U87	**56548**	CHST7	2.5134412	0.9369477	5.709804	carbohydrate (N-acetylglucosamine 6-O) sulfotransferase 7
UP in U87	**2531**	KDSR	1.9380239	0.9241471	3.831804	3-ketodihydrosphingosine reductase

UP in U87	**57821**	CCDC181	5.9034515	0.8306132	59.85714	coiled-coil domain containing 181
UP in U87	**5789**	PTPRD	7.4234661	0.8687308	171.6667	protein tyrosine phosphatase, receptor type,
UP in U87	**6334**	SCN8A	7.1759237	0.9128665	144.6	sodium channel, voltage gated, type VIII, alpha subunit
UP in U87	**9498**	SLC4A8	2.9450621	0.9239782	7.701087	solute carrier family 4, sodium bicarbonate cotransporter, member 8
UP in U87	**26133**	TRPC4AP	1.3968902	0.9040838	2.633333	transient receptor potential cation channel, subfamily C, member 4 associated protein
UP in U87	**124044**	SPATA2L	1.9876917	0.8882163	3.966019	spermatogenesis associated 2-like
UP in U87	**7425**	VGF	5.4995052	0.9904084	45.23932	VGF nerve growth factor inducible
UP in U87	**4675**	NAP1L3	6.6244909	0.9320497	98.66667	nucleosome assembly protein 1-like 3
UP in U87	**131566**	DCBLD2	4.7992438	0.9934663	27.84302	discoidin, CUB and LCCL domain
UP in U87	**338692**	ANKRD13	1.2264376	0.8862962	2.339885	ankyrin repeat domain 13 family, member D
UP in U87	**122616**	C14orf79	1.1250014	0.851201	2.181018	chromosome 14 open reading frame 79
UP in U87	**23148**	NACAD	2.6136453	0.8902164	6.120482	NAC alpha domain containing
UP in U87	**57458**	TMCC3	6.1713555	0.9417836	72.07143	transmembrane and coiled-coil domain
UP in U87	**84996**	C21orf119	2.282934	0.9081995	4.866667	chromosome 21 open reading frame 119
UP in U87	**23208**	SYT11	10.047858	0.9983466	1058.538	synaptotagmin XI
UP in U87	**54093**	SETD4	1.0406254	0.8494586	2.057119	SET domain containing 4
UP in U87	**5747**	PTK2	1.5440897	0.9069461	2.9162	protein tyrosine kinase 2
UP in U87	**84292**	WDR83	1.3680601	0.8869451	2.581232	WD repeat domain 83
UP in U87	**3798**	KIF5A	9.4846215	0.9904884	716.4	kinesin family member 5A
UP in U87	**6252**	RTN1	9.5628784	0.9823457	756.3333	reticulon 1
UP in U87	**56927**	GPR108	1.4302734	0.9036482	2.694978	G protein-coupled receptor 108
UP in U87	**81603**	TRIM8	1.2018936	0.8928476	2.300414	tripartite motif containing 8
UP in U87	**78997**	GDAP1L1	7.541742	0.8811225	186.3333	ganglioside induced differentiation associated protein 1-like 1
UP in U87	**6948**	TCN2	2.8453522	0.9182001	7.186813	transcobalamin II
UP in U87	**51099**	ABHD5	1.020699	0.8472185	2.028902	abhydrolase domain containing 5
UP in U87	**4192**	MDK	1.4973778	0.8974168	2.823291	midkine (neurite growth-promoting factor 2)
UP in U87	**9871**	SEC24D	1.5950216	0.9143865	3.02099	SEC24 family, member D (S. cerevisiae)
UP in U87	**814**	CAMK4	4.142212	0.9453482	17.65753	calcium/calmodulin-dependent protein kinase
UP in U87	**387895**	LOC38789	6.9503129	0.8137678	123.6667	uncharacterized LOC387895
UP in U87	**375484**	SIMC1	7.8747618	0.9715274	234.7143	SUMO-interacting motifs containing 1
UP in U87	**84436**	ZNF528	2.8165044	0.9335431	7.044534	zinc finger protein 528
UP in U87	**80700**	UBXN6	1.4952676	0.9073195	2.819164	UBX domain protein 6
UP in U87	**122509**	IFI27L1	1.0612763	0.8829093	2.086777	interferon, alpha-inducible protein 27-like 1
UP in U87	**100750247**	HIF1A-	2.5687186	0.9526197	5.932822	HIF1A antisense RNA 2
UP in U87	**728239**	MAGED4	8.4864999	0.9491795	358.6667	melanoma antigen family D, 4
UP in U87	**79739**	TTLL7	2.857108	0.9143065	7.245614	tubulin tyrosine ligase-like family, member 7
UP in U87	**162417**	NAGS	5.3463433	0.9878127	40.68269	N-acetylglutamate synthase
UP in U87	**256227**	STEAP1B	10.709084	0.9939019	1674	STEAP family member 1B
UP in U87	**8459**	TPST2	1.1714998	0.8805003	2.252457	tyrosylprotein sulfotransferase 2
UP in U87	**6364**	CCL20	7.2087569	0.9790567	147.9286	chemokine (C-C motif) ligand 20
UP in U87	**78990**	OTUB2	2.8522092	0.8556634	7.221053	OTU domain, ubiquitin aldehyde binding 2
UP in U87	**11159**	RABL2A	2.1802	0.8356446	4.532164	RAB, member of RAS oncogene family-like
UP in U87	**8482**	SEMA7A	5.7865964	0.9963998	55.2	semaphorin 7A, GPI membrane anchor (John Milton Hagen blood group)
UP in U87	**8907**	AP1M1	2.1724279	0.9417391	4.507814	adaptor-related protein complex 1, mu 1
UP in U87	**64648**	SPANXD	9.489179	0.9809501	718.6667	SPANX family, member D
UP in U87	**6553**	SLC9A5	2.8836615	0.9234715	7.380208	solute carrier family 9, subfamily A (NHE5, cation proton antiporter 5), member 5
UP in U87	**3706**	ITPKA	1.7877364	0.8902164	3.452727	inositol-trisphosphate 3-kinase A
UP in U87	**29950**	SERTAD1	2.1523061	0.934841	4.445378	SERTA domain containing 1
UP in U87	**11007**	CCDC85B	1.7932683	0.9266272	3.465992	coiled-coil domain containing 85B
UP in U87	**57146**	TMEM159	2.9680205	0.9609046	7.824619	transmembrane protein 159

UP in U87	**170690**	ADAMTS 16	6.7944159	0.8441695	111	ADAM metallopeptidase with thrombospondin type 1 motif, 16
UP in U87	**283624**	LINC0064	1.2012582	0.8002471	2.299401	long intergenic non-protein coding RNA 641
UP in U87	**64641**	EBF2	9.4971865	0.9811457	722.6667	early B-cell factor 2
UP in U87	**1762**	DMWD	1.0711866	0.8698508	2.101161	dystrophia myotonica, WD repeat containing
UP in U87	**9509**	ADAMTS 2	7.3339007	0.8585347	161.3333	ADAM metallopeptidase with thrombospondin type 1 motif, 2
UP in U87	**493**	ATP2B4	2.861773	0.964087	7.269081	ATPase, Ca++ transporting, plasma
UP in U87	**2752**	GLUL	2.300282	0.93717	4.92554	glutamate-ammonia ligase
UP in U87	**6913**	TBX15	8.60807	0.9781855	390.2	T-box 15
UP in U87	**1993**	ELAVL2	3.3817093	0.8382403	10.42308	ELAV (embryonic lethal, abnormal vision, Drosophila)-like 2 (Hu antigen B)
UP in U87	**400**	ARL1	1.0088473	0.8802869	2.012303	ADP-ribosylation factor-like 1
UP in U87	**9832**	JAKMIP2	4.9454438	0.9350099	30.8125	janus kinase and microtubule interacting protein 2
UP in U87	**5533**	PPP3CC	1.6992311	0.9129554	3.247278	protein phosphatase 3, catalytic subunit, gamma isozyme
UP in U87	**103910**	MYL12B	1.2689144	0.9003236	2.409802	myosin, light chain 12B, regulatory
UP in U87	**54432**	YIPF1	1.7455994	0.9179778	3.353342	Yip1 domain family, member 1
UP in U87	**100874362**	HOXB-	7.946419	0.9163955	246.6667	HOXB cluster antisense RNA 1
UP in U87	**5266**	PI3	8.8757494	0.9996355	469.75	peptidase inhibitor 3, skin-derived
UP in U87	**257194**	NEGR1	5.5130696	0.8261952	45.66667	neuronal growth regulator 1
UP in U87	**221421**	RSPH9	4.9825549	0.8229683	31.61538	radial spoke head 9 homolog
UP in U87	**4854**	NOTCH3	5.4627068	0.8378491	44.1	notch 3
UP in U87	**25796**	PGLS	1.0672303	0.8880118	2.095407	6-phosphogluconolactonase
UP in U87	**129642**	MBOAT2	2.2353083	0.931463	4.708633	membrane bound O-acyltransferase domain containing 2
UP in U87	**8444**	DYRK3	2.746749	0.953642	6.712029	dual-specificity tyrosine-(Y)-phosphorylation regulated kinase 3
UP in U87	**285464**	CRIPAK	1.918654	0.8697442	3.780702	cysteine-rich PAK1 inhibitor
UP in U87	**89781**	HPS4	1.0511483	0.8615482	2.072178	Hermansky-Pudlak syndrome 4
UP in U87	**23529**	CLCF1	1.6363805	0.9102708	3.108849	cardiotrophin-like cytokine factor 1
UP in U87	**5524**	PPP2R4	1.2115667	0.89627	2.31589	protein phosphatase 2A activator, regulatory subunit 4
UP in U87	**65249**	ZSWIM4	1.8520804	0.888803	3.610204	zinc finger, SWIM-type containing 4
UP in U87	**25900**	IFFO1	1.4369458	0.8980034	2.707471	intermediate filament family orphan 1
UP in U87	**80331**	DNAJC5	1.7052944	0.9234626	3.260955	DnaJ (Hsp40) homolog, subfamily C,
UP in U87	**22839**	DLGAP4	2.0335458	0.9378456	4.094098	discs, large (Drosophila) homolog-associated protein 4
UP in U87	**7102**	TSPAN7	6.3439544	0.9456149	81.23077	tetraspanin 7
UP in U87	**51635**	DHRS7	2.2746562	0.9413124	4.838823	dehydrogenase/reductase (SDR family)
UP in U87	**9948**	WDR1	1.5033833	0.9084929	2.835068	WD repeat domain 1
UP in U87	**7436**	VLDLR	1.4908612	0.8953189	2.810567	very low density lipoprotein receptor
UP in U87	**135398**	C6orf141	8.8626374	0.9908706	465.5	chromosome 6 open reading frame 141
UP in U87	**162394**	SLFN5	4.5004822	0.989235	22.63498	schlafen family member 5
UP in U87	**113157**	RPLP0P2	4.6766623	0.9359166	25.575	ribosomal protein, large, P0 pseudogene 2
UP in U87	**79867**	TCTN2	2.309719	0.9304496	4.957865	tectonic family member 2
UP in U87	**440356**	LOC44035	3.7422599	0.8198748	13.38235	uncharacterized LOC440356
UP in U87	**9372**	ZFYVE9	1.7138741	0.9051416	3.280405	zinc finger, FYVE domain containing 9
UP in U87	**602**	BCL3	1.7098519	0.9205024	3.271272	B-cell CLL/lymphoma 3
UP in U87	**214**	ALCAM	1.000744	0.8749889	2.001032	activated leukocyte cell adhesion molecule
UP in U87	**254428**	SLC41A1	1.0966191	0.8831404	2.13853	solute carrier family 41, member 1
UP in U87	**196051**	PPAPDC1 A	10.426614	0.9919907	1376.333	phosphatidic acid phosphatase type 2 domain containing 1A
UP in U87	**55332**	DRAM1	1.297902	0.8880918	2.458711	DNA-damage regulated autophagy modulator
UP in U87	**4502**	MT2A	1.9806491	0.9301741	3.946706	metallothionein 2A
UP in U87	**51733**	UPB1	7.1632303	0.8386492	143.3333	ureidopropionase, beta

UP in U87	**7347**	UCHL3	1.0214448	0.8840205	2.029951	ubiquitin carboxyl-terminal esterase L3 (ubiquitin thiolesterase)
UP in U87	**2152**	F3	3.7526388	0.9805767	13.47897	coagulation factor III (thromboplastin, tissue factor)
UP in U87	**128338**	DRAM2	1.1223206	0.8801803	2.176969	DNA-damage regulated autophagy modulator
UP in U87	**8935**	SKAP2	3.660844	0.9744431	12.64806	src kinase associated phosphoprotein 2
UP in U87	**9379**	NRXN2	8.8677935	0.9867282	467.1667	neurexin 2
UP in U87	**2934**	GSN	1.6866503	0.923587	3.219084	gelsolin
UP in U87	**51668**	HSPB11	1.0411342	0.8862073	2.057845	heat shock protein family B (small), member
UP in U87	**9114**	ATP6V0D 1	1.0708325	0.8881096	2.100645	ATPase, H+ transporting, lysosomal 38kDa, V0 subunit d1
UP in U87	**55704**	CCDC88A	1.2643239	0.8869451	2.402146	coiled-coil domain containing 88A
UP in U87	**51171**	HSD17B14	2.2610861	0.9204046	4.793522	hydroxysteroid (17-beta) dehydrogenase 14
UP in U87	**6857**	SYT1	7.5018372	0.9135687	181.25	synaptotagmin I
UP in U87	**80127**	CCDC176	3.0783162	0.9051061	8.446281	coiled-coil domain containing 176
UP in U87	**1307**	COL16A1	4.1596799	0.9830391	17.87263	collagen, type XVI, alpha 1
UP in U87	**100131897**	FAM196B	9.7188998	0.994942	842.7143	family with sequence similarity 196, member
UP in U87	**100820829**	MYZAP	6.8926118	0.9600334	118.8182	myocardial zonula adherens protein
UP in U87	**84069**	PLEKHN1	3.6286718	0.9541753	12.36913	pleckstrin homology domain containing, family N member 1
UP in U87	**728712**	SPANXA2	11.298063	0.996222	2518	SPANX family, member A2
UP in U87	**23135**	KDM6B	1.0250488	0.8255996	2.035028	lysine (K)-specific demethylase 6B
UP in U87	**5138**	PDE2A	6.5736472	0.8172969	95.25	phosphodiesterase 2A, cGMP-stimulated
UP in U87	**8897**	MTMR3	1.3281113	0.8751311	2.510738	myotubularin related protein 3
UP in U87	**7693**	ZNF134	1.443116	0.8749178	2.719075	zinc finger protein 134
UP in U87	**85352**	KIAA1644	1.584237	0.9175512	2.998492	KIAA1644
UP in U87	**118**	ADD1	1.3580943	0.9018881	2.563463	adducin 1 (alpha)
UP in U87	**10157**	AASS	1.6748576	0.8417249	3.192878	aminoadipate-semialdehyde synthase
UP in U87	**116071**	BATF2	5.3186455	0.836329	39.90909	basic leucine zipper transcription factor, ATF-like 2
UP in U87	**114907**	FBXO32	4.2350821	0.9620335	18.83158	F-box protein 32
UP in U87	**140739**	UBE2F	1.2338686	0.8913009	2.351968	ubiquitin-conjugating enzyme E2F (putative)
UP in U87	**5797**	PTPRM	1.8125538	0.9082618	3.512635	protein tyrosine phosphatase, receptor type,
UP in U87	**79971**	WLS	1.4461386	0.9057728	2.724778	wntless homolog (Drosophila)
UP in U87	**5228**	PGF	2.2753577	0.8464007	4.841176	placental growth factor
UP in U87	**8635**	RNASET2	1.8441002	0.9165467	3.59029	ribonuclease T2
UP in U87	**29967**	LRP12	1.757294	0.9139065	3.380634	low density lipoprotein receptor-related protein 12
UP in U87	**7110**	TMF1	1.6738425	0.9107241	3.190633	TATA element modulatory factor 1
UP in U87	**715**	C1R	4.1289297	0.9855281	17.49571	complement component 1, r subcomponent
UP in U87	**2201**	FBN2	7.4911243	0.9781233	179.9091	fibrillin 2
UP in U87	**3931**	LCAT	2.0421695	0.8783668	4.118644	lecithin-cholesterol acyltransferase
UP in U87	**4692**	NDN	1.833613	0.9091685	3.564286	necdin, melanoma antigen (MAGE) family member
UP in U87	**79931**	TNIP3	7.6653359	0.8932565	203	TNFAIP3 interacting protein 3
UP in U87	**728113**	ANXA8L1	1.3068005	0.8044073	2.473923	annexin A8-like 1
UP in U87	**5289**	PIK3C3	1.6262718	0.9138621	3.087142	phosphatidylinositol 3-kinase, catalytic subunit type 3
UP in U87	**64760**	FAM160B	1.4575629	0.8964834	2.74644	family with sequence similarity 160, member
UP in U87	**51299**	NRN1	8.1137422	0.9778477	277	neuritin 1
UP in U87	**6382**	SDC1	2.0891731	0.9406546	4.255041	syndecan 1
UP in U87	**11221**	DUSP10	3.249756	0.9601223	9.512048	dual specificity phosphatase 10
UP in U87	**219333**	USP12	1.4364295	0.9000924	2.706502	ubiquitin specific peptidase 12
UP in U87	**50651**	SLC45A1	4.0370893	0.9039149	16.41667	solute carrier family 45, member 1
UP in U87	**8675**	STX16	1.1242394	0.8897097	2.179866	syntaxin 16
UP in U87	**100505881**	MAGI2-	8.1969932	0.9887727	293.4545	MAGI2 antisense RNA 3
UP in U87	**9895**	TECPR2	1.0534139	0.8417427	2.075435	tectonin beta-propeller repeat containing 2

UP in U87	**728621**	CCDC30	2.9733051	0.8350756	7.853333	coiled-coil domain containing 30
UP in U87	**9482**	STX8	1.3809237	0.8991235	2.604351	syntaxin 8
UP in U87	**590**	BCHE	7.456149	0.9738919	175.6	butyrylcholinesterase
UP in U87	**27177**	IL36B	10.490851	0.9924529	1439	interleukin 36, beta
UP in U87	**23057**	NMNAT2	7.5387278	0.9890661	185.9444	nicotinamide nucleotide adenylyltransferase
UP in U87	**11151**	CORO1A	3.1043367	0.8639216	8.6	coronin, actin binding protein, 1A
UP in U87	**51108**	METTL9	1.5835761	0.9221381	2.997118	methyltransferase like 9
UP in U87	**1362**	CPD	1.7753659	0.9221114	3.423248	carboxypeptidase D
UP in U87	**255919**	CNEP1R1	1.2371859	0.8712554	2.357383	CTD nuclear envelope phosphatase 1 regulatory subunit 1
UP in U87	**83544**	DNAL1	1.4285174	0.8378936	2.6917	dynein, axonemal, light chain 1
UP in U87	**2184**	FAH	2.0419073	0.938699	4.117896	fumarylacetoacetate hydrolase (fumarylacetoacetase)
UP in U87	**56900**	TMEM167	2.2580409	0.9396501	4.783415	transmembrane protein 167B
UP in U87	**5465**	PPARA	1.180067	0.8380358	2.265873	peroxisome proliferator-activated receptor
UP in U87	**7849**	PAX8	2.8681498	0.9083951	7.301282	paired box 8
UP in U87	**5565**	PRKAB2	1.0037029	0.862446	2.00514	protein kinase, AMP-activated, beta 2 non-catalytic subunit
UP in U87	**80228**	ORAI2	1.096888	0.8639572	2.138928	ORAI calcium release-activated calcium modulator 2
UP in U87	**192670**	AGO4	1.8126794	0.8770157	3.512941	argonaute RISC catalytic component 4
UP in U87	**3097**	HIVEP2	1.5320136	0.8523299	2.891892	human immunodeficiency virus type I enhancer binding protein 2
UP in U87	**55884**	WSB2	2.5609945	0.9527708	5.901143	WD repeat and SOCS box containing 2
UP in U87	**780776**	TVP23A	3.1246957	0.8056874	8.722222	trans-golgi network vesicle protein 23 homolog A (S. cerevisiae)
UP in U87	**55589**	BMP2K	1.6735144	0.8969723	3.189907	BMP2 inducible kinase
UP in U87	**11010**	GLIPR1	3.9556186	0.9835813	15.51529	GLI pathogenesis-related 1
UP in U87	**2771**	GNAI2	1.0075602	0.8873629	2.010508	guanine nucleotide binding protein (G protein), alpha inhibiting activity polypeptide 2
UP in U87	**54885**	TBC1D8B	7.8037724	0.9787722	223.4444	TBC1 domain family, member 8B (with GRAM domain)
UP in U87	**1635**	DCTD	1.0361918	0.8805892	2.050807	dCMP deaminase
UP in U87	**8382**	NME5	8.3143944	0.9400679	318.3333	NME/NM23 family member 5
UP in U87	**55329**	MNS1	1.0151349	0.815768	2.021092	meiosis-specific nuclear structural 1
UP in U87	**80139**	ZNF703	2.2516054	0.9150621	4.762125	zinc finger protein 703
UP in U87	**84709**	MGARP	5.6170633	0.9538642	49.08	mitochondria-localized glutamic acid-rich
UP in U87	**3778**	KCNMA1	6.7456343	0.9969421	107.3095	potassium large conductance calcium-activated channel, subfamily M, alpha member 1
UP in U87	**100128890**	FAM66B	8.7347096	0.9600512	426	family with sequence similarity 66, member
UP in U87	**1051**	CEBPB	2.5818517	0.9595178	5.987077	CCAAT/enhancer binding protein (C/EBP),
UP in U87	**384**	ARG2	1.0299015	0.8640639	2.041885	arginase, type II
UP in U87	**4052**	LTBP1	2.1138778	0.9404324	4.328532	latent transforming growth factor beta binding protein 1
UP in U87	**286411**	RP1-	5.2384047	0.8850339	37.75	uncharacterized LOC286411
UP in U87	**79042**	TSEN34	1.0550527	0.8843049	2.077794	tRNA splicing endonuclease 34 homolog (S. cerevisiae)
UP in U87	**5971**	RELB	4.3604686	0.9828524	20.54148	v-rel reticuloendotheliosis viral oncogene homolog B
UP in U87	**7268**	TTC4	1.169661	0.8908653	2.249588	tetratricopeptide repeat domain 4
UP in U87	**65981**	CAPRIN2	2.677914	0.9484239	6.399299	caprin family member 2
UP in U87	**55663**	ZNF446	1.4619447	0.8681707	2.754795	zinc finger protein 446
UP in U87	**285613**	RELL2	2.4396231	0.9385834	5.425	RELT-like 2
UP in U87	**51594**	NBAS	1.094856	0.8749356	2.135918	neuroblastoma amplified sequence
UP in U87	**3702**	ITK	7.9425145	0.9158888	246	IL2-inducible T-cell kinase

UP in U87	**11245**	GPR176	1.8981474	0.9194535	3.727343	G protein-coupled receptor 176
UP in U87	**79710**	MORC4	1.9568036	0.9143332	3.882009	MORC family CW-type zinc finger 4
UP in U87	**7474**	WNT5A	1.2912534	0.8982612	2.447406	wingless-type MMTV integration site family, member 5A
UP in U87	**134**	ADORA1	2.9537916	0.8875762	7.747826	adenosine A1 receptor
UP in U87	**3290**	HSD11B1	6.8703647	0.8024695	117	hydroxysteroid (11-beta) dehydrogenase 1
UP in U87	**4131**	MAP1B	2.0940221	0.9408324	4.269367	microtubule-associated protein 1B
UP in U87	**185**	AGTR1	7.1931146	0.8422938	146.3333	angiotensin II receptor, type 1
UP in U87	**100528022**	BLOC1S1-RDH5	1.8523023	0.8562146	3.610759	BLOC1S1-RDH5 readthrough
UP in U87	**10618**	TGOLN2	1.0508999	0.8874162	2.071822	trans-golgi network protein 2
UP in U87	**3588**	IL10RB	1.4788918	0.8988657	2.787345	interleukin 10 receptor, beta
UP in U87	**57403**	RAB22A	1.7180815	0.9152133	3.289986	RAB22A, member RAS oncogene family
UP in U87	**10401**	PIAS3	1.0650013	0.8808381	2.092172	protein inhibitor of activated STAT, 3
UP in U87	**23164**	MPRIP	1.4703735	0.9063328	2.770936	myosin phosphatase Rho interacting protein
UP in U87	**23643**	LY96	10.921097	0.9948086	1939	lymphocyte antigen 96
UP in U87	**8671**	SLC4A4	7.6911619	0.8958433	206.6667	solute carrier family 4, sodium bicarbonate cotransporter, member 4
UP in U87	**51646**	YPEL5	2.8897101	0.9622913	7.411215	yippee-like 5 (Drosophila)
UP in U87	**100506581**	C16orf95	1.6680182	0.8476097	3.177778	chromosome 16 open reading frame 95
UP in U87	**10402**	ST3GAL6	7.0134623	0.8999502	129.2	ST3 beta-galactoside alpha-2,3-
UP in U87	**3241**	HPCAL1	2.6465665	0.9596067	6.261753	hippocalcin-like 1
UP in U87	**55752**	11-Sep	1.0721539	0.8878251	2.10257	septin 11
UP in U87	**8767**	RIPK2	2.2369902	0.9386723	4.714126	receptor-interacting serine-threonine kinase 2
UP in U87	**399665**	FAM102A	1.717452	0.9226181	3.288551	family with sequence similarity 102, member
UP in U87	**54795**	TRPM4	3.4244978	0.9485661	10.73684	transient receptor potential cation channel, subfamily M, member 4
UP in U87	**301**	ANXA1	2.3627771	0.9520597	5.143595	annexin A1
UP in U87	**56129**	PCDHB7	7.3219281	0.8574324	160	protocadherin beta 7
UP in U87	**84875**	PARP10	1.5347957	0.8698864	2.897474	poly (ADP-ribose) polymerase family,
UP in U87	**2258**	FGF13	9.3353904	0.9781233	646	fibroblast growth factor 13
UP in U87	**10565**	ARFGEF1	1.217058	0.8870251	2.324722	ADP-ribosylation factor guanine nucleotide-exchange factor 1 (brefeldin A-inhibited)
UP in U87	**64968**	MRPS6	1.1429741	0.8912209	2.208358	mitochondrial ribosomal protein S6
UP in U87	**6703**	SPRR2D	9.9929383	0.9882572	1019	small proline-rich protein 2D
UP in U87	**1161**	ERCC8	1.7173549	0.9049816	3.28833	excision repair cross-complementing rodent repair deficiency, complementation group 8
UP in U87	**100506844**	LOC100506844	1.8390129	0.9146799	3.577652	uncharacterized LOC100506844
UP in U87	**285671**	RNF180	7.169925	0.8397603	144	ring finger protein 180
UP in U87	**126298**	IRGQ	1.3285127	0.8759045	2.511436	immunity-related GTPase family, Q
UP in U87	**55711**	FAR2	4.8515476	0.9839369	28.87097	fatty acyl CoA reductase 2
UP in U87	**84284**	NTPCR	1.1729706	0.8925632	2.254755	nucleoside-triphosphatase, cancer-related
UP in U87	**118611**	C10orf90	7.9731848	0.9742653	251.2857	chromosome 10 open reading frame 90
UP in U87	**23200**	ATP11B	1.1310741	0.8631305	2.190217	ATPase, class VI, type 11B
UP in U87	**112942**	CCDC104	2.2898815	0.9411702	4.890159	coiled-coil domain containing 104
UP in U87	**128866**	CHMP4B	1.325071	0.9029193	2.505452	charged multivesicular body protein 4B
UP in U87	**79415**	C17orf62	1.0781852	0.8845627	2.111378	chromosome 17 open reading frame 62
UP in U87	**23765**	IL17RA	1.1557411	0.8181325	2.227987	interleukin 17 receptor A
UP in U87	**2650**	GCNT1	3.531934	0.9413924	11.56693	glucosaminyl (N-acetyl) transferase 1, core 2
UP in U87	**9454**	HOMER3	4.2203658	0.9884083	18.64046	homer homolog 3 (Drosophila)
UP in U87	**9536**	PTGES	2.4527994	0.9523975	5.474774	prostaglandin E synthase
UP in U87	**54677**	CROT	7.0139242	0.9900795	129.2414	carnitine O-octanoyltransferase
UP in U87	**51312**	SLC25A37	1.6418224	0.9191335	3.120598	solute carrier family 25 (mitochondrial iron transporter), member 37
UP in U87	**55359**	STYK1	3.2453068	0.8364091	9.482759	serine/threonine/tyrosine kinase 1
UP in U87	**55901**	THSD1	4.7755188	0.8506587	27.38889	thrombospondin, type I, domain containing 1

UP in U87	**80008**	TMEM156	6.6384359	0.9899817	99.625	transmembrane protein 156
UP in U87	**79583**	TMEM231	1.2378809	0.8370224	2.358519	transmembrane protein 231
UP in U87	**84824**	FCRLA	8.9801396	0.9686294	505	Fc receptor-like A
UP in U87	**665**	BNIP3L	2.4308491	0.9512685	5.392107	BCL2/adenovirus E1B 19kDa interacting protein 3-like
UP in U87	**153769**	SH3RF2	2.585568	0.9387256	6.002519	SH3 domain containing ring finger 2
UP in U87	**51088**	KLHL5	1.7726307	0.9222092	3.416764	kelch-like family member 5
UP in U87	**79365**	BHLHE41	8.2644426	0.9906395	307.5	basic helix-loop-helix family, member e41
UP in U87	**375449**	MAST4	3.5104055	0.9183779	11.3956	microtubule associated serine/threonine kinase family member 4
UP in U87	**84902**	CEP89	2.4276463	0.9357744	5.38015	centrosomal protein 89kDa
UP in U87	**1462**	VCAN	11.834339	0.9976177	3651.667	versican
UP in U87	**654433**	LOC65443	3.5249211	0.9763187	11.51084	uncharacterized LOC654433
UP in U87	**23412**	COMMD3	1.4264784	0.9014614	2.687898	COMM domain containing 3
UP in U87	**220929**	ZNF438	2.1759051	0.8614504	4.518692	zinc finger protein 438
UP in U87	**50614**	GALNT9	12.302734	0.9985866	5052.333	UDP-N-acetyl-alpha-D-galactosamine:polypeptide N-acetylgalactosaminyltransferase 9 (GalNAc-T9)
UP in U87	**285203**	EOGT	2.0271623	0.9211514	4.076023	EGF domain-specific O-linked N-acetylglucosamine (GlcNAc) transferase
UP in U87	**728047**	GOLGA8O	2.9432043	0.816808	7.691176	golgin A8 family, member O
UP in U87	**83854**	ANGPTL6	3.3457748	0.8971412	10.16667	angiopoietin-like 6
UP in U87	**1827**	RCAN1	1.8265433	0.9234537	3.546862	regulator of calcineurin 1
UP in U87	**144455**	E2F7	1.5048268	0.9035949	2.837906	E2F transcription factor 7
UP in U87	**54210**	TREM1	6.8948178	0.9739897	119	triggering receptor expressed on myeloid
UP in U87	**79800**	ALS2CR8	2.2171175	0.8088876	4.649635	amyotrophic lateral sclerosis 2 (juvenile) chromosome region, candidate 8
UP in U87	**203859**	ANO5	7.2946207	0.8967767	157	anoctamin 5
UP in U87	**56895**	AGPAT4	1.41884	0.8397426	2.673704	1-acylglycerol-3-phosphate O-acyltransferase
UP in U87	**153642**	ARSK	1.3554178	0.8318044	2.558712	arylsulfatase family, member K
UP in U87	**84752**	B3GNT9	2.4037027	0.946166	5.291595	UDP-GlcNAc:betaGal beta-1,3-N-acetylglucosaminyltransferase 9
UP in U87	**90203**	SNX21	1.3787614	0.8922076	2.60045	sorting nexin family member 21
UP in U87	**79890**	RIN3	4.4284998	0.980639	21.53333	Ras and Rab interactor 3
UP in U87	**64853**	AIDA	1.2336422	0.8975056	2.351599	axin interactor, dorsalization associated
UP in U87	**83874**	TBC1D10	3.019956	0.963278	8.111429	TBC1 domain family, member 10A
UP in U87	**116115**	ZNF526	1.1520763	0.8524366	2.222335	zinc finger protein 526
UP in U87	**196740**	VSTM4	2.3247915	0.909533	5.009934	V-set and transmembrane domain containing
UP in U87	**3988**	LIPA	1.0442012	0.8824826	2.062224	lipase A, lysosomal acid, cholesterol esterase
UP in U87	**56122**	PCDHB14	4.3677318	0.8844027	20.64516	protocadherin beta 14
UP in U87	**5617**	PRL	6.2114016	0.9138354	74.1	prolactin
UP in U87	**57134**	MAN1C1	6.8808954	0.9258627	117.8571	mannosidase, alpha, class 1C, member 1
UP in U87	**1613**	DAPK3	1.5350002	0.9071506	2.897885	death-associated protein kinase 3
UP in U87	**57520**	HECW2	2.0413408	0.8175103	4.116279	HECT, C2 and WW domain containing E3 ubiquitin protein ligase 2
UP in U87	**9048**	ARTN	3.993545	0.8215638	15.92857	artemin
UP in U87	**6094**	ROM1	1.2714987	0.818097	2.414122	retinal outer segment membrane protein 1
UP in U87	**3196**	TLX2	7.1943092	0.9702295	146.4545	T-cell leukemia homeobox 2
UP in U87	**7046**	TGFBR1	2.4471344	0.9490995	5.453318	transforming growth factor, beta receptor 1
UP in U87	**2048**	EPHB2	2.5930721	0.9524686	6.033822	EPH receptor B2
UP in U87	**92979**	9-Mar	1.8897128	0.9042349	3.705615	membrane-associated ring finger (C3HC4) 9
UP in U87	**57185**	NIPAL3	1.3588853	0.8918165	2.564869	NIPA-like domain containing 3
UP in U87	**117584**	RFFL	1.3874689	0.8910342	2.616193	ring finger and FYVE-like domain containing E3 ubiquitin protein ligase

UP in U87	**10962**	MLLT11	5.6468522	0.9950842	50.10394	myeloid/lymphoid or mixed-lineage leukemia (trithorax homolog, Drosophila); translocated to, 11
UP in U87	**4638**	MYLK	6.2036456	0.994373	73.7027	myosin light chain kinase
UP in U87	**6662**	SOX9	1.2500778	0.807812	2.378543	SRY (sex determining region Y)-box 9
UP in U87	**548593**	SLX1A	1.2973221	0.896999	2.457723	SLX1 structure-specific endonuclease subunit homolog A (S. cerevisiae)
UP in U87	**64319**	FBRS	1.0404938	0.8836738	2.056932	fibrosin
UP in U87	**100506714**	LOC100506714	1.4615801	0.8633794	2.754098	uncharacterized LOC100506714
UP in U87	**23549**	DNPEP	1.043104	0.8830515	2.060656	aspartyl aminopeptidase
UP in U87	**375323**	LHFPL4	6.033423	0.8678063	65.5	lipoma HMGIC fusion partner-like 4
UP in U87	**126433**	FBXO27	2.7203393	0.949224	6.590278	F-box protein 27
UP in U87	**284677**	C1orf204	4.8110306	0.8128078	28.07143	chromosome 1 open reading frame 204
UP in U87	**1032**	CDKN2D	3.122004	0.9641314	8.705964	cyclin-dependent kinase inhibitor 2D (p19, inhibits CDK4)
UP in U87	**65251**	ZNF649	6.5637683	0.8533522	94.6	zinc finger protein 649
UP in U87	**51177**	PLEKHO1	4.3304233	0.9896172	20.11812	pleckstrin homology domain containing, family O member 1
UP in U87	**146880**	LOC14688	1.5791317	0.9060483	2.9879	Rho GTPase activating protein 27
UP in U87	**197320**	ZNF778	1.0700517	0.8258307	2.099509	zinc finger protein 778
UP in U87	**54625**	PARP14	1.0567319	0.8160346	2.080214	poly (ADP-ribose) polymerase family,
UP in U87	**26262**	TSPAN17	1.4309011	0.9019148	2.696151	tetraspanin 17
UP in U87	**84513**	PPAPDC1B	2.1967287	0.9361299	4.584387	phosphatidic acid phosphatase type 2 domain containing 1B
UP in U87	**3383**	ICAM1	1.6237992	0.9167067	3.081855	intercellular adhesion molecule 1
UP in U87	**375295**	LOC37529	2.6342847	0.9511885	6.208672	uncharacterized LOC375295
UP in U87	**23179**	RGL1	2.6059475	0.8978346	6.087912	ral guanine nucleotide dissociation stimulator-like 1
UP in U87	**79735**	TBC1D17	1.805851	0.9232315	3.496353	TBC1 domain family, member 17
UP in U87	**5051**	PAFAH2	1.1133356	0.8527121	2.163453	platelet-activating factor acetylhydrolase 2, 40kDa
UP in U87	**8974**	P4HA2	2.9925552	0.9652604	7.958824	prolyl 4-hydroxylase, alpha polypeptide II
UP in U87	**257396**	LOC25739	2.4009623	0.8862428	5.281553	uncharacterized LOC257396
UP in U87	**6253**	RTN2	2.0319663	0.9309208	4.089618	reticulon 2
UP in U87	**28512**	NKIRAS1	1.1537804	0.8660284	2.224961	NFKB inhibitor interacting Ras-like 1
UP in U87	**8871**	SYNJ2	1.935266	0.9251693	3.824486	synaptojanin 2
UP in U87	**4907**	NT5E	8.215678	0.9997067	297.2799	5'-nucleotidase, ecto (CD73)
UP in U87	**54039**	PCBP3	7.6510517	0.9704873	201	poly(rC) binding protein 3
UP in U87	**9455**	HOMER2	4.4126751	0.9822302	21.29843	homer homolog 2 (Drosophila)
UP in U87	**8497**	PPFIA4	2.8224302	0.8930165	7.073529	protein tyrosine phosphatase, receptor type, f polypeptide (PTPRF), interacting protein (liprin), alpha 4
UP in U87	**8439**	NSMAF	1.0190845	0.8798336	2.026633	neutral sphingomyelinase (N-SMase) activation associated factor
UP in U87	**9936**	CD302	4.4357397	0.9753054	21.64167	CD302 molecule
UP in U87	**80704**	SLC19A3	6.1085245	0.8558945	69	solute carrier family 19, member 3
UP in U87	**80301**	PLEKHO2	1.5771412	0.9101641	2.98378	pleckstrin homology domain containing, family O member 2
UP in U87	**7185**	TRAF1	4.1305316	0.9380856	17.51515	TNF receptor-associated factor 1
UP in U87	**57763**	ANKRA2	1.8378696	0.8937899	3.574818	ankyrin repeat, family A (RFXANK-like), 2
UP in U87	**967**	CD63	1.9750704	0.9301741	3.931474	CD63 molecule
UP in U87	**9289**	GPR56	3.4264284	0.9759098	10.75122	G protein-coupled receptor 56
UP in U87	**9853**	RUSC2	1.9508052	0.9245204	3.865902	RUN and SH3 domain containing 2
UP in U87	**322**	APBB1	3.2150129	0.9683094	9.285714	amyloid beta (A4) precursor protein-binding, family B, member 1 (Fe65)
UP in U87	**960**	CD44	3.1013339	0.9695006	8.582119	CD44 molecule (Indian blood group)

UP in U87	**84668**	FAM126A	2.1304375	0.9380145	4.378503	family with sequence similarity 126, member
UP in U87	**55012**	PPP2R3C	1.203545	0.8783491	2.303049	protein phosphatase 2, regulatory subunit B", gamma
UP in U87	**100506394**	LOC100506394	2.2858588	0.8417871	4.876543	uncharacterized LOC100506394
UP in U87	**10395**	DLC1	3.1785461	0.9645048	9.053942	deleted in liver cancer 1
UP in U87	**441531**	PGAM4	6.4274625	0.981759	86.07143	phosphoglycerate mutase family member 4
UP in U87	**7538**	ZFP36	2.1857265	0.9331964	4.549558	ZFP36 ring finger protein
UP in U87	**132160**	PPM1M	1.2087594	0.8655217	2.311388	protein phosphatase, Mg2+/Mn2+ dependent, 1M
UP in U87	**157807**	CLVS1	1.3597849	0.8809892	2.566469	clavesin 1
UP in U87	**55773**	TBC1D23	1.421237	0.8974168	2.67815	TBC1 domain family, member 23
UP in U87	**51754**	TMEM8B	3.9732669	0.9687272	15.70625	transmembrane protein 8B
UP in U87	**1948**	EFNB2	2.1883341	0.9081995	4.557789	ephrin-B2
UP in U87	**89796**	NAV1	1.3053643	0.8870784	2.471461	neuron navigator 1
UP in U87	**2596**	GAP43	9.5287797	0.9817235	738.6667	growth associated protein 43
UP in U87	**57669**	EPB41L5	2.1192989	0.8836827	4.344828	erythrocyte membrane protein band 4.1 like 5
UP in U87	**202181**	LOC202181	5.129283	0.8965278	35	SUMO-interacting motifs containing 1 pseudogene
UP in U87	**2643**	GCH1	1.8621432	0.9124664	3.635473	GTP cyclohydrolase 1
UP in U87	**6508**	SLC4A3	4.0512089	0.9305563	16.57813	solute carrier family 4, anion exchanger,
UP in U87	**26010**	SPATS2L	1.4104168	0.8960389	2.65814	spermatogenesis associated, serine-rich 2-like
UP in U87	**57192**	MCOLN1	1.1313888	0.8768201	2.190695	mucolipin 1
UP in U87	**10468**	FST	2.5564824	0.8772023	5.882716	follistatin
UP in U87	**11162**	NUDT6	2.2158334	0.935801	4.645498	nudix (nucleoside diphosphate linked moiety X)-type motif 6
UP in U87	**160897**	GPR180	1.1189683	0.8285064	2.171916	G protein-coupled receptor 180
UP in U87	**23008**	KLHDC10	1.3726428	0.8977279	2.589445	kelch domain containing 10
UP in U87	**1384**	CRAT	3.831966	0.9824435	14.24088	carnitine O-acetyltransferase
UP in U87	**219771**	CCNY	1.2287851	0.8907853	2.343695	cyclin Y
UP in U87	**126119**	JOSD2	2.6020188	0.9570822	6.071356	Josephin domain containing 2
UP in U87	**132864**	CPEB2	2.3327598	0.9167333	5.037681	cytoplasmic polyadenylation element binding protein 2
UP in U87	**83999**	KREMEN	1.7977014	0.906475	3.476658	kringle containing transmembrane protein 1
UP in U87	**27247**	NFU1	1.4289123	0.9017459	2.692437	NFU1 iron-sulfur cluster scaffold homolog (S. cerevisiae)
UP in U87	**201191**	SAMD14	7.2889548	0.9785944	156.3846	sterile alpha motif domain containing 14
UP in U87	**79600**	TCTN1	1.5636735	0.8862784	2.956056	tectonic family member 1
UP in U87	**64215**	DNAJC1	1.1482391	0.8806247	2.216432	DnaJ (Hsp40) homolog, subfamily C,
UP in U87	**8942**	KYNU	2.3363077	0.9518285	5.050085	kynureninase
UP in U87	**652968**	GATSL3	3.8482412	0.9349565	14.40244	GATS protein-like 3
UP in U87	**6560**	SLC12A4	2.3257158	0.9435614	5.013145	solute carrier family 12 (potassium/chloride transporters), member 4
UP in U87	**7571**	ZNF23	2.7624182	0.9410813	6.785326	zinc finger protein 23
UP in U87	**171022**	LINC0003	7.033423	0.8236884	131	long intergenic non-protein coding RNA 35
UP in U87	**9242**	MSC	2.0950202	0.9324408	4.272321	musculin
UP in U87	**199745**	THAP8	1.9586731	0.8629082	3.887043	THAP domain containing 8
UP in U87	**149473**	CCDC24	2.2396953	0.9153466	4.722973	coiled-coil domain containing 24
UP in U87	**83648**	FAM167A	4.0589533	0.9828791	16.66736	family with sequence similarity 167, member
UP in U87	**4121**	MAN1A1	8.2827796	0.9968532	311.4333	mannosidase, alpha, class 1A, member 1
UP in U87	**84243**	ZDHHC18	1.251744	0.884865	2.381291	zinc finger, DHHC-type containing 18
UP in U87	**56675**	NRIP3	5.7418231	0.9953598	53.51321	nuclear receptor interacting protein 3
UP in U87	**51429**	SNX9	1.7967911	0.9247693	3.474466	sorting nexin 9
UP in U87	**54918**	CMTM6	1.2607901	0.8955589	2.396269	CKLF-like MARVEL transmembrane domain containing 6
UP in U87	**29775**	CARD10	2.4622405	0.943437	5.510719	caspase recruitment domain family, member

UP in U87	**30008**	EFEMP2	5.6705753	0.9931463	50.93464	EGF containing fibulin-like extracellular matrix protein 2
UP in U87	**8774**	NAPG	1.570492	0.8978346	2.97006	N-ethylmaleimide-sensitive factor attachment protein, gamma
UP in U87	**10421**	CD2BP2	1.2699654	0.8971412	2.411558	CD2 (cytoplasmic tail) binding protein 2
UP in U87	**205251**	LINC0011	1.0052177	0.852481	2.007246	long intergenic non-protein coding RNA 116
UP in U87	**9447**	AIM2	9.4864999	0.980879	717.3333	absent in melanoma 2
UP in U87	**80216**	ALPK1	2.774565	0.831591	6.842697	alpha-kinase 1
UP in U87	**11309**	SLCO2B1	6.606273	0.9049194	97.42857	solute carrier organic anion transporter family, member 2B1
UP in U87	**130814**	PQLC3	2.79477	0.9474016	6.939203	PQ loop repeat containing 3
UP in U87	**3667**	IRS1	1.3545246	0.8923943	2.557128	insulin receptor substrate 1
UP in U87	**283651**	HMGN2P4 6	4.7086369	0.9004214	26.14815	high mobility group nucleosomal binding domain 2 pseudogene 46
UP in U87	**89853**	MVB12B	5.1779178	0.9071417	36.2	multivesicular body subunit 12B
UP in U87	**64755**	C16orf58	1.7985365	0.9241826	3.478672	chromosome 16 open reading frame 58
UP in U87	**127943**	FCRLB	5.4329594	0.9558288	43.2	Fc receptor-like B
UP in U87	**100101467**	ZSCAN30	4.4982509	0.9630558	22.6	zinc finger and SCAN domain containing 30
UP in U87	**57449**	PLEKHG5	2.0530424	0.929694	4.149802	pleckstrin homology domain containing, family G (with RhoGef domain) member 5
UP in U87	**116442**	RAB39B	8.8360504	0.9636425	457	RAB39B, member RAS oncogene family
UP in U87	**55086**	CXorf57	6.1189411	0.9249827	69.5	chromosome X open reading frame 57
UP in U87	**153469**	JAKMIP2-AS1	6.1025382	0.8547834	68.71429	JAKMIP2 antisense RNA 1
UP in U87	**3113**	HLA-DPA1	11.245553	0.9960798	2428	major histocompatibility complex, class II, DP alpha 1
UP in U87	**653513**	LOC65351 3	8.5456081	0.9522197	373.6667	phosphodiesterase 4D interacting protein pseudogene
UP in U87	**9037**	SEMA5A	6.3011695	0.9700428	78.85714	sema domain, seven thrombospondin repeats (type 1 and type 1-like), transmembrane domain (TM) and short cytoplasmic domain, (semaphorin) 5A
UP in U87	**89122**	TRIM4	2.064879	0.9234715	4.183989	tripartite motif containing 4
UP in U87	**25913**	POT1	1.0276361	0.8606503	2.038681	protection of telomeres 1
UP in U87	**3598**	IL13RA2	10.634811	0.9934219	1590	interleukin 13 receptor, alpha 2
UP in U87	**55048**	VPS37C	1.3020066	0.8876029	2.465716	vacuolar protein sorting 37 homolog C (S. cerevisiae)
UP in U87	**55625**	ZDHHC7	1.4738968	0.902777	2.777712	zinc finger, DHHC-type containing 7
UP in U87	**88455**	ANKRD13	1.7255283	0.9218981	3.307012	ankyrin repeat domain 13A
UP in U87	**57507**	ZNF608	5.5759174	0.8516276	47.7	zinc finger protein 608
UP in U87	**6487**	ST3GAL3	2.5912763	0.9424769	6.026316	ST3 beta-galactoside alpha-2,3-
UP in U87	**6575**	SLC20A2	1.3671331	0.8813892	2.579574	solute carrier family 20 (phosphate transporter), member 2
UP in U87	**1520**	CTSS	8.9401668	0.9984444	491.2	cathepsin S
UP in U87	**6320**	CLEC11A	12.009595	0.9980266	4123.333	C-type lectin domain family 11, member A
UP in U87	**3516**	RBPJ	1.5871975	0.9187246	3.004651	recombination signal binding protein for immunoglobulin kappa J region
UP in U87	**828**	CAPS	3.4305006	0.9067417	10.78161	calcyphosine
UP in U87	**414328**	IDNK	2.5765221	0.9034704	5.965	idnK, gluconokinase homolog (E. coli)
UP in U87	**55578**	SUPT20H	1.1014373	0.8813626	2.145683	suppressor of Ty 20 homolog (S. cerevisiae)
UP in U87	**1725**	DHPS	2.0642237	0.9391434	4.182089	deoxyhypusine synthase
UP in U87	**4600**	MX2	4.6950599	0.9144399	25.90323	myxovirus (influenza virus) resistance 2
UP in U87	**1848**	DUSP6	5.8453117	0.9950131	57.49289	dual specificity phosphatase 6
UP in U87	**4882**	NPR2	9.5548629	0.9942397	752.1429	natriuretic peptide receptor B/guanylate cyclase B (atrionatriuretic peptide receptor B)
UP in U87	**51321**	AMZ2	1.4576034	0.9058528	2.746517	archaelysin family metallopeptidase 2

UP in U87	**116092**	DNTTIP1	1.0212879	0.8843761	2.02973	deoxynucleotidyltransferase, terminal, interacting protein 1
UP in U87	**340277**	FAM221A	4.654636	0.8161591	25.1875	family with sequence similarity 221, member
UP in U87	**90589**	ZNF625	7.0803734	0.8291198	135.3333	zinc finger protein 625
UP in U87	**54587**	MXRA8	3.8485837	0.9728697	14.40586	matrix-remodelling associated 8
UP in U87	**81792**	ADAMTS 12	9.4241663	0.9797589	687	ADAM metallopeptidase with thrombospondin type 1 motif, 12
UP in U87	**51647**	FAM96B	1.6910413	0.9248671	3.228897	family with sequence similarity 96, member
UP in U87	**84260**	TCHP	1.0758677	0.8763756	2.107989	trichoplein, keratin filament binding
UP in U87	**3077**	HFE	1.0283037	0.8326844	2.039625	hemochromatosis
UP in U87	**10039**	PARP3	1.3379567	0.8933454	2.52793	poly (ADP-ribose) polymerase family,
UP in U87	**56111**	PCDHGA4	6.864874	0.9791011	116.5556	protocadherin gamma subfamily A, 4
UP in U87	**9201**	DCLK1	4.2212719	0.8201149	18.65217	doublecortin-like kinase 1
UP in U87	**23424**	TDRD7	2.523562	0.9378989	5.75	tudor domain containing 7
UP in U87	**5055**	SERPINB2	15.524878	1	47147	serpin peptidase inhibitor, clade B (ovalbumin), member 2
UP in U87	**23180**	RFTN1	2.1179644	0.9349832	4.34081	raftlin, lipid raft linker 1
UP in U87	**2004**	ELK3	1.6802625	0.918769	3.204863	ELK3, ETS-domain protein (SRF accessory protein 2)
UP in U87	**2297**	FOXD1	5.6395286	0.994133	49.85024	forkhead box D1
UP in U87	**55733**	HHAT	4.3441428	0.8731132	20.31034	hedgehog acyltransferase
UP in U87	**3236**	HOXD10	8.6474584	0.9567888	401	homeobox D10
UP in U87	**2272**	FHIT	7.8927958	0.912342	237.6667	fragile histidine triad
UP in U87	**28951**	TRIB2	3.8974186	0.9663538	14.90184	tribbles homolog 2 (Drosophila)
UP in U87	**5580**	PRKCD	1.369265	0.8979234	2.583389	protein kinase C, delta
UP in U87	**59**	ACTA2	3.2310931	0.9637847	9.389791	actin, alpha 2, smooth muscle, aorta
UP in U87	**7461**	CLIP2	3.1253433	0.9670916	8.726138	CAP-GLY domain containing linker protein
UP in U87	**126308**	MOB3A	1.9026491	0.924796	3.738991	MOB kinase activator 3A
UP in U87	**2247**	FGF2	2.9128724	0.9625136	7.531161	fibroblast growth factor 2 (basic)
UP in U87	**375190**	FAM228B	5.2048588	0.889603	36.88235	family with sequence similarity 228, member
UP in U87	**114804**	RNF157	1.7009373	0.870322	3.251121	ring finger protein 157
UP in U87	**57688**	ZSWIM6	1.617937	0.9029015	3.069358	zinc finger, SWIM-type containing 6
UP in U87	**131578**	LRRC15	7.4957013	0.9990133	180.4808	leucine rich repeat containing 15
UP in U87	**58491**	ZNF71	2.1586775	0.9029726	4.465054	zinc finger protein 71
UP in U87	**90321**	ZNF766	1.6068317	0.8433694	3.045822	zinc finger protein 766
UP in U87	**4017**	LOXL2	3.0899099	0.9688428	8.51443	lysyl oxidase-like 2
UP in U87	**377677**	CA13	3.3438026	0.8798603	10.15278	carbonic anhydrase XIII
UP in U87	**132789**	GNPDA2	1.5148525	0.9004036	2.857696	glucosamine-6-phosphate deaminase 2
UP in U87	**933**	CD22	5.2275357	0.8744911	37.46667	CD22 molecule
UP in U87	**58476**	TP53INP2	1.4150375	0.8981368	2.666667	tumor protein p53 inducible nuclear protein 2
UP in U87	**399473**	SPRED3	4.3438026	0.9547354	20.30556	sprouty-related, EVH1 domain containing 3
UP in U87	**79695**	GALNT12	8.2240017	0.9970487	299	UDP-N-acetyl-alpha-D-galactosamine:polypeptide N-acetylgalactosaminyltransferase 12 (GalNAc-T12)
UP in U87	**2679**	GGT3P	2.7270421	0.870482	6.620968	gamma-glutamyltransferase 3 pseudogene
UP in U87	**149773**	APCDD1L-AS1	5.6820369	0.9786211	51.34091	APCDD1L antisense RNA 1 (head to head)
UP in U87	**3964**	LGALS8	1.0545603	0.8747578	2.077085	lectin, galactoside-binding, soluble, 8
UP in U87	**11147**	HHLA3	2.1088108	0.9197735	4.313356	HERV-H LTR-associating 3
UP in U87	**79629**	OCEL1	1.4290037	0.8880118	2.692607	occludin/ELL domain containing 1
UP in U87	**388963**	C2orf81	4.9004643	0.8357957	29.86667	chromosome 2 open reading frame 81
UP in U87	**5585**	PKN1	1.2318217	0.8983501	2.348634	protein kinase N1
UP in U87	**6443**	SGCB	3.1113337	0.9642648	8.641811	sarcoglycan, beta (43kDa dystrophin-associated glycoprotein)
UP in U87	**6277**	S100A6	1.7883419	0.9270628	3.454177	S100 calcium binding protein A6
UP in U87	**643723**	LOC64372	6.101538	0.8919676	68.66667	uncharacterized LOC643723

UP in U87	**144717**	FAM109A	2.1908223	0.9174	4.565657	family with sequence similarity 109, member
UP in U87	**5264**	PHYH	2.251516	0.939259	4.76183	phytanoyl-CoA 2-hydroxylase
UP in U87	**150290**	DUSP18	3.1274499	0.9343254	8.738889	dual specificity phosphatase 18
UP in U87	**650794**	MIPEPP3	3.1335409	0.8184614	8.775862	mitochondrial intermediate peptidase pseudogene 3
UP in U87	**85377**	MICALL1	2.1955452	0.9381211	4.580627	MICAL-like 1
UP in U87	**200316**	APOBEC3F	5.0353652	0.9446904	32.79412	apolipoprotein B mRNA editing enzyme, catalytic polypeptide-like 3F
UP in U87	**64943**	NT5DC2	2.578149	0.9596067	5.97173	5'-nucleotidase domain containing 2
UP in U87	**57707**	TLDC1	3.2398186	0.962318	9.446753	TBC/LysM-associated domain containing 1
UP in U87	**122809**	SOCS4	1.0538894	0.8707664	2.076119	suppressor of cytokine signaling 4
UP in U87	**399959**	MIR100HG	3.9649033	0.9839725	15.61546	mir-100-let-7a-2 cluster host gene (non-protein coding)
UP in U87	**54858**	PGPEP1	1.4330467	0.8868295	2.700163	pyroglutamyl-peptidase I
UP in U87	**8702**	B4GALT4	2.0424609	0.9321297	4.119476	UDP-Gal:betaGlcNAc beta 1,4-galactosyltransferase, polypeptide 4
UP in U87	**149934**	NCOR1P1	6.9503129	0.8137678	123.6667	nuclear receptor corepressor 1 pseudogene 1
UP in U87	**147463**	ANKRD29	2.4946302	0.9367877	5.635838	ankyrin repeat domain 29
UP in U87	**4053**	LTBP2	3.115639	0.9552954	8.667638	latent transforming growth factor beta binding protein 2
UP in U87	**1496**	CTNNA2	6.9848931	0.9522908	126.6667	catenin (cadherin-associated protein), alpha 2
UP in U87	**128989**	TANGO2	1.7614041	0.9223603	3.390279	transport and golgi organization 2 homolog (Drosophila)
UP in U87	**610**	HCN2	3.0493564	0.9633403	8.278426	hyperpolarization activated cyclic nucleotide-gated potassium channel 2
UP in U87	**157773**	C8orf48	5.3364534	0.9681227	40.40476	chromosome 8 open reading frame 48
UP in U87	**8710**	SERPINB7	12.725437	0.9991644	6772.333	serpin peptidase inhibitor, clade B (ovalbumin), member 7
UP in U87	**253980**	KCTD13	1.2644481	0.8839405	2.402353	potassium channel tetramerisation domain containing 13
UP in U87	**6690**	SPINK1	6.9307373	0.8105499	122	serine peptidase inhibitor, Kazal type 1
UP in U87	**5651**	TMPRSS1	10.522722	0.997431	1471.143	transmembrane protease, serine 15
UP in U87	**65062**	TMEM237	1.1287641	0.867104	2.186713	transmembrane protein 237
UP in U87	**347404**	LANCL3	7.5824556	0.8856739	191.6667	LanC lantibiotic synthetase component C-like 3 (bacterial)
UP in U87	**10755**	GIPC1	1.1987074	0.8967323	2.295339	GIPC PDZ domain containing family,
UP in U87	**10903**	MTMR11	1.4654055	0.8382936	2.761411	myotubularin related protein 11
UP in U87	**114**	ADCY8	9.4723524	0.9806479	710.3333	adenylate cyclase 8 (brain)
UP in U87	**23586**	DDX58	3.2277295	0.9075062	9.367925	DEAD (Asp-Glu-Ala-Asp) box polypeptide
UP in U87	**55691**	FRMD4A	4.5708246	0.9729141	23.76596	FERM domain containing 4A
UP in U87	**145567**	TTC7B	3.7309247	0.9750831	13.27762	tetratricopeptide repeat domain 7B
UP in U87	**126375**	ZNF792	3.9785659	0.9472239	15.76404	zinc finger protein 792
UP in U87	**23515**	MORC3	1.3778317	0.8853717	2.598775	MORC family CW-type zinc finger 3
UP in U87	**54704**	PDP1	2.8742289	0.9595978	7.332112	pyruvate dehyrogenase phosphatase catalytic subunit 1
UP in U87	**1513**	CTSK	6.3751238	0.9970221	83.00485	cathepsin K
UP in U87	**1912**	PHC2	2.0465833	0.9401479	4.131264	polyhomeotic homolog 2 (Drosophila)
UP in U87	**4148**	MATN3	3.2143551	0.9255427	9.281481	matrilin 3
UP in U87	**719**	C3AR1	8.7747871	0.9615091	438	complement component 3a receptor 1
UP in U87	**6773**	STAT2	1.4550794	0.9001547	2.741717	signal transducer and activator of transcription 2, 113kDa
UP in U87	**388389**	CCDC103	2.6364732	0.9421569	6.218097	coiled-coil domain containing 103
UP in U87	**376497**	SLC27A1	2.8525765	0.9452771	7.222892	solute carrier family 27 (fatty acid transporter), member 1
UP in U87	**55129**	ANO10	2.9068906	0.9623802	7.5	anoctamin 10
UP in U87	**10810**	WASF3	5.478204	0.9814035	44.57627	WAS protein family, member 3
UP in U87	**79668**	PARP8	7.8765169	0.9108664	235	poly (ADP-ribose) polymerase family,

UP in U87	**171489**	SPANXE	8.741467	0.9603268	428	SPANX family, member E
UP in U87	**7545**	ZIC1	7.8600523	0.9092396	232.3333	Zic family member 1
UP in U87	**162968**	ZNF497	4.4618584	0.8760556	22.03704	zinc finger protein 497
UP in U87	**11257**	TP53TG1	4.3773282	0.9752787	20.78295	TP53 target 1 (non-protein coding)
UP in U87	**84807**	NFKBID	1.1154772	0.8061586	2.166667	nuclear factor of kappa light polypeptide gene enhancer in B-cells inhibitor, delta
UP in U87	**10600**	USP16	1.0671352	0.8807581	2.095269	ubiquitin specific peptidase 16
UP in U87	**10490**	VTI1B	1.3083939	0.8995324	2.476657	vesicle transport through interaction with t-SNAREs 1B
UP in U87	**57464**	STRIP2	2.52813	0.9450993	5.768235	striatin interacting protein 2
UP in U87	**149951**	COMMD7	1.7107625	0.9213914	3.273338	COMM domain containing 7
UP in U87	**164284**	APCDD1L	7.1578004	0.9937774	142.7949	adenomatosis polyposis coli down-regulated 1-like
UP in U87	**100506144**	ZMYM6N	1.7028838	0.9195424	3.255511	ZMYM6 neighbor
UP in U87	**64764**	CREB3L2	1.486218	0.9052572	2.801536	cAMP responsive element binding protein 3-like 2
UP in U87	**10227**	MFSD10	1.1471622	0.8909097	2.214778	major facilitator superfamily domain
UP in U87	**79630**	C1orf54	3.6641892	0.9478017	12.67742	chromosome 1 open reading frame 54
UP in U87	**84981**	MIR22HG	1.9585876	0.9243693	3.886813	MIR22 host gene (non-protein coding)
UP in U87	**64750**	SMURF2	1.1959073	0.8801181	2.290889	SMAD specific E3 ubiquitin protein ligase 2
UP in U87	**100533483**	DYX1C1-CCPG1	5.7364019	0.9259783	53.3125	DYX1C1-CCPG1 readthrough (non-protein coding)
UP in U87	**152559**	PAQR3	1.6125638	0.9149021	3.057948	progestin and adipoQ receptor family
