# Supplementary material for: Integrated analysis identified core signal pathways and hypoxic characteristics of human glioblastoma
Source: J Cell Mol Med. 2019 Jul 7;23(9):6228–37. doi: 10.1111/jcmm.14507 (PMC6714287; doi:10.1111/jcmm.14507)
Supplement: Supplementary file 12 [file JCMM-23-6228-s013.pdf]

Table S5 List of over-represented gene ontology biological process subcategories (GOBPID) in the genes differentially expressed (log2 fold change >1) with statistical significance (BH-corrected p-value <0.05) between U87-MG versus HEB cell.

a. List of gene ontology biological process subcategories in genes up-regulated in U87-MG versus HEB cell

	GOBPID	P value	Count	%	GO Term	P value.BH corrected
1	GO:0032989	2.1554E-08	112	0.25629291	cellular component morphogenesis	0.0001044
2	GO:0010634	8.4744E-07	33	0.41772152	positive regulation of epithelial cell migration	0.0013965
3	GO:0030335	1.1797E-06	75	0.26785714	positive regulation of cell migration	0.0013965
4	GO:0051272	1.2329E-06	78	0.26351351	positive regulation of cellular component movement	0.0013965
5	GO:2000147	1.442E-06	76	0.26480836	positive regulation of cell motility	0.0013965
6	GO:0022604	1.9678E-06	98	0.24019608	regulation of cell morphogenesis	0.001588
7	GO:0040017	2.8825E-06	78	0.25657895	positive regulation of locomotion	0.0019939
8	GO:0045766	5.3748E-06	36	0.35643564	positive regulation of angiogenesis	0.0029057
9	GO:0030198	5.4748E-06	87	0.24233983	extracellular matrix organization	0.0029057
10	GO:0043062	6.0011E-06	87	0.24166667	extracellular structure organization	0.0029057
11	GO:0030155	8.1159E-06	82	0.24404762	regulation of cell adhesion	0.0035725
12	GO:0001525	9.0263E-06	63	0.26694915	angiogenesis	0.0036421
13	GO:0045860	1.0725E-05	103	0.22587719	positive regulation of protein kinase activity	0.0039946
14	GO:0010632	1.2398E-05	41	0.31782946	regulation of epithelial cell migration	0.0042879
15	GO:0033674	1.8195E-05	106	0.22083333	positive regulation of kinase activity	0.0058732
16	GO:0071345	3.3426E-05	103	0.21868365	cellular response to cytokine stimulus	0.0101155
17	GO:0010595	3.699E-05	23	0.41818182	positive regulation of endothelial cell	0.0103552
18	GO:0006954	3.8495E-05	86	0.2287234	inflammatory response	0.0103552
19	GO:0000902	4.6387E-05	54	0.26470588	cell morphogenesis	0.011764
20	GO:0044708	4.8591E-05	83	0.22928177	single-organism behavior	0.011764
21	GO:0010563	6.9144E-05	99	0.21615721	negative regulation of phosphorus metabolic process	0.0152179
22	GO:0045936	6.9144E-05	99	0.21615721	negative regulation of phosphate metabolic process	0.0152179
23	GO:0007264	9.2951E-05	95	0.21640091	small GTPase mediated signal transduction	0.0182043
24	GO:0032990	9.3451E-05	61	0.24596774	cell part morphogenesis	0.0182043
25	GO:0006790	9.3992E-05	73	0.23248408	sulfur compound metabolic process	0.0182043
26	GO:0001933	0.000104	63	0.24230769	negative regulation of protein	0.0193674
27	GO:0048858	0.00010856	57	0.25	cell projection morphogenesis	0.0194687
28	GO:1902532	0.00011936	78	0.22608696	negative regulation of intracellular signal transduction	0.0197438
29	GO:0045785	0.00011975	46	0.26900585	positive regulation of cell adhesion	0.0197438
30	GO:0060333	0.00012233	24	0.36923077	interferon-gamma-mediated signaling	0.0197438
31	GO:0032940	0.00013209	90	0.21686747	secretion by cell	0.0205487
32	GO:0010720	0.0001358	80	0.22346369	positive regulation of cell development	0.0205487
33	GO:0071900	0.00014185	90	0.21634615	regulation of protein serine/threonine kinase activity	0.0208137
34	GO:0007033	0.00015883	23	0.37096774	vacuole organization	0.0226196
35	GO:0070201	0.00017161	94	0.21266968	regulation of establishment of protein	0.0235083
36	GO:0007596	0.00017964	99	0.20974576	blood coagulation	0.0235083
37	GO:0050817	0.00017964	99	0.20974576	coagulation	0.0235083
38	GO:0043542	0.00018801	20	0.4	endothelial cell migration	0.023956
39	GO:0006986	0.00021214	38	0.28148148	response to unfolded protein	0.0261966
40	GO:1903034	0.00021641	77	0.22190202	regulation of response to wounding	0.0261966
41	GO:0001817	0.00023416	100	0.20746888	regulation of cytokine production	0.0273724
42	GO:0009611	0.00024457	44	0.26347305	response to wounding	0.0273724
43	GO:0010594	0.00024724	29	0.31521739	regulation of endothelial cell migration	0.0273724

44	GO:0010769	0.0002557	59	0.23790323	regulation of cell morphogenesis involved in differentiation	0.0273724
45	GO:0007599	0.00026456	99	0.20711297	hemostasis	0.0273724
46	GO:0097485	0.00026614	80	0.21798365	neuron projection guidance	0.0273724
47	GO:0007411	0.00026614	80	0.21798365	axon guidance	0.0273724
48	GO:0008360	0.00027135	33	0.29464286	regulation of cell shape	0.0273724
49	GO:0031349	0.00029948	63	0.23161765	positive regulation of defense response	0.029594
50	GO:0007229	0.00030873	27	0.32142857	integrin-mediated signaling pathway	0.0296277
51	GO:0010770	0.00031206	35	0.28455285	positive regulation of cell morphogenesis involved in differentiation	0.0296277
52	GO:0019221	0.00035061	75	0.21929825	cytokine-mediated signaling pathway	0.0324804
53	GO:0042326	0.00035553	72	0.22153846	negative regulation of phosphorylation	0.0324804
54	GO:1901215	0.00036533	41	0.26451613	negative regulation of neuron death	0.0327576
55	GO:0034976	0.00038074	39	0.26896552	response to endoplasmic reticulum stress	0.0335193
56	GO:0050890	0.00045727	54	0.23788546	cognition	0.0391841
57	GO:0006469	0.00046128	47	0.24867725	negative regulation of protein kinase activity	0.0391841
58	GO:0006887	0.00047737	55	0.2360515	exocytosis	0.0398523
59	GO:0045773	0.00048717	13	0.5	positive regulation of axon extension	0.039981
60	GO:0043405	0.00051554	62	0.22710623	regulation of MAP kinase activity	0.041056
61	GO:0032103	0.00051723	49	0.24378109	positive regulation of response to external stimulus	0.041056
62	GO:0035966	0.0005459	38	0.26573427	response to topologically incorrect protein	0.0425855
63	GO:0071346	0.00056288	27	0.30681818	cellular response to interferon-gamma	0.0425855
64	GO:0048812	0.00056746	44	0.25142857	neuron projection morphogenesis	0.0425855
65	GO:0043122	0.00057168	51	0.23943662	regulation of I-kappaB kinase/NF-kappaB signaling	0.0425855
66	GO:0010810	0.00059546	39	0.26174497	regulation of cell-substrate adhesion	0.0431671
67	GO:0010575	0.00059731	12	0.52173913	positive regulation vascular endothelial growth factor production	0.0431671
68	GO:0010631	0.00061132	22	0.33846154	epithelial cell migration	0.0435298
69	GO:0010574	0.00064186	13	0.48148148	regulation of vascular endothelial growth factor production	0.0450416
70	GO:1903510	0.0006577	34	0.27419355	mucopolysaccharide metabolic process	0.045494
71	GO:0034110	0.00072161	11	0.55	regulation of homotypic cell-cell adhesion	0.0492121

b. List of gene ontology biological process subcategories in genes up-regulated in U87-MG versus HEB cell after processing with REViGO.

Term_ID	Description	Uniqueness	Dispensability
GO:0044708	single-organism behavior	0.908	0.089
GO:0006790	sulfur compound metabolic process	0.898	0.042
GO:0007033	vacuole organization	0.814	0.317
GO:0006954	inflammatory response	0.812	0
GO:0050890	cognition	0.812	0.326
GO:0006986	response to unfolded protein	0.807	0.435
GO:0050817	coagulation	0.806	0.342
GO:0035966	response to topologically incorrect protein	0.806	0.485
GO:0032103	positive regulation of response to external stimulus	0.798	0.167
GO:0032940	secretion by cell	0.785	0.298
GO:0034976	response to endoplasmic reticulum stress	0.777	0.448
GO:0030198	extracellular matrix organization	0.764	0.086
GO:0043062	extracellular structure organization	0.764	0.37
GO:0030155	regulation of cell adhesion	0.747	0.175
GO:1901215	negative regulation of neuron death	0.734	0.165
GO:0007229	integrin-mediated signaling pathway	0.724	0.337
GO:0001817	regulation of cytokine production	0.698	0.35
GO:0007264	small GTPase mediated signal transduction	0.671	0.206
GO:0001525	angiogenesis	0.655	0.019
GO:0019221	cytokine-mediated signaling pathway	0.632	0.48
GO:0045860	positive regulation of protein kinase activity	0.596	0.458
GO:0010720	positive regulation of cell development	0.567	0.431
GO:0030335	positive regulation of cell migration	0.541	0

c. List of gene ontology biological process subcategories in genes down-regulated in U87-MG versus HEB cell

	**GOBPID**	**P value**	**Count**	**%**	**GO Term**	**Pvalue.BH corrected**
1	GO:004232	7.6323E-07	78	0.24	negative regulation of phosphorylation	0.003649
2	GO:000626	1.1002E-05	49	0.26344086	DNA replication	0.0191681
3	GO:000193	1.2028E-05	62	0.23846154	negative regulation of protein phosphorylation	0.0191681
4	GO:000631	6.4377E-05	48	0.24489796	DNA recombination	0.0471051
5	GO:001038	6.6256E-05	16	0.47058824	regulation of G2/M transition of mitotic cell cycle	0.0471051
6	GO:004593	6.8968E-05	91	0.19868996	negative regulation of phosphate metabolic process	0.0471051
7	GO:001056	6.8968E-05	91	0.19868996	negative regulation of phosphorus metabolic proces	0.0471051
8	GO:000734	8.1732E-05	80	0.20460358	regulation of mitotic cell cycle	0.0487975
9	GO:002241	9.9147E-05	73	0.20857143	cellular component disassembly	0.0487975
10	GO:007190	0.00011195	33	0.27966102	negative regulation of protein serine/threonine kinas	0.0487975
11	GO:190274	0.00011227	16	0.44444444	regulation of cell cycle G2/M phase transition	0.0487975

d. List of gene ontology biological process subcategories in genes down-regulated in U87-MG versus HEB cell after processing with REViGO.

Term_ID	Description	Uniqueness	Dispensability
GO:0001933	negative regulation of protein phosphorylation	0.216	0
GO:0042326	negative regulation of phosphorylation	0.232	0.955
GO:0071901	negative regulation of protein serine/threonine kinase acti	0.243	0.88
GO:0010563	negative regulation of phosphorus metabolic process	0.327	0.647
GO:0045936	negative regulation of phosphate metabolic process	0.23	0.982
GO:0022411	cellular component disassembly	0.72	0.042
GO:0007346	regulation of mitotic cell cycle	0.443	0.178
GO:0010389	regulation of G2/M transition of mitotic cell cycle	0.445	0.654
GO:1902749	regulation of cell cycle G2/M phase transition	0.448	0.826
GO:0006260	DNA replication	0.557	0.208
GO:0006310	DNA recombination	0.563	0.675
