# Supplementary material for: Integrated analysis identified core signal pathways and hypoxic characteristics of human glioblastoma
Source: J Cell Mol Med. 2019 Jul 7;23(9):6228–37. doi: 10.1111/jcmm.14507 (PMC6714287; doi:10.1111/jcmm.14507)
Supplement: Supplementary file 13 [file JCMM-23-6228-s014.pdf]

Table S6 Differential gene expression analysis by Noiseq for pairwise comparison of U87-MG normoxia versus 1% hypoxia, based on RNA-seq data.

	Gene	Symbol	log_2Ratio	Probability	Ratio	Description
UP in Normoxia	**10309**	CCNO	-1.373501	0.8480426	0.3859536	cyclin O
UP in Normoxia	**80176**	SPSB1	-1.161828	0.8760638	0.446946	splA/ryanodine receptor domain and SOCS box containing 1
UP in Normoxia	**2634**	GBP2	-1.942466	0.8945534	0.2601713	guanylate binding protein 2, interferon-
UP in Normoxia	**339263**	C17orf51	-1.362414	0.8733584	0.388931	chromosome 17 open reading frame 51
UP in Normoxia	**1969**	EPHA2	-1.575676	0.9017289	0.3354859	EPH receptor A2
UP in Normoxia	**84275**	SLC25A33	-2.746663	0.9217952	0.1489951	solute carrier family 25 (pyrimidine nucleotide carrier), member 33
UP in Normoxia	**9961**	MVP	-1.066233	0.8836513	0.4775643	major vault protein
UP in Normoxia	**5464**	PPA1	-1.573604	0.9026247	0.335968	pyrophosphatase (inorganic) 1
UP in Normoxia	**8193**	DPF1	-1.968501	0.8846815	0.2555184	D4, zinc and double PHD fingers family 1
UP in Normoxia	**4261**	CIITA	-3.851938	0.9621965	0.069255	class II, major histocompatibility complex, transactivator
UP in Normoxia	**100289092**	LOC100289092	-2.212792	0.9213921	0.2157165	uncharacterized LOC100289092
UP in Normoxia	**84649**	DGAT2	-2.034661	0.919269	0.2440653	diacylglycerol O-acyltransferase 2
UP in Normoxia	**6631**	SNRPC	-1.102041	0.8818508	0.465857	small nuclear ribonucleoprotein polypeptide
UP in Normoxia	**79154**	DHRS11	-2.179387	0.92184	0.2207695	dehydrogenase/reductase (SDR family) member 11
UP in Normoxia	**23171**	GPD1L	-1.970279	0.9118248	0.2552036	glycerol-3-phosphate dehydrogenase 1-like
UP in Normoxia	**221491**	C6orf1	-1.157564	0.8763594	0.4482688	chromosome 6 open reading frame 1
UP in Normoxia	**3419**	IDH3A	-1.749618	0.9175311	0.2973804	isocitrate dehydrogenase 3 (NAD+) alpha
UP in Normoxia	**7514**	XPO1	-1.258483	0.8917495	0.4179831	exportin 1 (CRM1 homolog, yeast)
UP in Normoxia	**39**	ACAT2	-1.505549	0.8933083	0.3521961	acetyl-CoA acetyltransferase 2
UP in Normoxia	**51025**	PAM16	-1.026812	0.8676789	0.4907937	presequence translocase-associated motor 16 homolog (S. cerevisiae)
UP in Normoxia	**79823**	CAMKMT	-1.708951	0.8249485	0.3058824	calmodulin-lysine N-methyltransferase
UP in Normoxia	**506**	ATP5B	-1.045865	0.883078	0.4843545	ATP synthase, H+ transporting, mitochondrial F1 complex, beta polypeptide
UP in Normoxia	**64782**	AEN	-2.055336	0.9345964	0.2405926	apoptosis enhancing nuclease
UP in Normoxia	**4218**	RAB8A	-1.018868	0.8771925	0.4935033	RAB8A, member RAS oncogene family
UP in Normoxia	**1983**	EIF5	-1.215564	0.8904416	0.4306048	eukaryotic translation initiation factor 5
UP in Normoxia	**7247**	TSN	-1.088254	0.8799875	0.4703301	translin
UP in Normoxia	**84816**	RTN4IP1	-2.555485	0.9343098	0.170107	reticulon 4 interacting protein 1
UP in Normoxia	**9401**	RECQL4	-1.644863	0.906002	0.3197767	RecQ protein-like 4
UP in Normoxia	**23277**	CLUH	-1.474002	0.8971513	0.3599824	clustered mitochondria (cluA/CLU1)
UP in Normoxia	**154467**	CCDC16	-1.298694	0.8663352	0.406494	coiled-coil domain containing 167
UP in Normoxia	**9133**	CCNB2	-1.363362	0.8939443	0.3886755	cyclin B2
UP in Normoxia	**146059**	CDAN1	-1.282762	0.8322404	0.4110079	codanin 1
UP in Normoxia	**8607**	RUVBL1	-1.821286	0.9212936	0.2829687	RuvB-like 1 (E. coli)
UP in Normoxia	**788**	SLC25A20	-1.213281	0.852683	0.4312865	solute carrier family 25 (carnitine/ acylcarnitine translocase), member 20
UP in Normoxia	**144402**	CPNE8	-1.403558	0.8767536	0.3779956	copine VIII
UP in Normoxia	**84315**	MON1A	-1.730834	0.9037535	0.3012777	MON1 homolog A (yeast)
UP in Normoxia	**6502**	SKP2	-1.245146	0.873027	0.4218652	S-phase kinase-associated protein 2, E3 ubiquitin protein ligase
UP in Normoxia	**10544**	PROCR	-1.013944	0.8795754	0.4951907	protein C receptor, endothelial
UP in Normoxia	**10713**	USP39	-1.196589	0.8860701	0.4363057	ubiquitin specific peptidase 39
UP in Normoxia	**653238**	GTF2H2B	-1.14802	0.8639703	0.4512441	general transcription factor IIH, polypeptide 2B (pseudogene)
UP in Normoxia	**146909**	KIF18B	-1.907405	0.907283	0.2665716	kinesin family member 18B

UP in Normoxia	**10550**	ARL6IP5	-1.16647	0.887154	0.4455101	ADP-ribosylation-like factor 6 interacting protein 5
UP in Normoxia	**64216**	TFB2M	-1.283118	0.8805518	0.4109065	transcription factor B2, mitochondrial
UP in Normoxia	**83882**	TSPAN1	-1.211214	0.8592403	0.431905	tetraspanin 10
UP in Normoxia	**125476**	INO80C	-1.489552	0.8437965	0.3561232	INO80 complex subunit C
UP in Normoxia	**55213**	RCBTB1	-1.401307	0.8626892	0.3785861	regulator of chromosome condensation (RCC1) and BTB (POZ) domain containing protein 1
UP in Normoxia	**100533975**	SLMO2-ATP5E	-1.026051	0.8675625	0.4910524	SLMO2-ATP5E readthrough
UP in Normoxia	**55706**	TMEM48	-1.486099	0.8940697	0.3569765	transmembrane protein 48
UP in Normoxia	**10885**	WDR3	-1.525494	0.8947416	0.3473605	WD repeat domain 3
UP in Normoxia	**29114**	TAGLN3	-1.949063	0.9184897	0.2589843	transgelin 3
UP in Normoxia	**2936**	GSR	-1.553353	0.8995521	0.3407172	glutathione reductase
UP in Normoxia	**9497**	SLC4A7	-1.725615	0.9127116	0.3023697	solute carrier family 4, sodium bicarbonate cotransporter, member 7
UP in Normoxia	**256691**	MAMDC	-2.489584	0.8185165	0.1780576	MAM domain containing 2
UP in Normoxia	**8884**	SLC5A6	-1.535191	0.891212	0.3450336	solute carrier family 5 (sodium-dependent vitamin transporter), member 6
UP in Normoxia	**7262**	PHLDA2	-1.607173	0.9076503	0.3282408	pleckstrin homology-like domain, family A, member 2
UP in Normoxia	**29781**	NCAPH2	-1.308707	0.8904506	0.4036824	non-SMC condensin II complex, subunit H2
UP in Normoxia	**65094**	JMJD4	-2.104546	0.9210517	0.2325244	jumonji domain containing 4
UP in Normoxia	**10451**	VAV3	-1.003662	0.8150856	0.4987326	vav 3 guanine nucleotide exchange factor
UP in Normoxia	**4076**	CAPRIN	-1.028429	0.879871	0.4902437	cell cycle associated protein 1
UP in Normoxia	**3832**	KIF11	-2.061579	0.928478	0.2395537	kinesin family member 11
UP in Normoxia	**1655**	DDX5	-1.034354	0.8811341	0.4882345	DEAD (Asp-Glu-Ala-Asp) box helicase 5
UP in Normoxia	**5077**	PAX3	-1.827731	0.8271343	0.2817073	paired box 3
UP in Normoxia	**201254**	STRA13	-1.142208	0.8844576	0.4530655	stimulated by retinoic acid 13
UP in Normoxia	**55299**	BRIX1	-2.225036	0.9371316	0.2138934	BRX1, biogenesis of ribosomes, homolog (S. cerevisiae)
UP in Normoxia	**78991**	PCYOX1	-2.262894	0.9153095	0.2083536	prenylcysteine oxidase 1 like
UP in Normoxia	**741**	ZNHIT2	-1.526691	0.8806772	0.3470724	zinc finger, HIT-type containing 2
UP in Normoxia	**2806**	GOT2	-1.586463	0.9165278	0.3329869	glutamic-oxaloacetic transaminase 2, mitochondrial (aspartate aminotransferase 2)
UP in Normoxia	**1545**	CYP1B1	-3.215212	0.9703664	0.1076774	cytochrome P450, family 1, subfamily B, polypeptide 1
UP in Normoxia	**1058**	CENPA	-1.787839	0.9069605	0.2896055	centromere protein A
UP in Normoxia	**55723**	ASF1B	-2.88227	0.9602795	0.1356283	ASF1 anti-silencing function 1 homolog B (S. cerevisiae)
UP in Normoxia	**79672**	FN3KRP	-1.025732	0.8751321	0.4911612	fructosamine 3 kinase related protein
UP in Normoxia	**5564**	PRKAB1	-1.008883	0.8625549	0.4969309	protein kinase, AMP-activated, beta 1 non-catalytic subunit
UP in Normoxia	**4521**	NUDT1	-1.288199	0.8909433	0.4094618	nudix (nucleoside diphosphate linked moiety X)-type motif 1
UP in Normoxia	**84942**	WDR73	-1.018418	0.8617755	0.4936572	WD repeat domain 73
UP in Normoxia	**5976**	UPF1	-1.062656	0.8767088	0.4787499	UPF1 regulator of nonsense transcripts homolog (yeast)
UP in Normoxia	**51112**	TRAPPC 12	-1.007286	0.8725343	0.4974811	trafficking protein particle complex 12
UP in Normoxia	**9200**	PTPLA	-1.196208	0.8697393	0.4364207	protein tyrosine phosphatase-like (proline instead of catalytic arginine), member A
UP in Normoxia	**79590**	MRPL24	-1.192184	0.8886321	0.4376397	mitochondrial ribosomal protein L24
UP in Normoxia	**79959**	CEP76	-1.726943	0.8658336	0.3020914	centrosomal protein 76kDa
UP in Normoxia	**80185**	TTI2	-1.076542	0.8540804	0.4741641	TELO2 interacting protein 2
UP in Normoxia	**58489**	ABHD17	-1.094619	0.8098002	0.4682599	abhydrolase domain containing 17C
UP in Normoxia	**27346**	TMEM97	-1.934487	0.913482	0.2616143	transmembrane protein 97

UP in Normoxia	**9127**	P2RX6	-1.557195	0.8667204	0.3398112	purinergic receptor P2X, ligand-gated ion channel, 6
UP in Normoxia	**51434**	ANAPC7	-1.611856	0.9117173	0.3271772	anaphase promoting complex subunit 7
UP in Normoxia	**10921**	RNPS1	-1.166722	0.8855057	0.4454321	RNA binding protein S1, serine-rich domain
UP in Normoxia	**3108**	HLA-DMA	-1.567763	0.8915077	0.337331	major histocompatibility complex, class II, DM alpha
UP in Normoxia	**644**	BLVRA	-1.309989	0.8928245	0.403324	biliverdin reductase A
UP in Normoxia	**1802**	DPH2	-1.757512	0.910911	0.2957579	DPH2 homolog (S. cerevisiae)
UP in Normoxia	**79968**	WDR76	-3.084177	0.9553794	0.1179153	WD repeat domain 76
UP in Normoxia	**92335**	STRADA	-1.146389	0.8728209	0.4517544	STE20-related kinase adaptor alpha
UP in Normoxia	**4357**	MPST	-1.077707	0.8665771	0.4737813	mercaptopyruvate sulfurtransferase
UP in Normoxia	**5796**	PTPRK	-2.180824	0.9244648	0.2205497	protein tyrosine phosphatase, receptor type,
UP in Normoxia	**26517**	TIMM13	-1.08937	0.874514	0.4699665	translocase of inner mitochondrial membrane 13 homolog (yeast)
UP in Normoxia	**56683**	C21orf59	-1.078352	0.8801577	0.4735696	chromosome 21 open reading frame 59
UP in Normoxia	**5245**	PHB	-1.079595	0.8837499	0.4731616	prohibitin
UP in Normoxia	**84899**	TMTC4	-1.470271	0.8637463	0.3609145	transmembrane and tetratricopeptide repeat containing 4
UP in Normoxia	**1435**	CSF1	-2.327369	0.9384843	0.1992471	colony stimulating factor 1 (macrophage)
UP in Normoxia	**54496**	PRMT7	-1.258688	0.8808474	0.4179239	protein arginine methyltransferase 7
UP in Normoxia	**483**	ATP1B3	-1.113488	0.8849772	0.4621752	ATPase, Na+/K+ transporting, beta 3 polypeptide
UP in Normoxia	**27338**	UBE2S	-1.443164	0.9007167	0.3677598	ubiquitin-conjugating enzyme E2S
UP in Normoxia	**58527**	ABRACL	-1.506353	0.8785989	0.352	ABRA C-terminal like
UP in Normoxia	**23411**	SIRT1	-1.077019	0.8509182	0.4740074	sirtuin 1
UP in Normoxia	**55165**	CEP55	-2.00649	0.9347398	0.2488778	centrosomal protein 55kDa
UP in Normoxia	**81493**	SYNC	-1.041558	0.8785362	0.4858025	syncoilin, intermediate filament protein
UP in Normoxia	**9521**	EEF1E1	-1.991849	0.9186151	0.2514164	eukaryotic translation elongation factor 1 epsilon 1
UP in Normoxia	**8458**	TTF2	-2.596251	0.9434829	0.1653677	transcription termination factor, RNA polymerase II
UP in Normoxia	**115752**	DIS3L	-1.870124	0.9027143	0.27355	DIS3 mitotic control homolog (S.
UP in Normoxia	**51686**	OAZ3	-1.564378	0.8376064	0.3381234	ornithine decarboxylase antizyme 3
UP in Normoxia	**285989**	ZNF789	-1.058285	0.8434561	0.4802026	zinc finger protein 789
UP in Normoxia	**81691**	LOC8169	-1.665258	0.8942578	0.315288	exonuclease NEF-sp
UP in Normoxia	**55872**	PBK	-1.970945	0.9228792	0.2550859	PDZ binding kinase
UP in Normoxia	**11218**	DDX20	-1.73555	0.8923497	0.3002944	DEAD (Asp-Glu-Ala-Asp) box polypeptide
UP in Normoxia	**26084**	ARHGEF 26	-2.436387	0.8257547	0.1847458	Rho guanine nucleotide exchange factor (GEF) 26
UP in Normoxia	**273**	AMPH	-1.294192	0.8789035	0.4077644	amphiphysin
UP in Normoxia	**29128**	UHRF1	-3.536985	0.9726059	0.0861512	ubiquitin-like with PHD and ring finger domains 1
UP in Normoxia	**84542**	KIAA184	-1.036994	0.8382245	0.4873418	KIAA1841
UP in Normoxia	**10425**	ARIH2	-1.200634	0.8863567	0.4350842	ariadne homolog 2 (Drosophila)
UP in Normoxia	**79810**	PTCD2	-1.017223	0.847532	0.4940665	pentatricopeptide repeat domain 2
UP in Normoxia	**51192**	CKLF	-1.489848	0.8997402	0.3560502	chemokine-like factor
UP in Normoxia	**51765**	MST4	-2.199531	0.9236137	0.2177083	serine/threonine protein kinase MST4
UP in Normoxia	**3084**	NRG1	-1.444284	0.8984413	0.3674744	neuregulin 1
UP in Normoxia	**54758**	KLHDC4	-1.661281	0.895933	0.3161583	kelch domain containing 4
UP in Normoxia	**23195**	MDN1	-1.101553	0.8436531	0.4660147	MDN1, midasin homolog (yeast)
UP in Normoxia	**56652**	C10orf2	-1.799196	0.902786	0.2873346	chromosome 10 open reading frame 2
UP in Normoxia	**896**	CCND3	-1.590143	0.9157932	0.3321385	cyclin D3
UP in Normoxia	**54873**	PALMD	-2.112403	0.9346144	0.2312614	palmdelphin
UP in Normoxia	**6470**	SHMT1	-1.826487	0.9123444	0.2819503	serine hydroxymethyltransferase 1 (soluble)
UP in Normoxia	**9526**	MPDU1	-1.047235	0.8794141	0.4838945	mannose-P-dolichol utilization defect 1
UP in Normoxia	**7027**	TFDP1	-1.241415	0.8904685	0.4229577	transcription factor Dp-1

UP in Normoxia	**115708**	TRMT61 A	-1.274396	0.8797366	0.4133983	tRNA methyltransferase 61 homolog A (S. cerevisiae)
UP in Normoxia	**81539**	SLC38A1	-1.393209	0.8949745	0.3807171	solute carrier family 38, member 1
UP in Normoxia	**9636**	ISG15	-1.153338	0.8798083	0.4495837	ISG15 ubiquitin-like modifier
UP in Normoxia	**7701**	ZNF142	-1.11163	0.8465198	0.462771	zinc finger protein 142
UP in Normoxia	**25903**	OLFML2	-2.715639	0.9312192	0.1522339	olfactomedin-like 2B
UP in Normoxia	**401505**	TOMM5	-1.327298	0.896354	0.3985139	translocase of outer mitochondrial membrane 5 homolog (yeast)
UP in Normoxia	**79637**	ARMC7	-1.022643	0.8112783	0.4922139	armadillo repeat containing 7
UP in Normoxia	**26271**	FBXO5	-1.975752	0.9151841	0.2542373	F-box protein 5
UP in Normoxia	**29117**	BRD7	-1.029165	0.8742094	0.4899935	bromodomain containing 7
UP in Normoxia	**5684**	PSMA3	-1.823685	0.9228164	0.2824985	proteasome (prosome, macropain) subunit, alpha type, 3
UP in Normoxia	**285855**	RPL7L1	-1.009438	0.8759742	0.4967398	ribosomal protein L7-like 1
UP in Normoxia	**78988**	MRP63	-1.057333	0.8189824	0.4805195	mitochondrial ribosomal protein 63
UP in Normoxia	**129831**	RBM45	-1.353343	0.8658425	0.3913841	RNA binding motif protein 45
UP in Normoxia	**10898**	CPSF4	-1.104251	0.873672	0.4651438	cleavage and polyadenylation specific factor 4, 30kDa
UP in Normoxia	**7867**	MAPKA PK3	-1.24484	0.8911404	0.4219546	mitogen-activated protein kinase-activated protein kinase 3
UP in Normoxia	**3554**	IL1R1	-1.451386	0.88865	0.3656699	interleukin 1 receptor, type I
UP in Normoxia	**6936**	GCFC2	-1.835969	0.8933799	0.2801034	GC-rich sequence DNA-binding factor 2
UP in Normoxia	**1000**	CDH2	-1.144748	0.8680462	0.4522687	cadherin 2, type 1, N-cadherin (neuronal)
UP in Normoxia	**11222**	MRPL3	-1.303565	0.893577	0.4051238	mitochondrial ribosomal protein L3
UP in Normoxia	**5367**	PMCH	-2.100871	0.8759832	0.2331175	pro-melanin-concentrating hormone
UP in Normoxia	**22974**	TPX2	-2.07019	0.9364866	0.2381282	TPX2, microtubule-associated, homolog (Xenopus laevis)
UP in Normoxia	**54899**	PXK	-1.393269	0.8896623	0.3807011	PX domain containing serine/threonine
UP in Normoxia	**55039**	TRMT12	-1.324488	0.8374183	0.3992908	tRNA methyltransferase 12 homolog (S. cerevisiae)
UP in Normoxia	**84992**	PIGY	-1.048725	0.8749798	0.4833953	phosphatidylinositol glycan anchor biosynthesis, class Y
UP in Normoxia	**79607**	FAM118	-1.483972	0.8556033	0.3575032	family with sequence similarity 118,
UP in Normoxia	**55664**	CDC37L	-1.0774	0.8546359	0.473882	cell division cycle 37-like 1
UP in Normoxia	**3912**	LAMB1	-1.217329	0.8887396	0.4300783	laminin, beta 1
UP in Normoxia	**6637**	SNRPG	-1.082955	0.883723	0.4720608	small nuclear ribonucleoprotein polypeptide
UP in Normoxia	**80829**	ZFP91	-1.25087	0.889089	0.4201948	ZFP91 zinc finger protein
UP in Normoxia	**7186**	TRAF2	-1.37974	0.8792887	0.3842881	TNF receptor-associated factor 2
UP in Normoxia	**10346**	TRIM22	-1.200294	0.8821374	0.4351867	tripartite motif containing 22
UP in Normoxia	**132228**	LSMEM2	-1.832735	0.8886948	0.2807318	leucine-rich single-pass membrane protein 2
UP in Normoxia	**7965**	AIMP2	-1.842406	0.9198513	0.2788564	aminoacyl tRNA synthetase complex-interacting multifunctional protein 2
UP in Normoxia	**79183**	TTPAL	-1.040758	0.8679746	0.4860721	tocopherol (alpha) transfer protein-like
UP in Normoxia	**51726**	DNAJB1	-1.129853	0.883723	0.4569623	DnaJ (Hsp40) homolog, subfamily B,
UP in Normoxia	**5606**	MAP2K3	-1.155313	0.8851026	0.4489687	mitogen-activated protein kinase kinase 3
UP in Normoxia	**100287932**	TIMM23	-1.38112	0.8974828	0.3839206	translocase of inner mitochondrial membrane 23 homolog (yeast)
UP in Normoxia	**150468**	CKAP2L	-1.307496	0.8855953	0.4040216	cytoskeleton associated protein 2-like
UP in Normoxia	**9875**	URB1	-1.769342	0.9043626	0.2933426	URB1 ribosome biogenesis 1 homolog (S. cerevisiae)
UP in Normoxia	**116238**	TLCD1	-1.8399	0.8792171	0.2793411	TLC domain containing 1
UP in Normoxia	**79465**	ULBP3	-2.592017	0.8777658	0.1658537	UL16 binding protein 3
UP in Normoxia	**23536**	ADAT1	-1.611194	0.9005375	0.3273273	adenosine deaminase, tRNA-specific 1
UP in Normoxia	**9477**	MED20	-1.162646	0.8131595	0.4466926	mediator complex subunit 20
UP in Normoxia	**112479**	ERI2	-1.512668	0.8685837	0.3504625	ERI1 exoribonuclease family member 2
UP in Normoxia	**64801**	ARV1	-1.172425	0.8754994	0.443675	ARV1 homolog (S. cerevisiae)
UP in Normoxia	**10947**	AP3M2	-1.360578	0.878796	0.3894262	adaptor-related protein complex 3, mu 2

UP in Normoxia	**2992**	GYG1	-1.386493	0.8953059	0.3824935	glycogenin 1
UP in Normoxia	**2639**	GCDH	-1.681939	0.8912031	0.3116635	glutaryl-CoA dehydrogenase
UP in Normoxia	**29109**	FHOD1	-2.074277	0.9332975	0.2374545	formin homology 2 domain containing 1
UP in Normoxia	**51567**	TDP2	-1.251318	0.8843053	0.4200643	tyrosyl-DNA phosphodiesterase 2
UP in Normoxia	**25979**	DHRS7B	-1.424763	0.8757144	0.3724806	dehydrogenase/reductase (SDR family) member 7B
UP in Normoxia	**51373**	MRPS17	-1.022896	0.8509988	0.4921274	mitochondrial ribosomal protein S17
UP in Normoxia	**131601**	TPRA1	-1.251154	0.8903968	0.420112	transmembrane protein, adipocyte
UP in Normoxia	**4716**	NDUFB10	-1.024204	0.8786437	0.4916816	NADH dehydrogenase (ubiquinone) 1 beta subcomplex, 10, 22kDa
UP in Normoxia	**221830**	TWISTN	-1.481661	0.8880588	0.3580763	TWIST neighbor
UP in Normoxia	**7520**	XRCC5	-1.484809	0.9016214	0.3572958	X-ray repair complementing defective repair in Chinese hamster cells 5 (double-strand-break rejoining)
UP in Normoxia	**3797**	KIF3C	-1.125484	0.8836066	0.4583482	kinesin family member 3C
UP in Normoxia	**3052**	HCCS	-1.032116	0.8720595	0.4889924	holocytochrome c synthase
UP in Normoxia	**701**	BUB1B	-2.103136	0.933181	0.2327518	BUB1 mitotic checkpoint serine/threonine kinase B
UP in Normoxia	**2491**	CENPI	-2.674647	0.9393532	0.1566214	centromere protein I
UP in Normoxia	**10314**	LANCL1	-1.043011	0.8746126	0.4853136	LanC lantibiotic synthetase component C-like 1 (bacterial)
UP in Normoxia	**9793**	CKAP5	-1.228419	0.8892681	0.426785	cytoskeleton associated protein 5
UP in Normoxia	**328**	APEX1	-1.179062	0.886939	0.4416384	APEX nuclease (multifunctional DNA repair enzyme) 1
UP in Normoxia	**5001**	ORC5	-1.60825	0.9061363	0.327996	origin recognition complex, subunit 5
UP in Normoxia	**9605**	VPS9D1	-1.291655	0.877954	0.4084821	VPS9 domain containing 1
UP in Normoxia	**9833**	MELK	-2.900276	0.9601182	0.133946	maternal embryonic leucine zipper kinase
UP in Normoxia	**51463**	GPR89B	-1.343377	0.8530682	0.3940972	G protein-coupled receptor 89B
UP in Normoxia	**23367**	LARP1	-1.062459	0.8804712	0.4788152	La ribonucleoprotein domain family,
UP in Normoxia	**11274**	USP18	-2.803877	0.9141449	0.1432019	ubiquitin specific peptidase 18
UP in Normoxia	**23760**	PITPNB	-1.075113	0.8801577	0.4746339	phosphatidylinositol transfer protein, beta
UP in Normoxia	**54552**	GNL3L	-1.503433	0.8554869	0.3527132	guanine nucleotide binding protein-like 3 (nucleolar)-like
UP in Normoxia	**2717**	GLA	-1.594311	0.9142793	0.3311804	galactosidase, alpha
UP in Normoxia	**8706**	B3GALNT1	-1.548561	0.8535519	0.3418508	beta-1,3-N-acetylgalactosaminyltransferase 1 (globoside blood group)
UP in Normoxia	**10940**	POP1	-1.596983	0.9003494	0.3305675	processing of precursor 1, ribonuclease P/MRP subunit (S. cerevisiae)
UP in Normoxia	**5202**	PFDN2	-1.109785	0.8853624	0.4633632	prefoldin subunit 2
UP in Normoxia	**29074**	MRPL18	-1.115303	0.8847622	0.4615942	mitochondrial ribosomal protein L18
UP in Normoxia	**57862**	ZNF410	-1.004004	0.873027	0.4986143	zinc finger protein 410
UP in Normoxia	**10199**	MPHOSPH10	-1.261448	0.8839111	0.417125	M-phase phosphoprotein 10 (U3 small nucleolar ribonucleoprotein)
UP in Normoxia	**22929**	SEPHS1	-1.06862	0.8696049	0.4767748	selenophosphate synthetase 1
UP in Normoxia	**55262**	C7orf43	-2.00916	0.9253606	0.2484177	chromosome 7 open reading frame 43
UP in Normoxia	**65258**	MPPE1	-1.203119	0.8599839	0.4343351	metallophosphoesterase 1
UP in Normoxia	**55656**	INTS8	-1.087536	0.8724716	0.4705644	integrator complex subunit 8
UP in Normoxia	**23231**	SEL1L3	-1.08274	0.8803637	0.4721311	sel-1 suppressor of lin-12-like 3 (C. elegans)
UP in Normoxia	**27090**	ST6GALNAC4	-1.673772	0.9063872	0.3134328	ST6 (alpha-N-acetyl-neuraminyl-2,3-beta-galactosyl-1,3)-N-acetylgalactosaminide alpha-2,6-sialyltransferase 4
UP in Normoxia	**7153**	TOP2A	-1.526179	0.9014512	0.3471957	topoisomerase (DNA) II alpha 170kDa
UP in Normoxia	**5860**	QDPR	-1.167547	0.8849055	0.4451776	quinoid dihydropteridine reductase
UP in Normoxia	**55616**	ASAP3	-1.215879	0.835985	0.4305107	ArfGAP with SH3 domain, ankyrin repeat and PH domain 3
UP in Normoxia	**84895**	FAM73B	-1.04563	0.8477022	0.4844334	family with sequence similarity 73, member
UP in Normoxia	**83932**	SPRTN	-1.554002	0.8498343	0.340564	SprT-like N-terminal domain

UP in Normoxia	**4982**	TNFRSF 11B	-2.319151	0.8019708	0.2003854	tumor necrosis factor receptor superfamily, member 11b
UP in Normoxia	**23671**	TMEFF2	-1.537965	0.8257279	0.3443709	transmembrane protein with EGF-like and two follistatin-like domains 2
UP in Normoxia	**56128**	PCDHB8	-2.061401	0.8641584	0.2395833	protocadherin beta 8
UP in Normoxia	**348110**	C15orf38	-1.503168	0.8880677	0.3527778	chromosome 15 open reading frame 38
UP in Normoxia	**79693**	YRDC	-1.151874	0.8792887	0.4500402	yrdC domain containing (E. coli)
UP in Normoxia	**112487**	DTD2	-1.352591	0.8374183	0.3915881	D-tyrosyl-tRNA deacylase 2 (putative)
UP in Normoxia	**122060**	SLAIN1	-1.927695	0.8071128	0.2628488	SLAIN motif family, member 1
UP in Normoxia	**548321**	FAM27A	-1.341991	0.8386814	0.3944759	family with sequence similarity 27, member
UP in Normoxia	**27341**	RRP7A	-1.592214	0.9093165	0.3316622	ribosomal RNA processing 7 homolog A (S. cerevisiae)
UP in Normoxia	**5166**	PDK4	-2.822924	0.8386455	0.1413238	pyruvate dehydrogenase kinase, isozyme 4
UP in Normoxia	**113174**	SAAL1	-1.464569	0.8906656	0.3623438	serum amyloid A-like 1
UP in Normoxia	**3301**	DNAJA1	-1.615173	0.918418	0.3264257	DnaJ (Hsp40) homolog, subfamily A,
UP in Normoxia	**64928**	MRPL14	-1.016538	0.8789662	0.4943012	mitochondrial ribosomal protein L14
UP in Normoxia	**25940**	FAM98A	-1.379379	0.8958882	0.3843842	family with sequence similarity 98, member
UP in Normoxia	**54555**	DDX49	-1.316511	0.8847084	0.4015048	DEAD (Asp-Glu-Ala-Asp) box polypeptide
UP in Normoxia	**6117**	RPA1	-1.143693	0.8794679	0.4525994	replication protein A1, 70kDa
UP in Normoxia	**5426**	POLE	-1.976394	0.9088775	0.2541243	polymerase (DNA directed), epsilon, catalytic subunit
UP in Normoxia	**3148**	HMGB2	-1.249734	0.8883186	0.4205257	high mobility group box 2
UP in Normoxia	**7064**	THOP1	-1.45807	0.8983338	0.3639798	thimet oligopeptidase 1
UP in Normoxia	**85002**	FAM86B	-1.75524	0.8790558	0.296224	family with sequence similarity 86, member
UP in Normoxia	**54965**	PIGX	-1.104012	0.8530861	0.4652209	phosphatidylinositol glycan anchor biosynthesis, class X
UP in Normoxia	**672**	BRCA1	-2.582126	0.9463137	0.1669947	breast cancer 1, early onset
UP in Normoxia	**1746**	DLX2	-1.243824	0.8346144	0.422252	distal-less homeobox 2
UP in Normoxia	**56952**	PRTFDC	-1.318646	0.8718266	0.4009112	phosphoribosyl transferase domain
UP in Normoxia	**93058**	COQ10A	-1.087306	0.8489026	0.4706394	coenzyme Q10 homolog A (S. cerevisiae)
UP in Normoxia	**2107**	ETF1	-1.213374	0.8894562	0.431259	eukaryotic translation termination factor 1
UP in Normoxia	**4130**	MAP1A	-1.41462	0.898504	0.3751086	microtubule-associated protein 1A
UP in Normoxia	**84912**	SLC35B4	-1.376226	0.8898504	0.3852252	solute carrier family 35, member B4
UP in Normoxia	**339487**	ZBTB8O S	-1.054263	0.8754994	0.4815432	zinc finger and BTB domain containing 8 opposite strand
UP in Normoxia	**10069**	RWDD2	-1.35501	0.8856401	0.3909322	RWD domain containing 2B
UP in Normoxia	**2653**	GCSH	-2.115937	0.9306459	0.2306957	glycine cleavage system protein H (aminomethyl carrier)
UP in Normoxia	**55003**	PAK1IP1	-1.817584	0.9108394	0.2836957	PAK1 interacting protein 1
UP in Normoxia	**994**	CDC25B	-1.937605	0.9137239	0.2610494	cell division cycle 25B
UP in Normoxia	**84549**	MAK16	-1.304973	0.8614172	0.4047288	MAK16 homolog (S. cerevisiae)
UP in Normoxia	**55705**	IPO9	-1.205261	0.8784646	0.433691	importin 9
UP in Normoxia	**258010**	SVIP	-1.325142	0.8926274	0.3991098	small VCP/p97-interacting protein
UP in Normoxia	**138199**	C9orf41	-1.155777	0.8633432	0.4488243	chromosome 9 open reading frame 41
UP in Normoxia	**387885**	CCDC42	-1.510282	0.8705097	0.3510427	coiled-coil domain containing 42B
UP in Normoxia	**5718**	PSMD12	-1.0559	0.8810266	0.4809969	proteasome (prosome, macropain) 26S subunit, non-ATPase, 12
UP in Normoxia	**56829**	ZC3HAV	-1.974894	0.9121025	0.2543886	zinc finger CCCH-type, antiviral 1
UP in Normoxia	**80174**	DBF4B	-1.582977	0.8895279	0.3337925	DBF4 homolog B (S. cerevisiae)
UP in Normoxia	**55602**	CDKN2A IP	-1.608507	0.8780167	0.3279376	CDKN2A interacting protein
UP in Normoxia	**57407**	NMRAL	-1.373002	0.8688614	0.386087	NmrA-like family domain containing 1
UP in Normoxia	**6772**	STAT1	-2.031456	0.9345964	0.2446081	signal transducer and activator of transcription 1, 91kDa
UP in Normoxia	**23428**	SLC7A8	-1.217462	0.8363791	0.4300385	solute carrier family 7 (amino acid transporter light chain, L system), member 8

UP in Normoxia	**10813**	UTP14A	-1.308676	0.8802562	0.4036912	UTP14, U3 small nucleolar ribonucleoprotein, homolog A (yeast)
UP in Normoxia	**80723**	SLC35G2	-1.001404	0.8676252	0.4995135	solute carrier family 35, member G2
UP in Normoxia	**63901**	FAM111	-1.818223	0.9148526	0.28357	family with sequence similarity 111,
UP in Normoxia	**5514**	PPP1R10	-1.194819	0.8804712	0.4368412	protein phosphatase 1, regulatory subunit 10
UP in Normoxia	**51360**	MBTPS2	-1.05971	0.8719699	0.4797285	membrane-bound transcription factor peptidase, site 2
UP in Normoxia	**51602**	NOP58	-1.211566	0.8867598	0.4317995	NOP58 ribonucleoprotein
UP in Normoxia	**7374**	UNG	-1.038319	0.8685389	0.4868945	uracil-DNA glycosylase
UP in Normoxia	**836**	CASP3	-1.632132	0.8897519	0.3226112	caspase 3, apoptosis-related cysteine
UP in Normoxia	**28232**	SLCO3A 1	-1.558641	0.8651259	0.3394707	solute carrier organic anion transporter family, member 3A1
UP in Normoxia	**57181**	SLC39A1 0	-1.039084	0.8568575	0.4866362	solute carrier family 39 (zinc transporter), member 10
UP in Normoxia	**9262**	STK17B	-1.022302	0.8510795	0.4923302	serine/threonine kinase 17b
UP in Normoxia	**25929**	GEMIN5	-1.417682	0.8798262	0.3743132	gem (nuclear organelle) associated protein 5
UP in Normoxia	**3980**	LIG3	-1.22668	0.8611574	0.4272997	ligase III, DNA, ATP-dependent
UP in Normoxia	**669**	BPGM	-1.859418	0.9157753	0.2755875	2,3-bisphosphoglycerate mutase
UP in Normoxia	**55093**	WDYHV	-1.013608	0.8493595	0.495306	WDYHV motif containing 1
UP in Normoxia	**22948**	CCT5	-1.741898	0.9214906	0.2989761	chaperonin containing TCP1, subunit 5
UP in Normoxia	**10038**	PARP2	-1.707894	0.9084207	0.3061067	poly (ADP-ribose) polymerase 2
UP in Normoxia	**4832**	NME3	-1.037405	0.8420765	0.4872029	NME/NM23 nucleoside diphosphate kinase
UP in Normoxia	**10171**	RCL1	-1.760469	0.897859	0.2951523	RNA terminal phosphate cyclase-like 1
UP in Normoxia	**5552**	SRGN	-1.130154	0.8781242	0.456867	serglycin
UP in Normoxia	**9894**	TELO2	-1.820759	0.9022306	0.283072	TEL2, telomere maintenance 2, homolog (S. cerevisiae)
UP in Normoxia	**2861**	GPR37	-3.303449	0.922476	0.1012891	G protein-coupled receptor 37 (endothelin receptor type B-like)
UP in Normoxia	**23503**	ZFYVE2	-1.177058	0.8587029	0.4422524	zinc finger, FYVE domain containing 26
UP in Normoxia	**57700**	FAM160 B1	-1.017074	0.8519932	0.4941176	family with sequence similarity 160, member B1
UP in Normoxia	**57406**	ABHD6	-1.724641	0.9050614	0.3025738	abhydrolase domain containing 6
UP in Normoxia	**26263**	FBXO22	-1.105829	0.8747648	0.4646353	F-box protein 22
UP in Normoxia	**29089**	UBE2T	-2.927417	0.9610051	0.1314497	ubiquitin-conjugating enzyme E2T
UP in Normoxia	**8379**	MAD1L1	-1.208479	0.8764042	0.4327247	MAD1 mitotic arrest deficient-like 1 (yeast)
UP in Normoxia	**117246**	FTSJ3	-1.352753	0.8933083	0.3915443	FtsJ homolog 3 (E. coli)
UP in Normoxia	**10717**	AP4B1	-1.642226	0.9054734	0.3203617	adaptor-related protein complex 4, beta 1
UP in Normoxia	**29767**	TMOD2	-1.097975	0.8255935	0.4671717	tropomodulin 2 (neuronal)
UP in Normoxia	**85028**	SNHG12	-1.091746	0.8717549	0.4691933	small nucleolar RNA host gene 12 (non-protein coding)
UP in Normoxia	**65008**	MRPL1	-1.897512	0.8897519	0.2684058	mitochondrial ribosomal protein L1
UP in Normoxia	**5073**	PARN	-1.227306	0.884798	0.4271141	poly(A)-specific ribonuclease
UP in Normoxia	**55353**	LAPTM4	-1.153279	0.8812058	0.4496023	lysosomal protein transmembrane 4 beta
UP in Normoxia	**728833**	FAM72D	-1.671147	0.8788229	0.3140037	family with sequence similarity 72, member
UP in Normoxia	**541468**	LURAP1	-2	0.8396668	0.25	leucine rich adaptor protein 1
UP in Normoxia	**10963**	STIP1	-1.014318	0.8806772	0.4950623	stress-induced-phosphoprotein 1
UP in Normoxia	**65057**	ACD	-1.446397	0.8933799	0.3669367	adrenocortical dysplasia homolog (mouse)
UP in Normoxia	**8540**	AGPS	-1.265524	0.8777031	0.4159483	alkylglycerone phosphate synthase
UP in Normoxia	**55669**	MFN1	-1.031121	0.8699006	0.4893298	mitofusin 1
UP in Normoxia	**219285**	SAMD9L	-1.188337	0.812846	0.4388084	sterile alpha motif domain containing 9-like
UP in Normoxia	**283578**	TMED8	-1.019611	0.8412613	0.4932493	transmembrane emp24 protein transport domain containing 8
UP in Normoxia	**6867**	TACC1	-1.389017	0.8899579	0.3818248	transforming, acidic coiled-coil containing protein 1
UP in Normoxia	**1033**	CDKN3	-1.69677	0.9183822	0.3084759	cyclin-dependent kinase inhibitor 3
UP in Normoxia	**2736**	GLI2	-1.021487	0.8314252	0.4926082	GLI family zinc finger 2
UP in Normoxia	**116496**	FAM129	-1.91473	0.914127	0.2652216	family with sequence similarity 129,

UP in Normoxia	**56623**	INPP5E	-1.106151	0.8599839	0.4645317	inositol polyphosphate-5-phosphatase, 72
UP in Normoxia	**6821**	SUOX	-1.3603	0.8597689	0.3895013	sulfite oxidase
UP in Normoxia	**10460**	TACC3	-2.214635	0.9367285	0.215441	transforming, acidic coiled-coil containing protein 3
UP in Normoxia	**10139**	ARFRP1	-1.330888	0.8859088	0.3975235	ADP-ribosylation factor related protein 1
UP in Normoxia	**8880**	FUBP1	-1.154009	0.8785542	0.4493747	far upstream element (FUSE) binding
UP in Normoxia	**55847**	CISD1	-1.066166	0.8675356	0.4775866	CDGSH iron sulfur domain 1
UP in Normoxia	**6909**	TBX2	-1.267099	0.881797	0.4154945	T-box 2
UP in Normoxia	**201562**	PTPLB	-1.968016	0.914342	0.2556042	protein tyrosine phosphatase-like (proline instead of catalytic arginine), member b
UP in Normoxia	**10733**	PLK4	-2.011477	0.8858103	0.248019	polo-like kinase 4
UP in Normoxia	**121441**	NEDD1	-1.607521	0.8892054	0.3281617	neural precursor cell expressed, developmentally down-regulated 1
UP in Normoxia	**196410**	METTL7	-3.25406	0.9619995	0.1048166	methyltransferase like 7B
UP in Normoxia	**9416**	DDX23	-1.075755	0.8809012	0.4744228	DEAD (Asp-Glu-Ala-Asp) box polypeptide
UP in Normoxia	**6347**	CCL2	-2.200508	0.9140374	0.217561	chemokine (C-C motif) ligand 2
UP in Normoxia	**100506054**	LOC100506054	-1.229713	0.8613903	0.4264024	uncharacterized LOC100506054
UP in Normoxia	**219844**	HYLS1	-2.229913	0.8066828	0.2131716	hydrolethalus syndrome 1
UP in Normoxia	**55677**	IWS1	-1.012244	0.8729195	0.4957746	IWS1 homolog (S. cerevisiae)
UP in Normoxia	**6627**	SNRPA1	-1.149832	0.884789	0.4506776	small nuclear ribonucleoprotein polypeptide
UP in Normoxia	**1059**	CENPB	-1.123541	0.8780256	0.4589661	centromere protein B, 80kDa
UP in Normoxia	**80196**	RNF34	-1.128893	0.8756069	0.4572666	ring finger protein 34, E3 ubiquitin protein
UP in Normoxia	**10615**	SPAG5	-1.576075	0.9144585	0.3353931	sperm associated antigen 5
UP in Normoxia	**9980**	DOPEY2	-1.312723	0.8348293	0.4025605	dopey family member 2
UP in Normoxia	**1984**	EIF5A	-1.243809	0.8929858	0.4222563	eukaryotic translation initiation factor 5A
UP in Normoxia	**5558**	PRIM2	-1.935163	0.9160799	0.2614918	primase, DNA, polypeptide 2 (58kDa)
UP in Normoxia	**84295**	PHF6	-1.055017	0.8657261	0.4812916	PHD finger protein 6
UP in Normoxia	**8863**	PER3	-1.730658	0.8423273	0.3013145	period circadian clock 3
UP in Normoxia	**55273**	TMEM10	-2.496576	0.9105348	0.1771968	transmembrane protein 100
UP in Normoxia	**51755**	CDK12	-1.025798	0.8668548	0.4911387	cyclin-dependent kinase 12
UP in Normoxia	**4719**	NDUFS1	-1.295517	0.8909523	0.4073902	NADH dehydrogenase (ubiquinone) Fe-S protein 1, 75kDa (NADH-coenzyme Q reductase)
UP in Normoxia	**100526740**	ATP5J2-PTCD1	-7.108524	0.8508913	0.0072464	ATP5J2-PTCD1 readthrough
UP in Normoxia	**80173**	IFT74	-1.059599	0.8274209	0.4797654	intraflagellar transport 74 homolog (Chlamydomonas)
UP in Normoxia	**54939**	COMMD	-1.233541	0.8914091	0.4252723	COMM domain containing 4
UP in Normoxia	**3712**	IVD	-1.237249	0.8775956	0.4241808	isovaleryl-CoA dehydrogenase
UP in Normoxia	**3298**	HSF2	-1.169546	0.8728657	0.4445612	heat shock transcription factor 2
UP in Normoxia	**64710**	NUCKS1	-1.066703	0.880086	0.4774088	nuclear casein kinase and cyclin-dependent kinase substrate 1
UP in Normoxia	**112970**	KTI12	-1.520237	0.8895906	0.3486287	KTI12 homolog, chromatin associated (S. cerevisiae)
UP in Normoxia	**165055**	CCDC13	-2.489543	0.8511332	0.1780627	coiled-coil domain containing 138
UP in Normoxia	**51441**	YTHDF2	-1.141838	0.8826032	0.453182	YTH domain family, member 2
UP in Normoxia	**5959**	RDH5	-1.042143	0.8315417	0.4856056	retinol dehydrogenase 5 (11-cis/9-cis)
UP in Normoxia	**26872**	STEAP1	-2.522379	0.9486518	0.1740557	six transmembrane epithelial antigen of the prostate 1
UP in Normoxia	**10437**	IFI30	-2.427969	0.9493147	0.1858269	interferon, gamma-inducible protein 30
UP in Normoxia	**83903**	GSG2	-2.81563	0.9076861	0.14204	germ cell associated 2 (haspin)
UP in Normoxia	**26747**	NUFIP1	-1.321928	0.8371406	0.4	nuclear fragile X mental retardation protein interacting protein 1
UP in Normoxia	**5229**	PGGT1B	-1.114035	0.8430261	0.462	protein geranylgeranyltransferase type I, beta subunit
UP in Normoxia	**8480**	RAE1	-1.010369	0.8774792	0.4964194	RAE1 RNA export 1 homolog (S. pombe)

UP in Normoxia	**2593**	GAMT	-1.018493	0.8740661	0.4936318	guanidinoacetate N-methyltransferase
UP in Normoxia	**81553**	FAM49A	-1.551961	0.8714414	0.3410463	family with sequence similarity 49, member
UP in Normoxia	**148327**	CREB3L4	-1.563967	0.8166174	0.3382199	cAMP responsive element binding protein 3-like 4
UP in Normoxia	**51571**	FAM49B	-1.179685	0.8749619	0.441448	family with sequence similarity 49, member
UP in Normoxia	**9900**	SV2A	-1.272712	0.8879334	0.4138811	synaptic vesicle glycoprotein 2A
UP in Normoxia	**55131**	RBM28	-1.402225	0.8917316	0.3783451	RNA binding motif protein 28
UP in Normoxia	**25855**	BRMS1	-1.582789	0.9161964	0.3338358	breast cancer metastasis suppressor 1
UP in Normoxia	**84105**	PCBD2	-1.317443	0.8161337	0.4012456	pterin-4 alpha-carbinolamine dehydratase/dimerization cofactor of hepatocyte nuclear factor 1 alpha (TCF1) 2
UP in Normoxia	**9677**	PPIP5K1	-1.033305	0.8509988	0.4885896	diphosphoinositol pentakisphosphate kinase
UP in Normoxia	**1876**	E2F6	-1.043226	0.8533459	0.4852412	E2F transcription factor 6
UP in Normoxia	**8208**	CHAF1B	-2.363595	0.9409746	0.1943064	chromatin assembly factor 1, subunit B
UP in Normoxia	**4605**	MYBL2	-4.07338	0.9773896	0.0594005	v-myb myeloblastosis viral oncogene homolog (avian)-like 2
UP in Normoxia	**509**	ATP5C1	-1.036857	0.8816268	0.4873882	ATP synthase, H+ transporting, mitochondrial F1 complex, gamma polypeptide 1
UP in Normoxia	**8601**	RGS20	-1.283868	0.889286	0.410693	regulator of G-protein signaling 20
UP in Normoxia	**51096**	UTP18	-1.265942	0.8906656	0.4158278	UTP18 small subunit (SSU) processome component homolog (yeast)
UP in Normoxia	**51495**	PTPLAD1	-1.594575	0.9156678	0.3311198	protein tyrosine phosphatase-like A domain containing 1
UP in Normoxia	**79172**	CENPO	-2.172112	0.9315238	0.2218856	centromere protein O
UP in Normoxia	**9404**	LPXN	-1.189667	0.8878079	0.438404	leupaxin
UP in Normoxia	**3945**	LDHB	-1.042116	0.8824778	0.4856146	lactate dehydrogenase B
UP in Normoxia	**83401**	ELOVL3	-1.908813	0.8202544	0.2663116	ELOVL fatty acid elongase 3
UP in Normoxia	**51163**	DBR1	-1.54763	0.8582729	0.3420716	debranching enzyme homolog 1 (S.
UP in Normoxia	**9975**	NR1D2	-1.118439	0.8760459	0.460592	nuclear receptor subfamily 1, group D,
UP in Normoxia	**10007**	GNPDA1	-1.375239	0.8962465	0.3854887	glucosamine-6-phosphate deaminase 1
UP in Normoxia	**25885**	POLR1A	-1.989221	0.9144226	0.2518748	polymerase (RNA) I polypeptide A, 194kDa
UP in Normoxia	**127396**	ZNF684	-2.446256	0.8141629	0.1834862	zinc finger protein 684
UP in Normoxia	**54107**	POLE3	-1.312027	0.8912837	0.4027545	polymerase (DNA directed), epsilon 3, accessory subunit
UP in Normoxia	**2764**	GMFB	-1.081213	0.8802293	0.4726312	glia maturation factor, beta
UP in Normoxia	**64105**	CENPK	-2.524489	0.9424975	0.1738014	centromere protein K
UP in Normoxia	**54443**	ANLN	-2.178978	0.936836	0.2208321	anillin, actin binding protein
UP in Normoxia	**92106**	OXNAD1	-1.631068	0.84626	0.3228492	oxidoreductase NAD-binding domain containing 1
UP in Normoxia	**2072**	ERCC4	-1.033407	0.8069605	0.4885551	excision repair cross-complementing rodent repair deficiency, complementation group 4
UP in Normoxia	**11097**	NUPL2	-1.149952	0.8693362	0.4506401	nucleoporin like 2
UP in Normoxia	**84135**	UTP15	-2.096862	0.9232464	0.2337662	UTP15, U3 small nucleolar ribonucleoprotein, homolog (S. cerevisiae)
UP in Normoxia	**729991**	MEF2BN	-1.261693	0.878375	0.4170543	MEF2B neighbor
UP in Normoxia	**25994**	HIGD1A	-1.005387	0.8772911	0.4981365	HIG1 hypoxia inducible domain family, member 1A
UP in Normoxia	**26275**	HIBCH	-1.040157	0.859742	0.4862745	3-hydroxyisobutyryl-CoA hydrolase
UP in Normoxia	**84188**	FAR1	-1.11432	0.8780077	0.4619088	fatty acyl CoA reductase 1
UP in Normoxia	**100129405**	MSTO2P	-1.153979	0.8425871	0.449384	misato homolog 2 pseudogene
UP in Normoxia	**8507**	ENC1	-2.0568	0.9305384	0.2403485	ectodermal-neural cortex 1 (with BTB
UP in Normoxia	**55922**	NKRF	-1.008922	0.8180417	0.4969174	NFKB repressing factor
UP in Normoxia	**26240**	FAM50B	-1.756941	0.8023112	0.2958748	family with sequence similarity 50, member
UP in Normoxia	**10915**	TCERG1	-1.060493	0.8749261	0.4794682	transcription elongation regulator 1
UP in Normoxia	**401541**	CENPP	-1.213466	0.8400878	0.4312312	centromere protein P
UP in Normoxia	**11065**	UBE2C	-1.435262	0.9004031	0.3697797	ubiquitin-conjugating enzyme E2C

UP in Normoxia	**6574**	SLC20A1	-1.271751	0.8932903	0.4141568	solute carrier family 20 (phosphate transporter), member 1
UP in Normoxia	**1806**	DPYD	-1.820652	0.8176028	0.2830931	dihydropyrimidine dehydrogenase
UP in Normoxia	**4013**	VWA5A	-1.598897	0.900421	0.3301293	von Willebrand factor A domain containing
UP in Normoxia	**4254**	KITLG	-3.020716	0.9090836	0.1232179	KIT ligand
UP in Normoxia	**51263**	MRPL30	-1.206884	0.8762071	0.4332033	mitochondrial ribosomal protein L30
UP in Normoxia	**80308**	FLAD1	-1.705965	0.9146556	0.3065161	flavin adenine dinucleotide synthetase 1
UP in Normoxia	**5983**	RFC3	-3.169435	0.9661023	0.1111489	replication factor C (activator 1) 3, 38kDa
UP in Normoxia	**54433**	GAR1	-1.301496	0.8678133	0.4057052	GAR1 ribonucleoprotein homolog (yeast)
UP in Normoxia	**90957**	DHX57	-1.172764	0.860163	0.4435706	DEAH (Asp-Glu-Ala-Asp/His) box polypeptide 57
UP in Normoxia	**8438**	RAD54L	-2.768434	0.9440383	0.1467636	RAD54-like (S. cerevisiae)
UP in Normoxia	**1468**	SLC25A10	-2.031547	0.9274926	0.2445927	solute carrier family 25 (mitochondrial carrier; dicarboxylate transporter), member 10
UP in Normoxia	**22853**	LMTK2	-1.303965	0.8488489	0.4050117	lemur tyrosine kinase 2
UP in Normoxia	**9245**	GCNT3	-2.681178	0.9058945	0.155914	glucosaminyl (N-acetyl) transferase 3, mucin type
UP in Normoxia	**54977**	SLC25A3	-1.062637	0.8744065	0.4787562	solute carrier family 25, member 38
UP in Normoxia	**705**	BYSL	-1.921251	0.9115023	0.2640254	bystin-like
UP in Normoxia	**51699**	VPS29	-1.016736	0.8786885	0.4942332	vacuolar protein sorting 29 homolog (S. cerevisiae)
UP in Normoxia	**90379**	DCAF15	-1.189825	0.8791006	0.4383562	DDB1 and CUL4 associated factor 15
UP in Normoxia	**8930**	MBD4	-1.055867	0.8718803	0.481008	methyl-CpG binding domain protein 4
UP in Normoxia	**10385**	BTN2A2	-1.911944	0.8524053	0.2657343	butyrophilin, subfamily 2, member A2
UP in Normoxia	**84319**	CMSS1	-1.651878	0.9145391	0.3182255	cms1 ribosomal small subunit homolog
UP in Normoxia	**4968**	OGG1	-1.432823	0.8880408	0.3704055	8-oxoguanine DNA glycosylase
UP in Normoxia	**8034**	SLC25A16	-1.188488	0.8575652	0.4387624	solute carrier family 25 (mitochondrial carrier; Graves disease autoantigen), member 16
UP in Normoxia	**4726**	NDUFS6	-1.244471	0.8922601	0.4220625	NADH dehydrogenase (ubiquinone) Fe-S protein 6, 13kDa (NADH-coenzyme Q reductase)
UP in Normoxia	**51593**	SRRT	-1.456687	0.8981188	0.3643289	serrate RNA effector molecule homolog (Arabidopsis)
UP in Normoxia	**80267**	EDEM3	-1.094651	0.8298486	0.4682493	ER degradation enhancer, mannosidase alpha-like 3
UP in Normoxia	**10514**	MYBBP1A	-1.968855	0.9193138	0.2554557	MYB binding protein (P160) 1a
UP in Normoxia	**27349**	MCAT	-1.212393	0.8700887	0.4315523	malonyl CoA:ACP acyltransferase (mitochondrial)
UP in Normoxia	**11022**	TDRKH	-1.330313	0.8703843	0.3976819	tudor and KH domain containing
UP in Normoxia	**7386**	UQCRFS1	-1.054232	0.880946	0.4815536	ubiquinol-cytochrome c reductase, Rieske iron-sulfur polypeptide 1
UP in Normoxia	**51155**	HN1	-1.363085	0.8956642	0.38875	hematological and neurological expressed 1
UP in Normoxia	**153443**	SRFBP1	-1.331997	0.8592583	0.3972179	serum response factor binding protein 1
UP in Normoxia	**2618**	GART	-1.933784	0.9223775	0.2617417	phosphoribosylglycinamide formyl-transferase, phosphoribosylglycinamide synthetase, phosphoribosyl-aminoimidazole synthetase
UP in Normoxia	**29893**	PSMC3IP	-1.577579	0.9141091	0.3350436	PSMC3 interacting protein
UP in Normoxia	**8453**	CUL2	-1.089103	0.8773179	0.4700537	cullin 2
UP in Normoxia	**51110**	LACTB2	-2.330645	0.8843143	0.1987952	lactamase, beta 2
UP in Normoxia	**7290**	HIRA	-1.056755	0.8696318	0.4807122	HIR histone cell cycle regulation defective homolog A (S. cerevisiae)
UP in Normoxia	**10328**	EMC8	-1.424453	0.8904596	0.3725606	ER membrane protein complex subunit 8
UP in Normoxia	**6700**	SPRR2A	-2.159904	0.8944728	0.2237711	small proline-rich protein 2A

UP in Normoxia	**56852**	RAD18	-1.248388	0.8726059	0.4209184	RAD18 homolog (S. cerevisiae)
UP in Normoxia	**5198**	PFAS	-2.711184	0.949037	0.1527046	phosphoribosylformylglycinamidine
UP in Normoxia	**211**	ALAS1	-1.213781	0.8890173	0.4311371	aminolevulinate, delta-, synthase 1
UP in Normoxia	**10244**	RABEPK	-1.2548	0.8817433	0.4190515	Rab9 effector protein with kelch motifs
UP in Normoxia	**9692**	KIAA039	-1.315844	0.8729822	0.4016906	KIAA0391
UP in Normoxia	**29105**	C16orf80	-1.11757	0.8763863	0.4608696	chromosome 16 open reading frame 80
UP in Normoxia	**78995**	C17orf53	-1.108636	0.8279764	0.4637322	chromosome 17 open reading frame 53
UP in Normoxia	**64859**	NABP1	-2.152046	0.9336827	0.2249933	nucleic acid binding protein 1
UP in Normoxia	**1741**	DLG3	-1.280819	0.8145391	0.4115617	discs, large homolog 3 (Drosophila)
UP in Normoxia	**8975**	USP13	-2.314264	0.9029024	0.2010652	ubiquitin specific peptidase 13 (isopeptidase T-3)
UP in Normoxia	**89958**	SAPCD2	-2.399514	0.9298934	0.1895283	suppressor APC domain containing 2
UP in Normoxia	**80021**	TMEM62	-1.664816	0.880946	0.3153846	transmembrane protein 62
UP in Normoxia	**122769**	LRR1	-1.935711	0.9103288	0.2613924	leucine rich repeat protein 1
UP in Normoxia	**2966**	GTF2H2	-1.843751	0.8779092	0.2785965	general transcription factor IIH, polypeptide 2, 44kDa
UP in Normoxia	**54957**	TXNL4B	-1.05534	0.8540267	0.4811838	thioredoxin-like 4B
UP in Normoxia	**4173**	MCM4	-2.369871	0.9470931	0.193463	minichromosome maintenance complex component 4
UP in Normoxia	**6427**	SRSF2	-1.417876	0.8998208	0.3742629	serine/arginine-rich splicing factor 2
UP in Normoxia	**1646**	AKR1C2	-2.200482	0.9133566	0.2175649	aldo-keto reductase family 1, member C2
UP in Normoxia	**4286**	MITF	-2.376492	0.9105706	0.192577	microphthalmia-associated transcription
UP in Normoxia	**195828**	ZNF367	-2.882987	0.9421123	0.1355609	zinc finger protein 367
UP in Normoxia	**7301**	TYRO3	-1.279885	0.884807	0.4118283	TYRO3 protein tyrosine kinase
UP in Normoxia	**51702**	PADI3	-2.22097	0.829759	0.214497	peptidyl arginine deiminase, type III
UP in Normoxia	**26050**	SLITRK5	-1.563607	0.8782317	0.3383042	SLIT and NTRK-like family, member 5
UP in Normoxia	**91942**	NDUFAF 2	-1.324614	0.8937562	0.3992559	NADH dehydrogenase (ubiquinone) complex I, assembly factor 2
UP in Normoxia	**55652**	SLC48A1	-1.247093	0.8458121	0.4212963	solute carrier family 48 (heme transporter), member 1
UP in Normoxia	**6491**	STIL	-1.959906	0.9034758	0.2570453	SCL/TAL1 interrupting locus
UP in Normoxia	**55612**	FERMT1	-1.820366	0.8106781	0.2831492	fermitin family member 1
UP in Normoxia	**5933**	RBL1	-1.885933	0.9117979	0.2705687	retinoblastoma-like 1 (p107)
UP in Normoxia	**22847**	ZNF507	-1.385923	0.8283884	0.3826446	zinc finger protein 507
UP in Normoxia	**274**	BIN1	-1.162589	0.8847174	0.4467101	bridging integrator 1
UP in Normoxia	**54821**	ERCC6L	-2.451749	0.9168951	0.1827889	excision repair cross-complementing rodent repair deficiency, complementation group 6-like
UP in Normoxia	**7407**	VARS	-1.475962	0.8979486	0.3594937	valyl-tRNA synthetase
UP in Normoxia	**113444**	SMIM12	-1.106866	0.8492699	0.4643016	small integral membrane protein 12
UP in Normoxia	**8323**	FZD6	-1.435285	0.8830422	0.3697739	frizzled family receptor 6
UP in Normoxia	**53**	ACP2	-1.075228	0.8761623	0.4745962	acid phosphatase 2, lysosomal
UP in Normoxia	**5591**	PRKDC	-1.546946	0.9018902	0.3422338	protein kinase, DNA-activated, catalytic polypeptide
UP in Normoxia	**5905**	RANGA	-2.084673	0.9372839	0.2357496	Ran GTPase activating protein 1
UP in Normoxia	**51073**	MRPL4	-1.308867	0.8924662	0.4036379	mitochondrial ribosomal protein L4
UP in Normoxia	**53981**	CPSF2	-1.317682	0.8916599	0.4011789	cleavage and polyadenylation specific factor 2, 100kDa
UP in Normoxia	**9183**	ZW10	-1.018029	0.8352414	0.4937904	zw10 kinetochore protein
UP in Normoxia	**79165**	LENG1	-1.217392	0.8660665	0.4300595	leukocyte receptor cluster (LRC) member 1
UP in Normoxia	**4124**	MAN2A1	-1.053427	0.8681	0.4818223	mannosidase, alpha, class 2A, member 1
UP in Normoxia	**54332**	GDAP1	-1.434895	0.8754367	0.3698738	ganglioside induced differentiation associated protein 1
UP in Normoxia	**1184**	CLCN5	-2.36995	0.8430171	0.1934524	chloride channel, voltage-sensitive 5
UP in Normoxia	**7468**	WHSC1	-1.254562	0.888014	0.4191209	Wolf-Hirschhorn syndrome candidate 1
UP in Normoxia	**5393**	EXOSC9	-2.070039	0.9306907	0.2381531	exosome component 9
UP in Normoxia	**147007**	TMEM19	-1.420793	0.8949476	0.3735071	transmembrane protein 199

UP in Normoxia	**57580**	PREX1	-1.07108	0.8819224	0.4759624	phosphatidylinositol-3,4,5-trisphosphate-dependent Rac exchange factor 1
UP in Normoxia	**5889**	RAD51C	-1.088949	0.8755084	0.4701038	RAD51 homolog C (S. cerevisiae)
UP in Normoxia	**25804**	LSM4	-1.182201	0.888005	0.4406788	LSM4 homolog, U6 small nuclear RNA associated (S. cerevisiae)
UP in Normoxia	**339201**	ASB16-AS1	-1.369444	0.8540536	0.3870403	ASB16 antisense RNA 1
UP in Normoxia	**1798**	DPAGT1	-1.144977	0.872597	0.452197	dolichyl-phosphate (UDP-N-acetyl-glucosamine) N-acetylglucosamine-phosphotransferase 1 (GlcNAc-1-P transferase)
UP in Normoxia	**64093**	SMOC1	-2.658654	0.9517872	0.1583672	SPARC related modular calcium binding 1
UP in Normoxia	**9879**	DDX46	-1.35737	0.8911672	0.3902932	DEAD (Asp-Glu-Ala-Asp) box polypeptide
UP in Normoxia	**5496**	PPM1G	-1.539628	0.9031174	0.3439742	protein phosphatase, Mg2+/Mn2+ dependent, 1G
UP in Normoxia	**9031**	BAZ1B	-1.23816	0.8893487	0.423913	bromodomain adjacent to zinc finger
UP in Normoxia	**146956**	EME1	-1.653499	0.903207	0.3178683	essential meiotic endonuclease 1 homolog 1 (S. pombe)
UP in Normoxia	**55034**	MOCOS	-1.928188	0.8965242	0.262759	molybdenum cofactor sulfurase
UP in Normoxia	**11163**	NUDT4	-1.012893	0.8412613	0.4955516	nudix (nucleoside diphosphate linked moiety X)-type motif 4
UP in Normoxia	**51729**	WBP11	-1.032177	0.8785004	0.4889719	WW domain binding protein 11
UP in Normoxia	**10477**	UBE2E3	-1.323646	0.8957449	0.399524	ubiquitin-conjugating enzyme E2E 3
UP in Normoxia	**284266**	SIGLEC1	-1.713431	0.9195646	0.3049341	sialic acid binding Ig-like lectin 15
UP in Normoxia	**54503**	ZDHHC1	-1.862636	0.9118606	0.2749734	zinc finger, DHHC-type containing 13
UP in Normoxia	**283951**	C16orf91	-1.170688	0.8453194	0.4442094	chromosome 16 open reading frame 91
UP in Normoxia	**253714**	MMS22L	-1.8895	0.886285	0.2699005	MMS22-like, DNA repair protein
UP in Normoxia	**1955**	MEGF9	-1.950858	0.8719878	0.2586624	multiple EGF-like-domains 9
UP in Normoxia	**95681**	CEP41	-1.118818	0.852898	0.460471	centrosomal protein 41kDa
UP in Normoxia	**5866**	RAB3IL1	-1.161794	0.8381797	0.4469565	RAB3A interacting protein (rabin3)-like 1
UP in Normoxia	**5293**	PIK3CD	-1.001956	0.8677685	0.4993226	phosphatidylinositol-4,5-bisphosphate 3-kinase, catalytic subunit delta
UP in Normoxia	**10486**	CAP2	-1.167416	0.8523963	0.4452182	CAP, adenylate cyclase-associated protein, 2 (yeast)
UP in Normoxia	**8924**	HERC2	-1.596173	0.9027681	0.3307532	HECT and RLD domain containing E3 ubiquitin protein ligase 2
UP in Normoxia	**5601**	MAPK9	-1.364816	0.8896892	0.3882841	mitogen-activated protein kinase 9
UP in Normoxia	**993**	CDC25A	-4.570699	0.9676969	0.0420807	cell division cycle 25A
UP in Normoxia	**59271**	EVA1C	-1.455411	0.8895996	0.3646512	eva-1 homolog C (C. elegans)
UP in Normoxia	**9136**	RRP9	-2.221606	0.9358864	0.2144026	ribosomal RNA processing 9, small subunit (SSU) processome component, homolog (yeast)
UP in Normoxia	**7804**	LRP8	-2.54781	0.9405088	0.1710145	low density lipoprotein receptor-related protein 8, apolipoprotein e receptor
UP in Normoxia	**55111**	PLEKHJ1	-1.501125	0.8993371	0.3532779	pleckstrin homology domain containing, family J member 1
UP in Normoxia	**55319**	TMA16	-1.24209	0.8485085	0.4227598	translation machinery associated 16 homolog (S. cerevisiae)
UP in Normoxia	**79833**	GEMIN6	-1.879629	0.9183374	0.2717537	gem (nuclear organelle) associated protein 6
UP in Normoxia	**3399**	ID3	-3.515443	0.9743169	0.0874473	inhibitor of DNA binding 3, dominant negative helix-loop-helix protein
UP in Normoxia	**3575**	IL7R	-1.864068	0.8743617	0.2747006	interleukin 7 receptor
UP in Normoxia	**3159**	HMGA1	-1.557877	0.9042641	0.3396505	high mobility group AT-hook 1
UP in Normoxia	**10561**	IFI44	-2.151546	0.9304757	0.2250712	interferon-induced protein 44
UP in Normoxia	**8544**	PIR	-1.348168	0.8491445	0.3927906	pirin (iron-binding nuclear protein)
UP in Normoxia	**2035**	EPB41	-1.275294	0.8565439	0.4131409	erythrocyte membrane protein band 4.1 (elliptocytosis 1, RH-linked)

UP in Normoxia	**55278**	QRSL1	-1.596344	0.8996865	0.330714	glutaminyl-tRNA synthase (glutamine-hydrolyzing)-like 1
UP in Normoxia	**3028**	HSD17B	-1.380283	0.8976978	0.3841434	hydroxysteroid (17-beta) dehydrogenase 10
UP in Normoxia	**3978**	LIG1	-1.672145	0.8982084	0.3137864	ligase I, DNA, ATP-dependent
UP in Normoxia	**51735**	RAPGEF	-1.107219	0.8374272	0.4641881	Rap guanine nucleotide exchange factor
UP in Normoxia	**117178**	SSX2IP	-1.069835	0.8528442	0.4763734	synovial sarcoma, X breakpoint 2 interacting protein
UP in Normoxia	**57620**	STIM2	-1.04145	0.8066918	0.4858388	stromal interaction molecule 2
UP in Normoxia	**256586**	LYSMD2	-2.27572	0.9102034	0.2065095	LysM, putative peptidoglycan-binding, domain containing 2
UP in Normoxia	**617**	BCS1L	-1.255668	0.8816358	0.4187997	BC1 (ubiquinol-cytochrome c reductase) synthesis-like
UP in Normoxia	**6932**	TCF7	-1.069707	0.8765654	0.4764157	transcription factor 7 (T-cell specific, HMG-
UP in Normoxia	**65109**	UPF3B	-1.151095	0.8664517	0.4502832	UPF3 regulator of nonsense transcripts homolog B (yeast)
UP in Normoxia	**22937**	SCAP	-1.003183	0.8708322	0.498898	SREBF chaperone
UP in Normoxia	**10440**	TIMM17 A	-1.295599	0.892287	0.4073671	translocase of inner mitochondrial membrane 17 homolog A (yeast)
UP in Normoxia	**100128191**	TMPO-AS1	-2.635462	0.8779181	0.1609337	TMPO antisense RNA 1
UP in Normoxia	**26147**	PHF19	-1.667242	0.9064947	0.3148547	PHD finger protein 19
UP in Normoxia	**23567**	ZNF346	-1.217109	0.8113052	0.4301439	zinc finger protein 346
UP in Normoxia	**64794**	DDX31	-1.282367	0.8642659	0.4111205	DEAD (Asp-Glu-Ala-Asp) box polypeptide
UP in Normoxia	**79657**	RPAP3	-1.324707	0.8812954	0.3992301	RNA polymerase II associated protein 3
UP in Normoxia	**7203**	CCT3	-1.251966	0.8930126	0.4198757	chaperonin containing TCP1, subunit 3
UP in Normoxia	**387921**	NHLRC3	-1.056714	0.8527815	0.4807256	NHL repeat containing 3
UP in Normoxia	**9212**	AURKB	-2.376183	0.9460808	0.1926184	aurora kinase B
UP in Normoxia	**327**	APEH	-1.244547	0.890361	0.4220405	N-acylaminoacyl-peptide hydrolase
UP in Normoxia	**3214**	HOXB4	-1.385205	0.8126937	0.3828351	homeobox B4
UP in Normoxia	**5833**	PCYT2	-1.747535	0.9105975	0.2978102	phosphate cytidylyltransferase 2,
UP in Normoxia	**5422**	POLA1	-1.985916	0.8862492	0.2524526	polymerase (DNA directed), alpha 1, catalytic subunit
UP in Normoxia	**1633**	DCK	-1.365079	0.8799875	0.3882133	deoxycytidine kinase
UP in Normoxia	**51377**	UCHL5	-1.503922	0.8844845	0.3525936	ubiquitin carboxyl-terminal hydrolase L5
UP in Normoxia	**79801**	SHCBP1	-2.389797	0.9456329	0.1908092	SHC SH2-domain binding protein 1
UP in Normoxia	**6950**	TCP1	-1.293263	0.8944818	0.4080271	t-complex 1
UP in Normoxia	**2983**	GUCY1B	-3.692395	0.9323121	0.0773532	guanylate cyclase 1, soluble, beta 3
UP in Normoxia	**5711**	PSMD5	-1.003987	0.8660754	0.4986203	proteasome (prosome, macropain) 26S subunit, non-ATPase, 5
UP in Normoxia	**10276**	NET1	-1.505378	0.8837857	0.3522378	neuroepithelial cell transforming 1
UP in Normoxia	**221150**	SKA3	-2.07227	0.8854699	0.237785	spindle and kinetochore associated complex subunit 3
UP in Normoxia	**3182**	HNRNP AB	-2.562437	0.9500851	0.1692894	heterogeneous nuclear ribonucleoprotein A/B
UP in Normoxia	**955**	ENTPD6	-1.309585	0.8805429	0.4034368	ectonucleoside triphosphate diphosphohydrolase 6 (putative)
UP in Normoxia	**190**	NR0B1	-1.494899	0.8678939	0.3548057	nuclear receptor subfamily 0, group B,
UP in Normoxia	**124976**	SPNS2	-1.642544	0.8483741	0.3202912	spinster homolog 2 (Drosophila)
UP in Normoxia	**56252**	YLPM1	-1.007038	0.8636478	0.4975669	YLP motif containing 1
UP in Normoxia	**63892**	THADA	-1.014088	0.8415928	0.4951413	thyroid adenoma associated
UP in Normoxia	**84970**	C1orf94	-1.135117	0.8815193	0.455298	chromosome 1 open reading frame 94
UP in Normoxia	**123811**	FOPNL	-1.089445	0.8771477	0.4699423	FGFR1OP N-terminal like
UP in Normoxia	**113251**	LARP4	-1.757479	0.9161426	0.2957646	La ribonucleoprotein domain family,
UP in Normoxia	**9618**	TRAF4	-1.093356	0.856535	0.4686698	TNF receptor-associated factor 4
UP in Normoxia	**5993**	RFX5	-1.525253	0.8950103	0.3474187	regulatory factor X, 5 (influences HLA class II expression)

UP in Normoxia	**5431**	POLR2B	-1.087482	0.8793783	0.4705819	polymerase (RNA) II (DNA directed) polypeptide B, 140kDa
UP in Normoxia	**79915**	ATAD5	-2.476737	0.8363702	0.1796502	ATPase family, AAA domain containing 5
UP in Normoxia	**121053**	C12orf45	-1.053428	0.869381	0.481822	chromosome 12 open reading frame 45
UP in Normoxia	**25788**	RAD54B	-2.098163	0.9177551	0.2335554	RAD54 homolog B (S. cerevisiae)
UP in Normoxia	**9391**	CIAO1	-1.0921	0.8810356	0.4690782	cytosolic iron-sulfur protein assembly 1
UP in Normoxia	**10926**	DBF4	-2.030818	0.919269	0.2447164	DBF4 homolog (S. cerevisiae)
UP in Normoxia	**9837**	GINS1	-2.985989	0.9313446	0.1262199	GINS complex subunit 1 (Psf1 homolog)
UP in Normoxia	**4487**	MSX1	-1.151866	0.8029204	0.4500427	msh homeobox 1
UP in Normoxia	**4678**	NASP	-1.478064	0.8987459	0.3589702	nuclear autoantigenic sperm protein (histone-binding)
UP in Normoxia	**222236**	NAPEPL D	-1.438324	0.8429902	0.3689956	N-acyl phosphatidylethanolamine phospholipase D
UP in Normoxia	**3304**	HSPA1B	-1.936077	0.9240079	0.261326	heat shock 70kDa protein 1B
UP in Normoxia	**56243**	KIAA121	-1.488663	0.8928156	0.3563425	KIAA1217
UP in Normoxia	**4361**	MRE11A	-1.009558	0.802132	0.4966985	MRE11 meiotic recombination 11 homolog A (S. cerevisiae)
UP in Normoxia	**51474**	LIMA1	-1.77682	0.9201111	0.291826	LIM domain and actin binding 1
UP in Normoxia	**389136**	VGLL3	-1.749936	0.8786796	0.297315	vestigial like 3 (Drosophila)
UP in Normoxia	**83743**	GRWD1	-2.051084	0.9196542	0.2413027	glutamate-rich WD repeat containing 1
UP in Normoxia	**3936**	LCP1	-1.049002	0.842193	0.4833024	lymphocyte cytosolic protein 1 (L-plastin)
UP in Normoxia	**58526**	MID1IP1	-1.359358	0.8947236	0.3897558	MID1 interacting protein 1
UP in Normoxia	**171568**	POLR3H	-1.313847	0.8874586	0.4022468	polymerase (RNA) III (DNA directed) polypeptide H (22.9kD)
UP in Normoxia	**30849**	PIK3R4	-1.361603	0.8811251	0.3891496	phosphoinositide-3-kinase, regulatory
UP in Normoxia	**2274**	FHL2	-1.04844	0.8817074	0.4834908	four and a half LIM domains 2
UP in Normoxia	**3239**	HOXD13	-1.115993	0.8167697	0.4613734	homeobox D13
UP in Normoxia	**25909**	AHCTF1	-1.211216	0.8810445	0.4319045	AT hook containing transcription factor 1
UP in Normoxia	**5825**	ABCD3	-1.226801	0.8777569	0.4272637	ATP-binding cassette, sub-family D (ALD), member 3
UP in Normoxia	**23397**	NCAPH	-2.49445	0.9398638	0.177458	non-SMC condensin I complex, subunit H
UP in Normoxia	**55718**	POLR3E	-2.0621	0.9256383	0.2394672	polymerase (RNA) III (DNA directed) polypeptide E (80kD)
UP in Normoxia	**5536**	PPP5C	-1.018958	0.874729	0.4934726	protein phosphatase 5, catalytic subunit
UP in Normoxia	**2710**	GK	-1.650551	0.877936	0.3185185	glycerol kinase
UP in Normoxia	**200916**	RPL22L1	-1.914468	0.9235779	0.2652697	ribosomal protein L22-like 1
UP in Normoxia	**55906**	ZC4H2	-1.004817	0.8259876	0.4983333	zinc finger, C4H2 domain containing
UP in Normoxia	**91544**	UBXN11	-1.583375	0.8974828	0.3337004	UBX domain protein 11
UP in Normoxia	**54865**	GPATCH	-2.774746	0.9538923	0.1461229	G patch domain containing 4
UP in Normoxia	**1019**	CDK4	-1.157922	0.8877452	0.4481576	cyclin-dependent kinase 4
UP in Normoxia	**606**	NBEAP1	-1.003876	0.8532653	0.4986586	neurobeachin pseudogene 1
UP in Normoxia	**53343**	NUDT9	-1.10358	0.8173609	0.4653604	nudix (nucleoside diphosphate linked moiety X)-type motif 9
UP in Normoxia	**708**	C1QBP	-1.552665	0.9036012	0.3408798	complement component 1, q subcomponent binding protein
UP in Normoxia	**11146**	GLMN	-1.782409	0.8631819	0.2906977	glomulin, FKBP associated protein
UP in Normoxia	**80777**	CYB5B	-1.192343	0.8852817	0.4375916	cytochrome b5 type B (outer mitochondrial membrane)
UP in Normoxia	**55020**	TTC38	-1.078625	0.8213384	0.4734799	tetratricopeptide repeat domain 38
UP in Normoxia	**54534**	MRPL50	-1.234977	0.8859446	0.4248492	mitochondrial ribosomal protein L50
UP in Normoxia	**142**	PARP1	-1.582262	0.9149601	0.3339579	poly (ADP-ribose) polymerase 1
UP in Normoxia	**79947**	DHDDS	-1.081293	0.8752038	0.4726049	dehydrodolichyl diphosphate synthase
UP in Normoxia	**51649**	MRPS23	-1.087243	0.881161	0.47066	mitochondrial ribosomal protein S23
UP in Normoxia	**729440**	CCDC61	-1.047816	0.8033593	0.4836998	coiled-coil domain containing 61
UP in Normoxia	**83596**	BCL2L12	-1.155915	0.8779002	0.4487814	BCL2-like 12 (proline rich)
UP in Normoxia	**55143**	CDCA8	-1.8144	0.9184628	0.2843225	cell division cycle associated 8

UP in Normoxia	**284293**	HMSD	-1.987791	0.8932993	0.2521246	histocompatibility (minor) serpin domain containing
UP in Normoxia	**9941**	EXOG	-1.956391	0.891642	0.2576723	endo/exonuclease (5'-3'), endonuclease G-
UP in Normoxia	**51081**	MRPS7	-1.069034	0.8807668	0.476638	mitochondrial ribosomal protein S7
UP in Normoxia	**22943**	DKK1	-2.606464	0.8555765	0.1642012	dickkopf 1 homolog (Xenopus laevis)
UP in Normoxia	**10574**	CCT7	-1.041664	0.8821195	0.485767	chaperonin containing TCP1, subunit 7 (eta)
UP in Normoxia	**11168**	PSIP1	-1.301225	0.8883723	0.4057816	PC4 and SFRS1 interacting protein 1
UP in Normoxia	**7272**	TTK	-2.069725	0.9193407	0.2382048	TTK protein kinase
UP in Normoxia	**25847**	ANAPC1	-1.757359	0.9203261	0.2957891	anaphase promoting complex subunit 13
UP in Normoxia	**51075**	TMX2	-1.040316	0.8809191	0.4862209	thioredoxin-related transmembrane protein 2
UP in Normoxia	**91750**	LIN52	-1.450809	0.86552	0.3658163	lin-52 homolog (C. elegans)
UP in Normoxia	**4856**	NOV	-2.185093	0.9278061	0.2198981	nephroblastoma overexpressed
UP in Normoxia	**26973**	CHORD C1	-1.689932	0.9055361	0.3099415	cysteine and histidine-rich domain (CHORD) containing 1
UP in Normoxia	**26108**	PYGO1	-1.461521	0.8337544	0.36311	pygopus homolog 1 (Drosophila)
UP in Normoxia	**11177**	BAZ1A	-1.634716	0.8833647	0.3220339	bromodomain adjacent to zinc finger
UP in Normoxia	**735301**	SNHG9	-1.333492	0.8512138	0.3968066	small nucleolar RNA host gene 9 (non-protein coding)
UP in Normoxia	**6322**	SCML1	-1.306596	0.8834722	0.4042737	sex comb on midleg-like 1 (Drosophila)
UP in Normoxia	**55163**	PNPO	-1.430376	0.8919287	0.3710342	pyridoxamine 5'-phosphate oxidase
UP in Normoxia	**55135**	WRAP53	-1.603269	0.9079638	0.3291304	WD repeat containing, antisense to TP53
UP in Normoxia	**9486**	CHST10	-1.28384	0.8685568	0.4107008	carbohydrate sulfotransferase 10
UP in Normoxia	**83879**	CDCA7	-3.654248	0.9304219	0.0794258	cell division cycle associated 7
UP in Normoxia	**51388**	NIP7	-1.720508	0.9127385	0.3034419	NIP7, nucleolar pre-rRNA processing
UP in Normoxia	**60493**	FASTKD	-1.167757	0.817997	0.4451128	FAST kinase domains 5
UP in Normoxia	**6741**	SSB	-1.13005	0.8845203	0.4568999	Sjogren syndrome antigen B (autoantigen
UP in Normoxia	**80179**	MYO19	-1.393141	0.8934516	0.3807351	myosin XIX
UP in Normoxia	**375444**	C5orf34	-2.256802	0.897635	0.2092352	chromosome 5 open reading frame 34
UP in Normoxia	**116461**	TSEN15	-1.352925	0.8787781	0.3914975	tRNA splicing endonuclease 15 homolog (S. cerevisiae)
UP in Normoxia	**54925**	ZSCAN3	-1.206264	0.8098361	0.4333895	zinc finger and SCAN domain containing 32
UP in Normoxia	**1212**	CLTB	-1.786516	0.9195467	0.2898712	clathrin, light chain B
UP in Normoxia	**2739**	GLO1	-1.102259	0.8832572	0.4657867	glyoxalase I
UP in Normoxia	**51637**	C14orf16	-1.004292	0.8785273	0.4985148	chromosome 14 open reading frame 166
UP in Normoxia	**837**	CASP4	-1.09863	0.8758398	0.4669596	caspase 4, apoptosis-related cysteine
UP in Normoxia	**9021**	SOCS3	-1.334882	0.8946161	0.3964246	suppressor of cytokine signaling 3
UP in Normoxia	**55157**	DARS2	-1.779651	0.8981905	0.2912538	aspartyl-tRNA synthetase 2, mitochondrial
UP in Normoxia	**84306**	PDCD2L	-1.493454	0.8844665	0.3551613	programmed cell death 2-like
UP in Normoxia	**8420**	SNHG3	-1.689573	0.9162412	0.3100187	small nucleolar RNA host gene 3 (non-protein coding)
UP in Normoxia	**56938**	ARNTL2	-1.69455	0.9088507	0.3089509	aryl hydrocarbon receptor nuclear translocator-like 2
UP in Normoxia	**347240**	KIF24	-2.127875	0.8596793	0.2287946	kinesin family member 24
UP in Normoxia	**9542**	NRG2	-2.972779	0.8903162	0.127381	neuregulin 2
UP in Normoxia	**9972**	NUP153	-1.02728	0.855899	0.4906342	nucleoporin 153kDa
UP in Normoxia	**29843**	SENP1	-1.345964	0.8716922	0.393391	SUMO1/sentrin specific peptidase 1
UP in Normoxia	**31**	ACACA	-1.066963	0.8547613	0.4773226	acetyl-CoA carboxylase alpha
UP in Normoxia	**3336**	HSPE1	-1.856179	0.92356	0.2762069	heat shock 10kDa protein 1 (chaperonin 10)
UP in Normoxia	**10112**	KIF20A	-1.504265	0.8936487	0.3525097	kinesin family member 20A
UP in Normoxia	**5702**	PSMC3	-1.212601	0.8906656	0.4314899	proteasome (prosome, macropain) 26S subunit, ATPase, 3
UP in Normoxia	**93081**	TEX30	-1.590069	0.9097017	0.3321555	testis expressed 30
UP in Normoxia	**83540**	NUF2	-1.541422	0.893801	0.3435467	NUF2, NDC80 kinetochore complex component, homolog (S. cerevisiae)
UP in Normoxia	**10329**	TMEM5	-1.10122	0.864239	0.4661222	transmembrane protein 5
UP in Normoxia	**595**	CCND1	-1.66814	0.9194482	0.3146588	cyclin D1
UP in Normoxia	**55347**	ABHD10	-1.263862	0.8781779	0.4164276	abhydrolase domain containing 10

UP in Normoxia	**54982**	CLN6	-2.142717	0.9327421	0.2264529	ceroid-lipofuscinosis, neuronal 6, late infantile, variant
UP in Normoxia	**56902**	PNO1	-1.825703	0.9186061	0.2821037	partner of NOB1 homolog (S. cerevisiae)
UP in Normoxia	**254552**	NUDT8	-1.020033	0.8378662	0.4931051	nudix (nucleoside diphosphate linked moiety X)-type motif 8
UP in Normoxia	**57650**	KIAA152	-2.280684	0.9316671	0.2058002	KIAA1524
UP in Normoxia	**23310**	NCAPD3	-2.602665	0.9459733	0.1646341	non-SMC condensin II complex, subunit D3
UP in Normoxia	**57678**	GPAM	-3.249161	0.9667204	0.1051732	glycerol-3-phosphate acyltransferase, mitochondrial
UP in Normoxia	**2115**	ETV1	-2.350701	0.9429186	0.1960508	ets variant 1
UP in Normoxia	**7112**	TMPO	-2.174874	0.9328406	0.2214612	thymopoietin
UP in Normoxia	**284618**	RUSC1-AS1	-1.387543	0.833405	0.3822153	RUSC1 antisense RNA 1
UP in Normoxia	**55038**	CDCA4	-1.791796	0.9110275	0.2888122	cell division cycle associated 4
UP in Normoxia	**650**	BMP2	-1.153258	0.8511153	0.4496088	bone morphogenetic protein 2
UP in Normoxia	**91442**	C19orf40	-1.801817	0.9036191	0.2868132	chromosome 19 open reading frame 40
UP in Normoxia	**5226**	PGD	-1.456818	0.9009585	0.3642959	phosphogluconate dehydrogenase
UP in Normoxia	**10162**	LPCAT3	-1.707438	0.910714	0.3062033	lysophosphatidylcholine acyltransferase 3
UP in Normoxia	**116832**	RPL39L	-1.564321	0.8858909	0.3381367	ribosomal protein L39-like
UP in Normoxia	**55619**	DOCK10	-2.078144	0.9246618	0.2368189	dedicator of cytokinesis 10
UP in Normoxia	**118424**	UBE2J2	-1.637362	0.9166801	0.3214438	ubiquitin-conjugating enzyme E2, J2
UP in Normoxia	**9933**	KIAA002	-1.524651	0.8970796	0.3475635	KIAA0020
UP in Normoxia	**23274**	CLEC16	-1.098208	0.8601451	0.4670963	C-type lectin domain family 16, member A
UP in Normoxia	**79598**	CEP97	-1.395519	0.8350085	0.3801079	centrosomal protein 97kDa
UP in Normoxia	**64785**	GINS3	-1.978974	0.9095763	0.2536702	GINS complex subunit 3 (Psf3 homolog)
UP in Normoxia	**84286**	TMEM17	-1.411758	0.8773717	0.3758534	transmembrane protein 175
UP in Normoxia	**9**	NAT1	-1.74742	0.8545194	0.2978339	N-acetyltransferase 1 (arylamine N-acetyltransferase)
UP in Normoxia	**64225**	ATL2	-1.135535	0.8735824	0.4551661	atlastin GTPase 2
UP in Normoxia	**27242**	TNFRSF 21	-4.186827	0.9730359	0.0549085	tumor necrosis factor receptor superfamily, member 21
UP in Normoxia	**23228**	PLCL2	-2.476285	0.868727	0.1797066	phospholipase C-like 2
UP in Normoxia	**23067**	SETD1B	-1.618985	0.8657888	0.3255645	SET domain containing 1B
UP in Normoxia	**29985**	SLC39A3	-1.382995	0.8920631	0.3834219	solute carrier family 39 (zinc transporter), member 3
UP in Normoxia	**1737**	DLAT	-1.374202	0.8671414	0.3857661	dihydrolipoamide S-acetyltransferase
UP in Normoxia	**79847**	TMEM18	-1.114742	0.809639	0.4617737	transmembrane protein 180
UP in Normoxia	**9493**	KIF23	-2.12863	0.9346323	0.2286749	kinesin family member 23
UP in Normoxia	**10643**	IGF2BP3	-1.134935	0.8816806	0.4553553	insulin-like growth factor 2 mRNA binding protein 3
UP in Normoxia	**5631**	PRPS1	-2.13826	0.9360745	0.2271536	phosphoribosyl pyrophosphate synthetase 1
UP in Normoxia	**29078**	NDUFAF 4	-2.037421	0.920353	0.2435989	NADH dehydrogenase (ubiquinone) complex I, assembly factor 4
UP in Normoxia	**6949**	TCOF1	-1.182073	0.8843411	0.4407179	Treacher Collins-Franceschetti syndrome 1
UP in Normoxia	**653519**	GPR89A	-1.169925	0.8749888	0.4444444	G protein-coupled receptor 89A
UP in Normoxia	**11153**	FICD	-1.494838	0.8635313	0.3548206	FIC domain containing
UP in Normoxia	**5698**	PSMB9	-2.510816	0.9407597	0.1754564	proteasome (prosome, macropain) subunit, beta type, 9
UP in Normoxia	**3014**	H2AFX	-2.67319	0.9507032	0.1567797	H2A histone family, member X
UP in Normoxia	**641**	BLM	-2.754888	0.8958882	0.1481481	Bloom syndrome, RecQ helicase-like
UP in Normoxia	**10598**	AHSA1	-1.446306	0.9001612	0.3669597	AHA1, activator of heat shock 90kDa protein ATPase homolog 1 (yeast)
UP in Normoxia	**10194**	TSHZ1	-1.372192	0.8212667	0.3863038	teashirt zinc finger homeobox 1
UP in Normoxia	**55972**	SLC25A4	-1.156762	0.8589716	0.4485179	solute carrier family 25, member 40
UP in Normoxia	**84255**	SLC37A3	-1.048238	0.8555765	0.4835583	solute carrier family 37 (glycerol-3-phosphate transporter), member 3
UP in Normoxia	**79863**	RBFA	-1.119718	0.8102661	0.4601838	ribosome binding factor A (putative)

UP in Normoxia	**57020**	C16orf62	-1.172591	0.8779719	0.443624	chromosome 16 open reading frame 62
UP in Normoxia	**6059**	ABCE1	-2.059367	0.9294186	0.2399213	ATP-binding cassette, sub-family E (OABP), member 1
UP in Normoxia	**1105**	CHD1	-1.130548	0.8663979	0.4567422	chromodomain helicase DNA binding
UP in Normoxia	**57617**	VPS18	-1.028248	0.8694168	0.4903052	vacuolar protein sorting 18 homolog (S. cerevisiae)
UP in Normoxia	**10497**	UNC13B	-1.32786	0.8470483	0.3983586	unc-13 homolog B (C. elegans)
UP in Normoxia	**9711**	KIAA022	-1.110464	0.8533907	0.463145	KIAA0226
UP in Normoxia	**2805**	GOT1	-1.103851	0.8807758	0.465273	glutamic-oxaloacetic transaminase 1, soluble (aspartate aminotransferase 1)
UP in Normoxia	**5723**	PSPH	-1.301447	0.8832572	0.4057192	phosphoserine phosphatase
UP in Normoxia	**80155**	NAA15	-2.144753	0.9264445	0.2261335	N(alpha)-acetyltransferase 15, NatA auxiliary subunit
UP in Normoxia	**578**	BAK1	-1.307248	0.8627788	0.4040909	BCL2-antagonist/killer 1
UP in Normoxia	**55644**	OSGEP	-1.526873	0.8476126	0.3470286	O-sialoglycoprotein endopeptidase
UP in Normoxia	**9144**	SYNGR2	-1.40349	0.8876736	0.3780137	synaptogyrin 2
UP in Normoxia	**29929**	ALG6	-1.77961	0.8251724	0.2912621	ALG6, alpha-1,3-glucosyltransferase
UP in Normoxia	**9585**	KIF20B	-1.948278	0.9098809	0.2591253	kinesin family member 20B
UP in Normoxia	**113675**	SDSL	-1.884272	0.9145749	0.2708804	serine dehydratase-like
UP in Normoxia	**100505854**	RSBN1L-AS1	-1.42864	0.8546359	0.371481	RSBN1L antisense RNA 1
UP in Normoxia	**8200**	GDF5	-3.543805	0.9183284	0.0857449	growth differentiation factor 5
UP in Normoxia	**10539**	GLRX3	-1.074208	0.8832034	0.4749316	glutaredoxin 3
UP in Normoxia	**3925**	STMN1	-1.704777	0.9202455	0.3067686	stathmin 1
UP in Normoxia	**85021**	REPS1	-1.016996	0.8496909	0.4941443	RALBP1 associated Eps domain containing
UP in Normoxia	**51399**	TRAPPC	-1.293153	0.8862671	0.4080584	trafficking protein particle complex 4
UP in Normoxia	**8669**	EIF3J	-1.62153	0.9135358	0.3249905	eukaryotic translation initiation factor 3, subunit J
UP in Normoxia	**5515**	PPP2CA	-1.0002	0.8785631	0.4999307	protein phosphatase 2, catalytic subunit, alpha isozyme
UP in Normoxia	**162681**	C18orf54	-1.387904	0.8404192	0.3821197	chromosome 18 open reading frame 54
UP in Normoxia	**10654**	PMVK	-1.284656	0.887575	0.4104687	phosphomevalonate kinase
UP in Normoxia	**58478**	ENOPH1	-1.265168	0.8788408	0.416051	enolase-phosphatase 1
UP in Normoxia	**220064**	ORAOV1	-1.159154	0.8739676	0.4477752	oral cancer overexpressed 1
UP in Normoxia	**28976**	ACAD9	-1.608888	0.9115112	0.327851	acyl-CoA dehydrogenase family, member 9
UP in Normoxia	**253635**	GPATCH 11	-1.283146	0.8826928	0.4108984	G patch domain containing 11
UP in Normoxia	**10929**	SRSF8	-1.115105	0.8090657	0.4616576	serine/arginine-rich splicing factor 8
UP in Normoxia	**5468**	PPARG	-2.348279	0.8932455	0.1963801	peroxisome proliferator-activated receptor gamma
UP in Normoxia	**9128**	PRPF4	-1.59135	0.9127296	0.3318607	PRP4 pre-mRNA processing factor 4 homolog (yeast)
UP in Normoxia	**5069**	PAPPA	-1.446556	0.8941055	0.3668962	pregnancy-associated plasma protein A, pappalysin 1
UP in Normoxia	**2356**	FPGS	-1.208151	0.8857565	0.4328229	folylpolyglutamate synthase
UP in Normoxia	**387104**	SOGA3	-1.515746	0.8782854	0.3497157	SOGA family member 3
UP in Normoxia	**10198**	MPHOSP H9	-1.730691	0.8850399	0.3013076	M-phase phosphoprotein 9
UP in Normoxia	**132299**	OCIAD2	-1.472785	0.9013437	0.360286	OCIA domain containing 2
UP in Normoxia	**51318**	MRPL35	-1.012517	0.8698289	0.4956807	mitochondrial ribosomal protein L35
UP in Normoxia	**79581**	SLC52A2	-1.328982	0.8946699	0.3980489	solute carrier family 52, riboflavin transporter, member 2
UP in Normoxia	**84134**	TOMM4 0L	-2.281723	0.925056	0.205652	translocase of outer mitochondrial membrane 40 homolog (yeast)-like
UP in Normoxia	**5538**	PPT1	-1.221285	0.8879781	0.4289006	palmitoyl-protein thioesterase 1
UP in Normoxia	**55756**	INTS9	-1.090663	0.8468781	0.4695455	integrator complex subunit 9
UP in Normoxia	**9518**	GDF15	-1.080082	0.8834722	0.4730021	growth differentiation factor 15

UP in Normoxia	**10061**	ABCF2	-1.420258	0.8988175	0.3736454	ATP-binding cassette, sub-family F (GCN20), member 2
UP in Normoxia	**81839**	VANGL1	-1.627537	0.8840634	0.3236403	VANGL planar cell polarity protein 1
UP in Normoxia	**50804**	MYEF2	-1.17988	0.8307623	0.4413883	myelin expression factor 2
UP in Normoxia	**5036**	PA2G4	-1.585566	0.9175222	0.3331938	proliferation-associated 2G4, 38kDa
UP in Normoxia	**1836**	SLC26A2	-2.334586	0.9324375	0.1982529	solute carrier family 26 (sulfate transporter), member 2
UP in Normoxia	**54470**	ARMCX	-1.023186	0.8682523	0.4920287	armadillo repeat containing, X-linked 6
UP in Normoxia	**84250**	ANKRD3	-2.209719	0.8953865	0.2161765	ankyrin repeat domain 32
UP in Normoxia	**55008**	HERC6	-2.377563	0.8289886	0.1924342	HECT and RLD domain containing E3 ubiquitin protein ligase family member 6
UP in Normoxia	**7633**	ZNF79	-1.060121	0.8085371	0.4795918	zinc finger protein 79
UP in Normoxia	**1479**	CSTF3	-1.178011	0.8842336	0.4419603	cleavage stimulation factor, 3' pre-RNA, subunit 3, 77kDa
UP in Normoxia	**54478**	FAM64A	-1.88486	0.9129535	0.27077	family with sequence similarity 64, member
UP in Normoxia	**55748**	CNDP2	-1.168554	0.8831139	0.4448669	CNDP dipeptidase 2 (metallopeptidase M20 family)
UP in Normoxia	**440926**	H3F3AP4	-1.005394	0.87945	0.498134	H3 histone, family 3A, pseudogene 4
UP in Normoxia	**9787**	DLGAP5	-1.821249	0.9166353	0.282976	discs, large (Drosophila) homolog-associated protein 5
UP in Normoxia	**51367**	POP5	-1.409262	0.8958434	0.3765042	processing of precursor 5, ribonuclease P/MRP subunit (S. cerevisiae)
UP in Normoxia	**220323**	OAF	-1.136927	0.8771746	0.4547273	OAF homolog (Drosophila)
UP in Normoxia	**4281**	MID1	-1.97936	0.9108752	0.2536023	midline 1 (Opitz/BBB syndrome)
UP in Normoxia	**91408**	BTF3L4	-1.179295	0.8658963	0.4415673	basic transcription factor 3-like 4
UP in Normoxia	**79836**	LONRF3	-1.777608	0.8625997	0.2916667	LON peptidase N-terminal domain and ring finger 3
UP in Normoxia	**6840**	SVIL	-1.476854	0.9003315	0.3592715	supervillin
UP in Normoxia	**57568**	SIPA1L2	-2.138303	0.8351339	0.2271468	signal-induced proliferation-associated 1
UP in Normoxia	**51719**	CAB39	-1.19662	0.8853355	0.4362964	calcium binding protein 39
UP in Normoxia	**26272**	FBXO4	-1.66182	0.8863925	0.3160402	F-box protein 4
UP in Normoxia	**127253**	TYW3	-1.08145	0.8687808	0.4725537	tRNA-yW synthesizing protein 3 homolog (S. cerevisiae)
UP in Normoxia	**6432**	SRSF7	-1.656304	0.916268	0.3172509	serine/arginine-rich splicing factor 7
UP in Normoxia	**84876**	ORAI1	-2.135042	0.9328406	0.2276609	ORAI calcium release-activated calcium modulator 1
UP in Normoxia	**3981**	LIG4	-1.373841	0.8790379	0.3858625	ligase IV, DNA, ATP-dependent
UP in Normoxia	**643314**	KIAA075	-2.087837	0.9268028	0.2352332	KIAA0754
UP in Normoxia	**26128**	KIAA127	-1.305745	0.8894114	0.4045122	KIAA1279
UP in Normoxia	**121642**	ALKBH2	-1.363204	0.8838305	0.3887179	alkB, alkylation repair homolog 2 (E. coli)
UP in Normoxia	**84108**	PCGF6	-1.006022	0.848813	0.4979175	polycomb group ring finger 6
UP in Normoxia	**11113**	CIT	-1.59815	0.91006	0.3303003	citron (rho-interacting, serine/threonine kinase 21)
UP in Normoxia	**152503**	SH3D19	-2.106199	0.9117531	0.2322581	SH3 domain containing 19
UP in Normoxia	**90550**	MCU	-1.011783	0.861874	0.4959328	mitochondrial calcium uniporter
UP in Normoxia	**7150**	TOP1	-1.279417	0.8912389	0.4119621	topoisomerase (DNA) I
UP in Normoxia	**8890**	EIF2B4	-1.071206	0.877094	0.475921	eukaryotic translation initiation factor 2B, subunit 4 delta, 67kDa
UP in Normoxia	**38**	ACAT1	-1.148827	0.8768252	0.4509917	acetyl-CoA acetyltransferase 1
UP in Normoxia	**196**	AHR	-1.041548	0.8682254	0.4858058	aryl hydrocarbon receptor
UP in Normoxia	**30968**	STOML2	-1.026626	0.8810087	0.4908567	stomatin (EPB72)-like 2
UP in Normoxia	**79750**	ZNF385	-1.70663	0.8922154	0.3063749	zinc finger protein 385D
UP in Normoxia	**25896**	INTS7	-1.923482	0.9032966	0.2636175	integrator complex subunit 7
UP in Normoxia	**2967**	GTF2H3	-1.106101	0.8733315	0.4645477	general transcription factor IIH, polypeptide 3, 34kDa
UP in Normoxia	**4860**	PNP	-1.777968	0.9139568	0.2915939	purine nucleoside phosphorylase

UP in Normoxia	**1678**	TIMM8A	-1.513737	0.8888919	0.350203	translocase of inner mitochondrial membrane 8 homolog A (yeast)
UP in Normoxia	**26168**	SENP3	-1.165274	0.8849145	0.4458795	SUMO1/sentrin/SMT3 specific peptidase 3
UP in Normoxia	**11226**	GALNT6	-1.780964	0.9102929	0.2909889	UDP-N-acetyl-alpha-D-alactosamine: polypeptide N-acetylgalactosaminyl-transferase 6 (GalNAc-T6)
UP in Normoxia	**244**	ANXA8L	-1.008634	0.8578966	0.4970167	annexin A8-like 2
UP in Normoxia	**152742**	LOC152742	-4.704134	0.8293828	0.0383632	uncharacterized LOC152742
UP in Normoxia	**84967**	LSM10	-1.439767	0.8970348	0.3686267	LSM10, U7 small nuclear RNA associated
UP in Normoxia	**54331**	GNG2	-2.059454	0.888435	0.2399068	guanine nucleotide binding protein (G protein), gamma 2
UP in Normoxia	**348654**	GEN1	-2.021332	0.8992206	0.2463306	Gen endonuclease homolog 1 (Drosophila)
UP in Normoxia	**51512**	GTSE1	-1.476572	0.8925826	0.3593417	G-2 and S-phase expressed 1
UP in Normoxia	**64118**	DUS1L	-1.332087	0.8923856	0.3971931	dihydrouridine synthase 1-like (S.
UP in Normoxia	**10212**	DDX39A	-2.079238	0.9370241	0.2366394	DEAD (Asp-Glu-Ala-Asp) box polypeptide
UP in Normoxia	**114034**	TOE1	-1.968345	0.9037445	0.2555461	target of EGR1, member 1 (nuclear)
UP in Normoxia	**92822**	ZNF276	-1.241643	0.8497895	0.4228909	zinc finger protein 276
UP in Normoxia	**6838**	SURF6	-1.107707	0.8733405	0.464031	surfeit 6
UP in Normoxia	**56990**	CDC42S	-1.080353	0.8585147	0.4729131	CDC42 small effector 2
UP in Normoxia	**5111**	PCNA	-2.645745	0.9563558	0.1597906	proliferating cell nuclear antigen
UP in Normoxia	**55969**	C20orf24	-1.247054	0.8931022	0.4213076	chromosome 20 open reading frame 24
UP in Normoxia	**83660**	TLN2	-1.222392	0.8127206	0.4285714	talin 2
UP in Normoxia	**9874**	TLK1	-1.011739	0.8611664	0.4959481	tousled-like kinase 1
UP in Normoxia	**983**	CDK1	-1.703519	0.9184807	0.3070362	cyclin-dependent kinase 1
UP in Normoxia	**57171**	DOLPP1	-2.096368	0.9159635	0.2338462	dolichyl pyrophosphate phosphatase 1
UP in Normoxia	**338382**	RAB7B	-2.17039	0.9021768	0.2221507	RAB7B, member RAS oncogene family
UP in Normoxia	**3145**	HMBS	-2.48558	0.9438681	0.1785525	hydroxymethylbilane synthase
UP in Normoxia	**2305**	FOXM1	-1.548723	0.9003673	0.3418124	forkhead box M1
UP in Normoxia	**79066**	METTL1	-1.028234	0.8563917	0.4903101	methyltransferase like 16
UP in Normoxia	**26468**	LHX6	-1.620323	0.8907731	0.3252627	LIM homeobox 6
UP in Normoxia	**134359**	POC5	-1.736168	0.8986832	0.3001658	POC5 centriolar protein homolog (Chlamydomonas)
UP in Normoxia	**63979**	FIGNL1	-1.481226	0.8830422	0.3581842	fidgetin-like 1
UP in Normoxia	**2633**	GBP1	-1.608575	0.8285945	0.3279221	guanylate binding protein 1, interferon-
UP in Normoxia	**677838**	SNORA6	-7.212699	0.8623757	0.0067416	small nucleolar RNA, H/ACA box 61
UP in Normoxia	**5570**	PKIB	-2.228532	0.9038072	0.2133758	protein kinase (cAMP-dependent, catalytic) inhibitor beta
UP in Normoxia	**390940**	PINLYP	-1.884749	0.9165009	0.2707909	phospholipase A2 inhibitor and LY6/PLAUR domain containing
UP in Normoxia	**27085**	MTBP	-2.247928	0.9031264	0.2105263	Mdm2, transformed 3T3 cell double minute 2, p53 binding protein (mouse) binding protein, 104kDa
UP in Normoxia	**3161**	HMMR	-1.097356	0.8774075	0.4673721	hyaluronan-mediated motility receptor (RHAMM)
UP in Normoxia	**118980**	SFXN2	-3.542402	0.8470125	0.0858283	sideroflexin 2
UP in Normoxia	**56906**	THAP10	-1.224966	0.806629	0.4278075	THAP domain containing 10
UP in Normoxia	**134285**	TMEM17	-2.296393	0.9219833	0.2035714	transmembrane protein 171
UP in Normoxia	**3833**	KIFC1	-1.73107	0.9073815	0.3012284	kinesin family member C1
UP in Normoxia	**79017**	GGCT	-2.946103	0.955675	0.1297581	gamma-glutamylcyclotransferase
UP in Normoxia	**84128**	WDR75	-1.536335	0.8956822	0.3447602	WD repeat domain 75
UP in Normoxia	**5691**	PSMB3	-1.253083	0.8929768	0.4195506	proteasome (prosome, macropain) subunit, beta type, 3
UP in Normoxia	**51540**	SCLY	-2.996705	0.9482756	0.1252858	selenocysteine lyase
UP in Normoxia	**414236**	C10orf55	-3.934446	0.9629401	0.0654054	chromosome 10 open reading frame 55
UP in Normoxia	**121274**	ZNF641	-1.948726	0.8108573	0.2590449	zinc finger protein 641
UP in Normoxia	**79768**	KATNBL	-1.180267	0.8731613	0.4412698	katanin p80 subunit B-like 1

UP in Normoxia	**9221**	NOLC1	-2.162818	0.9367016	0.2233197	nucleolar and coiled-body phosphoprotein 1
UP in Normoxia	**79077**	DCTPP1	-2.451996	0.9469677	0.1827576	dCTP pyrophosphatase 1
UP in Normoxia	**148741**	ANKRD3	-2.994353	0.9371316	0.1254902	ankyrin repeat domain 35
UP in Normoxia	**11004**	KIF2C	-1.606969	0.9133118	0.3282873	kinesin family member 2C
UP in Normoxia	**5664**	PSEN2	-2.157477	0.9261578	0.2241479	presenilin 2 (Alzheimer disease 4)
UP in Normoxia	**85026**	C9orf37	-1.048087	0.8605572	0.483609	chromosome 9 open reading frame 37
UP in Normoxia	**10036**	CHAF1A	-2.16453	0.9308967	0.2230548	chromatin assembly factor 1, subunit A
UP in Normoxia	**27327**	TNRC6A	-1.068519	0.8715399	0.4768082	trinucleotide repeat containing 6A
UP in Normoxia	**57095**	PITHD1	-1.386176	0.8942309	0.3825774	PITH (C-terminal proteasome-interacting domain of thioredoxin-like) domain containing 1
UP in Normoxia	**85019**	TMEM24	-1.463322	0.8579593	0.3626571	transmembrane protein 241
UP in Normoxia	**54806**	AHI1	-1.062566	0.8173609	0.4787798	Abelson helper integration site 1
UP in Normoxia	**84515**	MCM8	-2.546956	0.9363343	0.1711157	minichromosome maintenance complex component 8
UP in Normoxia	**6728**	SRP19	-1.493138	0.8960315	0.355239	signal recognition particle 19kDa
UP in Normoxia	**3717**	JAK2	-1.012862	0.80129	0.4955621	Janus kinase 2
UP in Normoxia	**29088**	MRPL15	-1.816232	0.9192511	0.2839617	mitochondrial ribosomal protein L15
UP in Normoxia	**7296**	TXNRD1	-2.85371	0.9627967	0.13834	thioredoxin reductase 1
UP in Normoxia	**259266**	ASPM	-1.639627	0.8887485	0.3209393	asp (abnormal spindle) homolog, microcephaly associated (Drosophila)
UP in Normoxia	**59353**	TMEM35	-3.636037	0.8361552	0.0804348	transmembrane protein 35
UP in Normoxia	**57505**	AARS2	-1.343815	0.8690047	0.3939774	alanyl-tRNA synthetase 2, mitochondrial
UP in Normoxia	**6043**	SNORA6	-7.129283	0.8530771	0.0071429	small nucleolar RNA, H/ACA box 63
UP in Normoxia	**57567**	ZNF319	-1.631656	0.8686016	0.3227176	zinc finger protein 319
UP in Normoxia	**51715**	RAB23	-1.579688	0.9112694	0.3345543	RAB23, member RAS oncogene family
UP in Normoxia	**440275**	EIF2AK4	-1.168458	0.8802383	0.4448966	eukaryotic translation initiation factor 2 alpha kinase 4
UP in Normoxia	**64087**	MCCC2	-1.631133	0.9064857	0.3228346	methylcrotonoyl-CoA carboxylase 2 (beta)
UP in Normoxia	**79171**	RBM42	-1.026153	0.8796918	0.4910177	RNA binding motif protein 42
UP in Normoxia	**245972**	ATP6V0 D2	-1.477104	0.880086	0.3592091	ATPase, H+ transporting, lysosomal 38kDa, V0 subunit d2
UP in Normoxia	**57698**	KIAA159	-1.683467	0.893147	0.3113336	KIAA1598
UP in Normoxia	**51121**	RPL26L1	-1.005751	0.8791096	0.4980109	ribosomal protein L26-like 1
UP in Normoxia	**8318**	CDC45	-3.166505	0.9661113	0.1113748	cell division cycle 45
UP in Normoxia	**64793**	CEP85	-1.33125	0.8628684	0.3974237	centrosomal protein 85kDa
UP in Normoxia	**10531**	PITRM1	-1.189378	0.8878706	0.4384918	pitrilysin metallopeptidase 1
UP in Normoxia	**84529**	C15orf41	-1.000566	0.8465825	0.4998038	chromosome 15 open reading frame 41
UP in Normoxia	**124245**	ZC3H18	-1.041791	0.8715578	0.4857242	zinc finger CCCH-type containing 18
UP in Normoxia	**6573**	SLC19A1	-1.021915	0.817549	0.4924623	solute carrier family 19 (folate transporter), member 1
UP in Normoxia	**199857**	ALG14	-1.075849	0.822279	0.4743918	ALG14, UDP-N-acetylglucosaminyltransferase subunit
UP in Normoxia	**79081**	C11orf48	-1.624321	0.9151662	0.3243625	chromosome 11 open reading frame 48
UP in Normoxia	**5162**	PDHB	-1.051235	0.8796202	0.4825549	pyruvate dehydrogenase (lipoamide) beta
UP in Normoxia	**860**	RUNX2	-1.179409	0.8286034	0.4415323	runt-related transcription factor 2
UP in Normoxia	**545**	ATR	-2.072617	0.9213294	0.2377279	ataxia telangiectasia and Rad3 related
UP in Normoxia	**51633**	OTUD6B	-1.581961	0.8930126	0.3340275	OTU domain containing 6B
UP in Normoxia	**23035**	PHLPP2	-1.013632	0.8167786	0.4952978	PH domain and leucine rich repeat protein phosphatase 2
UP in Normoxia	**3397**	ID1	-1.020735	0.8811699	0.4928652	inhibitor of DNA binding 1, dominant negative helix-loop-helix protein
UP in Normoxia	**10579**	TACC2	-2.199644	0.9039237	0.2176913	transforming, acidic coiled-coil containing protein 2
UP in Normoxia	**2820**	GPD2	-1.332644	0.8828899	0.3970401	glycerol-3-phosphate dehydrogenase 2 (mitochondrial)

UP in Normoxia	**27235**	COQ2	-1.722196	0.9051778	0.303087	coenzyme Q2 homolog, prenyltransferase (yeast)
UP in Normoxia	**199953**	TMEM20	-2.46362	0.9332348	0.1812911	transmembrane protein 201
UP in Normoxia	**3082**	HGF	-1.818315	0.8939174	0.283552	hepatocyte growth factor (hepapoietin A; scatter factor)
UP in Normoxia	**4595**	MUTYH	-1.127728	0.8434023	0.4576357	mutY homolog (E. coli)
UP in Normoxia	**9903**	KLHL21	-1.01199	0.8772373	0.4958618	kelch-like family member 21
UP in Normoxia	**132720**	C4orf32	-1.358067	0.8123175	0.3901047	chromosome 4 open reading frame 32
UP in Normoxia	**51478**	HSD17B	-1.287518	0.835752	0.4096552	hydroxysteroid (17-beta) dehydrogenase 7
UP in Normoxia	**130574**	LYPD6	-2.488607	0.8876736	0.1781782	LY6/PLAUR domain containing 6
UP in Normoxia	**9188**	DDX21	-1.896717	0.9218579	0.2685537	DEAD (Asp-Glu-Ala-Asp) box helicase 21
UP in Normoxia	**374659**	HDDC3	-1.006076	0.8632446	0.4978986	HD domain containing 3
UP in Normoxia	**11128**	POLR3A	-1.091657	0.8705724	0.4692221	polymerase (RNA) III (DNA directed) polypeptide A, 155kDa
UP in Normoxia	**79954**	NOL10	-1.150376	0.8734659	0.4505079	nucleolar protein 10
UP in Normoxia	**55151**	TMEM38 B	-1.802054	0.9142972	0.2867661	transmembrane protein 38B
UP in Normoxia	**3790**	KCNS3	-1.019856	0.8614978	0.4931657	potassium voltage-gated channel, delayed-rectifier, subfamily S, member 3
UP in Normoxia	**2827**	GPR3	-2.118611	0.8868763	0.2302685	G protein-coupled receptor 3
UP in Normoxia	**79663**	HSPBAP	-1.819607	0.876879	0.2832981	HSPB (heat shock 27kDa) associated
UP in Normoxia	**92126**	DSEL	-1.628301	0.8949028	0.323469	dermatan sulfate epimerase-like
UP in Normoxia	**2130**	EWSR1	-1.382203	0.8977605	0.3836325	Ewing sarcoma breakpoint region 1
UP in Normoxia	**90809**	TMEM55 B	-1.342907	0.8815641	0.3942254	transmembrane protein 55B
UP in Normoxia	**84262**	PSMG3	-1.161258	0.8811431	0.4471225	proteasome (prosome, macropain) assembly chaperone 3
UP in Normoxia	**23354**	HAUS5	-1.502045	0.8719072	0.3530525	HAUS augmin-like complex, subunit 5
UP in Normoxia	**284129**	SLC26A1	-1.031208	0.8254501	0.4893002	solute carrier family 26, member 11
UP in Normoxia	**157753**	TMEM74	-2.372456	0.805357	0.1931166	transmembrane protein 74
UP in Normoxia	**54868**	TMEM10	-2.014373	0.923336	0.2475218	transmembrane protein 104
UP in Normoxia	**222194**	RSBN1L	-1.215312	0.8496909	0.43068	round spermatid basic protein 1-like
UP in Normoxia	**2059**	EPS8	-2.132501	0.9152558	0.2280622	epidermal growth factor receptor pathway substrate 8
UP in Normoxia	**63939**	FAM217	-1.277074	0.8536057	0.4126316	family with sequence similarity 217,
UP in Normoxia	**119392**	SFR1	-1.18066	0.853955	0.4411496	SWI5-dependent recombination repair 1
UP in Normoxia	**80772**	GLTPD1	-1.041733	0.8738422	0.4857435	glycolipid transfer protein domain
UP in Normoxia	**284403**	WDR62	-2.740313	0.9429275	0.1496524	WD repeat domain 62
UP in Normoxia	**81624**	DIAPH3	-2.200359	0.9137866	0.2175835	diaphanous homolog 3 (Drosophila)
UP in Normoxia	**7748**	ZNF195	-1.237915	0.8009316	0.4239849	zinc finger protein 195
UP in Normoxia	**219743**	TYSND1	-1.514918	0.8772104	0.3499164	trypsin domain containing 1
UP in Normoxia	**23590**	PDSS1	-2.157884	0.9167966	0.2240847	prenyl (decaprenyl) diphosphate synthase, subunit 1
UP in Normoxia	**10262**	SF3B4	-1.181983	0.8882917	0.4407451	splicing factor 3b, subunit 4, 49kDa
UP in Normoxia	**222229**	LRWD1	-1.195004	0.8824241	0.4367852	leucine-rich repeats and WD repeat domain containing 1
UP in Normoxia	**4522**	MTHFD1	-2.847777	0.9611036	0.1389101	methylenetetrahydrofolate dehydrogenase (NADP+ dependent) 1, methenyl-tetrahydrofolate cyclohydrolase, formyltetrahydrofolate synthetase
UP in Normoxia	**11031**	RAB31	-1.767384	0.9214727	0.293741	RAB31, member RAS oncogene family
UP in Normoxia	**79594**	MUL1	-1.024341	0.8703126	0.4916347	mitochondrial E3 ubiquitin protein ligase 1
UP in Normoxia	**54976**	C20orf27	-1.389109	0.8923587	0.3818005	chromosome 20 open reading frame 27
UP in Normoxia	**8651**	SOCS1	-2.153831	0.922279	0.2247151	suppressor of cytokine signaling 1
UP in Normoxia	**22854**	NTNG1	-2.2481	0.9157395	0.2105012	netrin G1
UP in Normoxia	**645638**	LOC6456 38	-1.499368	0.877506	0.3537082	WDNM1-like pseudogene

UP in Normoxia	**53840**	TRIM34	-1.421599	0.8631372	0.3732984	tripartite motif containing 34
UP in Normoxia	**84318**	CCDC77	-1.458122	0.8798531	0.3639666	coiled-coil domain containing 77
UP in Normoxia	**83746**	L3MBTL	-1.718563	0.9041118	0.3038512	l(3)mbt-like 2 (Drosophila)
UP in Normoxia	**140710**	SOGA1	-1.587568	0.9121114	0.3327318	suppressor of glucose, autophagy associated
UP in Normoxia	**55166**	CENPQ	-1.655448	0.9064409	0.3174391	centromere protein Q
UP in Normoxia	**23052**	ENDOD1	-1.024693	0.8575204	0.491515	endonuclease domain containing 1
UP in Normoxia	**4693**	NDP	-1.025662	0.8531488	0.4911848	Norrie disease (pseudoglioma)
UP in Normoxia	**55661**	DDX27	-1.154296	0.8851474	0.4492854	DEAD (Asp-Glu-Ala-Asp) box polypeptide
UP in Normoxia	**22979**	EFR3B	-2.903818	0.9265162	0.1336176	EFR3 homolog B (S. cerevisiae)
UP in Normoxia	**79713**	IGFLR1	-2.662329	0.9233808	0.1579643	IGF-like family receptor 1
UP in Normoxia	**23246**	BOP1	-2.274682	0.9359939	0.2066581	block of proliferation 1
UP in Normoxia	**1861**	TOR1A	-1.274968	0.8890621	0.4132344	torsin family 1, member A (torsin A)
UP in Normoxia	**2825**	GPR1	-1.991283	0.8060557	0.2515152	G protein-coupled receptor 1
UP in Normoxia	**5719**	PSMD13	-1.037389	0.8812774	0.4872086	proteasome (prosome, macropain) 26S subunit, non-ATPase, 13
UP in Normoxia	**100303755**	PET117	-1.094376	0.8256741	0.4683386	PET117 homolog (S. cerevisiae)
UP in Normoxia	**3268**	AGFG2	-1.978959	0.8567231	0.2536729	ArfGAP with FG repeats 2
UP in Normoxia	**81608**	FIP1L1	-1.101873	0.875159	0.4659111	FIP1 like 1 (S. cerevisiae)
UP in Normoxia	**84946**	LTV1	-2.008151	0.9328138	0.2485915	LTV1 homolog (S. cerevisiae)
UP in Normoxia	**22994**	AZI1	-1.187103	0.8619815	0.439184	5-azacytidine induced 1
UP in Normoxia	**6790**	AURKA	-2.024584	0.9357789	0.245776	aurora kinase A
UP in Normoxia	**54820**	NDE1	-1.140692	0.8504345	0.4535419	nudE nuclear distribution E homolog 1 (A. nidulans)
UP in Normoxia	**4172**	MCM3	-3.445299	0.9733853	0.091804	minichromosome maintenance complex component 3
UP in Normoxia	**151636**	DTX3L	-1.899619	0.9007973	0.2680141	deltex 3-like (Drosophila)
UP in Normoxia	**4670**	HNRNP	-1.347627	0.8972051	0.3929379	heterogeneous nuclear ribonucleoprotein M
UP in Normoxia	**220134**	SKA1	-1.542627	0.8957359	0.3432599	spindle and kinetochore associated complex subunit 1
UP in Normoxia	**84934**	C12orf52	-1.116455	0.8772911	0.4612258	chromosome 12 open reading frame 52
UP in Normoxia	**11340**	EXOSC8	-1.397122	0.8935949	0.3796858	exosome component 8
UP in Normoxia	**23517**	SKIV2L2	-1.006299	0.873009	0.4978218	superkiller viralicidic activity 2-like 2 (S. cerevisiae)
UP in Normoxia	**7415**	VCP	-1.00174	0.8803458	0.4993974	valosin containing protein
UP in Normoxia	**6884**	TAF13	-1.378453	0.8970707	0.3846311	TAF13 RNA polymerase II, TATA box binding protein (TBP)-associated factor, 18kDa
UP in Normoxia	**51053**	GMNN	-1.686287	0.9074801	0.3107256	geminin, DNA replication inhibitor
UP in Normoxia	**79643**	CHMP6	-1.475123	0.8908	0.3597028	charged multivesicular body protein 6
UP in Normoxia	**122553**	TRAPPC 6B	-1.186247	0.843474	0.4394444	trafficking protein particle complex 6B
UP in Normoxia	**85476**	GFM1	-1.653734	0.9127027	0.3178165	G elongation factor, mitochondrial 1
UP in Normoxia	**10602**	CDC42E	-1.075939	0.8682523	0.4743621	CDC42 effector protein (Rho GTPase
UP in Normoxia	**3772**	KCNJ15	-2.760146	0.8093613	0.1476091	potassium inwardly-rectifying channel, subfamily J, member 15
UP in Normoxia	**54733**	SLC35F2	-1.183122	0.8787333	0.4403974	solute carrier family 35, member F2
UP in Normoxia	**55848**	PLGRKT	-1.086289	0.8791543	0.4709713	plasminogen receptor, C-terminal lysine transmembrane protein
UP in Normoxia	**699**	BUB1	-1.449227	0.8956195	0.3662175	BUB1 mitotic checkpoint serine/threonine
UP in Normoxia	**5291**	PIK3CB	-1.379576	0.8808743	0.3843318	phosphatidylinositol-4,5-bisphosphate 3-kinase, catalytic subunit beta
UP in Normoxia	**51728**	POLR3K	-2.515087	0.9420675	0.1749377	polymerase (RNA) III (DNA directed) polypeptide K, 12.3 kDa
UP in Normoxia	**133396**	IL31RA	-2.369528	0.9369076	0.1935089	interleukin 31 receptor A
UP in Normoxia	**9119**	KRT75	-1.436214	0.8858998	0.3695357	keratin 75
UP in Normoxia	**899**	CCNF	-1.841651	0.8882917	0.2790023	cyclin F
UP in Normoxia	**55052**	MRPL20	-1.576919	0.9174236	0.3351969	mitochondrial ribosomal protein L20

UP in Normoxia	**6341**	SCO1	-1.410803	0.8905491	0.3761022	SCO1 cytochrome c oxidase assembly
UP in Normoxia	**137682**	NDUFAF6	-1.218504	0.844325	0.4297282	NADH dehydrogenase (ubiquinone) complex I, assembly factor 6
UP in Normoxia	**1503**	CTPS1	-3.432417	0.9725611	0.0926274	CTP synthase 1
UP in Normoxia	**10280**	SIGMAR	-2.075655	0.9368091	0.2372278	sigma non-opioid intracellular receptor 1
UP in Normoxia	**100507463**	LOC100507463	-1.065275	0.8232375	0.4778816	uncharacterized LOC100507463
UP in Normoxia	**83990**	BRIP1	-1.240935	0.8478456	0.4230983	BRCA1 interacting protein C-terminal
UP in Normoxia	**5495**	PPM1B	-1.069829	0.8593299	0.4763754	protein phosphatase, Mg2+/Mn2+ dependent, 1B
UP in Normoxia	**348327**	ZNF530	-1.742915	0.8206844	0.2987654	zinc finger protein 530
UP in Normoxia	**5328**	PLAU	-4.86068	0.9941503	0.0344183	plasminogen activator, urokinase
UP in Normoxia	**26009**	ZZZ3	-1.015103	0.8605034	0.4947931	zinc finger, ZZ-type containing 3
UP in Normoxia	**92715**	WDR85	-1.526253	0.8833916	0.3471778	WD repeat domain 85
UP in Normoxia	**7884**	SLBP	-1.424522	0.8979127	0.3725427	stem-loop binding protein
UP in Normoxia	**10607**	TBL3	-1.545921	0.8898862	0.3424769	transducin (beta)-like 3
UP in Normoxia	**8604**	SLC25A12	-1.503855	0.889501	0.35261	solute carrier family 25 (aspartate/glutamate carrier), member 12
UP in Normoxia	**283643**	C14orf80	-1.660429	0.9043537	0.316345	chromosome 14 open reading frame 80
UP in Normoxia	**9055**	PRC1	-1.874882	0.9221446	0.2726492	protein regulator of cytokinesis 1
UP in Normoxia	**7058**	THBS2	-1.838591	0.8978769	0.2795947	thrombospondin 2
UP in Normoxia	**4354**	MPP1	-1.076827	0.8770133	0.4740702	membrane protein, palmitoylated 1, 55kDa
UP in Normoxia	**55787**	TXLNG	-1.235737	0.8570814	0.4246255	taxilin gamma
UP in Normoxia	**138428**	PTRH1	-1.897812	0.915408	0.2683501	peptidyl-tRNA hydrolase 1 homolog (S. cerevisiae)
UP in Normoxia	**86**	ACTL6A	-1.577825	0.9144495	0.3349866	actin-like 6A
UP in Normoxia	**10024**	TROAP	-2.119274	0.9320254	0.2301626	trophinin associated protein
UP in Normoxia	**55304**	SPTLC3	-2.90105	0.9290692	0.1338742	serine palmitoyltransferase, long chain base subunit 3
UP in Normoxia	**57405**	SPC25	-1.18846	0.8822987	0.4387709	SPC25, NDC80 kinetochore complex component, homolog (S. cerevisiae)
UP in Normoxia	**137392**	FAM92A	-1.170847	0.87816	0.4441604	family with sequence similarity 92, member
UP in Normoxia	**10436**	EMG1	-1.661489	0.9178088	0.3161128	EMG1 nucleolar protein homolog (S.
UP in Normoxia	**23198**	PSME4	-1.345378	0.8882558	0.3935508	proteasome (prosome, macropain) activator subunit 4
UP in Normoxia	**100125556**	FAM86JP	-2.324502	0.8155783	0.1996435	family with sequence similarity 86, member A pseudogene
UP in Normoxia	**51020**	HDDC2	-1.00667	0.8744871	0.4976938	HD domain containing 2
UP in Normoxia	**1163**	CKS1B	-1.094251	0.8839201	0.4683794	CDC28 protein kinase regulatory subunit 1B
UP in Normoxia	**55033**	FKBP14	-1.195793	0.863818	0.4365463	FK506 binding protein 14, 22 kDa
UP in Normoxia	**115701**	ALPK2	-1.092238	0.8084386	0.4690332	alpha-kinase 2
UP in Normoxia	**79763**	ISOC2	-1.071355	0.8789662	0.475872	isochorismatase domain containing 2
UP in Normoxia	**155400**	NSUN5P1	-1.069288	0.8339246	0.476554	NOP2/Sun domain family, member 5 pseudogene 1
UP in Normoxia	**7866**	IFRD2	-1.758068	0.9188211	0.2956439	interferon-related developmental regulator 2
UP in Normoxia	**65260**	SELRC1	-1.836647	0.8773538	0.2799718	Sel1 repeat containing 1
UP in Normoxia	**80142**	PTGES2	-1.293936	0.8894742	0.407837	prostaglandin E synthase 2
UP in Normoxia	**63967**	CLSPN	-3.174632	0.9404103	0.1107492	claspin
UP in Normoxia	**1660**	DHX9	-1.313084	0.8922781	0.4024597	DEAH (Asp-Glu-Ala-His) box polypeptide
UP in Normoxia	**3887**	KRT81	-1.850074	0.9030279	0.2773781	keratin 81
UP in Normoxia	**113115**	MTFR2	-2.175512	0.8976709	0.2213633	mitochondrial fission regulator 2
UP in Normoxia	**5685**	PSMA4	-1.346794	0.8973663	0.3931647	proteasome (prosome, macropain) subunit, alpha type, 4
UP in Normoxia	**284184**	C17orf89	-1.113855	0.8829974	0.4620579	chromosome 17 open reading frame 89
UP in Normoxia	**9897**	KIAA019	-1.005676	0.8742542	0.4980366	KIAA0196
UP in Normoxia	**81875**	ISG20L2	-1.568551	0.8928335	0.3371469	interferon stimulated exonuclease gene 20kDa-like 2

UP in Normoxia	**84798**	C19orf48	-2.052571	0.9346681	0.2410542	chromosome 19 open reading frame 48
UP in Normoxia	**6764**	ST5	-1.328031	0.8828182	0.3983116	suppression of tumorigenicity 5
UP in Normoxia	**89839**	ARHGA P11B	-1.165237	0.8099704	0.445891	Rho GTPase activating protein 11B
UP in Normoxia	**197021**	LCTL	-2.458488	0.8607722	0.1819372	lactase-like
UP in Normoxia	**3176**	HNMT	-1.000896	0.8167876	0.4996896	histamine N-methyltransferase
UP in Normoxia	**51605**	TRMT6	-1.349338	0.8877363	0.3924721	tRNA methyltransferase 6 homolog (S. cerevisiae)
UP in Normoxia	**9890**	LPPR4	-1.264641	0.8424886	0.4162028	lipid phosphate phosphatase-related protein type 4
UP in Normoxia	**140809**	SRXN1	-1.301033	0.8899937	0.4058355	sulfiredoxin 1
UP in Normoxia	**56998**	CTNNBI P1	-1.194137	0.8231837	0.4370478	catenin, beta interacting protein 1
UP in Normoxia	**83666**	PARP9	-3.06386	0.9337185	0.1195876	poly (ADP-ribose) polymerase family,
UP in Normoxia	**5359**	PLSCR1	-1.568694	0.8853624	0.3371134	phospholipid scramblase 1
UP in Normoxia	**54969**	C4orf27	-1.247278	0.8761892	0.4212424	chromosome 4 open reading frame 27
UP in Normoxia	**10635**	RAD51A P1	-2.26461	0.9292574	0.208106	RAD51 associated protein 1
UP in Normoxia	**8624**	PSMG1	-1.956908	0.9233181	0.2575799	proteasome (prosome, macropain) assembly chaperone 1
UP in Normoxia	**23266**	LPHN2	-1.474393	0.8195198	0.3598848	latrophilin 2
UP in Normoxia	**5646**	PRSS3	-1.186377	0.8825137	0.439405	protease, serine, 3
UP in Normoxia	**23513**	SCRIB	-1.228567	0.8827824	0.4267412	scribbled planar cell polarity protein
UP in Normoxia	**9650**	MTFR1	-1.183032	0.8798889	0.4404248	mitochondrial fission regulator 1
UP in Normoxia	**11339**	OIP5	-1.947376	0.8764579	0.2592873	Opa interacting protein 5
UP in Normoxia	**81928**	CABLES	-1.241199	0.8554242	0.4230209	Cdk5 and Abl enzyme substrate 2
UP in Normoxia	**2963**	GTF2F2	-1.700563	0.9183194	0.3076661	general transcription factor IIF, polypeptide 2, 30kDa
UP in Normoxia	**6446**	SGK1	-1.610505	0.9047926	0.3274838	serum/glucocorticoid regulated kinase 1
UP in Normoxia	**23595**	ORC3	-1.102185	0.8742184	0.4658104	origin recognition complex, subunit 3
UP in Normoxia	**2135**	EXTL2	-1.227551	0.8775956	0.4270418	exostosin-like glycosyltransferase 2
UP in Normoxia	**25926**	NOL11	-1.449299	0.8938457	0.3661993	nucleolar protein 11
UP in Normoxia	**57805**	KIAA196	-1.178148	0.8863119	0.4419184	KIAA1967
UP in Normoxia	**113000**	RPUSD1	-1.446749	0.8870465	0.3668471	RNA pseudouridylate synthase domain containing 1
UP in Normoxia	**865**	CBFB	-1.135996	0.8829705	0.4550207	core-binding factor, beta subunit
UP in Normoxia	**5557**	PRIM1	-2.438555	0.9408582	0.1844683	primase, DNA, polypeptide 1 (49kDa)
UP in Normoxia	**1116**	CHI3L1	-1.044333	0.8140464	0.4848689	chitinase 3-like 1 (cartilage glycoprotein-39)
UP in Normoxia	**10483**	SEC23B	-1.166401	0.8784825	0.4455313	Sec23 homolog B (S. cerevisiae)
UP in Normoxia	**152302**	CIDECP	-1.352119	0.8758846	0.3917164	cell death-inducing DFFA-like effector c pseudogene
UP in Normoxia	**84313**	VPS25	-1.179036	0.8872077	0.4416464	vacuolar protein sorting 25 homolog (S. cerevisiae)
UP in Normoxia	**129401**	NUP35	-2.184992	0.926767	0.2199134	nucleoporin 35kDa
UP in Normoxia	**58538**	MPP4	-1.300166	0.8262743	0.4060795	membrane protein, palmitoylated 4 (MAGUK p55 subfamily member 4)
UP in Normoxia	**2222**	FDFT1	-1.015637	0.8781152	0.4946099	farnesyl-diphosphate farnesyltransferase 1
UP in Normoxia	**56052**	ALG1	-1.236968	0.8692645	0.4242634	ALG1, chitobiosyldiphosphodolichol beta-mannosyltransferase
UP in Normoxia	**3151**	HMGN2	-1.310756	0.894652	0.4031096	high mobility group nucleosomal binding domain 2
UP in Normoxia	**9582**	APOBEC 3B	-1.954122	0.9218131	0.2580778	apolipoprotein B mRNA editing enzyme, catalytic polypeptide-like 3B
UP in Normoxia	**93436**	ARMC6	-1.814322	0.9135806	0.2843379	armadillo repeat containing 6
UP in Normoxia	**10535**	RNASEH 2A	-2.210038	0.938323	0.2161286	ribonuclease H2, subunit A
UP in Normoxia	**80318**	GKAP1	-1.966955	0.8612559	0.2557924	G kinase anchoring protein 1

UP in Normoxia	**79980**	DSN1	-2.19768	0.9356714	0.217988	DSN1, MIND kinetochore complex component, homolog (S. cerevisiae)
UP in Normoxia	**284098**	PIGW	-1.968622	0.9026068	0.2554969	phosphatidylinositol glycan anchor biosynthesis, class W
UP in Normoxia	**5383**	PMS2P5	-1.092489	0.8527636	0.4689516	postmeiotic segregation increased 2 pseudogene 5
UP in Normoxia	**10667**	FARS2	-1.22136	0.8488668	0.4288782	phenylalanyl-tRNA synthetase 2,
UP in Normoxia	**100506233**	LOC100506233	-1.402182	0.8308071	0.3783564	uncharacterized LOC100506233
UP in Normoxia	**25865**	PRKD2	-1.086304	0.8714503	0.4709664	protein kinase D2
UP in Normoxia	**84131**	CEP78	-2.015015	0.9200215	0.2474116	centrosomal protein 78kDa
UP in Normoxia	**80006**	TRAPPC13	-1.241051	0.8717459	0.4230645	trafficking protein particle complex 13
UP in Normoxia	**23670**	TMEM2	-1.760891	0.8931022	0.295066	transmembrane protein 2
UP in Normoxia	**55714**	TENM3	-1.304709	0.8170653	0.4048027	teneurin transmembrane protein 3
UP in Normoxia	**516**	ATP5G1	-1.793166	0.92184	0.2885381	ATP synthase, H+ transporting, mitochondrial Fo complex, subunit C1 (subunit 9)
UP in Normoxia	**692224**	FBXO22-AS1	-1.030053	0.8745409	0.489692	FBXO22 antisense RNA 1
UP in Normoxia	**60625**	DHX35	-1.401814	0.8677864	0.378453	DEAH (Asp-Glu-Ala-His) box polypeptide
UP in Normoxia	**3181**	HNRNPA2B1	-1.236655	0.8928335	0.4243554	heterogeneous nuclear ribonucleoprotein A2/B1
UP in Normoxia	**80209**	PROSER	-1.413559	0.8789841	0.3753846	proline and serine rich 1
UP in Normoxia	**387893**	SETD8	-1.171862	0.8768163	0.443848	SET domain containing (lysine methyltransferase) 8
UP in Normoxia	**10623**	POLR3C	-1.144655	0.8718624	0.4522977	polymerase (RNA) III (DNA directed) polypeptide C (62kD)
UP in Normoxia	**219**	ALDH1B	-1.988054	0.9074174	0.2520786	aldehyde dehydrogenase 1 family, member
UP in Normoxia	**9533**	POLR1C	-1.152095	0.8690943	0.4499712	polymerase (RNA) I polypeptide C, 30kDa
UP in Normoxia	**55651**	NHP2	-1.078115	0.8836693	0.4736474	NHP2 ribonucleoprotein
UP in Normoxia	**22809**	ATF5	-1.466339	0.8980023	0.3618994	activating transcription factor 5
UP in Normoxia	**7465**	WEE1	-1.437625	0.8834991	0.3691745	WEE1 homolog (S. pombe)
UP in Normoxia	**5366**	PMAIP1	-2.932606	0.9581116	0.1309778	phorbol-12-myristate-13-acetate-induced protein 1
UP in Normoxia	**23463**	ICMT	-1.993278	0.9202275	0.2511675	isoprenylcysteine carboxyl
UP in Normoxia	**115509**	ZNF689	-1.313547	0.8379557	0.4023304	zinc finger protein 689
UP in Normoxia	**11101**	ATE1	-2.155838	0.9178984	0.2244027	arginyltransferase 1
UP in Normoxia	**79733**	E2F8	-2.743952	0.8613097	0.1492754	E2F transcription factor 8
UP in Normoxia	**3146**	HMGB1	-1.690795	0.9189107	0.3097561	high mobility group box 1
UP in Normoxia	**94115**	CGB8	-2.773892	0.9432231	0.1462094	chorionic gonadotropin, beta polypeptide 8
UP in Normoxia	**51763**	INPP5K	-1.07926	0.8567858	0.4732716	inositol polyphosphate-5-phosphatase K
UP in Normoxia	**54505**	DHX29	-1.599732	0.9113321	0.3299383	DEAH (Asp-Glu-Ala-His) box polypeptide
UP in Normoxia	**65977**	PLEKHA3	-1.080929	0.8756159	0.4727245	pleckstrin homology domain containing, family A (phosphoinositide binding specific) member 3
UP in Normoxia	**150967**	PKI55	-1.637895	0.8950013	0.3213251	DKFZp434H1419
UP in Normoxia	**1645**	AKR1C1	-2.161529	0.9372122	0.2235192	aldo-keto reductase family 1, member C1
UP in Normoxia	**324**	APC	-1.152213	0.8534982	0.4499344	adenomatous polyposis coli
UP in Normoxia	**5987**	TRIM27	-1.067211	0.8711726	0.4772408	tripartite motif containing 27
UP in Normoxia	**114787**	GPRIN1	-1.088928	0.8562662	0.4701107	G protein regulated inducer of neurite outgrowth 1
UP in Normoxia	**57122**	NUP107	-1.620493	0.9119502	0.3252243	nucleoporin 107kDa
UP in Normoxia	**11102**	RPP14	-1.104618	0.8646063	0.4650255	ribonuclease P/MRP 14kDa subunit
UP in Normoxia	**8243**	SMC1A	-1.514522	0.8906298	0.3500123	structural maintenance of chromosomes 1A
UP in Normoxia	**10946**	SF3A3	-1.117878	0.8833289	0.4607711	splicing factor 3a, subunit 3, 60kDa
UP in Normoxia	**1075**	CTSC	-1.855901	0.9195467	0.27626	cathepsin C

UP in Normoxia	**55173**	MRPS10	-1.249993	0.8832661	0.4204504	mitochondrial ribosomal protein S10
UP in Normoxia	**10102**	TSFM	-1.11354	0.8775598	0.4621585	Ts translation elongation factor,
UP in Normoxia	**10423**	CDIPT	-1.032965	0.8785631	0.4887048	CDP-diacylglycerol--inositol 3-phosphatidyltransferase
UP in Normoxia	**80020**	FOXRED 2	-2.26039	0.8611036	0.2087156	FAD-dependent oxidoreductase domain containing 2
UP in Normoxia	**22928**	SEPHS2	-1.276054	0.8883902	0.4129234	selenophosphate synthetase 2
UP in Normoxia	**92667**	MGME1	-2.10927	0.9212219	0.2317643	mitochondrial genome maintenance exonuclease 1
UP in Normoxia	**29083**	GTPBP8	-1.022564	0.8188211	0.4922408	GTP-binding protein 8 (putative)
UP in Normoxia	**79728**	PALB2	-1.627373	0.8971065	0.3236771	partner and localizer of BRCA2
UP in Normoxia	**388552**	BLOC1S 3	-1.060576	0.8636388	0.4794406	biogenesis of lysosomal organelles complex-1, subunit 3
UP in Normoxia	**80324**	PUS1	-1.039918	0.8683687	0.486355	pseudouridylate synthase 1
UP in Normoxia	**81620**	CDT1	-2.319164	0.940043	0.2003835	chromatin licensing and DNA replication factor 1
UP in Normoxia	**55536**	CDCA7L	-1.120932	0.8749978	0.4597966	cell division cycle associated 7-like
UP in Normoxia	**7779**	SLC30A1	-2.091936	0.9306728	0.2345657	solute carrier family 30 (zinc transporter), member 1
UP in Normoxia	**55308**	DDX19A	-1.126422	0.8758936	0.4580503	DEAD (Asp-Glu-Ala-Asp) box polypeptide
UP in Normoxia	**79073**	TMEM10	-1.517603	0.902132	0.3492657	transmembrane protein 109
UP in Normoxia	**401431**	ATP6V0 E2-AS1	-3.242857	0.8105348	0.1056338	ATP6V0E2 antisense RNA 1
UP in Normoxia	**55010**	PARPBP	-2.327687	0.9313894	0.1992032	PARP1 binding protein
UP in Normoxia	**292**	SLC25A5	-1.339481	0.8976171	0.3951629	solute carrier family 25 (mitochondrial carrier; adenine nucleotide translocator), member 5
UP in Normoxia	**23212**	RRS1	-3.743445	0.9779898	0.0746639	RRS1 ribosome biogenesis regulator homolog (S. cerevisiae)
UP in Normoxia	**2175**	FANCA	-2.87434	0.9531488	0.1363758	Fanconi anemia, complementation group A
UP in Normoxia	**23276**	KLHL18	-1.214928	0.8606289	0.4307944	kelch-like family member 18
UP in Normoxia	**1353**	COX11	-1.332712	0.894213	0.3970213	cytochrome c oxidase assembly homolog 11 (yeast)
UP in Normoxia	**10293**	TRAIP	-1.615683	0.8861059	0.3263104	TRAF interacting protein
UP in Normoxia	**121268**	RHEBL1	-1.891851	0.8028487	0.2694611	Ras homolog enriched in brain like 1
UP in Normoxia	**55835**	CENPJ	-1.588357	0.817137	0.3325499	centromere protein J
UP in Normoxia	**220042**	C11orf82	-3.107626	0.9322225	0.1160142	chromosome 11 open reading frame 82
UP in Normoxia	**411**	ARSB	-1.151509	0.8632894	0.4501542	arylsulfatase B
UP in Normoxia	**220002**	CYB561	-1.799933	0.920138	0.2871878	cytochrome b561 family, member A3
UP in Normoxia	**54892**	NCAPG2	-1.679947	0.9176207	0.3120941	non-SMC condensin II complex, subunit G2
UP in Normoxia	**6634**	SNRPD3	-1.135113	0.8849503	0.4552993	small nuclear ribonucleoprotein D3 polypeptide 18kDa
UP in Normoxia	**92609**	TIMM50	-1.015333	0.8760459	0.4947142	translocase of inner mitochondrial membrane 50 homolog (S. cerevisiae)
UP in Normoxia	**51010**	EXOSC3	-1.22383	0.8835797	0.4281447	exosome component 3
UP in Normoxia	**23636**	NUP62	-1.119584	0.8810714	0.4602267	nucleoporin 62kDa
UP in Normoxia	**80135**	RPF1	-1.285629	0.8750426	0.4101919	ribosome production factor 1 homolog (S. cerevisiae)
UP in Normoxia	**1183**	CLCN4	-1.782409	0.8631819	0.2906977	chloride channel, voltage-sensitive 4
UP in Normoxia	**91869**	RFT1	-1.334995	0.8781958	0.3963935	RFT1 homolog (S. cerevisiae)
UP in Normoxia	**4825**	NKX6-1	-1.725967	0.8151214	0.3022959	NK6 homeobox 1
UP in Normoxia	**25939**	SAMHD	-2.301048	0.9221177	0.2029157	SAM domain and HD domain 1
UP in Normoxia	**11164**	NUDT5	-1.191833	0.8887396	0.4377463	nudix (nucleoside diphosphate linked moiety X)-type motif 5
UP in Normoxia	**84769**	MPV17L	-3.188918	0.9662546	0.1096579	MPV17 mitochondrial membrane protein-
UP in Normoxia	**9125**	RQCD1	-1.0851	0.8773	0.4713596	RCD1 required for cell differentiation1 homolog (S. pombe)

UP in Normoxia	**57819**	LSM2	-1.698035	0.9126937	0.3082056	LSM2 homolog, U6 small nuclear RNA associated (S. cerevisiae)
UP in Normoxia	**55605**	KIF21A	-1.427812	0.8437786	0.3716941	kinesin family member 21A
UP in Normoxia	**2669**	GEM	-1.064511	0.8812237	0.4781346	GTP binding protein overexpressed in skeletal muscle
UP in Normoxia	**5810**	RAD1	-1.265182	0.8664785	0.416047	RAD1 homolog (S. pombe)
UP in Normoxia	**11169**	WDHD1	-2.487721	0.9302965	0.1782877	WD repeat and HMG-box DNA binding protein 1
UP in Normoxia	**84939**	MUM1	-1.167887	0.8723461	0.4450727	melanoma associated antigen (mutated) 1
UP in Normoxia	**7398**	USP1	-2.257187	0.9348831	0.2091794	ubiquitin specific peptidase 1
UP in Normoxia	**56910**	STARD7	-1.378894	0.896578	0.3845135	StAR-related lipid transfer (START) domain containing 7
UP in Normoxia	**27158**	NDOR1	-1.850492	0.9119054	0.2772977	NADPH dependent diflavin oxidoreductase
UP in Normoxia	**155066**	ATP6V0	-1.603137	0.9117173	0.3291605	ATPase, H+ transporting V0 subunit e2
UP in Normoxia	**6421**	SFPQ	-1.44253	0.8996506	0.3679215	splicing factor proline/glutamine-rich
UP in Normoxia	**5433**	POLR2D	-1.146863	0.8758667	0.4516063	polymerase (RNA) II (DNA directed) polypeptide D
UP in Normoxia	**55798**	METTL2	-1.096955	0.8748903	0.4675022	methyltransferase like 2B
UP in Normoxia	**55055**	ZWILCH	-2.470676	0.9445937	0.1804066	zwilch kinetochore protein
UP in Normoxia	**4676**	NAP1L4	-1.405744	0.897859	0.3774236	nucleosome assembly protein 1-like 4
UP in Normoxia	**678**	ZFP36L2	-1.818535	0.9058318	0.2835088	ZFP36 ring finger protein-like 2
UP in Normoxia	**144110**	TMEM86 A	-2.254622	0.9255756	0.2095517	transmembrane protein 86A
UP in Normoxia	**59277**	NTN4	-2.679557	0.8369524	0.1560892	netrin 4
UP in Normoxia	**6612**	SUMO3	-1.086573	0.8820478	0.4708786	SMT3 suppressor of mif two 3 homolog 3 (S. cerevisiae)
UP in Normoxia	**1981**	EIF4G1	-1.174905	0.8892681	0.4429129	eukaryotic translation initiation factor 4 gamma, 1
UP in Normoxia	**64761**	PARP12	-1.826603	0.8290513	0.2819277	poly (ADP-ribose) polymerase family,
UP in Normoxia	**246243**	RNASEH	-1.559379	0.9007435	0.3392971	ribonuclease H1
UP in Normoxia	**84280**	BTBD10	-1.180062	0.8808833	0.4413324	BTB (POZ) domain containing 10
UP in Normoxia	**677777**	SCARNA 12	-2.139789	0.8412703	0.2269129	small Cajal body-specific RNA 12
UP in Normoxia	**984**	CDK11B	-1.113142	0.8799248	0.4622863	cyclin-dependent kinase 11B
UP in Normoxia	**440957**	SMIM4	-1.759912	0.9183374	0.2952663	small integral membrane protein 4
UP in Normoxia	**55247**	NEIL3	-2.824558	0.8977425	0.1411638	nei endonuclease VIII-like 3 (E. coli)
UP in Normoxia	**27101**	CACYBP	-1.543954	0.8969005	0.3429443	calcyclin binding protein
UP in Normoxia	**3015**	H2AFZ	-1.695702	0.9205769	0.3087044	H2A histone family, member Z
UP in Normoxia	**54938**	SARS2	-1.166274	0.8618113	0.4455706	seryl-tRNA synthetase 2, mitochondrial
UP in Normoxia	**8723**	SNX4	-1.01129	0.8630117	0.4961026	sorting nexin 4
UP in Normoxia	**158158**	RASEF	-3.121015	0.8378393	0.1149425	RAS and EF-hand domain containing
UP in Normoxia	**91624**	NEXN	-2.501729	0.9043358	0.176565	nexilin (F actin binding protein)
UP in Normoxia	**642**	BLMH	-1.142899	0.8644809	0.4528487	bleomycin hydrolase
UP in Normoxia	**90324**	CCDC97	-1.057889	0.8601899	0.4803345	coiled-coil domain containing 97
UP in Normoxia	**26164**	GTPBP5	-1.121774	0.8746842	0.4595285	GTP binding protein 5 (putative)
UP in Normoxia	**10190**	TXNDC9	-1.395705	0.8952522	0.380059	thioredoxin domain containing 9
UP in Normoxia	**55666**	NPLOC4	-1.219769	0.8908358	0.4293515	nuclear protein localization 4 homolog (S. cerevisiae)
UP in Normoxia	**55144**	LRRC8D	-1.994954	0.8821285	0.250876	leucine rich repeat containing 8 family, member D
UP in Normoxia	**728215**	FAM155	-1.416804	0.8542059	0.374541	family with sequence similarity 155,
UP in Normoxia	**23404**	EXOSC2	-1.033639	0.8721401	0.4884763	exosome component 2
UP in Normoxia	**11073**	TOPBP1	-1.269013	0.8847801	0.4149435	topoisomerase (DNA) II binding protein 1
UP in Normoxia	**9688**	NUP93	-2.202026	0.9361999	0.2173322	nucleoporin 93kDa
UP in Normoxia	**2791**	GNG11	-1.012544	0.8810087	0.4956714	guanine nucleotide binding protein (G protein), gamma 11
UP in Normoxia	**64858**	DCLRE1	-2.015788	0.9147183	0.2472791	DNA cross-link repair 1B

UP in Normoxia	**80895**	ILKAP	-1.63021	0.9120129	0.3230411	integrin-linked kinase-associated serine/threonine phosphatase
UP in Normoxia	**23078**	VWA8	-1.299295	0.8474335	0.4063246	von Willebrand factor A domain containing
UP in Normoxia	**55635**	DEPDC1	-1.541225	0.895297	0.3435935	DEP domain containing 1
UP in Normoxia	**54908**	SPDL1	-2.297753	0.9347039	0.2033796	spindle apparatus coiled-coil protein 1
UP in Normoxia	**3628**	INPP1	-1.528288	0.8385918	0.3466885	inositol polyphosphate-1-phosphatase
UP in Normoxia	**4176**	MCM7	-2.584819	0.9562842	0.1666832	minichromosome maintenance complex component 7
UP in Normoxia	**116236**	ABHD15	-1.885877	0.9051689	0.2705793	abhydrolase domain containing 15
UP in Normoxia	**928**	CD9	-1.045972	0.8819403	0.4843187	CD9 molecule
UP in Normoxia	**7274**	TTPA	-3.72792	0.8451671	0.0754717	tocopherol (alpha) transfer protein
UP in Normoxia	**54674**	LRRN3	-3.602036	0.9420586	0.0823529	leucine rich repeat neuronal 3
UP in Normoxia	**4928**	NUP98	-1.262537	0.8876467	0.4168105	nucleoporin 98kDa
UP in Normoxia	**10575**	CCT4	-1.076583	0.8840007	0.4741504	chaperonin containing TCP1, subunit 4
UP in Normoxia	**51520**	LARS	-1.065585	0.8791185	0.4777788	leucyl-tRNA synthetase
UP in Normoxia	**5690**	PSMB2	-1.144869	0.8844845	0.4522307	proteasome (prosome, macropain) subunit, beta type, 2
UP in Normoxia	**80320**	SP6	-1.066515	0.8099167	0.4774709	Sp6 transcription factor
UP in Normoxia	**84978**	FRMD5	-3.30558	0.8833736	0.1011396	FERM domain containing 5
UP in Normoxia	**2553**	GABPB1	-1.243695	0.8758577	0.4222898	GA binding protein transcription factor, beta subunit 1
UP in Normoxia	**84930**	MASTL	-1.371804	0.8826391	0.3864078	microtubule associated serine/threonine kinase-like
UP in Normoxia	**51013**	EXOSC1	-1.051986	0.8766192	0.4823039	exosome component 1
UP in Normoxia	**64897**	C12orf43	-1.3799	0.8813133	0.3842453	chromosome 12 open reading frame 43
UP in Normoxia	**3839**	KPNA3	-1.106131	0.8684314	0.4645381	karyopherin alpha 3 (importin alpha 4)
UP in Normoxia	**2289**	FKBP5	-2.630681	0.825701	0.1614679	FK506 binding protein 5
UP in Normoxia	**286333**	FAM225 A	-1.719701	0.8304846	0.3036117	family with sequence similarity 225, member A (non-protein coding)
UP in Normoxia	**2730**	GCLM	-1.534612	0.8965332	0.3451722	glutamate-cysteine ligase, modifier subunit
UP in Normoxia	**150465**	TTL	-1.27368	0.889725	0.4136033	tubulin tyrosine ligase
UP in Normoxia	**54622**	ARL15	-1.247796	0.8279136	0.4210909	ADP-ribosylation factor-like 15
UP in Normoxia	**3430**	IFI35	-1.233406	0.8783212	0.4253122	interferon-induced protein 35
UP in Normoxia	**9097**	USP14	-1.119905	0.881152	0.460124	ubiquitin specific peptidase 14 (tRNA-guanine transglycosylase)
UP in Normoxia	**55759**	WDR12	-1.422706	0.8922422	0.3730119	WD repeat domain 12
UP in Normoxia	**127703**	C1orf216	-1.050257	0.8681	0.4828821	chromosome 1 open reading frame 216
UP in Normoxia	**22827**	PUF60	-1.216692	0.8907193	0.430268	poly-U binding splicing factor 60KDa
UP in Normoxia	**116028**	RMI2	-1.810795	0.9024366	0.2850337	RMI2, RecQ mediated genome instability 2, homolog (S. cerevisiae)
UP in Normoxia	**5874**	RAB27B	-1.741997	0.9190899	0.2989556	RAB27B, member RAS oncogene family
UP in Normoxia	**28960**	DCPS	-1.437005	0.849467	0.3693333	decapping enzyme, scavenger
UP in Normoxia	**441024**	MTHFD2 L	-1.482855	0.816913	0.35778	methylenetetrahydrofolate dehydrogenase (NADP+ dependent) 2-like
UP in Normoxia	**285958**	SNHG15	-1.069436	0.8681806	0.4765053	small nucleolar RNA host gene 15 (non-protein coding)
UP in Normoxia	**23642**	SNHG1	-1.562759	0.9020514	0.3385031	small nucleolar RNA host gene 1 (non-protein coding)
UP in Normoxia	**4285**	MIPEP	-1.318788	0.8666398	0.4008715	mitochondrial intermediate peptidase
UP in Normoxia	**129293**	TRABD2 A	-1.391982	0.8671594	0.3810409	TraB domain containing 2A
UP in Normoxia	**8045**	RASSF7	-1.036168	0.8532384	0.4876211	Ras association (RalGDS/AF-6) domain family (N-terminal) member 7
UP in Normoxia	**79691**	QTRTD1	-1.441457	0.8815731	0.3681953	queuine tRNA-ribosyltransferase domain containing 1
UP in Normoxia	**60386**	SLC25A1 9	-2.791186	0.9343814	0.1444672	solute carrier family 25 (mitochondrial thiamine pyrophosphate carrier), member 19

UP in Normoxia	**80210**	ARMC9	-1.02312	0.8750605	0.4920512	armadillo repeat containing 9
UP in Normoxia	**257236**	CCDC96	-2.002281	0.8108394	0.2496051	coiled-coil domain containing 96
UP in Normoxia	**57545**	CC2D2A	-1.054145	0.8678491	0.4815825	coiled-coil and C2 domain containing 2A
UP in Normoxia	**8120**	AP3B2	-1.106057	0.8245633	0.4645619	adaptor-related protein complex 3, beta 2
UP in Normoxia	**84986**	ARHGA P19	-1.083186	0.8268835	0.4719854	Rho GTPase activating protein 19
UP in Normoxia	**4234**	METTL1	-1.022669	0.8662635	0.4922049	methyltransferase like 1
UP in Normoxia	**65083**	NOL6	-2.500781	0.9439129	0.176681	nucleolar protein 6 (RNA-associated)
UP in Normoxia	**9848**	MFAP3L	-1.933485	0.8025352	0.261796	microfibrillar-associated protein 3-like
UP in Normoxia	**9847**	C2CD5	-1.327235	0.8424707	0.3985314	C2 calcium-dependent domain containing 5
UP in Normoxia	**1763**	DNA2	-1.857835	0.8681806	0.27589	DNA replication helicase 2 homolog (yeast)
UP in Normoxia	**10999**	SLC27A4	-1.880186	0.9181224	0.2716487	solute carrier family 27 (fatty acid transporter), member 4
UP in Normoxia	**224**	ALDH3A	-2.330435	0.9385559	0.1988242	aldehyde dehydrogenase 3 family, member
UP in Normoxia	**150771**	ITPRIPL 1	-1.81953	0.8289617	0.2833133	inositol 1,4,5-trisphosphate receptor interacting protein-like 1
UP in Normoxia	**5424**	POLD1	-1.425433	0.8891158	0.3723077	polymerase (DNA directed), delta 1, catalytic subunit
UP in Normoxia	**26191**	PTPN22	-4.409752	0.9397295	0.047047	protein tyrosine phosphatase, non-receptor type 22 (lymphoid)
UP in Normoxia	**7260**	TSSC1	-1.089396	0.8753113	0.4699582	tumor suppressing subtransferable candidate
UP in Normoxia	**440248**	HERC2P	-1.7141	0.8872436	0.3047927	hect domain and RLD 2 pseudogene 9
UP in Normoxia	**1663**	DDX11	-1.431756	0.8879871	0.3706794	DEAD/H (Asp-Glu-Ala-Asp/His) box
UP in Normoxia	**51018**	RRP15	-1.271829	0.8321688	0.4141343	ribosomal RNA processing 15 homolog (S. cerevisiae)
UP in Normoxia	**310**	ANXA7	-1.377087	0.8976888	0.3849955	annexin A7
UP in Normoxia	**341880**	SLC35F4	-3.426815	0.8797635	0.0929878	solute carrier family 35, member F4
UP in Normoxia	**9704**	DHX34	-1.035381	0.8590164	0.4878872	DEAH (Asp-Glu-Ala-His) box polypeptide
UP in Normoxia	**51809**	GALNT7	-1.584963	0.8995073	0.3333333	UDP-N-acetyl-alpha-D-galactosamine: polypeptide N-acetylgalactosaminyl-transferase 7 (GalNAc-T7)
UP in Normoxia	**55526**	DHTKD1	-1.287609	0.8685479	0.4096293	dehydrogenase E1 and transketolase domain containing 1
UP in Normoxia	**22919**	MAPRE1	-1.338025	0.8965332	0.3955619	microtubule-associated protein, RP/EB family, member 1
UP in Normoxia	**57291**	DANCR	-1.03132	0.8767625	0.4892622	differentiation antagonizing non-protein coding RNA
UP in Normoxia	**10137**	RBM12	-1.103358	0.8682433	0.4654318	RNA binding motif protein 12
UP in Normoxia	**471**	ATIC	-1.823035	0.9211054	0.2826257	5-aminoimidazole-4-carboxamide ribonucleotide formyltransferase/ IMP cyclohydrolase
UP in Normoxia	**23545**	ATP6V0 A2	-1.278518	0.840464	0.4122186	ATPase, H+ transporting, lysosomal V0 subunit a2
UP in Normoxia	**28989**	NTMT1	-1.199076	0.8866792	0.435554	N-terminal Xaa-Pro-Lys N-
UP in Normoxia	**7991**	TUSC3	-2.316314	0.8008779	0.2007797	tumor suppressor candidate 3
UP in Normoxia	**1839**	HBEGF	-1.845655	0.9024993	0.2782291	heparin-binding EGF-like growth factor
UP in Normoxia	**6888**	TALDO1	-1.300431	0.8948311	0.406005	transaldolase 1
UP in Normoxia	**5984**	RFC4	-2.425185	0.9461256	0.1861858	replication factor C (activator 1) 4, 37kDa
UP in Normoxia	**94239**	H2AFV	-1.211101	0.8886679	0.4319388	H2A histone family, member V
UP in Normoxia	**10721**	POLQ	-2.269283	0.8861596	0.207433	polymerase (DNA directed), theta
UP in Normoxia	**8502**	PKP4	-1.270987	0.8803637	0.4143763	plakophilin 4
UP in Normoxia	**83461**	CDCA3	-1.49858	0.8959061	0.3539015	cell division cycle associated 3
UP in Normoxia	**5019**	OXCT1	-1.574191	0.8973484	0.3358314	3-oxoacid CoA transferase 1
UP in Normoxia	**54512**	EXOSC4	-1.65356	0.9140374	0.3178548	exosome component 4
UP in Normoxia	**9015**	TAF1A	-1.653816	0.8929768	0.3177985	TATA box binding protein (TBP)-associated factor, RNA polymerase I, A, 48kDa

UP in Normoxia	**170506**	DHX36	-1.001822	0.8707964	0.4993689	DEAH (Asp-Glu-Ala-His) box polypeptide
UP in Normoxia	**4839**	NOP2	-2.479592	0.9471737	0.1792951	NOP2 nucleolar protein
UP in Normoxia	**84254**	CAMKK 1	-1.02192	0.8508824	0.4924605	calcium/calmodulin-dependent protein kinase kinase 1, alpha
UP in Normoxia	**65975**	STK33	-2.02003	0.8842515	0.2465531	serine/threonine kinase 33
UP in Normoxia	**55110**	MAGOH	-1.113969	0.8634417	0.4620213	mago-nashi homolog B (Drosophila)
UP in Normoxia	**78996**	C7orf49	-1.393911	0.8952074	0.3805318	chromosome 7 open reading frame 49
UP in Normoxia	**10745**	PHTF1	-1.107753	0.8659052	0.4640163	putative homeodomain transcription factor 1
UP in Normoxia	**6241**	RRM2	-1.796587	0.9214279	0.2878549	ribonucleotide reductase M2
UP in Normoxia	**5817**	PVR	-1.245688	0.8879781	0.4217066	poliovirus receptor
UP in Normoxia	**133308**	SLC9B2	-1.431339	0.8095852	0.3707865	solute carrier family 9, subfamily B (NHA2, cation proton antiporter 2), member 2
UP in Normoxia	**84811**	BUD13	-1.158936	0.8418705	0.4478427	BUD13 homolog (S. cerevisiae)
UP in Normoxia	**27131**	SNX5	-1.047276	0.8774702	0.4838808	sorting nexin 5
UP in Normoxia	**64949**	MRPS26	-1.226709	0.8794768	0.4272911	mitochondrial ribosomal protein S26
UP in Normoxia	**112495**	GTF3C6	-1.490889	0.8941324	0.3557932	general transcription factor IIIC, polypeptide 6, alpha 35kDa
UP in Normoxia	**5608**	MAP2K6	-4.103691	0.8418257	0.0581655	mitogen-activated protein kinase kinase 6
UP in Normoxia	**151195**	CCNYL1	-1.250737	0.8721759	0.4202335	cyclin Y-like 1
UP in Normoxia	**100288637**	LOC1002 88637	-1.537581	0.8395413	0.3444625	OTU domain containing 7A pseudogene
UP in Normoxia	**898**	CCNE1	-1.305398	0.8427573	0.4046095	cyclin E1
UP in Normoxia	**55379**	LRRC59	-1.020427	0.880937	0.4929704	leucine rich repeat containing 59
UP in Normoxia	**26022**	TMEM98	-1.152441	0.87773	0.4498635	transmembrane protein 98
UP in Normoxia	**81572**	PDRG1	-1.0723	0.8773269	0.4755602	p53 and DNA-damage regulated 1
UP in Normoxia	**2592**	GALT	-1.211252	0.8600914	0.4318937	galactose-1-phosphate uridylyltransferase
UP in Normoxia	**9131**	AIFM1	-2.243623	0.935116	0.2111554	apoptosis-inducing factor, mitochondrion-associated, 1
UP in Normoxia	**1123**	CHN1	-1.525378	0.8724178	0.3473886	chimerin 1
UP in Normoxia	**5902**	RANBP1	-2.300184	0.9397832	0.2030372	RAN binding protein 1
UP in Normoxia	**25886**	POC1A	-1.592096	0.9018812	0.3316893	POC1 centriolar protein homolog A (Chlamydomonas)
UP in Normoxia	**348995**	NUP43	-1.581356	0.9100152	0.3341677	nucleoporin 43kDa
UP in Normoxia	**27433**	TOR2A	-1.095556	0.8359581	0.4679558	torsin family 2, member A
UP in Normoxia	**4830**	NME1	-1.722084	0.9205052	0.3031105	NME/NM23 nucleoside diphosphate kinase
UP in Normoxia	**11157**	LSM6	-1.496823	0.8894294	0.3543327	LSM6 homolog, U6 small nuclear RNA associated (S. cerevisiae)
UP in Normoxia	**6300**	MAPK12	-1.178396	0.8737795	0.4418425	mitogen-activated protein kinase 12
UP in Normoxia	**23223**	RRP12	-2.419935	0.9452925	0.1868645	ribosomal RNA processing 12 homolog (S. cerevisiae)
UP in Normoxia	**6118**	RPA2	-1.461304	0.8994894	0.3631647	replication protein A2, 32kDa
UP in Normoxia	**874**	CBR3	-1.380991	0.8817343	0.383955	carbonyl reductase 3
UP in Normoxia	**25807**	RHBDD3	-1.487949	0.8843859	0.3565189	rhomboid domain containing 3
UP in Normoxia	**10248**	POP7	-1.501024	0.8983696	0.3533024	processing of precursor 7, ribonuclease P/MRP subunit (S. cerevisiae)
UP in Normoxia	**23178**	PASK	-2.356983	0.9153364	0.195199	PAS domain containing serine/threonine
UP in Normoxia	**55341**	LSG1	-1.448009	0.8931022	0.3665268	large subunit GTPase 1 homolog (S.
UP in Normoxia	**3337**	DNAJB1	-1.299731	0.8944011	0.4062019	DnaJ (Hsp40) homolog, subfamily B,
UP in Normoxia	**11057**	ABHD2	-2.175893	0.937266	0.2213049	abhydrolase domain containing 2
UP in Normoxia	**2013**	EMP2	-1.330727	0.8785362	0.3975678	epithelial membrane protein 2
UP in Normoxia	**54329**	GPR85	-3.219678	0.8837588	0.1073446	G protein-coupled receptor 85
UP in Normoxia	**64151**	NCAPG	-2.309741	0.9349727	0.2016967	non-SMC condensin I complex, subunit G
UP in Normoxia	**3930**	LBR	-1.580688	0.9002598	0.3343225	lamin B receptor
UP in Normoxia	**93594**	WDR67	-1.977632	0.875168	0.2539062	WD repeat domain 67
UP in Normoxia	**79613**	TANGO6	-1.334743	0.8347666	0.3964628	transport and golgi organization 6 homolog (Drosophila)

UP in Normoxia	**642946**	FLVCR1-AS1	-1.677396	0.882021	0.3126464	FLVCR1 antisense RNA 1 (head to head)
UP in Normoxia	**7775**	ZNF232	-1.172978	0.8159903	0.443505	zinc finger protein 232
UP in Normoxia	**388272**	C16orf87	-1.282701	0.8023291	0.4110251	chromosome 16 open reading frame 87
UP in Normoxia	**223082**	ZNRF2	-1.025034	0.8022306	0.4913987	zinc and ring finger 2
UP in Normoxia	**283373**	ANKRD5	-1.691359	0.9158739	0.3096351	ankyrin repeat domain 52
UP in Normoxia	**24137**	KIF4A	-2.093555	0.9294455	0.2343026	kinesin family member 4A
UP in Normoxia	**112858**	TP53RK	-1.018552	0.8439936	0.4936116	TP53 regulating kinase
UP in Normoxia	**196483**	FAM86A	-2.171709	0.9133029	0.2219476	family with sequence similarity 86, member
UP in Normoxia	**6999**	TDO2	-2.138828	0.857601	0.2270642	tryptophan 2,3-dioxygenase
UP in Normoxia	**23347**	SMCHD1	-1.177677	0.8691033	0.4420628	structural maintenance of chromosomes flexible hinge domain containing 1
UP in Normoxia	**2956**	MSH6	-1.103687	0.8772642	0.4653257	mutS homolog 6 (E. coli)
UP in Normoxia	**55789**	DEPDC1	-1.975452	0.906432	0.2542902	DEP domain containing 1B
UP in Normoxia	**23649**	POLA2	-2.974714	0.957807	0.1272102	polymerase (DNA directed), alpha 2, accessory subunit
UP in Normoxia	**51056**	LAP3	-1.753322	0.9192601	0.296618	leucine aminopeptidase 3
UP in Normoxia	**26519**	TIMM10	-1.5237	0.901075	0.3477928	translocase of inner mitochondrial membrane 10 homolog (yeast)
UP in Normoxia	**6696**	SPP1	-1.334331	0.8921079	0.3965758	secreted phosphoprotein 1
UP in Normoxia	**29087**	THYN1	-1.059351	0.8760996	0.4798479	thymocyte nuclear protein 1
UP in Normoxia	**29980**	DONSO	-1.681261	0.9127206	0.31181	downstream neighbor of SON
UP in Normoxia	**2475**	MTOR	-1.654788	0.9114933	0.3175844	mechanistic target of rapamycin (serine/threonine kinase)
UP in Normoxia	**4931**	NVL	-1.263535	0.8759025	0.4165222	nuclear VCP-like
UP in Normoxia	**23474**	ETHE1	-1.114461	0.883732	0.4618637	ethylmalonic encephalopathy 1
UP in Normoxia	**55509**	BATF3	-1.387783	0.8837051	0.3821517	basic leucine zipper transcription factor, ATF-like 3
UP in Normoxia	**83695**	RHNO1	-1.120676	0.8754905	0.4598783	RAD9-HUS1-RAD1 interacting nuclear
UP in Normoxia	**96764**	TGS1	-1.352001	0.8703664	0.3917484	trimethylguanosine synthase 1
UP in Normoxia	**64146**	PDF	-1.423459	0.8682075	0.3728174	peptide deformylase (mitochondrial)
UP in Normoxia	**1287**	COL4A5	-1.55773	0.8392547	0.3396852	collagen, type IV, alpha 5
UP in Normoxia	**55729**	ATF7IP	-1.366428	0.8757682	0.3878505	activating transcription factor 7 interacting protein
UP in Normoxia	**55269**	PSPC1	-1.294273	0.8811431	0.4077416	paraspeckle component 1
UP in Normoxia	**9768**	KIAA010	-1.841241	0.9187763	0.2790817	KIAA0101
UP in Normoxia	**9824**	ARHGAP11A	-1.749061	0.917343	0.2974953	Rho GTPase activating protein 11A
UP in Normoxia	**25977**	NECAP1	-1.192578	0.8814924	0.4375204	NECAP endocytosis associated 1
UP in Normoxia	**81614**	NIPA2	-1.514519	0.8998119	0.3500131	non imprinted in Prader-Willi/Angelman syndrome 2
UP in Normoxia	**374393**	FAM111	-4.707819	0.973654	0.0382653	family with sequence similarity 111,
UP in Normoxia	**51203**	NUSAP1	-1.993361	0.9231479	0.2511532	nucleolar and spindle associated protein 1
UP in Normoxia	**136051**	ZNF786	-1.623127	0.8235868	0.3246311	zinc finger protein 786
UP in Normoxia	**256987**	SERINC5	-1.217353	0.8693362	0.4300711	serine incorporator 5
UP in Normoxia	**4154**	MBNL1	-1.124911	0.8814745	0.4585302	muscleblind-like splicing regulator 1
UP in Normoxia	**1407**	CRY1	-1.354359	0.8552898	0.3911086	cryptochrome 1 (photolyase-like)
UP in Normoxia	**54058**	C21orf58	-1.584963	0.8562752	0.3333333	chromosome 21 open reading frame 58
UP in Normoxia	**55239**	OGFOD1	-1.873649	0.9197438	0.2728824	2-oxoglutarate and iron-dependent oxygenase domain containing 1
UP in Normoxia	**55879**	GABRQ	-1.189398	0.8469139	0.4384858	gamma-aminobutyric acid (GABA) A receptor, theta
UP in Normoxia	**10257**	ABCC4	-1.763329	0.9018006	0.2945677	ATP-binding cassette, sub-family C (CFTR/MRP), member 4
UP in Normoxia	**6182**	MRPL12	-1.519607	0.9022395	0.3487809	mitochondrial ribosomal protein L12
UP in Normoxia	**4257**	MGST1	-1.853592	0.9238646	0.2767025	microsomal glutathione S-transferase 1
UP in Normoxia	**10869**	USP19	-1.09841	0.8742184	0.4670309	ubiquitin specific peptidase 19

UP in Normoxia	**57103**	C12orf5	-1.436162	0.8414673	0.3695492	chromosome 12 open reading frame 5
UP in Normoxia	**8914**	TIMELESS	-2.657047	0.9461704	0.1585437	timeless circadian clock
UP in Normoxia	**3437**	IFIT3	-1.03622	0.8185255	0.4876033	interferon-induced protein with tetratricopeptide repeats 3
UP in Normoxia	**90417**	KNSTRN	-1.874469	0.9205679	0.2727273	kinetochore-localized astrin/SPAG5 binding protein
UP in Normoxia	**2357**	FPR1	-2.635832	0.9476306	0.1608924	formyl peptide receptor 1
UP in Normoxia	**79866**	BORA	-1.274765	0.8675625	0.4132926	bora, aurora kinase A activator
UP in Normoxia	**116225**	ZMYND	-2.22597	0.9312461	0.213755	zinc finger, MYND-type containing 19
UP in Normoxia	**5883**	RAD9A	-1.372233	0.8801218	0.3862928	RAD9 homolog A (S. pombe)
UP in Normoxia	**9410**	SNRNP4	-1.460634	0.8964436	0.3633333	small nuclear ribonucleoprotein 40kDa (U5)
UP in Normoxia	**6890**	TAP1	-1.086399	0.8704381	0.4709355	transporter 1, ATP-binding cassette, sub-family B (MDR/TAP)
UP in Normoxia	**145389**	SLC38A6	-1.122521	0.8755621	0.4592906	solute carrier family 38, member 6
UP in Normoxia	**124454**	EARS2	-1.342762	0.8778106	0.3942652	glutamyl-tRNA synthetase 2, mitochondrial
UP in Normoxia	**55081**	IFT57	-1.351661	0.8910418	0.3918407	intraflagellar transport 57 homolog (Chlamydomonas)
UP in Normoxia	**970**	CD70	-1.230101	0.8829884	0.4262877	CD70 molecule
UP in Normoxia	**25914**	RTTN	-1.586982	0.8890262	0.3328671	rotatin
UP in Normoxia	**29126**	CD274	-1.882643	0.8516259	0.2711864	CD274 molecule
UP in Normoxia	**9673**	SLC25A4	-1.616696	0.9062797	0.3260815	solute carrier family 25, member 44
UP in Normoxia	**22845**	DOLK	-1.408985	0.8821912	0.3765766	dolichol kinase
UP in Normoxia	**5411**	PNN	-1.462831	0.8966138	0.3627804	pinin, desmosome associated protein
UP in Normoxia	**3363**	HTR7	-1.46658	0.8714055	0.3618391	5-hydroxytryptamine (serotonin) receptor 7, adenylate cyclase-coupled
UP in Normoxia	**27430**	MAT2B	-1.09667	0.8807668	0.4675944	methionine adenosyltransferase II, beta
UP in Normoxia	**286343**	LURAP1	-1.324525	0.8655111	0.3992806	leucine rich adaptor protein 1-like
UP in Normoxia	**8638**	OASL	-2.984811	0.9498343	0.126323	2'-5'-oligoadenylate synthetase-like
UP in Normoxia	**55238**	SLC38A7	-1.400695	0.8788498	0.3787466	solute carrier family 38, member 7
UP in Normoxia	**134510**	UBLCP1	-1.430763	0.891436	0.3709348	ubiquitin-like domain containing CTD phosphatase 1
UP in Normoxia	**64422**	ATG3	-1.180434	0.8877273	0.4412188	autophagy related 3
UP in Normoxia	**9915**	ARNT2	-1.288339	0.8794231	0.4094222	aryl-hydrocarbon receptor nuclear
UP in Normoxia	**3910**	LAMA4	-1.471445	0.8471916	0.360621	laminin, alpha 4
UP in Normoxia	**79751**	SLC25A22	-1.041955	0.8670787	0.4856688	solute carrier family 25 (mitochondrial carrier: glutamate), member 22
UP in Normoxia	**10980**	COPS6	-1.046453	0.8822897	0.484157	COP9 signalosome subunit 6
UP in Normoxia	**163786**	SASS6	-1.497846	0.8152826	0.3540816	spindle assembly 6 homolog (C. elegans)
UP in Normoxia	**60672**	MIIP	-1.537016	0.8938637	0.3445974	migration and invasion inhibitory protein
UP in Normoxia	**27229**	TUBGCP	-1.038767	0.849252	0.4867432	tubulin, gamma complex associated protein
UP in Normoxia	**56474**	CTPS2	-1.467439	0.8437517	0.3616236	CTP synthase 2
UP in Normoxia	**57552**	NCEH1	-2.038452	0.9339604	0.2434248	neutral cholesterol ester hydrolase 1
UP in Normoxia	**153129**	SLC38A9	-1.146001	0.8413599	0.451876	solute carrier family 38, member 9
UP in Normoxia	**8412**	BCAR3	-1.982876	0.9076413	0.2529851	breast cancer anti-estrogen resistance 3
UP in Normoxia	**63943**	FKBPL	-1.536511	0.8417003	0.344718	FK506 binding protein like
UP in Normoxia	**84928**	TMEM20	-1.223942	0.8861686	0.4281115	transmembrane protein 209
UP in Normoxia	**161424**	NOP9	-1.211796	0.8533548	0.4317308	NOP9 nucleolar protein
UP in Normoxia	**79022**	TMEM106C	-1.181044	0.8871719	0.4410323	transmembrane protein 106C
UP in Normoxia	**79778**	MICALL	-1.446385	0.865314	0.3669396	MICAL-like 2
UP in Normoxia	**6734**	SRPR	-1.115144	0.882648	0.4616452	signal recognition particle receptor (docking protein)
UP in Normoxia	**4691**	NCL	-1.258347	0.8936845	0.4180228	nucleolin
UP in Normoxia	**29889**	GNL2	-1.02873	0.8772731	0.4901413	guanine nucleotide binding protein-like 2 (nucleolar)
UP in Normoxia	**29901**	SAC3D1	-1.642331	0.9066201	0.3203385	SAC3 domain containing 1

UP in Normoxia	**2151**	F2RL2	-3.8009	0.8348025	0.0717489	coagulation factor II (thrombin) receptor-
UP in Normoxia	**387103**	CENPW	-1.985541	0.9238377	0.2525182	centromere protein W
UP in Normoxia	**1719**	DHFR	-1.61653	0.9152647	0.3261189	dihydrofolate reductase
UP in Normoxia	**4171**	MCM2	-2.263897	0.9373376	0.2082088	minichromosome maintenance complex component 2
UP in Normoxia	**55631**	LRRC40	-1.222136	0.8759205	0.4286475	leucine rich repeat containing 40
UP in Normoxia	**22995**	CEP152	-1.028238	0.8065395	0.4903087	centrosomal protein 152kDa
UP in Normoxia	**85302**	FBF1	-1.244955	0.8443608	0.421921	Fas (TNFRSF6) binding factor 1
UP in Normoxia	**2194**	FASN	-1.10591	0.8761802	0.4646092	fatty acid synthase
UP in Normoxia	**64854**	USP46	-1.341815	0.8484278	0.394524	ubiquitin specific peptidase 46
UP in Normoxia	**27246**	RNF115	-1.050248	0.8486518	0.4828851	ring finger protein 115
UP in Normoxia	**10495**	ENOX2	-1.24821	0.8654125	0.4209703	ecto-NOX disulfide-thiol exchanger 2
UP in Normoxia	**400506**	KNOP1	-1.840947	0.9010212	0.2791385	lysine-rich nucleolar protein 1
UP in Normoxia	**23169**	SLC35D1	-1.430577	0.8831049	0.3709826	solute carrier family 35 (UDP-glucuronic acid/UDP-N-acetylgalactosamine dual transporter), member D1
UP in Normoxia	**85461**	TANC1	-1.408112	0.8643555	0.3768044	tetratricopeptide repeat, ankyrin repeat and coiled-coil containing 1
UP in Normoxia	**114991**	ZNF618	-3.223563	0.803431	0.107056	zinc finger protein 618
UP in Normoxia	**3036**	HAS1	-2.807254	0.9626355	0.1428672	hyaluronan synthase 1
UP in Normoxia	**84975**	MFSD5	-1.301605	0.8895637	0.4056748	major facilitator superfamily domain
UP in Normoxia	**7405**	UVRAG	-1.33454	0.8083759	0.3965184	UV radiation resistance associated
UP in Normoxia	**26586**	CKAP2	-1.333814	0.883947	0.3967181	cytoskeleton associated protein 2
UP in Normoxia	**22927**	HABP4	-1.082498	0.8709845	0.4722104	hyaluronan binding protein 4
UP in Normoxia	**2146**	EZH2	-1.413757	0.8889098	0.3753329	enhancer of zeste homolog 2 (Drosophila)
UP in Normoxia	**51491**	NOP16	-2.514681	0.9461166	0.1749869	NOP16 nucleolar protein
UP in Normoxia	**54934**	KANSL2	-1.235195	0.8679208	0.4247851	KAT8 regulatory NSL complex subunit 2
UP in Normoxia	**23072**	HECW1	-2.131118	0.877085	0.228281	HECT, C2 and WW domain containing E3 ubiquitin protein ligase 1
UP in Normoxia	**366**	AQP9	-2.31982	0.9332258	0.2002924	aquaporin 9
UP in Normoxia	**57228**	SMAGP	-1.300873	0.8873421	0.4058806	small cell adhesion glycoprotein
UP in Normoxia	**645369**	TMEM200C	-1.386527	0.8206127	0.3824844	transmembrane protein 200C
UP in Normoxia	**7443**	VRK1	-1.391588	0.8937382	0.3811451	vaccinia related kinase 1
UP in Normoxia	**348180**	CTU2	-2.058612	0.924402	0.2400468	cytosolic thiouridylase subunit 2 homolog (S. pombe)
UP in Normoxia	**23303**	KIF13B	-1.000795	0.8600555	0.4997245	kinesin family member 13B
UP in Normoxia	**56255**	TMX4	-1.196089	0.8747111	0.436457	thioredoxin-related transmembrane protein 4
UP in Normoxia	**10105**	PPIF	-1.296156	0.8943474	0.4072098	peptidylprolyl isomerase F
UP in Normoxia	**23484**	LEPROTL1	-1.309673	0.8853982	0.4034123	leptin receptor overlapping transcript-like 1
UP in Normoxia	**3416**	IDE	-2.304304	0.9251814	0.2024582	insulin-degrading enzyme
UP in Normoxia	**25879**	DCAF13	-1.357064	0.8949924	0.390376	DDB1 and CUL4 associated factor 13
UP in Normoxia	**90293**	KLHL13	-2.809456	0.9249395	0.1426492	kelch-like family member 13
UP in Normoxia	**3692**	EIF6	-1.221289	0.8922601	0.4288994	eukaryotic translation initiation factor 6
UP in Normoxia	**58492**	ZNF77	-1.462544	0.8382514	0.3628527	zinc finger protein 77
UP in Normoxia	**23328**	SASH1	-1.019535	0.8691391	0.4932752	SAM and SH3 domain containing 1
UP in Normoxia	**647135**	SRGAP2	-1.949626	0.8282451	0.2588832	SLIT-ROBO Rho GTPase activating protein
UP in Normoxia	**51759**	C9orf78	-1.051682	0.8785721	0.4824056	chromosome 9 open reading frame 78
UP in Normoxia	**11284**	PNKP	-1.324591	0.8845651	0.3992623	polynucleotide kinase 3'-phosphatase
UP in Normoxia	**202**	AIM1	-1.717324	0.9047389	0.3041122	absent in melanoma 1
UP in Normoxia	**6229**	RPS24	-1.26801	0.8940697	0.4152322	ribosomal protein S24
UP in Normoxia	**285033**	LOC285033	-1.057699	0.8319627	0.4803976	uncharacterized LOC285033
UP in Normoxia	**10078**	TSSC4	-1.106044	0.8799606	0.4645661	tumor suppressing subtransferable candidate
UP in Normoxia	**11165**	NUDT3	-1.379975	0.8567052	0.3842254	nudix (nucleoside diphosphate linked moiety X)-type motif 3

UP in Normoxia	**79157**	MFSD11	-1.015538	0.8672221	0.494644	major facilitator superfamily domain containing 11
UP in Normoxia	**730394**	GTF2H2	-1.854523	0.9120577	0.276524	general transcription factor IIH, polypeptide
UP in Normoxia	**25776**	CBY1	-1.036724	0.8632805	0.4874332	chibby homolog 1 (Drosophila)
UP in Normoxia	**91687**	CENPL	-1.683526	0.8557735	0.3113208	centromere protein L
UP in Normoxia	**89795**	NAV3	-2.537724	0.9110185	0.1722142	neuron navigator 3
UP in Normoxia	**79905**	TMC7	-2.43958	0.8694347	0.1843373	transmembrane channel-like 7
UP in Normoxia	**83640**	FAM103A1	-1.268349	0.8819851	0.4151347	family with sequence similarity 103, member A1
UP in Normoxia	**84233**	TMEM126A	-1.320997	0.8902087	0.4002583	transmembrane protein 126A
UP in Normoxia	**2630**	GBAP1	-1.352611	0.8156947	0.3915828	glucosidase, beta, acid pseudogene 1
UP in Normoxia	**6636**	SNRPF	-1.235781	0.888014	0.4246125	small nuclear ribonucleoprotein polypeptide
UP in Normoxia	**51645**	PPIL1	-2.01011	0.9203171	0.2482542	peptidylprolyl isomerase (cyclophilin)-like 1
UP in Normoxia	**64398**	MPP5	-1.04853	0.8690585	0.4834606	membrane protein, palmitoylated 5 (MAGUK p55 subfamily member 5)
UP in Normoxia	**55128**	TRIM68	-1.390927	0.8703216	0.3813196	tripartite motif containing 68
UP in Normoxia	**3119**	HLA-DQB1	-1.021971	0.8660127	0.492443	major histocompatibility complex, class II, DQ beta 1
UP in Normoxia	**23530**	NNT	-1.209336	0.8336379	0.4324675	nicotinamide nucleotide transhydrogenase
UP in Normoxia	**2119**	ETV5	-1.11601	0.8775508	0.461368	ets variant 5
UP in Normoxia	**10906**	TRAFD1	-1.03978	0.8544746	0.4864017	TRAF-type zinc finger domain containing 1
UP in Normoxia	**157570**	ESCO2	-3.190594	0.9364149	0.1095306	establishment of cohesion 1 homolog 2 (S. cerevisiae)
UP in Normoxia	**135250**	RAET1E	-1.105067	0.8309953	0.464881	retinoic acid early transcript 1E
UP in Normoxia	**6339**	SCNN1D	-1.43948	0.8500493	0.3687003	sodium channel, non-voltage-gated 1, delta subunit
UP in Normoxia	**9861**	PSMD6	-1.391246	0.8981098	0.3812355	proteasome (prosome, macropain) 26S subunit, non-ATPase, 6
UP in Normoxia	**56130**	PCDHB6	-1.257259	0.8017648	0.4183381	protocadherin beta 6
UP in Normoxia	**115106**	HAUS1	-1.559301	0.8987996	0.3393155	HAUS augmin-like complex, subunit 1
UP in Normoxia	**1647**	GADD45	-1.109067	0.8839291	0.4635936	growth arrest and DNA-damage-inducible,
UP in Normoxia	**6603**	SMARCD2	-1.020817	0.8734749	0.4928371	SWI/SNF related, matrix associated, actin dependent regulator of chromatin, subfamily d, member 2
UP in Normoxia	**1303**	COL12A	-1.078649	0.8617934	0.4734719	collagen, type XII, alpha 1
UP in Normoxia	**91695**	RRP7B	-1.511828	0.8541342	0.3506667	ribosomal RNA processing 7 homolog B (S. cerevisiae)
UP in Normoxia	**170394**	PWWP2	-1.632268	0.8984861	0.3225806	PWWP domain containing 2B
UP in Normoxia	**728192**	LINC004	-1.657948	0.8566156	0.3168896	long intergenic non-protein coding RNA
UP in Normoxia	**51805**	COQ3	-1.977305	0.913052	0.2539638	coenzyme Q3 homolog, methyltransferase (S. cerevisiae)
UP in Normoxia	**1759**	DNM1	-1.011547	0.8759921	0.4960139	dynamin 1
UP in Normoxia	**6941**	TCF19	-2.01488	0.9301084	0.2474348	transcription factor 19
UP in Normoxia	**55148**	UBR7	-2.054026	0.9300546	0.2408112	ubiquitin protein ligase E3 component n-recognin 7 (putative)
UP in Normoxia	**2189**	FANCG	-1.550548	0.8961569	0.3413805	Fanconi anemia, complementation group G
UP in Normoxia	**121227**	LRIG3	-3.52686	0.8246081	0.086758	leucine-rich repeats and immunoglobulin-like domains 3
UP in Normoxia	**22795**	NID2	-1.864289	0.8736182	0.2746586	nidogen 2 (osteonidogen)
UP in Normoxia	**55320**	MIS18BP	-1.324687	0.8093613	0.3992359	MIS18 binding protein 1
UP in Normoxia	**79723**	SUV39H2	-1.171398	0.853758	0.4439909	suppressor of variegation 3-9 homolog 2 (Drosophila)
UP in Normoxia	**5427**	POLE2	-4.270529	0.9553256	0.0518135	polymerase (DNA directed), epsilon 2, accessory subunit
UP in Normoxia	**2863**	GPR39	-1.499203	0.8112962	0.3537487	G protein-coupled receptor 39

UP in Normoxia	**10197**	PSME3	-1.493001	0.9008421	0.3552727	proteasome (prosome, macropain) activator subunit 3 (PA28 gamma; Ki)
UP in Normoxia	**92345**	NAF1	-1.367094	0.84282	0.3876712	nuclear assembly factor 1 ribonucleoprotein
UP in Normoxia	**93663**	ARHGA P18	-2.82556	0.9206934	0.1410658	Rho GTPase activating protein 18
UP in Normoxia	**7516**	XRCC2	-2.078206	0.9167966	0.2368088	X-ray repair complementing defective repair in Chinese hamster cells 2
UP in Normoxia	**109**	ADCY3	-1.520595	0.8922422	0.3485422	adenylate cyclase 3
UP in Normoxia	**118460**	EXOSC6	-1.206451	0.8622951	0.4333333	exosome component 6
UP in Normoxia	**2651**	GCNT2	-3.236574	0.817137	0.1060948	glucosaminyl (N-acetyl) transferase 2, I-branching enzyme (I blood group)
UP in Normoxia	**79772**	MCTP1	-1.725939	0.9031533	0.3023016	multiple C2 domains, transmembrane 1
UP in Normoxia	**3682**	ITGAE	-1.813533	0.8263012	0.2844933	integrin, alpha E (antigen CD103, human mucosal lymphocyte antigen 1; alpha polypeptide)
UP in Normoxia	**165918**	RNF168	-1.07268	0.8380991	0.475435	ring finger protein 168, E3 ubiquitin protein ligase
UP in Normoxia	**56993**	TOMM2 2	-1.179181	0.8877811	0.4416023	translocase of outer mitochondrial membrane 22 homolog (yeast)
UP in Normoxia	**122704**	MRPL52	-1.220276	0.8866703	0.4292005	mitochondrial ribosomal protein L52
UP in Normoxia	**7351**	UCP2	-1.320918	0.8636657	0.4002802	uncoupling protein 2 (mitochondrial, proton carrier)
UP in Normoxia	**64981**	MRPL34	-1.039853	0.877524	0.486377	mitochondrial ribosomal protein L34
UP in Normoxia	**5134**	PDCD2	-1.217319	0.8880498	0.4300811	programmed cell death 2
UP in Normoxia	**991**	CDC20	-2.52162	0.9495387	0.1741473	cell division cycle 20
UP in Normoxia	**65990**	FAM173	-1.010976	0.8463675	0.4962106	family with sequence similarity 173,
UP in Normoxia	**29925**	GMPPB	-1.489089	0.8957986	0.3562374	GDP-mannose pyrophosphorylase B
UP in Normoxia	**55317**	AP5S1	-1.288073	0.8360656	0.4094976	adaptor-related protein complex 5, sigma 1 subunit
UP in Normoxia	**28969**	BZW2	-1.391336	0.8923587	0.3812116	basic leucine zipper and W2 domains 2
UP in Normoxia	**653604**	HIST2H3	-2.887525	0.8280659	0.1351351	histone cluster 2, H3d
UP in Normoxia	**55179**	FAIM	-1.17074	0.8673923	0.4441935	Fas apoptotic inhibitory molecule
UP in Normoxia	**81788**	NUAK2	-1.073176	0.8264624	0.4752714	NUAK family, SNF1-like kinase, 2
UP in Normoxia	**344595**	LOC3445 95	-1.763077	0.8896354	0.2946191	uncharacterized LOC344595
UP in Normoxia	**3396**	ICT1	-1.067042	0.8772731	0.4772965	immature colon carcinoma transcript 1
UP in Normoxia	**8898**	MTMR2	-1.078057	0.8585147	0.4736663	myotubularin related protein 2
UP in Normoxia	**100507424**	LOC1005 07424	-1.334038	0.8721491	0.3966565	uncharacterized LOC100507424
UP in Normoxia	**9392**	TGFBRA P1	-1.405131	0.8680194	0.3775838	transforming growth factor, beta receptor associated protein 1
UP in Normoxia	**51005**	AMDHD	-1.053411	0.8356535	0.4818276	amidohydrolase domain containing 2
UP in Normoxia	**7372**	UMPS	-1.361375	0.8539729	0.3892111	uridine monophosphate synthetase
UP in Normoxia	**399512**	SLC25A3	-1.1403	0.8219923	0.4536653	solute carrier family 25, member 35
UP in Normoxia	**79913**	ACTR5	-1.170808	0.8499866	0.4441724	ARP5 actin-related protein 5 homolog
UP in Normoxia	**9854**	C2CD2L	-1.509103	0.8388964	0.3513296	C2CD2-like
UP in Normoxia	**79682**	MLF1IP	-2.744409	0.9395234	0.1492281	MLF1 interacting protein
UP in Normoxia	**3308**	HSPA4	-1.768098	0.9209621	0.2935955	heat shock 70kDa protein 4
UP in Normoxia	**9093**	DNAJA3	-1.099501	0.8759025	0.4666779	DnaJ (Hsp40) homolog, subfamily A,
UP in Normoxia	**27037**	TRMT2A	-1.294766	0.8842784	0.4076023	tRNA methyltransferase 2 homolog A (S. cerevisiae)
UP in Normoxia	**10067**	SCAMP3	-1.197452	0.8887306	0.4360448	secretory carrier membrane protein 3
UP in Normoxia	**3184**	HNRNP D	-1.063654	0.8810356	0.4784188	heterogeneous nuclear ribonucleo-protein D (AU-rich element RNA binding protein 1, 37kDa)
UP in Normoxia	**10534**	SSSCA1	-1.503423	0.8990146	0.3527154	Sjogren syndrome/scleroderma autoantigen
UP in Normoxia	**58477**	SRPRB	-1.717952	0.9129445	0.3039798	signal recognition particle receptor, B

UP in Normoxia	**60496**	AASDHPPT	-1.285487	0.8751142	0.4102323	aminoadipate-semialdehyde dehydrogenase-phosphopantetheinyl transferase
UP in Normoxia	**55568**	GALNT10	-1.394752	0.8914539	0.3803101	UDP-N-acetyl-alpha-D-galactosamine:polypeptide N-acetylgalactosaminyltransferase 10 (GalNAc-T10)
UP in Normoxia	**80025**	PANK2	-1.080306	0.8630297	0.4729285	pantothenate kinase 2
UP in Normoxia	**5420**	PODXL	-1.482782	0.8972946	0.3577982	podocalyxin-like
UP in Normoxia	**221393**	GPR115	-1.784271	0.8905043	0.2903226	G protein-coupled receptor 115
UP in Normoxia	**113130**	CDCA5	-2.31152	0.9366568	0.201448	cell division cycle associated 5
UP in Normoxia	**87178**	PNPT1	-1.001861	0.8659231	0.4993554	polyribonucleotide nucleotidyltransferase 1
UP in Normoxia	**84925**	DIRC2	-1.270182	0.850327	0.4146074	disrupted in renal carcinoma 2
UP in Normoxia	**51393**	TRPV2	-5.273975	0.9675177	0.0258449	transient receptor potential cation channel, subfamily V, member 2
UP in Normoxia	**126526**	C19orf47	-1.60073	0.894652	0.3297101	chromosome 19 open reading frame 47
UP in Normoxia	**79979**	TRMT2B	-1.282575	0.868745	0.4110613	tRNA methyltransferase 2 homolog B (S. cerevisiae)
UP in Normoxia	**56123**	PCDHB1	-1.824064	0.8276718	0.2824242	protocadherin beta 13
UP in Normoxia	**56890**	MDM1	-1.683526	0.8875213	0.3113208	Mdm1 nuclear protein homolog (mouse)
UP in Normoxia	**8317**	CDC7	-1.72768	0.8760907	0.3019372	cell division cycle 7
UP in Normoxia	**79070**	KDELC1	-1.734204	0.8845561	0.3005747	KDEL (Lys-Asp-Glu-Leu) containing 1
UP in Normoxia	**54453**	RIN2	-1.044029	0.8265878	0.4849711	Ras and Rab interactor 2
UP in Normoxia	**6674**	SPAG1	-2.201848	0.8949028	0.2173591	sperm associated antigen 1
UP in Normoxia	**51184**	GPN3	-1.247479	0.8864821	0.4211836	GPN-loop GTPase 3
UP in Normoxia	**91775**	NXPE3	-1.497141	0.8861955	0.3542547	neurexophilin and PC-esterase domain family, member 3
UP in Normoxia	**389831**	LOC389831	-1.228841	0.87773	0.4266601	uncharacterized LOC389831
UP in Normoxia	**9751**	SNPH	-1.962719	0.8775508	0.2565445	syntaphilin
UP in Normoxia	**9937**	DCLRE1	-1.307649	0.828478	0.4039786	DNA cross-link repair 1A
UP in Normoxia	**7263**	TST	-1.850526	0.8882917	0.2772913	thiosulfate sulfurtransferase (rhodanese)
UP in Normoxia	**55253**	TYW1	-1.099984	0.8498164	0.4665217	tRNA-yW synthesizing protein 1 homolog (S. cerevisiae)
UP in Normoxia	**729533**	FAM72A	-1.824069	0.9002419	0.2824232	family with sequence similarity 72, member
UP in Normoxia	**92170**	MTG1	-1.22795	0.8851832	0.4269236	mitochondrial GTPase 1 homolog (S.
UP in Normoxia	**7172**	TPMT	-1.062347	0.866586	0.4788523	thiopurine S-methyltransferase
UP in Normoxia	**8692**	HYAL2	-1.346329	0.892502	0.3932916	hyaluronoglucosaminidase 2
UP in Normoxia	**51435**	SCARA3	-1.018975	0.8727045	0.4934669	scavenger receptor class A, member 3
UP in Normoxia	**144501**	KRT80	-1.880828	0.8797904	0.2715278	keratin 80
UP in Normoxia	**55692**	LUC7L	-1.086496	0.8514378	0.4709038	LUC7-like (S. cerevisiae)
UP in Normoxia	**10124**	ARL4A	-1.720263	0.8705456	0.3034934	ADP-ribosylation factor-like 4A
UP in Normoxia	**3838**	KPNA2	-1.864855	0.923766	0.2745508	karyopherin alpha 2 (RAG cohort 1, importin alpha 1)
UP in Normoxia	**285704**	RGMB	-1.710847	0.9057601	0.3054807	RGM domain family, member B
UP in Normoxia	**80222**	TARS2	-1.198764	0.8821733	0.4356483	threonyl-tRNA synthetase 2, mitochondrial (putative)
UP in Normoxia	**93323**	HAUS8	-1.9874	0.9081251	0.252193	HAUS augmin-like complex, subunit 8
UP in Normoxia	**182**	JAG1	-2.49806	0.8610141	0.1770145	jagged 1
UP in Normoxia	**10056**	FARSB	-2.232574	0.9375526	0.2127788	phenylalanyl-tRNA synthetase, beta subunit
UP in Normoxia	**9631**	NUP155	-1.712995	0.9135985	0.3050263	nucleoporin 155kDa
UP in Normoxia	**26284**	ERAL1	-1.084728	0.8796023	0.4714811	Era G-protein-like 1 (E. coli)
UP in Normoxia	**5892**	RAD51D	-2.14009	0.9123802	0.2268657	RAD51 homolog D (S. cerevisiae)
UP in Normoxia	**84705**	GTPBP3	-1.529343	0.8921706	0.3464351	GTP binding protein 3 (mitochondrial)
UP in Normoxia	**51236**	FAM203	-2.460175	0.9356087	0.1817245	family with sequence similarity 203,
UP in Normoxia	**84727**	SPSB2	-1.469788	0.8871002	0.3610354	splA/ryanodine receptor domain and SOCS box containing 2
UP in Normoxia	**9425**	CDYL	-1.335537	0.8627878	0.3962446	chromodomain protein, Y-like

UP in Normoxia	**51028**	VPS36	-1.156855	0.8243483	0.448489	vacuolar protein sorting 36 homolog (S. cerevisiae)
UP in Normoxia	**51573**	GDE1	-1.178764	0.8850219	0.4417297	glycerophosphodiester phosphodiesterase 1
UP in Normoxia	**8829**	NRP1	-1.356502	0.8966407	0.3905281	neuropilin 1
UP in Normoxia	**10435**	CDC42E	-1.016009	0.8735824	0.4944824	CDC42 effector protein (Rho GTPase
UP in Normoxia	**23395**	LARS2	-1.20955	0.8784735	0.4324034	leucyl-tRNA synthetase 2, mitochondrial
UP in Normoxia	**80011**	FAM192	-1.066943	0.8786437	0.4773293	family with sequence similarity 192,
UP in Normoxia	**23657**	SLC7A11	-2.656124	0.9061543	0.1586453	solute carrier family 7 (anionic amino acid transporter light chain, xc- system), member 11
UP in Normoxia	**51023**	MRPS18	-1.207025	0.8783839	0.4331608	mitochondrial ribosomal protein S18C
UP in Normoxia	**1537**	CYC1	-1.144059	0.8874227	0.4524847	cytochrome c-1
UP in Normoxia	**55361**	PI4K2A	-1.180285	0.8849682	0.4412643	phosphatidylinositol 4-kinase type 2 alpha
UP in Normoxia	**81610**	FAM83D	-2.04205	0.9322136	0.2428184	family with sequence similarity 83, member
UP in Normoxia	**9994**	CASP8AP2	-1.44364	0.8595449	0.3676387	caspase 8 associated protein 2
UP in Normoxia	**8872**	CDC123	-1.257356	0.892287	0.41831	cell division cycle 123
UP in Normoxia	**1277**	COL1A1	-1.364705	0.8894652	0.3883137	collagen, type I, alpha 1
UP in Normoxia	**27436**	EML4	-1.378043	0.8863209	0.3847403	echinoderm microtubule associated protein
UP in Normoxia	**386618**	KCTD4	-1.755121	0.8975723	0.2962484	potassium channel tetramerisation domain containing 4
UP in Normoxia	**9331**	B4GALT6	-2.314605	0.8883275	0.2010178	UDP-Gal:betaGlcNAc beta 1,4-galactosyltransferase, polypeptide 6
UP in Normoxia	**80018**	NAA25	-1.755413	0.9106871	0.2961884	N(alpha)-acetyltransferase 25, NatB auxiliary subunit
UP in Normoxia	**51550**	CINP	-1.010538	0.8754815	0.4963611	cyclin-dependent kinase 2 interacting
UP in Normoxia	**54834**	GDAP2	-1.035594	0.8469766	0.487815	ganglioside induced differentiation associated protein 2
UP in Normoxia	**55604**	LRRC16	-1.346411	0.8099436	0.3932692	leucine rich repeat containing 16A
UP in Normoxia	**54780**	NSMCE4A	-2.720448	0.9495655	0.1517273	non-SMC element 4 homolog A (S. cerevisiae)
UP in Normoxia	**23165**	NUP205	-1.365984	0.8951357	0.3879697	nucleoporin 205kDa
UP in Normoxia	**51514**	DTL	-3.465838	0.966371	0.0905063	denticleless E3 ubiquitin protein ligase homolog (Drosophila)
UP in Normoxia	**4008**	LMO7	-1.347845	0.8518409	0.3928783	LIM domain 7
UP in Normoxia	**9134**	CCNE2	-1.950089	0.897868	0.2588002	cyclin E2
UP in Normoxia	**79669**	C3orf52	-1.549956	0.8637821	0.3415205	chromosome 3 open reading frame 52
UP in Normoxia	**653820**	FAM72B	-1.575744	0.8921347	0.3354701	family with sequence similarity 72, member
UP in Normoxia	**1736**	DKC1	-1.602075	0.9151483	0.329403	dyskeratosis congenita 1, dyskerin
UP in Normoxia	**10236**	HNRNPR	-1.639371	0.9179074	0.3209964	heterogeneous nuclear ribonucleoprotein R
UP in Normoxia	**10153**	CEBPZ	-1.259926	0.8863746	0.4175653	CCAAT/enhancer binding protein (C/EBP),
UP in Normoxia	**79922**	MRM1	-1.118485	0.851599	0.4605773	mitochondrial rRNA methyltransferase 1 homolog (S. cerevisiae)
UP in Normoxia	**79072**	FASTKD	-1.281943	0.8595539	0.4112412	FAST kinase domains 3
UP in Normoxia	**79035**	NABP2	-1.14115	0.8848876	0.453398	nucleic acid binding protein 2
UP in Normoxia	**25959**	KANK2	-1.262836	0.8892592	0.416724	KN motif and ankyrin repeat domains 2
UP in Normoxia	**4728**	NDUFS8	-1.497449	0.9024725	0.354179	NADH dehydrogenase (ubiquinone) Fe-S protein 8, 23kDa (NADH-coenzyme Q reductase)
UP in Normoxia	**3329**	HSPD1	-1.59389	0.9181582	0.331277	heat shock 60kDa protein 1 (chaperonin)
UP in Normoxia	**152579**	SCFD2	-2.562936	0.9151393	0.1692308	sec1 family domain containing 2
UP in Normoxia	**54663**	WDR74	-1.527721	0.8995521	0.3468248	WD repeat domain 74
UP in Normoxia	**2070**	EYA4	-1.080052	0.8607095	0.4730118	eyes absent homolog 4 (Drosophila)
UP in Normoxia	**28957**	MRPS28	-1.47633	0.8980202	0.3594018	mitochondrial ribosomal protein S28
UP in Normoxia	**201266**	SLC39A11	-1.436387	0.8678044	0.3694915	solute carrier family 39 (metal ion transporter), member 11
UP in Normoxia	**2622**	GAS8	-1.424577	0.8861059	0.3725286	growth arrest-specific 8

UP in Normoxia	**55225**	RAVER2	-1.983637	0.8593658	0.2528517	ribonucleoprotein, PTB-binding 2
UP in Normoxia	**55154**	MSTO1	-2.02398	0.9300457	0.2458789	misato homolog 1 (Drosophila)
UP in Normoxia	**51493**	C22orf28	-1.201707	0.8886769	0.4347606	chromosome 22 open reading frame 28
UP in Normoxia	**114885**	OSBPL1	-1.418513	0.8723372	0.3740977	oxysterol binding protein-like 11
UP in Normoxia	**10360**	NPM3	-1.511441	0.8970169	0.3507607	nucleophosmin/nucleoplasmin 3
UP in Normoxia	**1894**	ECT2	-1.638866	0.9142614	0.3211087	epithelial cell transforming sequence 2
UP in Normoxia	**2521**	FUS	-1.662557	0.9181582	0.3158789	fused in sarcoma
UP in Normoxia	**1002**	CDH4	-2.062348	0.8902893	0.239426	cadherin 4, type 1, R-cadherin (retinal)
UP in Normoxia	**2079**	ERH	-1.515767	0.9029383	0.3497105	enhancer of rudimentary homolog
UP in Normoxia	**55920**	RCC2	-2.084599	0.9263101	0.2357616	regulator of chromosome condensation 2
UP in Normoxia	**3613**	IMPA2	-1.840087	0.9118875	0.279305	inositol(myo)-1(or 4)-monophosphatase 2
UP in Normoxia	**10726**	NUDC	-1.454861	0.9005106	0.3647902	nuclear distribution C homolog (A.
UP in Normoxia	**51421**	AMOTL2	-1.202804	0.8767446	0.43443	angiomotin like 2
UP in Normoxia	**8727**	CTNNAL 1	-1.41944	0.8973663	0.3738575	catenin (cadherin-associated protein), alpha-like 1
UP in Normoxia	**51103**	NDUFAF 1	-1.037747	0.8658783	0.4870875	NADH dehydrogenase (ubiquinone) complex I, assembly factor 1
UP in Normoxia	**6804**	STX1A	-1.527119	0.9016662	0.3469696	syntaxin 1A (brain)
UP in Normoxia	**1062**	CENPE	-1.917806	0.8679208	0.2646566	centromere protein E, 312kDa
UP in Normoxia	**79023**	NUP37	-1.775191	0.918203	0.2921556	nucleoporin 37kDa
UP in Normoxia	**10403**	NDC80	-2.259367	0.9346144	0.2088635	NDC80 kinetochore complex component
UP in Normoxia	**9675**	TTI1	-1.000542	0.8682702	0.4998122	TELO2 interacting protein 1
UP in Normoxia	**4603**	MYBL1	-1.075601	0.8514199	0.4744733	v-myb myeloblastosis viral oncogene homolog (avian)-like 1
UP in Normoxia	**64978**	MRPL38	-1.341339	0.8933441	0.3946541	mitochondrial ribosomal protein L38
UP in Normoxia	**201931**	TMEM19	-1.881507	0.8824241	0.2714001	transmembrane protein 192
UP in Normoxia	**81671**	VMP1	-1.013398	0.8780435	0.4953781	vacuole membrane protein 1
UP in Normoxia	**5686**	PSMA5	-1.497993	0.8999731	0.3540456	proteasome (prosome, macropain) subunit, alpha type, 5
UP in Normoxia	**7515**	XRCC1	-1.328355	0.8930395	0.3982221	X-ray repair complementing defective repair in Chinese hamster cells 1
UP in Normoxia	**8775**	NAPA	-1.223805	0.8913464	0.4281519	N-ethylmaleimide-sensitive factor attachment protein, alpha
UP in Normoxia	**23594**	ORC6	-2.137852	0.9335931	0.2272178	origin recognition complex, subunit 6
UP in Normoxia	**8479**	HIRIP3	-1.115114	0.8654304	0.4616546	HIRA interacting protein 3
UP in Normoxia	**10196**	PRMT3	-2.307899	0.921419	0.2019544	protein arginine methyltransferase 3
UP in Normoxia	**54532**	USP53	-1.028652	0.8381528	0.4901679	ubiquitin specific peptidase 53
UP in Normoxia	**23087**	TRIM35	-1.41558	0.8884708	0.3748589	tripartite motif containing 35
UP in Normoxia	**5498**	PPOX	-1.199247	0.8625907	0.4355025	protoporphyrinogen oxidase
UP in Normoxia	**55133**	SRBD1	-1.021359	0.8081609	0.4926522	S1 RNA binding domain 1
UP in Normoxia	**9111**	NMI	-2.580544	0.9146735	0.1671779	N-myc (and STAT) interactor
UP in Normoxia	**1434**	CSE1L	-1.619267	0.9181045	0.3255009	CSE1 chromosome segregation 1-like
UP in Normoxia	**55839**	CENPN	-1.397716	0.8908985	0.3795295	centromere protein N
UP in Normoxia	**606495**	CYB5RL	-1.733893	0.8371943	0.3006397	cytochrome b5 reductase-like
UP in Normoxia	**79159**	NOL12	-1.11904	0.8551017	0.4604	nucleolar protein 12
UP in Normoxia	**2271**	FH	-1.665037	0.9174774	0.3153364	fumarate hydratase
UP in Normoxia	**645332**	FAM86C 2P	-2.636564	0.8674819	0.1608108	family with sequence similarity 86, member A pseudogene
UP in Normoxia	**1017**	CDK2	-1.892975	0.9213563	0.2692512	cyclin-dependent kinase 2
UP in Normoxia	**9816**	URB2	-2.342392	0.8998477	0.1971831	URB2 ribosome biogenesis 2 homolog (S. cerevisiae)
UP in Normoxia	**162**	AP1B1	-1.036158	0.8775329	0.4876244	adaptor-related protein complex 1, beta 1
UP in Normoxia	**119559**	SFXN4	-1.915807	0.9085819	0.2650236	sideroflexin 4
UP in Normoxia	**55732**	C1orf112	-2.058894	0.8680462	0.24	chromosome 1 open reading frame 112
UP in Normoxia	**80178**	C16orf59	-2.931722	0.9286303	0.131058	chromosome 16 open reading frame 59
UP in Normoxia	**10253**	SPRY2	-1.678931	0.9190719	0.312314	sprouty homolog 2 (Drosophila)
UP in Normoxia	**54535**	CCHCR1	-1.202622	0.871316	0.4344848	coiled-coil alpha-helical rod protein 1

UP in Normoxia	**10465**	PPIH	-1.197865	0.8852638	0.43592	peptidylprolyl isomerase H (cyclophilin H)
UP in Normoxia	**91752**	ZNF804	-1.048075	0.8497895	0.4836131	zinc finger protein 804A
UP in Normoxia	**10587**	TXNRD2	-1.150413	0.8729822	0.4504962	thioredoxin reductase 2
UP in Normoxia	**340252**	ZNF680	-1.263439	0.83233	0.4165498	zinc finger protein 680
UP in Normoxia	**692223**	SNORD9	-7.022368	0.8410284	0.0076923	small nucleolar RNA, C/D box 97
UP in Normoxia	**51524**	TMEM13	-2.287566	0.9358775	0.2048208	transmembrane protein 138
UP in Normoxia	**172**	AFG3L1 P	-1.34409	0.8498522	0.3939024	AFG3 ATPase family member 3-like 1 (S. cerevisiae), pseudogene
UP in Normoxia	**29920**	PYCR2	-1.288081	0.8912747	0.4094953	pyrroline-5-carboxylate reductase family, member 2
UP in Normoxia	**9735**	KNTC1	-1.90593	0.9056974	0.2668443	kinetochore associated 1
UP in Normoxia	**26953**	RANBP6	-1.378055	0.8729016	0.3847371	RAN binding protein 6
UP in Normoxia	**4999**	ORC2	-1.290318	0.8749619	0.4088608	origin recognition complex, subunit 2
UP in Normoxia	**51454**	GULP1	-1.695607	0.8888023	0.3087248	GULP, engulfment adaptor PTB domain containing 1
UP in Normoxia	**9530**	BAG4	-1.067551	0.8216429	0.4771283	BCL2-associated athanogene 4
UP in Normoxia	**112849**	L3HYPD	-1.219409	0.8846278	0.4294587	L-3-hydroxyproline dehydratase (trans-)
UP in Normoxia	**55210**	ATAD3A	-2.028977	0.9349816	0.2450287	ATPase family, AAA domain containing 3A
UP in Normoxia	**2294**	FOXF1	-1.603635	0.9114933	0.3290469	forkhead box F1
UP in Normoxia	**839**	CASP6	-1.508563	0.8382424	0.351461	caspase 6, apoptosis-related cysteine
UP in Normoxia	**51535**	PPHLN1	-1.114057	0.8807131	0.4619931	periphilin 1
UP in Normoxia	**79674**	VEPH1	-1.058419	0.8690047	0.4801579	ventricular zone expressed PH domain-containing 1
UP in Normoxia	**57574**	MARCH 4	-1.229188	0.8579772	0.4265575	membrane-associated ring finger (C3HC4) 4, E3 ubiquitin protein ligase
UP in Normoxia	**147841**	SPC24	-3.078792	0.9621607	0.1183563	SPC24, NDC80 kinetochore complex component, homolog (S. cerevisiae)
UP in Normoxia	**57019**	CIAPIN1	-1.735035	0.9176565	0.3004017	cytokine induced apoptosis inhibitor 1
UP in Normoxia	**7004**	TEAD4	-1.460778	0.8789662	0.3632973	TEA domain family member 4
UP in Normoxia	**79813**	EHMT1	-1.051587	0.8542775	0.4824373	euchromatic histone-lysine N-methyltransferase 1
UP in Normoxia	**3185**	HNRNPF	-1.38871	0.8978052	0.3819063	heterogeneous nuclear ribonucleoprotein F
UP in Normoxia	**3109**	HLA-DMB	-2.574867	0.9128998	0.1678371	major histocompatibility complex, class II, DM beta
UP in Normoxia	**10376**	TUBA1B	-1.249091	0.893362	0.4207132	tubulin, alpha 1b
UP in Normoxia	**3431**	SP110	-1.665081	0.8773358	0.3153266	SP110 nuclear body protein
UP in Normoxia	**80727**	TTYH3	-1.475189	0.8977246	0.3596862	tweety homolog 3 (Drosophila)
UP in Normoxia	**9126**	SMC3	-1.142638	0.881591	0.4529307	structural maintenance of chromosomes 3
UP in Normoxia	**9399**	STOML1	-2.041119	0.9219654	0.2429752	stomatin (EPB72)-like 1
UP in Normoxia	**79902**	NUP85	-1.869049	0.9193317	0.2737539	nucleoporin 85kDa
UP in Normoxia	**55859**	BEX1	-2.049955	0.9350085	0.2414917	brain expressed, X-linked 1
UP in Normoxia	**3149**	HMGB3	-1.723892	0.9065036	0.302731	high mobility group box 3
UP in Normoxia	**253558**	LCLAT1	-1.024887	0.8347756	0.4914487	lysocardiolipin acyltransferase 1
UP in Normoxia	**55520**	ELAC1	-1.791683	0.8252262	0.288835	elaC homolog 1 (E. coli)
UP in Normoxia	**100302739**	PCNA-AS1	-2.766592	0.9560692	0.1469511	PCNA antisense RNA 1
UP in Normoxia	**100130015**	LOC1001 30015	-1.151898	0.8280928	0.4500327	5-hydroxyisourate hydrolase pseudogene
UP in Normoxia	**4144**	MAT2A	-1.185465	0.8890442	0.4396829	methionine adenosyltransferase II, alpha
UP in Normoxia	**7466**	WFS1	-1.101283	0.8762877	0.4661017	Wolfram syndrome 1 (wolframin)
UP in Normoxia	**80218**	NAA50	-1.290835	0.8917227	0.4087144	N(alpha)-acetyltransferase 50, NatE catalytic subunit
UP in Normoxia	**6837**	MED22	-2.172463	0.9312461	0.2218317	mediator complex subunit 22
UP in Normoxia	**79828**	METTL8	-1.202255	0.8071576	0.4345955	methyltransferase like 8
UP in Normoxia	**4953**	ODC1	-1.785224	0.9222163	0.2901309	ornithine decarboxylase 1
UP in Normoxia	**83858**	ATAD3B	-2.01403	0.9319269	0.2475806	ATPase family, AAA domain containing 3B

UP in Normoxia	**5713**	PSMD7	-1.165682	0.8881931	0.4457534	proteasome (prosome, macropain) 26S subunit, non-ATPase, 7
UP in Normoxia	**262**	AMD1	-2.579335	0.9545463	0.1673181	adenosylmethionine decarboxylase 1
UP in Normoxia	**84166**	NLRC5	-1.579609	0.8434561	0.3345725	NLR family, CARD domain containing 5
UP in Normoxia	**89891**	WDR34	-1.785587	0.9206307	0.2900579	WD repeat domain 34
UP in Normoxia	**27339**	PRPF19	-1.45326	0.8995969	0.3651952	PRP19/PSO4 pre-mRNA processing factor 19 homolog (S. cerevisiae)
UP in Normoxia	**84057**	MND1	-1.979968	0.8896623	0.2534954	meiotic nuclear divisions 1 homolog (S. cerevisiae)
UP in Normoxia	**57546**	PDP2	-1.040249	0.8286124	0.4862437	pyruvate dehyrogenase phosphatase catalytic subunit 2
UP in Normoxia	**79050**	NOC4L	-1.539538	0.8954045	0.3439956	nucleolar complex associated 4 homolog (S. cerevisiae)
UP in Normoxia	**8891**	EIF2B3	-1.624164	0.9105886	0.3243978	eukaryotic translation initiation factor 2B, subunit 3 gamma, 58kDa
UP in Normoxia	**84365**	MKI67IP	-1.132791	0.8787691	0.4560327	MKI67 (FHA domain) interacting nucleolar phosphoprotein
UP in Normoxia	**55388**	MCM10	-4.357986	0.964463	0.0487658	minichromosome maintenance complex component 10
UP in Normoxia	**84172**	POLR1B	-2.195378	0.9111171	0.218336	polymerase (RNA) I polypeptide B, 128kDa
UP in Normoxia	**64403**	CDH24	-1.526104	0.8868494	0.3472137	cadherin 24, type 2
UP in Normoxia	**6874**	TAF4	-1.237179	0.832984	0.4242012	TAF4 RNA polymerase II, TATA box binding protein (TBP)-associated factor, 135kDa
UP in Normoxia	**6434**	TRA2B	-1.259224	0.8883544	0.4177687	transformer 2 beta homolog (Drosophila)
UP in Normoxia	**89978**	ATPBD4	-1.469012	0.831058	0.3612297	ATP binding domain 4
UP in Normoxia	**55248**	TMEM20	-1.252868	0.8762698	0.4196133	transmembrane protein 206
UP in Normoxia	**57630**	SH3RF1	-1.353298	0.8399176	0.3913963	SH3 domain containing ring finger 1
UP in Normoxia	**54955**	C1orf109	-1.414756	0.8826839	0.3750731	chromosome 1 open reading frame 109
UP in Normoxia	**1718**	DHCR24	-1.750383	0.9114844	0.2972229	24-dehydrocholesterol reductase
UP in Normoxia	**23160**	WDR43	-1.375007	0.8918749	0.3855508	WD repeat domain 43
UP in Normoxia	**79573**	TTC13	-1.338209	0.8610857	0.3955112	tetratricopeptide repeat domain 13
UP in Normoxia	**10938**	EHD1	-1.387531	0.8984323	0.3822183	EH-domain containing 1
UP in Normoxia	**724102**	SNHG4	-1.395373	0.8679477	0.3801464	small nucleolar RNA host gene 4 (non-protein coding)
UP in Normoxia	**9088**	PKMYT1	-3.036461	0.956965	0.1218804	protein kinase, membrane associated tyrosine/threonine 1
UP in Normoxia	**11198**	SUPT16	-1.153984	0.8825764	0.4493824	suppressor of Ty 16 homolog (S. cerevisiae)
UP in Normoxia	**81892**	SLIRP	-1.21595	0.8918929	0.4304894	SRA stem-loop interacting RNA binding
UP in Normoxia	**64283**	ARHGEF 28	-1.86576	0.8974111	0.2743786	Rho guanine nucleotide exchange factor (GEF) 28
UP in Normoxia	**158**	ADSL	-1.2897	0.8917585	0.409036	adenylosuccinate lyase
UP in Normoxia	**26151**	NAT9	-1.091241	0.8755084	0.4693575	N-acetyltransferase 9 (GCN5-related,
UP in Normoxia	**27079**	RPUSD2	-1.541832	0.8727134	0.3434489	RNA pseudouridylate synthase domain containing 2
UP in Normoxia	**5696**	PSMB8	-1.575089	0.9129445	0.3356224	proteasome (prosome, macropain) subunit, beta type, 8
UP in Normoxia	**146712**	B3GNTL 1	-1.160694	0.8526203	0.4472973	UDP-GlcNAc:betaGal beta-1,3-N-acetylglucosaminyltransferase-like 1
UP in Normoxia	**3918**	LAMC2	-3.036134	0.8486518	0.1219081	laminin, gamma 2
UP in Normoxia	**79650**	USB1	-1.620758	0.9166532	0.3251646	U6 snRNA biogenesis 1
UP in Normoxia	**2224**	FDPS	-1.012369	0.8792618	0.4957317	farnesyl diphosphate synthase
UP in Normoxia	**5080**	PAX6	-2.191912	0.80086	0.2188612	paired box 6
UP in Normoxia	**4998**	ORC1	-2.634646	0.9370689	0.1610247	origin recognition complex, subunit 1
UP in Normoxia	**8555**	CDC14B	-1.24726	0.8512138	0.4212474	cell division cycle 14B
UP in Normoxia	**9928**	KIF14	-1.263034	0.8326704	0.4166667	kinesin family member 14
UP in Normoxia	**55312**	RFK	-1.532824	0.8975186	0.3456002	riboflavin kinase

UP in Normoxia	**995**	CDC25C	-1.976413	0.883087	0.2541209	cell division cycle 25C
UP in Normoxia	**84969**	TOX2	-1.694557	0.8639792	0.3089494	TOX high mobility group box family
UP in Normoxia	**388121**	TNFAIP8L3	-1.207956	0.867661	0.4328814	tumor necrosis factor, alpha-induced protein 8-like 3
UP in Normoxia	**100526837**	EEF1E1-MUTED	-8.173261	0.9415928	0.0034642	EEF1E1-MUTED readthrough
UP in Normoxia	**728492**	SERF1B	-1.188616	0.8832572	0.4387235	small EDRK-rich factor 1B (centromeric)
UP in Normoxia	**80304**	C2orf44	-1.321059	0.849682	0.4002411	chromosome 2 open reading frame 44
UP in Normoxia	**23005**	MAPKBP1	-1.462798	0.8759742	0.3627888	mitogen-activated protein kinase binding protein 1
UP in Normoxia	**580**	BARD1	-1.777608	0.8701962	0.2916667	BRCA1 associated RING domain 1
UP in Normoxia	**6632**	SNRPD1	-2.010045	0.9336827	0.2482654	small nuclear ribonucleoprotein D1 polypeptide 16kDa
UP in Normoxia	**135114**	HINT3	-1.221996	0.8620174	0.4286892	histidine triad nucleotide binding protein 3
UP in Normoxia	**29063**	ZCCHC4	-1.127167	0.8671056	0.4578139	zinc finger, CCHC domain containing 4
UP in Normoxia	**7019**	TFAM	-1.350025	0.874514	0.3922853	transcription factor A, mitochondrial
UP in Normoxia	**54606**	DDX56	-1.449866	0.897859	0.3660555	DEAD (Asp-Glu-Ala-Asp) box helicase 56
UP in Normoxia	**91801**	ALKBH8	-1.372939	0.8212667	0.3861038	alkB, alkylation repair homolog 8 (E. coli)
UP in Normoxia	**51582**	AZIN1	-1.112139	0.8825764	0.4626077	antizyme inhibitor 1
UP in Normoxia	**1374**	CPT1A	-1.7968	0.917979	0.2878122	carnitine palmitoyltransferase 1A (liver)
UP in Normoxia	**9263**	STK17A	-1.52572	0.8983159	0.3473061	serine/threonine kinase 17a
UP in Normoxia	**84455**	EFCAB7	-1.49963	0.860172	0.353644	EF-hand calcium binding domain 7
UP in Normoxia	**523**	ATP6V1A	-1.254287	0.8877273	0.4192005	ATPase, H+ transporting, lysosomal 70kDa, V1 subunit A
UP in Normoxia	**414189**	AGAP6	-1.225629	0.8587118	0.4276109	ArfGAP with GTPase domain, ankyrin repeat and PH domain 6
UP in Normoxia	**90459**	ERI1	-1.313256	0.8786527	0.4024117	exoribonuclease 1
UP in Normoxia	**65263**	PYCRL	-3.403856	0.9610589	0.0944794	pyrroline-5-carboxylate reductase-like
UP in Normoxia	**835**	CASP2	-1.4616	0.8858819	0.3630901	caspase 2, apoptosis-related cysteine
UP in Normoxia	**1267**	CNP	-1.043604	0.8734301	0.4851141	2',3'-cyclic nucleotide 3' phosphodiesterase
UP in Normoxia	**54927**	CHCHD3	-1.039094	0.8808743	0.4866329	coiled-coil-helix-coiled-coil-helix domain containing 3
UP in Normoxia	**6895**	TARBP2	-1.298389	0.8883006	0.40658	TAR (HIV-1) RNA binding protein 2
UP in Normoxia	**654364**	NME1-NME2	-1.261502	0.8839201	0.4171094	NME1-NME2 readthrough
UP in Normoxia	**729438**	GATSL2	-2.371969	0.8306817	0.1931818	GATS protein-like 2
UP in Normoxia	**201725**	C4orf46	-1.683385	0.8329123	0.3113514	chromosome 4 open reading frame 46
UP in Normoxia	**57082**	CASC5	-1.987882	0.9004927	0.2521087	cancer susceptibility candidate 5
UP in Normoxia	**5255**	PHKA1	-1.89388	0.8733584	0.2690825	phosphorylase kinase, alpha 1 (muscle)
UP in Normoxia	**56919**	DHX33	-1.697216	0.9057064	0.3083806	DEAH (Asp-Glu-Ala-His) box polypeptide
UP in Normoxia	**4796**	TONSL	-2.097186	0.9207561	0.2337137	tonsoku-like, DNA repair protein
UP in Normoxia	**56942**	CMC2	-1.227385	0.8895369	0.4270908	COX assembly mitochondrial protein 2 homolog (S. cerevisiae)
UP in Normoxia	**1676**	DFFA	-1.360773	0.8903521	0.3893735	DNA fragmentation factor, 45kDa, alpha polypeptide
UP in Normoxia	**23658**	LSM5	-1.349584	0.8832841	0.3924051	LSM5 homolog, U6 small nuclear RNA associated (S. cerevisiae)
UP in Normoxia	**3608**	ILF2	-1.476416	0.9013616	0.3593806	interleukin enhancer binding factor 2
UP in Normoxia	**498**	ATP5A1	-1.012477	0.8808654	0.4956945	ATP synthase, H+ transporting, mitochondrial F1 complex, alpha subunit 1, cardiac muscle
UP in Normoxia	**790**	CAD	-1.842074	0.9168503	0.2789205	carbamoyl-phosphate synthetase 2, aspartate transcarbamylase, and dihydroorotase
UP in Normoxia	**79084**	WDR77	-1.96119	0.9212846	0.2568166	WD repeat domain 77
UP in Normoxia	**5932**	RBBP8	-1.168429	0.8786885	0.4449057	retinoblastoma binding protein 8
UP in Normoxia	**83877**	TM2D2	-1.051342	0.8651527	0.4825193	TM2 domain containing 2

UP in Normoxia	**494143**	CHAC2	-3.462734	0.9403207	0.0907012	ChaC, cation transport regulator homolog 2 (E. coli)
UP in Normoxia	**25851**	TECPR1	-1.023322	0.8538923	0.4919823	tectonin beta-propeller repeat containing 1
UP in Normoxia	**51022**	GLRX2	-1.31779	0.8760279	0.401149	glutaredoxin 2
UP in Normoxia	**57482**	KIAA121	-2.007777	0.8336021	0.2486559	KIAA1211
UP in Normoxia	**51750**	RTEL1	-1.871522	0.8912658	0.273285	regulator of telomere elongation helicase 1
UP in Normoxia	**64837**	KLC2	-1.085091	0.8799248	0.4713625	kinesin light chain 2
UP in Normoxia	**5891**	MOK	-1.495487	0.893783	0.3546611	MOK protein kinase
UP in Normoxia	**4288**	MKI67	-2.142218	0.9345785	0.2265313	antigen identified by monoclonal antibody
UP in Normoxia	**23076**	RRP1B	-1.10339	0.87902	0.4654217	ribosomal RNA processing 1 homolog B (S. cerevisiae)
UP in Normoxia	**81894**	SLC25A2 8	-1.849765	0.9134552	0.2774376	solute carrier family 25 (mitochondrial iron transporter), member 28
UP in Normoxia	**55572**	FOXRED 1	-1.894139	0.9113052	0.2690341	FAD-dependent oxidoreductase domain containing 1
UP in Normoxia	**63922**	CHTF18	-2.452068	0.9092807	0.1827485	CTF18, chromosome transmission fidelity factor 18 homolog (S. cerevisiae)
UP in Normoxia	**9532**	BAG2	-2.512192	0.9381976	0.1752891	BCL2-associated athanogene 2
UP in Normoxia	**51182**	HSPA14	-1.70308	0.9116008	0.3071298	heat shock 70kDa protein 14
UP in Normoxia	**2122**	MECOM	-2.121991	0.8237302	0.2297297	MDS1 and EVI1 complex locus
UP in Normoxia	**79888**	LPCAT1	-1.253884	0.8876915	0.4193179	lysophosphatidylcholine acyltransferase 1
UP in Normoxia	**10426**	TUBGCP	-1.340767	0.8754457	0.3948107	tubulin, gamma complex associated protein
UP in Normoxia	**25842**	ASF1A	-1.896561	0.9162232	0.2685828	ASF1 anti-silencing function 1 homolog A (S. cerevisiae)
UP in Normoxia	**25764**	HYPK	-1.560638	0.900421	0.3390012	huntingtin interacting protein K
UP in Normoxia	**55794**	DDX28	-1.563689	0.8556123	0.3382849	DEAD (Asp-Glu-Ala-Asp) box polypeptide
UP in Normoxia	**9498**	SLC4A8	-1.394483	0.8423363	0.3803811	solute carrier family 4, sodium bicarbonate cotransporter, member 8
UP in Normoxia	**1147**	CHUK	-1.180788	0.8780883	0.4411104	conserved helix-loop-helix ubiquitous kinase
UP in Normoxia	**4675**	NAP1L3	-2.255257	0.862743	0.2094595	nucleosome assembly protein 1-like 3
UP in Normoxia	**7307**	U2AF1	-1.227907	0.8918749	0.4269365	U2 small nuclear RNA auxiliary factor 1
UP in Normoxia	**23678**	SGK3	-2.887525	0.8814924	0.1351351	serum/glucocorticoid regulated kinase family, member 3
UP in Normoxia	**8520**	HAT1	-2.158155	0.9365851	0.2240426	histone acetyltransferase 1
UP in Normoxia	**10200**	MPHOSP H6	-1.121972	0.8807758	0.4594654	M-phase phosphoprotein 6
UP in Normoxia	**54093**	SETD4	-1.126745	0.8552988	0.4579477	SET domain containing 4
UP in Normoxia	**29071**	C1GALT 1C1	-1.020262	0.8629849	0.4930269	C1GALT1-specific chaperone 1
UP in Normoxia	**10629**	TAF6L	-1.119873	0.8463764	0.4601345	TAF6-like RNA polymerase II, p300/ CBP-associated factor (PCAF)- associated factor, 65kDa
UP in Normoxia	**55170**	PRMT6	-2.828764	0.8516886	0.1407529	protein arginine methyltransferase 6
UP in Normoxia	**10884**	MRPS30	-1.308496	0.89037	0.4037416	mitochondrial ribosomal protein S30
UP in Normoxia	**6252**	RTN1	-1.125473	0.8510615	0.4583517	reticulon 1
UP in Normoxia	**9830**	TRIM14	-1.043193	0.8618113	0.4852524	tripartite motif containing 14
UP in Normoxia	**51099**	ABHD5	-1.044724	0.8479441	0.4847375	abhydrolase domain containing 5
UP in Normoxia	**100507118**	LOC1005 07118	-2.154818	0.9062528	0.2245614	uncharacterized LOC100507118
UP in Normoxia	**10084**	PQBP1	-1.007612	0.8756786	0.4973687	polyglutamine binding protein 1
UP in Normoxia	**4927**	NUP88	-1.744984	0.9169488	0.2983372	nucleoporin 88kDa
UP in Normoxia	**375484**	SIMC1	-1.112261	0.8294813	0.4625685	SUMO-interacting motifs containing 1
UP in Normoxia	**64425**	POLR1E	-1.809121	0.9136343	0.2853648	polymerase (RNA) I polypeptide E, 53kDa
UP in Normoxia	**85465**	EPT1	-1.670207	0.9079638	0.3142083	ethanolaminephosphotransferase 1 (CDP-ethanolamine-specific)
UP in Normoxia	**90826**	PRMT10	-1.664816	0.8304578	0.3153846	protein arginine methyltransferase 10
UP in Normoxia	**9841**	ZBTB24	-1.301464	0.8501747	0.4057143	zinc finger and BTB domain containing 24

UP in Normoxia	**23761**	PISD	-1.509257	0.8907731	0.3512921	phosphatidylserine decarboxylase
UP in Normoxia	**84885**	ZDHHC1	-1.37924	0.896148	0.3844213	zinc finger, DHHC-type containing 12
UP in Normoxia	**56983**	POGLUT	-1.288996	0.8233987	0.4092357	protein O-glucosyltransferase 1
UP in Normoxia	**64963**	MRPS11	-1.123925	0.8804622	0.4588437	mitochondrial ribosomal protein S11
UP in Normoxia	**256227**	STEAP1	-2.280026	0.9321688	0.2058941	STEAP family member 1B
UP in Normoxia	**6428**	SRSF3	-1.38117	0.8965242	0.3839072	serine/arginine-rich splicing factor 3
UP in Normoxia	**51239**	ANKRD3	-1.406377	0.8824062	0.3772579	ankyrin repeat domain 39
UP in Normoxia	**79703**	C11orf80	-1.179259	0.8178357	0.4415781	chromosome 11 open reading frame 80
UP in Normoxia	**84296**	GINS4	-3.107935	0.9606916	0.1159894	GINS complex subunit 4 (Sld5 homolog)
UP in Normoxia	**5167**	ENPP1	-1.015328	0.81307	0.494716	ectonucleotide pyrophosphatase/phosphodiesterase 1
UP in Normoxia	**378708**	APITD1	-1.565138	0.8964526	0.3379453	apoptosis-inducing, TAF9-like domain 1
UP in Normoxia	**26073**	POLDIP2	-1.039364	0.8806324	0.486542	polymerase (DNA-directed), delta interacting protein 2
UP in Normoxia	**285331**	CCDC66	-1.099094	0.8151214	0.4668094	coiled-coil domain containing 66
UP in Normoxia	**1728**	NQO1	-1.56922	0.9037266	0.3369905	NAD(P)H dehydrogenase, quinone 1
UP in Normoxia	**9232**	PTTG1	-1.620879	0.9182747	0.3251373	pituitary tumor-transforming 1
UP in Normoxia	**26233**	FBXL6	-1.448215	0.8838395	0.3664747	F-box and leucine-rich repeat protein 6
UP in Normoxia	**64641**	EBF2	-1.698445	0.8932814	0.3081181	early B-cell factor 2
UP in Normoxia	**54069**	MIS18A	-1.449165	0.8911852	0.3662333	MIS18 kinetochore protein homolog A (S. pombe)
UP in Normoxia	**88745**	RRP36	-1.741104	0.9184897	0.2991407	ribosomal RNA processing 36 homolog (S. cerevisiae)
UP in Normoxia	**27340**	UTP20	-1.756286	0.9080176	0.2960092	UTP20, small subunit (SSU) processome component, homolog (yeast)
UP in Normoxia	**55852**	TEX2	-1.240624	0.8673744	0.4231895	testis expressed 2
UP in Normoxia	**55215**	FANCI	-1.939805	0.9194482	0.2606516	Fanconi anemia, complementation group I
UP in Normoxia	**1993**	ELAVL2	-3.151412	0.8435456	0.1125461	ELAV (embryonic lethal, abnormal vision, Drosophila)-like 2 (Hu antigen B)
UP in Normoxia	**84844**	PHF5A	-1.337805	0.8931739	0.3956222	PHD finger protein 5A
UP in Normoxia	**140459**	ASB6	-1.014266	0.869157	0.4950802	ankyrin repeat and SOCS box containing 6
UP in Normoxia	**339230**	CCDC13	-1.514024	0.8990056	0.3501332	coiled-coil domain containing 137
UP in Normoxia	**84913**	ATOH8	-3.388585	0.9387799	0.0954848	atonal homolog 8 (Drosophila)
UP in Normoxia	**65220**	NADK	-1.093021	0.8776942	0.4687786	NAD kinase
UP in Normoxia	**10217**	CTDSPL	-2.196223	0.8317119	0.2182081	CTD (carboxy-terminal domain, RNA polymerase II, polypeptide A) small phosphatase-like
UP in Normoxia	**10471**	PFDN6	-1.22612	0.8901012	0.4274654	prefoldin subunit 6
UP in Normoxia	**60491**	NIF3L1	-1.079539	0.8726955	0.4731801	NIF3 NGG1 interacting factor 3-like 1 (S. cerevisiae)
UP in Normoxia	**348235**	SKA2	-1.80575	0.9173341	0.2860324	spindle and kinetochore associated complex subunit 2
UP in Normoxia	**54463**	FAM134	-1.438303	0.8794768	0.3690011	family with sequence similarity 134,
UP in Normoxia	**5501**	PPP1CC	-1.013066	0.8784825	0.4954922	protein phosphatase 1, catalytic subunit, gamma isozyme
UP in Normoxia	**9368**	SLC9A3 R1	-1.049673	0.8619905	0.4830778	solute carrier family 9, subfamily A (NHE3, cation proton antiporter 3), member 3 regulator 1
UP in Normoxia	**57102**	C12orf4	-1.227197	0.8602974	0.4271466	chromosome 12 open reading frame 4
UP in Normoxia	**1869**	E2F1	-2.907947	0.9604049	0.1332357	E2F transcription factor 1
UP in Normoxia	**10868**	USP20	-1.155638	0.863594	0.4488675	ubiquitin specific peptidase 20
UP in Normoxia	**283254**	HARBI1	-1.60526	0.86423	0.3286765	harbinger transposase derived 1
UP in Normoxia	**26292**	MYCBP	-1.021637	0.8550479	0.4925571	c-myc binding protein
UP in Normoxia	**6610**	SMPD2	-1.12105	0.8462152	0.459759	sphingomyelin phosphodiesterase 2, neutral membrane (neutral sphingomyelinase)
UP in Normoxia	**25900**	IFFO1	-1.175752	0.8808474	0.4426529	intermediate filament family orphan 1

UP in Normoxia	**5440**	POLR2K	-1.020741	0.8784467	0.4928633	polymerase (RNA) II (DNA directed) polypeptide K, 7.0kDa
UP in Normoxia	**25821**	MTO1	-1.101188	0.8132939	0.4661326	mitochondrial translation optimization 1 homolog (S. cerevisiae)
UP in Normoxia	**135398**	C6orf141	-2.545225	0.9401953	0.1713212	chromosome 6 open reading frame 141
UP in Normoxia	**10528**	NOP56	-2.910091	0.9605572	0.1330379	NOP56 ribonucleoprotein
UP in Normoxia	**9306**	SOCS6	-1.073199	0.8644988	0.475264	suppressor of cytokine signaling 6
UP in Normoxia	**22796**	COG2	-1.521108	0.8756069	0.3484183	component of oligomeric golgi complex 2
UP in Normoxia	**10269**	ZMPSTE 24	-1.344548	0.8945355	0.3937772	zinc metallopeptidase STE24
UP in Normoxia	**55004**	LAMTO R1	-1.014044	0.8769148	0.4951563	late endosomal/lysosomal adaptor, MAPK and MTOR activator 1
UP in Normoxia	**54487**	DGCR8	-1.234839	0.8723551	0.42489	DiGeorge syndrome critical region gene 8
UP in Normoxia	**196051**	PPAPDC 1A	-1.568633	0.8923856	0.3371276	phosphatidic acid phosphatase type 2 domain containing 1A
UP in Normoxia	**55159**	RFWD3	-1.701163	0.9138941	0.3075382	ring finger and WD repeat domain 3
UP in Normoxia	**11130**	ZWINT	-2.828467	0.9622861	0.1407818	ZW10 interactor, kinetochore protein
UP in Normoxia	**145508**	CEP128	-2.132931	0.8949386	0.2279942	centrosomal protein 128kDa
UP in Normoxia	**81887**	LAS1L	-1.385356	0.8923587	0.3827951	LAS1-like (S. cerevisiae)
UP in Normoxia	**64927**	TTC23	-1.175002	0.8542059	0.442883	tetratricopeptide repeat domain 23
UP in Normoxia	**84524**	ZC3H8	-1.207314	0.858461	0.4330743	zinc finger CCCH-type containing 8
UP in Normoxia	**9159**	PCSK7	-1.511899	0.8838036	0.3506494	proprotein convertase subtilisin/kexin type 7
UP in Normoxia	**54556**	ING3	-1.139438	0.8034668	0.4539363	inhibitor of growth family, member 3
UP in Normoxia	**6857**	SYT1	-1.681658	0.8013885	0.3117241	synaptotagmin I
UP in Normoxia	**1875**	E2F5	-1.524753	0.860808	0.347539	E2F transcription factor 5, p130-binding
UP in Normoxia	**100131897**	FAM196	-1.12858	0.8771208	0.4573657	family with sequence similarity 196,
UP in Normoxia	**100820829**	MYZAP	-1.430202	0.8381528	0.3710788	myocardial zonula adherens protein
UP in Normoxia	**728712**	SPANXA	-1.37523	0.8925647	0.3854911	SPANX family, member A2
UP in Normoxia	**55161**	TMEM33	-1.398159	0.887154	0.3794131	transmembrane protein 33
UP in Normoxia	**5142**	PDE4B	-2.896549	0.9516169	0.1342926	phosphodiesterase 4B, cAMP-specific
UP in Normoxia	**5985**	RFC5	-1.953997	0.9201917	0.2581002	replication factor C (activator 1) 5, 36.5kDa
UP in Normoxia	**26061**	HACL1	-1.537833	0.8949655	0.3444023	2-hydroxyacyl-CoA lyase 1
UP in Normoxia	**10157**	AASS	-1.340143	0.8131237	0.3949814	aminoadipate-semialdehyde synthase
UP in Normoxia	**23384**	SPECC1 L	-1.107823	0.8737078	0.4639936	sperm antigen with calponin homology and coiled-coil domains 1-like
UP in Normoxia	**1659**	DHX8	-1.176065	0.8801845	0.4425569	DEAH (Asp-Glu-Ala-His) box polypeptide
UP in Normoxia	**805**	CALM2	-1.026615	0.8818955	0.4908606	calmodulin 2 (phosphorylase kinase, delta)
UP in Normoxia	**5707**	PSMD1	-1.634255	0.9180328	0.3221366	proteasome (prosome, macropain) 26S subunit, non-ATPase, 1
UP in Normoxia	**79971**	WLS	-1.159678	0.8874407	0.4476125	wntless homolog (Drosophila)
UP in Normoxia	**54842**	MFSD6	-1.35601	0.8657888	0.3906611	major facilitator superfamily domain
UP in Normoxia	**5955**	RCN2	-1.013137	0.8778106	0.4954679	reticulocalbin 2, EF-hand calcium binding domain
UP in Normoxia	**6119**	RPA3	-1.129405	0.8784825	0.4571042	replication protein A3, 14kDa
UP in Normoxia	**8089**	YEATS4	-1.480799	0.8850757	0.3582904	YEATS domain containing 4
UP in Normoxia	**51067**	YARS2	-1.016422	0.8604049	0.4943407	tyrosyl-tRNA synthetase 2, mitochondrial
UP in Normoxia	**26521**	TIMM8B	-1.320252	0.8639703	0.4004651	translocase of inner mitochondrial membrane 8 homolog B (yeast)
UP in Normoxia	**79879**	CCDC13	-3.04501	0.9412882	0.1211604	coiled-coil domain containing 134
UP in Normoxia	**79039**	DDX54	-1.540586	0.8981457	0.3437459	DEAD (Asp-Glu-Ala-Asp) box polypeptide
UP in Normoxia	**81573**	ANKRD1 3C	-1.124716	0.8609245	0.4585921	ankyrin repeat domain 13C
UP in Normoxia	**8883**	NAE1	-1.089952	0.8811699	0.4697769	NEDD8 activating enzyme E1 subunit 1
UP in Normoxia	**100526737**	RBM14-RBM4	-1.588987	0.8891875	0.3324048	RBM14-RBM4 readthrough
UP in Normoxia	**201299**	RDM1	-1.712527	0.8230852	0.3051251	RAD52 motif 1
UP in Normoxia	**23335**	WDR7	-1.185372	0.8231837	0.4397112	WD repeat domain 7

UP in Normoxia	**51021**	MRPS16	-1.360508	0.895709	0.3894451	mitochondrial ribosomal protein S16
UP in Normoxia	**22906**	TRAK1	-1.027301	0.8563379	0.490627	trafficking protein, kinesin binding 1
UP in Normoxia	**23450**	SF3B3	-1.123644	0.8804085	0.4589333	splicing factor 3b, subunit 3, 130kDa
UP in Normoxia	**10055**	SAE1	-1.86342	0.9227358	0.274824	SUMO1 activating enzyme subunit 1
UP in Normoxia	**6382**	SDC1	-1.636652	0.9182567	0.321602	syndecan 1
UP in Normoxia	**11221**	DUSP10	-1.032338	0.8571889	0.488917	dual specificity phosphatase 10
UP in Normoxia	**7639**	ZNF85	-2.256833	0.8251814	0.2092308	zinc finger protein 85
UP in Normoxia	**90861**	HN1L	-1.085503	0.8704381	0.4712279	hematological and neurological expressed 1-
UP in Normoxia	**79140**	CCDC28	-2.790991	0.9322136	0.1444867	coiled-coil domain containing 28B
UP in Normoxia	**64172**	OSGEPL	-1.319542	0.8235958	0.4006623	O-sialoglycoprotein endopeptidase-like 1
UP in Normoxia	**8216**	LZTR1	-1.056377	0.8657709	0.480838	leucine-zipper-like transcription regulator 1
UP in Normoxia	**79612**	NAA16	-1.601728	0.895073	0.3294821	N(alpha)-acetyltransferase 16, NatA auxiliary subunit
UP in Normoxia	**23283**	CSTF2T	-1.178803	0.8762071	0.4417178	cleavage stimulation factor, 3' pre-RNA, subunit 2, 64kDa, tau variant
UP in Normoxia	**29127**	RACGAP	-1.695901	0.9176834	0.3086619	Rac GTPase activating protein 1
UP in Normoxia	**590**	BCHE	-1.372936	0.8555048	0.3861048	butyrylcholinesterase
UP in Normoxia	**442582**	STAG3L	-1.024984	0.8638986	0.4914158	stromal antigen 3-like 2
UP in Normoxia	**23057**	NMNAT	-1.191968	0.8708501	0.4377054	nicotinamide nucleotide adenylyltransferase
UP in Normoxia	**11151**	CORO1A	-2.46048	0.8483562	0.181686	coronin, actin binding protein, 1A
UP in Normoxia	**388796**	LOC388796	-1.337893	0.8874675	0.395598	uncharacterized LOC388796
UP in Normoxia	**9277**	WDR46	-1.632447	0.9120487	0.3225407	WD repeat domain 46
UP in Normoxia	**1039**	CDR2	-1.518883	0.8917316	0.3489559	cerebellar degeneration-related protein 2,
UP in Normoxia	**100507246**	SNHG16	-1.117933	0.8789035	0.4607536	small nucleolar RNA host gene 16 (non-protein coding)
UP in Normoxia	**3320**	HSP90AA1	-1.370016	0.8985577	0.386887	heat shock protein 90kDa alpha (cytosolic), class A member 1
UP in Normoxia	**6643**	SNX2	-1.139905	0.8832661	0.4537894	sorting nexin 2
UP in Normoxia	**1786**	DNMT1	-2.149359	0.9352235	0.2254127	DNA (cytosine-5-)-methyltransferase 1
UP in Normoxia	**80228**	ORAI2	-1.192577	0.8677685	0.4375206	ORAI calcium release-activated calcium modulator 2
UP in Normoxia	**5257**	PHKB	-1.059148	0.8611753	0.4799154	phosphorylase kinase, beta
UP in Normoxia	**55584**	CHRNA9	-1.102275	0.8411896	0.4657814	cholinergic receptor, nicotinic, alpha 9 (neuronal)
UP in Normoxia	**55589**	BMP2K	-1.141232	0.8553525	0.4533722	BMP2 inducible kinase
UP in Normoxia	**100874222**	RNF219-AS1	-1.222083	0.8363791	0.4286632	RNF219 antisense RNA 1
UP in Normoxia	**6683**	SPAST	-1.251406	0.8563289	0.4200385	spastin
UP in Normoxia	**55329**	MNS1	-1.514953	0.8592403	0.3499079	meiosis-specific nuclear structural 1
UP in Normoxia	**29960**	FTSJ2	-1.329116	0.8905133	0.398012	FtsJ RNA methyltransferase homolog 2 (E.
UP in Normoxia	**10576**	CCT2	-1.66945	0.9194751	0.3143732	chaperonin containing TCP1, subunit 2
UP in Normoxia	**57129**	MRPL47	-1.248346	0.8892144	0.4209304	mitochondrial ribosomal protein L47
UP in Normoxia	**1534**	CYB561	-2.104086	0.9253158	0.2325986	cytochrome b561
UP in Normoxia	**51012**	SLMO2	-1.200332	0.8852638	0.435175	slowmo homolog 2 (Drosophila)
UP in Normoxia	**6697**	SPR	-1.51028	0.8975096	0.3510432	sepiapterin reductase (7,8-dihydrobiopterin:NADP+ oxidoreductase)
UP in Normoxia	**6183**	MRPS12	-1.384772	0.8962465	0.38295	mitochondrial ribosomal protein S12
UP in Normoxia	**518**	ATP5G3	-1.225071	0.8906835	0.4277764	**ATP synthase, H+ transporting, mitochondrial Fo complex, subunit C3 (subunit 9)**
UP in Normoxia	**51204**	TACO1	-1.378234	0.8909433	0.3846895	translational activator of mitochondrially encoded cytochrome c oxidase I
UP in Normoxia	**7268**	TTC4	-1.208878	0.8859536	0.4326048	tetratricopeptide repeat domain 4
UP in Normoxia	**5700**	PSMC1	-1.063516	0.8829347	0.4784646	proteasome (prosome, macropain) 26S subunit, ATPase, 1
UP in Normoxia	**1745**	DLX1	-2.13831	0.9194571	0.2271457	distal-less homeobox 1

UP in Normoxia	**27034**	ACAD8	-1.066261	0.84196	0.4775549	acyl-CoA dehydrogenase family, member 8
UP in Normoxia	**3702**	ITK	-2.972888	0.8773179	0.1273713	IL2-inducible T-cell kinase
UP in Normoxia	**29988**	SLC2A8	-1.154975	0.8590701	0.4490741	solute carrier family 2 (facilitated glucose transporter), member 8
UP in Normoxia	**79710**	MORC4	-1.601555	0.9025262	0.3295215	MORC family CW-type zinc finger 4
UP in Normoxia	**134**	ADORA1	-3.356338	0.9105617	0.0976431	adenosine A1 receptor
UP in Normoxia	**126133**	ALDH16 A1	-1.547675	0.8370868	0.3420608	aldehyde dehydrogenase 16 family, member A1
UP in Normoxia	**10714**	POLD3	-1.602373	0.8823882	0.3293348	polymerase (DNA-directed), delta 3, accessory subunit
UP in Normoxia	**29028**	ATAD2	-2.566178	0.9437158	0.1688509	ATPase family, AAA domain containing 2
UP in Normoxia	**10296**	MAEA	-1.000208	0.8769506	0.499928	macrophage erythroblast attacher
UP in Normoxia	**51095**	TRNT1	-1.61957	0.9017737	0.3254324	tRNA nucleotidyl transferase, CCA-adding,
UP in Normoxia	**56992**	KIF15	-2.104405	0.9133835	0.2325472	kinesin family member 15
UP in Normoxia	**834**	CASP1	-2.61891	0.840249	0.1627907	caspase 1, apoptosis-related cysteine
UP in Normoxia	**92935**	MARS2	-2.168237	0.9067903	0.2224824	methionyl-tRNA synthetase 2, mitochondrial
UP in Normoxia	**10131**	TRAP1	-1.060269	0.8760996	0.4795426	TNF receptor-associated protein 1
UP in Normoxia	**26094**	DCAF4	-1.335637	0.8610857	0.396217	DDB1 and CUL4 associated factor 4
UP in Normoxia	**5610**	EIF2AK2	-1.708683	0.9027233	0.3059392	eukaryotic translation initiation factor 2-alpha kinase 2
UP in Normoxia	**4001**	LMNB1	-1.844676	0.90877	0.2784179	lamin B1
UP in Normoxia	**3241**	HPCAL1	-1.181058	0.8895458	0.4410279	hippocalcin-like 1
UP in Normoxia	**22891**	ZNF365	-1.376891	0.8568037	0.3850478	zinc finger protein 365
UP in Normoxia	**2237**	FEN1	-2.961558	0.9612559	0.1283755	flap structure-specific endonuclease 1
UP in Normoxia	**5378**	PMS1	-1.02272	0.8272328	0.4921875	PMS1 postmeiotic segregation increased 1 (S. cerevisiae)
UP in Normoxia	**91419**	XRCC6B P1	-1.132046	0.8162322	0.4562682	XRCC6 binding protein 1
UP in Normoxia	**64946**	CENPH	-1.477519	0.8969363	0.3591057	centromere protein H
UP in Normoxia	**200894**	ARL13B	-1.376991	0.8605034	0.3850211	ADP-ribosylation factor-like 13B
UP in Normoxia	**253512**	SLC25A3	-1.0732	0.8507032	0.4752636	solute carrier family 25, member 30
UP in Normoxia	**63926**	ANKEF1	-2.706269	0.8693899	0.1532258	ankyrin repeat and EF-hand domain
UP in Normoxia	**399665**	FAM102	-1.740326	0.9189734	0.2993021	family with sequence similarity 102,
UP in Normoxia	**64979**	MRPL36	-1.019547	0.8781152	0.4932711	mitochondrial ribosomal protein L36
UP in Normoxia	**51002**	TPRKB	-1.35388	0.8930485	0.3912383	TP53RK binding protein
UP in Normoxia	**2258**	FGF13	-1.007464	0.8319896	0.49742	fibroblast growth factor 13
UP in Normoxia	**6703**	SPRR2D	-1.479869	0.8834005	0.3585214	small proline-rich protein 2D
UP in Normoxia	**26155**	NOC2L	-1.104746	0.8842068	0.4649843	nucleolar complex associated 2 homolog (S. cerevisiae)
UP in Normoxia	**1161**	ERCC8	-1.21272	0.8667831	0.4314544	excision repair cross-complementing rodent repair deficiency, complementation group 8
UP in Normoxia	**23466**	CBX6	-1.791385	0.910499	0.2888946	chromobox homolog 6
UP in Normoxia	**990**	CDC6	-2.905521	0.9572516	0.1334599	cell division cycle 6
UP in Normoxia	**80746**	TSEN2	-2.106586	0.8924751	0.2321958	tRNA splicing endonuclease 2 homolog (S. cerevisiae)
UP in Normoxia	**1063**	CENPF	-1.295635	0.8872973	0.4073567	centromere protein F, 350/400kDa
UP in Normoxia	**55316**	RSAD1	-1.049556	0.8701335	0.4831169	radical S-adenosyl methionine domain containing 1
UP in Normoxia	**10478**	SLC25A1 7	-1.132366	0.8733495	0.4561671	solute carrier family 25 (mitochondrial carrier; peroxisomal membrane protein, 34kDa), member 17
UP in Normoxia	**54816**	ZNF280	-1.375566	0.8386187	0.3854015	zinc finger protein 280D
UP in Normoxia	**29914**	UBIAD1	-1.519706	0.8517334	0.3487569	UbiA prenyltransferase domain containing 1
UP in Normoxia	**1662**	DDX10	-2.068593	0.9058766	0.2383918	DEAD (Asp-Glu-Ala-Asp) box polypeptide
UP in Normoxia	**9156**	EXO1	-4.005029	0.9740482	0.0622825	exonuclease 1
UP in Normoxia	**54962**	TIPIN	-1.473142	0.8904237	0.360197	TIMELESS interacting protein

UP in Normoxia	**56996**	SLC12A9	-1.165626	0.8652513	0.4457708	solute carrier family 12 (potassium/chloride transporters), member 9
UP in Normoxia	**51603**	METTL1	-1.090269	0.8632715	0.4696739	methyltransferase like 13
UP in Normoxia	**2650**	GCNT1	-1.178544	0.8273493	0.4417971	glucosaminyl (N-acetyl) transferase 1, core
UP in Normoxia	**159371**	SLC35G1	-1.94401	0.8469497	0.259893	solute carrier family 35, member G1
UP in Normoxia	**7726**	TRIM26	-1.166165	0.8591329	0.4456042	tripartite motif containing 26
UP in Normoxia	**53834**	FGFRL1	-1.069242	0.8626892	0.4765694	fibroblast growth factor receptor-like 1
UP in Normoxia	**54617**	INO80	-1.031364	0.8551913	0.4892473	INO80 homolog (S. cerevisiae)
UP in Normoxia	**54677**	CROT	-1.822057	0.911144	0.2828175	carnitine O-octanoyltransferase
UP in Normoxia	**23175**	LPIN1	-1.092173	0.8652334	0.4690544	lipin 1
UP in Normoxia	**6240**	RRM1	-1.889231	0.9234883	0.2699508	ribonucleotide reductase M1
UP in Normoxia	**153769**	SH3RF2	-3.276049	0.9548956	0.1032312	SH3 domain containing ring finger 2
UP in Normoxia	**4436**	MSH2	-1.888372	0.916698	0.2701118	mutS homolog 2, colon cancer, nonpolyposis type 1 (E. coli)
UP in Normoxia	**1462**	VCAN	-1.731926	0.9176565	0.3010497	versican
UP in Normoxia	**79000**	AUNIP	-2.440886	0.8418078	0.1841705	aurora kinase A and ninein interacting
UP in Normoxia	**890**	CCNA2	-1.736641	0.911556	0.3000676	cyclin A2
UP in Normoxia	**50614**	GALNT9	-1.272527	0.8911941	0.4139342	UDP-N-acetyl-alpha-D-galactosamine:polypeptide N-acetylgalactosaminyltransferase 9 (GalNAc-T9)
UP in Normoxia	**6891**	TAP2	-2.806927	0.9515184	0.1428995	transporter 2, ATP-binding cassette, sub-family B (MDR/TAP)
UP in Normoxia	**84437**	MSANT D4	-1.000704	0.8348204	0.4997562	Myb/SANT-like DNA-binding domain containing 4 with coiled-coils
UP in Normoxia	**83451**	ABHD11	-2.103266	0.9328944	0.2327308	abhydrolase domain containing 11
UP in Normoxia	**100130093**	LOC1001 30093	-1.772278	0.8162412	0.2927461	uncharacterized LOC100130093
UP in Normoxia	**57513**	CASKIN	-1.064968	0.8208546	0.4779834	CASK interacting protein 2
UP in Normoxia	**144455**	E2F7	-2.005622	0.9330108	0.2490277	E2F transcription factor 7
UP in Normoxia	**55863**	TMEM12 6B	-1.435357	0.8923587	0.3697552	transmembrane protein 126B
UP in Normoxia	**3954**	LETM1	-1.767107	0.9144316	0.2937972	leucine zipper-EF-hand containing transmembrane protein 1
UP in Normoxia	**1186**	CLCN7	-1.078251	0.8725432	0.4736028	chloride channel, voltage-sensitive 7
UP in Normoxia	**51660**	MPC1	-1.232324	0.8756427	0.4256312	mitochondrial pyruvate carrier 1
UP in Normoxia	**908**	CCT6A	-1.78865	0.9220281	0.2894427	chaperonin containing TCP1, subunit 6A
UP in Normoxia	**55646**	LYAR	-1.531205	0.8975455	0.3459882	Ly1 antibody reactive
UP in Normoxia	**7913**	DEK	-1.028367	0.8765654	0.4902648	DEK oncogene
UP in Normoxia	**3376**	IARS	-1.42489	0.8998029	0.3724476	isoleucyl-tRNA synthetase
UP in Normoxia	**1431**	CS	-1.318441	0.8962107	0.4009679	citrate synthase
UP in Normoxia	**4967**	OGDH	-1.145808	0.8846636	0.4519364	oxoglutarate (alpha-ketoglutarate) dehydrogenase (lipoamide)
UP in Normoxia	**6722**	SRF	-1.070337	0.8551913	0.4762078	serum response factor (c-fos serum response element-binding transcription factor)
UP in Normoxia	**5074**	PAWR	-1.650147	0.8041387	0.3186078	PRKC, apoptosis, WT1, regulator
UP in Normoxia	**2048**	EPHB2	-2.344203	0.942399	0.1969357	EPH receptor B2
UP in Normoxia	**5717**	PSMD11	-1.622376	0.9165368	0.3248	proteasome (prosome, macropain) 26S subunit, non-ATPase, 11
UP in Normoxia	**57016**	AKR1B1 0	-3.22015	0.8717549	0.1073095	aldo-keto reductase family 1, member B10 (aldose reductase)
UP in Normoxia	**23264**	ZC3H7B	-1.160773	0.8824241	0.4472727	zinc finger CCCH-type containing 7B
UP in Normoxia	**51251**	NT5C3A	-1.189825	0.8713876	0.4383562	5'-nucleotidase, cytosolic IIIA
UP in Normoxia	**23279**	NUP160	-1.231801	0.8758667	0.4257855	nucleoporin 160kDa
UP in Normoxia	**387978**	C14orf23	-2.26453	0.886079	0.2081174	chromosome 14 open reading frame 23
UP in Normoxia	**3312**	HSPA8	-1.62428	0.9188211	0.3243717	heat shock 70kDa protein 8
UP in Normoxia	**60528**	ELAC2	-2.146778	0.9351787	0.2258164	elaC homolog 2 (E. coli)

UP in Normoxia	**100303728**	SLC25A5-AS1	-1.421485	0.8741199	0.3733279	SLC25A5 antisense RNA 1
UP in Normoxia	**4638**	MYLK	-1.580736	0.9131058	0.3343112	myosin light chain kinase
UP in Normoxia	**5636**	PRPSAP2	-1.036093	0.8725074	0.4876463	phosphoribosyl pyrophosphate synthetase-associated protein 2
UP in Normoxia	**4751**	NEK2	-1.729953	0.9146914	0.3014618	NIMA-related kinase 2
UP in Normoxia	**26135**	SERBP1	-1.309751	0.8927349	0.4033905	SERPINE1 mRNA binding protein 1
UP in Normoxia	**11321**	GPN1	-1.019284	0.8780077	0.493361	GPN-loop GTPase 1
UP in Normoxia	**7756**	ZNF207	-1.209915	0.8899131	0.4322941	zinc finger protein 207
UP in Normoxia	**55013**	CCDC109B	-1.050136	0.8611216	0.4829225	coiled-coil domain containing 109B
UP in Normoxia	**488**	ATP2A2	-1.230824	0.890782	0.4260741	ATPase, Ca++ transporting, cardiac muscle, slow twitch 2
UP in Normoxia	**1138**	CHRNA5	-2.441471	0.8794948	0.1840959	cholinergic receptor, nicotinic, alpha 5 (neuronal)
UP in Normoxia	**26589**	MRPL46	-1.332618	0.8921258	0.3970469	mitochondrial ribosomal protein L46
UP in Normoxia	**84126**	ATRIP	-1.750139	0.8962107	0.2972731	ATR interacting protein
UP in Normoxia	**10799**	RPP40	-2.247928	0.9067545	0.2105263	ribonuclease P/MRP 40kDa subunit
UP in Normoxia	**4085**	MAD2L1	-1.875903	0.9191257	0.2724563	MAD2 mitotic arrest deficient-like 1 (yeast)
UP in Normoxia	**197320**	ZNF778	-1.374802	0.8540536	0.3856056	zinc finger protein 778
UP in Normoxia	**54625**	PARP14	-1.4692	0.853955	0.3611825	poly (ADP-ribose) polymerase family,
UP in Normoxia	**26262**	TSPAN1	-1.148825	0.8831945	0.4509923	tetraspanin 17
UP in Normoxia	**375295**	LOC375295	-1.382863	0.8872794	0.383457	uncharacterized LOC375295
UP in Normoxia	**79156**	PLEKHF1	-1.141712	0.8389143	0.4532213	pleckstrin homology domain containing, family F (with FYVE domain) member 1
UP in Normoxia	**81930**	KIF18A	-1.886093	0.9050703	0.2705388	kinesin family member 18A
UP in Normoxia	**2970**	GTF2IP1	-1.009764	0.8436621	0.4966273	general transcription factor IIi, pseudogene
UP in Normoxia	**11270**	NRM	-1.60789	0.9004569	0.3280778	nurim (nuclear envelope membrane protein)
UP in Normoxia	**28512**	NKIRAS	-1.013047	0.8525576	0.4954986	NFKB inhibitor interacting Ras-like 1
UP in Normoxia	**8871**	SYNJ2	-1.542949	0.8992386	0.3431833	synaptojanin 2
UP in Normoxia	**8803**	SUCLA2	-1.179831	0.8745499	0.4414033	succinate-CoA ligase, ADP-forming, beta subunit
UP in Normoxia	**84316**	LSMD1	-1.009206	0.8757413	0.4968195	LSM domain containing 1
UP in Normoxia	**100101490**	PSIMCT-1	-1.94301	0.8783212	0.2600733	malignant T cell amplified sequence 1 pseudogene
UP in Normoxia	**9455**	HOMER	-1.315912	0.8833378	0.4016716	homer homolog 2 (Drosophila)
UP in Normoxia	**79075**	DSCC1	-3.727567	0.9626982	0.0754902	defective in sister chromatid cohesion 1 homolog (S. cerevisiae)
UP in Normoxia	**11194**	ABCB8	-1.215971	0.8774165	0.4304833	ATP-binding cassette, sub-family B (MDR/TAP), member 8
UP in Normoxia	**23357**	ANGEL1	-1.624308	0.8867419	0.3243655	angel homolog 1 (Drosophila)
UP in Normoxia	**55342**	STRBP	-1.311359	0.8033324	0.4029412	spermatid perinuclear RNA binding protein
UP in Normoxia	**23234**	DNAJC9	-2.140902	0.936836	0.226738	DnaJ (Hsp40) homolog, subfamily C,
UP in Normoxia	**283989**	TSEN54	-1.054036	0.8529159	0.481619	tRNA splicing endonuclease 54 homolog (S. cerevisiae)
UP in Normoxia	**7444**	VRK2	-1.410267	0.88779	0.376242	vaccinia related kinase 2
UP in Normoxia	**9289**	GPR56	-1.691193	0.9201559	0.3096707	G protein-coupled receptor 56
UP in Normoxia	**79677**	SMC6	-1.207568	0.8744065	0.4329979	structural maintenance of chromosomes 6
UP in Normoxia	**90416**	C15orf57	-1.013773	0.8582102	0.4952494	chromosome 15 open reading frame 57
UP in Normoxia	**79019**	CENPM	-3.437766	0.9705724	0.0922846	centromere protein M
UP in Normoxia	**92856**	IMP4	-1.426388	0.8980919	0.3720611	IMP4, U3 small nucleolar ribonucleoprotein, homolog (yeast)
UP in Normoxia	**818**	CAMK2G	-1.518827	0.8951178	0.3489694	calcium/calmodulin-dependent protein kinase II gamma
UP in Normoxia	**9516**	LITAF	-1.211188	0.8833289	0.4319128	lipopolysaccharide-induced TNF factor
UP in Normoxia	**81831**	NETO2	-1.840915	0.9098629	0.2791447	neuropilin (NRP) and tolloid (TLL)-like 2

UP in Normoxia	**56521**	DNAJC1	-1.69291	0.825047	0.3093023	DnaJ (Hsp40) homolog, subfamily C,
UP in Normoxia	**11080**	DNAJB4	-1.402555	0.864669	0.3782587	DnaJ (Hsp40) homolog, subfamily B,
UP in Normoxia	**10395**	DLC1	-1.288111	0.8817164	0.4094867	deleted in liver cancer 1
UP in Normoxia	**891**	CCNB1	-2.061903	0.9364597	0.2394999	cyclin B1
UP in Normoxia	**22873**	DZIP1	-1.369308	0.8507928	0.3870769	DAZ interacting protein 1
UP in Normoxia	**1738**	DLD	-1.054068	0.8768521	0.4816083	dihydrolipoamide dehydrogenase
UP in Normoxia	**54502**	RBM47	-1.690945	0.8212398	0.3097239	RNA binding motif protein 47
UP in Normoxia	**10769**	PLK2	-2.545409	0.9373556	0.1712993	polo-like kinase 2
UP in Normoxia	**10785**	WDR4	-3.421657	0.963809	0.0933208	WD repeat domain 4
UP in Normoxia	**10808**	HSPH1	-2.39609	0.9476485	0.1899788	heat shock 105kDa/110kDa protein 1
UP in Normoxia	**29102**	DROSH	-1.045505	0.8640419	0.4844754	drosha, ribonuclease type III
UP in Normoxia	**285753**	CEP57L1	-1.114358	0.8517065	0.4618967	centrosomal protein 57kDa-like 1
UP in Normoxia	**8533**	COPS3	-1.370468	0.8958792	0.3867659	COP9 signalosome subunit 3
UP in Normoxia	**2596**	GAP43	-2.928867	0.943895	0.1313177	growth associated protein 43
UP in Normoxia	**9786**	KIAA058	-1.622222	0.8895817	0.3248347	KIAA0586
UP in Normoxia	**355**	FAS	-1.750505	0.9025083	0.2971976	Fas (TNF receptor superfamily, member 6)
UP in Normoxia	**283871**	PGP	-1.221957	0.8578787	0.4287008	phosphoglycolate phosphatase
UP in Normoxia	**55559**	HAUS7	-2.092009	0.9198423	0.2345538	HAUS augmin-like complex, subunit 7
UP in Normoxia	**57192**	MCOLN	-1.044794	0.866595	0.4847141	mucolipin 1
UP in Normoxia	**57691**	KIAA158	-1.019629	0.8112783	0.4932432	KIAA1586
UP in Normoxia	**22984**	PDCD11	-1.872535	0.9165726	0.2730931	programmed cell death 11
UP in Normoxia	**57645**	POGK	-1.422443	0.8742811	0.37308	pogo transposable element with KRAB
UP in Normoxia	**2068**	ERCC2	-1.059409	0.866174	0.4798285	excision repair cross-complementing rodent repair deficiency, complementation group 2
UP in Normoxia	**25894**	PLEKHG 4	-1.804422	0.8113142	0.2862958	pleckstrin homology domain containing, family G (with RhoGef domain) member 4
UP in Normoxia	**90121**	TSR2	-1.019466	0.8772194	0.4932989	TSR2, 20S rRNA accumulation, homolog (S. cerevisiae)
UP in Normoxia	**11056**	DDX52	-1.125383	0.8579414	0.4583804	DEAD (Asp-Glu-Ala-Asp) box polypeptide
UP in Normoxia	**2177**	FANCD2	-1.782304	0.9037445	0.2907187	Fanconi anemia, complementation group D2
UP in Normoxia	**3835**	KIF22	-1.873951	0.9204157	0.2728252	kinesin family member 22
UP in Normoxia	**2171**	FABP5	-2.347493	0.9404909	0.1964871	fatty acid binding protein 5 (psoriasis-associated)
UP in Normoxia	**10762**	NUP50	-1.48439	0.8945176	0.3573996	nucleoporin 50kDa
UP in Normoxia	**8942**	KYNU	-1.840877	0.9236316	0.2791521	kynureninase
UP in Normoxia	**27301**	APEX2	-1.089591	0.8762071	0.4698945	APEX nuclease (apurinic/apyrimidinic endonuclease) 2
UP in Normoxia	**7486**	WRN	-1.402774	0.8545104	0.3782013	Werner syndrome, RecQ helicase-like
UP in Normoxia	**58517**	RBM25	-1.039395	0.8748455	0.4865313	RNA binding motif protein 25
UP in Normoxia	**23122**	CLASP2	-1.122248	0.8572516	0.4593774	cytoplasmic linker associated protein 2
UP in Normoxia	**729082**	OIP5-	-1.688745	0.9046851	0.3101966	OIP5 antisense RNA 1
UP in Normoxia	**10165**	SLC25A1 3	-1.411938	0.8872257	0.3758065	solute carrier family 25 (aspartate/glutamate carrier), member 13
UP in Normoxia	**54918**	CMTM6	-1.174486	0.8851205	0.4430416	CKLF-like MARVEL transmembrane domain containing 6
UP in Normoxia	**29775**	CARD10	-1.813807	0.9133118	0.2844394	caspase recruitment domain family, member
UP in Normoxia	**7074**	TIAM1	-2.110424	0.8541342	0.2315789	T-cell lymphoma invasion and metastasis 1
UP in Normoxia	**122416**	ANKRD9	-1.075779	0.8713249	0.4744148	ankyrin repeat domain 9
UP in Normoxia	**26995**	TRUB2	-1.178178	0.8844845	0.4419092	TruB pseudouridine (psi) synthase homolog 2 (E. coli)
UP in Normoxia	**7168**	TPM1	-1.038948	0.8814745	0.4866823	tropomyosin 1 (alpha)
UP in Normoxia	**9343**	EFTUD2	-1.013564	0.8778644	0.4953212	elongation factor Tu GTP binding domain containing 2
UP in Normoxia	**55222**	LRRC20	-2.33174	0.9390039	0.1986444	leucine rich repeat containing 20
UP in Normoxia	**11309**	SLCO2B 1	-3.713188	0.8939712	0.0762463	solute carrier organic anion transporter family, member 2B1
UP in Normoxia	**3667**	IRS1	-1.236917	0.8817612	0.4242784	insulin receptor substrate 1

UP in Normoxia	**2870**	GRK6	-1.555663	0.8913912	0.3401722	G protein-coupled receptor kinase 6
UP in Normoxia	**23089**	PEG10	-1.921198	0.9185076	0.2640352	paternally expressed 10
UP in Normoxia	**22907**	DHX30	-1.391786	0.8948849	0.3810928	DEAH (Asp-Glu-Ala-His) box polypeptide
UP in Normoxia	**9911**	TMCC2	-2.35975	0.8390218	0.194825	transmembrane and coiled-coil domain
UP in Normoxia	**55751**	TMEM184C	-1.104737	0.8508824	0.4649872	transmembrane protein 184C
UP in Normoxia	**55164**	SHQ1	-1.105743	0.8613724	0.4646631	SHQ1, H/ACA ribonucleoprotein assembly factor
UP in Normoxia	**389362**	PSMG4	-1.098942	0.8621965	0.4668588	proteasome (prosome, macropain) assembly chaperone 4
UP in Normoxia	**117145**	THEM4	-1.2755	0.8466631	0.4130819	thioesterase superfamily member 4
UP in Normoxia	**55120**	FANCL	-1.501349	0.8932008	0.3532229	Fanconi anemia, complementation group L
UP in Normoxia	**5701**	PSMC2	-1.381612	0.8967213	0.3837897	proteasome (prosome, macropain) 26S subunit, ATPase, 2
UP in Normoxia	**23082**	PPRC1	-2.035454	0.9292395	0.2439311	peroxisome proliferator-activated receptor gamma, coactivator-related 1
UP in Normoxia	**136319**	MTPN	-8.575539	0.973018	0.0026212	myotrophin
UP in Normoxia	**5347**	PLK1	-1.847834	0.9215354	0.2778092	polo-like kinase 1
UP in Normoxia	**84309**	NUDT16L1	-1.271348	0.8593478	0.4142725	nudix (nucleoside diphosphate linked moiety X)-type motif 16-like 1
UP in Normoxia	**10195**	ALG3	-1.419758	0.8965511	0.373775	ALG3, alpha-1,3- mannosyltransferase
UP in Normoxia	**55149**	MTPAP	-1.113	0.8057601	0.4623315	mitochondrial poly(A) polymerase
UP in Normoxia	**166647**	GPR125	-2.511872	0.9408224	0.175328	G protein-coupled receptor 125
UP in Normoxia	**84300**	MNF1	-1.435672	0.8701066	0.3696748	mitochondrial nucleoid factor 1
UP in Normoxia	**54978**	SLC35F6	-1.542568	0.8934695	0.343274	solute carrier family 35, member F6
UP in Normoxia	**9738**	CCP110	-1.069869	0.8553794	0.4763623	centriolar coiled coil protein 110kDa
UP in Normoxia	**30836**	DNTTIP2	-1.403743	0.8906656	0.3779472	deoxynucleotidyltransferase, terminal, interacting protein 2
UP in Normoxia	**2230**	FDX1	-1.412468	0.847111	0.3756684	ferredoxin 1
UP in Normoxia	**11171**	STRAP	-1.256331	0.8931739	0.4186072	serine/threonine kinase receptor associated protein
UP in Normoxia	**51194**	IPO11	-1.138561	0.8722834	0.4542125	importin 11
UP in Normoxia	**51278**	IER5	-1.223813	0.8776763	0.4281496	immediate early response 5
UP in Normoxia	**84681**	HINT2	-1.178404	0.8872615	0.4418399	histidine triad nucleotide binding protein 2
UP in Normoxia	**64421**	DCLRE1	-1.172204	0.840267	0.4437428	DNA cross-link repair 1C
UP in Normoxia	**7697**	ZNF138	-1.171987	0.8679746	0.4438095	zinc finger protein 138
UP in Normoxia	**29081**	METTL5	-1.036187	0.8775508	0.4876146	methyltransferase like 5
UP in Normoxia	**1879**	EBF1	-1.72995	0.831058	0.3014623	early B-cell factor 1
UP in Normoxia	**5519**	PPP2R1B	-1.558107	0.8848249	0.3395964	protein phosphatase 2, regulatory subunit A, beta
UP in Normoxia	**1841**	DTYMK	-1.730987	0.9197438	0.3012459	deoxythymidylate kinase (thymidylate
UP in Normoxia	**55578**	SUPT20	-1.050954	0.8727761	0.4826488	suppressor of Ty 20 homolog (S. cerevisiae)
UP in Normoxia	**7298**	TYMS	-1.034353	0.8797456	0.4882347	thymidylate synthetase
UP in Normoxia	**8165**	AKAP1	-1.344973	0.869166	0.3936614	A kinase (PRKA) anchor protein 1
UP in Normoxia	**4706**	NDUFAB1	-1.010567	0.8790379	0.496351	NADH dehydrogenase (ubiquinone) 1, alpha/beta subcomplex, 1, 8kDa
UP in Normoxia	**80854**	SETD7	-1.319499	0.8849861	0.4006742	SET domain containing (lysine methyltransferase) 7
UP in Normoxia	**3070**	HELLS	-3.513696	0.9586939	0.0875532	helicase, lymphoid-specific
UP in Normoxia	**283742**	FAM98B	-1.252103	0.8852997	0.4198357	family with sequence similarity 98, member
UP in Normoxia	**29954**	POMT2	-1.293614	0.8569471	0.4079277	protein-O-mannosyltransferase 2
UP in Normoxia	**81792**	ADAMTS12	-1.65819	0.8898683	0.3168365	ADAM metallopeptidase with thrombospondin type 1 motif, 12
UP in Normoxia	**5834**	PYGB	-1.322297	0.8887844	0.3998978	phosphorylase, glycogen; brain
UP in Normoxia	**345778**	MTX3	-1.027079	0.8572875	0.4907028	metaxin 3
UP in Normoxia	**84260**	TCHP	-1.780271	0.9132043	0.2911287	trichoplein, keratin filament binding
UP in Normoxia	**100133941**	CD24	-2.558979	0.9425423	0.1696956	CD24 molecule

UP in Normoxia	**27297**	CRCP	-1.082553	0.8756965	0.4721924	CGRP receptor component
UP in Normoxia	**7371**	UCK2	-1.47978	0.8768252	0.3585434	uridine-cytidine kinase 2
UP in Normoxia	**80775**	TMEM17	-2.30754	0.8945803	0.2020046	transmembrane protein 177
UP in Normoxia	**7414**	VCL	-1.196168	0.8890173	0.436433	vinculin
UP in Normoxia	**84916**	CIRH1A	-1.244738	0.8896712	0.4219845	cirrhosis, autosomal recessive 1A (cirhin)
UP in Normoxia	**27000**	DNAJC2	-1.398952	0.8925378	0.3792045	DnaJ (Hsp40) homolog, subfamily C,
UP in Normoxia	**65993**	MRPS34	-1.164075	0.8851384	0.4462504	mitochondrial ribosomal protein S34
UP in Normoxia	**60481**	ELOVL5	-1.633826	0.9149333	0.3222325	ELOVL fatty acid elongase 5
UP in Normoxia	**57539**	WDR35	-1.618619	0.8896712	0.3256471	WD repeat domain 35
UP in Normoxia	**114804**	RNF157	-1.364846	0.8421034	0.3882759	ring finger protein 157
UP in Normoxia	**8805**	TRIM24	-1.035877	0.8541342	0.4877193	tripartite motif containing 24
UP in Normoxia	**80764**	THAP7	-1.303732	0.8872525	0.4050769	THAP domain containing 7
UP in Normoxia	**7283**	TUBG1	-1.63696	0.9184359	0.3215332	tubulin, gamma 1
UP in Normoxia	**4913**	NTHL1	-2.237703	0.9113142	0.2120236	nth endonuclease III-like 1 (E. coli)
UP in Normoxia	**9414**	TJP2	-1.946621	0.9203888	0.2594232	tight junction protein 2
UP in Normoxia	**4174**	MCM5	-3.021668	0.966174	0.1231367	minichromosome maintenance complex component 5
UP in Normoxia	**2052**	EPHX1	-1.514687	0.8970348	0.3499724	epoxide hydrolase 1, microsomal
UP in Normoxia	**114789**	SLC25A2 5	-1.855902	0.9094777	0.2762599	solute carrier family 25 (mitochondrial carrier; phosphate carrier), member 25
UP in Normoxia	**5357**	PLS1	-2.484628	0.8548688	0.1786704	plastin 1
UP in Normoxia	**5888**	RAD51	-2.377408	0.9216429	0.1924549	RAD51 homolog (S. cerevisiae)
UP in Normoxia	**54906**	FAM208	-1.48385	0.8850309	0.3575334	family with sequence similarity 208,
UP in Normoxia	**10556**	RPP30	-1.08908	0.8734211	0.470061	ribonuclease P/MRP 30kDa subunit
UP in Normoxia	**2679**	GGT3P	-2.14208	0.8511511	0.226553	gamma-glutamyltransferase 3 pseudogene
UP in Normoxia	**26985**	AP3M1	-1.08801	0.8759025	0.47041	adaptor-related protein complex 3, mu 1
UP in Normoxia	**51659**	GINS2	-3.219085	0.9654484	0.1073888	GINS complex subunit 2 (Psf2 homolog)
UP in Normoxia	**65095**	KRI1	-1.508912	0.894643	0.3513761	KRI1 homolog (S. cerevisiae)
UP in Normoxia	**84342**	COG8	-1.031422	0.8631909	0.4892275	component of oligomeric golgi complex 8
UP in Normoxia	**607**	BCL9	-1.348653	0.8738063	0.3926585	B-cell CLL/lymphoma 9
UP in Normoxia	**23313**	KIAA093	-1.381654	0.8852459	0.3837784	KIAA0930
UP in Normoxia	**643723**	LOC6437 23	-2.732304	0.8465556	0.1504854	uncharacterized LOC643723
UP in Normoxia	**2187**	FANCB	-2.996238	0.8856221	0.1253264	Fanconi anemia, complementation group B
UP in Normoxia	**6723**	SRM	-1.629157	0.9180686	0.3232771	spermidine synthase
UP in Normoxia	**154807**	VKORC1 L1	-1.411202	0.888444	0.3759983	vitamin K epoxide reductase complex, subunit 1-like 1
UP in Normoxia	**286826**	LIN9	-1.679359	0.8522082	0.3122212	lin-9 homolog (C. elegans)
UP in Normoxia	**55199**	FAM86C	-1.993976	0.8907552	0.251046	family with sequence similarity 86, member
UP in Normoxia	**85377**	MICALL	-1.137192	0.8813133	0.4546438	MICAL-like 1
UP in Normoxia	**91875**	TTC5	-1.599282	0.8548598	0.3300412	tetratricopeptide repeat domain 5
UP in Normoxia	**3417**	IDH1	-1.163515	0.8862492	0.4464236	isocitrate dehydrogenase 1 (NADP+),
UP in Normoxia	**4175**	MCM6	-2.689532	0.9522171	0.1550137	minichromosome maintenance complex component 6
UP in Normoxia	**55502**	HES6	-2.249825	0.9187136	0.2102497	hairy and enhancer of split 6 (Drosophila)
UP in Normoxia	**79622**	SNRNP2 5	-1.318518	0.8848965	0.4009466	small nuclear ribonucleoprotein 25kDa (U11/U12)
UP in Normoxia	**147463**	ANKRD2	-1.692447	0.9022843	0.3094017	ankyrin repeat domain 29
UP in Normoxia	**63899**	NSUN3	-1.139588	0.8081878	0.4538893	NOP2/Sun domain family, member 3
UP in Normoxia	**4053**	LTBP2	-1.437041	0.8810624	0.3693239	latent transforming growth factor beta binding protein 2
UP in Normoxia	**51167**	CYB5R4	-1.024926	0.8645884	0.4914355	cytochrome b5 reductase 4
UP in Normoxia	**10121**	ACTR1A	-1.182144	0.8887217	0.4406962	ARP1 actin-related protein 1 homolog A, centractin alpha (yeast)
UP in Normoxia	**128989**	TANGO2	-1.290176	0.8898056	0.4089013	transport and golgi organization 2 homolog (Drosophila)

UP in Normoxia	**54931**	TRMT10C	-1.075939	0.8678312	0.4743621	tRNA methyltransferase 10 homolog C (S. cerevisiae)
UP in Normoxia	**134430**	WDR36	-1.005186	0.8604407	0.4982059	WD repeat domain 36
UP in Normoxia	**84948**	TIGD5	-1.216288	0.8285318	0.4303887	tigger transposable element derived 5
UP in Normoxia	**171586**	ABHD3	-2.077348	0.9143062	0.2369496	abhydrolase domain containing 3
UP in Normoxia	**80010**	RMI1	-1.132394	0.8437696	0.4561581	RMI1, RecQ mediated genome instability 1, homolog (S. cerevisiae)
UP in Normoxia	**332**	BIRC5	-1.701953	0.9177103	0.3073698	baculoviral IAP repeat containing 5
UP in Normoxia	**1965**	EIF2S1	-1.090777	0.8818955	0.4695083	eukaryotic translation initiation factor 2, subunit 1 alpha, 35kDa
UP in Normoxia	**55740**	ENAH	-1.038742	0.8654125	0.4867517	enabled homolog (Drosophila)
UP in Normoxia	**10755**	GIPC1	-1.185898	0.8898056	0.4395508	GIPC PDZ domain containing family,
UP in Normoxia	**79007**	DBNDD1	-1.562489	0.8547075	0.3385666	dysbindin (dystrobrevin binding protein 1) domain containing 1
UP in Normoxia	**79596**	RNF219	-1.349013	0.8747917	0.3925605	ring finger protein 219
UP in Normoxia	**55035**	NOL8	-1.312068	0.8761892	0.4027431	nucleolar protein 8
UP in Normoxia	**57491**	AHRR	-1.467263	0.8834722	0.3616677	aryl-hydrocarbon receptor repressor
UP in Normoxia	**3491**	CYR61	-1.109485	0.8683598	0.4634595	cysteine-rich, angiogenic inducer, 61
UP in Normoxia	**54682**	MANSC1	-2.116193	0.8245543	0.2306548	MANSC domain containing 1
UP in Normoxia	**54704**	PDP1	-1.072168	0.8740213	0.4756037	pyruvate dehyrogenase phosphatase catalytic subunit 1
UP in Normoxia	**3619**	INCENP	-1.81156	0.9161874	0.2848827	inner centromere protein antigens
UP in Normoxia	**64975**	MRPL41	-1.153383	0.8873242	0.4495698	mitochondrial ribosomal protein L41
UP in Normoxia	**10051**	SMC4	-1.708292	0.9180686	0.3060221	structural maintenance of chromosomes 4
UP in Normoxia	**5394**	EXOSC1	-1.011283	0.8759921	0.4961048	exosome component 10
UP in Normoxia	**6448**	SGSH	-2.02429	0.9291409	0.2458261	N-sulfoglucosamine sulfohydrolase
UP in Normoxia	**192111**	PGAM5	-1.611783	0.9117262	0.3271938	phosphoglycerate mutase family member 5
UP in Normoxia	**57464**	STRIP2	-2.206881	0.9311565	0.2166021	striatin interacting protein 2
UP in Normoxia	**201973**	CCDC11	-1.306264	0.8155424	0.4043668	coiled-coil domain containing 111
UP in Normoxia	**149951**	COMMD	-1.429117	0.8961928	0.3713582	COMM domain containing 7
UP in Normoxia	**653308**	ASAH2B	-1.409078	0.8767177	0.3765522	N-acylsphingosine amidohydrolase (non-lysosomal ceramidase) 2B
UP in Normoxia	**7584**	ZNF35	-1.227133	0.8352683	0.4271654	zinc finger protein 35
UP in Normoxia	**64764**	CREB3L2	-1.23186	0.8895189	0.4257683	cAMP responsive element binding protein 3-like 2
UP in Normoxia	**201255**	LRRC45	-2.193403	0.9197438	0.2186351	leucine rich repeat containing 45
UP in Normoxia	**8568**	RRP1	-1.232945	0.8894114	0.4254481	ribosomal RNA processing 1 homolog (S. cerevisiae)
UP in Normoxia	**151648**	SGOL1	-1.94453	0.9005644	0.2597995	shugoshin-like 1 (S. pombe)
UP in Normoxia	**55862**	ECHDC1	-1.229082	0.8886858	0.4265887	enoyl CoA hydratase domain containing 1
UP in Normoxia	**64222**	TOR3A	-2.628358	0.9468064	0.1617281	torsin family 3, member A
UP in Normoxia	**10189**	ALYREF	-1.904826	0.9232912	0.2670486	Aly/REF export factor
UP in Normoxia	**100533483**	DYX1C1-CCPG1	-2.929047	0.891436	0.1313013	DYX1C1-CCPG1 readthrough (non-protein coding)
UP in Normoxia	**152559**	PAQR3	-1.792012	0.9148616	0.2887691	progestin and adipoQ receptor family
UP in Hypoxia	**9363**	RAB33A	2.214099	0.9176207	4.6399177	RAB33A, member RAS oncogene family
UP in Hypoxia	**57026**	PDXP	1.289521	0.8906835	2.4444694	pyridoxal (pyridoxine, vitamin B6)
UP in Hypoxia	**57658**	CALCOCO1	1.321082	0.8949297	2.4985337	calcium binding and coiled-coil domain 1
UP in Hypoxia	**100534611**	TM4SF19-TCTEX1D2	1.057072	0.8555317	2.0807043	TM4SF19-TCTEX1D2 readthrough
UP in Hypoxia	**8941**	CDK5R2	1.220198	0.8034399	2.3297872	cyclin-dependent kinase 5, regulatory subunit 2 (p39)
UP in Hypoxia	**727800**	RNF208	2.296241	0.8872794	4.9117647	ring finger protein 208
UP in Hypoxia	**710**	SERPING1	4.724027	0.9496282	26.428571	serpin peptidase inhibitor, clade G (C1 inhibitor), member 1

UP in Hypoxia	**7087**	ICAM5	4.546593	0.9894383	23.370115	intercellular adhesion molecule 5,
UP in Hypoxia	**6513**	SLC2A1	3.813783	0.9835797	14.062514	solute carrier family 2 (facilitated glucose transporter), member 1
UP in Hypoxia	**1294**	COL7A1	1.528893	0.9032339	2.8856433	collagen, type VII, alpha 1
UP in Hypoxia	**644128**	RPL23A P53	1.464575	0.8930574	2.759821	ribosomal protein L23a pseudogene 53
UP in Hypoxia	**5224**	PGAM2	1.125052	0.8418257	2.1810934	phosphoglycerate mutase 2 (muscle)
UP in Hypoxia	**9110**	MTMR4	1.87747	0.9238825	3.6743005	myotubularin related protein 4
UP in Hypoxia	**1289**	COL5A1	1.495577	0.8990236	2.819769	collagen, type V, alpha 1
UP in Hypoxia	**79625**	NDNF	3.681311	0.9766102	12.828767	neuron-derived neurotrophic factor
UP in Hypoxia	**55186**	SLC25A3 6	1.600345	0.9161874	3.0321573	solute carrier family 25 (pyrimidine nucleotide carrier), member 36
UP in Hypoxia	**84706**	GPT2	1.657465	0.9159007	3.1546166	glutamic pyruvate transaminase (alanine aminotransferase) 2
UP in Hypoxia	**168455**	CCDC71	1.01944	0.8778912	2.0271318	coiled-coil domain containing 71-like
UP in Hypoxia	**116987**	AGAP1	1.111129	0.8760817	2.1601463	ArfGAP with GTPase domain, ankyrin repeat and PH domain 1
UP in Hypoxia	**3687**	ITGAX	1.0119	0.8426498	2.016565	integrin, alpha X (complement component 3 receptor 4 subunit)
UP in Hypoxia	**63915**	BLOC1S 5	1.513013	0.824187	2.8540541	biogenesis of lysosomal organelles complex-1, subunit 5, muted
UP in Hypoxia	**51200**	CPA4	3.828024	0.9835349	14.202015	carboxypeptidase A4
UP in Hypoxia	**23363**	OBSL1	1.246061	0.8746215	2.3719298	obscurin-like 1
UP in Hypoxia	**10507**	SEMA4D	2.856232	0.9504076	7.2412178	sema domain, immunoglobulin domain (Ig), transmembrane domain (TM) and short cytoplasmic domain, (semaphorin) 4D
UP in Hypoxia	**29085**	PHPT1	1.12083	0.8855594	2.1747201	phosphohistidine phosphatase 1
UP in Hypoxia	**131583**	FAM43A	2.778279	0.9133656	6.8603352	family with sequence similarity 43, member
UP in Hypoxia	**338442**	HCAR2	3.310103	0.9555048	9.9183673	hydroxycarboxylic acid receptor 2
UP in Hypoxia	**7032**	TFF2	4.665336	0.8358954	25.375	trefoil factor 2
UP in Hypoxia	**56833**	SLAMF8	2.023303	0.8722566	4.0651341	SLAM family member 8
UP in Hypoxia	**154761**	LOC1547 61	3.499759	0.9747828	11.311815	family with sequence similarity 115, member C pseudogene
UP in Hypoxia	**4828**	NMB	1.991998	0.9232016	3.9778747	neuromedin B
UP in Hypoxia	**114897**	C1QTNF	1.208388	0.8898504	2.3107929	C1q and tumor necrosis factor related
UP in Hypoxia	**11174**	ADAMT S6	1.317124	0.854394	2.4916898	ADAM metallopeptidase with thrombospondin type 1 motif, 6
UP in Hypoxia	**7145**	TNS1	2.357202	0.9413599	5.1237569	tensin 1
UP in Hypoxia	**84725**	PLEKHA 8	1.062272	0.8619726	2.0882171	pleckstrin homology domain containing, family A (phosphoinositide binding specific) member 8
UP in Hypoxia	**26355**	FAM162	2.544371	0.9505241	5.8335389	family with sequence similarity 162,
UP in Hypoxia	**155382**	VPS37D	1.69543	0.9058228	3.2387344	vacuolar protein sorting 37 homolog D (S. cerevisiae)
UP in Hypoxia	**117195**	MRGPR	4.89573	0.988874	29.768817	MAS-related GPR, member X3
UP in Hypoxia	**2077**	ERF	1.130914	0.8857744	2.1899748	Ets2 repressor factor
UP in Hypoxia	**54928**	IMPAD1	1.033771	0.8799337	2.0473694	inositol monophosphatase domain
UP in Hypoxia	**374378**	GALNT1 8	1.570167	0.9016483	2.96939	UDP-N-acetyl-alpha-D-galactosamine: polypeptide N-acetylgalactosaminyl-transferase 18
UP in Hypoxia	**7163**	TPD52	1.401508	0.8932008	2.6417756	tumor protein D52
UP in Hypoxia	**339122**	RAB43	1.127767	0.874093	2.185202	RAB43, member RAS oncogene family
UP in Hypoxia	**10098**	TSPAN5	1.99427	0.9225298	3.984145	tetraspanin 5
UP in Hypoxia	**53831**	GPR84	2.620776	0.9532205	6.1508065	G protein-coupled receptor 84
UP in Hypoxia	**149708**	WFDC5	8.900364	0.9950372	477.83333	WAP four-disulfide core domain 5
UP in Hypoxia	**91947**	ARRDC4	1.378512	0.8876915	2.6	arrestin domain containing 4

UP in Hypoxia	**65996**	MGC2752	1.233499	0.8892323	2.3513662	CENPB DNA-binding domains containing 1 pseudogene
UP in Hypoxia	**2597**	GAPDH	1.204511	0.8908268	2.3045911	glyceraldehyde-3-phosphate dehydrogenase
UP in Hypoxia	**79930**	DOK3	4.063893	0.9814835	16.724518	docking protein 3
UP in Hypoxia	**7593**	MZF1	1.229304	0.8821912	2.3445378	myeloid zinc finger 1
UP in Hypoxia	**138429**	PIP5KL1	1.049595	0.8210069	2.0699482	phosphatidylinositol-4-phosphate 5-kinase-like 1
UP in Hypoxia	**9788**	MTSS1	1.209389	0.8849592	2.3123971	metastasis suppressor 1
UP in Hypoxia	**1959**	EGR2	4.394304	0.9884798	21.028933	early growth response 2
UP in Hypoxia	**6383**	SDC2	1.239856	0.8845292	2.3617503	syndecan 2
UP in Hypoxia	**100271835**	LIMS3-LOC440895	4.755825	0.9655917	27.017544	LIMS3-LOC440895 readthrough
UP in Hypoxia	**256714**	MAP7D2	2.736697	0.9310759	6.6654275	MAP7 domain containing 2
UP in Hypoxia	**10251**	SPRY3	2.727716	0.9302696	6.6240601	sprouty homolog 3 (Drosophila)
UP in Hypoxia	**54843**	SYTL2	2.464503	0.9470752	5.5193672	synaptotagmin-like 2
UP in Hypoxia	**25895**	METTL21B	2.330539	0.9458568	5.0299335	methyltransferase like 21B
UP in Hypoxia	**22917**	ZP1	3.92562	0.9830243	15.196	zona pellucida glycoprotein 1 (sperm
UP in Hypoxia	**23601**	CLEC5A	3.353789	0.9241602	10.223301	C-type lectin domain family 5, member A
UP in Hypoxia	**100507156**	LOC100507156	1.929022	0.857377	3.807971	uncharacterized LOC100507156
UP in Hypoxia	**55806**	HR	3.600559	0.8657529	12.130435	hair growth associated
UP in Hypoxia	**10397**	NDRG1	4.893579	0.9941593	29.724464	N-myc downstream regulated 1
UP in Hypoxia	**55139**	ANKZF1	1.111911	0.8808922	2.1613179	ankyrin repeat and zinc finger domain containing 1
UP in Hypoxia	**3105**	HLA-A	1.161868	0.88865	2.2374696	major histocompatibility complex, class I, A
UP in Hypoxia	**100132354**	LOC100132354	4.395585	0.845185	21.047619	uncharacterized LOC100132354
UP in Hypoxia	**771**	CA12	2.565474	0.9506495	5.9194951	carbonic anhydrase XII
UP in Hypoxia	**283070**	LOC283070	1.224616	0.8023291	2.336933	uncharacterized LOC283070
UP in Hypoxia	**51317**	PHF21A	1.277542	0.8837499	2.4242567	PHD finger protein 21A
UP in Hypoxia	**64856**	VWA1	3.830075	0.9508376	14.222222	von Willebrand factor A domain containing
UP in Hypoxia	**147040**	KCTD11	3.526612	0.9771119	11.524341	potassium channel tetramerisation domain containing 11
UP in Hypoxia	**283**	ANG	3.88031	0.9786617	14.726161	angiogenin, ribonuclease, RNase A family, 5
UP in Hypoxia	**115123**	MARCH3	1.514507	0.8854161	2.8570119	membrane-associated ring finger (C3HC4) 3, E3 ubiquitin protein ligase
UP in Hypoxia	**5990**	RFX2	1.332923	0.8565977	2.5191257	regulatory factor X, 2 (influences HLA class II expression)
UP in Hypoxia	**226**	ALDOA	1.103476	0.885873	2.1487176	aldolase A, fructose-bisphosphate
UP in Hypoxia	**139728**	PNCK	2.49057	0.8203798	5.62	pregnancy up-regulated non-ubiquitously expressed CaM kinase
UP in Hypoxia	**6604**	SMARCD3	2.014695	0.9323659	4.0409505	SWI/SNF related, matrix associated, actin dependent regulator of chromatin, subfamily d, member 3
UP in Hypoxia	**4232**	MEST	2.316884	0.9455254	4.982548	mesoderm specific transcript
UP in Hypoxia	**3136**	HLA-H	1.890177	0.8107319	3.7068063	major histocompatibility complex, class I, H (pseudogene)
UP in Hypoxia	**5863**	RGL2	1.016441	0.8700349	2.0229226	ral guanine nucleotide dissociation stimulator-like 2
UP in Hypoxia	**100505681**	NAPA-AS1	2.111508	0.8346323	4.3214286	NAPA antisense RNA 1
UP in Hypoxia	**4327**	MMP19	2.168893	0.9373556	4.4967811	matrix metallopeptidase 19
UP in Hypoxia	**574029**	DUSP5P	4.447982	0.866371	21.826087	dual specificity phosphatase 5 pseudogene 1
UP in Hypoxia	**6920**	TCEA3	2.236124	0.8832572	4.7112971	transcription elongation factor A (SII), 3

UP in Hypoxia	**55272**	IMP3	1.225714	0.8908806	2.3387115	IMP3, U3 small nucleolar ribonucleoprotein, homolog (yeast)
UP in Hypoxia	**353137**	LCE1F	3.583167	0.9523157	11.985075	late cornified envelope 1F
UP in Hypoxia	**118433**	RPL23A	1.099503	0.884377	2.1428093	ribosomal protein L23a pseudogene 7
UP in Hypoxia	**140686**	WFDC3	3.335411	0.9670698	10.093897	WAP four-disulfide core domain 3
UP in Hypoxia	**2548**	GAA	1.280408	0.8846815	2.4290764	glucosidase, alpha; acid
UP in Hypoxia	**29923**	HILPDA	6.187689	0.9984682	72.892032	hypoxia inducible lipid droplet-associated
UP in Hypoxia	**9120**	SLC16A6	2.914927	0.9629938	7.5418959	solute carrier family 16, member 6 (monocarboxylic acid transporter 7)
UP in Hypoxia	**622**	BDH1	3.993545	0.8388426	15.928571	3-hydroxybutyrate dehydrogenase, type 1
UP in Hypoxia	**2256**	FGF11	4.021311	0.8996775	16.238095	fibroblast growth factor 11
UP in Hypoxia	**5768**	QSOX1	1.33898	0.8971603	2.5297241	quiescin Q6 sulfhydryl oxidase 1
UP in Hypoxia	**57186**	RALGAP A2	1.249879	0.8391024	2.3782148	Ral GTPase activating protein, alpha subunit 2 (catalytic)
UP in Hypoxia	**261734**	NPHP4	1.274931	0.8376512	2.4198718	nephronophthisis 4
UP in Hypoxia	**5900**	RALGDS	1.734979	0.914772	3.3287469	ral guanine nucleotide dissociation
UP in Hypoxia	**84326**	C16orf13	1.031618	0.8798173	2.0443158	chromosome 16 open reading frame 13
UP in Hypoxia	**3017**	HIST1H2 BD	2.432018	0.9390666	5.3964758	histone cluster 1, H2bd
UP in Hypoxia	**79729**	SH3D21	2.518086	0.9458658	5.7282174	SH3 domain containing 21
UP in Hypoxia	**401152**	C4orf3	1.535197	0.9021858	2.8982795	chromosome 4 open reading frame 3
UP in Hypoxia	**2114**	ETS2	1.042857	0.8800143	2.0603037	v-ets erythroblastosis virus E26 oncogene homolog 2 (avian)
UP in Hypoxia	**10058**	ABCB6	1.101704	0.8680373	2.1460801	ATP-binding cassette, sub-family B (MDR/TAP), member 6
UP in Hypoxia	**3939**	LDHA	1.121478	0.8866971	2.1756974	lactate dehydrogenase A
UP in Hypoxia	**9066**	SYT7	6.612734	0.9956284	97.865854	synaptotagmin VII
UP in Hypoxia	**56270**	WDR45B	1.264269	0.8931649	2.4020543	WD repeat domain 45B
UP in Hypoxia	**8870**	IER3	1.987376	0.9257995	3.9651506	immediate early response 3
UP in Hypoxia	**9456**	HOMER	1.259119	0.8777031	2.3934949	homer homolog 1 (Drosophila)
UP in Hypoxia	**79939**	SLC35E1	1.411861	0.8976978	2.6608011	solute carrier family 35, member E1
UP in Hypoxia	**3339**	HSPG2	2.151928	0.9290782	4.4442127	heparan sulfate proteoglycan 2
UP in Hypoxia	**9744**	ACAP1	2.577507	0.833826	5.9690722	ArfGAP with coiled-coil, ankyrin repeat and PH domains 1
UP in Hypoxia	**84218**	TBC1D3	1.354719	0.8897877	2.5574737	TBC1 domain family, member 3F
UP in Hypoxia	**219539**	YPEL4	1.887127	0.8139837	3.6989796	yippee-like 4 (Drosophila)
UP in Hypoxia	**80194**	TMEM13	1.083743	0.8780704	2.1195278	transmembrane protein 134
UP in Hypoxia	**147906**	DACT3	1.595813	0.9033772	3.0226481	dapper, antagonist of beta-catenin, homolog 3 (Xenopus laevis)
UP in Hypoxia	**642799**	LOC6427 99	1.176878	0.8501299	2.2608696	nuclear pore complex-interacting protein-like 1-like
UP in Hypoxia	**3623**	INHA	3.902074	0.8802472	14.95	inhibin, alpha
UP in Hypoxia	**4163**	MCC	1.716821	0.9178715	3.2871126	mutated in colorectal cancers
UP in Hypoxia	**83943**	IMMP2L	1.269316	0.8705276	2.410473	IMP2 inner mitochondrial membrane peptidase-like (S. cerevisiae)
UP in Hypoxia	**100134229**	LOC1001 34229	2.902357	0.9469229	7.4764706	uncharacterized LOC100134229
UP in Hypoxia	**5125**	PCSK5	2.604538	0.8673475	6.0819672	proprotein convertase subtilisin/kexin type 5
UP in Hypoxia	**1291**	COL6A1	1.836519	0.923766	3.5714726	collagen, type VI, alpha 1
UP in Hypoxia	**200058**	FLJ23867	1.292828	0.8408672	2.4500792	uncharacterized protein FLJ23867
UP in Hypoxia	**84937**	ZNRF1	1.492642	0.8890531	2.8140376	zinc and ring finger 1, E3 ubiquitin protein
UP in Hypoxia	**8614**	STC2	1.557988	0.9038878	2.9444293	stanniocalcin 2
UP in Hypoxia	**55876**	GSDMB	1.78967	0.9096032	3.4573571	gasdermin B
UP in Hypoxia	**3484**	IGFBP1	2.989596	0.9576368	7.9425134	insulin-like growth factor binding protein 1
UP in Hypoxia	**22890**	ZBTB1	1.72499	0.9174774	3.3057775	zinc finger and BTB domain containing 1
UP in Hypoxia	**283229**	EFCAB4	1.715478	0.9167876	3.2840549	EF-hand calcium binding domain 4A
UP in Hypoxia	**3708**	ITPR1	1.901093	0.9210517	3.7349617	inositol 1,4,5-trisphosphate receptor, type 1

UP in Hypoxia	**25794**	FSCN2	3.484321	0.9674012	11.191419	fascin homolog 2, actin-bundling protein, retinal (Strongylocentrotus purpuratus)
UP in Hypoxia	**401320**	LOC401320	1.217591	0.8040133	2.3255814	uncharacterized LOC401320
UP in Hypoxia	**54429**	TAS2R5	4.084965	0.8797635	16.970588	taste receptor, type 2, member 5
UP in Hypoxia	**2668**	GDNF	1.890862	0.922055	3.7085684	glial cell derived neurotrophic factor
UP in Hypoxia	**23081**	KDM4C	1.209862	0.850954	2.3131548	lysine (K)-specific demethylase 4C
UP in Hypoxia	**23764**	MAFF	1.48201	0.9008331	2.7933757	v-maf musculoaponeurotic fibrosarcoma oncogene homolog F (avian)
UP in Hypoxia	**2100**	ESR2	3.339442	0.9375795	10.122137	estrogen receptor 2 (ER beta)
UP in Hypoxia	**147081**	CRHR1-IT1	1.754397	0.8764579	3.3738532	CRHR1 intronic transcript 1 (non-protein coding)
UP in Hypoxia	**7226**	TRPM2	3.638074	0.8496372	12.45	transient receptor potential cation channel, subfamily M, member 2
UP in Hypoxia	**5507**	PPP1R3C	2.481582	0.9496193	5.5850945	protein phosphatase 1, regulatory subunit 3C
UP in Hypoxia	**1027**	CDKN1B	1.306355	0.8922781	2.4731592	cyclin-dependent kinase inhibitor 1B (p27,
UP in Hypoxia	**1906**	EDN1	2.572116	0.8217683	5.9468085	endothelin 1
UP in Hypoxia	**54869**	EPS8L1	1.087257	0.854815	2.1246964	EPS8-like 1
UP in Hypoxia	**92565**	FANK1	2.172023	0.9139031	4.5065502	fibronectin type III and ankyrin repeat
UP in Hypoxia	**378706**	RN7SL2	1.110558	0.8849324	2.159292	RNA, 7SL, cytoplasmic 2
UP in Hypoxia	**83853**	ROPN1L	1.878009	0.8460898	3.6756757	rhophilin associated tail protein 1-like
UP in Hypoxia	**54541**	DDIT4	1.944751	0.9252083	3.8497142	DNA-damage-inducible transcript 4
UP in Hypoxia	**3099**	HK2	2.822208	0.9628684	7.0724415	hexokinase 2
UP in Hypoxia	**125228**	FAM210	1.374022	0.8914002	2.5919215	family with sequence similarity 210,
UP in Hypoxia	**100505715**	LOC100505715	2.45158	0.9126668	5.4701493	uncharacterized LOC100505715
UP in Hypoxia	**23199**	GSE1	1.046122	0.8132402	2.0649718	Gse1 coiled-coil protein
UP in Hypoxia	**256949**	KANK3	2.213085	0.8991311	4.6366559	KN motif and ankyrin repeat domains 3
UP in Hypoxia	**644714**	LIMD1-AS1	1.266826	0.8895189	2.4063153	LIMD1 antisense RNA 1
UP in Hypoxia	**50486**	G0S2	2.625763	0.9520022	6.1721068	G0/G1switch 2
UP in Hypoxia	**164237**	WFDC13	3.321928	0.8166891	10	WAP four-disulfide core domain 13
UP in Hypoxia	**154664**	ABCA13	5.080299	0.9848965	33.831579	ATP-binding cassette, sub-family A (ABC1), member 13
UP in Hypoxia	**283130**	SLC25A4	1.673556	0.8626265	3.19	solute carrier family 25, member 45
UP in Hypoxia	**7133**	TNFRSF1B	1.204107	0.8895996	2.3039456	tumor necrosis factor receptor superfamily, member 1B
UP in Hypoxia	**29995**	LMCD1	1.254151	0.8690047	2.3852667	LIM and cysteine-rich domains 1
UP in Hypoxia	**22997**	IGSF9B	2.517233	0.9334856	5.7248322	immunoglobulin superfamily, member 9B
UP in Hypoxia	**2192**	FBLN1	1.255847	0.8687808	2.3880726	fibulin 1
UP in Hypoxia	**254896**	LOC254896	1.821327	0.8707605	3.5340599	uncharacterized LOC254896
UP in Hypoxia	**6006**	RHCE	1.162653	0.8465556	2.2386878	Rh blood group, CcEe antigens
UP in Hypoxia	**144406**	WDR66	3.176054	0.9643107	9.0383142	WD repeat domain 66
UP in Hypoxia	**754**	PTTG1IP	1.996027	0.9367912	3.9889996	pituitary tumor-transforming 1 interacting protein
UP in Hypoxia	**230**	ALDOC	2.913153	0.9625459	7.5326274	aldolase C, fructose-bisphosphate
UP in Hypoxia	**7570**	ZNF22	1.015062	0.8729284	2.0209891	zinc finger protein 22
UP in Hypoxia	**7597**	ZBTB25	1.305449	0.87859	2.4716066	zinc finger and BTB domain containing 25
UP in Hypoxia	**146802**	SLC47A2	4.420332	0.8133477	21.411765	solute carrier family 47, member 2
UP in Hypoxia	**84458**	LCOR	1.02764	0.8256562	2.038687	ligand dependent nuclear receptor
UP in Hypoxia	**151556**	GPR155	3.037157	0.9575293	8.2087156	G protein-coupled receptor 155
UP in Hypoxia	**56975**	FAM20C	1.12555	0.8860701	2.1818473	family with sequence similarity 20, member
UP in Hypoxia	**3107**	HLA-C	1.6644	0.9197259	3.1698175	major histocompatibility complex, class I, C
UP in Hypoxia	**10307**	APBB3	1.663533	0.9098719	3.167913	amyloid beta (A4) precursor protein-binding, family B, member 3
UP in Hypoxia	**387763**	C11orf96	2.025768	0.9189644	4.0720859	chromosome 11 open reading frame 96

UP in Hypoxia	**440895**	LOC440895	3.681824	0.8061453	12.833333	two pore channel 3 pseudogene
UP in Hypoxia	**10076**	PTPRU	1.368777	0.8796291	2.5825163	protein tyrosine phosphatase, receptor type,
UP in Hypoxia	**1978**	EIF4EBP1	1.54538	0.9040133	2.9188092	eukaryotic translation initiation factor 4E binding protein 1
UP in Hypoxia	**2026**	ENO2	2.417992	0.9499418	5.3442673	enolase 2 (gamma, neuronal)
UP in Hypoxia	**10848**	PPP1R13L	1.404738	0.8628236	2.6476965	protein phosphatase 1, regulatory subunit 13 like
UP in Hypoxia	**7167**	TPI1	1.090941	0.8852997	2.1301298	triosephosphate isomerase 1
UP in Hypoxia	**3398**	ID2	1.918969	0.9191346	3.7815265	inhibitor of DNA binding 2, dominant negative helix-loop-helix protein
UP in Hypoxia	**5230**	PGK1	1.662206	0.9198065	3.1650006	phosphoglycerate kinase 1
UP in Hypoxia	**4741**	NEFM	2.073509	0.8609872	4.2090909	neurofilament, medium polypeptide
UP in Hypoxia	**5187**	PER1	1.262107	0.8817074	2.3984576	period circadian clock 1
UP in Hypoxia	**8840**	WISP1	2.287896	0.8522261	4.8834356	WNT1 inducible signaling pathway protein
UP in Hypoxia	**1326**	MAP3K8	2.098929	0.9278061	4.2839117	mitogen-activated protein kinase kinase
UP in Hypoxia	**170384**	FUT11	2.473577	0.9471647	5.5541911	fucosyltransferase 11 (alpha (1,3) fucosyltransferase)
UP in Hypoxia	**5054**	SERPINE1	1.907856	0.92485	3.7525103	serpin peptidase inhibitor, clade E (nexin, plasminogen activator inhibitor type 1), member 1
UP in Hypoxia	**122622**	ADSSL1	1.739221	0.8976709	3.3385491	adenylosuccinate synthase like 1
UP in Hypoxia	**2042**	EPHA3	5.499846	0.8867061	45.25	EPH receptor A3
UP in Hypoxia	**6095**	RORA	1.257739	0.8800143	2.3912063	RAR-related orphan receptor A
UP in Hypoxia	**5163**	PDK1	1.681252	0.9160709	3.2070604	pyruvate dehydrogenase kinase, isozyme 1
UP in Hypoxia	**158056**	MAMDC	2.835677	0.9355191	7.1387755	MAM domain containing 4
UP in Hypoxia	**100126793**	GHRLOS	1.480952	0.8186957	2.7913279	ghrelin opposite strand/antisense RNA
UP in Hypoxia	**441172**	FLJ46906	2.480873	0.8830959	5.5823529	uncharacterized LOC441172
UP in Hypoxia	**5526**	PPP2R5B	2.031944	0.9363522	4.0895544	protein phosphatase 2, regulatory subunit B', beta
UP in Hypoxia	**4312**	MMP1	1.056053	0.8805877	2.0792353	matrix metallopeptidase 1 (interstitial collagenase)
UP in Hypoxia	**10882**	C1QL1	1.559392	0.8743438	2.9472954	complement component 1, q subcomponent-like 1
UP in Hypoxia	**350**	APOH	3.52122	0.9661023	11.481343	apolipoprotein H (beta-2-glycoprotein I)
UP in Hypoxia	**9208**	LRRFIP1	1.246757	0.8916868	2.3730733	leucine rich repeat (in FLII) interacting
UP in Hypoxia	**149603**	RNF187	1.413541	0.8990952	2.6639015	ring finger protein 187
UP in Hypoxia	**4046**	LSP1	3.666757	0.8045955	12.7	lymphocyte-specific protein 1
UP in Hypoxia	**56967**	C14orf13	1.505312	0.8986742	2.8388596	chromosome 14 open reading frame 132
UP in Hypoxia	**342908**	ZNF404	2.845737	0.9467795	7.1887324	zinc finger protein 404
UP in Hypoxia	**390928**	PAPL	1.00413	0.878169	2.0057331	iron/zinc purple acid phosphatase-like
UP in Hypoxia	**23429**	RYBP	1.17348	0.8812147	2.2555516	RING1 and YY1 binding protein
UP in Hypoxia	**5165**	PDK3	1.658626	0.9046582	3.1571562	pyruvate dehydrogenase kinase, isozyme 3
UP in Hypoxia	**27129**	HSPB7	4.457974	0.939837	21.977778	heat shock 27kDa protein family, member 7 (cardiovascular)
UP in Hypoxia	**27063**	ANKRD1	3.931956	0.9732778	15.262887	ankyrin repeat domain 1 (cardiac muscle)
UP in Hypoxia	**56925**	LXN	1.955125	0.9202544	3.877496	latexin
UP in Hypoxia	**27124**	INPP5J	2.559199	0.8459912	5.8938053	inositol polyphosphate-5-phosphatase J
UP in Hypoxia	**23119**	HIC2	1.264012	0.8354654	2.401626	hypermethylated in cancer 2
UP in Hypoxia	**6038**	RNASE4	4.293262	0.9880408	19.606519	ribonuclease, RNase A family, 4
UP in Hypoxia	**94120**	SYTL3	1.85533	0.9225746	3.6183449	synaptotagmin-like 3
UP in Hypoxia	**22936**	ELL2	1.291699	0.8935949	2.4481618	elongation factor, RNA polymerase II, 2
UP in Hypoxia	**467**	ATF3	1.584963	0.9175401	3	activating transcription factor 3
UP in Hypoxia	**4490**	MT1B	2.935595	0.9325092	7.6507177	metallothionein 1B
UP in Hypoxia	**83706**	FERMT3	1.178673	0.8775329	2.263684	fermitin family member 3
UP in Hypoxia	**4776**	NFATC4	2.376823	0.9081161	5.1939163	nuclear factor of activated T-cells, cytoplasmic, calcineurin-dependent 4

UP in Hypoxia	**4093**	SMAD9	1.965179	0.9052047	3.9046105	SMAD family member 9
UP in Hypoxia	**92745**	SLC38A5	3.153805	0.8631192	8.9	solute carrier family 38, member 5
UP in Hypoxia	**4015**	LOX	4.75451	0.9935053	26.992939	lysyl oxidase
UP in Hypoxia	**6641**	SNTB1	1.275191	0.8850309	2.4203085	syntrophin, beta 1 (dystrophin-associated protein A1, 59kDa, basic component 1)
UP in Hypoxia	**114879**	OSBPL5	1.434496	0.8893667	2.7028773	oxysterol binding protein-like 5
UP in Hypoxia	**57572**	DOCK6	1.000434	0.8571173	2.0006013	dedicator of cytokinesis 6
UP in Hypoxia	**333926**	PPM1J	1.219734	0.866389	2.3290378	protein phosphatase, Mg2+/Mn2+ dependent, 1J
UP in Hypoxia	**723790**	HIST2H2 AA4	1.59303	0.9141181	3.0168224	histone cluster 2, H2aa4
UP in Hypoxia	**83937**	RASSF4	1.556136	0.8977515	2.9406528	Ras association (RalGDS/AF-6) domain family member 4
UP in Hypoxia	**4814**	NINJ1	1.58844	0.9180238	3.0072391	ninjurin 1
UP in Hypoxia	**6611**	SMS	1.067645	0.8838932	2.0960091	spermine synthase
UP in Hypoxia	**4665**	NAB2	1.063192	0.8796918	2.0895499	NGFI-A binding protein 2 (EGR1 binding protein 2)
UP in Hypoxia	**280664**	WFDC10	4.361811	0.956526	20.560606	WAP four-disulfide core domain 10B
UP in Hypoxia	**6640**	SNTA1	1.502073	0.9007435	2.8324933	syntrophin, alpha 1
UP in Hypoxia	**79953**	SYNDIG	2.263745	0.9370062	4.8023649	synapse differentiation inducing 1
UP in Hypoxia	**100506730**	LOC1005 06730	2.154478	0.9242229	4.4520744	uncharacterized LOC100506730
UP in Hypoxia	**1917**	EEF1A2	5.904885	0.9677596	59.916667	eukaryotic translation elongation factor 1 alpha 2
UP in Hypoxia	**861**	RUNX1	1.197123	0.8874496	2.2928196	runt-related transcription factor 1
UP in Hypoxia	**337**	APOA4	6.972207	0.9976888	125.55769	apolipoprotein A-IV
UP in Hypoxia	**30001**	ERO1L	2.403093	0.9495118	5.2893607	ERO1-like (S. cerevisiae)
UP in Hypoxia	**7045**	TGFBI	1.175149	0.890155	2.2581613	transforming growth factor, beta-induced,
UP in Hypoxia	**440944**	SETD5-AS1	1.014683	0.8570277	2.0204588	SETD5 antisense RNA 1
UP in Hypoxia	**641649**	TMEM91	1.351326	0.8806145	2.5514651	transmembrane protein 91
UP in Hypoxia	**1803**	DPP4	1.216094	0.8853803	2.3231688	dipeptidyl-peptidase 4
UP in Hypoxia	**257364**	SNX33	1.572719	0.9018364	2.9746491	sorting nexin 33
UP in Hypoxia	**57613**	KIAA146	1.49181	0.8902177	2.8124157	KIAA1467
UP in Hypoxia	**3300**	DNAJB2	1.093155	0.883947	2.1334012	DnaJ (Hsp40) homolog, subfamily B,
UP in Hypoxia	**6548**	SLC9A1	1.158652	0.8841351	2.2324867	solute carrier family 9, subfamily A (NHE1, cation proton antiporter 1), member 1
UP in Hypoxia	**3998**	LMAN1	1.622355	0.9186778	3.0787725	lectin, mannose-binding, 1
UP in Hypoxia	**126731**	CCSAP	1.696896	0.9086536	3.2420278	centriole, cilia and spindle-associated
UP in Hypoxia	**389524**	GTF2IR D2B	1.509635	0.8395593	2.8473804	GTF2I repeat domain containing 2B
UP in Hypoxia	**93129**	ORAI3	1.126867	0.8805787	2.1838404	ORAI calcium release-activated calcium modulator 3
UP in Hypoxia	**623**	BDKRB1	2.220137	0.9391382	4.6593768	bradykinin receptor B1
UP in Hypoxia	**10424**	PGRMC2	1.246675	0.8873779	2.3729382	progesterone receptor membrane component
UP in Hypoxia	**9478**	CABP1	2.889003	0.9553346	7.4075829	calcium binding protein 1
UP in Hypoxia	**4035**	LRP1	1.314353	0.8954045	2.4869083	low density lipoprotein receptor-related protein 1
UP in Hypoxia	**4684**	NCAM1	1.326342	0.8695691	2.5076609	neural cell adhesion molecule 1
UP in Hypoxia	**9557**	CHD1L	1.193345	0.8883992	2.2868235	chromodomain helicase DNA binding protein 1-like
UP in Hypoxia	**6348**	CCL3	1.340248	0.8970259	2.5319483	chemokine (C-C motif) ligand 3
UP in Hypoxia	**55340**	GIMAP5	8.273018	0.9465645	309.33333	GTPase, IMAP family member 5
UP in Hypoxia	**8660**	IRS2	1.798248	0.8954851	3.4779772	insulin receptor substrate 2
UP in Hypoxia	**51141**	INSIG2	2.585031	0.9563917	6.0002837	insulin induced gene 2
UP in Hypoxia	**8553**	BHLHE4	1.837678	0.9237212	3.5743422	basic helix-loop-helix family, member e40
UP in Hypoxia	**643641**	ZNF862	1.0229	0.826758	2.032	zinc finger protein 862

UP in Hypoxia	**9294**	S1PR2	1.173118	0.8332169	2.2549858	sphingosine-1-phosphate receptor 2
UP in Hypoxia	**55558**	PLXNA3	1.158054	0.8842247	2.2315619	plexin A3
UP in Hypoxia	**27440**	CECR5	1.420974	0.8977605	2.6776622	cat eye syndrome chromosome region, candidate 5
UP in Hypoxia	**24142**	NAT6	1.330581	0.8879961	2.5150393	N-acetyltransferase 6 (GCN5-related)
UP in Hypoxia	**122402**	TDRD9	4.153805	0.8037445	17.8	tudor domain containing 9
UP in Hypoxia	**55124**	PIWIL2	3.47468	0.9106781	11.116883	piwi-like RNA-mediated gene silencing 2
UP in Hypoxia	**10802**	SEC24A	1.269055	0.8900923	2.4100357	SEC24 family, member A (S. cerevisiae)
UP in Hypoxia	**2494**	NR5A2	3.906891	0.8854071	15	nuclear receptor subfamily 5, group A,
UP in Hypoxia	**23704**	KCNE4	2.966242	0.9368539	7.814978	potassium voltage-gated channel, Isk-related family, member 4
UP in Hypoxia	**196527**	ANO6	1.222655	0.8882648	2.3337574	anoctamin 6
UP in Hypoxia	**29780**	PARVB	1.059303	0.8824241	2.0839249	parvin, beta
UP in Hypoxia	**8780**	RIOK3	1.357485	0.8955388	2.562381	RIO kinase 3
UP in Hypoxia	**100288332**	LOC1002 88332	1.245266	0.8883544	2.3706226	NPIP-like protein 1-like
UP in Hypoxia	**53844**	COPG2I T1	3.127221	0.8772104	8.7375	COPG2 imprinted transcript 1 (non-protein coding)
UP in Hypoxia	**126917**	IFFO2	1.512879	0.8812147	2.8537887	intermediate filament family orphan 2
UP in Hypoxia	**3720**	JARID2	1.783401	0.9122189	3.4423676	jumonji, AT rich interactive domain 2
UP in Hypoxia	**9510**	ADAMT S1	1.019254	0.8783839	2.0268702	ADAM metallopeptidase with thrombospondin type 1 motif, 1
UP in Hypoxia	**23253**	ANKRD1	1.007562	0.8493953	2.0105105	ankyrin repeat domain 12
UP in Hypoxia	**9668**	ZNF432	1.207782	0.8775688	2.3098224	zinc finger protein 432
UP in Hypoxia	**6533**	SLC6A6	1.925862	0.9243752	3.7996383	solute carrier family 6 (neurotransmitter transporter, taurine), member 6
UP in Hypoxia	**8609**	KLF7	1.074792	0.8712801	2.1064189	Kruppel-like factor 7 (ubiquitous)
UP in Hypoxia	**57643**	ZSWIM5	2.807355	0.9546269	7	zinc finger, SWIM-type containing 5
UP in Hypoxia	**1187**	CLCNKA	5.120094	0.9782854	34.777778	chloride channel, voltage-sensitive Ka
UP in Hypoxia	**58494**	JAM2	3.115762	0.9654573	8.6683748	junctional adhesion molecule 2
UP in Hypoxia	**10570**	DPYSL4	3.259525	0.9664338	9.5766773	dihydropyrimidinase-like 4
UP in Hypoxia	**4335**	MNT	1.133449	0.8764759	2.1938257	MNT, MAX dimerization protein
UP in Hypoxia	**6855**	SYP	1.898623	0.8247604	3.7285714	synaptophysin
UP in Hypoxia	**11026**	LILRA3	5.292431	0.9309684	39.190476	leukocyte immunoglobulin-like receptor, subfamily A (without TM domain), member 3
UP in Hypoxia	**23097**	CDK19	1.184124	0.8777748	2.2722538	cyclin-dependent kinase 19
UP in Hypoxia	**283120**	H19	6.118941	0.849673	69.5	H19, imprinted maternally expressed transcript (non-protein coding)
UP in Hypoxia	**100874249**	DENND5 B-AS1	1.319068	0.8737167	2.4950495	DENND5B antisense RNA 1
UP in Hypoxia	**9337**	CNOT8	1.549719	0.9018364	2.9276001	CCR4-NOT transcription complex, subunit
UP in Hypoxia	**51517**	NCKIPS	1.009134	0.8720326	2.0127031	NCK interacting protein with SH3 domain
UP in Hypoxia	**2796**	GNRH1	2.019584	0.9051868	4.0546697	gonadotropin-releasing hormone 1 (luteinizing-releasing hormone)
UP in Hypoxia	**253461**	ZBTB38	1.034988	0.8777838	2.0490969	zinc finger and BTB domain containing 38
UP in Hypoxia	**7076**	TIMP1	1.379709	0.8989698	2.6021589	TIMP metallopeptidase inhibitor 1
UP in Hypoxia	**57478**	USP31	1.053044	0.8527815	2.0749035	ubiquitin specific peptidase 31
UP in Hypoxia	**55824**	PAG1	2.02453	0.9278509	4.0685921	phosphoprotein associated with glycosphingolipid microdomains 1
UP in Hypoxia	**604**	BCL6	1.117237	0.8819224	2.1693111	B-cell CLL/lymphoma 6
UP in Hypoxia	**5798**	PTPRN	1.290371	0.893586	2.4459095	protein tyrosine phosphatase, receptor type,
UP in Hypoxia	**594842**	HAS2-AS1	2.033631	0.8532115	4.0943396	HAS2 antisense RNA 1
UP in Hypoxia	**254263**	CNIH2	1.225988	0.839398	2.3391557	cornichon homolog 2 (Drosophila)
UP in Hypoxia	**5144**	PDE4D	2.241324	0.926767	4.7283073	phosphodiesterase 4D, cAMP-specific
UP in Hypoxia	**7728**	ZNF175	2.137122	0.9375437	4.3988352	zinc finger protein 175

UP in Hypoxia	**3669**	ISG20	5.33467	0.9595628	40.354839	interferon stimulated exonuclease gene
UP in Hypoxia	**10252**	SPRY1	2.902214	0.8804533	7.4757282	sprouty homolog 1, antagonist of FGF signaling (Drosophila)
UP in Hypoxia	**10370**	CITED2	1.614294	0.9172893	3.0616164	Cbp/p300-interacting transactivator, with Glu/Asp-rich carboxy-terminal domain, 2
UP in Hypoxia	**100630923**	LOC1006 30923	1.021417	0.8545821	2.0299116	LOC100289561-PRKRIP1 readthrough
UP in Hypoxia	**25823**	TPSG1	5.514983	0.8757502	45.727272	tryptase gamma 1
UP in Hypoxia	**271**	AMPD2	1.204288	0.8891696	2.3042359	adenosine monophosphate deaminase 2
UP in Hypoxia	**27032**	ATP2C1	1.012	0.8789125	2.0167049	ATPase, Ca++ transporting, type 2C,
UP in Hypoxia	**154860**	FEZF1-AS1	1.282424	0.8430529	2.4324734	FEZF1 antisense RNA 1
UP in Hypoxia	**4071**	TM4SF1	1.053093	0.8829705	2.0749735	transmembrane 4 L six family member 1
UP in Hypoxia	**55689**	YEATS2	1.424443	0.8971244	2.6841081	YEATS domain containing 2
UP in Hypoxia	**5028**	P2RY1	2.411395	0.9223506	5.3198847	purinergic receptor P2Y, G-protein coupled,
UP in Hypoxia	**2113**	ETS1	2.056642	0.9361731	4.1601688	v-ets erythroblastosis virus E26 oncogene homolog 1 (avian)
UP in Hypoxia	**100288695**	LIMS3L	2.58077	0.9556123	5.982588	LIM and senescent cell antigen-like domains 3-like
UP in Hypoxia	**8862**	APLN	2.317304	0.9484189	4.984	apelin
UP in Hypoxia	**84251**	SGIP1	1.741311	0.8749261	3.3433875	SH3-domain GRB2-like (endophilin) interacting protein 1
UP in Hypoxia	**100861555**	LINC005	1.283442	0.8433934	2.4341906	long intergenic non-protein coding RNA
UP in Hypoxia	**53358**	SHC3	1.440878	0.8937562	2.7148594	SHC (Src homology 2 domain containing) transforming protein 3
UP in Hypoxia	**8841**	HDAC3	1.259075	0.8924662	2.3934231	histone deacetylase 3
UP in Hypoxia	**84678**	KDM2B	1.021869	0.8711278	2.0305472	lysine (K)-specific demethylase 2B
UP in Hypoxia	**2997**	GYS1	1.684443	0.9193586	3.2141622	glycogen synthase 1 (muscle)
UP in Hypoxia	**4494**	MT1F	1.709399	0.9200394	3.2702467	metallothionein 1F
UP in Hypoxia	**60682**	SMAP1	1.056284	0.8665592	2.0795688	small ArfGAP 1
UP in Hypoxia	**2908**	NR3C1	1.081882	0.8798441	2.1167954	nuclear receptor subfamily 3, group C, member 1 (glucocorticoid receptor)
UP in Hypoxia	**8987**	STBD1	1.334487	0.8857207	2.5218579	starch binding domain 1
UP in Hypoxia	**11346**	SYNPO	1.509197	0.8995611	2.8465165	synaptopodin
UP in Hypoxia	**794**	CALB2	2.341144	0.9424259	5.0670444	calbindin 2
UP in Hypoxia	**84695**	LOXL3	2.165601	0.9386455	4.4865344	lysyl oxidase-like 3
UP in Hypoxia	**593**	BCKDH A	1.29365	0.8936128	2.4514748	branched chain keto acid dehydrogenase E1, alpha polypeptide
UP in Hypoxia	**10123**	ARL4C	1.201264	0.8889367	2.2994102	ADP-ribosylation factor-like 4C
UP in Hypoxia	**692312**	PPAN-P2RY11	1.488981	0.8874496	2.8069074	PPAN-P2RY11 readthrough
UP in Hypoxia	**142678**	MIB2	1.254772	0.8848786	2.386294	mindbomb E3 ubiquitin protein ligase 2
UP in Hypoxia	**599**	BCL2L2	1.002516	0.8737347	2.0034916	BCL2-like 2
UP in Hypoxia	**23639**	LRRC6	1.325649	0.8530592	2.5064562	leucine rich repeat containing 6
UP in Hypoxia	**9988**	DMTF1	1.028357	0.8747201	2.0397004	cyclin D binding myb-like transcription
UP in Hypoxia	**2335**	FN1	1.120869	0.8866613	2.1747796	fibronectin 1
UP in Hypoxia	**6515**	SLC2A3	2.366797	0.9490639	5.1579475	solute carrier family 2 (facilitated glucose transporter), member 3
UP in Hypoxia	**114548**	NLRP3	1.209772	0.8739676	2.3130107	NLR family, pyrin domain containing 3
UP in Hypoxia	**3557**	IL1RN	2.091598	0.9310848	4.2621996	interleukin 1 receptor antagonist
UP in Hypoxia	**203259**	FAM219	1.643731	0.917782	3.1247289	family with sequence similarity 219,
UP in Hypoxia	**3139**	HLA-L	2.301509	0.8664517	4.9297297	major histocompatibility complex, class I, L (pseudogene)
UP in Hypoxia	**8731**	RNMT	1.185863	0.8852101	2.274994	RNA (guanine-7-) methyltransferase
UP in Hypoxia	**7378**	UPP1	1.15686	0.8870286	2.2297158	uridine phosphorylase 1
UP in Hypoxia	**5032**	P2RY11	4.933505	0.9847801	30.558559	purinergic receptor P2Y, G-protein coupled,
UP in Hypoxia	**9783**	RIMS3	1.749563	0.9209979	3.3625661	regulating synaptic membrane exocytosis 3

UP in Hypoxia	**54814**	QPCTL	1.046988	0.8789394	2.0662119	glutaminyl-peptide cyclotransferase-like
UP in Hypoxia	**100529211**	C17orf61-PLSCR3	2.535172	0.8439488	5.7964602	C17orf61-PLSCR3 readthrough
UP in Hypoxia	**1831**	TSC22D3	2.830136	0.9621249	7.1114114	TSC22 domain family, member 3
UP in Hypoxia	**6720**	SREBF1	1.2957	0.8878348	2.4549613	sterol regulatory element binding transcription factor 1
UP in Hypoxia	**5915**	RARB	1.206671	0.8779181	2.3080448	retinoic acid receptor, beta
UP in Hypoxia	**11000**	SLC27A3	1.723029	0.9084923	3.3012876	solute carrier family 27 (fatty acid transporter), member 3
UP in Hypoxia	**728495**	FAM74A	6.857981	0.8206665	116	family with sequence similarity 74, member
UP in Hypoxia	**6876**	TAGLN	1.436938	0.8451044	2.707457	transgelin
UP in Hypoxia	**79001**	VKORC1	1.814456	0.9234614	3.5172688	vitamin K epoxide reductase complex,
UP in Hypoxia	**100506211**	MIR210HG	2.513137	0.94583	5.7085987	MIR210 host gene (non-protein coding)
UP in Hypoxia	**728537**	LOC728537	4.122522	0.9323121	17.418182	uncharacterized LOC728537
UP in Hypoxia	**9094**	UNC119	1.334881	0.8953865	2.5225464	unc-119 homolog (C. elegans)
UP in Hypoxia	**3037**	HAS2	1.873785	0.9168055	3.6649283	hyaluronan synthase 2
UP in Hypoxia	**439921**	MXRA7	1.121634	0.8866344	2.1759326	matrix-remodelling associated 7
UP in Hypoxia	**4170**	MCL1	1.414172	0.8993102	2.6650676	myeloid cell leukemia sequence 1 (BCL2-
UP in Hypoxia	**3725**	JUN	1.53856	0.9039058	2.9050445	jun proto-oncogene
UP in Hypoxia	**388**	RHOB	1.090327	0.8843411	2.1292223	ras homolog family member B
UP in Hypoxia	**9180**	OSMR	1.379803	0.897008	2.6023281	oncostatin M receptor
UP in Hypoxia	**51339**	DACT1	2.090112	0.9266505	4.2578125	dapper, antagonist of beta-catenin, homolog 1 (Xenopus laevis)
UP in Hypoxia	**220965**	FAM13C	2.774915	0.9305653	6.844358	family with sequence similarity 13, member
UP in Hypoxia	**401944**	LDLRAD2	2.245366	0.8921168	4.741573	low density lipoprotein receptor class A domain containing 2
UP in Hypoxia	**79899**	PRR5L	1.94398	0.8966676	3.8476562	proline rich 5 like
UP in Hypoxia	**55845**	BRK1	1.018543	0.8813581	2.0258719	BRICK1, SCAR/WAVE actin-nucleating complex subunit
UP in Hypoxia	**1382**	CRABP2	2.854588	0.9627788	7.2329678	cellular retinoic acid binding protein 2
UP in Hypoxia	**255394**	TCP11L2	1.613391	0.8769775	3.0597015	t-complex 11, testis-specific-like 2
UP in Hypoxia	**84457**	PHYHIPL	3.443686	0.8921526	10.880597	phytanoyl-CoA 2-hydroxylase interacting protein-like
UP in Hypoxia	**26471**	NUPR1	1.267791	0.8932545	2.4079262	nuclear protein, transcriptional regulator, 1
UP in Hypoxia	**9976**	CLEC2B	3.540881	0.9771388	11.638889	C-type lectin domain family 2, member B
UP in Hypoxia	**4082**	MARCKS	1.943222	0.9245543	3.8456342	myristoylated alanine-rich protein kinase C substrate
UP in Hypoxia	**1742**	DLG4	1.135832	0.882648	2.1974522	discs, large homolog 4 (Drosophila)
UP in Hypoxia	**774**	CACNA1B	7.392317	0.9176028	168	calcium channel, voltage-dependent, N type, alpha 1B subunit
UP in Hypoxia	**126014**	OSCAR	1.595207	0.8588999	3.0213777	osteoclast associated, immunoglobulin-like receptor
UP in Hypoxia	**5210**	PFKFB4	1.944612	0.9245991	3.8493434	6-phosphofructo-2-kinase/fructose-2,6-biphosphatase 4
UP in Hypoxia	**8740**	TNFSF14	2.37223	0.9442175	5.1774065	tumor necrosis factor (ligand) superfamily, member 14
UP in Hypoxia	**639**	PRDM1	1.061507	0.8571083	2.0871105	PR domain containing 1, with ZNF domain
UP in Hypoxia	**8372**	HYAL3	1.598399	0.9069963	3.0280702	hyaluronoglucosaminidase 3
UP in Hypoxia	**3106**	HLA-B	1.854788	0.9239093	3.6169853	major histocompatibility complex, class I, B
UP in Hypoxia	**100505806**	LOC100505806	1.06345	0.8713966	2.0899237	uncharacterized LOC100505806
UP in Hypoxia	**84771**	DDX11L2	1.35124	0.8133925	2.5513126	DEAD/H (Asp-Glu-Ala-Asp/His) box helicase 11 like 2
UP in Hypoxia	**4319**	MMP10	3.545703	0.9524142	11.677852	matrix metallopeptidase 10 (stromelysin 2)

UP in Hypoxia	**4784**	NFIX	1.685424	0.9150408	3.2163501	nuclear factor I/X (CCAAT-binding transcription factor)
UP in Hypoxia	**2745**	GLRX	2.138204	0.9384664	4.4021377	glutaredoxin (thioltransferase)
UP in Hypoxia	**3552**	IL1A	4.370498	0.9878617	20.684783	interleukin 1, alpha
UP in Hypoxia	**4582**	MUC1	4.092649	0.9829526	17.061224	mucin 1, cell surface associated
UP in Hypoxia	**5097**	PCDH1	4.005294	0.8717191	16.058824	protocadherin 1
UP in Hypoxia	**54550**	NECAB2	2.61548	0.9387709	6.1282723	N-terminal EF-hand calcium binding protein
UP in Hypoxia	**55784**	MCTP2	1.287783	0.8861328	2.4415265	multiple C2 domains, transmembrane 2
UP in Hypoxia	**1112**	FOXN3	1.756385	0.9144137	3.3785047	forkhead box N3
UP in Hypoxia	**51129**	ANGPTL	5.599436	0.9969453	48.483978	angiopoietin-like 4
UP in Hypoxia	**7159**	TP53BP2	1.092788	0.882003	2.132858	tumor protein p53 binding protein, 2
UP in Hypoxia	**8324**	FZD7	1.825845	0.8960584	3.5451448	frizzled family receptor 7
UP in Hypoxia	**353322**	ANKRD3	3.4755	0.9760459	11.1232	ankyrin repeat domain 37
UP in Hypoxia	**100505933**	LOC1005 05933	2.496832	0.8040401	5.6444444	uncharacterized LOC100505933
UP in Hypoxia	**284018**	C17orf58	1.294199	0.8814297	2.4524083	chromosome 17 open reading frame 58
UP in Hypoxia	**255488**	RNF144	3.208919	0.8780794	9.2465754	ring finger protein 144B
UP in Hypoxia	**347148**	QRFP	2.348728	0.8383678	5.09375	pyroglutamylated RFamide peptide
UP in Hypoxia	**4783**	NFIL3	1.634263	0.9161516	3.1042887	nuclear factor, interleukin 3 regulated
UP in Hypoxia	**150864**	FAM117	2.859189	0.9535967	7.2560748	family with sequence similarity 117,
UP in Hypoxia	**24145**	PANX1	1.117967	0.8791096	2.1704088	pannexin 1
UP in Hypoxia	**387856**	C12orf68	1.936548	0.9122189	3.8278867	chromosome 12 open reading frame 68
UP in Hypoxia	**114038**	LINC003	2.873338	0.9212309	7.3275862	long intergenic non-protein coding RNA
UP in Hypoxia	**7039**	TGFA	1.184455	0.8876198	2.2727746	transforming growth factor, alpha
UP in Hypoxia	**400027**	LOC4000 27	1.271641	0.8697214	2.4143599	uncharacterized LOC400027
UP in Hypoxia	**8337**	HIST2H2 AA3	1.59303	0.9141181	3.0168224	histone cluster 2, H2aa3
UP in Hypoxia	**901**	CCNG2	2.389042	0.9381707	5.2380952	cyclin G2
UP in Hypoxia	**90271**	LINC002	2.667425	0.9442892	6.3529412	long intergenic non-protein coding RNA
UP in Hypoxia	**5066**	PAM	1.206624	0.8904506	2.3079689	peptidylglycine alpha-amidating monooxygenase
UP in Hypoxia	**1820**	ARID3A	1.222525	0.8752307	2.333548	AT rich interactive domain 3A (BRIGHT-
UP in Hypoxia	**285966**	FAM115	2.69279	0.9530682	6.4656279	family with sequence similarity 115,
UP in Hypoxia	**2615**	LRRC32	3.9545	0.969372	15.503268	leucine rich repeat containing 32
UP in Hypoxia	**339044**	PKD1P1	1.118171	0.8850578	2.1707166	polycystic kidney disease 1 (autosomal dominant) pseudogene 1
UP in Hypoxia	**100505695**	LOC1005 05695	1.034403	0.8044164	2.0482655	uncharacterized LOC100505695
UP in Hypoxia	**140885**	SIRPA	2.705471	0.9558362	6.5227066	signal-regulatory protein alpha
UP in Hypoxia	**1437**	CSF2	1.361301	0.894437	2.5691672	colony stimulating factor 2 (granulocyte-macrophage)
UP in Hypoxia	**23526**	HMHA1	1.723026	0.891857	3.3012821	histocompatibility (minor) HA-1
UP in Hypoxia	**100506696**	KDM5B-AS1	2.851181	0.9467079	7.2159091	KDM5B antisense RNA 1 (head to head)
UP in Hypoxia	**7097**	TLR2	2.697934	0.8844218	6.4887218	toll-like receptor 2
UP in Hypoxia	**55647**	RAB20	1.301725	0.8906477	2.4652354	RAB20, member RAS oncogene family
UP in Hypoxia	**3576**	IL8	1.99785	0.9368808	3.9940422	interleukin 8
UP in Hypoxia	**624**	BDKRB2	1.985208	0.9226015	3.9591985	bradykinin receptor B2
UP in Hypoxia	**26152**	ZNF337	1.56707	0.8959778	2.9630234	zinc finger protein 337
UP in Hypoxia	**2138**	EYA1	1.23665	0.8240885	2.3565062	eyes absent homolog 1 (Drosophila)
UP in Hypoxia	**79656**	BEND5	5.564149	0.9252531	47.3125	BEN domain containing 5
UP in Hypoxia	**2632**	GBE1	2.152338	0.9383768	4.4454762	glucan (1,4-alpha-), branching enzyme 1
UP in Hypoxia	**6351**	CCL4	2.07447	0.8794231	4.2118959	chemokine (C-C motif) ligand 4
UP in Hypoxia	**150094**	SIK1	1.278723	0.891006	2.4262419	salt-inducible kinase 1
UP in Hypoxia	**164091**	PAQR7	1.17602	0.8791723	2.2595258	progestin and adipoQ receptor family member VII

UP in Hypoxia	**1292**	COL6A2	1.653726	0.9195198	3.1464517	collagen, type VI, alpha 2
UP in Hypoxia	**3987**	LIMS1	2.492843	0.9502105	5.6288594	LIM and senescent cell antigen-like domains
UP in Hypoxia	**5209**	PFKFB3	1.939532	0.9242766	3.8358116	6-phosphofructo-2-kinase/fructose-2,6-biphosphatase 3
UP in Hypoxia	**6349**	CCL3L1	4.041243	0.9673385	16.464	chemokine (C-C motif) ligand 3-like 1
UP in Hypoxia	**8292**	COLQ	4.893085	0.900636	29.714286	collagen-like tail subunit (single strand of homotrimer) of asymmetric acetylcholinesterase
UP in Hypoxia	**205**	AK4	1.707832	0.9184897	3.2666953	adenylate kinase 4
UP in Hypoxia	**79644**	SRD5A3	2.285539	0.9346591	4.8754606	steroid 5 alpha-reductase 3
UP in Hypoxia	**8013**	NR4A3	2.454363	0.9227985	5.4807122	nuclear receptor subfamily 4, group A,
UP in Hypoxia	**96626**	LIMS3	10.6254	0.9937382	1579.6667	LIM and senescent cell antigen-like domains
UP in Hypoxia	**550112**	LOC550112	1.25762	0.8696945	2.3910093	uncharacterized LOC550112
UP in Hypoxia	**400533**	FLJ26245	2.743712	0.8518588	6.6979167	uncharacterized LOC400533
UP in Hypoxia	**10957**	PNRC1	1.151885	0.8875302	2.2220397	proline-rich nuclear receptor coactivator 1
UP in Hypoxia	**3757**	KCNH2	11.41416	0.9968557	2729	potassium voltage-gated channel, subfamily H (eag-related), member 2
UP in Hypoxia	**6619**	SNAPC3	1.24764	0.8837409	2.3745271	small nuclear RNA activating complex, polypeptide 3, 50kDa
UP in Hypoxia	**84557**	MAP1LC3A	1.144449	0.8855505	2.2106166	microtubule-associated protein 1 light chain 3 alpha
UP in Hypoxia	**79845**	RNF122	2.288593	0.9097554	4.8857939	ring finger protein 122
UP in Hypoxia	**4351**	MPI	1.107913	0.8830153	2.1553358	mannose phosphate isomerase
UP in Hypoxia	**51400**	PPME1	1.098564	0.8817074	2.1414141	protein phosphatase methylesterase 1
UP in Hypoxia	**375607**	NAT16	2.347271	0.8999104	5.0886076	N-acetyltransferase 16 (GCN5-related,
UP in Hypoxia	**91355**	LRP5L	1.033312	0.8201827	2.0467172	low density lipoprotein receptor-related protein 5-like
UP in Hypoxia	**57631**	LRCH2	1.080282	0.8177999	2.1144492	leucine-rich repeats and calponin homology (CH) domain containing 2
UP in Hypoxia	**55504**	TNFRSF19	1.419158	0.865099	2.6742934	tumor necrosis factor receptor superfamily, member 19
UP in Hypoxia	**6273**	S100A2	1.322542	0.8887933	2.5010634	S100 calcium binding protein A2
UP in Hypoxia	**10628**	TXNIP	2.230113	0.939398	4.6917061	thioredoxin interacting protein
UP in Hypoxia	**22883**	CLSTN1	1.358371	0.8973305	2.5639556	calsyntenin 1
UP in Hypoxia	**51114**	ZDHHC9	1.579818	0.9155155	2.9893223	zinc finger, DHHC-type containing 9
UP in Hypoxia	**768**	CA9	3.391974	0.9750873	10.497502	carbonic anhydrase IX
UP in Hypoxia	**1415**	CRYBB2	2.142575	0.8159097	4.415493	crystallin, beta B2
UP in Hypoxia	**54206**	ERRFI1	4.324622	0.9900833	20.037386	ERBB receptor feedback inhibitor 1
UP in Hypoxia	**4803**	NGF	2.379335	0.9267312	5.2029703	nerve growth factor (beta polypeptide)
UP in Hypoxia	**7138**	TNNT1	1.197555	0.8904416	2.2935064	troponin T type 1 (skeletal, slow)
UP in Hypoxia	**84223**	IQCG	1.130579	0.8829437	2.1894661	IQ motif containing G
UP in Hypoxia	**338785**	KRT79	1.625887	0.9103288	3.0863183	keratin 79
UP in Hypoxia	**5325**	PLAGL1	1.32464	0.8891785	2.5047035	pleiomorphic adenoma gene-like 1
UP in Hypoxia	**10673**	TNFSF13B	3.807161	0.9798173	13.99812	tumor necrosis factor (ligand) superfamily, member 13b
UP in Hypoxia	**91653**	BOC	2.014862	0.8223506	4.0414201	BOC cell adhesion associated, oncogene regulated
UP in Hypoxia	**3697**	ITIH1	8.696968	0.9644182	415	inter-alpha-trypsin inhibitor heavy chain 1
UP in Hypoxia	**1466**	CSRP2	2.127628	0.9335662	4.3699825	cysteine and glycine-rich protein 2
UP in Hypoxia	**694**	BTG1	2.47958	0.948392	5.5773501	B-cell translocation gene 1, anti-
UP in Hypoxia	**3201**	HOXA4	2.230527	0.9242318	4.6930533	homeobox A4
UP in Hypoxia	**664**	BNIP3	2.791872	0.9579772	6.9252799	BCL2/adenovirus E1B 19kDa interacting protein 3
UP in Hypoxia	**84102**	SLC41A2	1.59192	0.9063603	3.0145033	solute carrier family 41, member 2
UP in Hypoxia	**219670**	ENKUR	2.217591	0.8124787	4.6511628	enkurin, TRPC channel interacting protein

UP in Hypoxia	**54084**	TSPEAR	6.61471	0.8762161	97.999998	thrombospondin-type laminin G domain and EAR repeats
UP in Hypoxia	**10865**	ARID5A	1.633023	0.9050614	3.1016225	AT rich interactive domain 5A (MRF1-like)
UP in Hypoxia	**79816**	TLE6	2.607683	0.8711995	6.0952381	transducin-like enhancer of split 6 (E(sp1) homolog, Drosophila)
UP in Hypoxia	**5143**	PDE4C	2.154061	0.9269282	4.4507898	phosphodiesterase 4C, cAMP-specific
UP in Hypoxia	**219699**	UNC5B	1.223703	0.8775329	2.335454	unc-5 homolog B (C. elegans)
UP in Hypoxia	**50944**	SHANK1	1.075996	0.8516617	2.1081772	SH3 and multiple ankyrin repeat domains 1
UP in Hypoxia	**26297**	SERGEF	1.103173	0.8692735	2.1482667	secretion regulating guanine nucleotide exchange factor
UP in Hypoxia	**133**	ADM	3.059791	0.9676521	8.3385159	adrenomedullin
UP in Hypoxia	**8843**	HCAR3	2.66862	0.8828003	6.358209	hydroxycarboxylic acid receptor 3
UP in Hypoxia	**79957**	PAQR6	2.053349	0.8065574	4.1506849	progestin and adipoQ receptor family
UP in Hypoxia	**8140**	SLC7A5	1.693316	0.9168055	3.2339916	solute carrier family 7 (amino acid transporter light chain, L system), member 5
UP in Hypoxia	**6535**	SLC6A8	2.742221	0.9578339	6.6909982	solute carrier family 6 (neurotransmitter transporter, creatine), member 8
UP in Hypoxia	**23480**	SEC61G	1.260301	0.8938368	2.3954572	Sec61 gamma subunit
UP in Hypoxia	**728233**	PI4KAP1	1.0017	0.8211771	2.0023585	phosphatidylinositol 4-kinase, catalytic, alpha pseudogene 1
UP in Hypoxia	**27106**	ARRDC2	1.142305	0.8789483	2.2073343	arrestin domain containing 2
UP in Hypoxia	**100131213**	ZNF503-AS2	1.00305	0.8570456	2.0042322	ZNF503 antisense RNA 2
UP in Hypoxia	**283174**	LOC283174	2.770116	0.9505868	6.8216292	uncharacterized LOC283174
UP in Hypoxia	**5742**	PTGS1	5.957772	0.9321956	62.153847	prostaglandin-endoperoxide synthase 1 (prostaglandin G/H synthase and cyclooxygenase)
UP in Hypoxia	**7422**	VEGFA	1.614512	0.9185255	3.0620793	vascular endothelial growth factor A
UP in Hypoxia	**131544**	CRYBG3	1.767068	0.9159724	3.4036145	beta-gamma crystallin domain containing 3
UP in Hypoxia	**114088**	TRIM9	2.059511	0.8996596	4.1684492	tripartite motif containing 9
UP in Hypoxia	**5365**	PLXNB3	2.211764	0.9358864	4.6324148	plexin B3
UP in Hypoxia	**3914**	LAMB3	1.860058	0.922055	3.6302222	laminin, beta 3
UP in Hypoxia	**3726**	JUNB	1.501642	0.9021679	2.8316474	jun B proto-oncogene
UP in Hypoxia	**6926**	TBX3	1.937979	0.9247514	3.8316863	T-box 3
UP in Hypoxia	**55366**	LGR4	1.101262	0.8764669	2.1454231	leucine-rich repeat containing G protein-coupled receptor 4
UP in Hypoxia	**2642**	GCGR	1.516144	0.8747559	2.8602564	glucagon receptor
UP in Hypoxia	**64412**	GZF1	1.083465	0.8567052	2.1191199	GDNF-inducible zinc finger protein 1
UP in Hypoxia	**26018**	LRIG1	2.264254	0.9388247	4.8040585	leucine-rich repeats and immunoglobulin-like domains 1
UP in Hypoxia	**6617**	SNAPC1	1.439316	0.8998388	2.7119228	small nuclear RNA activating complex, polypeptide 1, 43kDa
UP in Hypoxia	**5836**	PYGL	2.242635	0.9326973	4.7326069	phosphorylase, glycogen, liver
UP in Hypoxia	**285512**	FAM13A-AS1	2.379828	0.9199946	5.2047478	FAM13A antisense RNA 1
UP in Hypoxia	**8819**	SAP30	1.33112	0.8928962	2.5159794	Sin3A-associated protein, 30kDa
UP in Hypoxia	**80270**	HSD3B7	1.022092	0.8742094	2.0308611	hydroxy-delta-5-steroid dehydrogenase, 3 beta- and steroid delta-isomerase 7
UP in Hypoxia	**1947**	EFNB1	1.196975	0.8052943	2.2925852	ephrin-B1
UP in Hypoxia	**25**	ABL1	1.178715	0.8877363	2.26375	c-abl oncogene 1, non-receptor tyrosine
UP in Hypoxia	**5211**	PFKL	1.317512	0.8968915	2.4923586	phosphofructokinase, liver
UP in Hypoxia	**1052**	CEBPD	1.564133	0.8919287	2.9569971	CCAAT/enhancer binding protein (C/EBP),
UP in Hypoxia	**290**	ANPEP	5.703211	0.9508376	52.1	alanyl (membrane) aminopeptidase
UP in Hypoxia	**55779**	WDR52	2.182572	0.8870823	4.5396226	WD repeat domain 52
UP in Hypoxia	**339751**	MLK7-AS1	2.172617	0.8777121	4.5084034	MLK7 antisense RNA 1

UP in Hypoxia	**26118**	WSB1	1.979805	0.9253068	3.9443966	WD repeat and SOCS box containing 1
UP in Hypoxia	**7025**	NR2F1	1.06535	0.8796918	2.0926769	nuclear receptor subfamily 2, group F,
UP in Hypoxia	**90226**	UCN2	1.956153	0.9236675	3.8802581	urocortin 2
UP in Hypoxia	**112399**	EGLN3	7.562242	0.9476933	189	egl nine homolog 3 (C. elegans)
UP in Hypoxia	**441054**	C4orf47	3.906203	0.9665681	14.992857	chromosome 4 open reading frame 47
UP in Hypoxia	**575**	BAI1	6.696968	0.8503807	103.75	brain-specific angiogenesis inhibitor 1
UP in Hypoxia	**10809**	STARD10	1.042894	0.8150139	2.0603567	StAR-related lipid transfer (START) domain containing 10
UP in Hypoxia	**386757**	SLC6A10P	2.313158	0.8863478	4.969697	solute carrier family 6 (neurotransmitter transporter, creatine), member 10, pseudogene
UP in Hypoxia	**11009**	IL24	2.938599	0.9540715	7.6666667	interleukin 24
UP in Hypoxia	**3569**	IL6	3.130948	0.9677864	8.7601032	interleukin 6 (interferon, beta 2)
UP in Hypoxia	**923**	CD6	3.191486	0.9494939	9.135514	CD6 molecule
UP in Hypoxia	**57528**	KCTD16	1.757971	0.8139299	3.3822222	potassium channel tetramerisation domain containing 16
UP in Hypoxia	**100532724**	NPHP3-ACAD11	1.243354	0.8020066	2.3674833	NPHP3-ACAD11 readthrough
UP in Hypoxia	**3897**	L1CAM	5.960829	0.8558362	62.285715	L1 cell adhesion molecule
UP in Hypoxia	**122481**	AK7	1.968636	0.8191615	3.9139785	adenylate kinase 7
UP in Hypoxia	**10603**	SH2B2	1.414724	0.8801845	2.666087	SH2B adaptor protein 2
UP in Hypoxia	**2330**	FMO5	2.316672	0.8111798	4.9818182	flavin containing monooxygenase 5
UP in Hypoxia	**23516**	SLC39A14	1.524774	0.9033593	2.8774163	solute carrier family 39 (zinc transporter), member 14
UP in Hypoxia	**348013**	TMEM255B	1.44984	0.8923856	2.7317784	transmembrane protein 255B
UP in Hypoxia	**55768**	NGLY1	1.655181	0.9171907	3.1496276	N-glycanase 1
UP in Hypoxia	**9284**	NPIP	1.040689	0.8795485	2.0572096	nuclear pore complex interacting protein
UP in Hypoxia	**360132**	FKBP9L	2.248113	0.9131685	4.7506112	FK506 binding protein 9-like
UP in Hypoxia	**23635**	SSBP2	1.056504	0.8140285	2.0798859	single-stranded DNA binding protein 2
UP in Hypoxia	**3486**	IGFBP3	6.950393	0.9994088	123.67351	insulin-like growth factor binding protein 3
UP in Hypoxia	**5743**	PTGS2	1.636685	0.9189376	3.1095047	prostaglandin-endoperoxide synthase 2 (prostaglandin G/H synthase and cyclooxygenase)
UP in Hypoxia	**222962**	SLC29A4	3.071152	0.9439219	8.4044444	solute carrier family 29 (nucleoside transporters), member 4
UP in Hypoxia	**84657**	LINC008	1.660224	0.8364239	3.1606557	long intergenic non-protein coding RNA
UP in Hypoxia	**8994**	LIMD1	1.302469	0.8919735	2.4665067	LIM domains containing 1
UP in Hypoxia	**4355**	MPP2	1.484206	0.8958882	2.7976323	membrane protein, palmitoylated 2 (MAGUK p55 subfamily member 2)
UP in Hypoxia	**23523**	CABIN1	1.055518	0.8738332	2.0784641	calcineurin binding protein 1
UP in Hypoxia	**100131691**	LOC100131691	1.570918	0.8840545	2.9709365	uncharacterized LOC100131691
UP in Hypoxia	**151011**	SEPT10	1.279251	0.8934874	2.4271297	septin 10
UP in Hypoxia	**140901**	STK35	1.522888	0.8924214	2.8736576	serine/threonine kinase 35
UP in Hypoxia	**100507135**	SPG20O	1.729146	0.8064768	3.3153153	SPG20 opposite strand
UP in Hypoxia	**2826**	CCR10	1.559666	0.8809729	2.9478563	chemokine (C-C motif) receptor 10
UP in Hypoxia	**57571**	CARNS1	3.0182	0.9131148	8.1015625	carnosine synthase 1
UP in Hypoxia	**3299**	HSF4	1.29207	0.8600645	2.4487917	heat shock transcription factor 4
UP in Hypoxia	**339105**	PRSS53	2.899432	0.9592224	7.461326	protease, serine, 53
UP in Hypoxia	**5792**	PTPRF	5.228819	0.8800412	37.5	protein tyrosine phosphatase, receptor type,
UP in Hypoxia	**11067**	C10orf10	4.429742	0.9906477	21.551881	chromosome 10 open reading frame 10
UP in Hypoxia	**5033**	P4HA1	2.354348	0.9493237	5.1136313	prolyl 4-hydroxylase, alpha polypeptide I
UP in Hypoxia	**100526842**	RPL17-C18orf32	1.87629	0.8252889	3.6712963	RPL17-C18orf32 readthrough
UP in Hypoxia	**266675**	BEST4	2.953502	0.8302069	7.7462687	bestrophin 4
UP in Hypoxia	**7128**	TNFAIP3	2.36995	0.9483741	5.1692308	tumor necrosis factor, alpha-induced protein

UP in Hypoxia	**100288615**	WHAMMP1	1.399636	0.8677864	2.6383495	WAS protein homolog associated with actin, golgi membranes and microtubules pseudogene 1
UP in Hypoxia	**81606**	LBH	2.900464	0.8046224	7.4666667	limb bud and heart development
UP in Hypoxia	**26040**	SETBP1	1.788547	0.9016304	3.4546685	SET binding protein 1
UP in Hypoxia	**167359**	NIM1	2.418899	0.9382603	5.3476263	serine/threonine-protein kinase NIM1
UP in Hypoxia	**51428**	DDX41	1.777579	0.9219744	3.4285044	DEAD (Asp-Glu-Ala-Asp) box polypeptide
UP in Hypoxia	**81855**	SFXN3	1.175229	0.8882021	2.2582873	sideroflexin 3
UP in Hypoxia	**1410**	CRYAB	1.69381	0.8394159	3.2350993	crystallin, alpha B
UP in Hypoxia	**118788**	PIK3AP1	5.960829	0.8558362	62.285715	phosphoinositide-3-kinase adaptor protein 1
UP in Hypoxia	**79955**	PDZD7	1.4975	0.8450417	2.8235294	PDZ domain containing 7
UP in Hypoxia	**100529264**	RAB4B-EGLN2	7.716534	0.9108842	210.33333	RAB4B-EGLN2 readthrough (non-protein coding)
UP in Hypoxia	**5794**	PTPRH	2.192851	0.928899	4.5720824	protein tyrosine phosphatase, receptor type,
UP in Hypoxia	**146779**	EFCAB3	6.114868	0.9958613	69.304054	EF-hand calcium binding domain 3
UP in Hypoxia	**4601**	MXI1	2.593194	0.9565529	6.0343337	MAX interactor 1, dimerization protein
UP in Hypoxia	**80853**	JHDM1D	1.909317	0.9086446	3.7563131	jumonji C domain containing histone demethylase 1 homolog D (S. cerevisiae)
UP in Hypoxia	**1808**	DPYSL2	1.287151	0.8938637	2.4404568	dihydropyrimidinase-like 2
UP in Hypoxia	**23152**	CIC	1.385362	0.8963361	2.6123749	capicua transcriptional repressor
UP in Hypoxia	**54435**	HCG4	1.674789	0.8264624	3.1927273	HLA complex group 4 (non-protein coding)
UP in Hypoxia	**148898**	C1orf213	1.234657	0.8464033	2.3532537	chromosome 1 open reading frame 213
UP in Hypoxia	**100506060**	LOC100506060	1.383789	0.8904685	2.6095281	SMG1 homolog, phosphatidylinositol 3-kinase-related kinase (C. elegans) pseudogene
UP in Hypoxia	**4501**	MT1X	3.488347	0.9778375	11.222695	metallothionein 1X
UP in Hypoxia	**1816**	DRD5	2.025391	0.8940966	4.0710227	dopamine receptor D5
UP in Hypoxia	**5310**	PKD1	1.18552	0.8818866	2.2744536	polycystic kidney disease 1 (autosomal dominant)
UP in Hypoxia	**79660**	PPP1R3B	2.048029	0.9323211	4.1354051	protein phosphatase 1, regulatory subunit 3B
UP in Hypoxia	**6676**	SPAG4	2.685398	0.9559169	6.4325843	sperm associated antigen 4
UP in Hypoxia	**10144**	FAM13A	2.34659	0.9437427	5.0862069	family with sequence similarity 13, member
UP in Hypoxia	**272**	AMPD3	2.448462	0.9495297	5.4583402	adenosine monophosphate deaminase 3
UP in Hypoxia	**134265**	AFAP1L	1.951745	0.8882379	3.8684211	actin filament associated protein 1-like 1
UP in Hypoxia	**3638**	INSIG1	1.699414	0.9203619	3.2476912	insulin induced gene 1
UP in Hypoxia	**10656**	KHDRBS3	2.358632	0.9451581	5.1288385	KH domain containing, RNA binding, signal transduction associated 3
UP in Hypoxia	**1944**	EFNA3	3.742006	0.971943	13.38	ephrin-A3
UP in Hypoxia	**23613**	ZMYND	1.185052	0.8875392	2.2737151	zinc finger, MYND-type containing 8
UP in Hypoxia	**100188947**	LOC100188947	1.278693	0.8006092	2.4261905	uncharacterized LOC100188947
UP in Hypoxia	**57221**	KIAA124	2.200441	0.9121383	4.5961995	KIAA1244
UP in Hypoxia	**11187**	PKP3	1.765212	0.8339067	3.3992395	plakophilin 3
UP in Hypoxia	**284836**	LINC003	2.981315	0.8350712	7.8970588	long intergenic non-protein coding RNA
UP in Hypoxia	**100529145**	TEN1-CDK3	1.346248	0.8077309	2.5425	TEN1-CDK3 readthrough (non-protein coding)
UP in Hypoxia	**4081**	MAB21L	1.875706	0.8646152	3.6698113	mab-21-like 1 (C. elegans)
UP in Hypoxia	**114134**	SLC2A13	1.960127	0.9143868	3.8909627	solute carrier family 2 (facilitated glucose transporter), member 13
UP in Hypoxia	**55893**	ZNF395	3.142135	0.967231	8.8282954	zinc finger protein 395
UP in Hypoxia	**284942**	RPL23AP82	1.725937	0.9198692	3.3079484	ribosomal protein L23a pseudogene 82
UP in Hypoxia	**6319**	SCD	2.089621	0.9370689	4.2563627	stearoyl-CoA desaturase (delta-9-desaturase)
UP in Hypoxia	**644242**	LINC006	2.16932	0.8859894	4.4981132	long intergenic non-protein coding RNA
UP in Hypoxia	**84957**	RELT	1.347336	0.8802293	2.5444191	RELT tumor necrosis factor receptor
UP in Hypoxia	**54583**	EGLN1	2.005935	0.9343994	4.0164904	egl nine homolog 1 (C. elegans)
UP in Hypoxia	**157313**	CDCA2	1.536471	0.9008779	2.9008415	cell division cycle associated 2

UP in Hypoxia	**222950**	NYAP1	2.754419	0.9451133	6.747807	neuronal tyrosine-phosphorylated phosphoinositide-3-kinase adaptor 1
UP in Hypoxia	**1181**	CLCN2	1.798763	0.9045687	3.4792176	chloride channel, voltage-sensitive 2
UP in Hypoxia	**4311**	MME	1.142104	0.8853355	2.2070267	membrane metallo-endopeptidase
UP in Hypoxia	**100506178**	LOC1005 06178	2.168471	0.9300994	4.4954683	uncharacterized LOC100506178
UP in Hypoxia	**55603**	FAM46A	1.112905	0.861023	2.1628075	family with sequence similarity 46, member
UP in Hypoxia	**134429**	STARD4	1.05393	0.8570725	2.0761773	StAR-related lipid transfer (START) domain containing 4
UP in Hypoxia	**6666**	SOX12	1.045817	0.8615247	2.0645356	SRY (sex determining region Y)-box 12
UP in Hypoxia	**91748**	ELMSA	1.04804	0.8660575	2.0677189	ELM2 and Myb/SANT-like domain
UP in Hypoxia	**23148**	NACAD	1.060483	0.8421034	2.0856299	NAC alpha domain containing
UP in Hypoxia	**204**	AK2	1.337959	0.8973126	2.5279341	adenylate kinase 2
UP in Hypoxia	**10670**	RRAGA	1.835051	0.92356	3.5678402	Ras-related GTP binding A
UP in Hypoxia	**6364**	CCL20	3.849428	0.9832124	14.414293	chemokine (C-C motif) ligand 20
UP in Hypoxia	**100506627**	DCDC5	4.209891	0.9623757	18.505618	doublecortin domain containing 5
UP in Hypoxia	**29950**	SERTAD	1.070873	0.8798531	2.1007046	SERTA domain containing 1
UP in Hypoxia	**10874**	NMU	3.878562	0.899776	14.708333	neuromedin U
UP in Hypoxia	**79854**	LINC001	1.244901	0.8546896	2.3700234	long intergenic non-protein coding RNA
UP in Hypoxia	**100505783**	LOC1005 05783	1.248616	0.8391024	2.3761329	uncharacterized LOC100505783
UP in Hypoxia	**170690**	ADAMT S16	1.366716	0.821625	2.5788288	ADAM metallopeptidase with thrombospondin type 1 motif, 16
UP in Hypoxia	**115330**	GPR146	4.432845	0.9844934	21.598291	G protein-coupled receptor 146
UP in Hypoxia	**2752**	GLUL	2.759397	0.9576458	6.7711313	glutamate-ammonia ligase
UP in Hypoxia	**5266**	PI3	4.205892	0.9884888	18.454391	peptidase inhibitor 3, skin-derived
UP in Hypoxia	**6518**	SLC2A5	4.805113	0.9037983	27.956522	solute carrier family 2 (facilitated glucose/fructose transporter), member 5
UP in Hypoxia	**8574**	AKR7A2	1.803961	0.9216071	3.4917765	aldo-keto reductase family 7, member A2 (aflatoxin aldehyde reductase)
UP in Hypoxia	**7436**	VLDLR	2.03714	0.9356625	4.1043099	very low density lipoprotein receptor
UP in Hypoxia	**971**	CD72	1.444696	0.8005733	2.7220544	CD72 molecule
UP in Hypoxia	**113157**	RPLP0P2	2.140321	0.9296067	4.4086021	ribosomal protein, large, P0 pseudogene 2
UP in Hypoxia	**7015**	TERT	2.17673	0.856096	4.5212766	telomerase reverse transcriptase
UP in Hypoxia	**2152**	F3	4.01695	0.9864284	16.189088	coagulation factor III (thromboplastin, tissue factor)
UP in Hypoxia	**57561**	ARRDC3	2.001141	0.933835	4.0031657	arrestin domain containing 3
UP in Hypoxia	**9379**	NRXN2	1.280895	0.886724	2.4298965	neurexin 2
UP in Hypoxia	**89886**	SLAMF9	5.284704	0.9776494	38.981132	SLAM family member 9
UP in Hypoxia	**6478**	SIAH2	1.147208	0.875589	2.2148492	siah E3 ubiquitin protein ligase 2
UP in Hypoxia	**84445**	LZTS2	1.023527	0.88031	2.0328832	leucine zipper, putative tumor suppressor 2
UP in Hypoxia	**65124**	SOWAH C	1.878985	0.9155155	3.6781609	sosondowah ankyrin repeat domain family member C
UP in Hypoxia	**5797**	PTPRM	1.196532	0.882657	2.2918808	protein tyrosine phosphatase, receptor type,
UP in Hypoxia	**5228**	PGF	1.57028	0.8797545	2.9696233	placental growth factor
UP in Hypoxia	**401232**	DKFZP6 86I15217	1.65545	0.8018723	3.1502146	uncharacterized LOC401232
UP in Hypoxia	**1152**	CKB	1.624149	0.8951088	3.0826033	creatine kinase, brain
UP in Hypoxia	**2201**	FBN2	1.037063	0.8653409	2.0520465	fibrillin 2
UP in Hypoxia	**402573**	C7orf61	1.832385	0.8684404	3.5612536	chromosome 7 open reading frame 61
UP in Hypoxia	**348487**	FAM131	1.344364	0.876879	2.5391823	family with sequence similarity 131,
UP in Hypoxia	**55726**	ASUN	1.028525	0.8767177	2.0399374	asunder, spermatogenesis regulator
UP in Hypoxia	**10272**	FSTL3	1.916512	0.8972946	3.7750929	follistatin-like 3 (secreted glycoprotein)
UP in Hypoxia	**51299**	NRN1	3.752985	0.9817074	13.482207	neuritin 1
UP in Hypoxia	**50651**	SLC45A1	1.858234	0.9066201	3.6256345	solute carrier family 45, member 1
UP in Hypoxia	**27177**	IL36B	2.09231	0.9365045	4.2643039	interleukin 36, beta
UP in Hypoxia	**55214**	LEPREL	1.849879	0.9182836	3.6046987	leprecan-like 1

UP in Hypoxia	**140766**	ADAMTS14	3.409679	0.8738422	10.627119	ADAM metallopeptidase with thrombospondin type 1 motif, 14
UP in Hypoxia	**121457**	IKBIP	1.107026	0.8799337	2.154012	IKBKB interacting protein
UP in Hypoxia	**3097**	HIVEP2	1.151618	0.8679835	2.2216288	human immunodeficiency virus type I enhancer binding protein 2
UP in Hypoxia	**23061**	TBC1D9B	1.351424	0.8971871	2.5516391	TBC1 domain family, member 9B (with GRAM domain)
UP in Hypoxia	**51477**	ISYNA1	2.036163	0.9124966	4.1015326	inositol-3-phosphate synthase 1
UP in Hypoxia	**1416**	CRYBB2P1	1.480721	0.8977246	2.7908809	crystallin, beta B2 pseudogene 1
UP in Hypoxia	**84709**	MGARP	1.107041	0.8564096	2.1540342	mitochondria-localized glutamic acid-rich
UP in Hypoxia	**284023**	LOC284023	1.192855	0.8491893	2.2860465	uncharacterized LOC284023
UP in Hypoxia	**729975**	FLJ30403	1.414014	0.8867957	2.6647763	uncharacterized LOC729975
UP in Hypoxia	**1051**	CEBPB	1.100442	0.8857476	2.1442044	CCAAT/enhancer binding protein (C/EBP),
UP in Hypoxia	**169611**	OLFML2	1.925999	0.9020783	3.8	olfactomedin-like 2A
UP in Hypoxia	**147015**	DHRS13	3.527258	0.9742901	11.529496	dehydrogenase/reductase (SDR family) member 13
UP in Hypoxia	**760**	CA2	1.959565	0.8863209	3.8894472	carbonic anhydrase II
UP in Hypoxia	**7474**	WNT5A	1.051671	0.882003	2.0729288	wingless-type MMTV integration site family, member 5A
UP in Hypoxia	**4616**	GADD45	2.390022	0.9486249	5.2416538	growth arrest and DNA-damage-inducible,
UP in Hypoxia	**220979**	C10orf25	1.629528	0.8433127	3.0941176	chromosome 10 open reading frame 25
UP in Hypoxia	**4025**	LPO	8.286327	0.9737615	312.19999	lactoperoxidase
UP in Hypoxia	**5034**	P4HB	1.072697	0.8840545	2.1033613	prolyl 4-hydroxylase, beta polypeptide
UP in Hypoxia	**118611**	C10orf90	1.789079	0.9153453	3.4559409	chromosome 10 open reading frame 90
UP in Hypoxia	**55326**	AGPAT5	1.148539	0.8809908	2.2168923	1-acylglycerol-3-phosphate O-
UP in Hypoxia	**347735**	SERINC2	1.132201	0.8547792	2.1919283	serine incorporator 2
UP in Hypoxia	**9052**	GPRC5A	1.849556	0.9209531	3.6038922	G protein-coupled receptor, family C, group 5, member A
UP in Hypoxia	**84824**	FCRLA	1.449267	0.8882021	2.7306931	Fc receptor-like A
UP in Hypoxia	**665**	BNIP3L	2.394201	0.9497357	5.2568585	BCL2/adenovirus E1B 19kDa interacting protein 3-like
UP in Hypoxia	**79365**	BHLHE4	2.287746	0.9386724	4.8829268	basic helix-loop-helix family, member e41
UP in Hypoxia	**654433**	LOC654433	1.262778	0.8928245	2.3995742	uncharacterized LOC654433
UP in Hypoxia	**728047**	GOLGA8	2.122251	0.9165099	4.3537285	golgin A8 family, member O
UP in Hypoxia	**492311**	IGIP	1.056614	0.8351787	2.0800439	IgA-inducing protein homolog (Bos taurus)
UP in Hypoxia	**83854**	ANGPTL	1.094007	0.83577	2.1346604	angiopoietin-like 6
UP in Hypoxia	**1827**	RCAN1	3.656845	0.9804533	12.613051	regulator of calcineurin 1
UP in Hypoxia	**54210**	TREM1	3.171899	0.9702051	9.0123249	triggering receptor expressed on myeloid
UP in Hypoxia	**3386**	ICAM4	3.371707	0.9180955	10.351064	intercellular adhesion molecule 4 (Landsteiner-Wiener blood group)
UP in Hypoxia	**5997**	RGS2	2.625226	0.9467258	6.1698113	regulator of G-protein signaling 2, 24kDa
UP in Hypoxia	**63893**	UBE2O	1.496136	0.8986204	2.820861	ubiquitin-conjugating enzyme E2O
UP in Hypoxia	**205428**	C3orf58	2.590709	0.9550838	6.0239464	chromosome 3 open reading frame 58
UP in Hypoxia	**6675**	UAP1	1.173862	0.8867061	2.2561479	UDP-N-acteylglucosamine
UP in Hypoxia	**9048**	ARTN	1.594634	0.8630834	3.0201794	artemin
UP in Hypoxia	**6094**	ROM1	1.020946	0.8485174	2.029249	retinal outer segment membrane protein 1
UP in Hypoxia	**84929**	FIBCD1	3.695564	0.9510257	12.95614	fibrinogen C domain containing 1
UP in Hypoxia	**55243**	KIRREL	1.106518	0.8826212	2.1532532	kin of IRRE like (Drosophila)
UP in Hypoxia	**55076**	TMEM45A	3.167053	0.9705545	8.9821019	transmembrane protein 45A
UP in Hypoxia	**3624**	INHBA	2.011262	0.8871182	4.031348	inhibin, beta A
UP in Hypoxia	**6526**	SLC5A3	1.027451	0.8624922	2.0384193	solute carrier family 5 (sodium/myo-inositol cotransporter), member 3
UP in Hypoxia	**85301**	COL27A1	1.119207	0.8583893	2.1722756	collagen, type XXVII, alpha 1

UP in Hypoxia	**3383**	ICAM1	3.134292	0.9676521	8.7804338	intercellular adhesion molecule 1
UP in Hypoxia	**120**	ADD3	1.029696	0.8774433	2.0415945	adducin 3 (gamma)
UP in Hypoxia	**342667**	STAC2	2.230136	0.8320523	4.6917808	SH3 and cysteine rich domain 2
UP in Hypoxia	**8974**	P4HA2	1.906393	0.9248141	3.7487066	prolyl 4-hydroxylase, alpha polypeptide II
UP in Hypoxia	**54039**	PCBP3	1.932031	0.9183463	3.8159204	poly(rC) binding protein 3
UP in Hypoxia	**8497**	PPFIA4	3.226617	0.9684046	9.3607069	protein tyrosine phosphatase, receptor type, f polypeptide (PTPRF), interacting protein (liprin), alpha 4
UP in Hypoxia	**9123**	SLC16A3	1.552688	0.9039595	2.9336315	solute carrier family 16, member 3 (monocarboxylic acid transporter 4)
UP in Hypoxia	**54985**	HCFC1R	2.641371	0.9568664	6.2392423	host cell factor C1 regulator 1 (XPO1
UP in Hypoxia	**25816**	TNFAIP8	1.141585	0.8712085	2.2062323	tumor necrosis factor, alpha-induced protein
UP in Hypoxia	**55818**	KDM3A	1.577103	0.9160889	2.9837005	lysine (K)-specific demethylase 3A
UP in Hypoxia	**153090**	DAB2IP	1.740209	0.9056347	3.340836	DAB2 interacting protein
UP in Hypoxia	**7805**	LAPTM5	4.941106	0.9231031	30.72	lysosomal protein transmembrane 5
UP in Hypoxia	**11162**	NUDT6	1.992554	0.9247335	3.9794082	nudix (nucleoside diphosphate linked moiety X)-type motif 6
UP in Hypoxia	**100131060**	LOC100131060	2.64564	0.8420317	6.257732	uncharacterized LOC100131060
UP in Hypoxia	**8334**	HIST1H2AC	1.375381	0.8881036	2.5943647	histone cluster 1, H2ac
UP in Hypoxia	**201191**	SAMD14	1.333942	0.8872257	2.5209051	sterile alpha motif domain containing 14
UP in Hypoxia	**2170**	FABP3	2.540568	0.890791	5.8181818	fatty acid binding protein 3, muscle and heart (mammary-derived growth inhibitor)
UP in Hypoxia	**1132**	CHRM4	3.733354	0.8615605	13.3	cholinergic receptor, muscarinic 4
UP in Hypoxia	**80824**	DUSP16	1.084335	0.8691929	2.120398	dual specificity phosphatase 16
UP in Hypoxia	**253430**	IPMK	1.566807	0.8938637	2.9624838	inositol polyphosphate multikinase
UP in Hypoxia	**440584**	SLC2A1-AS1	3.38229	0.9298128	10.427273	SLC2A1 antisense RNA 1
UP in Hypoxia	**3782**	KCNN3	2.185643	0.8219206	4.5492958	potassium intermediate/small conductance calcium-activated channel, subfamily N, member 3
UP in Hypoxia	**283651**	HMGN2P46	2.346646	0.9381797	5.0864023	high mobility group nucleosomal binding domain 2 pseudogene 46
UP in Hypoxia	**5352**	PLOD2	2.857219	0.9629401	7.24617	procollagen-lysine, 2-oxoglutarate 5-dioxygenase 2
UP in Hypoxia	**284339**	TMEM14	3.249692	0.8034668	9.5116279	transmembrane protein 145
UP in Hypoxia	**56300**	IL36G	2.915936	0.8846905	7.5471698	interleukin 36, gamma
UP in Hypoxia	**9037**	SEMA5A	1.087463	0.8653677	2.125	sema domain, seven thrombospondin repeats (type 1 and type 1-like), trans-membrane domain (TM) and short cytoplasmic domain, (semaphorin) 5A
UP in Hypoxia	**100131551**	LOC100131551	7.491051	0.9784287	179.9	uncharacterized LOC100131551
UP in Hypoxia	**594855**	CPLX3	4.239769	0.9409478	18.892857	complexin 3
UP in Hypoxia	**554223**	LOC554223	2.6861	0.8885873	6.4357143	histocompatibility antigen-related
UP in Hypoxia	**64651**	CSRNP1	1.713552	0.915408	3.279672	cysteine-serine-rich nuclear protein 1
UP in Hypoxia	**828**	CAPS	1.479151	0.8785721	2.7878465	calcyphosine
UP in Hypoxia	**1634**	DCN	1.153525	0.8019887	2.2245681	decorin
UP in Hypoxia	**1848**	DUSP6	1.204511	0.8895996	2.3045915	dual specificity phosphatase 6
UP in Hypoxia	**81629**	TSSK3	1.0452	0.8619905	2.0636526	testis-specific serine kinase 3
UP in Hypoxia	**84628**	NTNG2	2.60564	0.9239989	6.0866142	netrin G2
UP in Hypoxia	**22856**	CHSY1	1.369712	0.8945534	2.5841892	chondroitin sulfate synthase 1
UP in Hypoxia	**65110**	UPF3A	1.086587	0.8829616	2.1237102	UPF3 regulator of nonsense transcripts homolog A (yeast)
UP in Hypoxia	**2247**	FGF2	2.242906	0.9393801	4.7334963	fibroblast growth factor 2 (basic)

UP in Hypoxia	**84988**	PPP1R16	1.057399	0.8776673	2.081176	protein phosphatase 1, regulatory subunit
UP in Hypoxia	**4017**	LOXL2	1.597869	0.9182299	3.0269578	lysyl oxidase-like 2
UP in Hypoxia	**4485**	MST1	1.366565	0.8394786	2.5785582	macrophage stimulating 1 (hepatocyte growth factor-like)
UP in Hypoxia	**58476**	TP53INP	1.164031	0.8854609	2.2408271	tumor protein p53 inducible nuclear protein
UP in Hypoxia	**3872**	KRT17	5.004738	0.8989429	32.105263	keratin 17
UP in Hypoxia	**25987**	TSKU	2.29434	0.936836	4.9052938	tsukushi, small leucine rich proteoglycan
UP in Hypoxia	**9235**	IL32	2.880286	0.9498164	7.362963	interleukin 32
UP in Hypoxia	**347404**	LANCL3	1.233461	0.8254233	2.3513043	LanC lantibiotic synthetase component C-like 3 (bacterial)
UP in Hypoxia	**114**	ADCY8	1.97714	0.9214279	3.9371187	adenylate cyclase 8 (brain)
UP in Hypoxia	**376497**	SLC27A1	1.762133	0.9170922	3.3919933	solute carrier family 27 (fatty acid transporter), member 1
UP in Hypoxia	**79668**	PARP8	1.700282	0.8952522	3.2496454	poly (ADP-ribose) polymerase family,
UP in Hypoxia	**9931**	HELZ	1.009014	0.852468	2.0125348	helicase with zinc finger
UP in Hypoxia	**22977**	AKR7A3	2.070389	0.8270985	4.2	aldo-keto reductase family 7, member A3 (aflatoxin aldehyde reductase)
UP in Hypoxia	**2027**	ENO3	1.462781	0.8632626	2.756391	enolase 3 (beta, muscle)
UP in Hypoxia	**1366**	CLDN7	1.633072	0.8767984	3.1017274	claudin 7
UP in Hypoxia	**440836**	ODF3B	1.526392	0.8060109	2.8806452	outer dense fiber of sperm tails 3B
