# Supplementary material for: Integrated analysis identified core signal pathways and hypoxic characteristics of human glioblastoma
Source: J Cell Mol Med. 2019 Jul 7;23(9):6228–37. doi: 10.1111/jcmm.14507 (PMC6714287; doi:10.1111/jcmm.14507)
Supplement: Supplementary file 14 [file JCMM-23-6228-s015.pdf]

Table S7 Differential gene expression analysis by Noiseq for pairwise comparison of HEB normoxia versus 1% hypoxia, based on RNA-seq data.

	Gene	Symbol	log_2Ratio	Probability	Ratio	Description
UP in Normoxia	**112812**	FDX1L	-1.19832	0.8803576	0.4357834	ferredoxin 1-like
UP in Normoxia	**84275**	SLC25A33	-1.13957	0.8764628	0.4538952	solute carrier family 25 (pyrimidine nucleotide carrier), member 33
UP in Normoxia	**10300**	KATNB	-1.24747	0.890317	0.4211867	katanin p80 (WD repeat containing) subunit B
UP in Normoxia	**79823**	CAMKMT	-1.30015	0.8487639	0.4060832	calmodulin-lysine N-methyltransferase
UP in Normoxia	**23277**	CLUH	-1.02351	0.8796621	0.4919192	clustered mitochondria (cluA/CLU1) homolog
UP in Normoxia	**84315**	MON1A	-1.04994	0.85851	0.4829892	MON1 homolog A (yeast)
UP in Normoxia	**2936**	GSR	-1.04663	0.8793375	0.4840968	glutathione reductase
UP in Normoxia	**8884**	SLC5A6	-1.03553	0.8727999	0.4878372	solute carrier family 5 (sodium-dependent vitamin transporter), member 6
UP in Normoxia	**1802**	DPH2	-1.09841	0.8816187	0.4670308	DPH2 homolog (S. cerevisiae)
UP in Normoxia	**5245**	PHB	-1.08184	0.8858751	0.4724272	prohibitin
UP in Normoxia	**115752**	DIS3L	-1.19738	0.8726608	0.4360674	DIS3 mitotic control homolog (S. cerevisiae)-
UP in Normoxia	**115708**	TRMT61A	-1.10824	0.8608376	0.4638592	tRNA methyltransferase 61 homolog A (S. cerevisiae)
UP in Normoxia	**6080**	SNORA73A	-1.36777	0.8656225	0.3874891	small nucleolar RNA, H/ACA box 73A
UP in Normoxia	**1844**	DUSP2	-1.39167	0.8896586	0.3811245	dual specificity phosphatase 2
UP in Normoxia	**8817**	FGF18	-1.21244	0.8630817	0.4315368	fibroblast growth factor 18
UP in Normoxia	**10940**	POP1	-1.28292	0.8854949	0.4109639	processing of precursor 1, ribonuclease P/MRP subunit (S. cerevisiae)
UP in Normoxia	**112724**	RDH13	-1.02555	0.8523155	0.4912224	retinol dehydrogenase 13 (all-trans/9-cis)
UP in Normoxia	**6542**	SLC7A2	-1.0201	0.8745711	0.4930832	solute carrier family 7 (cationic amino acid transporter, y+ system), member 2
UP in Normoxia	**201164**	PLD6	-1.5997	0.8496727	0.3299453	phospholipase D family, member 6
UP in Normoxia	**55003**	PAK1IP1	-1.12626	0.8844378	0.4581015	PAK1 interacting protein 1
UP in Normoxia	**25929**	GEMIN5	-1.38697	0.8958345	0.3823668	gem (nuclear organelle) associated protein 5
UP in Normoxia	**5699**	PSMB10	-1.24508	0.8809696	0.4218854	proteasome (prosome, macropain) subunit, beta type, 10
UP in Normoxia	**79814**	AGMAT	-1.24834	0.8402604	0.4209316	agmatine ureohydrolase (agmatinase)
UP in Normoxia	**4835**	NQO2	-1.17437	0.8911886	0.4430775	NAD(P)H dehydrogenase, quinone 2
UP in Normoxia	**54433**	GAR1	-1.07774	0.8745897	0.4737689	GAR1 ribonucleoprotein homolog (yeast)
UP in Normoxia	**10514**	MYBBP1A	-1.10491	0.8842059	0.4649323	MYB binding protein (P160) 1a
UP in Normoxia	**10244**	RABEPK	-1.38555	0.8926352	0.3827423	Rab9 effector protein with kelch motifs
UP in Normoxia	**91942**	NDUFAF2	-1.06828	0.8783546	0.4768862	NADH dehydrogenase (ubiquinone) complex I, assembly factor 2
UP in Normoxia	**55612**	FERMT1	-1.5441	0.8207775	0.3429084	fermitin family member 1
UP in Normoxia	**51073**	MRPL4	-1.22944	0.8935718	0.4264821	mitochondrial ribosomal protein L4
UP in Normoxia	**8814**	CDKL1	-1.40308	0.8863944	0.3781204	cyclin-dependent kinase-like 1 (CDC2-related kinase)
UP in Normoxia	**5866**	RAB3IL	-1.26499	0.8806821	0.4161011	RAB3A interacting protein (rabin3)-like 1
UP in Normoxia	**79080**	CCDC86	-1.23499	0.892153	0.4248465	coiled-coil domain containing 86
UP in Normoxia	**59271**	EVA1C	-1.06781	0.834409	0.477042	eva-1 homolog C (C. elegans)
UP in Normoxia	**9136**	RRP9	-1.5991	0.9162262	0.3300838	ribosomal RNA processing 9, small subunit (SSU) processome component, homolog (yeast)
UP in Normoxia	**7804**	LRP8	-1.12157	0.8696285	0.4595922	low density lipoprotein receptor-related protein 8, apolipoprotein e receptor
UP in Normoxia	**725**	C4BPB	-1.00891	0.8078321	0.4969212	complement component 4 binding protein,
UP in Normoxia	**359845**	FAM101	-1.49362	0.8742651	0.3551207	family with sequence similarity 101, member
UP in Normoxia	**124976**	SPNS2	-1.04318	0.8480684	0.4852566	spinster homolog 2 (Drosophila)

UP in Normoxia	**644019**	CBWD6	-1.09225	0.8418461	0.4690305	COBW domain containing 6
UP in Normoxia	**54865**	GPATC	-1.42637	0.8988205	0.3720649	G patch domain containing 4
UP in Normoxia	**55020**	TTC38	-1.06586	0.8735418	0.4776873	tetratricopeptide repeat domain 38
UP in Normoxia	**27124**	INPP5J	-1.05895	0.8367922	0.4799816	inositol polyphosphate-5-phosphatase J
UP in Normoxia	**51388**	NIP7	-1.03555	0.8795879	0.4878289	NIP7, nucleolar pre-rRNA processing protein
UP in Normoxia	**10329**	TMEM5	-1.14825	0.8666611	0.4511725	transmembrane protein 5
UP in Normoxia	**595**	CCND1	-1.13224	0.8806172	0.4562058	cyclin D1
UP in Normoxia	**56902**	PNO1	-1.0266	0.8745248	0.4908667	partner of NOB1 homolog (S. cerevisiae)
UP in Normoxia	**10969**	EBNA1B P2	-1.1879	0.8916894	0.4389408	EBNA1 binding protein 2
UP in Normoxia	**51042**	ZNF593	-1.10241	0.8828428	0.4657368	zinc finger protein 593
UP in Normoxia	**84967**	LSM10	-1.02169	0.8752202	0.492539	LSM10, U7 small nuclear RNA associated
UP in Normoxia	**10212**	DDX39A	-1.03947	0.8808769	0.4865071	DEAD (Asp-Glu-Ala-Asp) box polypeptide
UP in Normoxia	**11185**	INMT	-1.02734	0.8266752	0.4906139	indolethylamine N-methyltransferase
UP in Normoxia	**9221**	NOLC1	-1.02483	0.8822771	0.4914682	nucleolar and coiled-body phosphoprotein 1
UP in Normoxia	**79077**	DCTPP1	-1.14601	0.8885921	0.4518736	dCTP pyrophosphatase 1
UP in Normoxia	**30834**	ZNRD1	-1.01514	0.877418	0.4947789	zinc ribbon domain containing 1
UP in Normoxia	**6081**	SNORA7 3B	-3.60034	0.8987463	0.0824499	small nucleolar RNA, H/ACA box 73B
UP in Normoxia	**84908**	FAM136 A	-1.11552	0.8867468	0.4615247	family with sequence similarity 136, member A
UP in Normoxia	**23246**	BOP1	-1.06405	0.8817671	0.4782888	block of proliferation 1
UP in Normoxia	**84946**	LTV1	-1.08763	0.8792633	0.4705324	LTV1 homolog (S. cerevisiae)
UP in Normoxia	**133522**	PPARGC 1B	-1.13605	0.8077579	0.4550036	peroxisome proliferator-activated receptor gamma, coactivator 1 beta
UP in Normoxia	**5321**	PLA2G4 A	-1.05708	0.8554498	0.4806028	phospholipase A2, group IVA (cytosolic, calcium-dependent)
UP in Normoxia	**55622**	TTC27	-1.03747	0.8565904	0.4871795	tetratricopeptide repeat domain 27
UP in Normoxia	**65260**	SELRC1	-1.01796	0.8744506	0.4938139	Sel1 repeat containing 1
UP in Normoxia	**3887**	KRT81	-1.09287	0.8471225	0.4688269	keratin 81
UP in Normoxia	**26873**	OPLAH	-1.01025	0.8697954	0.4964586	5-oxoprolinase (ATP-hydrolysing)
UP in Normoxia	**6446**	SGK1	-1.35146	0.8999796	0.3918941	serum/glucocorticoid regulated kinase 1
UP in Normoxia	**516**	ATP5G1	-1.02284	0.8826666	0.4921481	ATP synthase, H+ transporting, mitochondrial Fo complex, subunit C1 (subunit 9)
UP in Normoxia	**22809**	ATF5	-1.06106	0.8506556	0.4792793	activating transcription factor 5
UP in Normoxia	**23212**	RRS1	-1.18456	0.8891856	0.4399587	RRS1 ribosome biogenesis regulator homolog (S. cerevisiae)
UP in Normoxia	**84769**	MPV17L	-1.25082	0.8840947	0.4202088	MPV17 mitochondrial membrane protein-like
UP in Normoxia	**246243**	RNASE	-1.01442	0.8766576	0.4950288	ribonuclease H1
UP in Normoxia	**677777**	SCARN A12	-1.03966	0.8589736	0.4864412	small Cajal body-specific RNA 12
UP in Normoxia	**26353**	HSPB8	-1.23249	0.8909104	0.4255836	heat shock 22kDa protein 8
UP in Normoxia	**65083**	NOL6	-1.39755	0.8968174	0.3795741	nucleolar protein 6 (RNA-associated)
UP in Normoxia	**57291**	DANCR	-1.04232	0.8816929	0.4855457	differentiation antagonizing non-protein coding RNA
UP in Normoxia	**54512**	EXOSC4	-1.07022	0.8800887	0.4762478	exosome component 4
UP in Normoxia	**4839**	NOP2	-1.08535	0.8833992	0.4712786	NOP2 nucleolar protein
UP in Normoxia	**84254**	CAMKK 1	-1.04751	0.8563215	0.483802	calcium/calmodulin-dependent protein kinase kinase 1, alpha
UP in Normoxia	**4830**	NME1	-1.0986	0.887841	0.4669679	NME/NM23 nucleoside diphosphate kinase 1
UP in Normoxia	**23223**	RRP12	-1.09715	0.8777425	0.4674391	ribosomal RNA processing 12 homolog (S. cerevisiae)
UP in Normoxia	**3757**	KCNH2	-1.20891	0.8618298	0.4325948	potassium voltage-gated channel, subfamily H (eag-related), member 2
UP in Normoxia	**5436**	POLR2G	-1.05926	0.8816651	0.4798797	polymerase (RNA) II (DNA directed) polypeptide G
UP in Normoxia	**196483**	FAM86A	-1.06808	0.8614496	0.4769533	family with sequence similarity 86, member A

UP in Normoxia	**26519**	TIMM10	-1.07845	0.8819062	0.4735386	translocase of inner mitochondrial membrane 10 homolog (yeast)
UP in Normoxia	**115572**	FAM46B	-1.21053	0.8469927	0.432111	family with sequence similarity 46, member B
UP in Normoxia	**197259**	MLKL	-1.05537	0.8097517	0.4811742	mixed lineage kinase domain-like
UP in Normoxia	**51491**	NOP16	-1.24578	0.8947681	0.4216796	NOP16 nucleolar protein
UP in Normoxia	**348180**	CTU2	-1.22679	0.8772974	0.4272658	cytosolic thiouridylase subunit 2 homolog (S. pombe)
UP in Normoxia	**730394**	GTF2H2	-1.03389	0.8678202	0.4883902	general transcription factor IIH, polypeptide
UP in Normoxia	**100532735**	INO80B-WBP1	-2.67939	0.9282906	0.1561075	INO80B-WBP1 readthrough
UP in Normoxia	**84627**	ZNF469	-1.4117	0.8142677	0.3758681	zinc finger protein 469
UP in Normoxia	**28999**	KLF15	-3.91648	0.8634804	0.0662252	Kruppel-like factor 15
UP in Normoxia	**10534**	SSSCA1	-1.07008	0.8800423	0.4762933	Sjogren syndrome/scleroderma autoantigen 1
UP in Normoxia	**152579**	SCFD2	-1.52073	0.8280847	0.3485099	sec1 family domain containing 2
UP in Normoxia	**10196**	PRMT3	-1.24802	0.8886014	0.4210252	protein arginine methyltransferase 3
UP in Normoxia	**55210**	ATAD3	-1.17671	0.8898626	0.4423589	ATPase family, AAA domain containing 3A
UP in Normoxia	**100008587**	RNA5-	-3.14245	0.9666166	0.1132477	RNA, 5.8S ribosomal 5
UP in Normoxia	**4953**	ODC1	-1.41539	0.9031603	0.3749071	ornithine decarboxylase 1
UP in Normoxia	**83858**	ATAD3B	-1.15288	0.8866355	0.449727	ATPase family, AAA domain containing 3B
UP in Normoxia	**8891**	EIF2B3	-1.16832	0.881424	0.4449383	eukaryotic translation initiation factor 2B, subunit 3 gamma, 58kDa
UP in Normoxia	**619568**	SNORA4	-1.97072	0.8269441	0.2551253	small nucleolar RNA, H/ACA box 4
UP in Normoxia	**100526837**	EEF1E1-MUTED	-7.7211	0.8947124	0.0047393	EEF1E1-MUTED readthrough
UP in Normoxia	**133584**	EGFLA M	-1.07758	0.8670228	0.4738226	EGF-like, fibronectin type III and laminin G domains
UP in Normoxia	**219738**	C10orf35	-1.12214	0.8471411	0.4594128	chromosome 10 open reading frame 35
UP in Normoxia	**1438**	CSF2RA	-1.48409	0.8324338	0.357473	colony stimulating factor 2 receptor, alpha, low-affinity (granulocyte-macrophage)
UP in Normoxia	**27340**	UTP20	-1.12267	0.875109	0.4592438	UTP20, small subunit (SSU) processome component, homolog (yeast)
UP in Normoxia	**6652**	SORD	-1.28582	0.8945362	0.4101385	sorbitol dehydrogenase
UP in Normoxia	**9277**	WDR46	-1.07525	0.8795879	0.4745904	WD repeat domain 46
UP in Normoxia	**2184**	FAH	-1.06274	0.8626644	0.4787206	fumarylacetoacetate hydrolase (fumarylacetoacetase)
UP in Normoxia	**100128881**	LOC100 128881	-1.38279	0.8883047	0.3834764	uncharacterized LOC100128881
UP in Normoxia	**131870**	NUDT16	-1.02041	0.8550975	0.4929746	nudix (nucleoside diphosphate linked moiety X)-type motif 16
UP in Normoxia	**92935**	MARS2	-1.04345	0.8565904	0.4851663	methionyl-tRNA synthetase 2, mitochondrial
UP in Normoxia	**91807**	MYLK3	-1.47032	0.8929969	0.3609023	myosin light chain kinase 3
UP in Normoxia	**1662**	DDX10	-1.15119	0.8763145	0.4502533	DEAD (Asp-Glu-Ala-Asp) box polypeptide
UP in Normoxia	**6891**	TAP2	-1.00966	0.8723363	0.4966639	transporter 2, ATP-binding cassette, sub-family B (MDR/TAP)
UP in Normoxia	**55646**	LYAR	-1.13774	0.8830839	0.4544714	Ly1 antibody reactive
UP in Normoxia	**8439**	NSMAF	-1.02347	0.8607355	0.4919334	neutral sphingomyelinase (N-SMase) activation associated factor
UP in Normoxia	**23109**	DDN	-2.36557	0.9022515	0.1940402	dendrin
UP in Normoxia	**139285**	AMER1	-1.03577	0.8035386	0.4877565	APC membrane recruitment protein 1
UP in Normoxia	**8293**	SERF1A	-7.52617	0.8750811	0.005425	small EDRK-rich factor 1A (telomeric)
UP in Normoxia	**728340**	GTF2H2	-1.10038	0.8093807	0.4663951	general transcription factor IIH, polypeptide
UP in Normoxia	**136319**	MTPN	-8.11201	0.9257776	0.0036145	myotrophin
UP in Normoxia	**6723**	SRM	-1.15099	0.8905302	0.4503149	spermidine synthase
UP in Normoxia	**55199**	FAM86C	-1.13159	0.8213709	0.4564134	family with sequence similarity 86, member
UP in Normoxia	**5026**	P2RX5	-1.43505	0.885838	0.369833	purinergic receptor P2X, ligand-gated ion channel, 5
UP in Normoxia	**3491**	CYR61	-1.44979	0.8949072	0.3660743	cysteine-rich, angiogenic inducer, 61

UP in Hypoxia	**57658**	CALCO CO1	1.043851	0.876741	2.0617231	calcium binding and coiled-coil domain 1
UP in Hypoxia	**646960**	PRSS56	1.479328	0.8374506	2.7881874	protease, serine, 56
UP in Hypoxia	**9935**	MAFB	3.696219	0.918359	12.962025	v-maf musculoaponeurotic fibrosarcoma oncogene homolog B (avian)
UP in Hypoxia	**492307**	C8orf22	1.312215	0.8971884	2.4832246	chromosome 8 open reading frame 22
UP in Hypoxia	**6513**	SLC2A1	2.258398	0.9397893	4.7846002	solute carrier family 2 (facilitated glucose transporter), member 1
UP in Hypoxia	**1294**	COL7A1	1.819285	0.924535	3.529063	collagen, type VII, alpha 1
UP in Hypoxia	**1675**	CFD	1.503043	0.9024741	2.834399	complement factor D (adipsin)
UP in Hypoxia	**1289**	COL5A1	1.419291	0.9032438	2.6745395	collagen, type V, alpha 1
UP in Hypoxia	**1030**	CDKN2 B	1.866104	0.903151	3.6454668	cyclin-dependent kinase inhibitor 2B (p15, inhibits CDK4)
UP in Hypoxia	**8912**	CACNA 1H	2.514904	0.8155845	5.7155963	calcium channel, voltage-dependent, T type, alpha 1H subunit
UP in Hypoxia	**51200**	CPA4	2.125669	0.9045142	4.3640553	carboxypeptidase A4
UP in Hypoxia	**3823**	KLRC3	1.357618	0.8288544	2.5626168	killer cell lectin-like receptor subfamily C,
UP in Hypoxia	**3162**	HMOX1	1.215023	0.8921345	2.3214444	heme oxygenase (decycling) 1
UP in Hypoxia	**154761**	LOC154 761	4.267104	0.935774	19.254237	family with sequence similarity 115, member C pseudogene
UP in Hypoxia	**10381**	TUBB3	1.105885	0.8759621	2.1523083	tubulin, beta 3 class III
UP in Hypoxia	**100131564**	LOC100 131564	1.169133	0.8103637	2.2487644	uncharacterized LOC100131564
UP in Hypoxia	**26355**	FAM162 A	2.155008	0.9385931	4.4537107	family with sequence similarity 162, member A
UP in Hypoxia	**6659**	SOX4	1.138646	0.8841874	2.2017426	SRY (sex determining region Y)-box 4
UP in Hypoxia	**79873**	NUDT18	1.628801	0.8729761	3.092559	nudix (nucleoside diphosphate linked moiety X)-type motif 18
UP in Hypoxia	**9022**	CLIC3	2.043249	0.9157254	4.1217257	chloride intracellular channel 3
UP in Hypoxia	**84189**	SLITRK	4.798742	0.9625642	27.833333	SLIT and NTRK-like family, member 6
UP in Hypoxia	**25946**	ZNF385	1.249439	0.8833806	2.3774896	zinc finger protein 385A
UP in Hypoxia	**3955**	LFNG	1.629403	0.9048109	3.0938486	LFNG O-fucosylpeptide 3-beta-N-acetylglucosaminyltransferase
UP in Hypoxia	**79930**	DOK3	1.039447	0.8516757	2.0554401	docking protein 3
UP in Hypoxia	**1591**	CYP24A 1	1.457392	0.8088707	2.746114	cytochrome P450, family 24, subfamily A, polypeptide 1
UP in Hypoxia	**113146**	AHNAK	1.676157	0.9179232	3.1957558	AHNAK nucleoprotein 2
UP in Hypoxia	**56901**	NDUFA4 L2	6.720207	0.9979414	105.43478	NADH dehydrogenase (ubiquinone) 1 alpha subcomplex, 4-like 2
UP in Hypoxia	**83394**	PITPNM	1.145949	0.8469834	2.2129162	PITPNM family member 3
UP in Hypoxia	**3678**	ITGA5	2.349292	0.9477735	5.0957424	integrin, alpha 5 (fibronectin receptor, alpha polypeptide)
UP in Hypoxia	**7852**	CXCR4	1.472603	0.9044214	2.7752215	chemokine (C-X-C motif) receptor 4
UP in Hypoxia	**55806**	HR	1.094891	0.8741909	2.1359699	hair growth associated
UP in Hypoxia	**10397**	NDRG1	4.033775	0.9846251	16.379001	N-myc downstream regulated 1
UP in Hypoxia	**55139**	ANKZF1	1.601177	0.9173019	3.0339071	ankyrin repeat and zinc finger domain
UP in Hypoxia	**771**	CA12	1.816761	0.9206588	3.5228947	carbonic anhydrase XII
UP in Hypoxia	**51317**	PHF21A	1.120818	0.8651032	2.1747026	PHD finger protein 21A
UP in Hypoxia	**147040**	KCTD11	2.382294	0.941375	5.2136525	potassium channel tetramerisation domain containing 11
UP in Hypoxia	**83719**	YPEL3	1.657527	0.9073147	3.1547531	yippee-like 3 (Drosophila)
UP in Hypoxia	**283**	ANG	1.857856	0.8711771	3.6246851	angiogenin, ribonuclease, RNase A family, 5
UP in Hypoxia	**7040**	TGFB1	1.415532	0.9004989	2.6675802	transforming growth factor, beta 1
UP in Hypoxia	**26232**	FBXO2	1.05557	0.8766854	2.0785393	F-box protein 2
UP in Hypoxia	**226**	ALDOA	1.411008	0.9026688	2.6592281	aldolase A, fructose-bisphosphate
UP in Hypoxia	**139728**	PNCK	4.231163	0.8995345	18.780488	pregnancy up-regulated non-ubiquitously expressed CaM kinase

UP in Hypoxia	**9249**	DHRS3	1.627964	0.904542	3.0907643	dehydrogenase/reductase (SDR family)
UP in Hypoxia	**100292680**	LOC100292680	1.086615	0.8795323	2.1237515	uncharacterized LOC100292680
UP in Hypoxia	**5493**	PPL	1.509237	0.9040876	2.8465954	periplakin
UP in Hypoxia	**140686**	WFDC3	6.130868	0.9309242	70.076923	WAP four-disulfide core domain 3
UP in Hypoxia	**29923**	HILPDA	1.735635	0.9193883	3.3302603	hypoxia inducible lipid droplet-associated
UP in Hypoxia	**2256**	FGF11	2.25293	0.9065821	4.7664975	fibroblast growth factor 11
UP in Hypoxia	**5768**	QSOX1	2.323372	0.9482279	5.0050076	quiescin Q6 sulfhydryl oxidase 1
UP in Hypoxia	**2057**	EPOR	1.002091	0.8282887	2.0029014	erythropoietin receptor
UP in Hypoxia	**79906**	MORN1	1.232116	0.8017304	2.3491124	MORN repeat containing 1
UP in Hypoxia	**3017**	HIST1H2BD	1.048784	0.875415	2.0687858	histone cluster 1, H2bd
UP in Hypoxia	**64866**	CDCP1	1.220193	0.8881099	2.329778	CUB domain containing protein 1
UP in Hypoxia	**401152**	C4orf3	2.016615	0.9345871	4.0463342	chromosome 4 open reading frame 3
UP in Hypoxia	**80856**	KIAA1715	1.176802	0.8686734	2.2607509	KIAA1715
UP in Hypoxia	**3939**	LDHA	1.22689	0.8960663	2.3406187	lactate dehydrogenase A
UP in Hypoxia	**22885**	ABLIM3	2.639787	0.8718726	6.2323944	actin binding LIM protein family, member 3
UP in Hypoxia	**8870**	IER3	1.416425	0.9030305	2.6692318	immediate early response 3
UP in Hypoxia	**5274**	SERPINI1	2.074048	0.9276044	4.2106643	serpin peptidase inhibitor, clade I (neuroserpin), member 1
UP in Hypoxia	**84218**	TBC1D3	1.261348	0.888453	2.3971963	TBC1 domain family, member 3F
UP in Hypoxia	**3623**	INHA	1.575086	0.8240973	2.9795322	inhibin, alpha
UP in Hypoxia	**51279**	C1RL	1.611489	0.9105788	3.0556701	complement component 1, r subcomponent-
UP in Hypoxia	**1291**	COL6A1	1.540808	0.8555704	2.9095745	collagen, type VI, alpha 1
UP in Hypoxia	**200058**	FLJ2386	2.365484	0.8973089	5.1532567	uncharacterized protein FLJ23867
UP in Hypoxia	**8614**	STC2	1.238761	0.8926074	2.3599572	stanniocalcin 2
UP in Hypoxia	**25794**	FSCN2	4.168288	0.913435	17.979592	fascin homolog 2, actin-bundling protein, retinal (Strongylocentrotus purpuratus)
UP in Hypoxia	**9537**	TP53I11	1.319998	0.8693318	2.4966571	tumor protein p53 inducible protein 11
UP in Hypoxia	**23764**	MAFF	1.862988	0.8655298	3.6376022	v-maf musculoaponeurotic fibrosarcoma oncogene homolog F (avian)
UP in Hypoxia	**629**	CFB	1.179425	0.847206	2.2648649	complement factor B
UP in Hypoxia	**64699**	TMPRSS	1.216871	0.8626273	2.3244207	transmembrane protease, serine 3
UP in Hypoxia	**117166**	WFIKKN1	3.201192	0.9566479	9.1971831	WAP, follistatin/kazal, immunoglobulin, kunitz and netrin domain containing 1
UP in Hypoxia	**254439**	C11orf86	1.699857	0.9163838	3.2486872	chromosome 11 open reading frame 86
UP in Hypoxia	**5507**	PPP1R3	1.790045	0.9218736	3.4582574	protein phosphatase 1, regulatory subunit 3C
UP in Hypoxia	**28959**	TMEM176B	1.307604	0.8484764	2.4753004	transmembrane protein 176B
UP in Hypoxia	**5909**	RAP1GA	1.177182	0.8760455	2.2613459	RAP1 GTPase activating protein
UP in Hypoxia	**54541**	DDIT4	1.326475	0.9005267	2.507891	DNA-damage-inducible transcript 4
UP in Hypoxia	**26035**	GLCE	1.211824	0.867913	2.3163035	glucuronic acid epimerase
UP in Hypoxia	**3099**	HK2	1.671437	0.9182106	3.1853182	hexokinase 2
UP in Hypoxia	**218**	ALDH3A1	1.224278	0.8496263	2.3363844	aldehyde dehydrogenase 3 family, member A1
UP in Hypoxia	**84343**	HPS3	1.107903	0.8721137	2.1553221	Hermansky-Pudlak syndrome 3
UP in Hypoxia	**4330**	MN1	1.396506	0.8710751	2.6326316	meningioma (disrupted in balanced translocation) 1
UP in Hypoxia	**7162**	TPBG	1.573707	0.9015375	2.9766867	trophoblast glycoprotein
UP in Hypoxia	**393**	ARHGAP4	2.989095	0.8461489	7.939759	Rho GTPase activating protein 4
UP in Hypoxia	**9185**	REPS2	1.62248	0.8106512	3.0790378	RALBP1 associated Eps domain containing 2
UP in Hypoxia	**3418**	IDH2	1.856911	0.9242104	3.622312	isocitrate dehydrogenase 2 (NADP+), mitochondrial
UP in Hypoxia	**230**	ALDOC	2.952878	0.9625271	7.7429221	aldolase C, fructose-bisphosphate
UP in Hypoxia	**8839**	WISP2	1.987332	0.9268625	3.9650298	WNT1 inducible signaling pathway protein 2

UP in Hypoxia	**2026**	ENO2	2.416662	0.9488956	5.3393408	enolase 2 (gamma, neuronal)
UP in Hypoxia	**284185**	LINC00482	2.037802	0.8459541	4.1061947	long intergenic non-protein coding RNA 482
UP in Hypoxia	**10420**	TESK2	1.010255	0.8036777	2.0142672	testis-specific kinase 2
UP in Hypoxia	**23581**	CASP14	8.159871	0.9289953	286	caspase 14, apoptosis-related cysteine
UP in Hypoxia	**5230**	PGK1	1.932642	0.926575	3.8175377	phosphoglycerate kinase 1
UP in Hypoxia	**347902**	AMIGO2	1.966942	0.8612363	3.9093851	adhesion molecule with Ig-like domain 2
UP in Hypoxia	**648791**	PPP1R3	4.291275	0.9583357	19.579545	protein phosphatase 1, regulatory subunit 3G
UP in Hypoxia	**170384**	FUT11	1.873091	0.9235149	3.6631648	fucosyltransferase 11 (alpha (1,3) fucosyltransferase)
UP in Hypoxia	**5054**	SERPINE1	4.009691	0.9802574	16.107843	serpin peptidase inhibitor, clade E (nexin, plasminogen activator inhibitor type 1), member 1
UP in Hypoxia	**122622**	ADSSL1	1.698953	0.9193049	3.2466516	adenylosuccinate synthase like 1
UP in Hypoxia	**1942**	EFNA1	1.289856	0.895547	2.4450359	ephrin-A1
UP in Hypoxia	**6095**	RORA	5.142958	0.8608932	35.333333	RAR-related orphan receptor A
UP in Hypoxia	**5163**	PDK1	2.388936	0.9440179	5.23771	pyruvate dehydrogenase kinase, isozyme 1
UP in Hypoxia	**56892**	C8orf4	1.179519	0.8868117	2.2650128	chromosome 8 open reading frame 4
UP in Hypoxia	**25787**	DGCR9	2.302299	0.8249689	4.9324324	DiGeorge syndrome critical region gene 9
UP in Hypoxia	**1852**	DUSP9	1.080524	0.80097	2.1148036	dual specificity phosphatase 9
UP in Hypoxia	**5526**	PPP2R5	2.117799	0.915067	4.3403141	protein phosphatase 2, regulatory subunit B',
UP in Hypoxia	**85360**	SYDE1	1.427345	0.8978375	2.6895138	synapse defective 1, Rho GTPase, homolog 1 (C. elegans)
UP in Hypoxia	**10882**	C1QL1	3.950079	0.9831043	15.455827	complement component 1, q subcomponent-
UP in Hypoxia	**56967**	C14orf13	2.130363	0.8729205	4.3782772	chromosome 14 open reading frame 132
UP in Hypoxia	**1991**	ELANE	4.658832	0.8699994	25.26087	elastase, neutrophil expressed
UP in Hypoxia	**5165**	PDK3	1.551363	0.8931916	2.9309392	pyruvate dehydrogenase kinase, isozyme 3
UP in Hypoxia	**3665**	IRF7	1.316314	0.8366531	2.4902913	interferon regulatory factor 7
UP in Hypoxia	**6038**	RNASE4	2.419826	0.8743578	5.3510638	ribonuclease, RNase A family, 4
UP in Hypoxia	**22936**	ELL2	1.081951	0.8647694	2.1168975	elongation factor, RNA polymerase II, 2
UP in Hypoxia	**389337**	ARHGEF37	2.602621	0.9015468	6.0738916	Rho guanine nucleotide exchange factor (GEF) 37
UP in Hypoxia	**5270**	SERPINE2	1.721299	0.9207793	3.2973318	serpin peptidase inhibitor, clade E (nexin, plasminogen activator inhibitor type 1), member 2
UP in Hypoxia	**4015**	LOX	4.055627	0.9819544	16.628966	lysyl oxidase
UP in Hypoxia	**6641**	SNTB1	1.645665	0.8125893	3.1289199	syntrophin, beta 1 (dystrophin-associated protein A1, 59kDa, basic component 1)
UP in Hypoxia	**280**	AMY2B	1.447573	0.8846974	2.7274882	amylase, alpha 2B (pancreatic)
UP in Hypoxia	**23015**	GOLGA8A	1.493339	0.9050706	2.8153982	golgin A8 family, member A
UP in Hypoxia	**5351**	PLOD1	1.671157	0.920835	3.1846998	procollagen-lysine, 2-oxoglutarate 5-
UP in Hypoxia	**79444**	BIRC7	1.715219	0.8067657	3.2834646	baculoviral IAP repeat containing 7
UP in Hypoxia	**2149**	F2R	1.454042	0.8942302	2.7397454	coagulation factor II (thrombin) receptor
UP in Hypoxia	**25976**	TIPARP	1.004929	0.8527884	2.0068451	TCDD-inducible poly(ADP-ribose)
UP in Hypoxia	**861**	RUNX1	1.230273	0.8803576	2.346114	runt-related transcription factor 1
UP in Hypoxia	**30001**	ERO1L	1.133971	0.8821195	2.1946191	ERO1-like (S. cerevisiae)
UP in Hypoxia	**7045**	TGFBI	1.090839	0.8871919	2.1299788	transforming growth factor, beta-induced,
UP in Hypoxia	**641649**	TMEM9	2.321928	0.8921067	5	transmembrane protein 91
UP in Hypoxia	**3300**	DNAJB2	1.226622	0.8890373	2.3401839	DnaJ (Hsp40) homolog, subfamily B, member
UP in Hypoxia	**60598**	KCNK15	1.305854	0.8776313	2.4723011	potassium channel, subfamily K, member 15
UP in Hypoxia	**57683**	ZDBF2	1.332983	0.8505722	2.5192308	zinc finger, DBF-type containing 2
UP in Hypoxia	**126731**	CCSAP	1.07354	0.8168178	2.1045918	centriole, cilia and spindle-associated protein
UP in Hypoxia	**389524**	GTF2IRD2B	1.76885	0.8539198	3.4078212	GTF2I repeat domain containing 2B
UP in Hypoxia	**404550**	C16orf74	1.301091	0.8518797	2.4641509	chromosome 16 open reading frame 74
UP in Hypoxia	**3155**	HMGCL	1.025202	0.8663551	2.0352442	3-hydroxymethyl-3-methylglutaryl-CoA lyase

UP in Hypoxia	**93129**	ORAI3	2.314922	0.9291437	4.9757785	ORAI calcium release-activated calcium modulator 3
UP in Hypoxia	**1748**	DLX4	2.535616	0.9303585	5.7982456	distal-less homeobox 4
UP in Hypoxia	**2821**	GPI	1.303201	0.8982548	2.4677573	glucose-6-phosphate isomerase
UP in Hypoxia	**2564**	GABRE	1.063962	0.8790037	2.0906656	gamma-aminobutyric acid (GABA) A receptor, epsilon
UP in Hypoxia	**654**	BMP6	1.031053	0.8412248	2.0435146	bone morphogenetic protein 6
UP in Hypoxia	**4035**	LRP1	2.109543	0.9373505	4.3155456	low density lipoprotein receptor-related
UP in Hypoxia	**8660**	IRS2	1.500093	0.8682375	2.82861	insulin receptor substrate 2
UP in Hypoxia	**51141**	INSIG2	2.262149	0.9352455	4.797054	insulin induced gene 2
UP in Hypoxia	**8553**	BHLHE4	1.795921	0.9234778	3.4723719	basic helix-loop-helix family, member e40
UP in Hypoxia	**84314**	TMEM107	1.110705	0.8724012	2.159512	transmembrane protein 107
UP in Hypoxia	**56917**	MEIS3	1.080896	0.8434875	2.1153497	Meis homeobox 3
UP in Hypoxia	**84518**	CNFN	1.766647	0.9076485	3.4026217	cornifelin
UP in Hypoxia	**6559**	SLC12A3	1.100564	0.8678573	2.144385	solute carrier family 12 (sodium/chloride transporters), member 3
UP in Hypoxia	**259173**	ALS2CL	1.300992	0.8050502	2.4639831	ALS2 C-terminal like
UP in Hypoxia	**6533**	SLC6A6	1.397736	0.9014911	2.6348781	solute carrier family 6 (neurotransmitter transporter, taurine), member 6
UP in Hypoxia	**10570**	DPYSL4	4.759849	0.973488	27.093023	dihydropyrimidinase-like 4
UP in Hypoxia	**283120**	H19	1.820062	0.9229771	3.5309639	H19, imprinted maternally expressed transcript (non-protein coding)
UP in Hypoxia	**100874249**	DENND5B-AS1	1.6572	0.8941282	3.1540373	DENND5B antisense RNA 1
UP in Hypoxia	**55824**	PAG1	1.511778	0.8369499	2.8516129	phosphoprotein associated with glycosphingolipid microdomains 1
UP in Hypoxia	**604**	BCL6	1.278956	0.8696007	2.4266327	B-cell CLL/lymphoma 6
UP in Hypoxia	**5798**	PTPRN	1.408482	0.8420223	2.6545769	protein tyrosine phosphatase, receptor type, N
UP in Hypoxia	**9244**	CRLF1	1.390743	0.8314416	2.6221374	cytokine receptor-like factor 1
UP in Hypoxia	**92126**	DSEL	1.05478	0.8750626	2.0774019	dermatan sulfate epimerase-like
UP in Hypoxia	**3669**	ISG20	2.326574	0.9156605	5.016129	interferon stimulated exonuclease gene 20kDa
UP in Hypoxia	**10252**	SPRY1	1.920692	0.8154454	3.7860465	sprouty homolog 1, antagonist of FGF signaling (Drosophila)
UP in Hypoxia	**10529**	NEBL	1.633636	0.8630446	3.1029412	nebulette
UP in Hypoxia	**4151**	MB	2.018942	0.8442942	4.0528634	myoglobin
UP in Hypoxia	**388341**	FAM211A	2.269916	0.8937944	4.8229508	family with sequence similarity 211, member A
UP in Hypoxia	**26585**	GREM1	2.51885	0.8667631	5.73125	gremlin 1, DAN family BMP antagonist
UP in Hypoxia	**10687**	PNMA2	1.174643	0.8843172	2.2573703	paraneoplastic Ma antigen 2
UP in Hypoxia	**8862**	APLN	2.736397	0.9371836	6.664042	apelin
UP in Hypoxia	**8698**	S1PR4	3.067745	0.8499972	8.3846154	sphingosine-1-phosphate receptor 4
UP in Hypoxia	**728806**	NSFP1	2.868356	0.8869693	7.3023256	N-ethylmaleimide-sensitive factor pseudogene
UP in Hypoxia	**100287569**	LINC00173	1.667624	0.8748864	3.1769088	long intergenic non-protein coding RNA 173
UP in Hypoxia	**23531**	MMD	1.14189	0.86272	2.2066999	monocyte to macrophage differentiation-associated
UP in Hypoxia	**2355**	FOSL2	1.28164	0.8937758	2.4311512	FOS-like antigen 2
UP in Hypoxia	**147650**	LINC00085	1.184942	0.8226414	2.2735426	long intergenic non-protein coding RNA 85
UP in Hypoxia	**2335**	FN1	4.679773	0.9916078	25.630197	fibronectin 1
UP in Hypoxia	**6515**	SLC2A3	2.16902	0.9384169	4.4971779	solute carrier family 2 (facilitated glucose transporter), member 3
UP in Hypoxia	**6237**	RRAS	1.088775	0.8706115	2.1269336	related RAS viral (r-ras) oncogene homolog
UP in Hypoxia	**441058**	MGC39584	1.315528	0.8153156	2.4889336	uncharacterized LOC441058
UP in Hypoxia	**23559**	WBP1	1.429042	0.8999425	2.692679	WW domain binding protein 1

UP in Hypoxia	**126868**	MAB21L	1.145689	0.8185612	2.2125182	mab-21-like 3 (C. elegans)
UP in Hypoxia	**23030**	KDM4B	1.187477	0.8819804	2.2775416	lysine (K)-specific demethylase 4B
UP in Hypoxia	**92092**	ZC3HAV 1L	1.085478	0.8694616	2.1220792	zinc finger CCCH-type, antiviral 1-like
UP in Hypoxia	**1195**	CLK1	1.320887	0.893442	2.4981971	CDC-like kinase 1
UP in Hypoxia	**731656**	LOC731 656	3.201634	0.8626829	9.2	uncharacterized LOC731656
UP in Hypoxia	**4237**	MFAP2	1.022141	0.8781969	2.0309309	microfibrillar-associated protein 2
UP in Hypoxia	**9514**	GAL3ST	5.83289	0.9093919	57	galactose-3-O-sulfotransferase 1
UP in Hypoxia	**6452**	SH3BP2	1.297481	0.8757674	2.4579926	SH3-domain binding protein 2
UP in Hypoxia	**23670**	TMEM2	1.036059	0.8580834	2.050618	transmembrane protein 2
UP in Hypoxia	**347733**	TUBB2B	3.474602	0.814685	11.116279	tubulin, beta 2B class IIb
UP in Hypoxia	**149478**	BTBD19	1.847211	0.8471967	3.5980392	BTB (POZ) domain containing 19
UP in Hypoxia	**100506211**	MIR210 HG	4.524822	0.9873885	23.020089	MIR210 host gene (non-protein coding)
UP in Hypoxia	**3725**	JUN	2.466223	0.9339843	5.5259516	jun proto-oncogene
UP in Hypoxia	**388**	RHOB	1.075543	0.8858844	2.1075152	ras homolog family member B
UP in Hypoxia	**9180**	OSMR	1.535166	0.9006194	2.8982169	oncostatin M receptor
UP in Hypoxia	**283212**	KLHL35	1.7046	0.8239582	3.2593857	kelch-like family member 35
UP in Hypoxia	**9315**	NREP	1.452805	0.901148	2.7373978	neuronal regeneration related protein
UP in Hypoxia	**643401**	LOC643 401	1.154328	0.8493759	2.2258065	uncharacterized LOC643401
UP in Hypoxia	**168667**	BMPER	4.400538	0.8520002	21.12	BMP binding endothelial regulator
UP in Hypoxia	**1382**	CRABP2	1.030953	0.8828057	2.0433736	cellular retinoic acid binding protein 2
UP in Hypoxia	**3691**	ITGB4	1.5736	0.8992563	2.9764652	integrin, beta 4
UP in Hypoxia	**255394**	TCP11L	1.912075	0.8930247	3.7635009	t-complex 11, testis-specific-like 2
UP in Hypoxia	**9976**	CLEC2B	1.663292	0.914047	3.1673838	C-type lectin domain family 2, member B
UP in Hypoxia	**5210**	PFKFB4	3.775807	0.9793672	13.697179	6-phosphofructo-2-kinase/fructose-2,6-biphosphatase 4
UP in Hypoxia	**4205**	MEF2A	1.02174	0.8659842	2.0303669	myocyte enhancer factor 2A
UP in Hypoxia	**639**	PRDM1	1.826159	0.8675884	3.5459184	PR domain containing 1, with ZNF domain
UP in Hypoxia	**55344**	PLCXD1	1.515239	0.8999703	2.8584627	phosphatidylinositol-specific phospholipase C, X domain containing 1
UP in Hypoxia	**10509**	SEMA4B	1.263958	0.89334	2.4015362	sema domain, immunoglobulin domain (Ig), transmembrane domain (TM) and short cytoplasmic domain, (semaphorin) 4B
UP in Hypoxia	**4582**	MUC1	1.390111	0.9008142	2.6209878	mucin 1, cell surface associated
UP in Hypoxia	**6281**	S100A10	1.10949	0.8882305	2.1576932	S100 calcium binding protein A10
UP in Hypoxia	**8793**	TNFRSF 10D	1.247414	0.8672268	2.3741554	tumor necrosis factor receptor superfamily, member 10d, decoy with truncated death domain
UP in Hypoxia	**23650**	TRIM29	1.818787	0.9040412	3.527845	tripartite motif containing 29
UP in Hypoxia	**51129**	ANGPTL	5.032379	0.9902168	32.726316	angiopoietin-like 4
UP in Hypoxia	**5129**	CDK18	1.936465	0.9164766	3.8276662	cyclin-dependent kinase 18
UP in Hypoxia	**283131**	NEAT1	1.345455	0.9009347	2.541104	nuclear paraspeckle assembly transcript 1 (non-protein coding)
UP in Hypoxia	**3489**	IGFBP6	1.395836	0.8467423	2.6314103	insulin-like growth factor binding protein 6
UP in Hypoxia	**353322**	ANKRD 37	2.702858	0.9309798	6.5109034	ankyrin repeat domain 37
UP in Hypoxia	**100507057**	MFI2-	1.44944	0.8268328	2.7310195	MFI2 antisense RNA 1
UP in Hypoxia	**4783**	NFIL3	1.020329	0.8685714	2.0283819	nuclear factor, interleukin 3 regulated
UP in Hypoxia	**8496**	PPFIBP1	1.041136	0.8799125	2.0578471	PTPRF interacting protein, binding protein 1 (liprin beta 1)
UP in Hypoxia	**901**	CCNG2	2.449244	0.940865	5.4612971	cyclin G2
UP in Hypoxia	**90271**	LINC002 63	1.586538	0.8947403	3.0032787	long intergenic non-protein coding RNA 263
UP in Hypoxia	**5066**	PAM	2.091004	0.9337154	4.2604433	peptidylglycine alpha-amidating

UP in Hypoxia	**285966**	FAM115	4.104796	0.9401973	17.205479	family with sequence similarity 115, member
UP in Hypoxia	**1809**	DPYSL3	1.080476	0.8230494	2.1147343	dihydropyrimidinase-like 3
UP in Hypoxia	**1400**	CRMP1	1.161751	0.8152043	2.2372881	collapsin response mediator protein 1
UP in Hypoxia	**5919**	RARRES 2	1.599601	0.9099575	3.0305958	retinoic acid receptor responder (tazarotene induced) 2
UP in Hypoxia	**11190**	CEP250	1.084637	0.8712328	2.1208421	centrosomal protein 250kDa
UP in Hypoxia	**100505648**	LOC100 505648	1.13465	0.8427549	2.1956522	uncharacterized LOC100505648
UP in Hypoxia	**26577**	PCOLCE	1.237263	0.8878225	2.357508	procollagen C-endopeptidase enhancer 2
UP in Hypoxia	**3576**	IL8	4.431553	0.8083051	21.578947	interleukin 8
UP in Hypoxia	**248**	ALPI	1.787931	0.9094939	3.4531934	alkaline phosphatase, intestinal
UP in Hypoxia	**3269**	HRH1	2.673705	0.9412081	6.3806584	histamine receptor H1
UP in Hypoxia	**2632**	GBE1	1.560351	0.9043565	2.9492549	glucan (1,4-alpha-), branching enzyme 1
UP in Hypoxia	**2118**	ETV4	1.14246	0.8272223	2.2075718	ets variant 4
UP in Hypoxia	**1292**	COL6A2	3.288519	0.8832601	9.7710843	collagen, type VI, alpha 2
UP in Hypoxia	**1356**	CP	2.588867	0.9136575	6.0162602	ceruloplasmin (ferroxidase)
UP in Hypoxia	**784**	CACNB3	1.101843	0.8721694	2.1462872	calcium channel, voltage-dependent, beta 3 subunit
UP in Hypoxia	**205**	AK4	2.365179	0.9456129	5.1521649	adenylate kinase 4
UP in Hypoxia	**1026**	CDKN1	1.96237	0.9211781	3.8970164	cyclin-dependent kinase inhibitor 1A (p21,
UP in Hypoxia	**4092**	SMAD7	1.017922	0.8419388	2.025	SMAD family member 7
UP in Hypoxia	**221416**	C6orf223	1.550263	0.8526308	2.9287054	chromosome 6 open reading frame 223
UP in Hypoxia	**3777**	KCNK3	2.524885	0.9012408	5.7552743	potassium channel, subfamily K, member 3
UP in Hypoxia	**6273**	S100A2	1.135448	0.8385356	2.196868	S100 calcium binding protein A2
UP in Hypoxia	**10628**	TXNIP	1.394935	0.90176	2.6297666	thioredoxin interacting protein
UP in Hypoxia	**768**	CA9	5.595117	0.9961331	48.339049	carbonic anhydrase IX
UP in Hypoxia	**54206**	ERRFI1	2.525954	0.9497302	5.759543	ERBB receptor feedback inhibitor 1
UP in Hypoxia	**7138**	TNNT1	1.177065	0.8788553	2.2611637	troponin T type 1 (skeletal, slow)
UP in Hypoxia	**6781**	STC1	2.241739	0.9364324	4.7296669	stanniocalcin 1
UP in Hypoxia	**1999**	ELF3	1.743625	0.9077598	3.3487544	E74-like factor 3 (ets domain transcription factor, epithelial-specific)
UP in Hypoxia	**1466**	CSRP2	1.201751	0.8878317	2.3001863	cysteine and glycine-rich protein 2
UP in Hypoxia	**694**	BTG1	1.043713	0.8751646	2.0615269	B-cell translocation gene 1, anti-proliferative
UP in Hypoxia	**6572**	SLC18A 3	7.996238	0.916968	255.33333	solute carrier family 18 (vesicular acetylcholine), member 3
UP in Hypoxia	**25907**	TMEM1 58	1.535412	0.9009904	2.8987121	transmembrane protein 158 (gene/pseudogene)
UP in Hypoxia	**664**	BNIP3	3.216382	0.9704557	9.2945278	BCL2/adenovirus E1B 19kDa interacting
UP in Hypoxia	**10865**	ARID5A	2.070389	0.8287895	4.2	AT rich interactive domain 5A (MRF1-like)
UP in Hypoxia	**79816**	TLE6	2.034243	0.9064801	4.0960784	transducin-like enhancer of split 6 (E(sp1) homolog, Drosophila)
UP in Hypoxia	**387849**	REP15	1.20427	0.8180233	2.3042071	RAB15 effector protein
UP in Hypoxia	**5143**	PDE4C	2.888969	0.9507224	7.4074074	phosphodiesterase 4C, cAMP-specific
UP in Hypoxia	**219699**	UNC5B	1.203321	0.884716	2.3026921	unc-5 homolog B (C. elegans)
UP in Hypoxia	**94241**	TP53INP	2.1802	0.8288729	4.5321637	tumor protein p53 inducible nuclear protein 1
UP in Hypoxia	**56937**	PMEPA1	1.997446	0.9318051	3.9929245	prostate transmembrane protein, androgen induced 1
UP in Hypoxia	**7103**	TSPAN8	1.058166	0.855728	2.0822827	tetraspanin 8
UP in Hypoxia	**857**	CAV1	1.022846	0.8822864	2.0319233	caveolin 1, caveolae protein, 22kDa
UP in Hypoxia	**133**	ADM	5.237929	0.9921271	37.737542	adrenomedullin
UP in Hypoxia	**57799**	RAB40C	1.049085	0.8758044	2.0692172	RAB40C, member RAS oncogene family
UP in Hypoxia	**90673**	PPP1R3	1.546634	0.8800701	2.9213483	protein phosphatase 1, regulatory subunit 3E
UP in Hypoxia	**6535**	SLC6A8	1.237684	0.8953523	2.3581974	solute carrier family 6 (neurotransmitter transporter, creatine), member 8
UP in Hypoxia	**1363**	CPE	1.579271	0.8633042	2.988189	carboxypeptidase E
UP in Hypoxia	**8646**	CHRD	2.858505	0.8499601	7.2526316	chordin

UP in Hypoxia	**100131213**	ZNF503-AS2	1.869221	0.901454	3.6533524	ZNF503 antisense RNA 2
UP in Hypoxia	**5742**	PTGS1	2.249349	0.9345685	4.7546838	prostaglandin-endoperoxide synthase 1 (prostaglandin G/H synthase and cyclooxygenase)
UP in Hypoxia	**57801**	HES4	1.116793	0.8704075	2.1686436	hairy and enhancer of split 4 (Drosophila)
UP in Hypoxia	**7422**	VEGFA	1.857787	0.9239044	3.624513	vascular endothelial growth factor A
UP in Hypoxia	**5365**	PLXNB3	2.014308	0.8728185	4.0398671	plexin B3
UP in Hypoxia	**23462**	HEY1	1.506892	0.9045513	2.8419704	hairy/enhancer-of-split related with YRPW
UP in Hypoxia	**3726**	JUNB	1.208258	0.8874794	2.3105846	jun B proto-oncogene
UP in Hypoxia	**23036**	ZNF292	1.008569	0.837497	2.0119149	zinc finger protein 292
UP in Hypoxia	**148229**	ATP8B3	1.181526	0.8067564	2.2681661	ATPase, aminophospholipid transporter, class I, type 8B, member 3
UP in Hypoxia	**143666**	LOC143666	1.487081	0.8398616	2.8032129	uncharacterized LOC143666
UP in Hypoxia	**285512**	FAM13A-AS1	1.916956	0.8825739	3.7762557	FAM13A antisense RNA 1
UP in Hypoxia	**2119**	ETV5	1.056619	0.8141842	2.0800508	ets variant 5
UP in Hypoxia	**5211**	PFKL	1.406987	0.9012222	2.6518273	phosphofructokinase, liver
UP in Hypoxia	**11247**	NXPH4	2.857191	0.9620356	7.2460317	neurexophilin 4
UP in Hypoxia	**126075**	CCDC15	1.073522	0.8019622	2.1045655	coiled-coil domain containing 159
UP in Hypoxia	**55779**	WDR52	1.538602	0.8226506	2.9051282	WD repeat domain 52
UP in Hypoxia	**26118**	WSB1	1.806276	0.9236818	3.4973829	WD repeat and SOCS box containing 1
UP in Hypoxia	**90226**	UCN2	2.075844	0.9189989	4.2159091	urocortin 2
UP in Hypoxia	**112399**	EGLN3	3.652982	0.9778928	12.579321	egl nine homolog 3 (C. elegans)
UP in Hypoxia	**113177**	IZUMO4	2.611115	0.8840576	6.1097561	IZUMO family member 4
UP in Hypoxia	**63920**	C5orf54	1.167524	0.8285947	2.2462585	chromosome 5 open reading frame 54
UP in Hypoxia	**386757**	SLC6A10P	1.513431	0.8153156	2.8548813	solute carrier family 6 (neurotransmitter transporter, creatine), member 10, pseudogene
UP in Hypoxia	**1953**	MEGF6	2.110228	0.8576105	4.3175966	multiple EGF-like-domains 6
UP in Hypoxia	**378825**	LINC00162	1.008528	0.8015356	2.0118577	long intergenic non-protein coding RNA 162
UP in Hypoxia	**10603**	SH2B2	1.255164	0.8285855	2.3869427	SH2B adaptor protein 2
UP in Hypoxia	**54437**	SEMA5B	6.738092	0.8313211	106.75	sema domain, seven thrombospondin repeats (type 1 and type 1-like), transmembrane domain (TM) and short cytoplasmic domain, (semaphorin) 5B
UP in Hypoxia	**3486**	IGFBP3	4.765868	0.9925166	27.206276	insulin-like growth factor binding protein 3
UP in Hypoxia	**222962**	SLC29A4	4.007694	0.9702239	16.085561	solute carrier family 29 (nucleoside transporters), member 4
UP in Hypoxia	**3868**	KRT16	4.42803	0.8083051	21.526316	keratin 16
UP in Hypoxia	**84808**	C1orf170	4.190212	0.9188505	18.254902	chromosome 1 open reading frame 170
UP in Hypoxia	**79987**	SVEP1	1.103425	0.8835475	2.1486424	sushi, von Willebrand factor type A, EGF and pentraxin domain containing 1
UP in Hypoxia	**51760**	SYT17	1.643904	0.904542	3.1251029	synaptotagmin XVII
UP in Hypoxia	**339105**	PRSS53	1.247068	0.8098908	2.3735849	protease, serine, 53
UP in Hypoxia	**90338**	ZNF160	1.275507	0.8443406	2.420839	zinc finger protein 160
UP in Hypoxia	**5033**	P4HA1	2.902995	0.9625271	7.4797746	prolyl 4-hydroxylase, alpha polypeptide I
UP in Hypoxia	**100288615**	WHAMMP1	1.150657	0.8325729	2.2201493	WAS protein homolog associated with actin, golgi membranes and microtubules pseudogene 1
UP in Hypoxia	**81606**	LBH	2.056755	0.8033995	4.1604938	limb bud and heart development
UP in Hypoxia	**81855**	SFXN3	1.068752	0.8833528	2.0976186	sideroflexin 3
UP in Hypoxia	**27344**	PCSK1N	1.121807	0.8671433	2.1761933	proprotein convertase subtilisin/kexin type 1 inhibitor
UP in Hypoxia	**340206**	TREML3P	5.338006	0.9476251	40.448276	triggering receptor expressed on myeloid cells-like 3, pseudogene

UP in Hypoxia	**4601**	MXI1	2.125202	0.9346427	4.3626417	MAX interactor 1, dimerization protein
UP in Hypoxia	**148898**	C1orf213	1.610762	0.8313952	3.0541311	chromosome 1 open reading frame 213
UP in Hypoxia	**123**	PLIN2	2.544663	0.9497394	5.8347181	perilipin 2
UP in Hypoxia	**100506123**	LOC100 506123	1.149773	0.8299208	2.2187902	uncharacterized LOC100506123
UP in Hypoxia	**84058**	WDR54	1.206253	0.8915688	2.3073751	WD repeat domain 54
UP in Hypoxia	**80727**	TTYH3	1.923102	0.9261485	3.7923747	tweety homolog 3 (Drosophila)
UP in Hypoxia	**7466**	WFS1	1.214432	0.8791057	2.3204938	Wolfram syndrome 1 (wolframin)
UP in Hypoxia	**638**	BIK	2.178053	0.8335466	4.5254237	BCL2-interacting killer (apoptosis-inducing)
UP in Hypoxia	**6676**	SPAG4	4.176935	0.9812589	18.087671	sperm associated antigen 4
UP in Hypoxia	**8913**	CACNA 1G	2.486132	0.8528626	5.6027397	calcium channel, voltage-dependent, T type, alpha 1G subunit
UP in Hypoxia	**10144**	FAM13A	1.52196	0.8944157	2.8718101	family with sequence similarity 13, member A
UP in Hypoxia	**134265**	AFAP1L	1.43206	0.8680799	2.6983173	actin filament associated protein 1-like 1
UP in Hypoxia	**9149**	DYRK1 B	1.126559	0.8299764	2.1833741	dual-specificity tyrosine-(Y)-phosphorylation regulated kinase 1B
UP in Hypoxia	**27345**	KCNMB 4	2.226509	0.8167715	4.68	potassium large conductance calcium-activated channel, subfamily M, beta member 4
UP in Hypoxia	**1944**	EFNA3	2.181775	0.9280124	4.5371134	ephrin-A3
UP in Hypoxia	**100499467**	LINC006 73	2.437532	0.8688403	5.4171429	long intergenic non-protein coding RNA 673
UP in Hypoxia	**9063**	PIAS2	1.044623	0.848393	2.0628272	protein inhibitor of activated STAT, 2
UP in Hypoxia	**284836**	LINC003 19	1.516629	0.8479386	2.8612167	long intergenic non-protein coding RNA 319
UP in Hypoxia	**55893**	ZNF395	2.306894	0.9276136	4.9481669	zinc finger protein 395
UP in Hypoxia	**10318**	TNIP1	1.150425	0.8881749	2.219793	TNFAIP3 interacting protein 1
UP in Hypoxia	**968**	CD68	1.135797	0.8690536	2.1973995	CD68 molecule
UP in Hypoxia	**54583**	EGLN1	1.635337	0.9150114	3.1066007	egl nine homolog 1 (C. elegans)
UP in Hypoxia	**168544**	ZNF467	1.183292	0.8818135	2.2709434	zinc finger protein 467
UP in Hypoxia	**57007**	CXCR7	1.160701	0.8771305	2.2356603	chemokine (C-X-C motif) receptor 7
UP in Hypoxia	**387841**	RPL13A P20	2.199741	0.9076207	4.5939675	ribosomal protein L13a pseudogene 20
UP in Hypoxia	**5266**	PI3	3.470862	0.8999796	11.0875	peptidase inhibitor 3, skin-derived
UP in Hypoxia	**7436**	VLDLR	1.879615	0.9170793	3.679768	very low density lipoprotein receptor
UP in Hypoxia	**2934**	GSN	1.319301	0.8969287	2.4954524	gelsolin
UP in Hypoxia	**3039**	HBA1	1.982421	0.8563864	3.9515571	hemoglobin, alpha 1
UP in Hypoxia	**23135**	KDM6B	1.731085	0.9012593	3.319774	lysine (K)-specific demethylase 6B
UP in Hypoxia	**5228**	PGF	2.629528	0.8890836	6.1882353	placental growth factor
UP in Hypoxia	**401232**	DKFZP6 86I15217	2.17657	0.8979859	4.5207756	uncharacterized LOC401232
UP in Hypoxia	**57326**	PBXIP1	1.090628	0.8778167	2.1296671	pre-B-cell leukemia homeobox interacting
UP in Hypoxia	**50651**	SLC45A	4.367779	0.9264545	20.645833	solute carrier family 45, member 1
UP in Hypoxia	**5540**	NPY4R	2.759888	0.8713626	6.7734375	neuropeptide Y receptor Y4
UP in Hypoxia	**3097**	HIVEP2	1.231119	0.8043732	2.3474903	human immunodeficiency virus type I enhancer binding protein 2
UP in Hypoxia	**3778**	KCNMA 1	2.469803	0.8323133	5.5396825	potassium large conductance calcium-activated channel, subfamily M, alpha member 1
UP in Hypoxia	**169611**	OLFML2 A	1.372287	0.8975871	2.588806	olfactomedin-like 2A
UP in Hypoxia	**100505678**	STARD4 -AS1	1.472896	0.8268607	2.7757848	STARD4 antisense RNA 1
UP in Hypoxia	**8714**	ABCC3	1.214542	0.8835846	2.3206704	ATP-binding cassette, sub-family C (CFTR/MRP), member 3
UP in Hypoxia	**51646**	YPEL5	1.392564	0.8836866	2.6254493	yippee-like 5 (Drosophila)

UP in Hypoxia	**100216545**	LOC100 216545	1.267435	0.8040672	2.407332	uncharacterized LOC100216545
UP in Hypoxia	**347735**	SERINC	1.063911	0.8759992	2.0905914	serine incorporator 2
UP in Hypoxia	**9052**	GPRC5A	1.65768	0.9203898	3.1550873	G protein-coupled receptor, family C, group 5, member A
UP in Hypoxia	**665**	BNIP3L	2.456524	0.9481908	5.4889267	BCL2/adenovirus E1B 19kDa interacting protein 3-like
UP in Hypoxia	**1028**	CDKN1	1.053239	0.8818413	2.0751832	cyclin-dependent kinase inhibitor 1C (p57,
UP in Hypoxia	**65989**	DLK2	1.500537	0.8026484	2.8294798	delta-like 2 homolog (Drosophila)
UP in Hypoxia	**27076**	LYPD3	1.567962	0.8830932	2.9648562	LY6/PLAUR domain containing 3
UP in Hypoxia	**54210**	TREM1	6.635174	0.964187	99.4	triggering receptor expressed on myeloid cells
UP in Hypoxia	**83986**	ITFG3	1.087282	0.8828057	2.1247341	integrin alpha FG-GAP repeat containing 3
UP in Hypoxia	**205428**	C3orf58	1.303702	0.8707969	2.4686156	chromosome 3 open reading frame 58
UP in Hypoxia	**1805**	DPT	4.169925	0.8513975	18	dermatopontin
UP in Hypoxia	**84929**	FIBCD1	1.19708	0.8852538	2.2927509	fibrinogen C domain containing 1
UP in Hypoxia	**6662**	SOX9	1.902601	0.8878503	3.7388664	SRY (sex determining region Y)-box 9
UP in Hypoxia	**55076**	TMEM4 5A	2.589345	0.9559803	6.0182547	transmembrane protein 45A
UP in Hypoxia	**85301**	COL27A	1.355265	0.8665684	2.5584416	collagen, type XXVII, alpha 1
UP in Hypoxia	**375295**	LOC375 295	1.686842	0.8923756	3.2195122	uncharacterized LOC375295
UP in Hypoxia	**5155**	PDGFB	3.304898	0.9436562	9.8826531	platelet-derived growth factor beta
UP in Hypoxia	**2970**	GTF2IP1	1.383585	0.8862089	2.6091593	general transcription factor IIi, pseudogene 1
UP in Hypoxia	**8974**	P4HA2	2.207475	0.9382592	4.6186613	prolyl 4-hydroxylase, alpha polypeptide II
UP in Hypoxia	**283248**	RCOR2	1.648921	0.9080565	3.135989	REST corepressor 2
UP in Hypoxia	**6253**	RTN2	1.096546	0.8473915	2.1384206	reticulon 2
UP in Hypoxia	**8497**	PPFIA4	5.423796	0.9893359	42.926471	protein tyrosine phosphatase, receptor type, f polypeptide (PTPRF), interacting protein (liprin), alpha 4
UP in Hypoxia	**9123**	SLC16A 3	1.187626	0.8935069	2.2777764	solute carrier family 16, member 3 (monocarboxylic acid transporter 4)
UP in Hypoxia	**143686**	SESN3	1.099794	0.8677832	2.1432403	sestrin 3
UP in Hypoxia	**89796**	NAV1	1.136605	0.8700736	2.1986301	neuron navigator 1
UP in Hypoxia	**55818**	KDM3A	1.900388	0.9228751	3.7331347	lysine (K)-specific demethylase 3A
UP in Hypoxia	**81544**	GDPD5	1.008197	0.8471967	2.011396	glycerophosphodiester phosphodiesterase domain containing 5
UP in Hypoxia	**6560**	SLC12A 4	1.005097	0.8252564	2.0070779	solute carrier family 12 (potassium/chloride transporters), member 4
UP in Hypoxia	**1290**	COL5A2	1.068098	0.8833899	2.0966667	collagen, type V, alpha 2
UP in Hypoxia	**949**	SCARB1	1.242057	0.8960941	2.3653555	scavenger receptor class B, member 1
UP in Hypoxia	**440584**	SLC2A1-AS1	4.867896	0.8731523	29.2	SLC2A1 antisense RNA 1
UP in Hypoxia	**26220**	DGCR5	2.37277	0.8673937	5.1793478	DiGeorge syndrome critical region gene 5 (non-protein coding)
UP in Hypoxia	**5352**	PLOD2	2.358695	0.9486545	5.1290627	procollagen-lysine, 2-oxoglutarate 5-
UP in Hypoxia	**85409**	NKD2	1.795513	0.8587604	3.4713896	naked cuticle homolog 2 (Drosophila)
UP in Hypoxia	**1907**	EDN2	4.512112	0.9773364	22.818182	endothelin 2
UP in Hypoxia	**6590**	SLPI	1.421769	0.9034199	2.6791377	secretory leukocyte peptidase inhibitor
UP in Hypoxia	**1634**	DCN	1.448712	0.8516107	2.7296417	decorin
UP in Hypoxia	**284904**	SEC14L4	1.498396	0.865604	2.8252841	SEC14-like 4 (S. cerevisiae)
UP in Hypoxia	**84858**	ZNF503	1.46882	0.9011573	2.7679546	zinc finger protein 503
UP in Hypoxia	**2297**	FOXD1	2.350907	0.877622	5.1014493	forkhead box D1
UP in Hypoxia	**53947**	A4GALT	1.374324	0.8868024	2.5924638	alpha 1,4-galactosyltransferase
UP in Hypoxia	**126308**	MOB3A	1.513533	0.8989039	2.8550841	MOB kinase activator 3A
UP in Hypoxia	**9619**	ABCG1	2.211875	0.8371353	4.6327684	ATP-binding cassette, sub-family G (WHITE), member 1
UP in Hypoxia	**4017**	LOXL2	2.942971	0.961878	7.6899329	lysyl oxidase-like 2

UP in Hypoxia	**3872**	KRT17	2.260496	0.932176	4.7915633	keratin 17
UP in Hypoxia	**11147**	HHLA3	1.146682	0.8010442	2.2140411	HERV-H LTR-associating 3
UP in Hypoxia	**7846**	TUBA1A	1.218467	0.8955656	2.3269927	tubulin, alpha 1a
UP in Hypoxia	**610**	HCN2	1.090676	0.8070254	2.1297376	hyperpolarization activated cyclic nucleotide-gated potassium channel 2
