# Supplementary material for: Integrated analysis identified core signal pathways and hypoxic characteristics of human glioblastoma
Source: J Cell Mol Med. 2019 Jul 7;23(9):6228–37. doi: 10.1111/jcmm.14507 (PMC6714287; doi:10.1111/jcmm.14507)
Supplement: Supplementary file 15 [file JCMM-23-6228-s016.pdf]

Table S8 List of over-represented gene ontology biological process subcategories (GOBPID) in the genes differentially expressed (log2 fold change >1) with statistical significance (BH-corrected p-value <0.05) between U87-MG cultuered in hypoxia versus normoxia.

a. List of gene ontology biological process subcategories in genes up-regulated in U87-MG cultuered in hypoxia versus normoxia

	GOBPID	P value	Count	%	GO Term	Pvalue.BH corrected
1	GO:0001666	1.99E-12	38	0.157676	response to hypoxia	5.497E-09
2	GO:0036293	3.11E-12	38	0.155102	response to decreased oxygen levels	5.497E-09
3	GO:0070482	1.375E-11	38	0.146718	response to oxygen levels	1.623E-08
4	GO:0006090	8.276E-08	16	0.225352	pyruvate metabolic process	6.823E-05
5	GO:0006096	9.637E-08	13	0.295455	glycolytic process	6.823E-05
6	GO:0071456	2.228E-07	18	0.181818	cellular response to hypoxia	0.0001281
7	GO:0006006	2.676E-07	22	0.147651	glucose metabolic process	0.0001281
8	GO:0036294	2.895E-07	18	0.178218	cellular response to decreased oxygen levels	0.0001281
9	GO:0030198	4.069E-07	36	0.100279	extracellular matrix organization	0.0001532
10	GO:0043062	4.326E-07	36	0.1	extracellular structure organization	0.0001532
11	GO:0048545	7.425E-07	36	0.097561	response to steroid hormone	0.00023
12	GO:0071453	7.797E-07	18	0.165138	cellular response to oxygen levels	0.00023
13	GO:1903532	2.711E-06	27	0.109312	positive regulation of secretion by cell	0.0007383
14	GO:0007568	3.101E-06	24	0.117647	aging	0.0007841
15	GO:0071396	3.744E-06	31	0.098413	cellular response to lipid	0.0008836
16	GO:0045765	4.218E-06	22	0.122905	regulation of angiogenesis	0.0009331
17	GO:0051047	5.43E-06	28	0.102564	positive regulation of secretion	0.0011308
18	GO:0019318	7.958E-06	22	0.117647	hexose metabolic process	0.0015651
19	GO:0005996	1.07E-05	24	0.108597	monosaccharide metabolic process	0.0019943
20	GO:1901342	1.674E-05	22	0.111675	regulation of vasculature development	0.0029632
21	GO:0045766	2E-05	15	0.148515	positive regulation of angiogenesis	0.0033714
22	GO:0010035	2.94E-05	32	0.086486	response to inorganic substance	0.0047312
23	GO:0019471	4.1E-05	6	0.461538	4-hydroxyproline metabolic process	0.0060482
24	GO:0019511	4.1E-05	6	0.461538	peptidyl-proline hydroxylation	0.0060482
25	GO:0051051	4.429E-05	31	0.085873	negative regulation of transport	0.0062718
26	GO:0032496	4.705E-05	23	0.100877	response to lipopolysaccharide	0.006406
27	GO:0044724	6.431E-05	15	0.132743	single-organism carbohydrate catabolic process	0.008432
28	GO:0048511	7.472E-05	24	0.095238	rhythmic process	0.009447
29	GO:0002687	9.893E-05	12	0.153846	positive regulation of leukocyte migration	0.0120759
30	GO:0018126	0.0001027	6	0.375	protein hydroxylation	0.0121215
31	GO:0002237	0.0001143	23	0.09465	response to molecule of bacterial origin	0.0130535
32	GO:0016052	0.0001185	15	0.125	carbohydrate catabolic process	0.0131087
33	GO:0051271	0.00015	19	0.103825	negative regulation of cellular component movement	0.015621
34	GO:0006094	0.00015	9	0.195652	gluconeogenesis	0.015621
35	GO:0042493	0.0001656	29	0.081921	response to drug	0.0164425
36	GO:0071222	0.0001672	14	0.127273	cellular response to lipopolysaccharide	0.0164425
37	GO:0010038	0.0002195	23	0.090196	response to metal ion	0.020886
38	GO:0042326	0.0002242	27	0.083077	negative regulation of phosphorylation	0.020886
39	GO:0032787	0.0002311	35	0.073996	monocarboxylic acid metabolic process	0.0209768
40	GO:0050678	0.0002565	23	0.089147	regulation of epithelial cell proliferation	0.0221492
41	GO:0019319	0.0002606	9	0.18	hexose biosynthetic process	0.0221492
42	GO:0016051	0.0002628	15	0.115385	carbohydrate biosynthetic process	0.0221492
43	GO:0071219	0.0002754	14	0.12069	cellular response to molecule of bacterial origin	0.0226713
44	GO:0071216	0.0003052	15	0.113636	cellular response to biotic stimulus	0.0245514
45	GO:0044057	0.0003435	29	0.078167	regulation of system process	0.0270194
46	GO:0001541	0.0003543	8	0.195122	ovarian follicle development	0.0271164
47	GO:0046887	0.00036	12	0.131868	positive regulation of hormone secretion	0.0271164
48	GO:1903531	0.0003797	15	0.111111	negative regulation of secretion by cell	0.0280029

49	GO:0051048	0.0004257	16	0.105263	negative regulation of secretion	0.0304602
50	GO:0009755	0.0004303	12	0.129032	hormone-mediated signaling pathway	0.0304602
51	GO:0001822	0.0004388	14	0.114754	kidney development	0.0304602
52	GO:0050873	0.0004554	7	0.21875	brown fat cell differentiation	0.0310054
53	GO:0040013	0.0005281	19	0.093137	negative regulation of locomotion	0.0352708
54	GO:0046364	0.0005465	9	0.160714	monosaccharide biosynthetic process	0.0358239
55	GO:2001237	0.0006058	12	0.123711	negative regulation of extrinsic apoptotic signaling pathway	0.0389936
56	GO:0002009	0.000679	24	0.081081	morphogenesis of an epithelium	0.0428924
57	GO:0009612	0.0007027	17	0.096591	response to mechanical stimulus	0.0428924
58	GO:0050679	0.0007039	15	0.104167	positive regulation of epithelial cell proliferation	0.0428924
59	GO:2001240	0.000727	7	0.2	negative regulation of extrinsic apoptotic signaling pathway in absence of ligand	0.0428924
60	GO:1901099	0.000727	7	0.2	negative regulation of signal transduction in absence of ligand	0.0428924
61	GO:0043401	0.000765	9	0.152542	steroid hormone mediated signaling pathway	0.0437097
62	GO:0051346	0.0007704	27	0.076271	negative regulation of hydrolase activity	0.0437097
63	GO:0032846	0.00078	14	0.107692	positive regulation of homeostatic process	0.0437097
64	GO:0035914	0.0007902	8	0.170213	skeletal muscle cell differentiation	0.0437097
65	GO:0046883	0.0008459	20	0.086957	regulation of hormone secretion	0.0455997
66	GO:0022617	0.0008502	13	0.112069	extracellular matrix disassembly	0.0455997
67	GO:0014074	0.0008938	14	0.106061	response to purine-containing compound	0.0472249
68	GO:0001936	0.0009367	11	0.125	regulation of endothelial cell proliferation	0.0487624

49	GO:0051048	0.0004257	16	0.105263	negative regulation of secretion	0.0304602
50	GO:0009755	0.0004303	12	0.129032	hormone-mediated signaling pathway	0.0304602
51	GO:0001822	0.0004388	14	0.114754	kidney development	0.0304602
52	GO:0050873	0.0004554	7	0.21875	brown fat cell differentiation	0.0310054
53	GO:0040013	0.0005281	19	0.093137	negative regulation of locomotion	0.0352708
54	GO:0046364	0.0005465	9	0.160714	monosaccharide biosynthetic process	0.0358239
55	GO:2001237	0.0006058	12	0.123711	negative regulation of extrinsic apoptotic signaling pathway	0.0389936
56	GO:0002009	0.000679	24	0.081081	morphogenesis of an epithelium	0.0428924
57	GO:0009612	0.0007027	17	0.096591	response to mechanical stimulus	0.0428924
58	GO:0050679	0.0007039	15	0.104167	positive regulation of epithelial cell proliferation	0.0428924
59	GO:2001240	0.000727	7	0.2	negative regulation of extrinsic apoptotic signaling pathway in absence of ligand	0.0428924
60	GO:1901099	0.000727	7	0.2	negative regulation of signal transduction in absence of ligand	0.0428924
61	GO:0043401	0.000765	9	0.152542	steroid hormone mediated signaling pathway	0.0437097
62	GO:0051346	0.0007704	27	0.076271	negative regulation of hydrolase activity	0.0437097
63	GO:0032846	0.00078	14	0.107692	positive regulation of homeostatic process	0.0437097
64	GO:0035914	0.0007902	8	0.170213	skeletal muscle cell differentiation	0.0437097
65	GO:0046883	0.0008459	20	0.086957	regulation of hormone secretion	0.0455997
66	GO:0022617	0.0008502	13	0.112069	extracellular matrix disassembly	0.0455997
67	GO:0014074	0.0008938	14	0.106061	response to purine-containing compound	0.0472249
68	GO:0001936	0.0009367	11	0.125	regulation of endothelial cell proliferation	0.0487624

b. List of gene ontology biological process subcategories in genes up-regulated in U87-MG cultuered in hypoxia versus normoxia after processing with REViGO.

Term_ID	Description	Uniqueness	Dispensability
GO:0043062	extracellular structure organization	0.89	0.37
GO:0030198	extracellular matrix organization	0.874	0
GO:0032787	monocarboxylic acid metabolic process	0.804	0.212
GO:0019511	peptidyl-proline hydroxylation	0.907	0.08
GO:0018126	protein hydroxylation	0.906	0.272
GO:0005996	monosaccharide metabolic process	0.748	0.019
GO:2001237	negative regulation of extrinsic apoptotic signaling pathway	0.712	0.43
GO:0042326	negative regulation of phosphorylation	0.784	0.07
GO:0010038	response to metal ion	0.779	0.475
GO:0050678	regulation of epithelial cell proliferation	0.827	0.176
GO:0051047	positive regulation of secretion	0.803	0.019
GO:0010035	response to inorganic substance	0.772	0.5
GO:0048545	response to steroid hormone	0.713	0.084
GO:0071216	cellular response to biotic stimulus	0.785	0.074
GO:0001666	response to hypoxia	0.789	0
GO:0032846	positive regulation of homeostatic process	0.901	0.153
GO:0051346	negative regulation of hydrolase activity	0.897	0.29
GO:0048511	rhythmic process	0.97	0
GO:0050873	brown fat cell differentiation	0.852	0.298
GO:0001541	ovarian follicle development	0.866	0.348
GO:0007568	aging	0.876	0.019
GO:0001822	kidney development	0.855	0.423
GO:0045765	regulation of angiogenesis	0.8	0.337
GO:0071456	cellular response to hypoxia	0.657	0.877
GO:0071453	cellular response to oxygen levels	0.66	0.874
GO:0070482	response to oxygen levels	0.814	0.736
GO:0036293	response to decreased oxygen levels	0.789	0.981
GO:0036294	cellular response to decreased oxygen levels	0.656	0.986
GO:0009612	response to mechanical stimulus	0.767	0.703
GO:0022617	extracellular matrix disassembly	0.881	0.872
GO:0002687	positive regulation of leukocyte migration	0.769	0.508
GO:0040013	negative regulation of locomotion	0.841	0.712
GO:0046887	positive regulation of hormone secretion	0.732	0.874
GO:0046883	regulation of hormone secretion	0.725	0.816
GO:0051271	negative regulation of cellular component movement	0.748	0.701
GO:0051048	negative regulation of secretion	0.773	0.785
GO:0051051	negative regulation of transport	0.786	0.654
GO:0044724	single-organism carbohydrate catabolic process	0.749	0.748
GO:0046364	monosaccharide biosynthetic process	0.746	0.856
GO:0016052	carbohydrate catabolic process	0.793	0.661
GO:0006006	glucose metabolic process	0.734	0.757
GO:0016051	carbohydrate biosynthetic process	0.754	0.756
GO:0019318	hexose metabolic process	0.729	0.952
GO:0019319	hexose biosynthetic process	0.743	0.977
GO:0006094	gluconeogenesis	0.745	0.854
GO:0006096	glycolytic process	0.685	0.953
GO:0071222	cellular response to lipopolysaccharide	0.653	0.907
GO:0009755	hormone-mediated signaling pathway	0.642	0.777
GO:0071396	cellular response to lipid	0.681	0.851

GO:0042493	response to drug	0.769	0.509
GO:0002237	response to molecule of bacterial origin	0.7	0.914
GO:0043401	steroid hormone mediated signaling pathway	0.628	0.766
GO:0071219	cellular response to molecule of bacterial origin	0.679	0.979
GO:0014074	response to purine-containing compound	0.757	0.747
GO:0032496	response to lipopolysaccharide	0.681	0.808
GO:0001936	regulation of endothelial cell proliferation	0.839	0.842
GO:0050679	positive regulation of epithelial cell proliferation	0.812	0.901
GO:0019471	4-hydroxyproline metabolic process	0.836	0.553
GO:0006090	pyruvate metabolic process	0.811	0.696
GO:0045766	positive regulation of angiogenesis	0.795	0.931
GO:0035914	skeletal muscle cell differentiation	0.824	0.548
GO:0044057	regulation of system process	0.849	0.538
GO:1901342	regulation of vasculature development	0.809	0.755
GO:0002009	morphogenesis of an epithelium	0.854	0.524
GO:2001240	negative regulation of extrinsic apoptotic signaling pathway in absence of ligand	0.72	0.855
GO:1901099	negative regulation of signal transduction in absence of ligand	0.727	0.872

c. List of gene ontology biological process subcategories in genes down-regulated in U87-MG cultuered in hypoxia versus normoxia

	GOBPID	P value	Count	%	GO Term	Pvalue.BH corrected
1	GO:0000278	0.00E+00	195	0.4826733	mitotic cell cycle	0.00E+00
2	GO:0006281	0.00E+00	152	0.3771712	DNA repair	0.00E+00
3	GO:0034660	0.00E+00	132	0.3975904	ncRNA metabolic process	0.00E+00
4	GO:0034470	0.00E+00	105	0.4356846	ncRNA processing	0.00E+00
5	GO:0044770	0.00E+00	108	0.3857143	cell cycle phase transition	0.00E+00
6	GO:0007067	0.00E+00	98	0.4083333	mitotic nuclear division	0.00E+00
7	GO:0044772	0.00E+00	106	0.3826715	mitotic cell cycle phase transition	0.00E+00
8	GO:0048285	0.00E+00	116	0.3569231	organelle fission	0.00E+00
9	GO:0000280	0.00E+00	110	0.3691275	nuclear division	0.00E+00
10	GO:0006260	0.00E+00	81	0.4354839	DNA replication	0.00E+00
11	GO:0051276	0.00E+00	83	0.4068627	chromosome organization	1E-14
12	GO:0000082	0.00E+00	70	0.4605263	G1/S transition of mitotic cell cycle	2E-14
13	GO:0044843	0.00E+00	70	0.4605263	cell cycle G1/S phase transition	2E-14
14	GO:0006310	0.00E+00	78	0.3979592	DNA recombination	2.2E-13
15	GO:0016072	3E-14	58	0.4603175	rRNA metabolic process	8.76E-12
16	GO:0006364	5E-14	56	0.4666667	rRNA processing	1.462E-11
17	GO:0022616	7.9E-13	30	0.8571429	DNA strand elongation	2.009E-10
18	GO:0006271	1.24E-12	29	0.8787879	DNA strand elongation involved in DNA replication	2.991E-10
19	GO:0009123	1.34E-12	115	0.2693208	nucleoside monophosphate metabolic process	3.056E-10
20	GO:0010564	2.16E-12	124	0.2577963	regulation of cell cycle process	4.683E-10
21	GO:0006302	2.51E-12	51	0.4473684	double-strand break repair	5.169E-10
22	GO:0009161	2.71E-12	112	0.2692308	ribonucleoside monophosphate metabolic process	5.336E-10
23	GO:0006399	3.99E-12	58	0.3972603	tRNA metabolic process	7.511E-10
24	GO:1901990	4.61E-12	80	0.3187251	regulation of mitotic cell cycle phase transition	8.309E-10
25	GO:0000075	5.03E-12	60	0.3846154	cell cycle checkpoint	8.708E-10
26	GO:1901987	1.66E-11	81	0.3068182	regulation of cell cycle phase transition	2.755E-09
27	GO:0007059	1.9E-11	43	0.4831461	chromosome segregation	3.051E-09
28	GO:1901991	2.49E-11	66	0.34375	negative regulation of mitotic cell cycle phase transition	3.842E-09
29	GO:0009167	3.02E-11	106	0.2636816	purine ribonucleoside monophosphate metabolic process	4.499E-09
30	GO:0009126	3.41E-11	106	0.2630273	purine nucleoside monophosphate metabolic process	4.919E-09
31	GO:1901988	4.41E-11	67	0.335	negative regulation of cell cycle phase transition	6.155E-09
32	GO:0009169	7.16E-11	92	0.2771084	purine ribonucleoside monophosphate catabolic process	9.392E-09
33	GO:0009158	7.16E-11	92	0.2771084	ribonucleoside monophosphate catabolic process	9.392E-09
34	GO:0009128	8.19E-11	92	0.2762763	purine nucleoside monophosphate catabolic process	1.039E-08
35	GO:0046034	8.41E-11	100	0.265252	ATP metabolic process	1.039E-08
36	GO:0009125	1.07E-10	92	0.2746269	nucleoside monophosphate catabolic process	1.282E-08
37	GO:0007346	1.2E-10	102	0.2608696	regulation of mitotic cell cycle	1.4E-08
38	GO:0006200	1.55E-10	90	0.2752294	ATP catabolic process	1.769E-08
39	GO:0032200	2.64E-10	34	0.5396825	telomere organization	2.927E-08
40	GO:0045930	4.67E-10	68	0.3105023	negative regulation of mitotic cell cycle	5.053E-08
41	GO:0090305	5.3E-10	70	0.3043478	nucleic acid phosphodiester bond hydrolysis	5.594E-08
42	GO:0008033	5.97E-10	42	0.4285714	tRNA processing	6.152E-08
43	GO:0000723	6.32E-10	33	0.5322581	telomere maintenance	6.354E-08
44	GO:0010948	8.62E-10	74	0.2913386	negative regulation of cell cycle process	8.471E-08
45	GO:0006284	3.98E-09	26	0.6190476	base-excision repair	3.825E-07
46	GO:0031145	6.04E-09	36	0.4390244	anaphase-promoting complex-dependent proteasomal ubiquitin-dependent protein catabolic process	5.679E-07
47	GO:0006312	1.04E-08	22	0.7096774	mitotic recombination	9.584E-07

48	GO:0008380	1.36E-08	81	0.2587859	RNA splicing	1.223E-06
49	GO:0000725	2.02E-08	30	0.483871	recombinational repair	1.781E-06
50	GO:0006270	3.08E-08	19	0.7916667	DNA replication initiation	2.663E-06
51	GO:0071103	4.13E-08	38	0.3838384	DNA conformation change	3.502E-06
52	GO:0000377	4.63E-08	54	0.3050847	RNA splicing, via transesterification reactions with bulged adenosine as nucleophile	3.734E-06
53	GO:0000398	4.63E-08	54	0.3050847	mRNA splicing, via spliceosome	3.734E-06
54	GO:0000724	4.66E-08	29	0.4754098	double-strand break repair via homologous recombination	3.734E-06
55	GO:0032201	5.02E-08	18	0.8181818	telomere maintenance via semi-conservative replication	3.948E-06
56	GO:0010467	6E-08	138	0.2053571	gene expression	4.597E-06
57	GO:0000375	6.16E-08	55	0.298913	RNA splicing, via transesterification reactions	4.597E-06
58	GO:0006333	6.16E-08	28	0.4827586	chromatin assembly or disassembly	4.597E-06
59	GO:0000722	7.56E-08	19	0.7307692	telomere maintenance via recombination	5.54E-06
60	GO:0007093	9.49E-08	38	0.368932	mitotic cell cycle checkpoint	6.84E-06
61	GO:0006397	1.23E-07	92	0.231738	mRNA processing	8.758E-06
62	GO:0051052	2.56E-07	68	0.2566038	regulation of DNA metabolic process	1.788E-05
63	GO:0000819	3.27E-07	17	0.7391304	sister chromatid segregation	2.247E-05
64	GO:0000070	3.45E-07	16	0.8	mitotic sister chromatid segregation	2.332E-05
65	GO:0007077	3.65E-07	21	0.5675676	mitotic nuclear envelope disassembly	2.428E-05
66	GO:0006839	4.44E-07	38	0.3423423	mitochondrial transport	2.908E-05
67	GO:0006308	4.89E-07	36	0.3529412	DNA catabolic process	3.158E-05
68	GO:0045786	4.97E-07	95	0.2209302	negative regulation of cell cycle	3.159E-05
69	GO:0051186	5.71E-07	65	0.254902	cofactor metabolic process	3.582E-05
70	GO:0000226	6.6E-07	63	0.2571429	microtubule cytoskeleton organization	4.08E-05
71	GO:0030397	7.02E-07	21	0.5384615	membrane disassembly	4.217E-05
72	GO:0051081	7.02E-07	21	0.5384615	nuclear envelope disassembly	4.217E-05
73	GO:0070507	8.22E-07	35	0.35	regulation of microtubule cytoskeleton organization	4.81E-05
74	GO:0031570	8.23E-07	32	0.372093	DNA integrity checkpoint	4.81E-05
75	GO:0010833	8.89E-07	20	0.5555556	telomere maintenance via telomere lengthening	5.129E-05
76	GO:0006998	9.99E-07	24	0.4615385	nuclear envelope organization	5.686E-05
77	GO:0006336	1.11E-06	19	0.5757576	DNA replication-independent nucleosome assembly	6.167E-05
78	GO:0034724	1.11E-06	19	0.5757576	DNA replication-independent nucleosome organization	6.167E-05
79	GO:0006732	1.43E-06	53	0.2704082	coenzyme metabolic process	7.85E-05
80	GO:0090068	1.82E-06	57	0.2590909	positive regulation of cell cycle process	9.847E-05
81	GO:0051983	1.9E-06	26	0.4126984	regulation of chromosome segregation	0.0001014
82	GO:0032508	2.17E-06	24	0.4363636	DNA duplex unwinding	0.0001147
83	GO:0051439	2.69E-06	29	0.3717949	regulation of ubiquitin-protein ligase activity involved in mitotic cell cycle	0.0001402
84	GO:0007051	3.03E-06	28	0.3783784	spindle organization	0.000156
85	GO:0032392	3.55E-06	24	0.4210526	DNA geometric change	0.0001808
86	GO:0000086	3.75E-06	41	0.294964	G2/M transition of mitotic cell cycle	0.0001867
87	GO:0044839	3.75E-06	41	0.294964	cell cycle G2/M phase transition	0.0001867
88	GO:0032886	4.02E-06	38	0.3064516	regulation of microtubule-based process	0.0001976
89	GO:0071897	4.85E-06	23	0.4259259	DNA biosynthetic process	0.0002356
90	GO:0051236	6.14E-06	43	0.2810458	establishment of RNA localization	0.0002887
91	GO:0050658	6.14E-06	43	0.2810458	RNA transport	0.0002887
92	GO:0050657	6.14E-06	43	0.2810458	nucleic acid transport	0.0002887
93	GO:0051188	6.38E-06	38	0.2992126	cofactor biosynthetic process	0.0002935
94	GO:0010212	6.38E-06	38	0.2992126	response to ionizing radiation	0.0002935
95	GO:2000045	8.6E-06	38	0.2945736	regulation of G1/S transition of mitotic cell cycle	0.0003915
96	GO:0009451	8.99E-06	30	0.3370787	RNA modification	0.0004053
97	GO:0031055	1.04E-05	15	0.6	chromatin remodeling at centromere	0.0004617
98	GO:1902806	1.15E-05	38	0.2900763	regulation of cell cycle G1/S phase transition	0.0005081
99	GO:0015931	1.2E-05	47	0.2611111	nucleobase-containing compound transport	0.0005246

100	GO:0006297	1.25E-05	14	0.6363636	nucleotide-excision repair, DNA gap filling	0.0005408
101	GO:0051437	1.32E-05	26	0.3611111	positive regulation of ubiquitin-protein ligase activity involved in mitotic cell cycle	0.0005653
102	GO:0000083	1.47E-05	15	0.5769231	regulation of transcription involved in G1/S transition of mitotic cell cycle	0.0006227
103	GO:0051351	1.52E-05	30	0.326087	positive regulation of ligase activity	0.0006402
104	GO:0051297	1.7E-05	24	0.375	centrosome organization	0.0007069
105	GO:0051340	1.78E-05	33	0.3055556	regulation of ligase activity	0.0007324
106	GO:0034080	1.81E-05	14	0.6086957	CENP-A containing nucleosome assembly	0.0007325
107	GO:0009108	1.81E-05	31	0.3163265	coenzyme biosynthetic process	0.0007325
108	GO:0007017	1.84E-05	89	0.2036613	microtubule-based process	0.0007325
109	GO:0010498	1.85E-05	52	0.245283	proteasomal protein catabolic process	0.0007325
110	GO:0006283	2.01E-05	20	0.4255319	transcription-coupled nucleotide-excision repair	0.0007886
111	GO:1902807	2.13E-05	31	0.3131313	negative regulation of cell cycle G1/S phase transition	0.000824
112	GO:2000134	2.13E-05	31	0.3131313	negative regulation of G1/S transition of mitotic cell cycle	0.000824
113	GO:0043486	2.19E-05	16	0.516129	histone exchange	0.0008396
114	GO:0031023	2.24E-05	25	0.3571429	microtubule organizing center organization	0.0008485
115	GO:0006289	2.35E-05	26	0.3466667	nucleotide-excision repair	0.0008846
116	GO:1903322	2.41E-05	42	0.2658228	positive regulation of protein modification by small protein conjugation or removal	0.0008969
117	GO:0051301	2.43E-05	37	0.2824427	cell division	0.0008983
118	GO:0006521	2.9E-05	23	0.3709677	regulation of cellular amino acid metabolic process	0.0010642
119	GO:0006353	2.96E-05	28	0.3255814	DNA-templated transcription, termination	0.0010767
120	GO:0051436	3.11E-05	24	0.358209	negative regulation of ubiquitin-protein ligase activity involved in mitotic cell cycle	0.0011228
121	GO:0000077	3.4E-05	26	0.3376623	DNA damage checkpoint	0.0012149
122	GO:0051443	3.51E-05	28	0.3218391	positive regulation of ubiquitin-protein transferase activity	0.0012453
123	GO:0051028	3.65E-05	37	0.2761194	mRNA transport	0.0012848
124	GO:0006520	3.69E-05	85	0.2019002	cellular amino acid metabolic process	0.0012861
125	GO:0031577	3.73E-05	18	0.4390244	spindle checkpoint	0.0012894
126	GO:0051438	4.01E-05	31	0.3009709	regulation of ubiquitin-protein transferase activity	0.0013753
127	GO:0031398	4.23E-05	39	0.2671233	positive regulation of protein ubiquitination	0.0014397
128	GO:0071459	4.53E-05	10	0.8333333	protein localization to chromosome, centromeric region	0.0015313
129	GO:0090501	4.9E-05	28	0.3146067	RNA phosphodiester bond hydrolysis	0.0016421
130	GO:0034502	5.01E-05	14	0.5384615	protein localization to chromosome	0.0016664
131	GO:0045787	6.35E-05	61	0.2194245	positive regulation of cell cycle	0.0020977
132	GO:0072401	6.62E-05	24	0.3380282	signal transduction involved in DNA integrity checkpoint	0.0021538
133	GO:0072422	6.62E-05	24	0.3380282	signal transduction involved in DNA damage checkpoint	0.0021538
134	GO:0007005	7.54E-05	44	0.2458101	mitochondrion organization	0.0024352
135	GO:0072395	7.92E-05	24	0.3333333	signal transduction involved in cell cycle checkpoint	0.0025317
136	GO:0000959	7.96E-05	12	0.6	mitochondrial RNA metabolic process	0.0025317
137	GO:0030163	8.4E-05	59	0.2193309	protein catabolic process	0.0026525
138	GO:0051303	8.86E-05	13	0.5416667	establishment of chromosome localization	0.0027759
139	GO:0072431	9.25E-05	23	0.3382353	signal transduction involved in mitotic G1 DNA damage checkpoint	0.0028584
140	GO:1902400	9.25E-05	23	0.3382353	intracellular signal transduction involved in G1 DNA damage checkpoint	0.0028584
141	GO:1903320	9.39E-05	52	0.2280702	regulation of protein modification by small protein conjugation or removal	0.0028817
142	GO:0006458	9.61E-05	20	0.3703704	'de novo' protein folding	0.0029271
143	GO:0043161	0.000101	48	0.2341463	proteasome-mediated ubiquitin-dependent protein catabolic process	0.003044
144	GO:0072413	0.000111	23	0.3333333	signal transduction involved in mitotic cell cycle checkpoint	0.0032623

145	GO:1902402	0.000111	23	0.3333333	signal transduction involved in mitotic DNA damage checkpoint	0.0032623
146	GO:1902403	0.000111	23	0.3333333	signal transduction involved in mitotic DNA integrity checkpoint	0.0032623
147	GO:0031396	0.000112	49	0.2311321	regulation of protein ubiquitination	0.0032623
148	GO:0051444	0.000112	24	0.3243243	negative regulation of ubiquitin-protein transferase activity	0.0032623
149	GO:0051352	0.000112	24	0.3243243	negative regulation of ligase activity	0.0032623
150	GO:0000737	0.000129	22	0.3384615	DNA catabolic process, endonucleolytic	0.0037292
151	GO:0002562	0.000156	15	0.4411765	somatic diversification of immune receptors via germline recombination within a single locus	0.0044479
152	GO:0016444	0.000156	15	0.4411765	somatic cell DNA recombination	0.0044479
153	GO:0065004	0.000167	31	0.2743363	protein-DNA complex assembly	0.0047128
154	GO:0033238	0.000183	25	0.304878	regulation of cellular amine metabolic process	0.0051291
155	GO:0006977	0.000185	22	0.3283582	DNA damage response, signal transduction by p53 class mediator resulting in cell cycle arrest	0.0051548
156	GO:0071824	0.000188	35	0.2573529	protein-DNA complex subunit organization	0.0052221
157	GO:0034501	0.0002	8	0.8888889	protein localization to kinetochore	0.0055007
158	GO:0010565	0.000231	42	0.2359551	regulation of cellular ketone metabolic process	0.0063234
159	GO:0042559	0.000247	10	0.625	pteridine-containing compound biosynthetic process	0.0067084
160	GO:0071479	0.00026	17	0.3777778	cellular response to ionizing radiation	0.0070371
161	GO:0042770	0.000277	29	0.2735849	signal transduction in response to DNA damage	0.0074429
162	GO:0046605	0.000291	12	0.5	regulation of centrosome cycle	0.0077657
163	GO:0006275	0.000316	31	0.2627119	regulation of DNA replication	0.008397
164	GO:0044774	0.000324	15	0.4054054	mitotic DNA integrity checkpoint	0.0084914
165	GO:0002200	0.000324	15	0.4054054	somatic diversification of immune receptors	0.0084914
166	GO:0051310	0.000353	10	0.5882353	metaphase plate congression	0.0091877
167	GO:0090502	0.000355	19	0.3392857	RNA phosphodiester bond hydrolysis, endonucleolytic	0.0091877
168	GO:0090407	0.000375	82	0.1880734	organophosphate biosynthetic process	0.0096446
169	GO:0031114	0.000388	12	0.48	regulation of microtubule depolymerization	0.0099291
170	GO:0043044	0.000413	18	0.3461538	ATP-dependent chromatin remodeling	0.0105061
171	GO:0060249	0.000415	46	0.2211538	anatomical structure homeostasis	0.0105061
172	GO:0072331	0.00044	33	0.25	signal transduction by p53 class mediator	0.0110563
173	GO:0090329	0.000449	14	0.4117647	regulation of DNA-dependent DNA replication	0.0112156
174	GO:0071156	0.000494	28	0.2666667	regulation of cell cycle arrest	0.0122839
175	GO:0000209	0.000505	42	0.2258065	protein polyubiquitination	0.0124049
176	GO:0000076	0.000508	8	0.7272727	DNA replication checkpoint	0.0124049
177	GO:0051988	0.000508	8	0.7272727	regulation of attachment of spindle microtubules to kinetochore	0.0124049
178	GO:0044085	0.000512	25	0.2808989	cellular component biogenesis	0.0124529
179	GO:0030071	0.00055	16	0.3636364	regulation of mitotic metaphase/anaphase transition	0.0132487
180	GO:0009411	0.000551	30	0.2564103	response to UV	0.0132487
181	GO:0051084	0.000579	17	0.3469388	'de novo' posttranslational protein folding	0.0138438
182	GO:0007088	0.000592	29	0.2589286	regulation of mitosis	0.0140766
183	GO:0071158	0.000618	24	0.2823529	positive regulation of cell cycle arrest	0.0146017
184	GO:0031397	0.000668	29	0.2566372	negative regulation of protein ubiquitination	0.0156492
185	GO:1902099	0.000671	16	0.3555556	regulation of metaphase/anaphase transition of cell cycle	0.0156492
186	GO:0030330	0.000673	25	0.2747253	DNA damage response, signal transduction by p53 class mediator	0.0156492
187	GO:0007026	0.000691	11	0.4782609	negative regulation of microtubule depolymerization	0.0159786
188	GO:0071174	0.000707	14	0.3888889	mitotic spindle checkpoint	0.0162432
189	GO:0009165	0.00071	45	0.2163462	nucleotide biosynthetic process	0.0162432
190	GO:0006334	0.000723	26	0.2680412	nucleosome assembly	0.0164687
191	GO:1901293	0.000773	45	0.215311	nucleoside phosphate biosynthetic process	0.0175086
192	GO:0034728	0.000782	30	0.25	nucleosome organization	0.0175292

193	GO:0042558	0.000786	13	0.40625	pteridine-containing compound metabolic process	0.0175292
194	GO:0031111	0.000786	13	0.40625	negative regulation of microtubule polymerization or depolymerization	0.0175292
195	GO:0051782	0.000827	20	0.3030303	negative regulation of cell division	0.0183452
196	GO:0022613	0.000841	19	0.3114754	ribonucleoprotein complex biogenesis	0.0185704
197	GO:0051783	0.000847	33	0.2391304	regulation of nuclear division	0.0186016
198	GO:0008334	0.000857	12	0.4285714	histone mRNA metabolic process	0.018721
199	GO:0033047	0.000942	15	0.3571429	regulation of mitotic sister chromatid segregation	0.0202715
200	GO:0010965	0.000942	15	0.3571429	regulation of mitotic sister chromatid separation	0.0202715
201	GO:0033045	0.000942	15	0.3571429	regulation of sister chromatid segregation	0.0202715
202	GO:0006220	0.000981	16	0.3404255	pyrimidine nucleotide metabolic process	0.0209918
203	GO:0071173	0.000987	13	0.3939394	spindle assembly checkpoint	0.0209918
204	GO:0031110	0.001	17	0.3269231	regulation of microtubule polymerization or depolymerization	0.0209918
205	GO:0043038	0.001	17	0.3269231	amino acid activation	0.0209918
206	GO:0043039	0.001	17	0.3269231	tRNA aminoacylation	0.0209918
207	GO:0032259	0.001024	54	0.2007435	methylation	0.0213924
208	GO:0006298	0.001092	12	0.4137931	mismatch repair	0.0226073
209	GO:0043928	0.001092	12	0.4137931	exonucleolytic nuclear-transcribed mRNA catabolic process involved in deadenylation-dependent decay	0.0226073
210	GO:0007080	0.001118	8	0.6153846	mitotic metaphase plate congression	0.0230343
211	GO:0043632	0.001128	69	0.1875	modification-dependent macromolecule catabolic process	0.0231329
212	GO:0002478	0.00117	38	0.2222222	antigen processing and presentation of exogenous peptide antigen	0.0238775
213	GO:0042590	0.001187	22	0.278481	antigen processing and presentation of exogenous peptide antigen via MHC class I	0.0240112
214	GO:0000288	0.001188	17	0.3207547	nuclear-transcribed mRNA catabolic process, deadenylation-dependent decay	0.0240112
215	GO:0042254	0.00123	13	0.3823529	ribosome biogenesis	0.0242963
216	GO:0045841	0.00123	13	0.3823529	negative regulation of mitotic metaphase/anaphase transition	0.0242963
217	GO:0033046	0.00123	13	0.3823529	negative regulation of sister chromatid segregation	0.0242963
218	GO:0033048	0.00123	13	0.3823529	negative regulation of mitotic sister chromatid segregation	0.0242963
219	GO:2000816	0.00123	13	0.3823529	negative regulation of mitotic sister chromatid separation	0.0242963
220	GO:0051302	0.001287	48	0.2051282	regulation of cell division	0.0253115
221	GO:0006400	0.001324	14	0.3589744	tRNA modification	0.0258222
222	GO:1903321	0.001325	29	0.2436975	negative regulation of protein modification by small protein conjugation or removal	0.0258222
223	GO:0006268	0.001347	7	0.7	DNA unwinding involved in DNA replication	0.0261223
224	GO:0051603	0.001356	72	0.1841432	proteolysis involved in cellular protein catabolic process	0.026196
225	GO:0019884	0.001401	38	0.2196532	antigen processing and presentation of exogenous antigen	0.0269091
226	GO:0006418	0.001406	16	0.3265306	tRNA aminoacylation for protein translation	0.0269091
227	GO:0002479	0.001435	21	0.28	antigen processing and presentation of exogenous peptide antigen via MHC class I, TAP-dependent	0.0273443
228	GO:0006261	0.001516	11	0.4230769	DNA-dependent DNA replication	0.0287185
229	GO:1902100	0.00152	13	0.3714286	negative regulation of metaphase/anaphase transition of cell cycle	0.0287185
230	GO:0022618	0.001544	32	0.2318841	ribonucleoprotein complex assembly	0.029048
231	GO:0031109	0.00159	8	0.5714286	microtubule polymerization or depolymerization	0.0297742
232	GO:0043623	0.0016	51	0.1992188	cellular protein complex assembly	0.0298313
233	GO:0000726	0.001661	9	0.5	non-recombinational repair	0.0305779
234	GO:0045005	0.001661	9	0.5	maintenance of fidelity involved in DNA-dependent DNA replication	0.0305779
235	GO:0006303	0.001661	9	0.5	double-strand break repair via nonhomologous end joining	0.0305779
236	GO:0042398	0.00167	16	0.32	cellular modified amino acid biosynthetic process	0.0306174

237	GO:0000291	0.001725	12	0.3870968	nuclear-transcribed mRNA catabolic process, exonucleolytic	0.031481
238	GO:0051985	0.001865	13	0.3611111	negative regulation of chromosome segregation	0.0338997
239	GO:0031100	0.001975	16	0.3137255	organ regeneration	0.0356875
240	GO:0006369	0.00198	15	0.326087	termination of RNA polymerase II transcription	0.0356875
241	GO:0051085	0.001997	7	0.6363636	chaperone mediated protein folding requiring cofactor	0.0358415
242	GO:0016447	0.0021	10	0.4347826	somatic recombination of immunoglobulin gene segments	0.0373876
243	GO:0000738	0.0021	10	0.4347826	DNA catabolic process, exonucleolytic	0.0373876
244	GO:0007094	0.002139	12	0.375	mitotic spindle assembly checkpoint	0.0377648
245	GO:0044773	0.002139	12	0.375	mitotic DNA damage checkpoint	0.0377648
246	GO:0006338	0.002214	28	0.2372881	chromatin remodeling	0.0389414
247	GO:0000387	0.002272	13	0.3513514	spliceosomal snRNP assembly	0.0397895
248	GO:0048002	0.002311	39	0.2108108	antigen processing and presentation of peptide antigen	0.0402387
249	GO:0000018	0.002325	16	0.3076923	regulation of DNA recombination	0.0402387
250	GO:0032435	0.002325	16	0.3076923	negative regulation of proteasomal ubiquitin-dependent protein catabolic process	0.0402387
251	GO:0051292	0.002362	6	0.75	nuclear pore complex assembly	0.0407126
252	GO:0009163	0.002456	28	0.2352941	nucleoside biosynthetic process	0.0421641
253	GO:0006221	0.002631	12	0.3636364	pyrimidine nucleotide biosynthetic process	0.0449624
254	GO:0070585	0.00264	17	0.2931034	protein localization to mitochondrion	0.0449624
255	GO:1901659	0.00272	28	0.2333333	glycosyl compound biosynthetic process	0.0461451
256	GO:0019941	0.002899	66	0.1813187	modification-dependent protein catabolic process	0.0488322
257	GO:0010824	0.002901	9	0.45	regulation of centrosome duplication	0.0488322

d. List of gene ontology biological process subcategories in genes down-regulated in U87-MG cultuered in hypoxia versus normoxia after processing with REViGO.

Term_ID	Description	Uniqueness	Dispensability
GO:0006260	DNA replication	0.659	0
GO:0006281	DNA repair	0.581	0.748
GO:0006839	mitochondrial transport	0.942	0
GO:0010212	response to ionizing radiation	0.968	0
GO:0009411	response to UV	0.968	0.798
GO:0071479	cellular response to ionizing radiation	0.894	0.745
GO:0051276	chromosome organization	0.734	0
GO:0051297	centrosome organization	0.715	0.762
GO:0032886	regulation of microtubule-based process	0.799	0.755
GO:0044085	cellular component biogenesis	0.818	0.534
GO:0070507	regulation of microtubule cytoskeleton organization	0.659	0.745
GO:0046605	regulation of centrosome cycle	0.535	0.895
GO:0051988	regulation of attachment of spindle microtubules to kinetochore	0.626	0.844
GO:0007051	spindle organization	0.545	0.759
GO:0031023	microtubule organizing center organization	0.716	0.766
GO:0007026	negative regulation of microtubule depolymerization	0.624	0.875
GO:0007005	mitochondrion organization	0.761	0.561
GO:0010824	regulation of centrosome duplication	0.544	0.875
GO:0031111	negative regulation of microtubule polymerization or depolymerization	0.637	0.912
GO:0031110	regulation of microtubule polymerization or depolymerization	0.671	0.926
GO:0031109	microtubule polymerization or depolymerization	0.722	0.747
GO:0031114	regulation of microtubule depolymerization	0.665	0.977
GO:0000226	microtubule cytoskeleton organization	0.693	0.59
GO:0051340	regulation of ligase activity	0.884	0
GO:0002200	somatic diversification of immune receptors	0.959	0.016
GO:0000082	G1/S transition of mitotic cell cycle	0.604	0.045
GO:0090068	positive regulation of cell cycle process	0.585	0.848
GO:0045841	negative regulation of mitotic metaphase/anaphase transition	0.464	0.998
GO:0072401	signal transduction involved in DNA integrity checkpoint	0.557	0.997
GO:0071158	positive regulation of cell cycle arrest	0.607	0.828
GO:0045930	negative regulation of mitotic cell cycle	0.633	0.62
GO:0044839	cell cycle G2/M phase transition	0.63	0.824
GO:0007346	regulation of mitotic cell cycle	0.597	0.824
GO:0044843	cell cycle G1/S phase transition	0.613	0.872
GO:0072431	signal transduction involved in mitotic G1 DNA damage checkpoint	0.521	0.998
GO:0072422	signal transduction involved in DNA damage checkpoint	0.529	0.997
GO:0072413	signal transduction involved in mitotic cell cycle checkpoint	0.553	0.993
GO:0000819	sister chromatid segregation	0.526	0.89
GO:0051783	regulation of nuclear division	0.65	0.765
GO:2000134	negative regulation of G1/S transition of mitotic cell cycle	0.564	0.923
GO:1902807	negative regulation of cell cycle G1/S phase transition	0.571	0.973
GO:1902806	regulation of cell cycle G1/S phase transition	0.581	0.947
GO:1902099	regulation of metaphase/anaphase transition of cell cycle	0.466	0.976
GO:1902100	negative regulation of metaphase/anaphase transition of cell cycle	0.467	0.979
GO:0010564	regulation of cell cycle process	0.589	0.717
GO:2000816	negative regulation of mitotic sister chromatid separation	0.477	0.956
GO:0030071	regulation of mitotic metaphase/anaphase transition	0.466	0.918
GO:1901987	regulation of cell cycle phase transition	0.574	0.897
GO:1901988	negative regulation of cell cycle phase transition	0.558	0.965

GO:0010965	regulation of mitotic sister chromatid separation	0.474	0.988
GO:0033047	regulation of mitotic sister chromatid segregation	0.474	0.999
GO:0033048	negative regulation of mitotic sister chromatid segregation	0.484	0.935
GO:0033045	regulation of sister chromatid segregation	0.496	0.935
GO:0033046	negative regulation of sister chromatid segregation	0.493	0.947
GO:1902400	intracellular signal transduction involved in G1 DNA damage checkpoint	0.527	0.992
GO:1902403	signal transduction involved in mitotic DNA integrity checkpoint	0.551	0.999
GO:1902402	signal transduction involved in mitotic DNA damage checkpoint	0.524	0.997
GO:1901990	regulation of mitotic cell cycle phase transition	0.556	0.887
GO:1901991	negative regulation of mitotic cell cycle phase transition	0.548	0.977
GO:2000045	regulation of G1/S transition of mitotic cell cycle	0.572	0.973
GO:0010948	negative regulation of cell cycle process	0.576	0.793
GO:0007093	mitotic cell cycle checkpoint	0.545	0.948
GO:0007094	mitotic spindle assembly checkpoint	0.455	0.976
GO:0000075	cell cycle checkpoint	0.654	0.723
GO:0000070	mitotic sister chromatid segregation	0.501	0.718
GO:0007088	regulation of mitosis	0.491	0.88
GO:0051985	negative regulation of chromosome segregation	0.709	0.998
GO:0000083	regulation of transcription involved in G1/S transition of mitotic cell cycle	0.522	0.825
GO:0051983	regulation of chromosome segregation	0.774	0.857
GO:0000086	G2/M transition of mitotic cell cycle	0.627	0.827
GO:0071156	regulation of cell cycle arrest	0.601	0.722
GO:0006977	DNA damage response, signal transduction by p53 class mediator resulting in cell cycle arrest	0.516	0.955
GO:0045787	positive regulation of cell cycle	0.636	0.736
GO:0045786	negative regulation of cell cycle	0.601	0.815
GO:0000280	nuclear division	0.683	0.046
GO:0048285	organelle fission	0.749	0.552
GO:0007067	mitotic nuclear division	0.508	0.928
GO:0006310	DNA recombination	0.68	0.046
GO:0006302	double-strand break repair	0.624	0.608
GO:0006303	double-strand break repair via nonhomologous end joining	0.665	0.813
GO:0006298	mismatch repair	0.64	0.69
GO:0030330	DNA damage response, signal transduction by p53 class mediator	0.762	0.64
GO:0006312	mitotic recombination	0.718	0.52
GO:0006271	DNA strand elongation involved in DNA replication	0.715	0.513
GO:0006270	DNA replication initiation	0.7	0.794
GO:0006275	regulation of DNA replication	0.624	0.674
GO:0006261	DNA-dependent DNA replication	0.68	0.782
GO:0006289	nucleotide-excision repair	0.634	0.704
GO:0006284	base-excision repair	0.647	0.675
GO:0022616	DNA strand elongation	0.732	0.517
GO:0072395	signal transduction involved in cell cycle checkpoint	0.566	0.847
GO:0071897	DNA biosynthetic process	0.685	0.604
GO:0044773	mitotic DNA damage checkpoint	0.516	0.949
GO:0044774	mitotic DNA integrity checkpoint	0.558	0.952
GO:0071174	mitotic spindle checkpoint	0.583	0.819
GO:0071173	spindle assembly checkpoint	0.662	0.946
GO:0045005	maintenance of fidelity involved in DNA-dependent DNA replication	0.716	0.751
GO:0000018	regulation of DNA recombination	0.648	0.809
GO:0090329	regulation of DNA-dependent DNA replication	0.651	0.824
GO:0000076	DNA replication checkpoint	0.698	0.781
GO:0000077	DNA damage checkpoint	0.583	0.666
GO:0031577	spindle checkpoint	0.655	0.841

GO:0031570	DNA integrity checkpoint	0.628	0.922
GO:0051052	regulation of DNA metabolic process	0.619	0.654
GO:0071103	DNA conformation change	0.677	0.648
GO:0002562	somatic diversification of immune receptors via germline recombination within a single locus	0.682	0.982
GO:0042770	signal transduction in response to DNA damage	0.772	0.646
GO:0016444	somatic cell DNA recombination	0.694	0.8
GO:0000726	non-recombinational repair	0.665	0.635
GO:0016447	somatic recombination of immunoglobulin gene segments	0.687	0.787
GO:0000725	recombinational repair	0.634	0.789
GO:0000724	double-strand break repair via homologous recombination	0.632	0.732
GO:0032259	methylation	0.942	0.056
GO:0060249	anatomical structure homeostasis	0.937	0.056
GO:0010833	telomere maintenance via telomere lengthening	0.568	0.922
GO:0032201	telomere maintenance via semi-conservative replication	0.452	0.877
GO:0000723	telomere maintenance	0.54	0.577
GO:0000722	telomere maintenance via recombination	0.574	0.882
GO:0072331	signal transduction by p53 class mediator	0.814	0.076
GO:0007059	chromosome segregation	0.893	0.08
GO:0007017	microtubule-based process	0.879	0.092
GO:0006732	coenzyme metabolic process	0.868	0.097
GO:0009108	coenzyme biosynthetic process	0.849	0.893
GO:0051188	cofactor biosynthetic process	0.847	0.914
GO:0051186	cofactor metabolic process	0.877	0.101
GO:0000278	mitotic cell cycle	0.641	0.101
GO:0044772	mitotic cell cycle phase transition	0.586	0.717
GO:0044770	cell cycle phase transition	0.634	0.745
GO:0051301	cell division	0.879	0.107
GO:0002478	antigen processing and presentation of exogenous peptide antigen	0.971	0.142
GO:0002479	antigen processing and presentation of exogenous peptide antigen via MHC class I, TAP-dependent	0.971	0.943
GO:0042590	antigen processing and presentation of exogenous peptide antigen via MHC class I	0.971	0.946
GO:0051302	regulation of cell division	0.778	0.159
GO:0051782	negative regulation of cell division	0.769	0.592
GO:0051084	'de novo' posttranslational protein folding	0.816	0.166
GO:0051085	chaperone mediated protein folding requiring cofactor	0.823	0.949
GO:0006458	'de novo' protein folding	0.817	0.708
GO:0019884	antigen processing and presentation of exogenous antigen	0.983	0.181
GO:0006200	ATP catabolic process	0.686	0.22
GO:1901293	nucleoside phosphate biosynthetic process	0.705	0.915
GO:0009123	nucleoside monophosphate metabolic process	0.699	0.746
GO:0009125	nucleoside monophosphate catabolic process	0.698	0.694
GO:0009126	purine nucleoside monophosphate metabolic process	0.69	0.94
GO:0009128	purine nucleoside monophosphate catabolic process	0.692	0.988
GO:0090407	organophosphate biosynthetic process	0.797	0.542
GO:0009163	nucleoside biosynthetic process	0.709	0.718
GO:0009161	ribonucleoside monophosphate metabolic process	0.69	0.937
GO:0009158	ribonucleoside monophosphate catabolic process	0.692	0.987
GO:0009169	purine ribonucleoside monophosphate catabolic process	0.692	0.988
GO:0009167	purine ribonucleoside monophosphate metabolic process	0.69	0.941
GO:0009165	nucleotide biosynthetic process	0.705	0.514
GO:0006220	pyrimidine nucleotide metabolic process	0.773	0.907
GO:0046034	ATP metabolic process	0.683	0.734

GO:1901659	glycosyl compound biosynthetic process	0.82	0.814
GO:0006221	pyrimidine nucleotide biosynthetic process	0.758	0.709
GO:0000959	mitochondrial RNA metabolic process	0.8	0.237
GO:0010467	gene expression	0.819	0.238
GO:0008334	histone mRNA metabolic process	0.782	0.241
GO:0000291	nuclear-transcribed mRNA catabolic process, exonucleolytic	0.737	0.802
GO:0000288	nuclear-transcribed mRNA catabolic process, deadenylation-dependent decay	0.721	0.595
GO:0043928	exonucleolytic nuclear-transcribed mRNA catabolic process involved in deadenylation-dependent decay	0.739	0.798
GO:0006353	DNA-templated transcription, termination	0.771	0.263
GO:0015931	nucleobase-containing compound transport	0.96	0.264
GO:0051236	establishment of RNA localization	0.952	0.987
GO:0050658	RNA transport	0.943	0.982
GO:0050657	nucleic acid transport	0.956	0.939
GO:0051028	mRNA transport	0.944	0.652
GO:0006369	termination of RNA polymerase II transcription	0.78	0.278
GO:0016072	rRNA metabolic process	0.746	0.28
GO:0006400	tRNA modification	0.723	0.732
GO:0006399	tRNA metabolic process	0.727	0.824
GO:0048002	antigen processing and presentation of peptide antigen	0.98	0.282
GO:0090501	RNA phosphodiester bond hydrolysis	0.761	0.289
GO:0090502	RNA phosphodiester bond hydrolysis, endonucleolytic	0.767	0.777
GO:0000738	DNA catabolic process, exonucleolytic	0.68	0.855
GO:0000737	DNA catabolic process, endonucleolytic	0.651	0.796
GO:0090305	nucleic acid phosphodiester bond hydrolysis	0.759	0.31
GO:0008380	RNA splicing	0.723	0.321
GO:0000377	RNA splicing, via transesterification reactions with bulged adenosine as nucleophile	0.728	0.993
GO:0000375	RNA splicing, via transesterification reactions	0.728	0.916
GO:0000398	mRNA splicing, via spliceosome	0.716	0.869
GO:0006397	mRNA processing	0.705	0.823
GO:0042559	pteridine-containing compound biosynthetic process	0.812	0.323
GO:0031100	organ regeneration	0.969	0.324
GO:0030397	membrane disassembly	0.797	0.324
GO:0051292	nuclear pore complex assembly	0.79	0.774
GO:0007077	mitotic nuclear envelope disassembly	0.591	0.854
GO:0051081	nuclear envelope disassembly	0.759	0.984
GO:0006998	nuclear envelope organization	0.781	0.577
GO:0009451	RNA modification	0.749	0.329
GO:0010565	regulation of cellular ketone metabolic process	0.754	0.337
GO:0006521	regulation of cellular amino acid metabolic process	0.732	0.84
GO:0033238	regulation of cellular amine metabolic process	0.755	0.338
GO:0034660	ncRNA metabolic process	0.741	0.34
GO:0042558	pteridine-containing compound metabolic process	0.821	0.342
GO:1903320	regulation of protein modification by small protein conjugation or removal	0.715	0.358
GO:1903321	negative regulation of protein modification by small protein conjugation or removal	0.669	0.891
GO:1903322	positive regulation of protein modification by small protein conjugation or removal	0.703	0.919
GO:0051352	negative regulation of ligase activity	0.859	0.939
GO:0051351	positive regulation of ligase activity	0.857	0.958
GO:0031396	regulation of protein ubiquitination	0.701	0.954

GO:0051436	negative regulation of ubiquitin-protein ligase activity involved in mitotic cell cycle	0.519	0.98
GO:0051437	positive regulation of ubiquitin-protein ligase activity involved in mitotic cell cycle	0.522	0.916
GO:0051438	regulation of ubiquitin-protein transferase activity	0.704	0.981
GO:0031398	positive regulation of protein ubiquitination	0.703	0.698
GO:0051439	regulation of ubiquitin-protein ligase activity involved in mitotic cell cycle	0.52	0.947
GO:0031397	negative regulation of protein ubiquitination	0.668	0.898
GO:0051443	positive regulation of ubiquitin-protein transferase activity	0.702	0.977
GO:0051444	negative regulation of ubiquitin-protein transferase activity	0.664	0.988
GO:0000209	protein polyubiquitination	0.777	0.707
GO:0034470	ncRNA processing	0.71	0.362
GO:0034502	protein localization to chromosome	0.954	0.388
GO:0070585	protein localization to mitochondrion	0.951	0.605
GO:0071824	protein-DNA complex subunit organization	0.832	0.39
GO:0022613	ribonucleoprotein complex biogenesis	0.834	0.408
GO:0008033	tRNA processing	0.722	0.859
GO:0022618	ribonucleoprotein complex assembly	0.813	0.886
GO:0006364	rRNA processing	0.623	0.854
GO:0000387	spliceosomal snRNP assembly	0.635	0.777
GO:0043039	tRNA aminoacylation	0.685	0.994
GO:0042254	ribosome biogenesis	0.834	0.53
GO:0006418	tRNA aminoacylation for protein translation	0.663	0.874
GO:0051303	establishment of chromosome localization	0.953	0.41
GO:0051310	metaphase plate congression	0.802	0.966
GO:0007080	mitotic metaphase plate congression	0.546	0.934
GO:0006520	cellular amino acid metabolic process	0.739	0.434
GO:0043038	amino acid activation	0.771	0.688
GO:0042398	cellular modified amino acid biosynthetic process	0.76	0.648
GO:0031145	anaphase-promoting complex-dependent proteasomal ubiquitin-dependent protein catabolic process	0.77	0.438
GO:0006308	DNA catabolic process	0.651	0.616
GO:0043161	proteasome-mediated ubiquitin-dependent protein catabolic process	0.744	0.903
GO:0019941	modification-dependent protein catabolic process	0.728	0.89
GO:0043632	modification-dependent macromolecule catabolic process	0.746	0.851
GO:0032435	negative regulation of proteasomal ubiquitin-dependent protein catabolic process	0.671	0.721
GO:0010498	proteasomal protein catabolic process	0.745	0.779
GO:0030163	protein catabolic process	0.775	0.729
GO:0051603	proteolysis involved in cellular protein catabolic process	0.726	0.972
GO:0032200	telomere organization	0.751	0.483
GO:0006268	DNA unwinding involved in DNA replication	0.713	0.863
GO:0006338	chromatin remodeling	0.734	0.665
GO:0006336	DNA replication-independent nucleosome assembly	0.571	0.515
GO:0006333	chromatin assembly or disassembly	0.731	0.595
GO:0006334	nucleosome assembly	0.524	0.946
GO:0032392	DNA geometric change	0.695	0.808
GO:0043623	cellular protein complex assembly	0.799	0.653
GO:0043044	ATP-dependent chromatin remodeling	0.748	0.796
GO:0065004	protein-DNA complex assembly	0.805	0.829
GO:0031055	chromatin remodeling at centromere	0.761	0.508
GO:0032508	DNA duplex unwinding	0.694	0.72
GO:0043486	histone exchange	0.745	0.808

GO:0034728	nucleosome organization	0.719	0.959
GO:0034724	DNA replication-independent nucleosome organization	0.75	0.762
GO:0034080	CENP-A containing nucleosome assembly	0.575	0.979
GO:0071459	protein localization to chromosome, centromeric region	0.956	0.486
GO:0034501	protein localization to kinetochore	0.954	0.809
GO:0006297	nucleotide-excision repair, DNA gap filling	0.68	0.495
GO:0006283	transcription-coupled nucleotide-excision repair	0.664	0.78
