# Supplementary material for: Integrated analysis identified core signal pathways and hypoxic characteristics of human glioblastoma
Source: J Cell Mol Med. 2019 Jul 7;23(9):6228–37. doi: 10.1111/jcmm.14507 (PMC6714287; doi:10.1111/jcmm.14507)
Supplement: Supplementary file 16 [file JCMM-23-6228-s017.pdf]

**Table S9** List of over-represented gene ontology biological process subcategories (GOBPID) in the genes differentially expressed (log2 fold change >1) with statistical significance (BH-corrected p-value <0.05) between HEB cultuered in hypoxia versus normoxia.

a. List of gene ontology biological process subcategories in genes up-regulated in HEB cultuered in hypoxia versus normoxia

| | GOBPID | Pvalue | Count | % | GO Term | Pvalue.BH corrected |
|---|---|---|---|---|---|---|
| 1 | GO:000166 | 0.00E+00 | 33 | 0.1369295 | response to hypoxia | 1.9E-13 |
| 2 | GO:007048 | 0.00E+00 | 34 | 0.1312741 | response to oxygen levels | 1.9E-13 |
| 3 | GO:003629 | 0.00E+00 | 33 | 0.1346939 | response to decreased oxygen levels | 1.9E-13 |
| 4 | GO:007145 | 4.603E-09 | 16 | 0.146789 | cellular response to oxygen levels | 3.279E-06 |
| 5 | GO:007145 | 9.609E-09 | 15 | 0.1515152 | cellular response to hypoxia | 5.475E-06 |
| 6 | GO:003629 | 1.224E-08 | 15 | 0.1485149 | cellular response to decreased oxygen levels | 5.811E-06 |
| 7 | GO:003019 | 2.174E-08 | 27 | 0.0752089 | extracellular matrix organization | 8.175E-06 |
| 8 | GO:004306 | 2.295E-08 | 27 | 0.075 | extracellular structure organization | 8.175E-06 |
| 9 | GO:000609 | 1.028E-07 | 12 | 0.1690141 | pyruvate metabolic process | 3.256E-05 |
| 10 | GO:003033 | 9.197E-07 | 16 | 0.0963855 | negative regulation of cell migration | 0.000262 |
| 11 | GO:000609 | 1.033E-06 | 9 | 0.2045455 | glycolytic process | 0.0002676 |
| 12 | GO:200014 | 1.229E-06 | 16 | 0.0941176 | negative regulation of cell motility | 0.0002918 |
| 13 | GO:004001 | 2.686E-06 | 17 | 0.0833333 | negative regulation of locomotion | 0.0005885 |
| 14 | GO:005127 | 2.991E-06 | 16 | 0.0874317 | negative regulation of cellular component movement | 0.0006086 |
| 15 | GO:000741 | 4.734E-06 | 23 | 0.0626703 | axon guidance | 0.000843 |
| 16 | GO:009748 | 4.734E-06 | 23 | 0.0626703 | neuron projection guidance | 0.000843 |
| 17 | GO:001003 | 5.373E-06 | 23 | 0.0621622 | response to inorganic substance | 0.0009005 |
| 18 | GO:001003 | 1.183E-05 | 18 | 0.0705882 | response to metal ion | 0.0018721 |
| 19 | GO:000152 | 1.622E-05 | 17 | 0.0720339 | angiogenesis | 0.0024322 |
| 20 | GO:001947 | 2.278E-05 | 5 | 0.3846154 | 4-hydroxyproline metabolic process | 0.0032449 |
| 21 | GO:000600 | 2.604E-05 | 13 | 0.0872483 | glucose metabolic process | 0.0035334 |
| 22 | GO:003278 | 2.92E-05 | 25 | 0.0528541 | monocarboxylic acid metabolic process | 0.0037813 |
| 23 | GO:000756 | 4.103E-05 | 15 | 0.0735294 | aging | 0.0050823 |
| 24 | GO:004361 | 4.718E-05 | 7 | 0.1707317 | regulation of transcription from RNA polymerase II promoter in response to stress | 0.0053234 |
| 25 | GO:003019 | 4.718E-05 | 7 | 0.1707317 | collagen fibril organization | 0.0053234 |
| 26 | GO:000999 | 4.858E-05 | 19 | 0.0607029 | response to extracellular stimulus | 0.0053234 |
| 27 | GO:000961 | 7.793E-05 | 13 | 0.0778443 | response to wounding | 0.0082229 |
| 28 | GO:004576 | 8.499E-05 | 10 | 0.0990099 | positive regulation of angiogenesis | 0.0086477 |
| 29 | GO:004362 | 9.059E-05 | 7 | 0.1521739 | regulation of DNA-templated transcription in response to stress | 0.0089 |
| 30 | GO:000599 | 9.596E-05 | 15 | 0.0678733 | monosaccharide metabolic process | 0.0091134 |
| 31 | GO:190134 | 0.0001039 | 14 | 0.071066 | regulation of vasculature development | 0.009547 |
| 32 | GO:006042 | 0.0001397 | 16 | 0.0625 | epithelium development | 0.0120727 |
| 33 | GO:005138 | 0.0001398 | 11 | 0.0846154 | response to glucocorticoid | 0.0120727 |
| 34 | GO:003009 | 0.0001451 | 12 | 0.0779221 | myeloid cell differentiation | 0.0121594 |
| 35 | GO:003166 | 0.000187 | 17 | 0.0584192 | response to nutrient levels | 0.0152212 |
| 36 | GO:001931 | 0.0002238 | 13 | 0.0695187 | hexose metabolic process | 0.0177151 |
| 37 | GO:006039 | 0.0002429 | 6 | 0.1578947 | regulation of pathway-restricted SMAD protein phosphorylation | 0.0187047 |
| 38 | GO:003196 | 0.0002554 | 11 | 0.0785714 | response to corticosteroid | 0.0191513 |
| 39 | GO:000317 | 0.000263 | 5 | 0.2083333 | heart valve morphogenesis | 0.0192128 |
| 40 | GO:005508 | 0.0002704 | 22 | 0.0486726 | cellular chemical homeostasis | 0.0192627 |
| 41 | GO:001951 | 0.0003191 | 4 | 0.3076923 | peptidyl-proline hydroxylation | 0.0221755 |
| 42 | GO:000610 | 0.0003385 | 11 | 0.0758621 | regulation of carbohydrate metabolic process | 0.0229603 |
| 43 | GO:004854 | 0.000366 | 19 | 0.0514905 | response to steroid hormone | 0.0242497 |
| 44 | GO:000193 | 0.0005256 | 7 | 0.1111111 | positive regulation of endothelial cell proliferation | 0.0333842 |
| 45 | GO:004576 | 0.0005273 | 12 | 0.0670391 | regulation of angiogenesis | 0.0333842 |

| | | | | | | |
|---|---|---|---|---|---|---|
| 46 | GO:000974 | 0.0005486 | 9 | 0.0849057 | response to glucose | 0.0336723 |
| 47 | GO:000759 | 0.0005595 | 22 | 0.0460251 | hemostasis | 0.0336723 |
| 48 | GO:003195 | 0.0005673 | 5 | 0.1724138 | regulation of protein autophosphorylation | 0.0336723 |
| 49 | GO:001812 | 0.0006192 | 4 | 0.25 | protein hydroxylation | 0.0356924 |
| 50 | GO:004232 | 0.0006264 | 17 | 0.0523077 | negative regulation of phosphorylation | 0.0356924 |
| 51 | GO:004352 | 0.0006679 | 10 | 0.075188 | negative regulation of neuron apoptotic process | 0.0373107 |
| 52 | GO:007149 | 0.0006941 | 12 | 0.0648649 | cellular response to external stimulus | 0.0380308 |
| 53 | GO:009009 | 0.0007258 | 12 | 0.0645161 | regulation of transmembrane receptor protein serine/threonine kinase signaling pathway | 0.0386429 |
| 54 | GO:000193 | 0.0007324 | 8 | 0.0909091 | regulation of endothelial cell proliferation | 0.0386429 |
| 55 | GO:000974 | 0.0007472 | 9 | 0.0810811 | response to hexose | 0.0387065 |
| 56 | GO:004472 | 0.0008415 | 9 | 0.079646 | single-organism carbohydrate catabolic process | 0.0422727 |
| 57 | GO:005087 | 0.0008458 | 5 | 0.15625 | brown fat cell differentiation | 0.0422727 |
| 58 | GO:004669 | 0.0009056 | 4 | 0.2222222 | decidualization | 0.0444827 |
| 59 | GO:003257 | 0.000958 | 5 | 0.1515152 | response to progesterone | 0.046258 |
| 60 | GO:003428 | 0.0010009 | 9 | 0.0775862 | response to monosaccharide | 0.0466345 |
| 61 | GO:004405 | 0.0010016 | 18 | 0.0485175 | regulation of system process | 0.0466345 |
| 62 | GO:000030 | 0.001017 | 10 | 0.070922 | response to reactive oxygen species | 0.0466345 |
| 63 | GO:005254 | 0.0010312 | 18 | 0.0483871 | regulation of peptidase activity | 0.0466345 |
| 64 | GO:000193 | 0.0010789 | 4 | 0.2105263 | endothelial cell proliferation | 0.0480278 |
| 65 | GO:001922 | 0.0010992 | 6 | 0.1153846 | regulation of vasoconstriction | 0.0481776 |
| 66 | GO:004254 | 0.001163 | 8 | 0.0842105 | response to hydrogen peroxide | 0.048757 |
| 67 | GO:005067 | 0.0011731 | 7 | 0.0958904 | epithelial cell proliferation | 0.048757 |
| 68 | GO:003166 | 0.001184 | 9 | 0.0756303 | cellular response to extracellular stimulus | 0.048757 |
| 69 | GO:006068 | 0.0012031 | 6 | 0.1132075 | regulation of morphogenesis of a branching structure | 0.048757 |
| 70 | GO:000620 | 0.0012043 | 3 | 0.375 | pyrimidine nucleobase catabolic process | 0.048757 |
| 71 | GO:004406 | 0.0012151 | 5 | 0.1428571 | regulation of endocrine process | 0.048757 |
| 72 | GO:001605 | 0.0012507 | 9 | 0.075 | carbohydrate catabolic process | 0.0488104 |
| 73 | GO:005092 | 0.0012507 | 9 | 0.075 | regulation of chemotaxis | 0.0488104 |
| 74 | GO:006041 | 0.0012742 | 4 | 0.2 | ventricular septum morphogenesis | 0.0490576 |

b. List of gene ontology biological process subcategories in genes up-regulated in HEB cultuered in hypoxia versus normoxia after processing with REViGO.

| Term_ID | Description | Uniqueness | Dispensability |
|---|---|---|---|
| GO:0001666 | response to hypoxia | 0.773 | 0 |
| GO:0071456 | cellular response to hypoxia | 0.665 | 0.877 |
| GO:0071453 | cellular response to oxygen levels | 0.69 | 0.874 |
| GO:0070482 | response to oxygen levels | 0.838 | 0.736 |
| GO:0036293 | response to decreased oxygen levels | 0.816 | 0.981 |
| GO:0036294 | cellular response to decreased oxygen levels | 0.686 | 0.986 |
| GO:0009611 | response to wounding | 0.852 | 0.513 |
| GO:0030198 | extracellular matrix organization | 0.871 | 0 |
| GO:0030199 | collagen fibril organization | 0.882 | 0.846 |
| GO:0001525 | angiogenesis | 0.748 | 0.02 |
| GO:0045766 | positive regulation of angiogenesis | 0.729 | 0.931 |
| GO:0045765 | regulation of angiogenesis | 0.719 | 0.857 |
| GO:0060412 | ventricular septum morphogenesis | 0.792 | 0.613 |
| GO:1901342 | regulation of vasculature development | 0.733 | 0.823 |
| GO:0003179 | heart valve morphogenesis | 0.791 | 0.616 |
| GO:0050673 | epithelial cell proliferation | 0.913 | 0.02 |
| GO:0001935 | endothelial cell proliferation | 0.903 | 0.759 |
| GO:0001936 | regulation of endothelial cell proliferation | 0.839 | 0.534 |
| GO:0001938 | positive regulation of endothelial cell proliferation | 0.84 | 0.958 |
| GO:0019511 | peptidyl-proline hydroxylation | 0.898 | 0.033 |
| GO:0071496 | cellular response to external stimulus | 0.841 | 0.077 |
| GO:0030336 | negative regulation of cell migration | 0.765 | 0.081 |
| GO:2000146 | negative regulation of cell motility | 0.763 | 0.983 |
| GO:0031668 | cellular response to extracellular stimulus | 0.767 | 0.888 |
| GO:0031667 | response to nutrient levels | 0.826 | 0.621 |
| GO:0050920 | regulation of chemotaxis | 0.653 | 0.775 |
| GO:0007411 | axon guidance | 0.482 | 0.615 |
| GO:0040013 | negative regulation of locomotion | 0.82 | 0.77 |
| GO:0009991 | response to extracellular stimulus | 0.833 | 0.656 |
| GO:0051271 | negative regulation of cellular component movement | 0.795 | 0.758 |
| GO:0097485 | neuron projection guidance | 0.482 | 0.858 |
| GO:0010035 | response to inorganic substance | 0.77 | 0.082 |
| GO:0032787 | monocarboxylic acid metabolic process | 0.826 | 0.093 |
| GO:0019471 | 4-hydroxyproline metabolic process | 0.85 | 0.553 |
| GO:0006090 | pyruvate metabolic process | 0.833 | 0.696 |
| GO:0055082 | cellular chemical homeostasis | 0.868 | 0.1 |
| GO:0007599 | hemostasis | 0.813 | 0.536 |
| GO:0090092 | regulation of transmembrane receptor protein serine/threonine kinase signaling pathway | 0.796 | 0.159 |
| GO:0060393 | regulation of pathway-restricted SMAD protein phosphorylation | 0.762 | 0.748 |
| GO:0005996 | monosaccharide metabolic process | 0.813 | 0.212 |
| GO:0044724 | single-organism carbohydrate catabolic process | 0.806 | 0.748 |
| GO:0016052 | carbohydrate catabolic process | 0.841 | 0.661 |
| GO:0006006 | glucose metabolic process | 0.812 | 0.757 |
| GO:0019318 | hexose metabolic process | 0.809 | 0.952 |
| GO:0006109 | regulation of carbohydrate metabolic process | 0.8 | 0.632 |
| GO:0006096 | glycolytic process | 0.748 | 0.953 |
| GO:0031952 | regulation of protein autophosphorylation | 0.834 | 0.273 |
| GO:0042326 | negative regulation of phosphorylation | 0.796 | 0.558 |
| GO:0018126 | protein hydroxylation | 0.898 | 0.274 |
| GO:0006208 | pyrimidine nucleobase catabolic process | 0.86 | 0.274 |
| GO:0050873 | brown fat cell differentiation | 0.83 | 0.303 |

| | | | |
|---|---|---|---|
| GO:0044057 | regulation of system process | 0.791 | 0.346 |
| GO:0044060 | regulation of endocrine process | 0.819 | 0.658 |
| GO:0019229 | regulation of vasoconstriction | 0.797 | 0.547 |
| GO:0007568 | aging | 0.847 | 0.369 |
| GO:0043062 | extracellular structure organization | 0.884 | 0.37 |
| GO:0043618 | regulation of transcription from RNA polymerase II promoter in response to stress | 0.763 | 0.373 |
| GO:0043524 | negative regulation of neuron apoptotic process | 0.838 | 0.413 |
| GO:0030099 | myeloid cell differentiation | 0.763 | 0.433 |
| GO:0043620 | regulation of DNA-templated transcription in response to stress | 0.762 | 0.45 |
| GO:0060429 | epithelium development | 0.834 | 0.459 |
| GO:0046697 | decidualization | 0.827 | 0.542 |
| GO:0010038 | response to metal ion | 0.768 | 0.462 |
| GO:0042542 | response to hydrogen peroxide | 0.72 | 0.832 |
| GO:0052547 | regulation of peptidase activity | 0.85 | 0.478 |
| GO:0060688 | regulation of morphogenesis of a branching structure | 0.762 | 0.496 |
| GO:0048545 | response to steroid hormone | 0.739 | 0.5 |
| GO:0034284 | response to monosaccharide | 0.739 | 0.952 |
| GO:0009749 | response to glucose | 0.74 | 0.528 |
| GO:0000302 | response to reactive oxygen species | 0.722 | 0.61 |
| GO:0009746 | response to hexose | 0.739 | 0.981 |
| GO:0051384 | response to glucocorticoid | 0.757 | 0.763 |
| GO:0031960 | response to corticosteroid | 0.756 | 0.772 |
| GO:0032570 | response to progesterone | 0.761 | 0.665 |

c. List of gene ontology biological process subcategories in genes down-regulated in U87-MG cultuered in hypoxia versus normoxia.

| | GOBPID | Pvalue | Count | % | GO Term | Pvalue.BH corrected |
|---|---|---|---|---|---|---|
| 1 | GO:0034660 | 4.2596E-10 | 16 | 0.04819277 | ncRNA metabolic process | 4.9624E-07 |
| 2 | GO:0016072 | 1.63309E-07 | 9 | 0.07142857 | rRNA metabolic process | 9.5127E-05 |
| 3 | GO:0034470 | 4.8074E-07 | 11 | 0.04564315 | ncRNA processing | 0.00018669 |
| 4 | GO:0006364 | 1.3484E-06 | 8 | 0.06666667 | rRNA processing | 0.00039272 |
| 5 | GO:0090501 | 2.85235E-05 | 6 | 0.06741573 | RNA phosphodiester bond hydrolysis | 0.00664597 |
| 6 | GO:0006596 | 0.00012583 | 3 | 0.23076923 | polyamine biosynthetic process | 0.02443206 |
| 7 | GO:0034661 | 0.000181695 | 3 | 0.2 | ncRNA catabolic process | 0.03023922 |

d. List of gene ontology biological process subcategories in genes up-regulated in HEB cultuered in hypoxia versus normoxia after processing with REViGO.

| Term_ID | Description | Uniqueness | Dispensability |
|---|---|---|---|
| GO:0034660 | ncRNA metabolic process | 0.288 | 0 |
| GO:0006596 | polyamine biosynthetic process | 0.471 | 0.162 |
| GO:0090501 | RNA phosphodiester bond hydrolysis | 0.32 | 0.327 |
| GO:0006364 | rRNA processing | 0.114 | 0.329 |
| GO:0016072 | rRNA metabolic process | 0.113 | 0.755 |
| GO:0034470 | ncRNA processing | 0.095 | 0.821 |
| GO:0034661 | ncRNA catabolic process | 0.156 | 0.644 |
