# Supplementary material for: Integrated analysis identified core signal pathways and hypoxic characteristics of human glioblastoma
Source: J Cell Mol Med. 2019 Jul 7;23(9):6228–37. doi: 10.1111/jcmm.14507 (PMC6714287; doi:10.1111/jcmm.14507)
Supplement: Supplementary file 17 [file JCMM-23-6228-s010.docx]

**Doc S1 Methods of the RNA-seq analyses**

**mRNA-seq library preparation and sequencing**

According to the TruSeq RNA Sample Prep Kit v2 (Illumina), we prepare the libraries follow as: mRNA isolation and fragmentation: 200ng total RNA sample was purified by oligo-dT beads, then poly (A)-containing mRNA was fragmented into small pieces with Elute, Prime, and Fragment Mix. cDNA synthesis: First-strand cDNA was generated by First Strand Master Mix and Super Script II (Invitrogen) reverse transcription(Reaction condition: 25℃ for 10 min；42℃ for 50 min；70℃for 15 min). Then, the Second Strand Master Mix was added to synthesize the second-strand cDNA (16℃ for 1 h). End repair, add A and adaptor ligation: the purified Fragmented cDNA combine with End Repair Mix, incubate at 30℃ 30min. Purify the end-repaired DNA with Ampure XP Beads (AGENCOURT). Then add A-Tailing Mix , mix well by pipetting, incubate at 37℃ for 30 min. Adapter Ligation: Combine the Adenylate 3’ Ends DNA, RNA Index Adapter and Ligation Mix, mix well by pipetting. Incubate the ligate reaction at 30℃ for 10 min. Purify the end-repaired DNA with Ampure XP Beads (AGENCOURT). RCR: Several rounds of PCR amplification with PCR Primer Cocktail and PCR Master Mix were performed to enrich the cDNA fragments. Then the PCR products are purified with Ampure XP Beads (AGENCOURT). The Qualified libraries will amplify on cBot to generate the cluster on the flowcell (TruSeq PE Cluster Kit V3–cBot–HS，Illumina). And the amplified flowcell will be sequenced pair end on the HiSeq 2000 System (TruSeq SBS KIT-HS V3, Illumina).

**Data Filtering**

We defined “dirty” reads as those containing the sequence of adaptor, high content of unknown bases, and low quality reads. They need to be removed before downstream analysis to decrease data noise. Filtering steps included 1) Remove reads with adaptors; 2) Remove reads in which unknown bases are more than 10%; 3) Remove low quality reads (the percentage of low quality bases is over 50% in a read, we define the low quality base to be the base whose sequencing quality is no more than 5). After filtering, the remaining reads are called “clean reads” and stored as FASTQ format ^1^.

**Reads Mapping**

We use Bowtie2 (version 2.2.4) ^2^ to map clean reads to reference gene and use BWA (version 0.7.10) ^3^ to reference genome. Their alignment parameters change a little according different sequencing strategy: Bowtie2 parameters for PE reads: -q --phred64 --sensitive --dpad 0 --gbar 99999999 --mp 1,1 --np 1 --score-min L,0,-0.1 -I 1 -X 1000 --no-mixed --no-discordant -p 16 -k 200

BWA parameters for PE reads: -o 1 -e 63 -i 90 -L -k 2 -l 31 -t 4 -q 10.

**Gene quantification**

RSEM (version v1.2.12) ^4^ is a quantification tool that computed maximum likelihood abundance estimates using the Expectation Maximization (EM) algorithm for its statistical model, including the modeling of paired-end (PE) and variable-length reads, fragment length distributions, and quality scores, to determine which transcripts are isoforms of the same gene. FPKM method is used to calculate expression level, and the formula is shown as below:

[
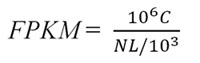
](http://xbio1.genomics.cn/NGS/report/BGI-RD-DEMO-PROJECT/BGI-RD-DEMO-PROJECT-5-online/report/resource/FPKM_formula.jpg)

According to be the expression of gene A , C is the number of fragments that are uniquely aligned to gene A , N is the number of fragments that are uniquely aligned to all genes, and L is the number of bases on gene A.

**Screening DEGs using NOISeq**

NOISeq (version 2.14.0)method ^5^ can screen differentially expressed genes between two groups, showing a good performance when comparing it to other differential expression methods, like Fisher's Exact Test (FET), edgeR, DESeq and baySeq. NOISeq maintains good True Positive and False Positive rates when increasing sequencing depth, while most other methods show poor performance. What's more, NOISeq models the noise distribution from the actual data, so it can better adapt to the size of the data set, and is more effective in controlling the rate of false discoveries.

First, NOISeq uses sample’s gene expression in each group to calculate log2 (fold change) M and absolute different value D of all pair conditions to build noise distribution model.

[
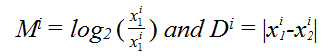
](http://xbio1.genomics.cn/NGS/report/BGI-RD-DEMO-PROJECT/BGI-RD-DEMO-PROJECT-5-online/report/resource/noiseq_formula1.png)

Second, for gene A, NOISeq computes its average expression “Control_avg” in control group and average expression “Treat_avg” in treatment group. Then the fold change (MA=log2((Control_avg)/(Treat_avg))) and absolute different value D (DA=|Congrol_avg-Treat_avg|）will be got. If MA and DA diverge from noise distribution model markedly, gene A will be defined as a DEG. There is a probability value to assess how MA and DA both diverge from noise distribution model:

[
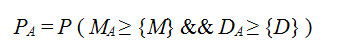
](http://xbio1.genomics.cn/NGS/report/BGI-RD-DEMO-PROJECT/BGI-RD-DEMO-PROJECT-5-online/report/resource/noiseq_formula2.png)

Finally, we screen differentially expressed genes according to the following default criteria: Fold change ≥2 and diverge probability ≥0.8.

**Gene Ontology Annotation**

To identify overrepresented GO terms (biological process subcategory, BP) in the lists of differentially expressed genes, non-conditional hypergeometric tests were performed followed by a correction for multiple testing using the procedure by Benjamini and Hochberg ^6^. GO terms with BH-adjusted P value < 0.05 were further processed using the REViGO online tool (http://revigo.irb.hr/) to remove redundant terms and determine their semantic similarity ^7^.

**KEGG Pathway Enrichment**

KEGG ^8^ (the major public pathway-related database) is used to perform pathway enrichment analysis of DEGs. This analysis identifies significantly enriched metabolic pathways or signal transduction pathways in DEGs comparing with the whole genome background.

**References:**

1. Cock PJ, Fields CJ, Goto N, et al. The Sanger FASTQ file format for sequences with quality scores, and the Solexa/Illumina FASTQ variants. Nucleic Acids Res. 2010;38:1767-1771.

2. Langmead B, Trapnell C, Pop M, et al. Ultrafast and memory-efficient alignment of short DNA sequences to the human genome. Genome Biol. 2009;10:R25.

3. Li H, Durbin R. Fast and accurate short read alignment with Burrows-Wheeler transform. Bioinformatics. 2009;25:1754-1760.

4. Li B, Dewey CN. RSEM: accurate transcript quantification from RNA-Seq data with or without a reference genome. BMC Bioinformatics. 2011;12:323.

5. Tarazona S, Garcia-Alcalde F, Dopazo J, et al. Differential expression in RNA-seq: a matter of depth. Genome Res. 2011;21:2213-2223.

6. Chen EY, Tan CM, Kou Y, et al. Enrichr: interactive and collaborative HTML5 gene list enrichment analysis tool. BMC Bioinformatics. 2013;14:128.

7. Supek F, Bosnjak M, Skunca N, et al. REVIGO summarizes and visualizes long lists of gene ontology terms. PloS One. 2011;6:e21800.

8. Kanehisa M, Araki M, Goto S, et al. KEGG for linking genomes to life and the environment. Nucleic Acids Res. 2008;36:D480-484.
